# Stereochemical Determination of Five-Membered Cyclic Ether Acetogenins Using a Spin-Spin Coupling Constant Approach and DFT Calculations

**DOI:** 10.3390/md12074031

**Published:** 2014-07-01

**Authors:** Adrián Gutiérrez-Cepeda, Antonio Hernández Daranas, José J. Fernández, Manuel Norte, María L. Souto

**Affiliations:** 1 University Institute of Bio-Organic Chemistry “Antonio González”, Center for Biomedical Research of the Canary Islands (CIBICAN), University of La Laguna, Astrofísico Francisco Sánchez 2, La Laguna 38206, Tenerife, Spain; E-Mails: aguticep@gmail.com (A.G.-C.); mnorte@ull.es (M.N.); 2 Department of Chemistry, Chemistry Institute, Sciences Faculty, Autonomous University of Santo Domingo, University City, Santo Domingo 1355, Dominican Republic; 3 Department of Chemical Engineering and Pharmaceutical Technology, University of La Laguna, La Laguna 38206, Tenerife, Spain; 4 Department of Organic Chemistry, University of La Laguna, La Laguna 38206, Tenerife, Spain

**Keywords:** C_15_ tetrahydrofuranyl-acetogenins, marilzafurollenes, marine natural products, *Laurencia*, five-membered rings, *J*-based methodologies, DFT calculations

## Abstract

Five-membered rings are of particular interest, due to their presence in some of the most common molecules in chemistry and biology. Despite their apparent simplicity, the structural resolution of these rings is complex, due to their inherent conformational flexibility. Here, we describe an application of a recently reported simple and efficient NMR protocol based on the measurement of spin-spin coupling constants to achieve the challenging relative configurations of five new halogenated C_15_ tetrahydrofuranyl-acetogenins, marilzafurollenes A–D (**1**–**4**) and 12-acetoxy-marilzafurenyne (**5**), isolated from the red alga, *Laurencia marilzae*. Although DFT chemical shift calculations were used to connect remote stereocenters, the NMR-based approach seems advantageous over computational techniques in this context, as the presence of halogens may interfere with reliable calculations.

## 1. Introduction

Marine organisms synthesize a multitude of molecules with fascinating chemical structures and potent biological properties [1]. However, to make full use of marine natural products in drug discovery, accurate structure determination is required [2,3]. For this reason, the development of effective methods to solve stereochemical problems has recently taken the limelight. In this context, new techniques based on NMR spectroscopy and/or modern computational calculations, especially DFT, is paying off [4,5,6]. Nevertheless, the elucidation of some molecular architectures, like flexible five-membered rings, are still problematic. Recently, we presented a simple and efficient spin-spin coupling constant approach designed for the stereochemical analysis of five-membered rings [7]. As a result, this usually complex problem can be easily solved in most cases by the measurement of a few coupling constants without the need for any conformational consideration.

**Figure 1:**
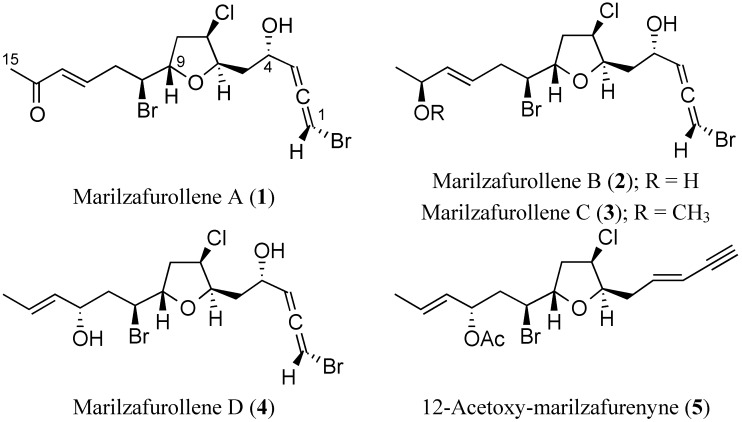
The structures of the new metabolites.

In the context of our ongoing studies of natural products from marine organisms [8,9,10], we now report the structures of five new halogenated C_15_ tetrahydrofuranyl-acetogenins (Figure 1). Marilzafurollenes A–D (**1**–**4**) along with 12-acetoxy-marilzafurenyne (**5**) were isolated from the red alga, *Laurencia marilzae* Gil-Rodríguez, Sentíes et M.T. Funjii [11], and their structures were elucidated by spectroscopic studies. Naturally occurring C_15_ tetrahydrofuranyl derivatives belong to a wider family of halogenated C_15_-acetogenins isolated from red algae of the species *Laurencia* [12], the majority of the structures of which have only partially had their relative configurations assigned or still need to be investigated to confirm or correct their reported structures [1,13,14]. The challenging relative configurations of the five-membered rings of compounds **1**–**5** were established by our simple and effective *J*-based methodology [7]. In addition, a detailed study of NMR chemical shifts by DFT calculation analysis was also undertaken with the aim of connecting remote chiralities within these tetrahydrofuranyl-acetogenins [15].

## 2. Results and Discussion

Fresh specimens of the alga, *Laurencia marilzae*, were extracted at room temperature using CH_2_Cl_2_/MeOH (1:1, *v*/*v*). The resulting extract was studied using a multi-step chromatographic fractionation sequence, including Sephadex LH-20, silica gel and normal-phase HPLC to yield compounds **1**–**5**.

Marilzafurollene A (**1**) was isolated as an optically active white amorphous solid. Its molecular formula was deduced to be C_15_H_19_Br_2_ClO_3_ by ESI-HRMS and isotopic pattern analysis of the four pseudomolecular [M + Na]^+^ ions at *m*/*z:* 462.9298, 464.9296, 466.9330 and 468.9385 (ratio: 38:100:97:42, calcd.: 462.9287, 464.9267, 466.9246 and 468.9217). From its ^13^C NMR data (Table 1), along with the analysis of the HSQC experiment, the presence of a bromoallene moiety was evident (δ_C_ 200.6 (s), 104.1 (d) and 74.9 (d)), as were two other olefinic carbon signals (δ_C_ 143.2 (d) and 133.4 (d)), five heteroatom-bearing methines (δ_C_ 80.0, 79.1, 66.4, 63.2 and 55.6), three methylenes (δ_C_ 40.9, 38.5 and 38.3), one methyl (δ_C_ 23.7), and one carbonyl carbon (δ_C_ 198.5). The ^1^H-^1^H COSY spectrum, as well as the HSQC correlations, revealed the presence of a single-spin system comprising C-3→C-13, including a double bond between C-12 and C-13, and heteroatoms located on carbons C-4, C-6, C-7, C-9 and C-10 (Table 1 and Table 2).

**Table 1:** ^13^C NMR (150 MHz) data for compounds **1**–**5** in CDCl_3_ (δ (ppm)).

C	1	2	3	4	5
1	74.9, CH	74.8, CH	74.8, CH	74.8, CH	77.0, CH
2	200.6, C	200.6, C	200.8, C	200.6, C	82.0, C
3	104.1, CH	104.1, CH	104.2, CH	104.1, CH	111.9, CH
4	66.4, CH	66.5, CH	66.6, CH	66.6, CH	140.7, CH
5	38.3, CH_2_	38.1, CH_2_	38.2, CH_2_	38.2, CH_2_	35.0, CH_2_
6	80.0, CH	79.7, CH	79.8, CH	79.8, CH	82.1, CH
7	63.2, CH	63.3, CH	63.4, CH	63.4, CH	62.3, CH
8	40.9, CH_2_	40.7, CH_2_	40.9, CH_2_	41.1, CH_2_	41.0, CH_2_
9	79.1, CH	79.1, CH	79.1, CH	80.0, CH	79.6, CH
10	55.6, CH	57.3, CH	57.9, CH	56.2, CH	54.8, CH
11	38.5, CH_2_	38.5, CH_2_	38.4, CH_2_	43.1, CH_2_	40.8, CH_2_
12	143.2, CH	126.2, CH	128.5, CH	70.4, CH	72.8, CH
13	133.4, CH	137.6, CH	135.8, CH	133.3, CH	128.7, CH
14	198.5, C	68.5, CH	77.7, CH	127.5, CH	130.0, CH
15	27.3, CH_3_	23.4, CH_3_	21.3, CH_3_	17.7, CH_3_	17.8, CH_3_
OCH_3_			56.0, CH_3_		
CO(Ac)					170.2, C
CH_3_(Ac)					21.3, CH_3_

**Table 2:** ^1^H NMR (600 MHz) data for marilzafurollenes A–C (**1**–**3**) in CDCl_3_ (δ (ppm)).

C	Marilzafurollene A (1)	Marilzafurollene B (2)	Marilzafurollene C (3)
1	6.13, dd (2.2, 5.6)	6.13, dd (2.2, 5.7)	6.13, ddd (1.5, 2.2, 5.6)
3	5.52, dd (5.6, 5.6)	5.52, dd (5.5, 5.7)	5.53, dd (5.6, 5.6)
4	4.56, ddd (3.6, 5.6, 8.2)	4.57, ddd (3.6, 5.5, 7.7)	4.57, ddd (3.4, 5.6, 8.0)
5	2.13, ddd (3.6, 8.8, 14.4)	2.15, ddd (3.6, 9.1, 14.5)	2.14, ddd (3.4, 8.7, 14.0)
	1.88, ddd (3.6, 8.2, 14.4)	1.86, ddd (3.5, 7.7, 14.5)	1.87, ddd (3.1, 8.0, 14.0)
6	4.48, ddd (3.0, 3.6, 8.8)	4.46, ddd (3.3, 3.5, 9.1)	4.46, ddd (2.5, 3.1, 8.7)
7	4.55, ddd (0.8, 3.0, 4.8)	4.53, ddd (0.8, 3.3, 4.8)	4.54, ddd (2.5, 3.4, 4.5)
8	α 2.55, ddd (4.8, 9.6, 13.9)	α 2.52, ddd (4.8, 9.6, 13.9)	α 2.52, ddd (4.5, 8.9, 14.1)
	β 2.42, ddd (0.8, 6.2, 13.9)	β 2.39, ddd (0.8, 6.2, 13.9)	β 2.39, ddd (3.4, 6.3, 14.1)
9	4.46, ddd (3.0, 6.2, 9.6)	4.47, ddd (3.1, 6.2, 9.6)	4.47, ddd (3.4, 6.3, 8.3)
10	4.10, ddd (3.0, 5.2, 8.5)	4.06, ddd (3.1, 5.7, 7.9)	4.03, ddd (3.4, 4.6, 8.6)
11	2.90, m (2H)	2.70, m (2H)	2.71, m (2H)
12	6.83, ddd (7.0, 7.0, 15.9)	5.72, ddd (6.4, 7.0, 15.6)	5.67, ddd (7.0, 7.2, 15.5)
13	6.18, br d (15.9)	5.65, br dd (6.1, 15.6)	5.48, dddd (1.5, 1.5, 6.8, 15.5)
14		4.30, dd (6.1, 6.3)	3.72, dd (6.6, 6.8)
15	2.28, s (3H)	1.28, d (6.3) (3H)	1.24, d (6.5) (3H)
OCH_3_			3.28, s (3H)

HMBC cross-peaks from H-6 (δ_H_ 4.48) to C-9 (δ_C_ 79.1) established an ether linkage between these positions, indicating the presence of a tetrahydrofuran ring in the molecule and, therefore, placed the remaining hydroxy group at C-4. Moreover, the HMBC correlations from the vinyl proton H-13 (δ_H_ 6.18) and the methyl singlet (δ_H_ 2.28) to the ketone carbon C-14 (δ_C_ 198.5) completed the planar structure of **1**.

**Figure 2:**
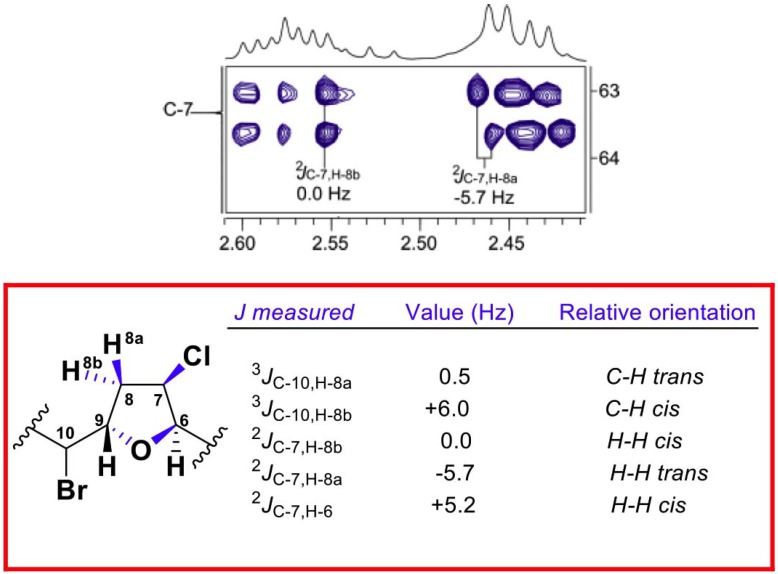
Representative section of the HSQC-HECADE spectrum (600 MHz, CDCl_3_, see Supplementary Figure S7), calculated *J*_C,H_ values and configuration analysis for the oxolane ring of marilzafurollene A (**1**).

The stereochemical relationships between the different chiral centers of **1** were mostly based on the analysis of homo- and hetero-nuclear *J* couplings. Thus, the *^n^J*_C,H_ values were accurately measured using the HSQC-HECADE (Heteronuclear couplings from ASSCI-Domain Experiments with E.COSY-type cross peaks) experiment. The value of the coupling constant between H-12 and H-13 (^3^*J*_H-12,H-13_ = 15.9 Hz) indicated the *E* geometry for the double bond. The relative configuration of the oxolane ring was solved by using the NMR-based approach developed by our research group. Therefore, the relative *cis* orientation between H-6 and H-7 was deduced from the ^2^*J*_C,H_ value of 5.2 Hz for H-6/C-7, while a ^2^*J*_C,H_ value of −5.7 Hz for one of the diastereotopic H-8 methylene protons (δ_H_ 2.42) and C-7 suggested a *trans* orientation between H-7 and H-8a. The relative configuration of the C-9 stereocenter was determined by evaluating the relationship between methine H-10 and these stereospecifically defined H8-methylene protons. In this case, a small value for ^3^*J*_C-10,H-8a_ (0.5 Hz) and a large coupling constant for ^3^*J*_C-10,H-8b_ (6.0 Hz) were consistent with a *cis* configuration for H-8a and H-9 (Figure 2).

**Figure 3:**
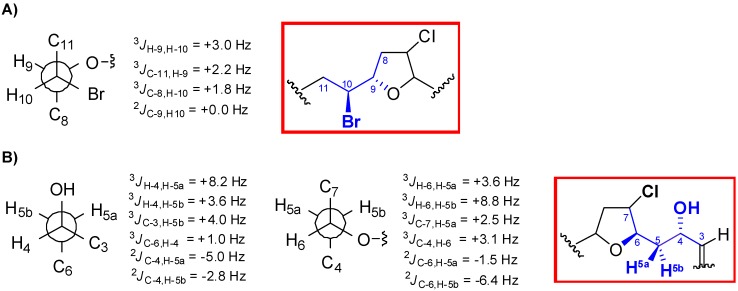
*J*-based configuration analysis for the (**A**) C-9/C-10 and (**B**) C-4/C-6 fragments of marilzafurollene A (**1**).

In order to complete the structural determination of **1**, the relative configurations for the 1,3-methine system C-4/C-6, as well as for the C-9/C-10 segment were determined via *J*-based configuration analysis (Figure 3) [16]. Accordingly, based on the observed homo- and hetero-nuclear *J* couplings, H-4 was found to be *erythro* to H-5b (δ_H_ 2.13) and H-6 *threo* to H-5a (δ_H_ 1.88). Similarly, our data were consistent with a *threo* relationship between H-9 and H-10. Therefore, the relative configuration within the C-3→C-13 moiety of **1** was determined to be 4S*,6R*,7R*,9S*,10S*. Finally, the intensive positive rotation of **1**, as well as the existence of a positive Cotton effect, enabled the absolute configuration of the bromoallene moiety to be assigned as *S* [17,18,19,20].

**Figure 4:**
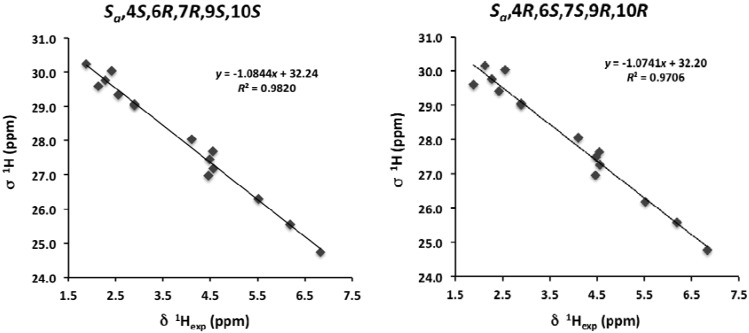
^1^H correlations between calculated isotropic shieldings and experimentally observed chemical shifts for the two studied diastereoisomers of marilzafurollene A (**1**). Fitting parameters are indicated.

The last step in the elucidation of the stereochemical relationships of **1** was the connection between the configurations of the bromoallene and the C-4/C-10 stereocluster. We approached this task using quantum mechanical calculations of theoretical NMR chemical shifts that have also been shown to be effective in complex marine natural products [21]. Thus, taking into account that the allene configuration can be considered as absolute, we built models of the two possible diastereoisomers (*S_a_*,4*S*,6*R*,7*R*,9*S*,10*S* (diastereoisomer **1a**) and *S_a_*,4*R*,6*S*,7*S*,9*R*,10*R* (diastereoisomer **1b**)) and performed conformational searches on each one using 5000 steps of a hybrid MCMM (Monte Carlo Multiple Minimum), Low-Mode sampling and the MMFF94 (Merck Molecular Force Field) force field. Redundant conformers were eliminated using an RMSD cutoff of 1.0 Å. All the resulting structures within an energy window of 10 kJ/mol of the global minimum found (69 conformers for **1a** and 54 for **1b**) were further submitted to density functional theory (DFT) calculations [22]. Due to the existence of bromine in this molecule, we used the B3LYP (Becke three-parameter Lee-Yang-Parr exchange functional) functional with the LACVP ** basis set to calculate the isotropic chemical shieldings and relative energy values for each conformer [23]. Still, as expected, calculations for those carbon atoms attached to bromine showed higher than average errors; thus, their values were not included in the subsequent analysis. Fortunately, this heavy atom effect is very local and does not significantly affect nearby atoms [5]. Although the experimental NMR data were obtained using chloroform as the solvent, all calculations were performed *in vacuo*, as this has been shown to be a valid approach [21,22,24]. Finally, we estimated average chemical shift values according to the relative Boltzmann population of each conformer (NMR calculations were performed for all conformers within the selected 10-kJ/mol threshold). The result was that the correlations obtained after the linear regression of those calculated against the experimental values were almost identical for the ^13^C chemical shifts (*R*^2^ 0.9957 *vs.* 0.9956), but slightly better for the *S_a_*,4*S*,6*R*,7*R*,9*S*,10*S* diastereoisomer using the ^1^H chemical shifts (*R*^2^ 0.9820 *vs.* 0.9706) (Figure 4, Supplementary Figure S19 and Table S2). Moreover, we also used the computed chemical shift values to calculate the so-called DP4 parameter, which found the *S_a_*,4*S*,6*R*,7*R*,9*S*,10*S* isomer as the most likely solution, with a probability value of 99.7% [24]. Despite this kind of solution, based on theoretical calculations, having a degree of uncertainty, the fact that it is in accordance with the biogenetic hypothesis proposed below further supports it.

The ESI-HRMS spectrum of marilzafurollene B (**2**) established a molecular formula of C_15_H_21_Br_2_ClO_3_ (*m*/*z* calcd. for [M + Na]^+^ 464.9444, 466.9423, 468.9403, 470.9373; found 464.9453, 466.9432, 468.9418, 470.9395). A detailed comparison of the ^1^H and ^13^C NMR data of compounds **1** and **2** (Table 1 and Table 2) revealed a great similarity in their structures and suggested that compound **2** contained an additional hydroxy group (δ_C_ 68.5, δ_H_ 4.30, dd, *J* = 6.1, 6.3 Hz) instead of the carbonyl group. This was also supported by the HMBC and ^1^H-^1^H COSY analyses.

Marilzafurollene C (**3**), albeit unstable (see the Experimental Section), was shown to have the formula C_16_H_23_Br_2_ClO_3_; thus, the ^1^H NMR spectrum of **3** was nearly identical to that of **2**, except for the presence of the *O*-methyl group at δ_H_ 3.28 in **3** and the relative upfield shift of H-14 (δ_H_ 4.30 in **2** *vs*. δ_H_ 3.72 in **3**) (Table 2). These changes indicate that a methoxy group replaces the hydroxy group at C-14 in **3**. This was further confirmed by observation of the ^13^C NMR spectrum (an additional methyl peak appeared at δ_C_ 56.0 ppm, and C-14 was significantly deshielded in **3** compared to **2**). In order to solve the relative configuration of the remote stereocenter at C-14, we calculated ^1^H and ^13^C chemical shifts of the two possible diastereoisomers at the DFT level as a diagnostic tool. According to our results, the 4S*,6R*,7R*,9S*,10S*,14S* diastereoisomer showed a slightly better match with the experimental data (*R*^2^ 0.971 *vs.* 0.968 using δ ^1^H) and a DP4 probability of 98.7% (Supplementary Table S3 and Figure S20).

**Figure 5:**
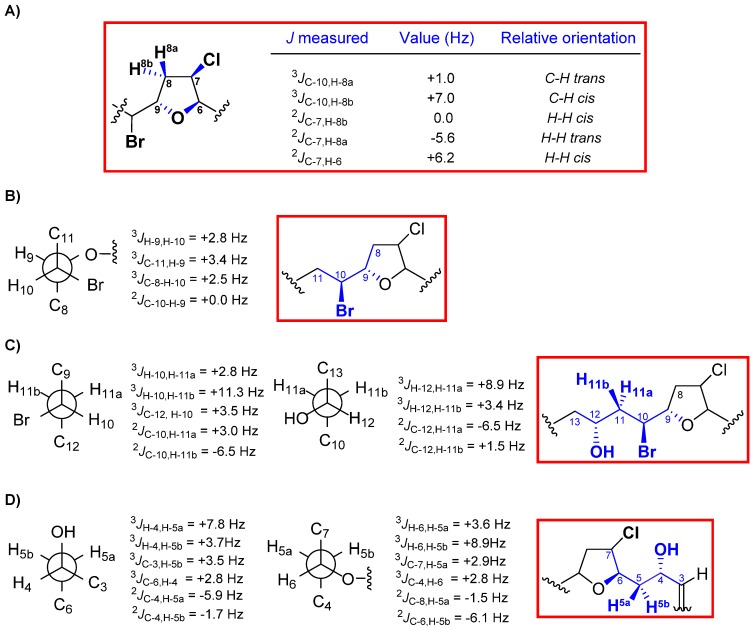
(**A**) Configuration analysis for the oxolane ring; (**B**) C-9/C-10; (**C**) C-10/C-12; and (**D**) C-4/C-6 fragments of marilzafurollene D (**4**).

Marilzafurollene D (**4**) was analyzed for the same molecular formula as **2**, C_15_H_21_Br_2_ClO_3_, and showed similar spectral features to those of **2** (Table 1 and Table 3). A comparison of the spectroscopic data clearly showed that compounds **2** and **4** were structural isomers. Thus, the analysis of the ^1^H-^1^H COSY correlations indicated the position of the *E* double bond to be between C-13–C-14, as well as the hydroxy group at C-12 in **4**. Furthermore, chemical shift differences between **2** and **4** were observed for H-9 and particularly for H-10, while values observed for H-4, H_2_-5, H-6, H-7 and H_2_-8 remained virtually the same. Therefore, we thought that these variations could be explained either by the proximity of the hydroxy group at C-12 or by a change in the relative configurations of the carbon atoms. Again, an NMR configurational analysis performed using the above-described methods provided conclusive proof of the relative configurations at all the stereogenic centers of the molecule. This time, because of the overlapping ^1^H NMR signals observed in CDCl_3_, a different solvent (C_6_D_6_) was also used to record the experimental data (see Supplementary Information). The results of the NMR measurements are shown in Figure 5 and the conclusion was that the relative configurations of C-4, C-6, C-7, C-9 and C-10 were identical to those of compounds **1**–**3**, whereas the relative configuration of the new chiral center at C-12 was assigned as 12S* (Figure 5C). Again, the absolute configuration of the bromoallene moiety was also established as *S* based on the observation of a positive Cotton effect [17].

**Table 3:** ^1^H NMR (600 MHz) data for compounds **4** and **5** in CDCl_3_ (δ (ppm)).

C	Marilzafurollene D (4)	12-Acetoxy-marilzafurenyne (5)
1	6.12, dd (2.1, 5.6)	2.84, br d (1.7)
3	5.52, dd (5.6, 5.6)	5.64, dd (1.7, 16.1)
4	4.57, ddd (3.7, 5.6, 7.8)	6.20, ddd (7.4, 7.4, 16.1)
5	2.15, ddd (3.7, 8.9, 14.3)	2.60, ddd (6.8, 7.4, 14.7)
	1.87, ddd (3.6, 7.8, 14.3)	2.50, ddd (6.8, 7.4, 14.7)
6	4.47, ddd (3.4, 3.6, 8.9)	4.19, ddd (2.8, 6.8, 6.8)
7	4.54, dd (3.4, 4.5)	4.49, dd (2.8, 4.8)
8	α 2.56, ddd (4.5, 9.5, 13.9)	α 2.56, ddd (4.8, 9.8, 13.9)
	β 2.40, dd (6.2, 13.9)	β 2.38, dd (6.1, 13.9)
9	4.42, ddd (2.8, 6.2, 9.5)	4.39 ddd (2.5, 6.1, 9.8)
10	4.35, ddd (2.8, 2.8, 11.3)	4.05 ddd (2.5, 3.1, 10.7)
11	2.09, ddd (3.4, 11.3, 15.0)	2.22, ddd (3.3, 10.7, 14.3)
	1.91, ddd (2.8, 8.9, 15.0)	2.17, ddd (3.1, 9.8, 14.3)
12	4.41, ddd (3.4, 6.7, 8.9)	5.48, ddd (3.3, 7.0, 9.8)
13	5.54, br dd (6.7, 15.2)	5.43, br dd (7.0, 15.1)
14	5.74, dq (6.4, 15.2)	5.80, dq (6.5, 15.1)
15	1.70, d (6.4) (3H)	1.69, br d (6.5) (3H)
CH_3_(Ac)		2.05, s (3H)

12-Acetoxy-marilzafurenyne (**5**) has the molecular formula C_17_H_22_BrClO_3_, as deduced from mass spectral data (ESI-HRMS ions at *m*/*z* 411.0332, 413.0320, 415.0298; ([M + Na]^+^). The IR spectrum revealed absorption bands at 3293, 2326 (terminal alkyne moiety), 1733 (ester carbonyl group), 1645 (double bond) and 1050 (ether functionality) cm^−1^. The ^13^C NMR data of compound **5** (Table 1) exhibited signals for 17 carbons corresponding to two quaternary carbons, ten methines, three methylenes and two methyl groups. Among these carbons, one was assigned as a carbonyl, two were halogenated, three were bonded to oxygen and four were olefinic. The presence of a terminal enyne moiety was evident from the tertiary carbon resonances at δ_C_ 77.0, 111.9 and 140.7 and the quaternary carbon at δ_C_ 82.0. Furthermore, the ^1^H NMR spectrum (Table 2) showed signals at δ_H_ 2.84 (1H, br d, *J* = 1.7 Hz), 5.64 (1H, dd, *J* = 1.7, 16.1 Hz) and 6.20 (1H, ddd, *J* = 7.4, 7.4, 16.1 Hz), supporting the presence of the *E*-enyne unit. A detailed study of the 1D and 2D NMR data of compound **5** compared with those of compound **4** concluded that both compounds possessed a similar structure, but with the significant difference of the conjugated terminal enyne functionality instead of a bromoallene unit in **5**. In addition, the ^1^H NMR spectrum of **5** included a signal for an additional acetate methyl group (δ_H_ 2.05 (s)) and a new deshielded oxygenated methine (δ_H_ 5.48 (ddd, *J* = 3.3, 7.0, 9.8 Hz)) that bears it. The acetate functionality present in the molecule was placed at C-12 on the basis of HMBC NMR cross-peaks observed between the methine and methylene protons, H-12 and H_2_-11, and the corresponding carbonyl ester carbon (Table 1).

The relative configuration of the C-6→C-10 moiety was assigned as identical to that of Molecules **1**–**4** on the basis of the observed similarity in their ^3^*J*_HH_ and chemical shift values (Table 3). One more time, the relative configuration at C-12 was studied using the ^1^H and ^13^C chemical shift DFT calculations. Thus, the calculated values for the 4S*,6R*,7R*,9S*,10S*,12S* diastereoisomer showed a better match with the experimental data (*R*^2^ 0.986 *vs*. 0.965 for δ ^1^H) and a DP4 probability of 100% (Supplementary Table S4 and Figure S21).

**Scheme 1:**
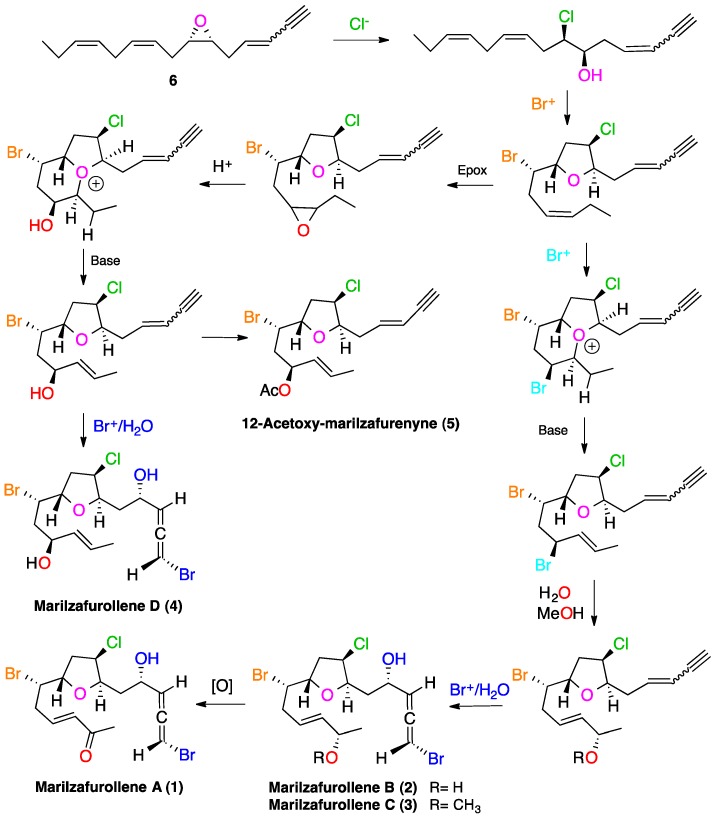
Suggested biogenesis of marilzafurollenes A–D (**1**–**4**) and 12-acetoxy-marilzafurenyne (**5**).

The biogenesis of the large family of halogenated cyclic acetogenins, all functionalized at C-6, C-7, C-9, C-10, C-12 and C-13, has been suggested by Murai as ultimately arising from (*Z*,*Z*,*Z*)-hexadeca-4,7,10,13-tetraenoic acid via (*Z*)-6,7-epoxide **6** (or a closely related precursor) and electrophilic bromination events [25]. Based on this work, Braddock recently proposed a hypothesis concerning the biosynthesis of an interesting subset of these halogenated C_15_ acetogenins, the obtusallenes [26], whose initial steps could explain the biogenetic origin of compounds **1**–**5**. Thus, it seems reasonable to suggest that these compounds derive from epoxide **6** by nucleophilic ring-opening with chloride to provide a *threo*-hydroxychloride derivative (Scheme 1). Subsequent bromoetherification gives a *trans*-tetrahydrofuran intermediate that evolves to generate bicyclic oxonium ions, which, in turn, can be fragmented to give compounds **4**–**5** and the allylic precursors of compounds **1**–**3**. Lastly, the terminal bromoallene moiety in compounds **1**–**4** may be produced biosynthetically by bromonium ion formation on the terminal enyne, followed by nucleophilic attack of water. Finally, it has to be noted that the configurations proposed by us for these molecules (**1**–**5**) are consistent with this biogenetic proposal.

## 3. Experimental Section

### 3.1. General Experimental Procedures

Optical rotations were measured at room temperature in CHCl_3_ using a sodium lamp. IR spectra were recorded using methanolic solutions over a NaCl disk. NMR spectra were recorded on a 600 MHz equipped with a 5-mm TCI (Triple Resonance CryoProbe) inverse detection cryo-probe. ^1^H and ^13^C NMR chemical shifts were referenced either to the CDCl_3_ or C_6_D_6_ solvent peaks at 300 K (CDCl_3_: δ_H_ 7.26, δ_C_ 77.0; C_6_D_6_: δ_H_ 7.16, δ_C_ 128.4). COSY, HSQC, HMBC and ROESY experiments were performed using standard pulse sequences. ^3^*J*_H,H_ values were measured from 1D ^1^H NMR. The HSQC-HECADE pulse sequence was used to measure long-range heteronuclear coupling constants. All experiments were performed in the phase-sensitive mode (States-TPPI (Time-Proportional Phase-Incrementation frequency discrimination) or echo-antiecho for quadrature detection in F1) and used gradient coherence selection. The HSQC-HECADE experiment was recorded using DIPSI during the 40 ms of the isotropic mixing period using a bandwidth of 10 kHz, and a *J*-scale factor of **1** was used. Prior to Fourier transformation, zero filling was performed to expand the data to at least double the number of acquired data points. HPLC separations were carried out with a preparative silica column (10 μ, 19 × 150 mm) and a photodiode array detector. TLC was visualized by spraying with phosphomolybdic acid reagent (10% in EtOH) and heating.

### 3.2. Computational Methods

Conformational searches were performed using the Macromodel software (version 8.5, Schrödinger Inc., San Diego, CA, USA) and the MMFF94 force field. Solvation effects were simulated using the generalized Born/surface area (GBSA) solvation model with chloroform. Extended non-bonded cutoff distances (a van der Waals cutoff of 8.0 Å and an electrostatic cutoff of 20.0 Å) were used. Local minima within 10 kJ of the global minimum were saved. Analysis of the results was undertaken using Maestro software.

Quantum mechanical calculations were carried out with the Jaguar package (Jaguar; Schrödinger LLC, New York, NY, USA). Single-point energy calculations were performed at the DFT theoretical level in the gas phase. The B3LYP hybrid functional with the LACVP ** basis set was used. Chemical shifts were calculated using the gauge-including atomic orbital (GIAO) method. Chemical shifts were calculated from their shielding constants that were first averaged according to their relative Boltzmann populations using a Schrödinger Inc. python script. Proton chemical shifts for each methyl group were averaged due to their conformational freedom.

### 3.3. Biological Material

Specimens of *Laurencia marilzae* Gil-Rodríguez, Sentíes et M.T. Funjii [11], were collected by hand in the intertidal zone at Paraíso Floral (Tenerife, Canary Islands, Spain). A voucher specimen was deposited at the Department of Biología Vegetal, Botánica, University of La Laguna, Tenerife (TFC Phyc 9860 (Herbarium code of University of La Laguna)).

### 3.4. Extraction and Isolation

Fresh alga (1.3 kg) was extracted with CH_2_Cl_2_:MeOH (1:1, *v*/*v*) at room temperature and the solvent removed *in vacuo* to give a dark-green viscous oil (42.9 g). The extract was subjected to Sephadex LH-20 (*n*-Hex:CH_2_Cl_2_:MeOH (2:1:1)) column chromatography. Selected fractions exhibiting similar TLC profiles were rechromatographed on a medium-pressure normal-phase chromatography using a Lobar LiChroprep Si 60 column with *n*-hexane:EtOAc (4:1). Final purifications were achieved on a μ-Porasil HPLC column, 10 μ, 19 × 150 mm, using *n*-hexane:EtOAc (9:1 and 7:3), yielding compounds **1** (1.8 mg), **2** (2.2 mg), **3** (1.2 mg), **4** (0.8 mg) and **5** (1.0 mg).

Marilzafurollene A (**1**): white, amorphous substance; [α]^D^_25_ +32 (*c* 0.06, CHCl_3_); UV (MeOH) λ_max_ (logε) 205 (3.58) nm; CD (CH_3_CN): λ_max_ (Δε) 217 (+0.46) nm; IR (CHCl_3_) ν_max_ 3439, 3060, 2928, 2859, 1962, 1729, 1674, 1663, 1447, 1368, 1263, 1072 cm^−1^; ^1^H and ^13^C NMR data (CDCl_3_), see Table 1 and Table 2; ESI-HRMS *m*/*z* 462.9298, 464.9296, 466.9330, 468.9385 [M + Na]^+^ (38:100:97:42) (calcd. for C_15_H_19_^79^Br_2_^35^ClO_3_Na, 462.9287; C_15_H_19_^79^Br^81^Br^35^ClO_3_Na, 464.9267; C_15_H_19_^81^Br_2_^35^ClO_3_Na, 466.9246; C_15_H_19_^81^Br_2_^37^ClO_3_Na, 468.9217).

Marilzafurollene B (**2**): white, amorphous substance; [α]^D^_25_ +60 (*c* 0.10, CHCl_3_); UV (MeOH) λ_max_ (logε) 203 (3.49) nm; CD (CH_3_CN): λ_max_ (Δε) 221 (+0.67) nm; IR (CHCl_3_) ν_max_ 3413, 2965, 2930, 1962, 1724, 1634, 1444, 1376, 1266, 1194, 1065 cm^−1^; ^1^H and ^13^C NMR data (CDCl_3_), see Table 1 and Table 2; ESI-HRMS *m*/*z* 464.9453, 466.9432, 468.9418, 470.9395 [M + Na]^+^ (46:100:71:15) (calcd. for C_15_H_21_^79^Br_2_^35^ClO_3_Na, 464.9444; C_15_H_21_^79^Br^81^Br^35^ClO_3_Na, 466.9423; C_15_H_21_^81^Br_2_^35^ClO_3_Na, 468.9403; C_15_H_21_^81^Br_2_^37^ClO_3_Na, 470.9373).

Marilzafurollene C (**3**): white, amorphous substance; ^1^H and ^13^C NMR data (CDCl_3_), see Table 1 and Table 2. Complementary spectroscopic data are not available due to the fast degradation of the sample.

Marilzafurollene D (**4**): white, amorphous substance; [α]^D^_25_ +22 (*c* 0.08, CHCl_3_); UV (MeOH) λ_max_ (logε) 204 (3.52) nm; CD (CH_3_CN): λ_max_ (Δε) 218 (+0.62) nm; IR ν_max_ (CHCl_3_) 3413, 2965, 2856, 1962, 1724, 1634, 1444, 1376, 1266, 1194, 1065 cm^−1^; ^1^H and ^13^C NMR data (CDCl_3_), see Table 1 and Table 3; ^1^H and ^13^C NMR data (C_6_D_6_), see Supplementary Table S1; ESI-HRMS *m*/*z* 464.9445, 466.9428, 468.9416, 470.9414 [M + Na]^+^ (49:100:74:12) (calcd. for C_15_H_21_^79^Br_2_^35^ClO_3_Na, 464.9444; C_15_H_21_^79^Br^81^Br^35^ClO_3_Na, 466.9423; C_15_H_21_^79^Br^81^Br^37^ClO_3_Na, 468.9394; C_15_H_21_^81^Br_2_^37^ClO_3_Na, 470.9373).

12-Acetoxy-marilzafurenyne (**5**): white, amorphous substance; [α]^D^_25_ −13 (*c* 0.07, CHCl_3_); UV (MeOH) λ_max_ (logε) 225 (2.79) nm; IR ν_max_ (CHCl_3_) 3293, 2926, 2326, 1960, 1733, 1645, 1378, 1259, 1188, 1050 cm^−1^; ^1^H and ^13^C NMR data (CDCl_3_), see Table 1 and Table 3; ESI-HRMS *m*/*z* 411.0332, 413.0320, 415.0298 [M + Na]^+^ (77:100:26) (calcd. for C_17_H_22_^79^Br^35^ClO_3_Na, 411.0339, C_17_H_22_^81^Br^35^ClO_3_Na, 413.0318, C_17_H_22_^79^Br^37^ClO_3_Na, 413.0309, C_17_H_22_^81^Br^37^ClO_3_Na, 415.0289).

## 4. Conclusions

We have demonstrated the value of our simple and efficient NMR protocol based on the measurement of spin-spin coupling constants to achieve the challenging relative configurations of five new halogenated C_15_ tetrahydrofuranyl-acetogenins isolated from indigenous species of *Laurencia* (*Laurencia marilzae*). Furthermore, a detailed study of NMR chemical shifts by DFT calculations was also undertaken with the aim of connecting remote chiralities within these tetrahydrofuranyl-acetogenins. Inspection of the isolated structures also provides new insights into the biosynthetic pathway of this class of compounds.

## Supplementary Files

Supplementary File 1 Supplementary Information (PDF, 9065 KB)
