# Supplementary material for: Stereochemical Determination of Five-Membered Cyclic Ether Acetogenins Using a Spin-Spin Coupling Constant Approach and DFT Calculations
Source: Mar Drugs. 2014 Jul 1;12(7):4031–44. doi: 10.3390/md12074031 (PMC4113813; doi:10.3390/md12074031)
Supplement: Supplementary File 1 — Supplementary Information (PDF, 9065 KB) [file marinedrugs-12-04031-s001.pdf]

Supplementary Information

Table S1. ^{1}H (600 MHz), and ^{13}C NMR (150 MHz) spectroscopic data of **4** in C_6D_6. S2
Figure S1. ^{1}H NMR spectrum (600 MHz, $CDCl_3$) of marilzafurollene A (**1**). S3
Figure S2. ^{13}C NMR spectrum (150 MHz, $CDCl_3$) of compound **1**. S4
Figure S3. ^{1}H-^{1}H COSY NMR spectrum (600 MHz, $CDCl_3$) of compound **1**. S5
Figure S4. HSQC NMR spectrum (600 MHz, $CDCl_3$) of compound **1**. S6
Figure S5. HMBC NMR spectrum (600 MHz, $CDCl_3$) of compound **1**. S7
Figure S6. ROESY NMR spectrum (600 MHz, $CDCl_3$) of compound **1**. S8
Figure S7. Selected sections of the HSQC-HECADE NMR spectrum (600 MHz, $CDCl_3$) of compound **1**. S9
Figure S8. ^{1}H NMR spectrum (600 MHz, $CDCl_3$) of marilzafurollene B (**2**). S10
Figure S9. ^{13}C NMR spectrum (150 MHz, $CDCl_3$) of compound **2**. S11
Figure S10. ^{1}H NMR spectrum (600 MHz, $CDCl_3$) of marilzafurollene C (**3**). S12
Figure S11. ^{13}C NMR spectrum (150 MHz, $CDCl_3$) of compound **3**. S13
Figure S12. ^{1}H NMR spectrum (600 MHz, $CDCl_3$) of marilzafurollene D (**4**). S14
Figure S13. ^{13}C NMR spectrum (150 MHz, $CDCl_3$) of compound **4**. S15
Figure S14. ^{1}H NMR spectrum (600 MHz, C_6D_6) of compound **4**. S16
Figure S15. HSQC NMR spectrum (600 MHz, C_6D_6) of compound **4**. S17
Figure S16. Selected sections of the HSQC-HECADE NMR spectrum (600 MHz, C_6D_6) of compound **4**. S18
Figure S17. ^{1}H NMR spectrum (600 MHz, $CDCl_3$) of 12-acetoxy-marilzafurenyne (**5**). S19
Figure S18. ^{13}C NMR spectrum (150 MHz, $CDCl_3$) of compound **5**. S20
Table S2. Calculated ^{13}C and ^{1}H chemical shifts for marilzafurollene A (**1**). S21
Figure S19. Correlation plots obtained for marilzafurollene A (**1**). S21
Table S3. Calculated ^{13}C and ^{1}H chemical shifts for marilzafurollene C (**3**). S22
Figure S20. Correlation plots obtained for marilzafurollene C (**3**). S23
Table S4. Calculated ^{13}C and ^{1}H chemical shifts 12-acetoxy-marilzafurenyne (**5**). S23
Figure S21. Correlation plots obtained for 12-acetoxy-marilzafurenyne (**5**). S24
Table S5. Calculated Energies (Hartrees) for diastereoisomer S_a,4*S*,6*R*,7*R*,9*S*,10*S* of **1**. S25
Table S6. Coordinates (Angstroms) of calculated geometries for diastereoisomer S_a,4*S*,6*R*,7*R*,9*S*,10*S* of **1**. S27
Table S7. Calculated Energies (Hartrees) for diastereoisomer S_a,4*R*,6*S*,7*S*,9*R*,10*R* of **1**. S96
Table S8. Coordinates (Angstroms) of calculated geometries for diastereoisomer S_a,4*R*,6*S*,7*S*,9*R*,10*R* of **1**. S97
Table S9. Calculated Energies (Hartrees) for diastereoisomer 4*S**,6*R**,7*R**,9*S**,10*S**,14*S** of **3**. S144
Table S10. Coordinates (Angstroms) of calculated geometries for diastereoisomer 4*S**,6*R**,7*R**,9*S**,10*S**,14*S** of **3**. S148
Table S11. Calculated Energies (Hartrees) for diastereoisomer 4*S**,6*R**,7*R**,9*S**,10*S**,14*R** of **3**. S330

Table S12. Coordinates (Angstroms) of calculated geometries for diastereoisomer 4*S**,6*R**,7*R**,9*S**,10*S**,14*R** of **3**. S331
Table S13. Calculated Energies (Hartrees) for diastereoisomer 4*S**,6*R**,7*R**,9*S**,10*S**,12*S** of **5**. S393
Table S14. Coordinates (Angstroms) of calculated geometries for diastereoisomer 4*S**,6*R**,7*R**,9*S**,10*S**,12*S** of **5**. S393
Table S15. Calculated Energies (Hartrees) for diastereoisomer 4*S**,6*R**,7*R**,9*S**,10*S**,12*R** of **5**. S408
Table S16. Coordinates (Angstroms) of calculated geometries for diastereoisomer 4*S**,6*R**,7*R**,9*S**,10*S**,12*R** of **5**. S408

Table S1. ^{1}H (600 MHz), and ^{13}C NMR (150 MHz) spectroscopic data of **4** in C_6D_6.

No.	Marilzafurollene D (4) [a]	
	**δ_C Mult.**	**δ_H (*J* in Hz)**
1	74.4, CH	5.67, dd (2.0, 5.3)
2	200.2, C	
3	103.9, CH	5.02, dd (5.3, 5.6)
4	66.0, CH	4.23, m
5	38.6, CH_2	b 1.99, m<br>a 1.58, ddd (3.5, 8.3, 14.0)
6	79.3, CH	4.17, ddd (3.1, 3.5, 8.9)
7	63.6, CH	3.85, br dd (3.1, 5.0)
8	40.6, CH_2	b 2.04, ddd (5.0, 9.8, 13.8)<br>a 1.85, dd (6.3, 13.8)
9	79.7, CH	4.14, ddd (2.8, 6.3, 9.8)
10	56.6, CH	4.27, m
11	42.9, CH_2	b 2.00, ddd (2.6, 11.4, 14.3)<br>a 1.66, m
12	69.9, CH	4.29, m
13	133.4, CH	5.30, br dd (6.4, 15.3)
14	125.3, C	5.40, dq (6.3, 15.3)
15	17.3, CH_3	1.48, d, (6.3)

[a] Data recorded at 600/150 MHz (^{1}H/^{13}C nuclei); Assignments were made based on COSY, HSQC and HMBC experiments.

Figure S1. ^{1}H NMR spectrum (600 MHz, $CDCl_3$) of marilzafurollene A (**1**).

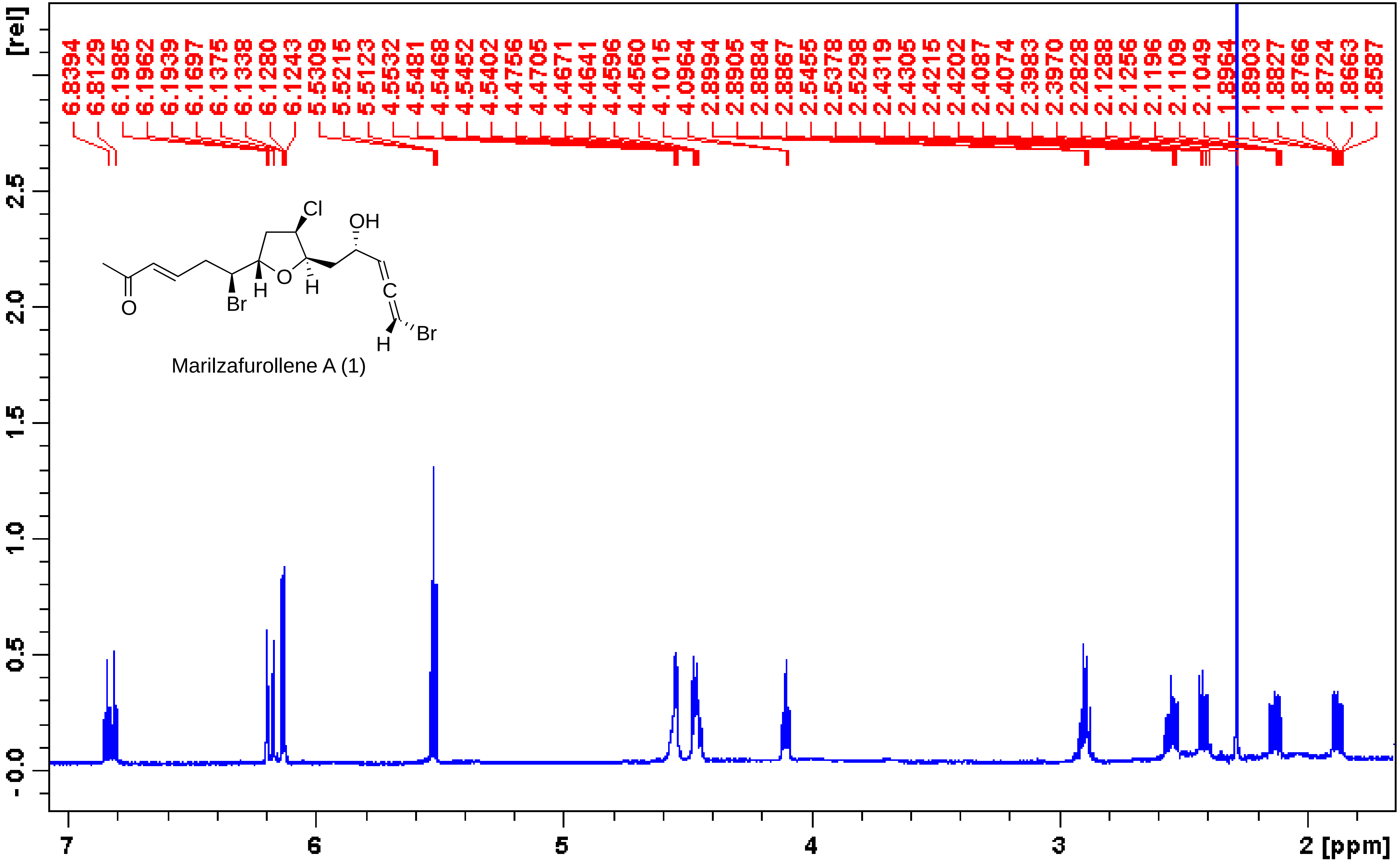

Figure S2. ^{13}C NMR spectrum (150 MHz, $CDCl_3$) of compound **1**.

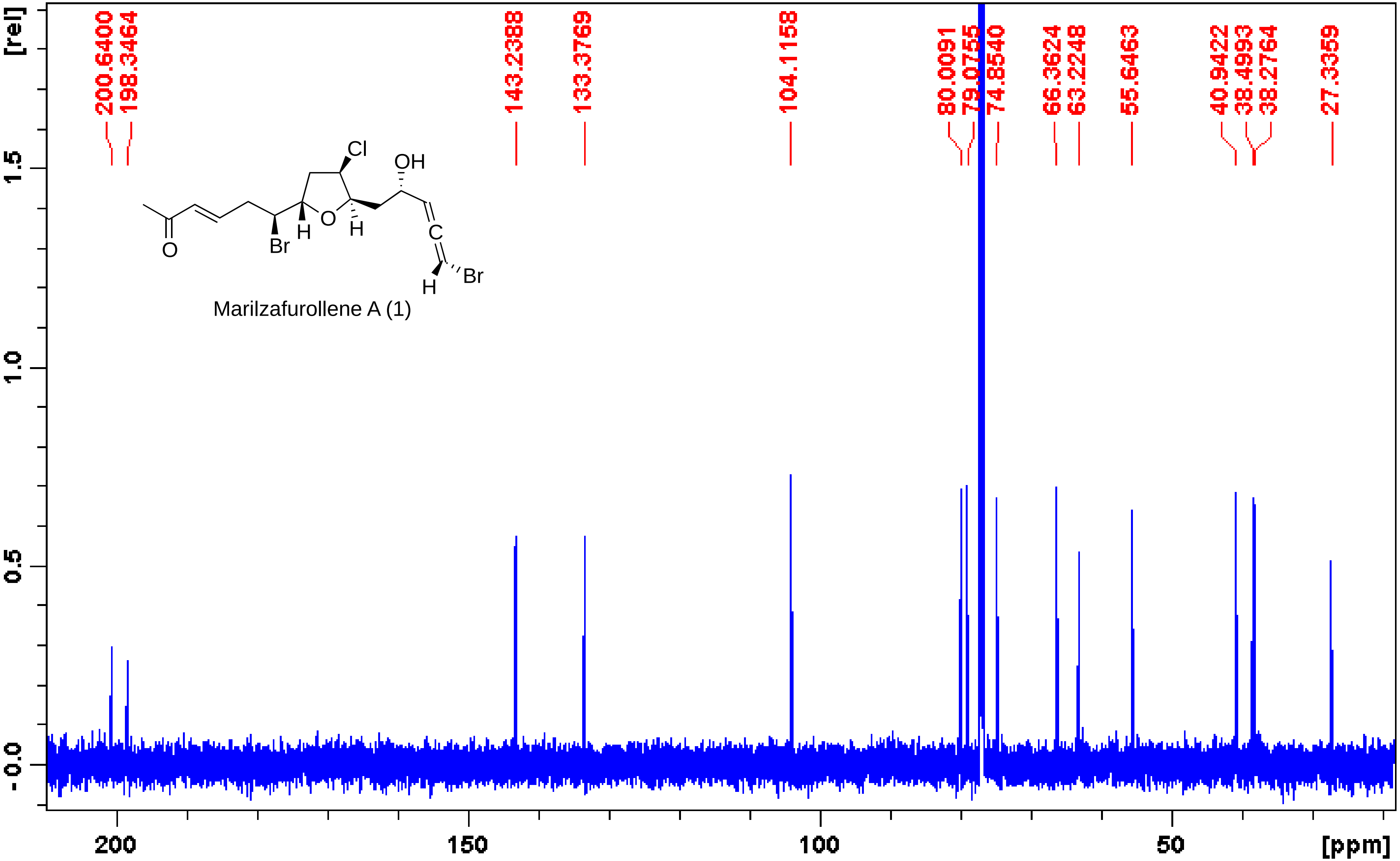

Figure S3. ^{1}H-^{1}H COSY NMR spectrum (600 MHz, $CDCl_3$) of compound **1**.

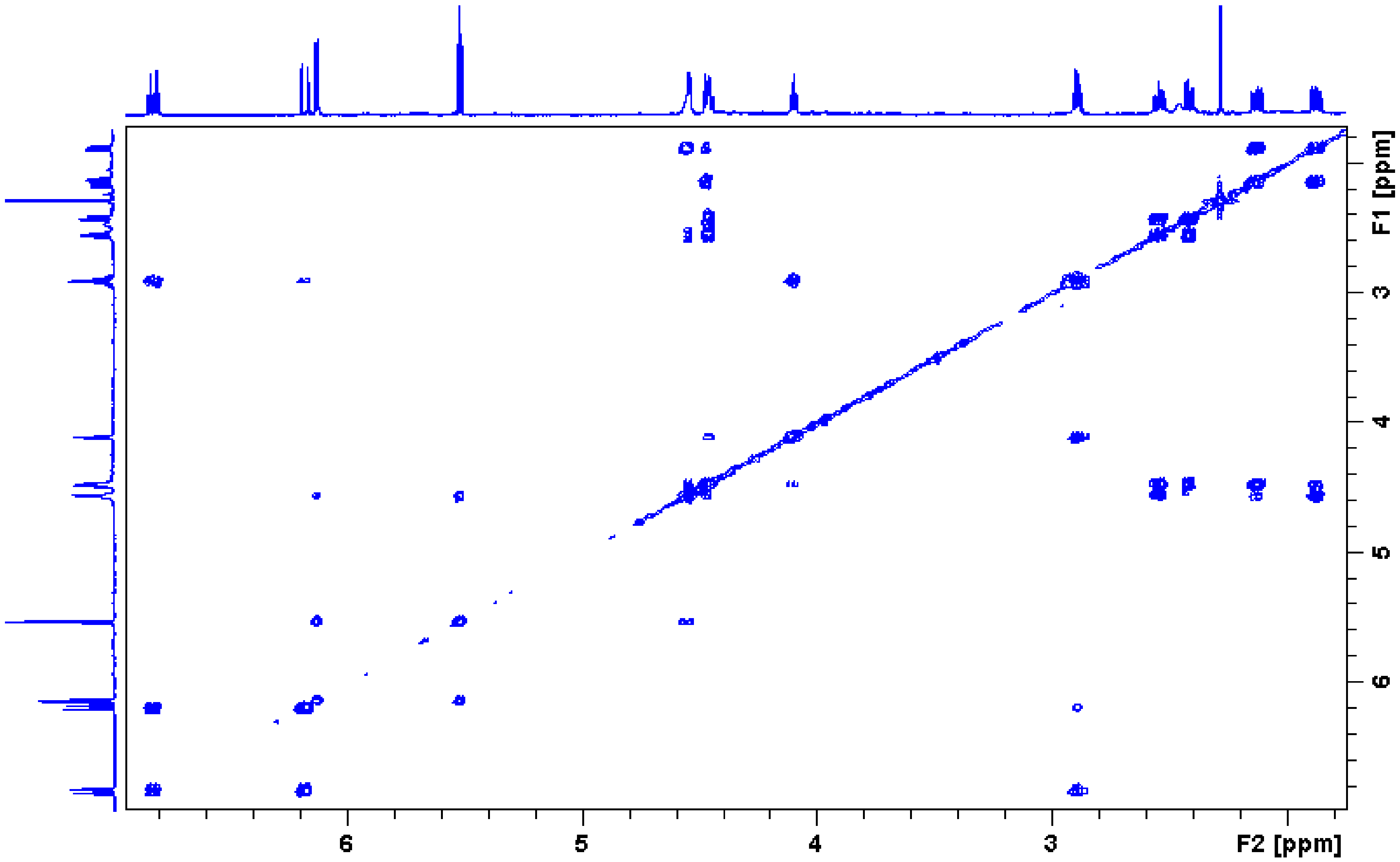

Figure S4. HSQC NMR spectrum (600 MHz, $CDCl_3$) of compound **1**.

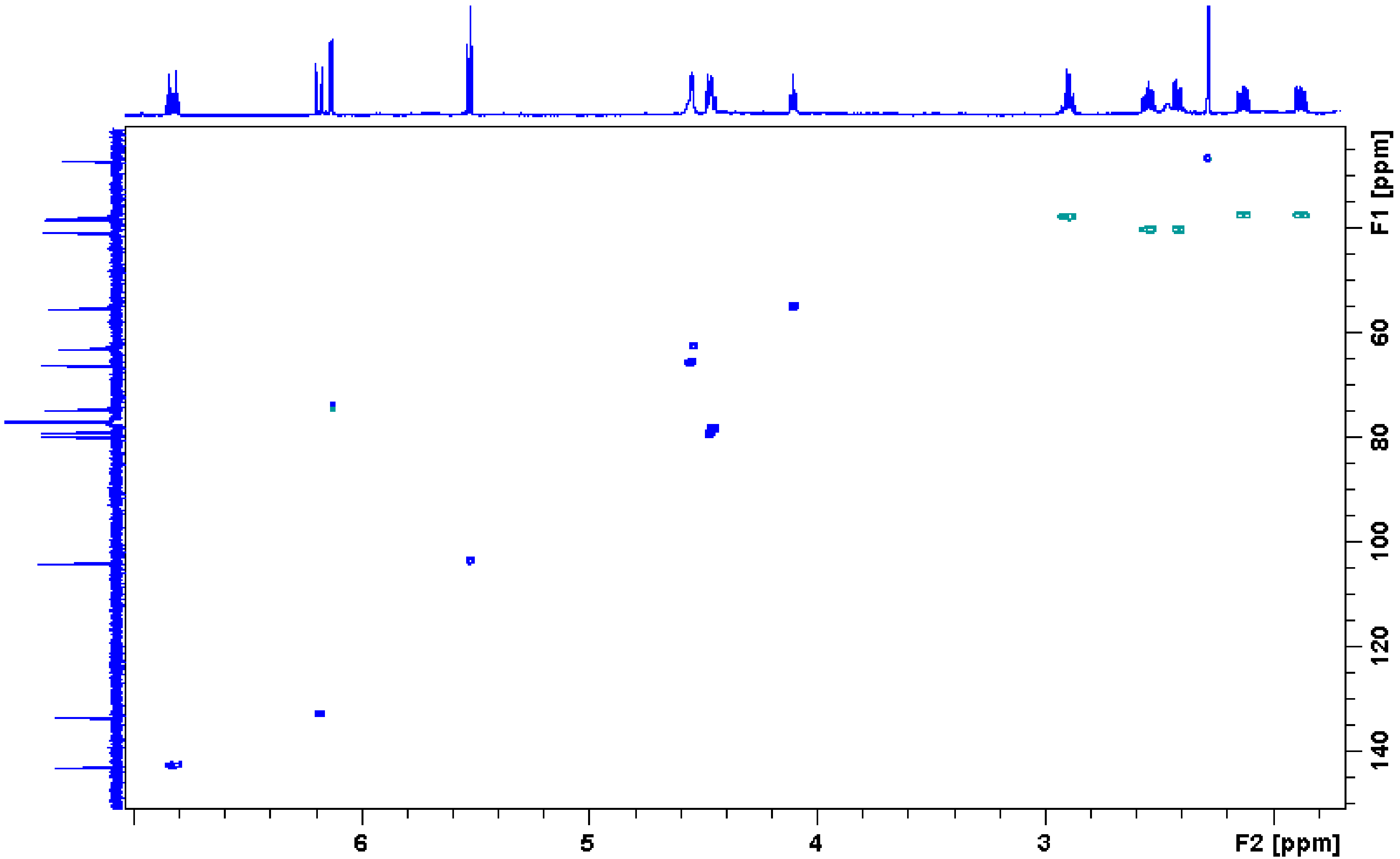

Figure S5. HMBC NMR spectrum (600 MHz, $CDCl_3$) of compound **1**.

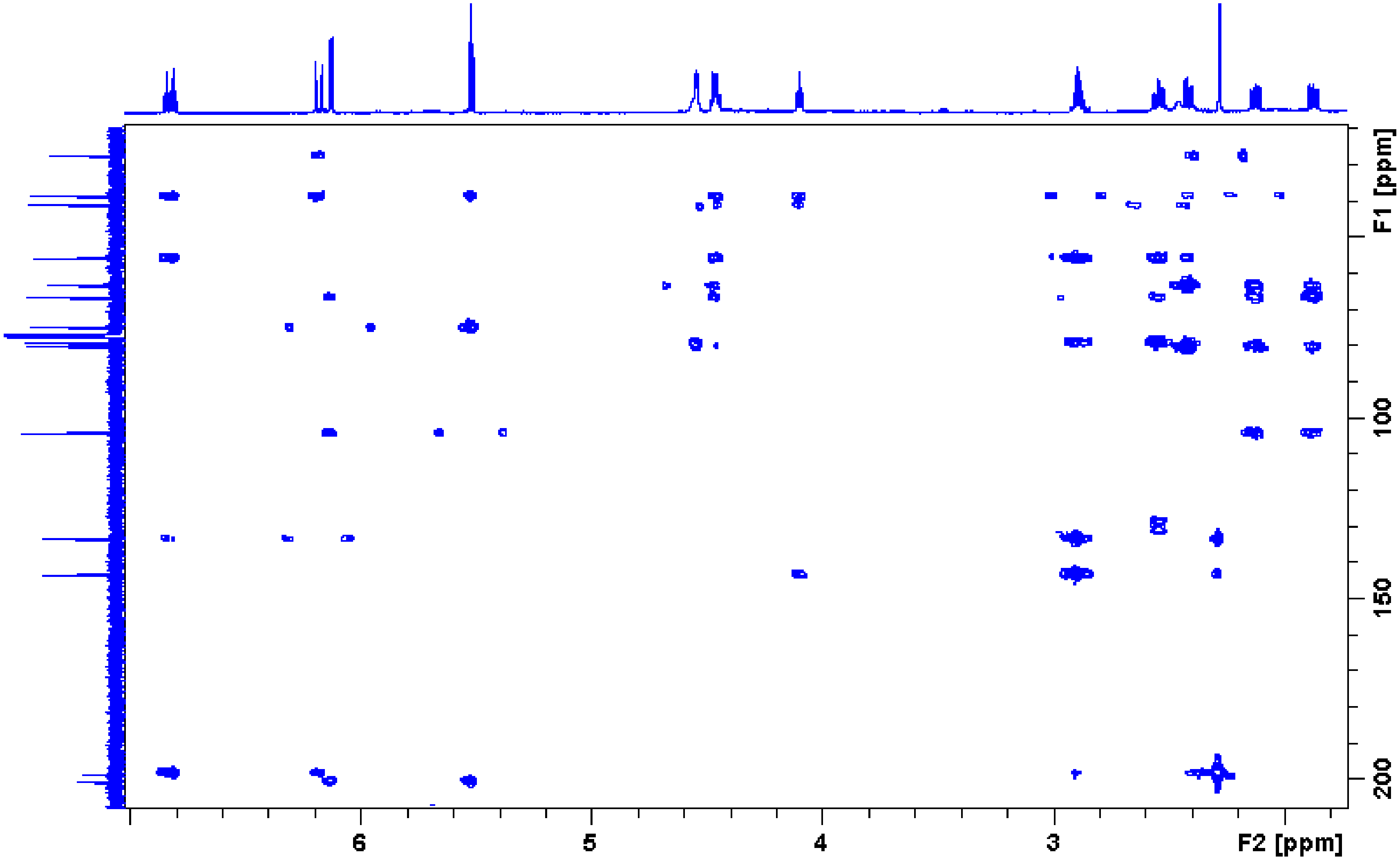

Figure S6. ROESY NMR spectrum (600 MHz, $CDCl_3$) of compound **1**.

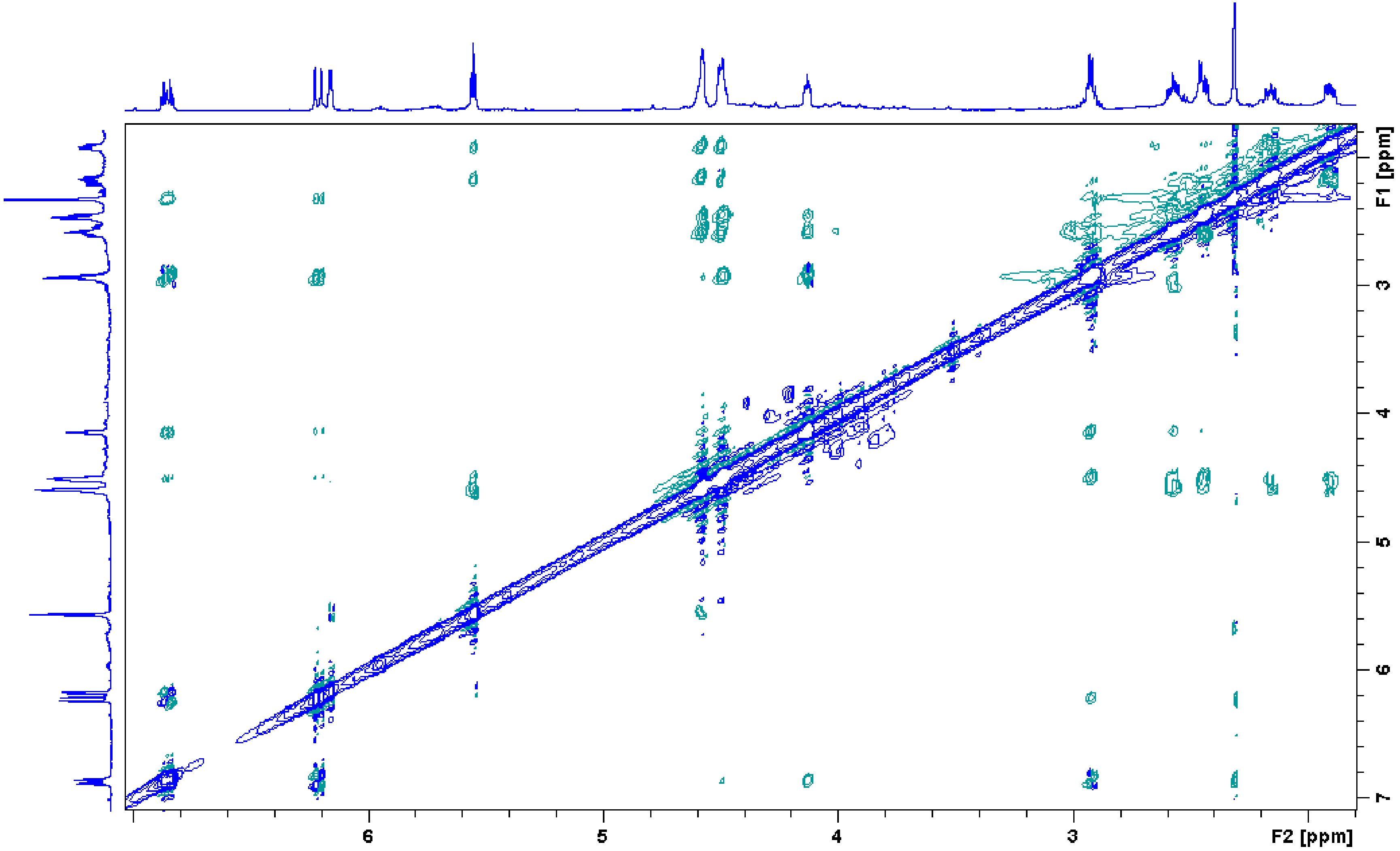

Figure S7. Selected sections of the HSQC-HECADE spectrum (600 MHz, $CDCl_3$) of compound **1**.

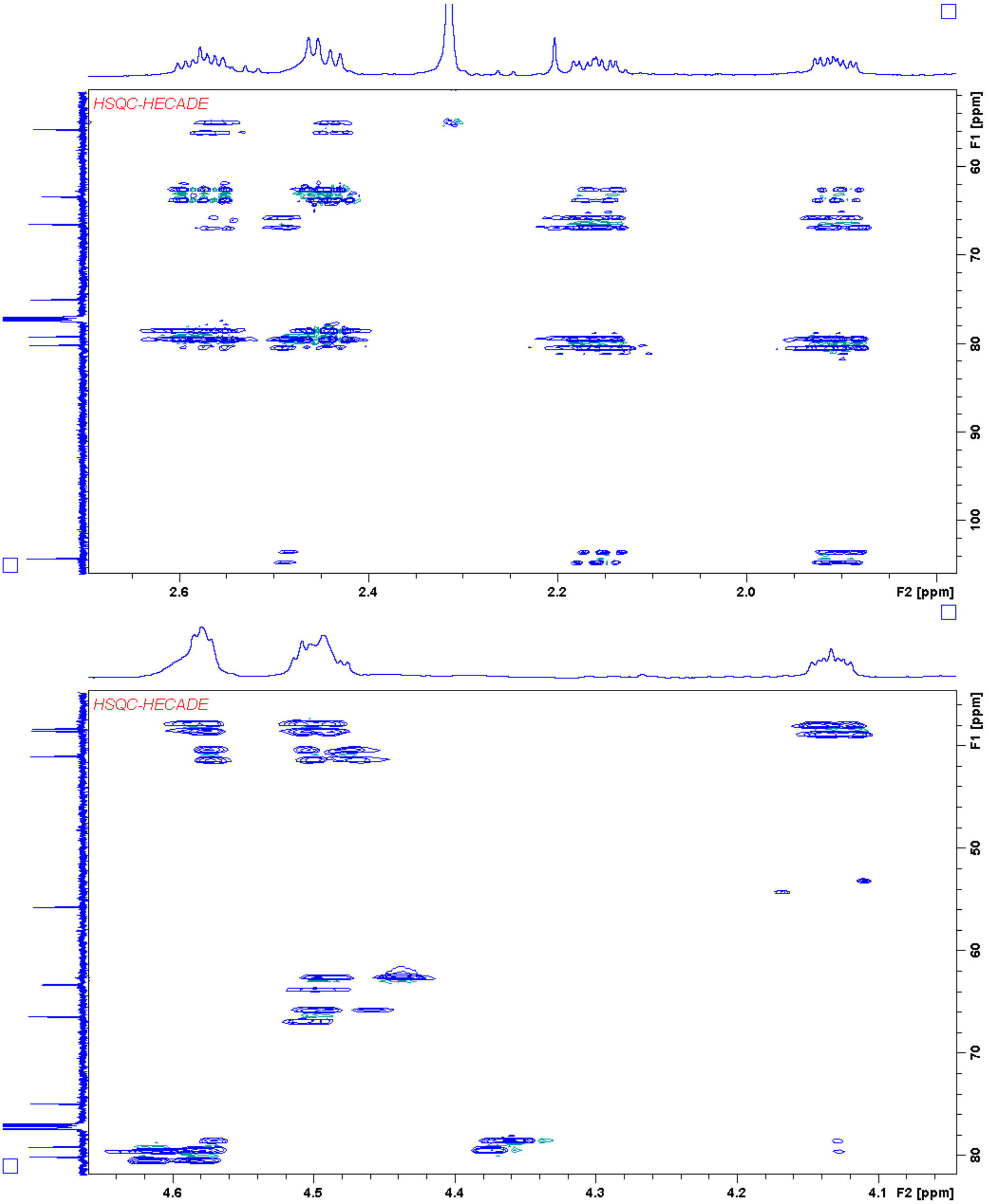

Figure S8. ^{1}H NMR spectrum (600 MHz, $CDCl_3$) of marilzafurollene B (**2**).

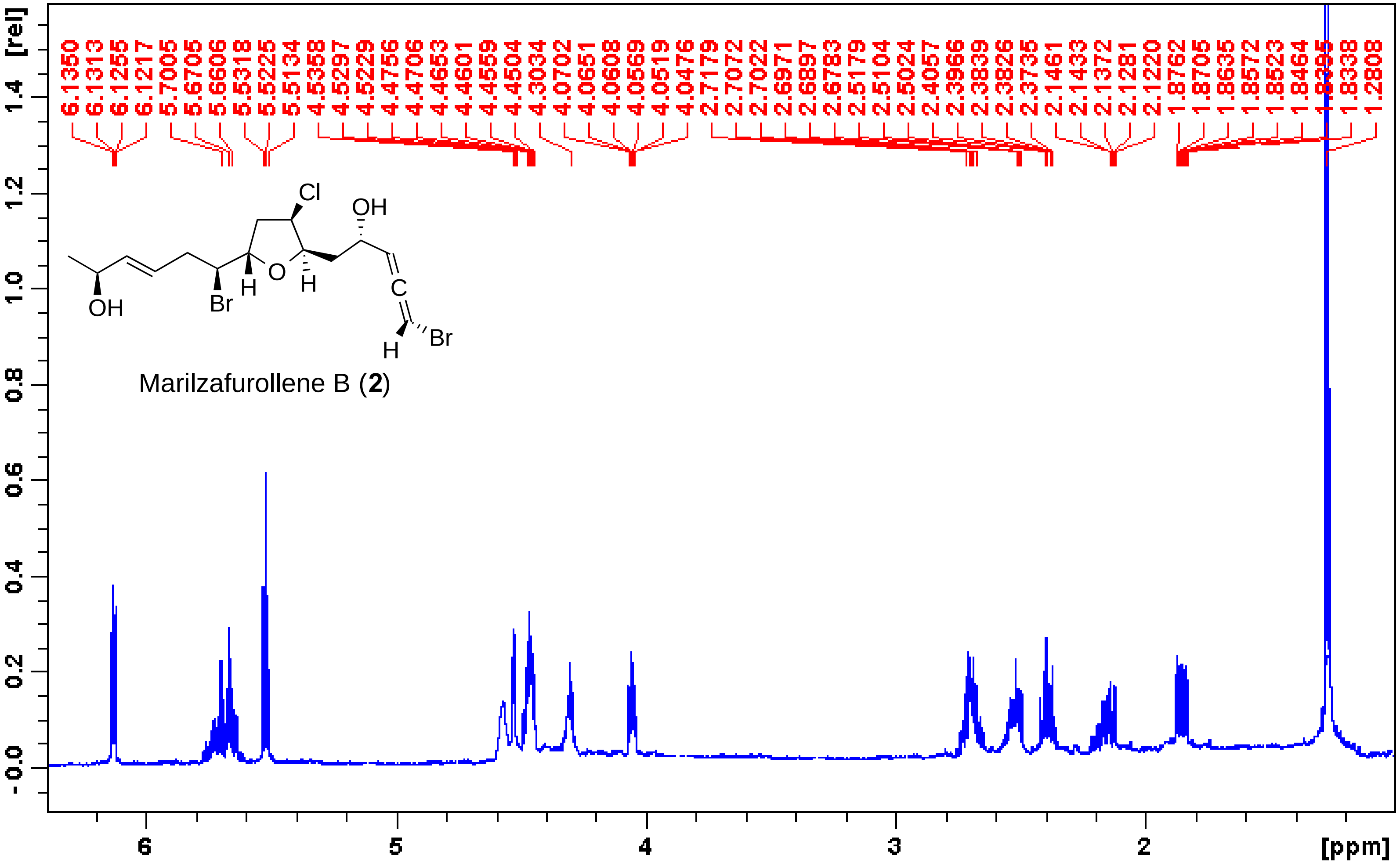

Figure S9. ^{13}C NMR spectrum (150 MHz, $CDCl_3$) of compound **2**.

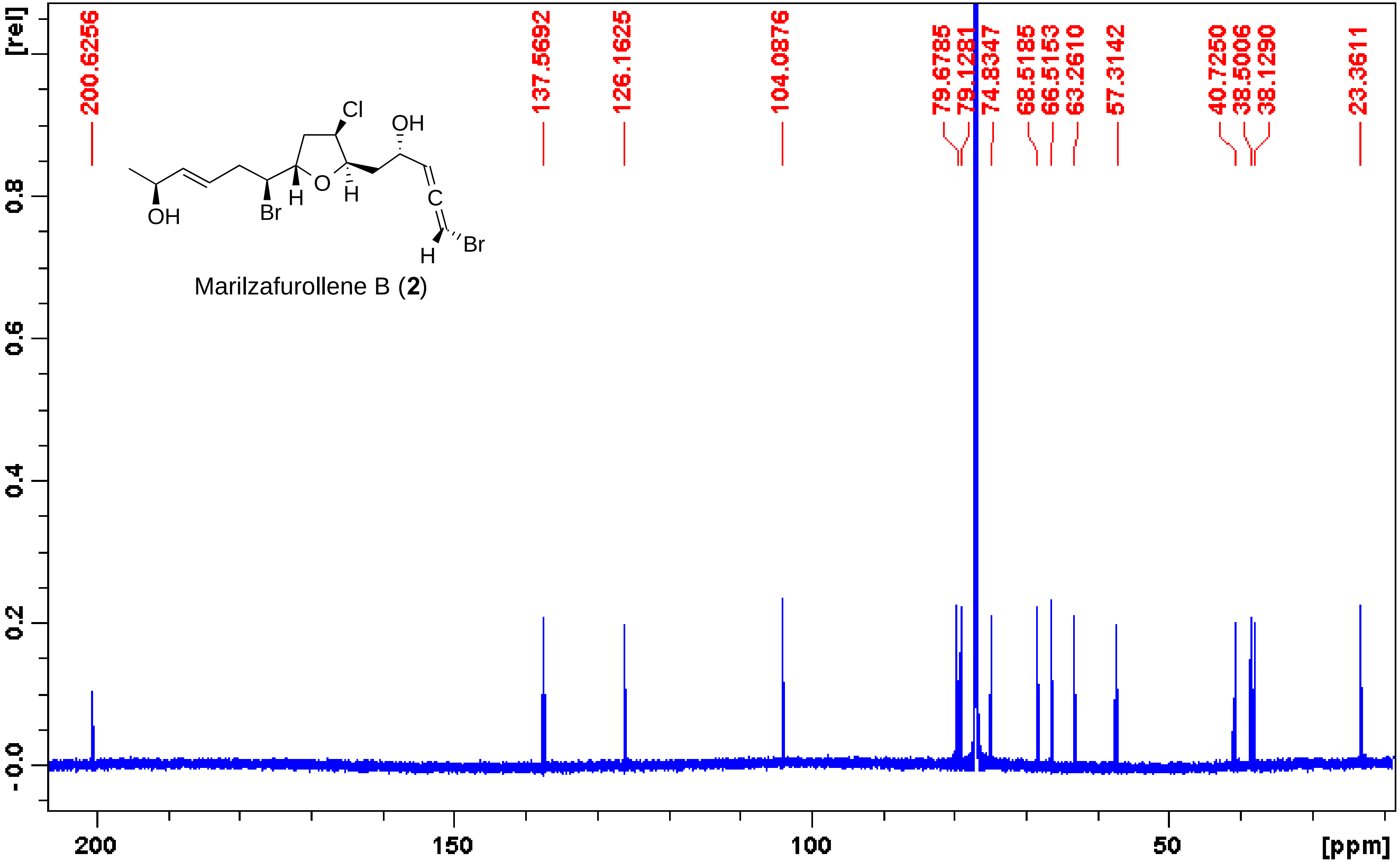

Figure S10. ^{1}H NMR spectrum (600 MHz, $CDCl_3$) of marilzafurollene C (**3**).

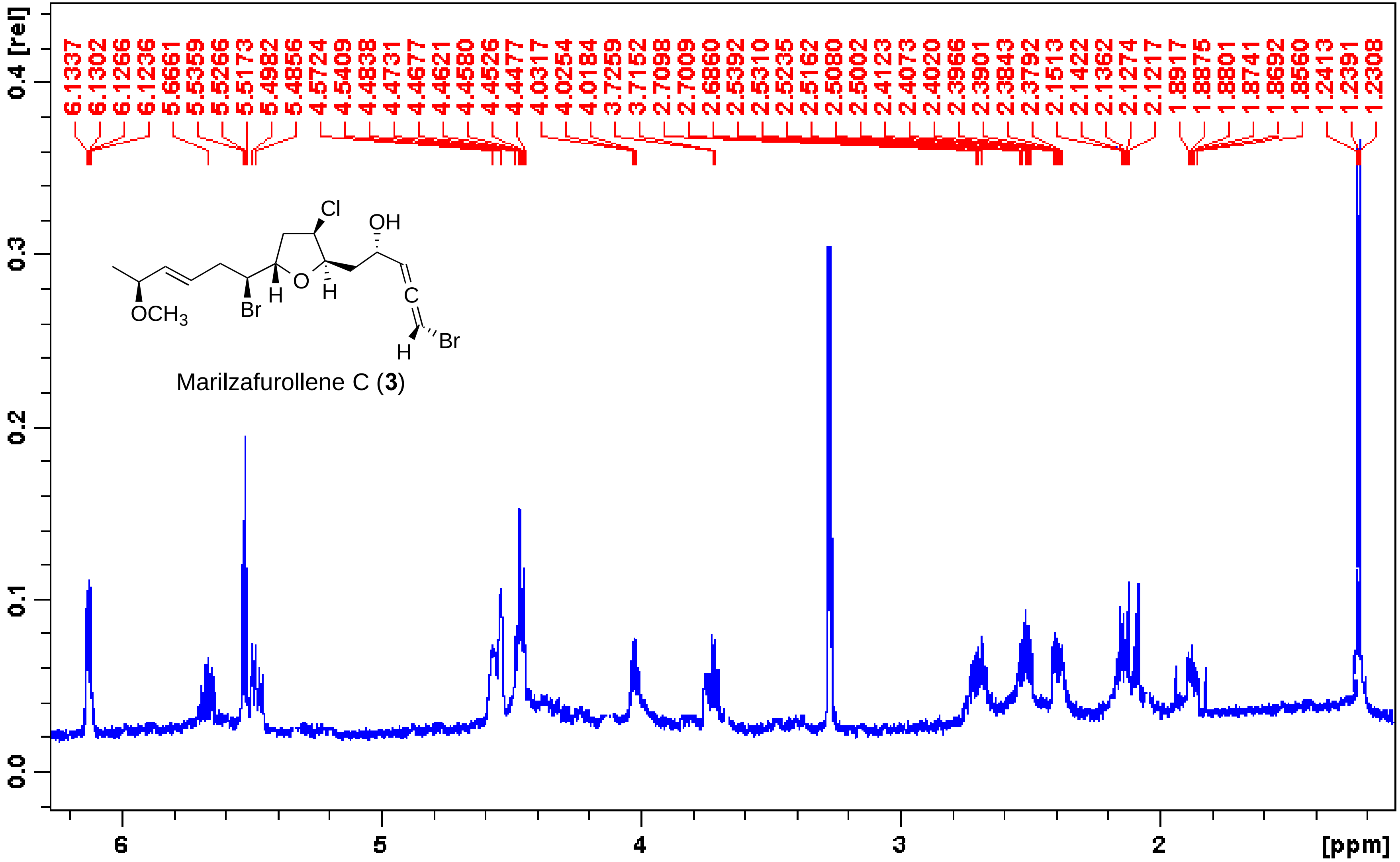

Figure S11. ^{13}C NMR spectrum (150 MHz, $CDCl_3$) of compound **3**.

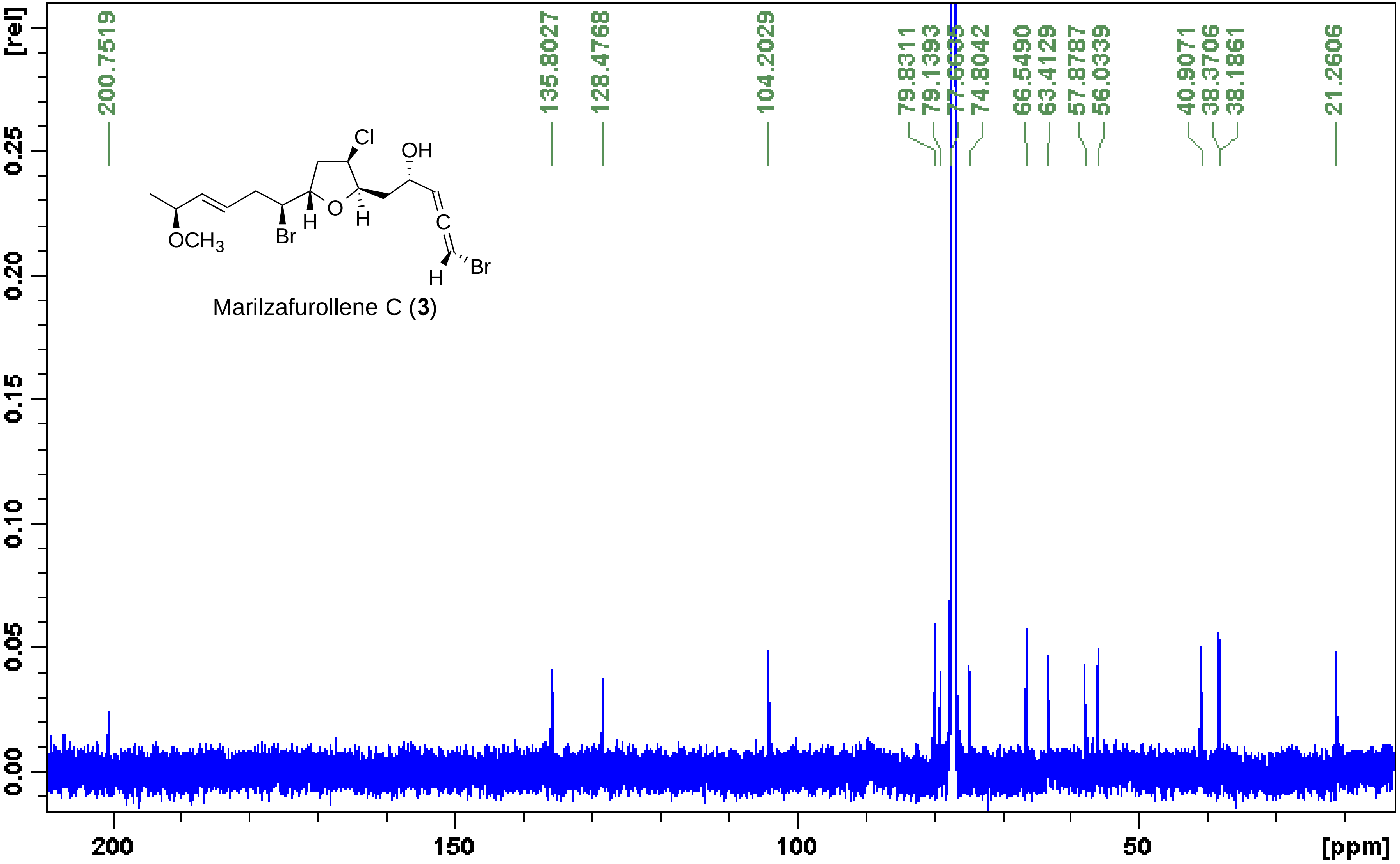

Figure S12. ^{1}H NMR spectrum (600 MHz, $CDCl_3$) of marilzafurollene D (**4**).

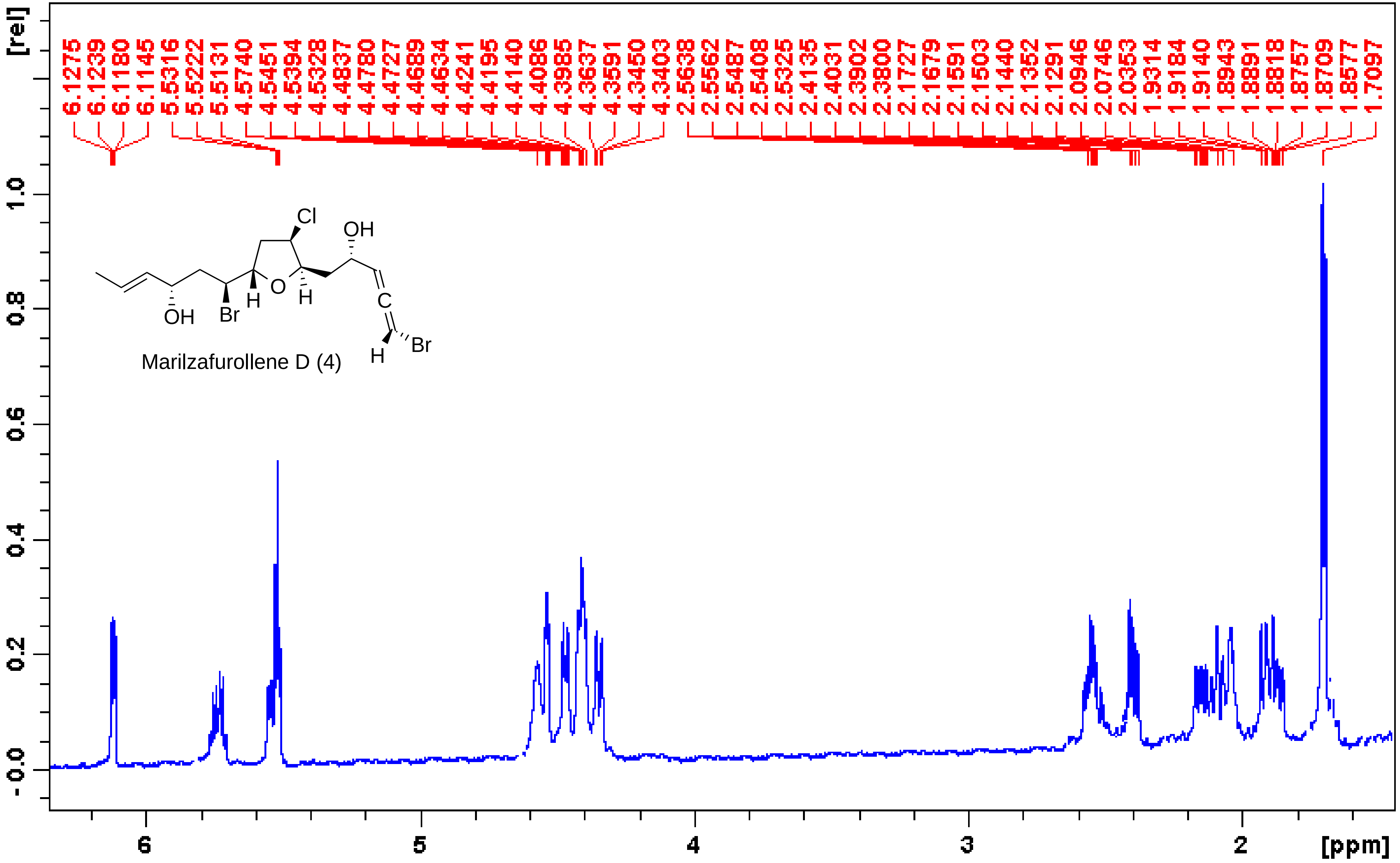

Figure S13. ^{13}C NMR spectrum (150 MHz, $CDCl_3$) of compound **4**.

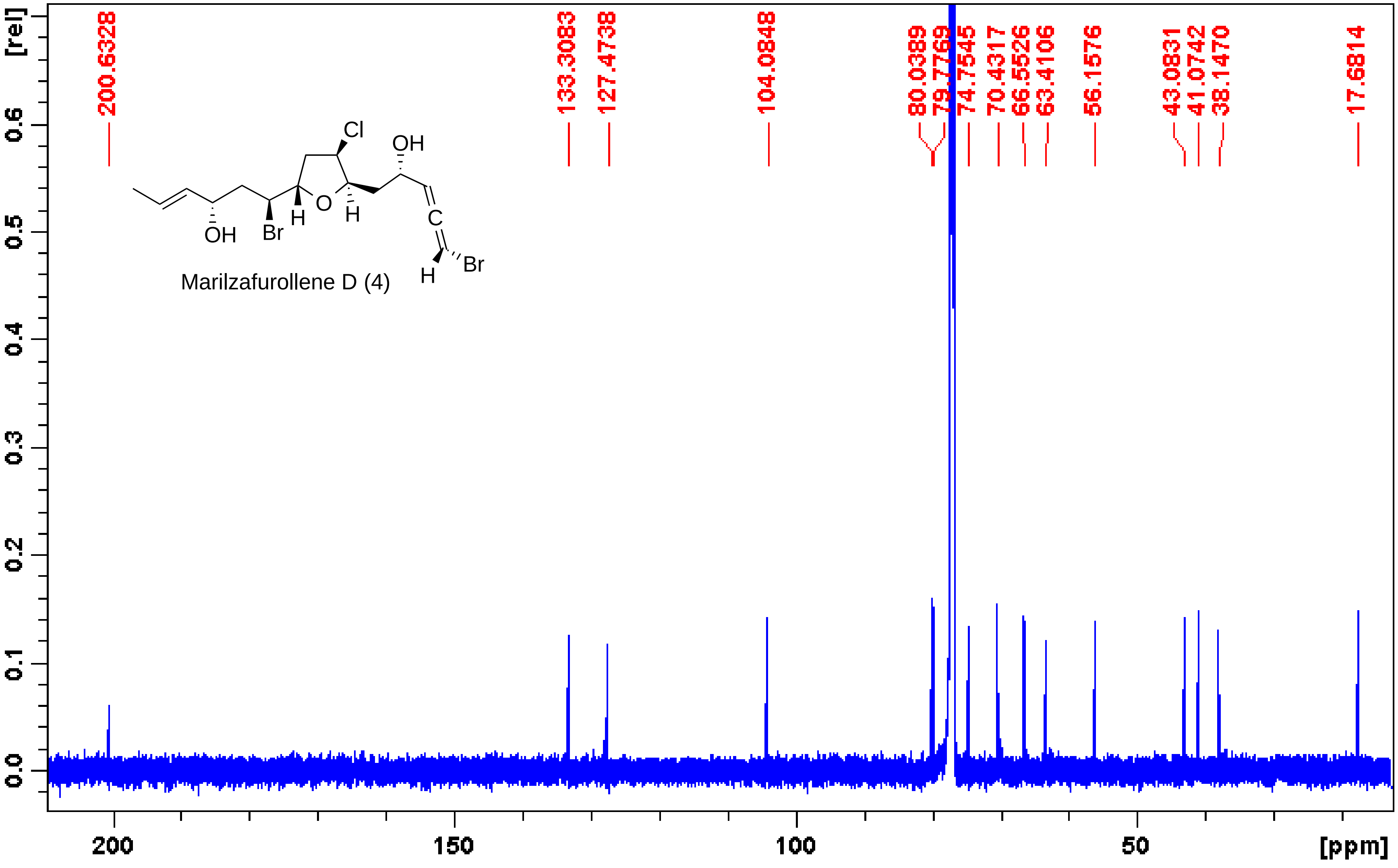

Figure S14. ^{1}H NMR spectrum (600 MHz, C_6D_6) of compound **4**.

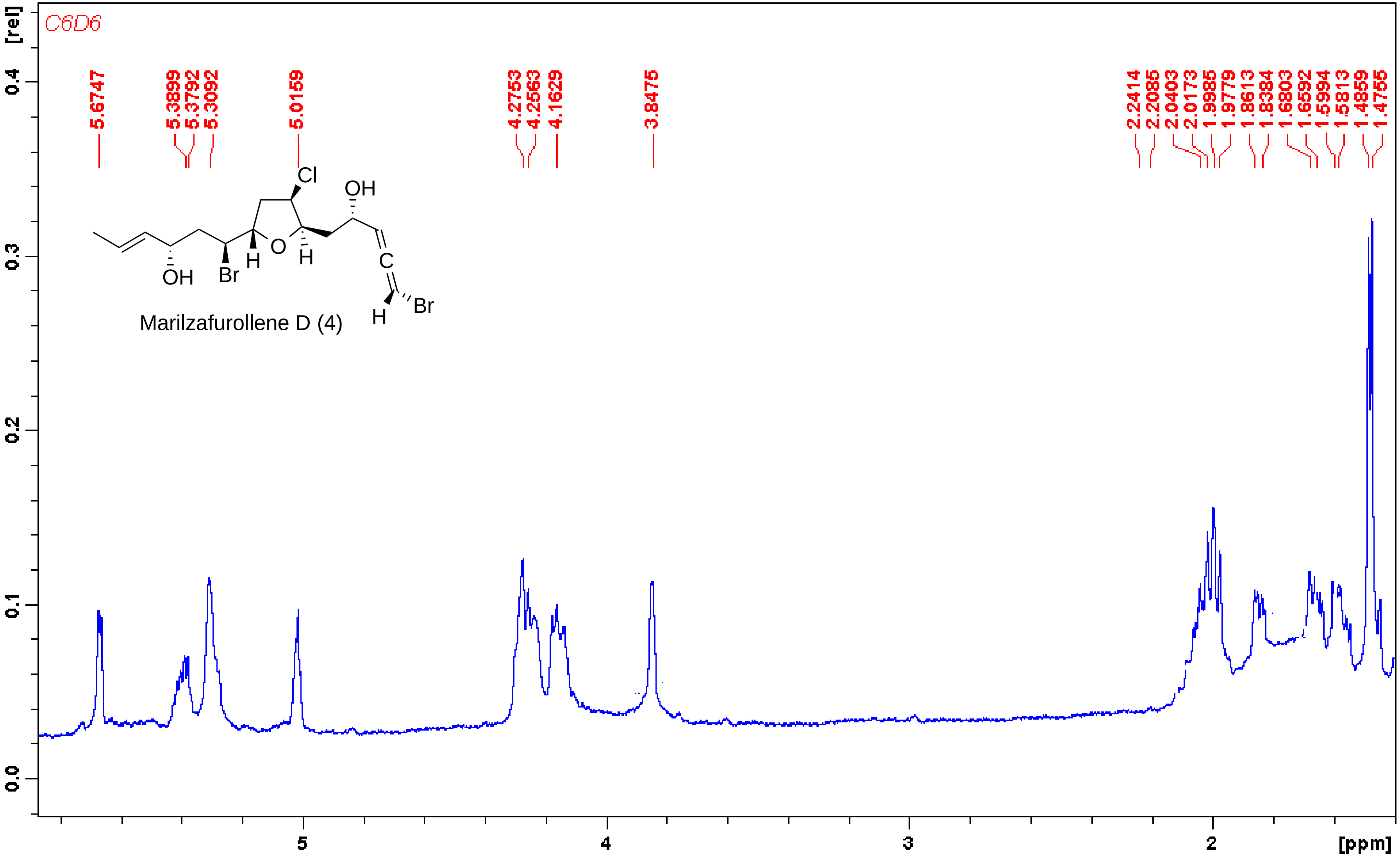

Figure S15. HSQC NMR spectrum (600 MHz, C_6D_6) of compound **4**.

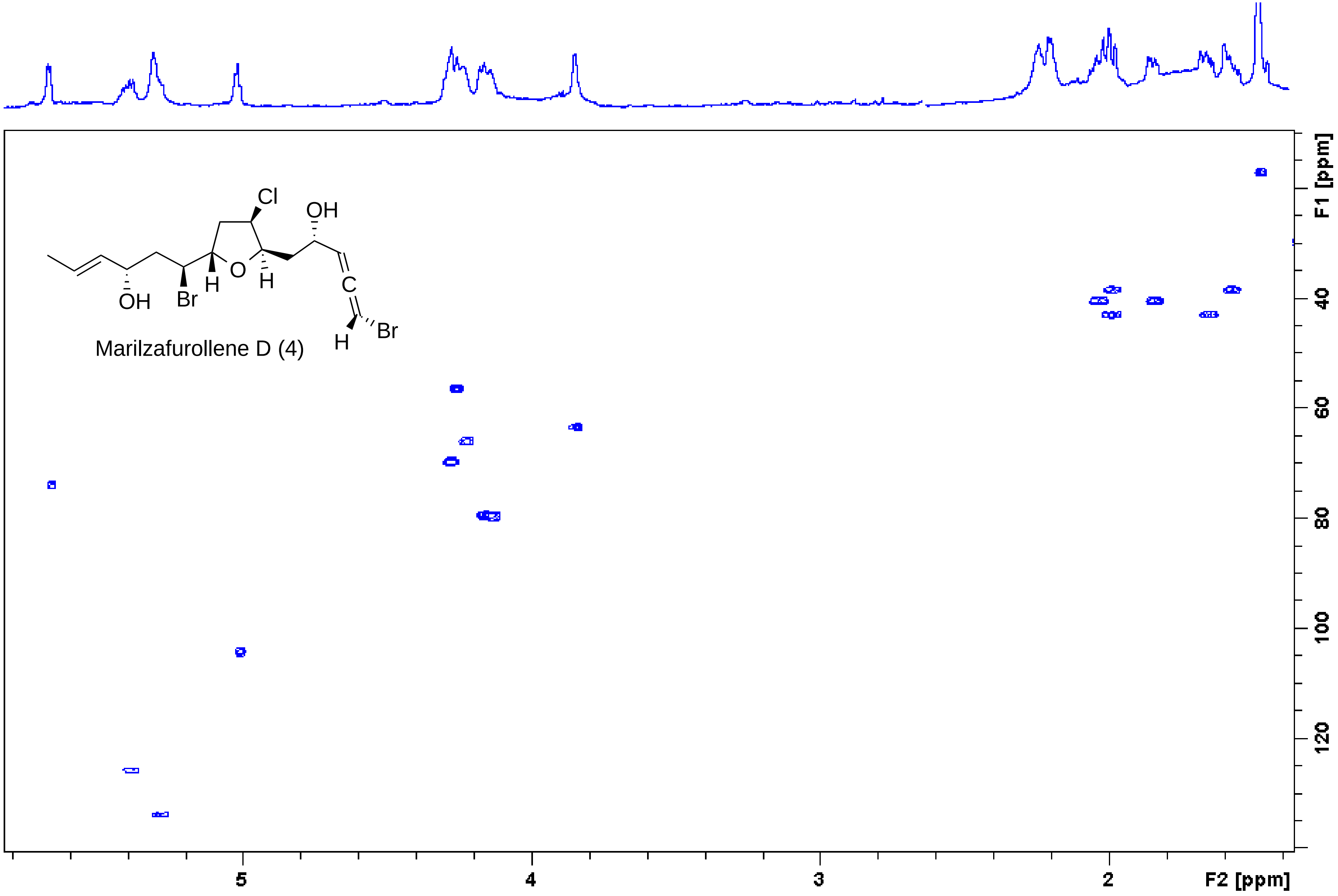

Figure S16. Selected sections of the HSQC-HECADE spectrum (600 MHz, C_6D_6) of compound **4**.

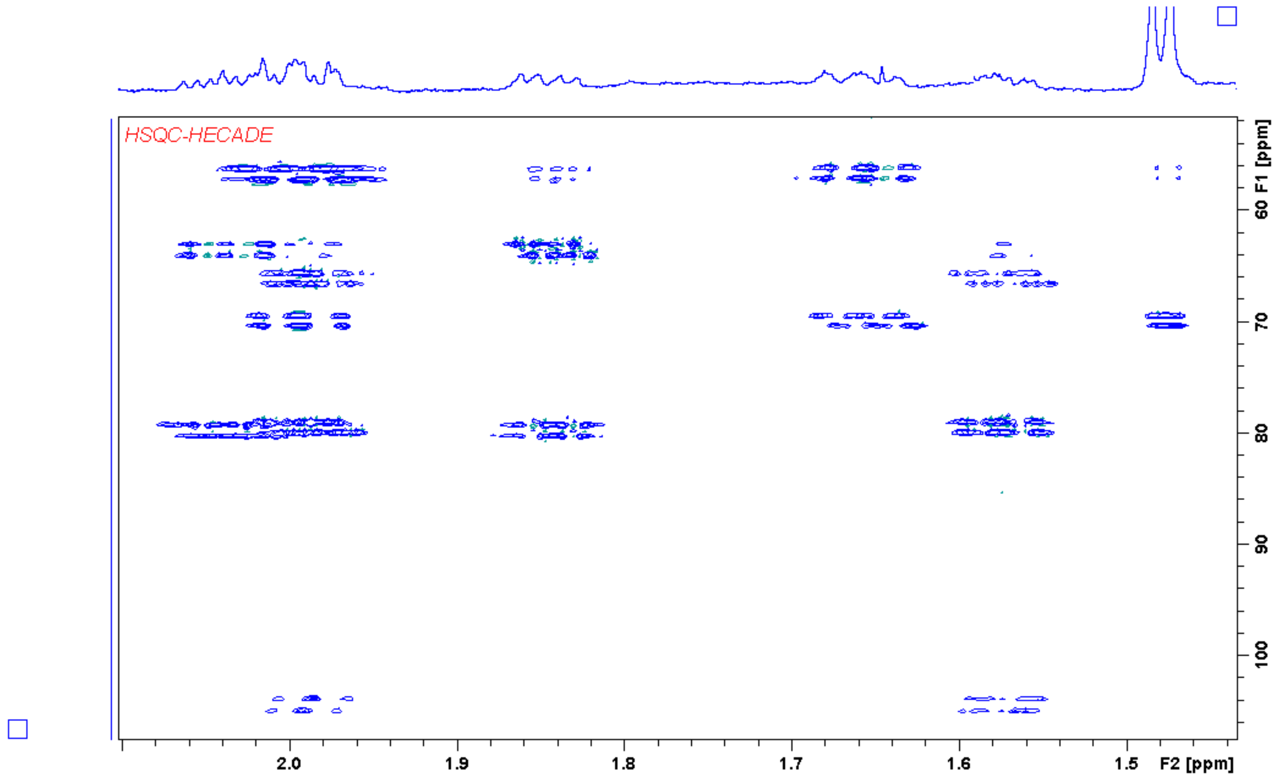

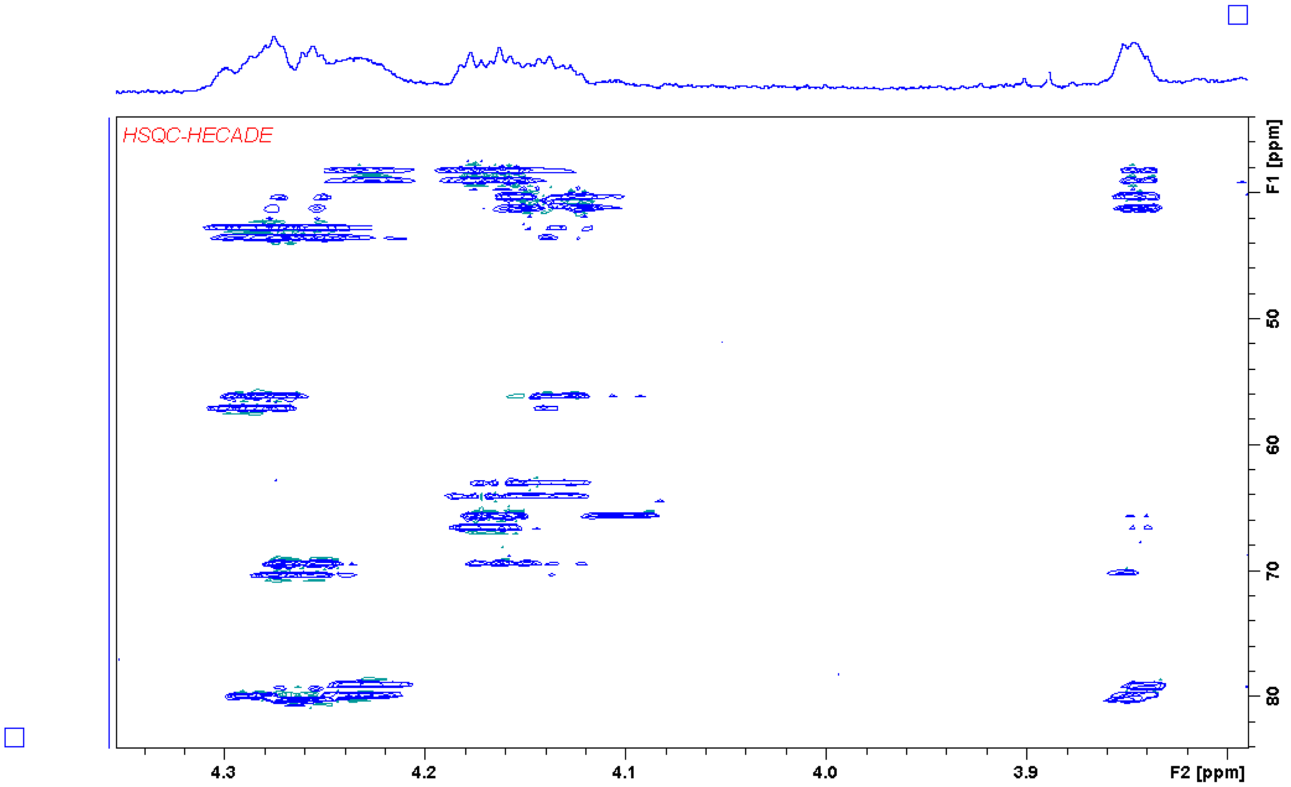

Figure S17. ^{1}H NMR spectrum (600 MHz, $CDCl_3$) of 12-acetoxy-marilzafurenyne (**5**).

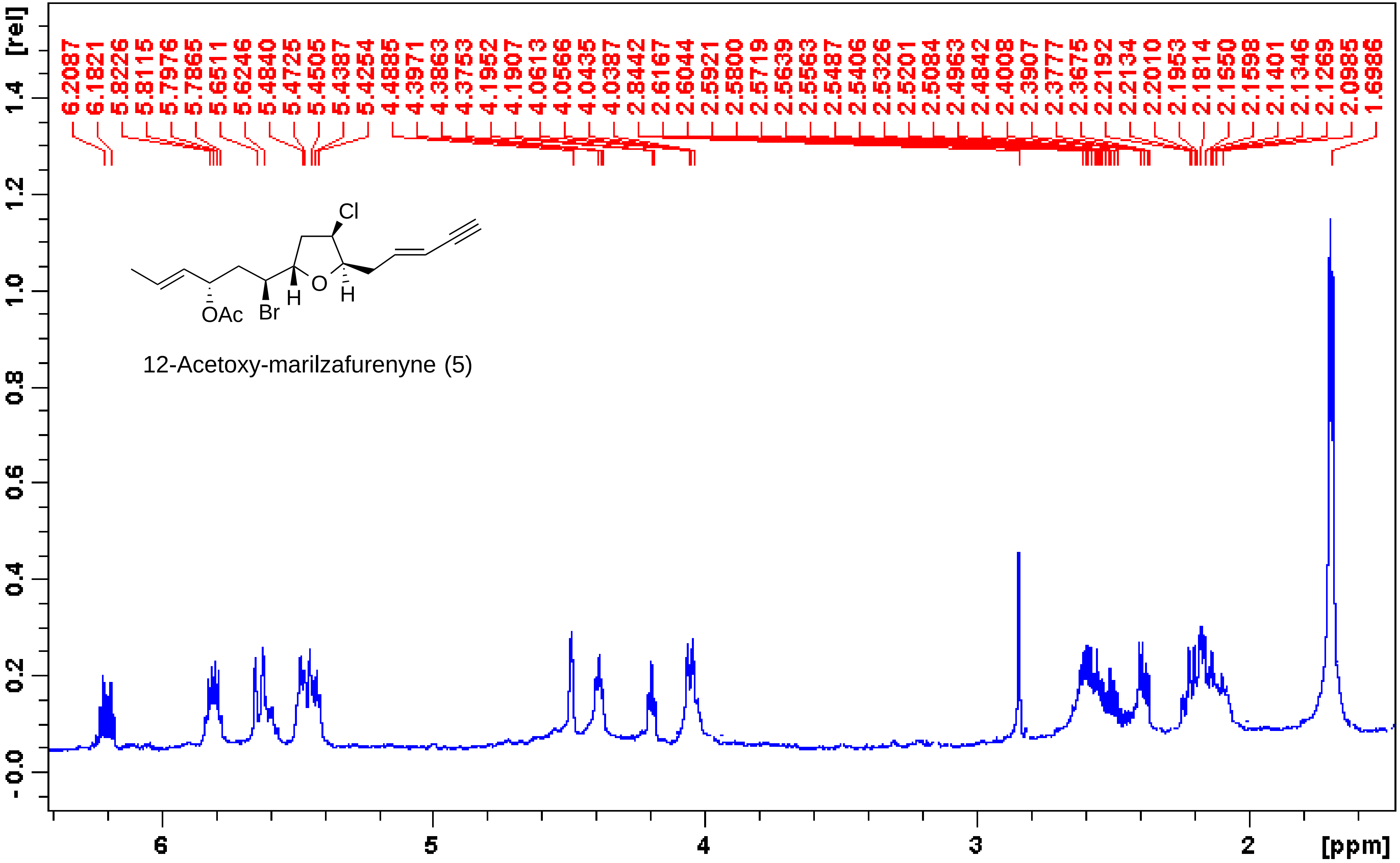

Figure S18. ^{13}C NMR spectrum (150 MHz, $CDCl_3$) of compound **5**.

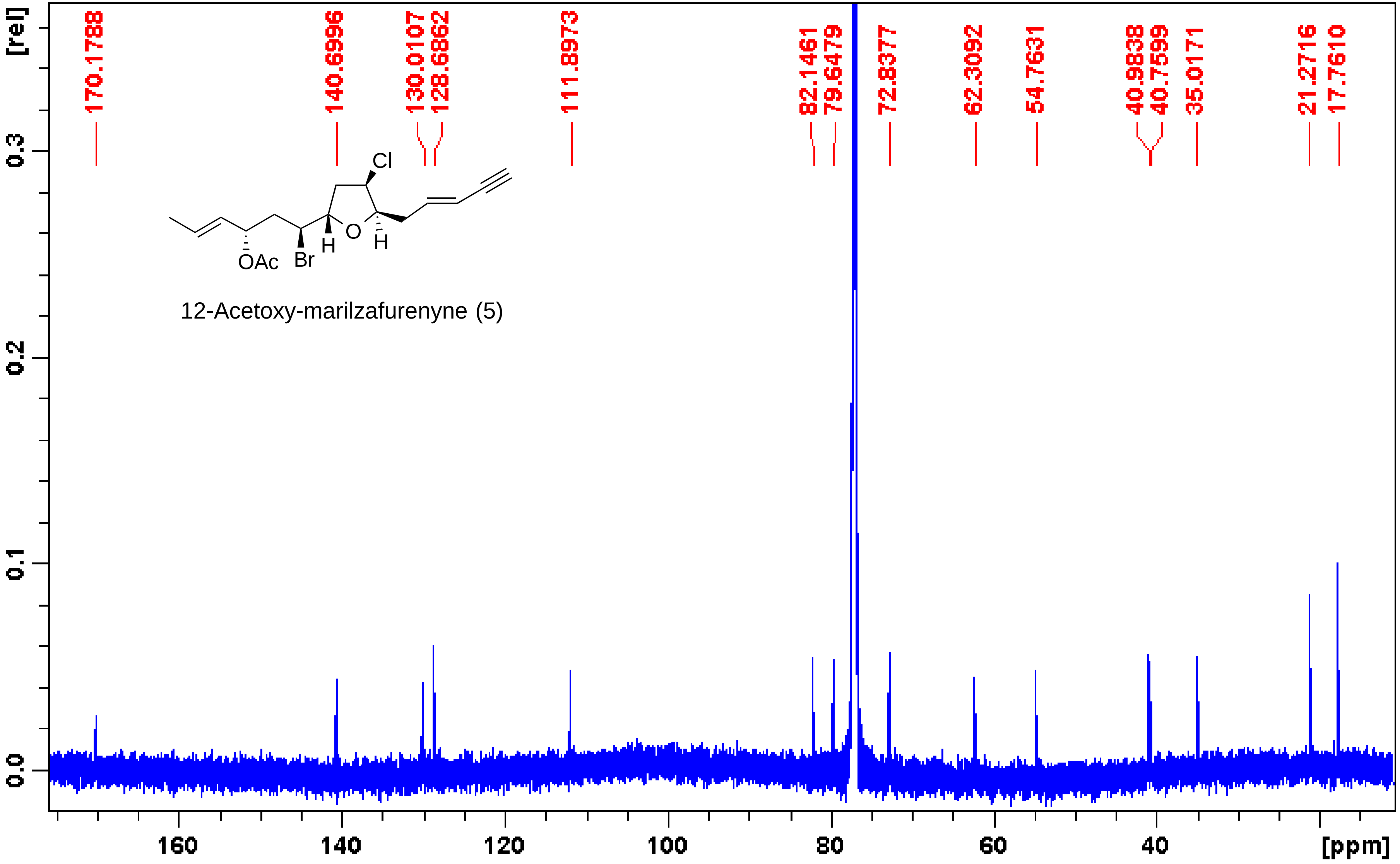

Table S2. Calculated ^{13}C and ^{1}H chemical shifts for marilzafurollene A (**1**).

C	***S$_a$*,4*S*,6*R*,7*R*,9*S*,10*S***	***S$_a$*,4*R*,6*S*,7*S*,9*R*,10*R***	H	***S$_a$*,4*S*,6*R*,7*R*,9*S*,10*S***	***S$_a$*,4*R*,6*S*,7*S*,9*R*,10*R***
**1**	81.4	82.6	**1**	5.28	5.46
**2**	204.9	204.9	**2**	-	-
**3**	94.0	93.8	**3**	5.49	5.67
**4**	68.6	68.1	**4**	4.66	4.60
**5**	39.9	40.0	**5b**	2.44	1.69
			**5a**	1.84	2.26
**6**	81.4	81.9	**6**	4.42	4.38
**7**	66.3	66.4	**7**	4.20	4.21
**8**	39.5	39.6	**8b**	2.68	1.82
			**8a**	2.04	2.45
**9**	80.9	80.4	**9**	4.86	4.90
**10**	64.9	66.2	**10**	3.88	3.81
**11**	42.4	42.7	**11b**	2.96	2.84
			**11a**	2.93	2.81
**12**	142.9	142.3	**12**	6.91	7.08
**13**	132.4	132.9	**13**	6.17	6.29
**14**	197.1	197.4	**14**	-	-
**15**	23.2	23.1	**15**	2.28	2.10

Values were obtained after statistical analysis by linear regression of computed isotropic shieldings against experimental chemical shifts.

Figure S19. Correlation plots obtained for marilzafurollene A (**1**).

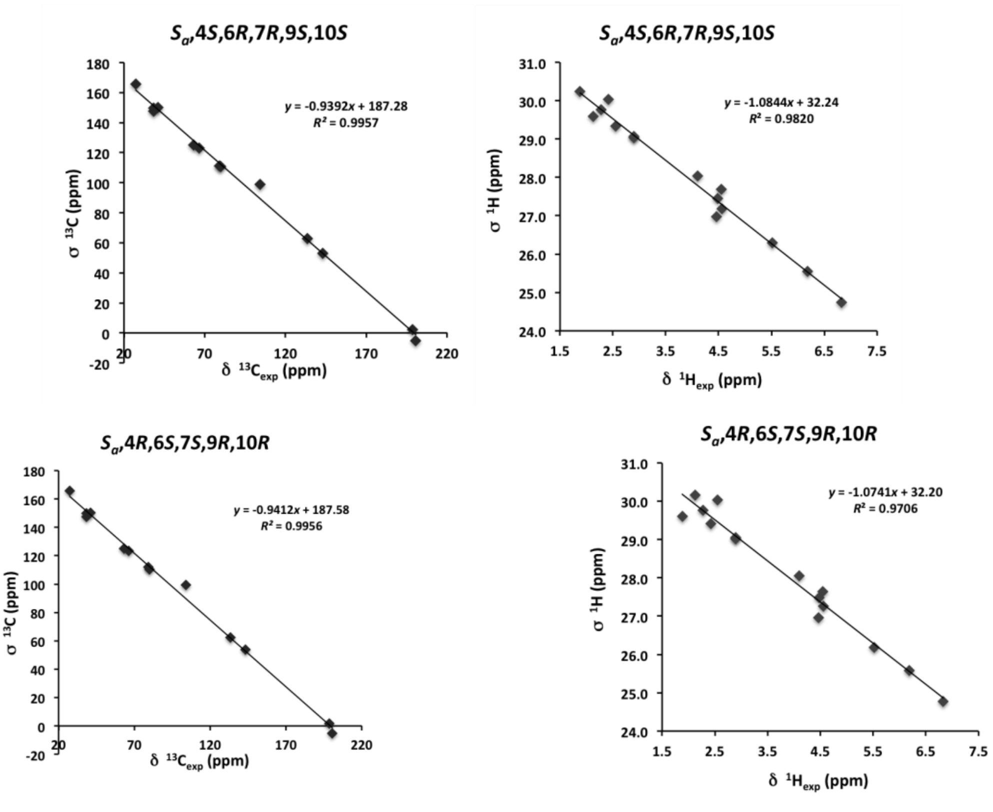

Table S3. Calculated ^{13}C and ^{1}H chemical shifts for marilzafurollene C (**3**).

C	***S**_a_,4S,6R,7R,9S,10S,14S*	***S**_a_,4R,6S,7S,9R,10R,14R*	H	***S**_a_,4S,6R,7R,9S,10S,14S*	***S**_a_,4R,6S,7S,9R,10R,14R*
**1**	96.9	95.5	**1**	6.25	6.28
**2**	201.4	199.4	**2**	-	-
**3**	103.3	100.6	**3**	5.70	5.67
**4**	69.3	71.0	**4**	4.49	4.46
**5**	38.8	37.5	**5b**	1.78	1.77
			**5a**	2.24	2.26
**6**	80.6	82.5	**6**	4.35	4.41
**7**	64.1	62.9	**7**	4.21	4.18
**8**	40.2	37.2	**8b**	2.53	2.54
			**8a**	2.11	2.15
**9**	76.5	77.3	**9**	4.76	4.89
**10**	71.2	71.7	**10**	3.85	3.86
**11**	42.4	39.1	**11b**	2.78	2.75
			**11a**	2.87	2.82
**12**	129.2	127.2	**12**	5.85	5.88
**13**	137.0	136.1	**13**	5.72	5.62
**14**	76.1	76.1	**14**	3.56	3.52
**15**	20.8	17.7	**15**	1.45	1.43
**16**	52.4	51.0	**16**	3.27	3.28

Values were obtained after statistical analysis by linear regression of computed isotropic shieldings against experimental chemical shifts.

Figure S20. Correlation plots obtained for marilzafurollene C (**3**).

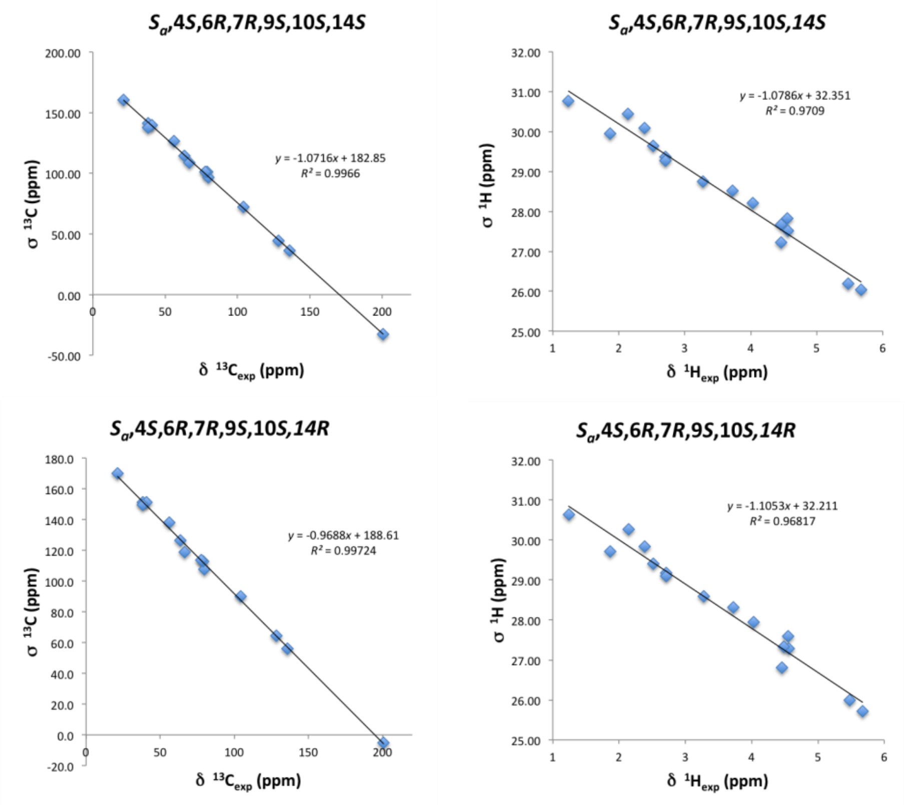

Table S4. Calculated ^{13}C and ^{1}H chemical shifts for 12-acetoxy-marilzafurenyne (**5**).

C	4*S*,6*R*,7*R*,9*S*,10*S*,12*S*	4*R*,6*S*,7*S*,9*R*,10*R*,12*R*	H	4*S*,6*R*,7*R*,9*S*,10*S*,12*S*	4*R*,6*S*,7*S*,9*R*,10*R*,12*R*
**1**	73.9	73.4	**1**	3.08	3.03
**2**	74.9	75.3	**2**	-	-
**3**	107.5	107.6	**3**	5.46	5.45
**4**	144.5	145.5	**4**	6.39	6.42
**5**	38.0	38.2	**5b**	2.67	2.62
			**5a**	2.31	2.28
**6**	83.5	83.8	**6**	4.22	4.18
**7**	64.7	65.3	**7**	4.08	4.01
**8**	40.1	40.5	**8b**	2.56	2.42
			**8a**	2.16	2.21
**9**	80.9	77.1	**9**	4.58	5.25
**10**	70.0	71.7	**10**	3.93	3.83
**11**	44.1	45.1	**11b**	2.24	2.14
			**11a**	2.12	2.46
**12**	70.6	71.4	**12**	5.43	5.18
**13**	128.7	129.9	**13**	5.40	5.61
**14**	132.8	129.8	**14**	6.04	5.92
**15**	17.8	17.9	**15**	1.90	1.86
**16**	170.2	171.5	**16**	-	-
**17**	20.8	20.9	**17**	2.12	2.11

Values were obtained after statistical analysis by linear regression of computed isotropic shieldings against experimental chemical shifts.

Figure S21. Correlation plots obtained for 12-acetoxy-marilzafurenyne (**5**).

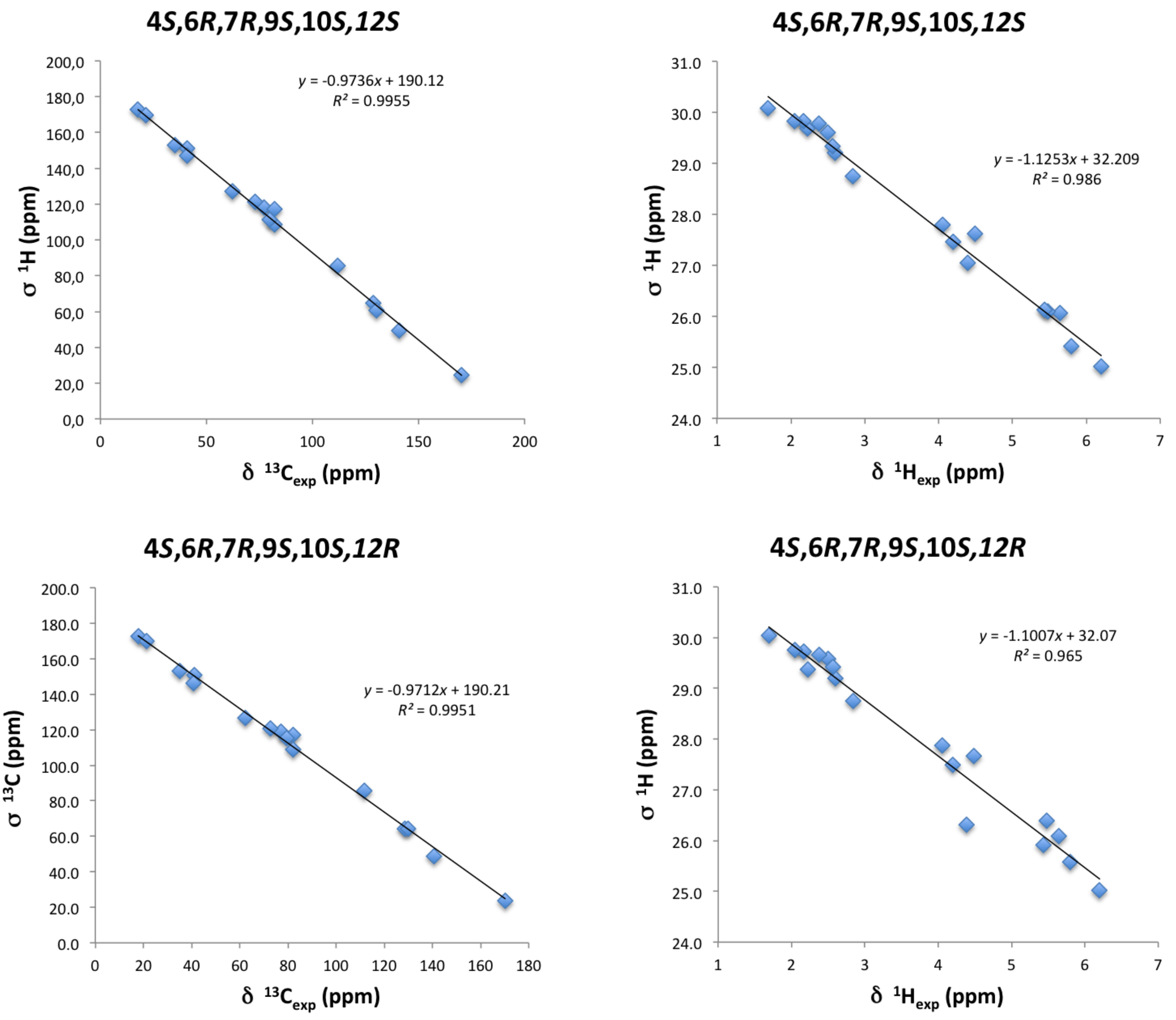

Table S5. Calculated Energies (Hartrees) for diastereoisomer S_a,4*S*,6*R*,7*R*,9*S*,10*S* of **1**.

Entry ID	Gas Phase Energy
1	−849,889,732
2	−849,888,667
3	−849,888,966
4	−84,988,891
5	−849,888,974
6	−849,888,926
7	−849,888,972
8	−849,889,026
9	−849,888,969
10	−849,888,891
11	−849,887,938
12	−849,886,933
13	−849,889,308
14	−849,888,746
15	−849,889,561
16	−849,885,682
17	−849,888,414
18	−84,988,757
19	−849,886,403
20	−849,889,367
21	−849,889,111
22	−849,887,528
23	−849,887,684
24	−849,888,722
25	−849,886,912
26	−849,888,443
27	−84,988,468
28	−849,886,486
29	−849,889,128
30	−849,888,472
31	−849,888,025
32	−849,886,947
33	−849,888,084
34	−849,887,247
35	−849,885,538
36	−849,884,153
37	−849,891,294
38	−849,887,349
39	−849,887,769
40	−849,887,456
41	−849,884,487
42	−849,884,354
43	−849,884,733
44	−849,884,722
45	−849,883,607

Table S5. *Cont.*

46	−849,887,933
47	−849,886,773
48	−849,884,928
49	−849,887,161
50	−849,888,234
51	−849,885,235
52	−849,886,184
53	−849,887,786
54	−849,886,996
55	−849,887,917
56	−849,889,128
57	−849,884,162
58	−849,884,633
59	−849,886,797
60	−84,988,321
61	−849,885,029
62	−849,884,135
63	−849,884,101
64	−849,883,757
65	−849,887,386
66	−849,888,699
67	−849,883,317
68	−849,883,094
69	−849,887,465

Table S6. Coordinates (Angstroms) of calculated geometries for diastereoisomer S_a,4*S*,6*R*,7*R*,9*S*,10*S* of **1**.

**Marilzafurollene_A_1**			
C	−0.83250	−3.28330	−0.59460
O	−2.12440	−2.71510	−0.89840
C	−0.66080	−4.68810	−1.19310
C	−0.63790	−3.16120	0.91540
C	−2.61620	−2.05240	0.28790
C	−1.42240	−1.90560	1.23820
C	−3.35450	−0.77040	−0.09270
Cl	−0.41540	−0.45810	0.92620
C	−4.62620	−1.03170	−0.90860
C	−0.83370	−4.72950	−2.72030
Br	−1.98070	−5.94670	−0.43350
C	0.24120	−3.97420	−3.45540
C	0.00500	−2.88900	−4.20890
C	1.11880	−2.18700	−4.90980
C	0.75570	−0.98210	−5.73730
O	2.28530	−2.56060	−4.79750
C	−5.71960	−1.66540	−0.10150
O	−5.12420	0.22950	−1.36010
C	−6.17620	−2.87270	−0.27490
C	−6.62750	−4.07550	−0.43690
H	−7.39630	−4.35450	−1.14500
Br	−6.02170	−5.38290	0.71160
H	−0.10020	−2.62190	−1.07460
H	0.31240	−5.10280	−0.90650
H	−1.08880	−4.00080	1.45380
H	0.41840	−3.12350	1.19990
H	−3.31850	−2.75540	0.75300
H	−1.73270	−1.85470	2.28550
H	−3.60160	−0.19280	0.80750
H	−2.70500	−0.13030	−0.70360
H	−4.39090	−1.62620	−1.79910
H	−0.79420	−5.76930	−3.07110
H	−1.82730	−4.35650	−3.00160
H	1.25700	−4.35760	−3.36280
H	−0.99300	−2.48160	−4.32540
H	1.02360	−0.07470	−5.19030
H	1.29960	−1.02160	−6.68520
H	−0.31380	−0.96370	−5.96400
H	−6.14890	−1.06280	0.69470
H	−5.89080	0.03660	−1.92840

Table S6. *Cont.*

Marilzafurollene_A_2			
C	−0.83310	−3.28290	−0.59440
O	−2.12420	−2.71530	−0.89870
C	−0.65790	−4.68700	−1.19150
C	−0.63600	−3.15840	0.91460
C	−2.61580	−2.05250	0.28790
C	−1.42060	−1.90270	1.23610
C	−3.35700	−0.77180	−0.09080
Cl	−0.41370	−0.45650	0.91720
C	−4.63090	−1.03380	−0.90270
C	−0.80340	−4.67080	−2.72290
Br	−1.99790	−5.93380	−0.44890
C	−0.33080	−5.94290	−3.38030
C	−1.12320	−6.74980	−4.10290
C	−0.58600	−7.99490	−4.72420
C	−1.56490	−8.86190	−5.47170
O	0.60620	−8.28840	−4.65130
C	−5.71750	−1.67900	−0.09560
O	−5.13780	0.22870	−1.34050
C	−6.18590	−2.87840	−0.29110
C	−6.65010	−4.07330	−0.47450
H	−7.42790	−4.32940	−1.18130
Br	−6.06460	−5.40510	0.65640
H	−0.10080	−2.62700	−1.08420
H	0.31290	−5.09910	−0.89390
H	−1.08580	−3.99810	1.45420
H	0.42070	−3.11980	1.19750
H	−3.31610	−2.75660	0.75470
H	−1.72880	−1.84900	2.28390
H	−3.60190	−0.19440	0.81010
H	−2.71020	−0.13070	−0.70350
H	−4.39610	−1.61990	−1.79880
H	−1.84020	−4.45280	−3.01010
H	−0.19210	−3.85730	−3.13410
H	0.72190	−6.19420	−3.25390
H	−2.17500	−6.53370	−4.25130
H	−1.48080	−8.66230	−6.54280
H	−1.34000	−9.91300	−5.26950
H	−2.59080	−8.67050	−5.14550
H	−6.13430	−1.09090	0.71800
H	−5.89500	0.03570	−1.92140

Table S6. *Cont.*

**Marilzafurollene_A_3**			
C	−0.83310	−3.28300	−0.59450
O	−2.12410	−2.71530	−0.89850
C	−0.65510	−4.68240	−1.20130
C	−0.63920	−3.16520	0.91520
C	−2.61590	−2.05250	0.28780
C	−1.42260	−1.90940	1.23990
C	−3.35110	−0.76830	−0.09100
Cl	−0.41140	−0.46390	0.93060
C	−4.61550	−1.02210	−0.92040
C	−0.81760	−4.65660	−2.73080
Br	−1.97740	−5.94390	−0.45360
C	−0.34060	−5.91890	−3.40350
C	−1.13180	−6.72220	−4.13150
C	−0.58840	−7.95440	−4.77290
C	−1.56130	−8.81350	−5.53710
O	0.60490	−8.24430	−4.70230
C	−5.71140	−1.67700	−0.13410
O	−5.11800	0.24470	−1.35060
C	−6.19010	−2.86640	−0.36270
C	−6.66510	−4.05080	−0.58250
H	−7.43960	−4.27970	−1.30210
Br	−6.06230	−5.43050	0.47930
H	−0.10100	−2.62340	−1.07980
H	0.32140	−5.08980	−0.91620
H	−1.09210	−4.00570	1.45080
H	0.41690	−3.12960	1.20080
H	−3.32080	−2.75450	0.75100
H	−1.73360	−1.85950	2.28700
H	−3.60530	−0.19660	0.81090
H	−2.69670	−0.12380	−0.69190
H	−4.36960	−1.59820	−1.82000
H	−1.85930	−4.44580	−3.00520
H	−0.21800	−3.83460	−3.14240
H	0.71440	−6.16440	−3.28600
H	−2.18550	−6.51060	−4.27240
H	−1.44710	−8.62420	−6.60730
H	−1.35720	−9.86580	−5.32010
H	−2.59260	−8.60390	−5.24060
H	−6.13030	−1.10330	0.68870
H	−5.87170	0.05760	−1.93780

Table S6. *Cont.*

**Marilzafurollene_A_4**			
C	−0.83310	−3.28300	−0.59450
O	−2.12410	−2.71530	−0.89850
C	−0.65310	−4.68090	−1.20400
C	−0.64110	−3.16840	0.91570
C	−2.61590	−2.05250	0.28790
C	−1.42350	−1.91220	1.24150
C	−3.34830	−0.76660	−0.09070
Cl	−0.41040	−0.46730	0.93590
C	−4.61390	−1.01760	−0.91900
C	−0.81270	−4.65220	−2.73370
Br	−1.97560	−5.94550	−0.46160
C	−0.33510	−5.91390	−3.40700
C	−1.12760	−6.72010	−4.13040
C	−0.58500	−7.95380	−4.76950
C	−1.56230	−8.82350	−5.51580
O	0.61040	−8.23740	−4.71000
C	−5.71120	−1.66780	−0.13060
O	−5.11280	0.25000	−1.35110
C	−6.18480	−2.86140	−0.34810
C	−6.65480	−4.04980	−0.55620
H	−7.42770	−4.28910	−1.27430
Br	−6.05170	−5.41540	0.52360
H	−0.10080	−2.62190	−1.07750
H	0.32320	−5.08820	−0.91800
H	−1.09570	−4.00940	1.44890
H	0.41470	−3.13450	1.20280
H	−3.32240	−2.75360	0.74980
H	−1.73560	−1.86370	2.28830
H	−3.60080	−0.19410	0.81130
H	−2.69270	−0.12370	−0.69210
H	−4.37050	−1.59610	−1.81780
H	−1.85380	−4.44060	−3.00970
H	−0.21200	−3.82980	−3.14300
H	0.72070	−6.15750	−3.29250
H	−2.18230	−6.51100	−4.26700
H	−1.35660	−9.87260	−5.28520
H	−2.59200	−8.61000	−5.21670
H	−1.45430	−8.64920	−6.58920
H	−6.13310	−1.08810	0.68630
H	−5.86710	0.06410	−1.93810

Table S6. *Cont.*

Marilzafurollene_A_5			
C	−0.83310	−3.28300	−0.59450
O	−2.12410	−2.71530	−0.89850
C	−0.65540	−4.68270	−1.20060
C	−0.63850	−3.16460	0.91510
C	−2.61590	−2.05260	0.28780
C	−1.42240	−1.90910	1.23980
C	−3.35100	−0.76820	−0.09060
Cl	−0.41160	−0.46340	0.93040
C	−4.61450	−1.02080	−0.92160
C	−0.81890	−4.65760	−2.73010
Br	−1.97690	−5.94420	−0.45150
C	−0.34110	−5.91960	−3.40270
C	−1.13170	−6.72340	−4.13080
C	−0.58750	−7.95530	−4.77200
C	−1.56010	−8.81590	−5.53500
O	0.60600	−8.24430	−4.70170
C	−5.70960	−1.68150	−0.13900
O	−5.11920	0.24700	−1.34650
C	−6.19810	−2.86360	−0.38440
C	−6.68470	−4.03980	−0.62220
H	−7.46200	−4.24950	−1.34470
Br	−6.09630	−5.44230	0.41740
H	−0.10110	−2.62370	−1.08030
H	0.32140	−5.08980	−0.91590
H	−1.09060	−4.00510	1.45120
H	0.41780	−3.12840	1.20010
H	−3.32080	−2.75460	0.75090
H	−1.73330	−1.85920	2.28690
H	−3.60610	−0.19740	0.81160
H	−2.69620	−0.12290	−0.69020
H	−4.36670	−1.59240	−1.82360
H	−1.86100	−4.44800	−3.00390
H	−0.22030	−3.83510	−3.14230
H	0.71410	−6.16460	−3.28490
H	−2.18540	−6.51220	−4.27180
H	−1.43990	−8.63440	−6.60590
H	−1.36110	−9.86740	−5.30960
H	−2.59210	−8.60020	−5.24540
H	−6.12280	−1.11670	0.69270
H	−5.87340	0.06120	−1.93350

Table S6. *Cont.*

**Marilzafurollene_A_6**			
C	−0.83310	−3.28300	−0.59450
O	−2.12410	−2.71530	−0.89850
C	−0.65640	−4.68370	−1.19860
C	−0.63750	−3.16280	0.91490
C	−2.61590	−2.05260	0.28780
C	−1.42190	−1.90750	1.23890
C	−3.35270	−0.76920	−0.09070
Cl	−0.41230	−0.46150	0.92760
C	−4.61610	−1.02370	−0.92130
C	−0.81960	−4.66070	−2.72810
Br	−1.97990	−5.94280	−0.44810
C	−0.34290	−5.92430	−3.39860
C	−1.13730	−6.73440	−4.11540
C	−0.59560	−7.96830	−4.75490
C	−1.57720	−8.84720	−5.48450
O	0.60210	−8.24470	−4.70930
C	−5.71020	−1.68520	−0.13790
O	−5.12240	0.24330	−1.34670
C	−6.19700	−2.86830	−0.38220
C	−6.68190	−4.04540	−0.61900
H	−7.45830	−4.25700	−1.34180
Br	−6.09620	−5.44490	0.42630
H	−0.10110	−2.62460	−1.08160
H	0.31990	−5.09150	−0.91310
H	−1.08850	−4.00310	1.45210
H	0.41900	−3.12560	1.19900
H	−3.31980	−2.75510	0.75170
H	−1.73220	−1.85690	2.28620
H	−3.60840	−0.19860	0.81160
H	−2.69890	−0.12320	−0.69050
H	−4.36800	−1.59550	−1.82300
H	−1.86150	−4.45060	−3.00240
H	−0.22040	−3.83940	−3.14180
H	0.71410	−6.16490	−3.28920
H	−2.19330	−6.52830	−4.24650
H	−1.35830	−9.89430	−5.25700
H	−2.60360	−8.64280	−5.16780
H	−1.48890	−8.67240	−6.55960
H	−6.12450	−1.12010	0.69300
H	−5.87680	0.05630	−1.93300

Table S6. *Cont.*

**Marilzafurollene_A_7**			
C	−0.83310	−3.28300	−0.59450
O	−2.12410	−2.71530	−0.89850
C	−0.65340	−4.68110	−1.20370
C	−0.64060	−3.16770	0.91560
C	−2.61590	−2.05250	0.28780
C	−1.42320	−1.91160	1.24120
C	−3.34900	−0.76690	−0.09070
Cl	−0.41060	−0.46660	0.93480
C	−4.61350	−1.01820	−0.92060
C	−0.81540	−4.65280	−2.73330
Br	−1.97400	−5.94580	−0.45870
C	−0.33630	−5.91310	−3.40810
C	−1.12600	−6.71670	−4.13740
C	−0.58060	−7.94700	−4.78070
C	−1.55310	−8.80820	−5.54300
O	0.61370	−8.23350	−4.71320
C	−5.71030	−1.67250	−0.13510
O	−5.11430	0.24970	−1.34990
C	−6.18690	−2.86320	−0.36130
C	−6.65990	−4.04890	−0.57860
H	−7.43360	−4.28060	−1.29820
Br	−6.05780	−5.42490	0.48840
H	−0.10090	−2.62220	−1.07800
H	0.32360	−5.08780	−0.91910
H	−1.09490	−4.00860	1.44940
H	0.41520	−3.13340	1.20240
H	−3.32210	−2.75380	0.75000
H	−1.73510	−1.86290	2.28810
H	−3.60270	−0.19530	0.81140
H	−2.69330	−0.12320	−0.69100
H	−4.36810	−1.59380	−1.82070
H	−1.85740	−4.44310	−3.00770
H	−0.21690	−3.82910	−3.14320
H	0.71920	−6.15700	−3.29100
H	−2.18010	−6.50680	−4.27780
H	−1.44800	−8.61200	−6.61290
H	−1.34040	−9.86030	−5.33360
H	−2.58360	−8.60740	−5.23740
H	−6.13080	−1.09780	0.68620
H	−5.86820	0.06410	−1.93730

Table S6. *Cont.*

**Marilzafurollene_A_8**			
C	−0.83310	−3.28300	−0.59450
O	−2.12410	−2.71530	−0.89850
C	−0.65200	−4.68000	−1.20590
C	−0.64190	−3.17010	0.91590
C	−2.61590	−2.05250	0.28790
C	−1.42390	−1.91390	1.24240
C	−3.34670	−0.76550	−0.09040
Cl	−0.40990	−0.46910	0.93920
C	−4.61210	−1.01430	−0.91970
C	−0.81310	−4.64940	−2.73550
Br	−1.97180	−5.94710	−0.46360
C	−0.33170	−5.90800	−3.41190
C	−1.11950	−6.71110	−4.14370
C	−0.57180	−7.93970	−4.78840
C	−1.54200	−8.79960	−5.55520
O	0.62250	−8.22570	−4.71860
C	−5.71040	−1.66460	−0.13280
O	−5.10970	0.25440	−1.35010
C	−6.18940	−2.85480	−0.35690
C	−6.66400	−4.04020	−0.57180
H	−7.43960	−4.27220	−1.28930
Br	−6.05320	−5.41700	0.48910
H	−0.10080	−2.62100	−1.07630
H	0.32520	−5.08620	−0.92130
H	−1.09740	−4.01130	1.44800
H	0.41370	−3.13690	1.20370
H	−3.32350	−2.75310	0.74890
H	−1.73680	−1.86630	2.28910
H	−3.59890	−0.19320	0.81170
H	−2.69000	−0.12310	−0.69110
H	−4.36870	−1.59160	−1.81920
H	−1.85520	−4.44090	−3.01020
H	−0.21550	−3.82420	−3.14370
H	0.72390	−6.15110	−3.29360
H	−2.17360	−6.50210	−4.28550
H	−1.32620	−9.85220	−5.35160
H	−2.57290	−8.60340	−5.24830
H	−1.43750	−8.59740	−6.62400
H	−6.12850	−1.08810	0.68840
H	−5.86480	0.07010	−1.93650

Table S6. *Cont.*

Marilzafurollene_A_9			
C	−0.83310	−3.28300	−0.59450
O	−2.12410	−2.71530	−0.89850
C	−0.65520	−4.68260	−1.20090
C	−0.63890	−3.16490	0.91520
C	−2.61590	−2.05250	0.28780
C	−1.42240	−1.90910	1.23970
C	−3.35140	−0.76840	−0.09080
Cl	−0.41150	−0.46360	0.93010
C	−4.61550	−1.02220	−0.92060
C	−0.81850	−4.65750	−2.73030
Br	−1.97680	−5.94400	−0.45190
C	−0.34240	−5.92030	−3.40250
C	−1.13470	−6.72440	−4.12860
C	−0.59230	−7.95730	−4.76940
C	−1.56630	−8.81670	−5.53200
O	0.60090	−8.24760	−4.69960
C	−5.71110	−1.67870	−0.13510
O	−5.11890	0.24470	−1.34960
C	−6.18980	−2.86770	−0.36580
C	−6.66480	−4.05160	−0.58780
H	−7.43900	−4.27930	−1.30830
Br	−6.06300	−5.43310	0.47210
H	−0.10100	−2.62360	−1.08010
H	0.32160	−5.08970	−0.91610
H	−1.09160	−4.00530	1.45100
H	0.41720	−3.12920	1.20060
H	−3.32060	−2.75460	0.75110
H	−1.73330	−1.85910	2.28690
H	−3.60610	−0.19710	0.81130
H	−2.69710	−0.12340	−0.69130
H	−4.36910	−1.59720	−1.82070
H	−1.86030	−4.44650	−3.00420
H	−0.21880	−3.83590	−3.14260
H	0.71270	−6.16590	−3.28610
H	−2.18860	−6.51270	−4.26830
H	−2.59720	−8.60670	−5.23440
H	−1.45320	−8.62810	−6.60250
H	−1.36210	−9.86890	−5.31460
H	−6.13030	−1.10630	0.68850
H	−5.87360	0.05780	−1.93550

Table S6. *Cont.*

**Marilzafurollene_A_10**			
C	−0.83310	−3.28300	−0.59450
O	−2.12410	−2.71530	−0.89850
C	−0.65620	−4.68380	−1.19820
C	−0.63730	−3.16240	0.91480
C	−2.61590	−2.05260	0.28780
C	−1.42190	−1.90720	1.23870
C	−3.35330	−0.76940	−0.09040
Cl	−0.41230	−0.46110	0.92700
C	−4.61600	−1.02390	−0.92190
C	−0.81960	−4.66170	−2.72770
Br	−1.97880	−5.94330	−0.44700
C	−0.34550	−5.92710	−3.39660
C	−1.14110	−6.73590	−4.11350
C	−0.60210	−7.97320	−4.74840
C	−1.58320	−8.84710	−5.48470
O	0.59360	−8.25670	−4.69330
C	−5.70950	−1.68870	−0.14060
O	−5.12380	0.24330	−1.34500
C	−6.19580	−2.87110	−0.38880
C	−6.68020	−4.04770	−0.62950
H	−7.45620	−4.25720	−1.35330
Br	−6.09690	−5.44970	0.41380
H	−0.10120	−2.62470	−1.08190
H	0.32020	−5.09120	−0.91280
H	−1.08830	−4.00270	1.45230
H	0.41910	−3.12520	1.19900
H	−3.31950	−2.75520	0.75190
H	−1.73200	−1.85650	2.28610
H	−3.60970	−0.19950	0.81210
H	−2.69950	−0.12250	−0.68940
H	−4.36650	−1.59340	−1.82480
H	−1.86120	−4.45000	−3.00200
H	−0.21900	−3.84190	−3.14210
H	0.71050	−6.17110	−3.28430
H	−2.19610	−6.52640	−4.24740
H	−1.47770	−8.68210	−6.55980
H	−1.37930	−9.89460	−5.24560
H	−2.61170	−8.62850	−5.18490
H	−6.12440	−1.12640	0.69190
H	−5.87770	0.05640	−1.93200

Table S6. *Cont.*

**Marilzafurollene_A_11**			
C	−0.83220	−3.28330	−0.59430
O	−2.12490	−2.71510	−0.89900
C	−0.67780	−4.70800	−1.14950
C	−0.61620	−3.12110	0.90960
C	−2.61600	−2.05240	0.28810
C	−1.41080	−1.86810	1.21600
C	−3.38430	−0.79070	−0.09970
Cl	−0.42980	−0.41520	0.84930
C	−4.67890	−1.09190	−0.86620
C	−0.83930	−4.79410	−2.67600
Br	−2.02390	−5.92120	−0.36120
C	0.26100	−4.09010	−3.42430
C	0.05620	−3.04570	−4.24180
C	1.19520	−2.39670	−4.95330
C	0.86380	−1.24940	−5.87080
O	2.35570	−2.76480	−4.77830
C	−5.75280	−1.66760	0.00620
O	−5.20190	0.13750	−1.37450
C	−6.13690	−2.91230	0.00670
C	−6.52040	−4.14900	0.01400
H	−7.24560	−4.57160	−0.66830
Br	−5.87370	−5.24260	1.34840
H	−0.10170	−2.64120	−1.10230
H	0.28670	−5.12900	−0.84320
H	−1.04650	−3.95420	1.47460
H	0.44400	−3.06370	1.17610
H	−3.29540	−2.76460	0.77180
H	−1.70480	−1.79540	2.26660
H	−3.61220	−0.19430	0.79320
H	−2.76110	−0.15860	−0.74510
H	−4.48550	−1.74440	−1.72560
H	−0.82200	−5.84530	−2.99270
H	−1.82090	−4.40570	−2.97770
H	1.26980	−4.47890	−3.28720
H	−0.93370	−2.63610	−4.40800
H	1.08780	−0.30660	−5.36580
H	1.46140	−1.33580	−6.78270
H	−0.19100	−1.26440	−6.15840
H	−6.24510	−0.97860	0.68720
H	−4.57510	0.44880	−2.05060

Table S6. *Cont.*

**Marilzafurollene_A_12**			
C	−0.83300	−3.28300	−0.59430
O	−2.12440	−2.71530	−0.89880
C	−0.66530	−4.69380	−1.17750
C	−0.62570	−3.14050	0.91180
C	−2.61580	−2.05260	0.28790
C	−1.41500	−1.88570	1.22590
C	−3.36830	−0.78130	−0.09940
Cl	−0.42030	−0.43660	0.88100
C	−4.64540	−1.06210	−0.90210
C	−0.82990	−4.69290	−2.70700
Br	−1.99620	−5.93300	−0.40710
C	−0.36300	−5.97030	−3.35770
C	−1.16310	−6.78470	−4.06330
C	−0.63050	−8.03370	−4.68080
C	−1.61660	−8.91250	−5.40450
O	0.56370	−8.32230	−4.62230
C	−5.73610	−1.66820	−0.07250
O	−5.16060	0.18120	−1.38380
C	−6.13420	−2.90650	−0.14180
C	−6.53280	−4.13670	−0.20750
H	−7.24970	−4.51240	−0.92510
Br	−5.93690	−5.30570	1.08590
H	−0.10210	−2.63460	−1.09620
H	0.30880	−5.10310	−0.88690
H	−1.06630	−3.97690	1.46400
H	0.43280	−3.09300	1.18650
H	−3.30670	−2.75960	0.76320
H	−1.71550	−1.82170	2.27530
H	−3.61330	−0.19650	0.79660
H	−2.72750	−0.14290	−0.72090
H	−4.43080	−1.68720	−1.77680
H	−1.87050	−4.47940	−2.98360
H	−0.22500	−3.88260	−3.13370
H	0.69150	−6.21880	−3.24190
H	−2.21690	−6.57150	−4.20060
H	−1.41050	−9.95850	−5.16050
H	−2.64280	−8.69260	−5.09790
H	−1.51910	−8.75420	−6.48140
H	−6.23280	−1.00770	0.63300
H	−4.52450	0.50910	−2.04330

Table S6. *Cont.*

Marilzafurollene_A_13			
C	−0.83280	−3.28320	−0.59480
O	−2.12440	−2.71480	−0.89750
C	−0.66170	−4.68740	−1.19590
C	−0.63250	−3.15690	0.91460
C	−2.61600	−2.05280	0.28710
C	−1.42350	−1.90650	1.24060
C	−3.35720	−0.77050	−0.08590
Cl	−0.41740	−0.45600	0.94120
C	−4.57120	−1.00800	−0.99150
C	−0.91470	−4.74650	−2.71150
Br	−1.91030	−5.97450	−0.36450
C	0.06000	−3.92520	−3.51150
C	−0.29980	−2.86700	−4.25460
C	0.71230	−2.09560	−5.03160
C	0.20050	−0.96630	−5.88660
O	1.91240	−2.35450	−4.95850
C	−5.66760	−1.78930	−0.33180
O	−5.11650	0.26900	−1.33050
C	−6.23590	−2.84630	−0.83850
C	−6.80450	−3.89080	−1.35100
H	−7.69810	−3.86630	−1.96030
Br	−5.97110	−5.52000	−1.14440
H	−0.10250	−2.62250	−1.07940
H	0.33380	−5.07860	−0.95700
H	−1.07600	−3.99550	1.45990
H	0.42500	−3.11380	1.19440
H	−3.32120	−2.75580	0.74900
H	−1.73660	−1.86180	2.28740
H	−3.66420	−0.23480	0.82170
H	−2.68800	−0.09210	−0.63040
H	−4.25180	−1.48430	−1.92590
H	−0.82670	−5.78400	−3.06030
H	−1.94580	−4.44330	−2.93680
H	1.10400	−4.23490	−3.47930
H	−1.33030	−2.53450	−4.31190
H	0.34070	−0.02010	−5.35830
H	0.75270	−0.95230	−6.83040
H	−0.85960	−1.09800	−6.12090
H	−6.04020	−1.40750	0.61480
H	−5.86080	0.10000	−1.93500

Table S6. *Cont.*

**Marilzafurollene_A_14**			
C	−0.83270	−3.28290	−0.59370
O	−2.12550	−2.71470	−0.89870
C	−0.68640	−4.71060	−1.14370
C	−0.61390	−3.11560	0.90970
C	−2.61490	−2.05320	0.28730
C	−1.41050	−1.86310	1.21520
C	−3.41000	−0.80410	−0.08330
Cl	−0.42950	−0.41150	0.84880
C	−4.61740	−1.08860	−0.98650
C	−0.85420	−4.80070	−2.66950
Br	−2.04160	−5.91160	−0.34720
C	0.24160	−4.09670	−3.42420
C	0.03380	−3.04030	−4.22550
C	1.16800	−2.38960	−4.94310
C	0.83460	−1.22040	−5.83180
O	2.32690	−2.77440	−4.79540
C	−5.63190	−2.01510	−0.37310
O	−5.23040	0.16470	−1.29870
C	−6.63430	−1.66990	0.38500
C	−7.63080	−1.32310	1.13580
H	−7.55500	−1.08680	2.18860
Br	−9.29190	−1.17810	0.35200
H	−0.10170	−2.64440	−1.10530
H	0.27600	−5.13680	−0.83820
H	−1.04050	−3.94830	1.47810
H	0.44690	−3.05580	1.17350
H	−3.28630	−2.77280	0.77360
H	−1.70490	−1.79080	2.26580
H	−3.73700	−0.28410	0.82640
H	−2.77220	−0.09550	−0.62670
H	−4.27880	−1.51790	−1.93630
H	−0.83760	−5.85270	−2.98370
H	−1.83740	−4.41410	−2.96830
H	1.24920	−4.49450	−3.30500
H	−0.95490	−2.61990	−4.37150
H	1.40950	−1.29970	−6.75880
H	−0.22660	−1.21260	−6.09490
H	1.08580	−0.29090	−5.31510
H	−5.44970	−3.07730	−0.51990
H	−6.08660	−0.03430	−1.71680

Table S6. *Cont.*

**Marilzafurollene_A_15**			
C	−0.83320	−3.28290	−0.59450
O	−2.12400	−2.71540	−0.89850
C	−0.65630	−4.68220	−1.20310
C	−0.63930	−3.16510	0.91520
C	−2.61590	−2.05250	0.28790
C	−1.42220	−1.90870	1.23930
C	−3.35180	−0.76890	−0.09160
Cl	−0.41120	−0.46360	0.92810
C	−4.62080	−1.02550	−0.91320
C	−0.81480	−4.65250	−2.73400
Br	−1.97820	−5.94210	−0.45290
C	−0.34280	−5.91610	−3.41550
C	0.72660	−5.96170	−4.22500
C	1.21360	−7.18440	−4.92410
C	0.47760	−8.48740	−4.75450
O	2.21880	−7.09680	−5.63020
C	−5.71560	−1.66780	−0.11490
O	−5.12030	0.23900	−1.35360
C	−6.18560	−2.86590	−0.31460
C	−6.65140	−4.05920	−0.50380
H	−7.42420	−4.31270	−1.21700
Br	−6.04330	−5.40420	0.59880
H	−0.10080	−2.62320	−1.07910
H	0.32050	−5.08980	−0.91920
H	−1.09250	−4.00530	1.45100
H	0.41680	−3.12980	1.20080
H	−3.32000	−2.75470	0.75160
H	−1.73270	−1.85790	2.28660
H	−3.60130	−0.19380	0.80950
H	−2.69970	−0.12730	−0.69800
H	−4.38160	−1.61150	−1.80830
H	−1.86180	−4.48210	−3.01530
H	−0.24870	−3.80090	−3.13370
H	−0.93890	−6.80640	−3.23010
H	1.32150	−5.07500	−4.42480
H	0.49300	−8.79480	−3.70570
H	−0.54970	−8.38970	−5.11500
H	0.97520	−9.26210	−5.34600
H	−6.13830	−1.07800	0.69440
H	−5.87540	0.04900	−1.93810

Table S6. *Cont.*

**Marilzafurollene_A_16**			
C	−0.83250	−3.28330	−0.59440
O	−2.12480	−2.71480	−0.89820
C	−0.67920	−4.70420	−1.15970
C	−0.61990	−3.12970	0.91080
C	−2.61580	−2.05270	0.28740
C	−1.41470	−1.87810	1.22300
C	−3.38340	−0.78730	−0.09010
Cl	−0.42860	−0.42460	0.87730
C	−4.64570	−1.07220	−0.91840
C	−0.85750	−4.78150	−2.68500
Br	−2.01650	−5.92600	−0.36550
C	0.22060	−4.05300	−3.44210
C	−0.01150	−2.99010	−4.22800
C	1.10460	−2.31390	−4.95020
C	0.74610	−1.13860	−5.82120
O	2.27010	−2.68640	−4.82380
C	−5.73940	−1.83710	−0.22710
O	−5.20430	0.17460	−1.34050
C	−6.06060	−1.75630	1.03370
C	−6.38350	−1.66880	2.28500
H	−6.20890	−2.44420	3.01920
Br	−6.93110	−0.02510	2.91450
H	−0.10130	−2.63780	−1.09700
H	0.28910	−5.12490	−0.86560
H	−1.05150	−3.96540	1.47080
H	0.43990	−3.07460	1.17980
H	−3.29980	−2.76650	0.76330
H	−1.71490	−1.81480	2.27260
H	−3.64180	−0.21470	0.80960
H	−2.74820	−0.12970	−0.69760
H	−4.36620	−1.61280	−1.82960
H	−0.82740	−5.82990	−3.00980
H	−1.84910	−4.40750	−2.97210
H	1.23490	−4.43750	−3.33830
H	−1.00820	−2.58330	−4.35750
H	1.06660	−0.21590	−5.33090
H	1.24760	−1.24300	−6.78730
H	−0.33080	−1.09110	−6.00350
H	−6.43230	−2.34860	−0.89120
H	−5.55990	0.61030	−0.54550

Table S6. *Cont.*

**Marilzafurollene_A_17**			
C	−0.83320	−3.28290	−0.59460
O	−2.12400	−2.71540	−0.89840
C	−0.65580	−4.68260	−1.20130
C	−0.63870	−3.16440	0.91500
C	−2.61590	−2.05250	0.28780
C	−1.42220	−1.90840	1.23920
C	−3.35220	−0.76900	−0.09130
Cl	−0.41120	−0.46320	0.92810
C	−4.61610	−1.02410	−0.92100
C	−0.81750	−4.65660	−2.73140
Br	−1.97470	−5.94530	−0.45270
C	−0.36130	−5.92820	−3.40750
C	−1.17000	−6.68920	−4.16140
C	−0.76070	−7.94490	−4.85080
C	0.64780	−8.45640	−4.69870
O	−1.59270	−8.53890	−5.53750
C	−5.71190	−1.67910	−0.13470
O	−5.11910	0.24210	−1.35270
C	−6.17930	−2.87500	−0.35290
C	−6.64360	−4.06560	−0.56220
H	−7.40920	−4.30890	−1.28660
Br	−6.06460	−5.42170	0.54270
H	−0.10120	−2.62380	−1.08050
H	0.32140	−5.08930	−0.91750
H	−1.09120	−4.00470	1.45110
H	0.41750	−3.12840	1.20010
H	−3.32000	−2.75480	0.75160
H	−1.73260	−1.85770	2.28650
H	−3.60720	−0.19730	0.81050
H	−2.69820	−0.12400	−0.69210
H	−4.36950	−1.60080	−1.82010
H	−1.85560	−4.42740	−3.00470
H	−0.20380	−3.84450	−3.14220
H	0.68230	−6.19460	−3.26060
H	−2.21170	−6.42130	−4.31470
H	1.35680	−7.73480	−5.11260
H	0.86560	−8.65400	−3.64590
H	0.75180	−9.39550	−5.25070
H	−6.14150	−1.09950	0.67830
H	−5.87060	0.05380	−1.94240

Table S6. *Cont.*

**Marilzafurollene_A_18**			
C	−0.83300	−3.28290	−0.59430
O	−2.12430	−2.71530	−0.89880
C	−0.66610	−4.69310	−1.18050
C	−0.62600	−3.14110	0.91190
C	−2.61570	−2.05260	0.28790
C	−1.41500	−1.88600	1.22600
C	−3.36850	−0.78130	−0.09930
Cl	−0.41990	−0.43730	0.88090
C	−4.64610	−1.06230	−0.90110
C	−0.83050	−4.68760	−2.71100
Br	−1.99260	−5.93290	−0.40490
C	−0.36620	−5.96390	−3.37370
C	0.69930	−6.02650	−4.18720
C	1.17930	−7.26200	−4.86840
C	0.44010	−8.55980	−4.67420
O	2.18160	−7.18900	−5.58030
C	−5.73670	−1.66720	−0.07040
O	−5.16100	0.18090	−1.38340
C	−6.12900	−2.90790	−0.13200
C	−6.52110	−4.14050	−0.18980
H	−7.23410	−4.52510	−0.90660
Br	−5.92340	−5.29660	1.11440
H	−0.10200	−2.63390	−1.09510
H	0.30950	−5.10150	−0.89370
H	−1.06690	−3.97730	1.46400
H	0.43250	−3.09390	1.18670
H	−3.30650	−2.75970	0.76320
H	−1.71550	−1.82190	2.27540
H	−3.61280	−0.19620	0.79660
H	−2.72790	−0.14320	−0.72140
H	−4.43250	−1.68820	−1.77540
H	−1.87790	−4.51730	−2.99110
H	−0.26240	−3.84500	−3.12660
H	−0.96430	−6.84910	−3.17080
H	1.29630	−5.14520	−4.40430
H	0.46050	−8.85060	−3.62080
H	−0.58880	−8.46440	−5.03040
H	0.93190	−9.34520	−5.25620
H	−6.23790	−1.00410	0.62940
H	−4.52670	0.50630	−2.04600

Table S6. *Cont.*

Marilzafurollene_A_19			
C	−0.83300	−3.28290	−0.59430
O	−2.12430	−2.71530	−0.89870
C	−0.66710	−4.69510	−1.17540
C	−0.62410	−3.13720	0.91130
C	−2.61580	−2.05260	0.28790
C	−1.41400	−1.88230	1.22380
C	−3.37220	−0.78360	−0.09960
Cl	−0.42100	−0.43330	0.87370
C	−4.64930	−1.06850	−0.90090
C	−0.83240	−4.69650	−2.70530
Br	−1.99460	−5.93310	−0.40030
C	−0.38830	−5.98420	−3.35850
C	−1.20430	−6.75130	−4.09820
C	−0.80710	−8.02310	−4.76490
C	0.59590	−8.54620	−4.60170
O	−1.64410	−8.62030	−5.44250
C	−5.73830	−1.67620	−0.07010
O	−5.16760	0.17320	−1.38360
C	−6.12010	−2.92040	−0.12380
C	−6.50160	−4.15680	−0.17300
H	−7.20770	−4.55360	−0.89000
Br	−5.91080	−5.29330	1.15150
H	−0.10240	−2.63620	−1.09860
H	0.30770	−5.10390	−0.88630
H	−1.06330	−3.97280	1.46560
H	0.43480	−3.08830	1.18450
H	−3.30410	−2.76080	0.76510
H	−1.71290	−1.81610	2.27350
H	−3.61780	−0.19880	0.79620
H	−2.73370	−0.14390	−0.72220
H	−4.43430	−1.69410	−1.77500
H	−1.86930	−4.46390	−2.98050
H	−0.21300	−3.89740	−3.13270
H	0.65300	−6.25760	−3.20720
H	−2.24360	−6.47650	−4.25630
H	0.68920	−9.49890	−5.13200
H	1.31230	−7.84190	−5.03220
H	0.81240	−8.72230	−3.54480
H	−6.24780	−1.01200	0.62280
H	−4.53520	0.49920	−2.04760

Table S6. *Cont.*

**Marilzafurollene_A_20**			
C	−0.83390	−3.28370	−0.59780
O	−2.12020	−2.71610	−0.89470
C	−0.51190	−4.51160	−1.46060
C	−0.82940	−3.47540	0.91230
C	−2.61910	−2.05110	0.28730
C	−1.55000	−2.22050	1.37820
C	−3.02480	−0.63070	−0.10620
Cl	−0.39070	−0.85930	1.49450
C	−4.23680	−0.62220	−1.04960
C	−0.30640	−4.18440	−2.95700
Br	−1.87990	−5.91050	−1.27570
C	−1.45480	−3.49000	−3.64870
C	−1.49570	−2.16250	−3.84640
C	−2.68420	−1.44530	−4.37870
C	−3.66830	−2.17840	−5.25100
O	−2.85330	−0.26670	−4.05650
C	−5.53440	−0.73730	−0.30700
O	−4.27350	0.62620	−1.74160
C	−6.27700	−1.80610	−0.25190
C	−7.01560	−2.86830	−0.19800
H	−7.86680	−3.05730	−0.83840
Br	−6.54760	−4.18620	1.00140
H	−0.09100	−2.51420	−0.84880
H	0.40390	−4.98340	−1.08590
H	−1.42230	−4.34910	1.20430
H	0.17740	−3.59890	1.32260
H	−3.50280	−2.61940	0.59930
H	−1.99510	−2.33270	2.37080
H	−3.23800	−0.02760	0.78520
H	−2.19160	−0.14050	−0.62680
H	−4.16480	−1.41320	−1.80440
H	0.59510	−3.56350	−3.04770
H	−0.08520	−5.10470	−3.51300
H	−2.29750	−4.12130	−3.92050
H	−0.69160	−1.51440	−3.51220
H	−3.14920	−2.85800	−5.93230
H	−4.37390	−2.73160	−4.62620
H	−4.21620	−1.45350	−5.86030
H	−5.85850	0.14490	0.23820
H	−3.70750	0.50780	−2.53690

Table S6. *Cont.*

**Marilzafurollene_A_21**			
C	−0.83280	−3.28330	−0.59490
O	−2.12400	−2.71520	−0.89800
C	−0.66220	−4.68850	−1.19210
C	−0.63850	−3.16270	0.91540
C	−2.61640	−2.05230	0.28780
C	−1.42420	−1.90860	1.24070
C	−3.35200	−0.76860	−0.09200
Cl	−0.41560	−0.46080	0.93710
C	−4.62020	−1.02510	−0.91470
C	−0.83080	−4.73280	−2.72010
Br	−1.98670	−5.94380	−0.43410
C	0.26340	−4.00700	−3.46270
C	0.03510	−2.93830	−4.24180
C	1.07840	−2.20510	−5.01260
C	2.51360	−2.65710	−4.94470
O	0.73740	−1.23870	−5.69520
C	−5.71600	−1.66700	−0.11740
O	−5.11940	0.23950	−1.35510
C	−6.18560	−2.86520	−0.31670
C	−6.65150	−4.05860	−0.50520
H	−7.42210	−4.31290	−1.22050
Br	−6.05580	−5.39980	0.60890
H	−0.10010	−2.62290	−1.07590
H	0.30910	−5.10530	−0.90230
H	−1.08850	−4.00340	1.45280
H	0.41790	−3.12480	1.19990
H	−3.32100	−2.75450	0.75090
H	−1.73670	−1.86150	2.28750
H	−3.60230	−0.19370	0.80900
H	−2.69930	−0.12700	−0.69780
H	−4.38020	−1.61100	−1.80960
H	−0.81160	−5.77520	−3.06510
H	−1.81650	−4.34250	−3.00560
H	1.26400	−4.41780	−3.35190
H	−0.96640	−2.53220	−4.35820
H	2.60600	−3.67630	−5.32850
H	3.12840	−1.99960	−5.56690
H	2.87960	−2.59510	−3.91660
H	−6.14060	−1.07610	0.69000
H	−5.86840	0.04930	−1.94740

Table S6. *Cont.*

**Marilzafurollene_A_22**			
C	−0.83270	−3.28320	−0.59460
O	−2.12430	−2.71520	−0.89840
C	−0.66560	−4.69290	−1.18240
C	−0.63140	−3.14950	0.91370
C	−2.61610	−2.05240	0.28780
C	−1.41890	−1.89460	1.23190
C	−3.36050	−0.77630	−0.09930
Cl	−0.41860	−0.44590	0.90290
C	−4.63360	−1.04780	−0.91140
C	−0.84260	−4.74810	−2.70920
Br	−1.98550	−5.94280	−0.40830
C	0.23100	−4.00110	−3.46080
C	−0.02590	−2.93650	−4.23650
C	0.99640	−2.17430	−5.00680
C	2.44280	−2.58880	−4.94220
O	0.62880	−1.21350	−5.68350
C	−5.73230	−1.65370	−0.09220
O	−5.14050	0.20010	−1.39000
C	−6.12740	−2.89300	−0.16290
C	−6.52200	−4.12440	−0.22990
H	−7.23030	−4.50370	−0.95410
Br	−5.93610	−5.28860	1.07240
H	−0.10110	−2.62730	−1.08310
H	0.30770	−5.10700	−0.89550
H	−1.07640	−3.98710	1.46020
H	0.42610	−3.10610	1.19280
H	−3.31270	−2.75690	0.75850
H	−1.72420	−1.83690	2.28020
H	−3.60840	−0.19360	0.79740
H	−2.71280	−0.13860	−0.71430
H	−4.41600	−1.66920	−1.78800
H	−0.80440	−5.79160	−3.04910
H	−1.83790	−4.37910	−2.99000
H	1.24120	−4.38900	−3.35510
H	−1.03710	−2.55380	−4.34700
H	2.56140	−3.60380	−5.32980
H	3.03950	−1.91320	−5.56280
H	2.80850	−2.52100	−3.91440
H	−6.23690	−0.99270	0.60710
H	−4.49920	0.52820	−2.04450

Table S6. *Cont.*

**Marilzafurollene_A_23**			
C	−0.83320	−3.28260	−0.59360
O	−2.12520	−2.71490	−0.89890
C	−0.67870	−4.70450	−1.15400
C	−0.61700	−3.12210	0.91020
C	−2.61470	−2.05330	0.28730
C	−1.41110	−1.86840	1.21780
C	−3.40530	−0.80090	−0.08200
Cl	−0.42450	−0.41940	0.85540
C	−4.61050	−1.07820	−0.99040
C	−0.82480	−4.72440	−2.68530
Br	−2.04210	−5.90900	−0.38000
C	−0.37430	−6.01980	−3.31140
C	−1.17500	−6.81990	−4.03260
C	−0.65650	−8.08600	−4.62720
C	−1.63720	−8.93330	−5.39470
O	0.52420	−8.41280	−4.51650
C	−5.62770	−2.00770	−0.38610
O	−5.22160	0.17800	−1.29460
C	−6.63590	−1.66650	0.36620
C	−7.63820	−1.32420	1.11120
H	−7.57030	−1.09170	2.16540
Br	−9.29490	−1.18330	0.31720
H	−0.10190	−2.64490	−1.10820
H	0.28510	−5.12490	−0.84540
H	−1.04780	−3.95620	1.47360
H	0.44330	−3.06610	1.17680
H	−3.28900	−2.77170	0.77130
H	−1.70660	−1.79760	2.26820
H	−3.73380	−0.28330	0.82850
H	−2.76380	−0.09210	−0.62050
H	−4.26970	−1.50110	−1.94220
H	−1.85770	−4.49540	−2.97770
H	−0.19930	−3.93210	−3.11620
H	0.67020	−6.29370	−3.16540
H	−2.21830	−6.58060	−4.20290
H	−1.48130	−8.78500	−6.46620
H	−1.48130	−9.98450	−5.13640
H	−2.66860	−8.67470	−5.14040
H	−5.44340	−3.06910	−0.53540
H	−6.07120	−0.01740	−1.72760

Table S6. *Cont.*

**Marilzafurollene_A_24**			
C	−0.83290	−3.28320	−0.59490
O	−2.12390	−2.71520	−0.89790
C	−0.66000	−4.68440	−1.20070
C	−0.64230	−3.16930	0.91640
C	−2.61640	−2.05230	0.28770
C	−1.42590	−1.91410	1.24360
C	−3.34670	−0.76590	−0.09290
Cl	−0.41400	−0.46710	0.94650
C	−4.61420	−1.01790	−0.91820
C	−0.82830	−4.73210	−2.72950
Br	−1.98250	−5.94510	−0.44350
C	0.24330	−3.98040	−3.47440
C	1.18020	−4.57010	−4.23340
C	2.20270	−3.75750	−4.95520
C	3.16360	−4.50070	−5.84560
O	2.27390	−2.53820	−4.81090
C	−5.71430	−1.65400	−0.12220
O	−5.10740	0.24840	−1.36090
C	−6.18780	−2.85110	−0.31970
C	−6.65710	−4.04340	−0.50650
H	−7.42820	−4.29670	−1.22160
Br	−6.05690	−5.38700	0.60220
H	−0.09950	−2.61870	−1.06840
H	0.31170	−5.10110	−0.91210
H	−1.09620	−4.01050	1.44980
H	0.41330	−3.13470	1.20420
H	−3.32450	−2.75260	0.74830
H	−1.74060	−1.86930	2.28990
H	−3.59670	−0.19040	0.80770
H	−2.69060	−0.12640	−0.69730
H	−4.37500	−1.60590	−1.81190
H	−0.83170	−5.77790	−3.06580
H	−1.80380	−4.32340	−3.02260
H	0.21970	−2.89340	−3.39910
H	1.22770	−5.64640	−4.35250
H	2.78520	−5.49600	−6.09390
H	3.28810	−3.94600	−6.77980
H	4.12610	−4.59700	−5.33720
H	−6.13770	−1.06030	0.68380
H	−5.85530	0.06030	−1.95510

Table S6. *Cont.*

**Marilzafurollene_A_25**			
C	−0.83280	−3.28320	−0.59480
O	−2.12410	−2.71520	−0.89810
C	−0.66850	−4.69350	−1.18200
C	−0.63040	−3.14800	0.91360
C	−2.61620	−2.05240	0.28770
C	−1.41940	−1.89410	1.23210
C	−3.36070	−0.77680	−0.10110
Cl	−0.42160	−0.44350	0.90550
C	−4.63300	−1.05000	−0.91400
C	−0.84840	−4.76260	−2.70860
Br	−1.98980	−5.93930	−0.39790
C	0.21270	−4.01530	−3.47260
C	1.14770	−4.60960	−4.23040
C	2.15850	−3.79980	−4.97160
C	3.12140	−4.54910	−5.85470
O	2.21910	−2.57770	−4.84780
C	−5.73330	−1.65180	−0.09370
O	−5.13820	0.19670	−1.39780
C	−6.12280	−2.89350	−0.15270
C	−6.51160	−4.12730	−0.20820
H	−7.21520	−4.51720	−0.93140
Br	−5.93470	−5.27280	1.11460
H	−0.10070	−2.62770	−1.08240
H	0.30410	−5.10900	−0.89460
H	−1.07320	−3.98570	1.46150
H	0.42740	−3.10290	1.19150
H	−3.31290	−2.75670	0.75850
H	−1.72540	−1.83780	2.28040
H	−3.60960	−0.19340	0.79470
H	−2.71270	−0.13960	−0.71630
H	−4.41540	−1.67490	−1.78810
H	−0.84920	−5.81290	−3.03060
H	−1.82830	−4.36280	−2.99940
H	0.18260	−2.92750	−3.41310
H	1.20200	−5.68710	−4.33440
H	3.24120	−4.00540	−6.79600
H	4.08550	−4.63420	−5.34730
H	2.74730	−5.54910	−6.09010
H	−6.24330	−0.98570	0.59680
H	−4.49530	0.52130	−2.05250

Table S6. *Cont.*

**Marilzafurollene_A_26**			
C	−0.83320	−3.28290	−0.59440
O	−2.12410	−2.71530	−0.89850
C	−0.65750	−4.68460	−1.19790
C	−0.63530	−3.15860	0.91440
C	−2.61580	−2.05260	0.28780
C	−1.42020	−1.90290	1.23600
C	−3.35690	−0.77210	−0.09170
Cl	−0.41300	−0.45670	0.91720
C	−4.62370	−1.03330	−0.91510
C	−0.82290	−4.66160	−2.72810
Br	−1.97660	−5.94110	−0.43660
C	−0.31150	−5.90820	−3.40730
C	0.72180	−5.92180	−4.26410
C	1.16630	−7.19120	−4.91130
C	2.30950	−7.09610	−5.88740
O	0.64120	−8.27030	−4.64290
C	−5.71540	−1.68580	−0.12080
O	−5.13050	0.23010	−1.35080
C	−6.19230	−2.87760	−0.34030
C	−6.66660	−4.06390	−0.55140
H	−7.43750	−4.29880	−1.27310
Br	−6.09560	−5.42760	0.54810
H	−0.10140	−2.62540	−1.08310
H	0.32060	−5.09000	−0.91510
H	−1.08470	−3.99780	1.45490
H	0.42160	−3.11980	1.19670
H	−3.31670	−2.75650	0.75410
H	−1.72830	−1.84910	2.28380
H	−3.60960	−0.19860	0.80950
H	−2.70720	−0.12710	−0.69710
H	−4.38000	−1.61410	−1.81230
H	−1.87580	−4.52510	−3.00560
H	−0.28460	−3.79400	−3.13160
H	−0.83890	−6.83680	−3.19000
H	1.26600	−5.02000	−4.51960
H	2.43520	−6.07290	−6.25190
H	3.23070	−7.42160	−5.39780
H	2.09890	−7.73550	−6.74930
H	−6.13640	−1.10590	0.69650
H	−5.88540	0.03770	−1.93490

Table S6. *Cont.*

**Marilzafurollene_A_27**			
C	−0.83310	−3.28290	−0.59420
O	−2.12460	−2.71500	−0.89840
C	−0.67380	−4.69950	−1.16610
C	−0.62100	−3.13230	0.91070
C	−2.61550	−2.05290	0.28750
C	−1.41380	−1.87940	1.22290
C	−3.38270	−0.78680	−0.08870
Cl	−0.42470	−0.42830	0.87450
C	−4.64430	−1.06940	−0.91890
C	−0.83050	−4.70910	−2.69640
Br	−2.02210	−5.92020	−0.39200
C	−0.37480	−5.99690	−3.33440
C	−1.17770	−6.80280	−4.04640
C	−0.65580	−8.06270	−4.65090
C	−1.64320	−8.92560	−5.39170
O	0.53160	−8.37260	−4.56830
C	−5.73590	−1.84220	−0.23300
O	−5.20700	0.17890	−1.33140
C	−6.05830	−1.76990	1.02810
C	−6.38240	−1.69130	2.27970
H	−6.20600	−2.47090	3.00910
Br	−6.93900	−0.05430	2.91860
H	−0.10180	−2.64010	−1.10260
H	0.29490	−5.11570	−0.86720
H	−1.05540	−3.96840	1.46810
H	0.43850	−3.07970	1.18100
H	−3.29940	−2.76680	0.76320
H	−1.71320	−1.81460	2.27260
H	−3.64200	−0.21600	0.81190
H	−2.74710	−0.12790	−0.69410
H	−4.36340	−1.60270	−1.83400
H	−1.86710	−4.48590	−2.98020
H	−0.21400	−3.90910	−3.12610
H	0.67440	−6.26040	−3.20400
H	−2.22610	−6.57410	−4.19960
H	−1.52080	−8.77310	−6.46680
H	−1.46170	−9.97430	−5.14010
H	−2.67120	−8.68550	−5.10700
H	−6.42680	−2.35160	−0.90070
H	−5.56340	0.60780	−0.53310

Table S6. *Cont.*

**Marilzafurollene_A_28**			
C	−0.83310	−3.28290	−0.59420
O	−2.12440	−2.71530	−0.89880
C	−0.66680	−4.69510	−1.17590
C	−0.62290	−3.13550	0.91110
C	−2.61570	−2.05260	0.28790
C	−1.41330	−1.88060	1.22280
C	−3.37340	−0.78460	−0.10000
Cl	−0.42180	−0.43120	0.87010
C	−4.65370	−1.07230	−0.89520
C	−0.83530	−4.69530	−2.70590
Br	−1.99240	−5.93100	−0.39240
C	−0.33430	−5.95610	−3.36620
C	0.69850	−5.99150	−4.22310
C	1.13200	−7.27450	−4.85080
C	2.27680	−7.20490	−5.82720
O	0.59620	−8.34450	−4.56720
C	−5.74140	−1.67090	−0.05620
O	−5.17050	0.16710	−1.38550
C	−6.12140	−2.91630	−0.09380
C	−6.50030	−4.15410	−0.12690
H	−7.20790	−4.56110	−0.83660
Br	−5.89830	−5.27440	1.20630
H	−0.10250	−2.63580	−1.09830
H	0.30950	−5.10190	−0.88890
H	−1.06090	−3.97050	1.46720
H	0.43620	−3.08540	1.18320
H	−3.30280	−2.76120	0.76600
H	−1.71150	−1.81340	2.27260
H	−3.61580	−0.19740	0.79510
H	−2.73720	−0.14680	−0.72670
H	−4.44320	−1.70490	−1.76540
H	−1.88770	−4.55550	−2.98350
H	−0.29160	−3.83810	−3.12430
H	−0.86920	−6.87700	−3.13460
H	1.25030	−5.09840	−4.49240
H	2.05440	−7.84440	−6.68610
H	2.42200	−6.18600	−6.19620
H	3.19180	−7.54500	−5.33570
H	−6.24980	−0.99940	0.63030
H	−4.53950	0.48620	−2.05430

Table S6. *Cont.*

**Marilzafurollene_A_29**			
C	−0.83310	−3.28300	−0.59470
O	−2.12390	−2.71530	−0.89820
C	−0.66070	−4.68420	−1.19990
C	−0.63670	−3.16050	0.91520
C	−2.61610	−2.05240	0.28780
C	−1.42240	−1.90550	1.23860
C	−3.35430	−0.77060	−0.09310
Cl	−0.41490	−0.45810	0.92730
C	−4.61930	−1.02970	−0.91990
C	−0.86740	−4.74090	−2.72390
Br	−1.94890	−5.95810	−0.40750
C	0.13230	−3.92550	−3.50570
C	1.11070	−4.47180	−4.24390
C	2.10120	−3.70390	−5.05020
C	2.02030	−2.20090	−5.10160
O	2.97160	−4.32570	−5.66030
C	−5.71410	−1.68020	−0.12830
O	−5.12250	0.23430	−1.35760
C	−6.18210	−2.87710	−0.33910
C	−6.64680	−4.06880	−0.54080
H	−7.41520	−4.31600	−1.26100
Br	−6.05690	−5.42100	0.56310
H	−0.10110	−2.62050	−1.07350
H	0.32300	−5.08810	−0.93450
H	−1.08640	−3.99920	1.45580
H	0.41990	−3.12180	1.19840
H	−3.31900	−2.75540	0.75240
H	−1.73290	−1.85500	2.28590
H	−3.60850	−0.19710	0.80770
H	−2.70200	−0.12640	−0.69660
H	−4.37440	−1.61120	−1.81630
H	−0.82090	−5.78610	−3.05930
H	−1.87390	−4.39190	−2.98910
H	−0.00280	−2.84710	−3.46480
H	1.24070	−5.54920	−4.29840
H	1.05760	−1.88920	−5.51490
H	2.16730	−1.78180	−4.10290
H	2.81170	−1.81930	−5.75400
H	−6.14110	−1.09660	0.68330
H	−5.87750	0.04340	−1.94200

Table S6. *Cont.*

**Marilzafurollene_A_30**			
C	−0.83330	−3.28280	−0.59440
O	−2.12440	−2.71490	−0.89780
C	−0.67780	−4.70370	−1.15770
C	−0.61050	−3.11760	0.90780
C	−2.61540	−2.05310	0.28700
C	−1.41040	−1.86850	1.21720
C	−3.39440	−0.79280	−0.08290
Cl	−0.42590	−0.41600	0.85870
C	−4.61400	−1.06470	−0.97110
C	−0.89810	−4.73460	−2.68030
Br	−1.96510	−5.94030	−0.31420
C	−0.47180	−6.03340	−3.32380
C	−1.31340	−6.82500	−4.00690
C	−0.93490	−8.11020	−4.65850
C	0.47370	−8.62740	−4.52850
O	−1.79090	−8.72350	−5.29680
C	−5.67760	−1.88060	−0.29940
O	−5.20270	0.19690	−1.29550
C	−6.24810	−2.93020	−0.81900
C	−6.82190	−3.96520	−1.34480
H	−7.72320	−3.93060	−1.94200
Br	−6.01780	−5.60730	−1.12660
H	−0.10530	−2.64570	−1.11450
H	0.30950	−5.10050	−0.89570
H	−1.03330	−3.95020	1.47850
H	0.45090	−3.05520	1.16840
H	−3.29570	−2.76810	0.76780
H	−1.70540	−1.80000	2.26790
H	−3.70340	−0.26140	0.82660
H	−2.75070	−0.09890	−0.63820
H	−4.29390	−1.52790	−1.91190
H	−1.94580	−4.51320	−2.92180
H	−0.29960	−3.94060	−3.14510
H	0.57770	−6.29420	−3.21340
H	−2.36140	−6.56270	−4.12390
H	0.73030	−8.76370	−3.47470
H	0.54780	−9.59990	−5.02480
H	1.17230	−7.93980	−5.01200
H	−6.03660	−1.52230	0.66150
H	−5.94270	0.00840	−1.89950

Table S6. *Cont.*

**Marilzafurollene_A_31**			
C	−0.83260	−3.28350	−0.59520
O	−2.12410	−2.71480	−0.89690
C	−0.64960	−4.67820	−1.21540
C	−0.63890	−3.17210	0.91630
C	−2.61650	−2.05250	0.28700
C	−1.42960	−1.92410	1.25160
C	−3.33350	−0.75700	−0.09000
Cl	−0.41490	−0.47330	0.97930
C	−4.52360	−0.96420	−1.03460
C	−0.94900	−4.73620	−2.72270
Br	−1.83870	−6.00570	−0.35990
C	−0.05830	−3.85140	−3.55200
C	−0.52070	−2.83140	−4.29150
C	0.39960	−1.98790	−5.10570
C	−0.23250	−0.89770	−5.93050
O	1.61740	−2.15620	−5.08200
C	−5.65240	−1.72810	−0.41500
O	−5.05500	0.31790	−1.37790
C	−6.14030	−2.84360	−0.87830
C	−6.63410	−3.94140	−1.35500
H	−7.42040	−3.98800	−2.09640
Br	−5.94610	−5.53770	−0.74800
H	−0.10360	−2.61420	−1.07030
H	0.36190	−5.04680	−1.01040
H	−1.08720	−4.01330	1.45320
H	0.41730	−3.13370	1.20120
H	−3.33640	−2.74700	0.73880
H	−1.75040	−1.89120	2.29650
H	−3.65920	−0.22870	0.81540
H	−2.64010	−0.08070	−0.60560
H	−4.19730	−1.44240	−1.96590
H	−0.81230	−5.76420	−3.08400
H	−2.00430	−4.49450	−2.90790
H	1.00670	−4.07980	−3.54710
H	−1.57590	−2.58270	−4.32050
H	0.33360	−0.77260	−6.85770
H	−1.26040	−1.15740	−6.19920
H	−0.22720	0.03610	−5.36280
H	−6.13750	−1.27080	0.44250
H	−4.37200	0.77410	−1.89950

Table S6. *Cont.*

**Marilzafurollene_A_32**			
C	−0.83290	−3.28310	−0.59450
O	−2.12420	−2.71530	−0.89850
C	−0.67150	−4.69700	−1.17300
C	−0.62180	−3.13300	0.91130
C	−2.61600	−2.05250	0.28790
C	−1.41410	−1.87920	1.22300
C	−3.37440	−0.78500	−0.10030
Cl	−0.42470	−0.42800	0.87250
C	−4.65150	−1.07220	−0.90080
C	−0.88200	−4.78140	−2.69530
Br	−1.96740	−5.94580	−0.35340
C	0.12740	−3.99510	−3.49430
C	1.09570	−4.56970	−4.22450
C	2.09580	−3.83220	−5.04710
C	2.03900	−2.32920	−5.12460
O	2.95400	−4.47860	−5.64870
C	−5.73850	−1.68230	−0.06940
O	−5.17270	0.16850	−1.38280
C	−6.12560	−2.92450	−0.13140
C	−6.51290	−4.15870	−0.18950
H	−7.22380	−4.54600	−0.90690
Br	−5.91640	−5.31100	1.11850
H	−0.10240	−2.63280	−1.09200
H	0.30980	−5.10280	−0.90210
H	−1.05760	−3.96680	1.47060
H	0.43750	−3.08080	1.18210
H	−3.30310	−2.76130	0.76590
H	−1.71300	−1.81310	2.27280
H	−3.62050	−0.19980	0.79510
H	−2.73720	−0.14480	−0.72370
H	−4.43550	−1.69710	−1.77530
H	−0.84970	−5.83350	−3.01000
H	−1.88420	−4.42450	−2.96570
H	0.00890	−2.91430	−3.47340
H	1.20910	−5.64980	−4.25940
H	2.19670	−1.89520	−4.13390
H	2.83400	−1.97200	−5.78640
H	1.07990	−2.00910	−5.53980
H	−6.24260	−1.02150	0.63060
H	−4.54080	0.49700	−2.04610

Table S6. *Cont.*

**Marilzafurollene_A_33**			
C	−0.83340	−3.28250	−0.59360
O	−2.12510	−2.71490	−0.89890
C	−0.67910	−4.70280	−1.15920
C	−0.61800	−3.12450	0.91050
C	−2.61460	−2.05340	0.28730
C	−1.41150	−1.87050	1.21900
C	−3.40380	−0.80000	−0.08210
Cl	−0.42350	−0.42190	0.85900
C	−4.60560	−1.07490	−0.99560
C	−0.83060	−4.71650	−2.69090
Br	−2.03380	−5.91140	−0.37740
C	−0.37970	−6.00880	−3.33150
C	0.69190	−6.09840	−4.13450
C	1.15940	−7.35000	−4.79480
C	0.39780	−8.63340	−4.59180
O	2.16980	−7.30190	−5.49710
C	−5.62230	−2.01070	−0.40040
O	−5.21910	0.18150	−1.29410
C	−6.62910	−1.67730	0.35720
C	−7.62990	−1.34260	1.10770
H	−7.55970	−1.12010	2.16390
Br	−9.28850	−1.19590	0.31910
H	−0.10180	−2.64340	−1.10600
H	0.28790	−5.12130	−0.85790
H	−1.04970	−3.95860	1.47300
H	0.44210	−3.06940	1.17790
H	−3.29030	−2.77120	0.77040
H	−1.70770	−1.80060	2.26920
H	−3.73530	−0.28440	0.82840
H	−2.76050	−0.08990	−0.61680
H	−4.26080	−1.49110	−1.94900
H	−1.87290	−4.53470	−2.98230
H	−0.24650	−3.88840	−3.11340
H	−0.99260	−6.88230	−3.12210
H	1.30390	−5.22910	−4.35810
H	−0.62570	−8.52640	−4.96010
H	0.88310	−9.43390	−5.15840
H	0.40250	−8.91080	−3.53450
H	−5.43770	−3.07060	−0.56050
H	−6.06670	−0.01360	−1.73110

Table S6. *Cont.*

**Marilzafurollene_A_34**			
C	−0.83340	−3.28250	−0.59370
O	−2.12510	−2.71490	−0.89880
C	−0.67940	−4.70400	−1.15560
C	−0.61690	−3.12230	0.91010
C	−2.61470	−2.05330	0.28730
C	−1.41110	−1.86860	1.21790
C	−3.40520	−0.80100	−0.08240
Cl	−0.42420	−0.41980	0.85540
C	−4.60790	−1.07800	−0.99420
C	−0.83180	−4.72310	−2.68670
Br	−2.03420	−5.91240	−0.37560
C	−0.40050	−6.02550	−3.31900
C	−1.22030	−6.79020	−4.05680
C	−0.83530	−8.07580	−4.70370
C	0.56160	−8.61150	−4.52980
O	−1.67780	−8.67430	−5.37360
C	−5.62430	−2.01180	−0.39530
O	−5.22140	0.17780	−1.29540
C	−6.62970	−1.67560	0.36300
C	−7.62900	−1.33780	1.11410
H	−7.55720	−1.11410	2.17000
Br	−9.28650	−1.18000	0.32530
H	−0.10220	−2.64490	−1.10850
H	0.28700	−5.12250	−0.85260
H	−1.04750	−3.95620	1.47370
H	0.44350	−3.06620	1.17640
H	−3.28930	−2.77160	0.77110
H	−1.70660	−1.79780	2.26820
H	−3.73620	−0.28460	0.82790
H	−2.76310	−0.09090	−0.61860
H	−4.26420	−1.49680	−1.94680
H	−1.86290	−4.47940	−2.97380
H	−0.19750	−3.93850	−3.11910
H	0.63530	−6.31180	−3.15440
H	−2.25410	−6.50290	−4.22830
H	0.76990	−8.78450	−3.47080
H	0.64900	−9.56740	−5.05540
H	1.28730	−7.91630	−4.95950
H	−5.44140	−3.07210	−0.55400
H	−6.07020	−0.01820	−1.72970

Table S6. *Cont.*

**Marilzafurollene_A_35**			
C	−0.83320	−3.28290	−0.59430
O	−2.12440	−2.71510	−0.89840
C	−0.67290	−4.69730	−1.17250
C	−0.62300	−3.13640	0.91130
C	−2.61550	−2.05280	0.28750
C	−1.41480	−1.88310	1.22510
C	−3.37960	−0.78480	−0.08850
Cl	−0.42320	−0.43260	0.88140
C	−4.63950	−1.06400	−0.92230
C	−0.83010	−4.69920	−2.70380
Br	−2.01490	−5.92240	−0.39550
C	−0.37270	−5.98310	−3.35670
C	0.69660	−6.05880	−4.16410
C	1.17010	−7.30190	−4.83610
C	0.41800	−8.59200	−4.64040
O	2.17750	−7.24150	−5.54180
C	−5.73240	−1.83760	−0.23940
O	−5.19970	0.18550	−1.33390
C	−6.07080	−1.75110	1.01670
C	−6.41230	−1.65880	2.26270
H	−6.23570	−2.42480	3.00610
Br	−7.02710	−0.02870	2.86430
H	−0.10150	−2.63780	−1.09920
H	0.29800	−5.11180	−0.87860
H	−1.05950	−3.97290	1.46640
H	0.43610	−3.08570	1.18320
H	−3.30190	−2.76560	0.76160
H	−1.71560	−1.82010	2.27450
H	−3.64030	−0.21510	0.81240
H	−2.74140	−0.12590	−0.69120
H	−4.35700	−1.59590	−1.83770
H	−1.87480	−4.52240	−2.98970
H	−0.25350	−3.86350	−3.12150
H	−0.97920	−6.86220	−3.15260
H	1.30200	−5.18360	−4.38270
H	−0.60780	−8.48910	−5.00350
H	0.90620	−9.38460	−5.21570
H	0.42910	−8.87800	−3.58550
H	−6.40980	−2.36350	−0.90820
H	−5.56320	0.61090	−0.53690

Table S6. *Cont.*

**Marilzafurollene_A_36**			
C	−0.83320	−3.28290	−0.59440
O	−2.12440	−2.71510	−0.89830
C	−0.67200	−4.69730	−1.17160
C	−0.62340	−3.13670	0.91120
C	−2.61560	−2.05280	0.28750
C	−1.41500	−1.88340	1.22520
C	−3.37860	−0.78430	−0.08910
Cl	−0.42290	−0.43310	0.88200
C	−4.64100	−1.06290	−0.91940
C	−0.83100	−4.70180	−2.70220
Br	−2.01240	−5.92510	−0.39830
C	−0.38970	−5.99330	−3.34990
C	−1.20610	−6.75970	−4.09000
C	−0.81120	−8.03470	−4.75190
C	0.59290	−8.55590	−4.59300
O	−1.65150	−8.63650	−5.42140
C	−5.73590	−1.83020	−0.23250
O	−5.19820	0.18730	−1.33360
C	−6.05830	−1.75420	1.02840
C	−6.38220	−1.67140	2.27980
H	−6.21030	−2.45070	3.01050
Br	−6.92370	−0.02850	2.91670
H	−0.10180	−2.63800	−1.09980
H	0.29890	−5.11140	−0.87710
H	−1.06020	−3.97330	1.46590
H	0.43570	−3.08630	1.18350
H	−3.30220	−2.76540	0.76130
H	−1.71600	−1.82070	2.27460
H	−3.63630	−0.21250	0.81130
H	−2.74060	−0.12770	−0.69450
H	−4.36210	−1.59850	−1.83370
H	−1.86570	−4.46530	−2.98240
H	−0.20630	−3.90660	−3.12910
H	0.65010	−6.26980	−3.19480
H	−2.24400	−6.48190	−4.25180
H	0.81050	−8.73740	−3.53730
H	0.68790	−9.50510	−5.12920
H	1.30760	−7.84790	−5.02010
H	−6.42910	−2.33740	−0.89960
H	−5.55550	0.61750	−0.53640

Table S6. *Cont.*

**Marilzafurollene_A_37**			
C	−0.83790	−3.27920	−0.59100
O	−2.12350	−2.71750	−0.90390
C	−0.37370	−4.31550	−1.62540
C	−0.95920	−3.69150	0.86660
C	−2.61160	−2.05410	0.28970
C	−1.69090	−2.49260	1.45190
C	−2.70430	−0.55210	−0.00110
Cl	−0.50010	−1.26250	1.98230
C	−3.72310	−0.21250	−1.09750
C	−0.51440	−3.85000	−3.09140
Br	−1.36780	−6.00930	−1.46350
C	−0.00660	−2.44980	−3.34340
C	−0.85750	−1.42710	−3.52390
C	−0.48020	0.00600	−3.60900
C	0.93080	0.38670	−3.96570
O	−1.34160	0.84700	−3.34120
C	−5.13730	−0.28000	−0.60510
O	−3.50700	1.13450	−1.52080
C	−5.99740	−1.20760	−0.91560
C	−6.85360	−2.12770	−1.22710
H	−7.48230	−2.10860	−2.10720
Br	−7.02820	−3.56630	−0.08960
H	−0.12060	−2.44830	−0.64480
H	0.66560	−4.59410	−1.41700
H	−1.60330	−4.56850	0.98740
H	0.00620	−3.90980	1.33260
H	−3.61070	−2.46480	0.47260
H	−2.26960	−2.75490	2.34230
H	−2.95000	0.00120	0.91420
H	−1.72730	−0.18250	−0.33950
H	−3.59770	−0.85530	−1.97510
H	0.03280	−4.53780	−3.74900
H	−1.56500	−3.91840	−3.40630
H	1.06840	−2.29770	−3.30130
H	−1.93100	−1.59630	−3.51690
H	1.30680	−0.24600	−4.77410
H	0.94440	1.42170	−4.32030
H	1.57090	0.29950	−3.08450
H	−5.45570	0.51630	0.06200
H	−2.73850	1.11530	−2.13410

Table S6. *Cont.*

**Marilzafurollene_A_38**			
C	−0.83340	−3.28250	−0.59350
O	−2.12520	−2.71490	−0.89890
C	−0.67950	−4.70460	−1.15500
C	−0.61490	−3.11930	0.90970
C	−2.61460	−2.05340	0.28730
C	−1.41010	−1.86590	1.21610
C	−3.40770	−0.80270	−0.08220
Cl	−0.42520	−0.41660	0.84980
C	−4.61090	−1.08230	−0.99260
C	−0.83800	−4.72410	−2.68590
Br	−2.03040	−5.91070	−0.36290
C	−0.35020	−6.00130	−3.32430
C	0.68460	−6.06260	−4.17730
C	1.10500	−7.36070	−4.78220
C	2.24860	−7.31910	−5.76160
O	0.56050	−8.42040	−4.47740
C	−5.62490	−2.01790	−0.39240
O	−5.22700	0.17220	−1.29360
C	−6.63000	−1.68360	0.36700
C	−7.62920	−1.34780	1.11920
H	−7.55670	−1.12400	2.17500
Br	−9.28810	−1.19430	0.33250
H	−0.10250	−2.64510	−1.10910
H	0.28910	−5.12040	−0.85530
H	−1.04370	−3.95230	1.47600
H	0.44570	−3.06150	1.17440
H	−3.28730	−2.77250	0.77250
H	−1.70440	−1.79340	2.26670
H	−3.73870	−0.28650	0.82820
H	−2.76750	−0.09170	−0.61940
H	−4.26730	−1.50080	−1.94540
H	−1.88660	−4.57380	−2.97250
H	−0.27960	−3.88070	−3.11270
H	−0.89630	−6.91210	−3.07960
H	1.24730	−5.18050	−4.46010
H	2.02630	−7.98480	−6.60040
H	2.39080	−6.31160	−6.16190
H	3.16480	−7.64260	−5.26130
H	−5.43990	−3.07790	−0.55110
H	−6.07610	−0.02570	−1.72650

Table S6. *Cont.*

**Marilzafurollene_A_39**			
C	−0.83310	−3.28280	−0.59380
O	−2.12510	−2.71480	−0.89850
C	−0.68270	−4.70630	−1.15270
C	−0.61930	−3.12550	0.91130
C	−2.61490	−2.05330	0.28720
C	−1.41350	−1.87240	1.22110
C	−3.40280	−0.79910	−0.08190
Cl	−0.42490	−0.42270	0.86850
C	−4.60170	−1.07130	−1.00010
C	−0.84540	−4.78760	−2.67980
Br	−2.03710	−5.91530	−0.36770
C	0.25690	−4.08590	−3.43330
C	0.03890	−3.03490	−4.23900
C	1.09240	−2.32120	−5.01430
C	2.52900	−2.75890	−4.90080
O	0.75850	−1.38170	−5.73670
C	−5.61690	−2.01470	−0.41440
O	−5.21860	0.18520	−1.29090
C	−6.62830	−1.69000	0.34070
C	−7.63380	−1.36410	1.08900
H	−7.56880	−1.14640	2.14650
Br	−9.29130	−1.22860	0.29600
H	−0.10130	−2.64030	−1.09960
H	0.27960	−5.13260	−0.84730
H	−1.05100	−3.96010	1.47300
H	0.44060	−3.07060	1.17940
H	−3.29190	−2.77060	0.76900
H	−1.71190	−1.80570	2.27090
H	−3.73720	−0.28550	0.82880
H	−2.75780	−0.08770	−0.61290
H	−4.25290	−1.47960	−1.95560
H	−0.83180	−5.83840	−2.99850
H	−1.82700	−4.39720	−2.97900
H	1.25510	−4.49720	−3.30450
H	−0.96010	−2.62900	−4.37540
H	2.63970	−3.78390	−5.26380
H	3.15440	−2.10660	−5.51810
H	2.86800	−2.67550	−3.86500
H	−5.42660	−3.07280	−0.57930
H	−6.06250	−0.00950	−1.73510

Table S6. *Cont.*

**Marilzafurollene_A_40**			
C	−0.83300	−3.28280	−0.59400
O	−2.12500	−2.71470	−0.89820
C	−0.68480	−4.70680	−1.15230
C	−0.61840	−3.12410	0.91120
C	−2.61510	−2.05320	0.28710
C	−1.41400	−1.87190	1.22120
C	−3.40340	−0.79960	−0.08310
Cl	−0.42750	−0.42040	0.87030
C	−4.59890	−1.07260	−1.00540
C	−0.85060	−4.80250	−2.67910
Br	−2.03960	−5.91240	−0.35690
C	0.24020	−4.10160	−3.44510
C	1.16460	−4.73820	−4.18120
C	2.20810	−3.97410	−4.92570
C	3.15230	−4.76870	−5.78910
O	2.30900	−2.75300	−4.81930
C	−5.61580	−2.01590	−0.42250
O	−5.21530	0.18370	−1.29890
C	−6.63150	−1.69060	0.32660
C	−7.64100	−1.36400	1.06910
H	−7.58130	−1.14300	2.12630
Br	−9.29510	−1.23420	0.26810
H	−0.10100	−2.64050	−1.09870
H	0.27710	−5.13390	−0.84650
H	−1.04830	−3.95870	1.47430
H	0.44170	−3.06770	1.17850
H	−3.29210	−2.77060	0.76880
H	−1.71280	−1.80610	2.27100
H	−3.74110	−0.28720	0.82710
H	−2.75770	−0.08690	−0.61140
H	−4.24690	−1.48110	−1.95950
H	−0.87780	−5.85860	−2.97990
H	−1.81550	−4.38100	−2.98880
H	0.24280	−3.01240	−3.40640
H	1.18700	−5.81870	−4.26490
H	4.10700	−4.88320	−5.26990
H	2.74380	−5.75700	−6.01720
H	3.30240	−4.24110	−6.73520
H	−5.42310	−3.07410	−0.58360
H	−6.05670	−0.01190	−1.74750

Table S6. *Cont.*

**Marilzafurollene_A_41**			
C	−0.83230	−3.28320	−0.59400
O	−2.12520	−2.71490	−0.89910
C	−0.68590	−4.71370	−1.13710
C	−0.60850	−3.10660	0.90750
C	−2.61560	−2.05260	0.28790
C	−1.40690	−1.85450	1.20820
C	−3.39920	−0.79940	−0.09540
Cl	−0.43300	−0.40150	0.82360
C	−4.67940	−1.10970	−0.88720
C	−0.86820	−4.81400	−2.66050
Br	−2.02440	−5.91850	−0.32030
C	0.21650	−4.10900	−3.43010
C	−0.00630	−3.06690	−4.24610
C	1.11830	−2.41670	−4.97930
C	0.76750	−1.27620	−5.89810
O	2.28300	−2.77920	−4.82120
C	−5.78810	−1.79460	−0.14050
O	−5.23150	0.11860	−1.36890
C	−5.94130	−1.87170	1.15180
C	−6.10320	−1.95560	2.43420
H	−5.93420	−2.85190	3.01580
Br	−6.47980	−0.40910	3.36200
H	−0.10270	−2.64790	−1.11170
H	0.28180	−5.13300	−0.83890
H	−1.03090	−3.93660	1.48280
H	0.45310	−3.04220	1.16700
H	−3.28440	−2.77340	0.77370
H	−1.69590	−1.77450	2.25970
H	−3.64160	−0.20840	0.79670
H	−2.77710	−0.15390	−0.72870
H	−4.43470	−1.71530	−1.76770
H	−0.84860	−5.86780	−2.96830
H	−1.85630	−4.43470	−2.95200
H	1.22870	−4.49440	−3.31010
H	−1.00010	−2.66040	−4.39560
H	1.00960	−0.33000	−5.40790
H	1.33970	−1.37400	−6.82500
H	−0.29480	−1.28780	−6.15670
H	−6.61890	−2.12210	−0.76070
H	−4.55830	0.52830	−1.93900

Table S6. *Cont.*

**Marilzafurollene_A_42**			
C	−0.83320	−3.28290	−0.59430
O	−2.12450	−2.71510	−0.89830
C	−0.67230	−4.69810	−1.17060
C	−0.62100	−3.13330	0.91080
C	−2.61550	−2.05290	0.28750
C	−1.41390	−1.88040	1.22340
C	−3.38110	−0.78600	−0.08890
Cl	−0.42400	−0.42950	0.87610
C	−4.64330	−1.06690	−0.91880
C	−0.83660	−4.70350	−2.70110
Br	−2.00980	−5.92290	−0.38540
C	−0.34470	−5.97200	−3.35350
C	0.68490	−6.02000	−4.21360
C	1.10880	−7.31120	−4.83070
C	2.24050	−7.25450	−5.82300
O	0.57660	−8.37700	−4.52540
C	−5.73650	−1.83630	−0.23160
O	−5.20300	0.18230	−1.33280
C	−6.06040	−1.75910	1.02880
C	−6.38620	−1.67560	2.27970
H	−6.21240	−2.45310	3.01180
Br	−6.93880	−0.03490	2.91240
H	−0.10200	−2.63850	−1.10090
H	0.30040	−5.11000	−0.87890
H	−1.05540	−3.96920	1.46850
H	0.43850	−3.08060	1.18090
H	−3.30030	−2.76630	0.76270
H	−1.71360	−1.81610	2.27300
H	−3.63960	−0.21460	0.81150
H	−2.74460	−0.12810	−0.69460
H	−4.36360	−1.60190	−1.83330
H	−1.88700	−4.55610	−2.98210
H	−0.28420	−3.85300	−3.12170
H	−0.88350	−6.88820	−3.11290
H	1.24020	−5.13230	−4.49340
H	2.01720	−7.92240	−6.65980
H	2.36480	−6.24500	−6.22420
H	3.16640	−7.56660	−5.33360
H	−6.42730	−2.34690	−0.89850
H	−5.56140	0.61140	−0.53550

Table S6. *Cont.*

Marilzafurollene_A_43			
C	−0.83280	−3.28310	−0.59460
O	−2.12450	−2.71500	−0.89800
C	−0.67680	−4.70170	−1.16420
C	−0.62400	−3.13650	0.91180
C	−2.61590	−2.05270	0.28740
C	−1.41640	−1.88400	1.22640
C	−3.37880	−0.78400	−0.08840
Cl	−0.42460	−0.43260	0.88800
C	−4.63990	−1.06160	−0.92100
C	−0.84060	−4.77270	−2.69170
Br	−2.02200	−5.92460	−0.38590
C	0.26050	−4.06470	−3.44130
C	0.04020	−3.01170	−4.24370
C	1.09230	−2.29230	−5.01550
C	2.53010	−2.72630	−4.90190
O	0.75690	−1.35120	−5.73510
C	−5.73510	−1.83180	−0.23790
O	−5.19830	0.18880	−1.33280
C	−6.05920	−1.76020	1.02290
C	−6.38490	−1.68220	2.27410
H	−6.21350	−2.46390	3.00220
Br	−6.93240	−0.04310	2.91540
H	−0.10080	−2.63560	−1.09360
H	0.28820	−5.12520	−0.86310
H	−1.06010	−3.97320	1.46680
H	0.43500	−3.08550	1.18430
H	−3.30290	−2.76530	0.76050
H	−1.71890	−1.82330	2.27540
H	−3.63800	−0.21360	0.81250
H	−2.74010	−0.12620	−0.69190
H	−4.35910	−1.59450	−1.83640
H	−0.82560	−5.82130	−3.01770
H	−1.82290	−4.38180	−2.98750
H	1.25980	−4.47350	−3.31290
H	−0.96000	−2.60830	−4.37940
H	2.64370	−3.75080	−5.26560
H	3.15390	−2.07200	−5.51860
H	2.86830	−2.64270	−3.86590
H	−6.42710	−2.33690	−0.90760
H	−5.55160	0.61930	−0.53400

Table S6. *Cont.*

**Marilzafurollene_A_44**			
C	−0.83280	−3.28320	−0.59470
O	−2.12440	−2.71490	−0.89770
C	−0.67720	−4.69990	−1.16900
C	−0.62530	−3.13910	0.91240
C	−2.61600	−2.05270	0.28730
C	−1.41790	−1.88700	1.22860
C	−3.37620	−0.78270	−0.08970
Cl	−0.42610	−0.43440	0.89690
C	−4.63440	−1.05840	−0.92720
C	−0.84580	−4.77940	−2.69640
Br	−2.02010	−5.92470	−0.38400
C	0.23960	−4.06390	−3.45670
C	1.16420	−4.68700	−4.20420
C	2.20200	−3.90920	−4.94210
C	3.14270	−4.68820	−5.82340
O	2.30160	−2.68980	−4.81670
C	−5.73080	−1.83060	−0.24820
O	−5.19190	0.19280	−1.33800
C	−6.06930	−1.74840	1.00810
C	−6.40990	−1.66020	2.25460
H	−6.23680	−2.43080	2.99410
Br	−7.00830	−0.02760	2.86610
H	−0.10040	−2.63350	−1.08960
H	0.28840	−5.12360	−0.87020
H	−1.06190	−3.97650	1.46590
H	0.43340	−3.08860	1.18590
H	−3.30530	−2.76420	0.75890
H	−1.72240	−1.82920	2.27730
H	−3.63810	−0.21320	0.81100
H	−2.73480	−0.12470	−0.69010
H	−4.35070	−1.58930	−1.84280
H	−0.86770	−5.83220	−3.00900
H	−1.81360	−4.35990	−2.99930
H	0.23770	−2.97540	−3.40370
H	1.19030	−5.76620	−4.30240
H	3.28590	−4.14560	−6.76210
H	4.10080	−4.80840	−5.31170
H	2.73520	−5.67370	−6.06430
H	−6.41010	−2.35030	−0.91980
H	−5.55050	0.62000	−0.53980

Table S6. *Cont.*

	**Marilzafurollene_A_45**		
C	−0.83280	−3.28300	−0.59400
O	−2.12480	−2.71520	−0.89920
C	−0.67460	−4.70340	−1.15690
C	−0.61600	−3.12170	0.90930
C	−2.61550	−2.05270	0.28800
C	−1.40950	−1.86760	1.21540
C	−3.38660	−0.79150	−0.09520
Cl	−0.42500	−0.41810	0.84470
C	−4.66580	−1.08830	−0.89390
C	−0.83810	−4.72380	−2.68640
Br	−2.01710	−5.92090	−0.36870
C	−0.38200	−6.01460	−3.31780
C	−1.18640	−6.82860	−4.01900
C	−0.66340	−8.09090	−4.61750
C	−1.65210	−8.96400	−5.34460
O	0.52590	−8.39520	−4.54070
C	−5.78550	−1.76190	−0.15310
O	−5.20210	0.14600	−1.37770
C	−5.94450	−1.84080	1.13830
C	−6.11240	−1.92610	2.41980
H	−5.95600	−2.82590	2.99970
Br	−6.47540	−0.37790	3.35020
H	−0.10250	−2.64430	−1.10890
H	0.29610	−5.11610	−0.85950
H	−1.04720	−3.95450	1.47410
H	0.44430	−3.06540	1.17560
H	−3.29260	−2.76970	0.76770
H	−1.70270	−1.79300	2.26610
H	−3.62790	−0.20090	0.79750
H	−2.75610	−0.14970	−0.72380
H	−4.42300	−1.69630	−1.77320
H	−1.87660	−4.50520	−2.96690
H	−0.22580	−3.92510	−3.12430
H	0.66890	−6.27350	−3.19210
H	−2.23660	−6.60500	−4.16720
H	−1.53140	−8.82640	−6.42190
H	−10.00910	−5.07870	
H	−2.67970	−8.71990	−5.06200
H	−6.61760	−2.07780	−0.77750
H	−4.52580	0.54190	−1.95420

Table S6. *Cont.*

Marilzafurollene_A_46			
C	−0.83330	−3.28270	−0.59380
O	−2.12490	−2.71490	−0.89850
C	−0.68410	−4.70540	−1.15430
C	−0.61500	−3.11880	0.91020
C	−2.61490	−2.05330	0.28720
C	−1.41160	−1.86640	1.21770
C	−3.40690	−0.80220	−0.08380
Cl	−0.42780	−0.41530	0.85630
C	−4.60750	−1.08110	−0.99770
C	−0.88350	−4.80560	−2.67700
Br	−2.00870	−5.92440	−0.33000
C	0.14260	−4.04270	−3.47740
C	1.11120	−4.63870	−4.18960
C	2.12700	−3.92430	−5.01360
C	2.08520	−2.42240	−5.11680
O	2.98550	−4.58870	−5.59480
C	−5.62350	−2.01650	−0.40070
O	−5.22190	0.17380	−1.30070
C	−6.63360	−1.68170	0.35190
C	−7.63790	−1.34540	1.09700
H	−7.57250	−1.11960	2.15280
Br	−9.29150	−1.19400	0.29880
H	−0.10230	−2.64110	−1.10150
H	0.28880	−5.12190	−0.86980
H	−1.04220	−3.95130	1.47810
H	0.44570	−3.05970	1.17440
H	−3.28870	−2.77190	0.77160
H	−1.70740	−1.79630	2.26800
H	−3.74020	−0.28630	0.82600
H	−2.76520	−0.09110	−0.61900
H	−4.26130	−1.49970	−1.94960
H	−0.86300	−5.86180	−2.97870
H	−1.87850	−4.43840	−2.96000
H	0.03610	−2.96040	−3.47380
H	1.21330	−5.72040	−4.20750
H	2.23870	−1.97320	−4.13220
H	2.88890	−2.08390	−5.77790
H	1.13260	−2.10030	−5.54550
H	−5.43690	−3.07660	−0.55640
H	−6.07010	−0.02340	−1.73560

Table S6. *Cont.*

**Marilzafurollene_A_47**			
C	−0.83270	−3.28390	−0.59650
O	−2.12250	−2.71520	−0.89580
C	−0.62320	−4.67330	−1.21680
C	−0.66870	−3.20680	0.92000
C	−2.61790	−2.05170	0.28710
C	−1.43960	−1.94780	1.26180
C	−3.31000	−0.74760	−0.10450
Cl	−0.40350	−0.50970	1.00540
C	−4.55780	−0.96850	−0.96920
C	−0.55500	−4.68110	−2.75680
Br	−1.99000	−5.95520	−0.61540
C	−1.76440	−4.15110	−3.48570
C	−1.75810	−3.01660	−4.20220
C	−2.94710	−2.44790	−4.89480
C	−4.24710	−3.20850	−4.89800
O	−2.83820	−1.35350	−5.44910
C	−5.70350	−1.55730	−0.20420
O	−5.01020	0.30100	−1.44440
C	−6.12930	−2.78360	−0.31200
C	−6.55350	−4.00270	−0.41310
H	−7.25930	−4.34860	−1.15620
Br	−5.95580	−5.22870	0.82530
H	−0.09570	−2.60920	−1.05260
H	0.31500	−5.09370	−0.83540
H	−1.14740	−4.05610	1.41930
H	0.38120	−3.19530	1.22910
H	−3.34920	−2.73810	0.73100
H	−1.76790	−1.91830	2.30440
H	−3.57410	−0.17250	0.79220
H	−2.62210	−0.11940	−0.68480
H	−4.32460	−1.58120	−1.84760
H	0.33300	−4.11000	−3.05900
H	−0.38020	−5.70620	−3.10900
H	−2.67190	−4.74120	−3.38610
H	−0.86080	−2.41190	−4.29410
H	−4.98150	−2.66410	−5.49940
H	−4.10620	−4.19560	−5.34580
H	−4.63390	−3.29670	−3.87950
H	−6.20770	−0.89800	0.49720
H	−4.35810	0.59870	−2.10360

Table S6. *Cont.*

**Marilzafurollene_A_48**			
C	−0.83310	−3.28300	−0.59450
O	−2.12430	−2.71510	−0.89800
C	−0.67650	−4.69880	−1.17000
C	−0.62120	−3.13290	0.91130
C	−2.61580	−2.05270	0.28740
C	−1.41520	−1.88080	1.22480
C	−3.38060	−0.78560	−0.09010
Cl	−0.42650	−0.42830	0.88200
C	−4.64080	−1.06620	−0.92310
C	−0.88130	−4.78540	−2.69290
Br	−1.98580	−5.93780	−0.35250
C	0.13150	−4.00120	−3.48940
C	1.10650	−4.57700	−4.20940
C	2.10970	−3.84070	−5.02950
C	2.04590	−2.33870	−5.11940
O	2.97630	−4.48750	−5.61870
C	−5.73580	−1.83570	−0.23900
O	−5.19900	0.18310	−1.33850
C	−6.06100	−1.76110	1.02130
C	−6.38770	−1.68000	2.27210
H	−6.21530	−2.45920	3.00270
Br	−6.93930	−0.04000	2.90780
H	−0.10190	−2.63440	−1.09300
H	0.30110	−5.11030	−0.89390
H	−1.05460	−3.96800	1.47060
H	0.43830	−3.07940	1.18120
H	−3.30130	−2.76590	0.76190
H	−1.71640	−1.81850	2.27410
H	−3.64100	−0.21460	0.81000
H	−2.74280	−0.12750	−0.69410
H	−4.35890	−1.60110	−1.83700
H	−0.84910	−5.83800	−3.00570
H	−1.88210	−4.42760	−2.96760
H	0.00910	−2.92060	−3.47590
H	1.22400	−5.65690	−4.23700
H	1.08850	−2.02680	−5.54470
H	2.19350	−1.89610	−4.13090
H	2.84420	−1.98260	−5.77770
H	−6.42660	−2.34380	−0.90780
H	−5.55990	0.61210	−0.54220

Table S6. *Cont.*

**Marilzafurollene_A_49**			
C	−0.83320	−3.28290	−0.59460
O	−2.12390	−2.71530	−0.89830
C	−0.65540	−4.68580	−1.19880
C	−0.63720	−3.16160	0.91480
C	−2.61600	−2.05250	0.28770
C	−1.42160	−1.90580	1.23780
C	−3.35480	−0.77070	−0.09190
Cl	−0.41250	−0.46000	0.92380
C	−4.61940	−1.02940	−0.91950
C	−0.81710	−4.65950	−2.73060
Br	−1.98790	−5.93640	−0.44930
C	−0.37530	−5.86180	−3.53400
C	0.24470	−6.96900	−3.09610
C	0.60640	−8.06530	−4.04460
C	1.23240	−9.30060	−3.45180
O	0.42750	−7.95590	−5.25650
C	−5.71310	−1.68490	−0.13050
O	−5.12530	0.23530	−1.35220
C	−6.18940	−2.87600	−0.35570
C	−6.66370	−4.06120	−0.57290
H	−7.43180	−4.29280	−1.29860
Br	−6.10260	−5.42810	0.52780
H	−0.10100	−2.62470	−1.08150
H	0.32130	−5.08530	−0.90660
H	−1.08810	−4.00160	1.45270
H	0.41930	−3.12410	1.19870
H	−3.31860	−2.75550	0.75270
H	−1.73110	−1.85400	2.28530
H	−3.60960	−0.19850	0.80950
H	−2.70280	−0.12530	−0.69440
H	−4.37290	−1.60710	−1.81800
H	−1.86680	−4.46350	−2.98690
H	−0.24420	−3.80960	−3.12490
H	−0.60050	−5.78320	−4.59920
H	0.50480	−7.12650	−2.05740
H	0.98470	−9.39820	−2.39130
H	0.84380	−10.18270	−3.96850
H	2.31760	−9.24830	−3.56900
H	−6.13670	−1.10780	0.68730
H	−5.87590	0.04460	−1.94230

Table S6. *Cont.*

**Marilzafurollene_A_50**			
C	−0.83250	−3.28350	−0.59500
O	−2.12420	−2.71500	−0.89780
C	−0.66170	−4.68760	−1.19520
C	−0.63770	−3.16250	0.91490
C	−2.61650	−2.05230	0.28760
C	−1.42330	−1.90800	1.23910
C	−3.34920	−0.76740	−0.09740
Cl	−0.41910	−0.45770	0.93080
C	−4.63560	−1.02580	−0.89480
C	−0.83660	−4.72820	−2.72240
Br	−1.98220	−5.94610	−0.43550
C	0.22880	−3.96150	−3.45920
C	−0.02220	−2.88020	−4.21370
C	1.08080	−2.16450	−4.91730
C	0.69840	−0.97090	−5.75230
O	2.25330	−2.51730	−4.80010
C	−5.74110	−1.60510	−0.06140
O	−5.11360	0.21490	−1.42210
C	−6.15480	−2.83900	−0.12210
C	−6.56420	−4.06620	−0.17610
H	−7.29920	−4.43940	−0.87650
Br	−5.91320	−5.24600	1.08060
H	−0.10010	−2.62160	−1.07430
H	0.31160	−5.10300	−0.91020
H	−1.08790	−4.00290	1.45270
H	0.41860	−3.12400	1.19940
H	−3.32110	−2.75280	0.75200
H	−1.73470	−1.85920	2.28620
H	−3.58080	−0.18010	0.80050
H	−2.70140	−0.13970	−0.72270
H	−4.42570	−1.66860	−1.75710
H	−0.78810	−5.76730	−3.07420
H	−1.83430	−4.36450	−3.00110
H	1.24940	−4.33210	−3.36750
H	−1.02570	−2.48590	−4.32950
H	1.26650	−0.99040	−6.68650
H	−0.36500	−0.98930	−6.00590
H	0.92230	−0.05590	−5.19830
H	−6.22100	−0.94570	0.65580
H	−5.16310	0.85130	−0.68870

Table S6. *Cont.*

**Marilzafurollene_A_51**			
C	−0.83310	−3.28300	−0.59440
O	−2.12420	−2.71530	−0.89860
C	−0.66450	−4.69620	−1.17720
C	−0.62490	−3.13870	0.91160
C	−2.61590	−2.05250	0.28780
C	−1.41470	−1.88380	1.22490
C	−3.37080	−0.78290	−0.10010
Cl	−0.42120	−0.43460	0.87780
C	−4.64810	−1.06710	−0.90130
C	−0.83070	−4.69300	−2.70870
Br	−2.00240	−5.92700	−0.40460
C	−0.39850	−5.91020	−3.49440
C	0.21680	−7.01400	−3.04130
C	0.56920	−8.12690	−3.97410
C	1.19200	−9.35580	−3.36500
O	0.38510	−8.03560	−5.18660
C	−5.73850	−1.67070	−0.06930
O	−5.16380	0.17450	−1.38700
C	−6.12300	−2.91420	−0.12060
C	−6.50710	−4.14990	−0.16810
H	−7.21320	−4.54610	−0.88540
Br	−5.91990	−5.28570	1.15850
H	−0.10210	−2.63490	−1.09670
H	0.31080	−5.09660	−0.88170
H	−1.06430	−3.97470	1.46500
H	0.43390	−3.09000	1.18530
H	−3.30520	−2.76020	0.76430
H	−1.71460	−1.81900	2.27440
H	−3.61600	−0.19750	0.79540
H	−2.73150	−0.14410	−0.72290
H	−4.43400	−1.69520	−1.77400
H	−1.88000	−4.49450	−2.96510
H	−0.25390	−3.85290	−3.11790
H	−0.62690	−5.84730	−4.56010
H	0.47970	−7.15660	−2.00120
H	0.95900	−9.42980	−2.29930
H	0.78790	−10.24440	−3.85830
H	2.27580	−9.31610	−3.49840
H	−6.24690	−1.00390	0.62180
H	−4.53090	0.49700	−2.05240

Table S6. *Cont.*

**Marilzafurollene_A_52**			
C	−0.83290	−3.28360	−0.59610
O	−2.12290	−2.71510	−0.89610
C	−0.62060	−4.67750	−1.20500
C	−0.66430	−3.20230	0.92040
C	−2.61730	−2.05210	0.28700
C	−1.43940	−1.94780	1.26360
C	−3.31430	−0.74420	−0.08270
Cl	−0.40230	−0.50880	1.01600
C	−4.53940	−0.93510	−0.98360
C	−0.57470	−4.70960	−2.74460
Br	−1.95920	−5.96970	−0.56230
C	−1.80070	−4.21370	−3.46930
C	−1.79670	−3.15440	−4.29290
C	−2.99570	−2.63610	−5.00800
C	−4.32900	−3.31050	−4.82070
O	−2.86580	−1.65870	−5.74620
C	−5.66190	−1.67020	−0.31520
O	−5.03760	0.36230	−1.31660
C	−6.27120	−2.71120	−0.80670
C	−6.88130	−3.74270	−1.29750
H	−7.79140	−3.69610	−1.88070
Br	−6.03310	−5.37480	−1.19370
H	−0.09720	−2.61120	−1.05740
H	0.32800	−5.08280	−0.83250
H	−1.13680	−4.05140	1.42520
H	0.38680	−3.18630	1.22540
H	−3.34910	−2.74110	0.72800
H	−1.76950	−1.92220	2.30580
H	−3.60080	−0.19990	0.82670
H	−2.62220	−0.08920	−0.62730
H	−4.24320	−1.42370	−1.91900
H	0.30120	−4.13180	−3.06850
H	−0.39050	−5.73910	−3.07900
H	−2.71630	−4.77040	−3.28610
H	−0.88940	−2.58850	−4.48170
H	−4.28210	−4.34370	−5.17430
H	−4.62480	−3.27350	−3.76970
H	−5.08660	−2.78110	−5.40660
H	−6.00680	−1.27430	0.63620
H	−5.76150	0.22570	−1.95300

Table S6. *Cont.*

**Marilzafurollene_A_53**			
C	−0.83260	−3.28360	−0.59570
O	−2.12330	−2.71520	−0.89700
C	−0.63960	−4.69790	−1.16280
C	−0.64150	−3.15760	0.91580
C	−2.61720	−2.05190	0.28750
C	−1.42390	−1.90130	1.23690
C	−3.35910	−0.77140	−0.09020
Cl	−0.41400	−0.45730	0.91670
C	−4.62080	−1.02950	−0.92160
C	−0.57210	−4.78190	−2.69980
Br	−2.01510	−5.94190	−0.50640
C	−1.78440	−4.31620	−3.46700
C	−2.52750	−5.12750	−4.23570
C	−3.72660	−4.70160	−5.01000
C	−4.19780	−3.27360	−4.93350
O	−4.31020	−5.53690	−5.70150
C	−5.71030	−1.70750	−0.14670
O	−5.13580	0.23610	−1.33960
C	−6.19210	−2.88910	−0.40730
C	−6.67210	−4.06550	−0.65730
H	−7.44410	−4.27420	−1.38570
Br	−6.09070	−5.47060	0.38260
H	−0.09800	−2.63110	−1.08620
H	0.29640	−5.10860	−0.76520
H	−1.09640	−3.99860	1.44990
H	0.41430	−3.12240	1.20320
H	−3.31860	−2.75600	0.75330
H	−1.73300	−1.84630	2.28440
H	−3.61710	−0.20200	0.81190
H	−2.70760	−0.12270	−0.68960
H	−4.36680	−1.59180	−1.82730
H	0.27900	−4.17770	−3.04060
H	−0.33160	−5.81500	−2.98560
H	−2.02590	−3.26030	−3.37560
H	−2.28940	−6.18240	−4.33550
H	−4.47360	−3.02500	−3.90580
H	−3.42110	−2.59870	−5.30180
H	−5.08400	−3.15110	−5.56400
H	−6.13000	−1.15440	0.68970
H	−5.91680	0.05090	−1.89060

Table S6. *Cont.*

**Marilzafurollene_A_54**			
C	−0.83320	−3.28290	−0.59450
O	−2.12400	−2.71540	−0.89860
C	−0.65340	−4.68460	−1.20070
C	−0.63900	−3.16420	0.91520
C	−2.61600	−2.05250	0.28800
C	−1.42190	−1.90770	1.23870
C	−3.35170	−0.76910	−0.09250
Cl	−0.41160	−0.46250	0.92530
C	−4.62920	−1.02830	−0.90010
C	−0.81210	−4.65530	−2.73320
Br	−1.98970	−5.93570	−0.45980
C	−0.38490	−5.86280	−3.54140
C	0.28010	−6.94370	−3.10430
C	0.67060	−8.10270	−3.95840
C	0.26170	−8.13890	−5.40760
O	1.32900	−9.01130	−3.45070
C	−5.72100	−1.65140	−0.08250
O	−5.12420	0.23340	−1.35360
C	−6.20290	−2.84810	−0.26100
C	−6.68110	−4.03980	−0.42820
H	−7.46510	−4.29660	−1.12780
Br	−6.05160	−5.38080	0.66740
H	−0.10070	−2.62350	−1.07960
H	0.32220	−5.08370	−0.90550
H	−1.09190	−4.00450	1.45110
H	0.41710	−3.12850	1.20060
H	−3.31960	−2.75480	0.75220
H	−1.73180	−1.85600	2.28600
H	−3.59190	−0.18790	0.80730
H	−2.70320	−0.13310	−0.70870
H	−4.40200	−1.62850	−1.78880
H	−1.86060	−4.44990	−2.98780
H	−0.23290	−3.80980	−3.12780
H	−0.66690	−5.79260	−4.59090
H	0.59220	−7.05390	−2.07120
H	0.59210	−9.08300	−5.85150
H	−0.82660	−8.08580	−5.49470
H	0.73540	−7.31840	−5.95240
H	−6.12980	−1.04800	0.72390
H	−5.88630	0.04040	−1.92800

Table S6. *Cont.*

**Marilzafurollene_A_55**			
C	−0.83290	−3.28360	−0.59610
O	−2.12270	−2.71530	−0.89640
C	−0.61640	−4.67150	−1.21660
C	−0.67270	−3.21460	0.92150
C	−2.61750	−2.05190	0.28730
C	−1.44270	−1.95760	1.26830
C	−3.30450	−0.74000	−0.08740
Cl	−0.39930	−0.52160	1.03060
C	−4.54050	−0.93420	−0.97280
C	−0.54440	−4.68420	−2.75570
Br	−1.97390	−5.96280	−0.61330
C	−1.75590	−4.17260	−3.49370
C	−1.73740	−3.09380	−4.29150
C	−2.92320	−2.55990	−5.01710
C	−4.26020	−3.23750	−4.86790
O	−2.77970	−1.56840	−5.73340
C	−5.67620	−1.60490	−0.26080
O	−5.00620	0.35990	−1.36050
C	−6.17250	−2.76910	−0.56810
C	−6.66760	−3.92870	−0.86240
H	−7.42970	−4.10160	−1.61040
Br	−6.09420	−5.38230	0.11330
H	−0.09580	−2.60610	−1.04760
H	0.32380	−5.08610	−0.83330
H	−1.15370	−4.06530	1.41610
H	0.37650	−3.20640	1.23330
H	−3.35390	−2.73750	0.72510
H	−1.77630	−1.93540	2.30940
H	−3.57790	−0.18360	0.81850
H	−2.61090	−0.09860	−0.64620
H	−4.26860	−1.47190	−1.88840
H	0.33940	−4.10650	−3.05750
H	−0.35940	−5.71010	−3.10060
H	−2.67360	−4.73520	−3.34270
H	−0.82760	−2.52230	−4.44860
H	−4.21050	−4.25720	−5.25830
H	−4.56910	−3.23870	−3.81950
H	−5.01020	−2.68520	−5.44240
H	−6.10750	−1.06820	0.58030
H	−5.72500	0.21560	−2.00100

Table S6. *Cont.*

**Marilzafurollene_A_56**			
C	−0.83350	−3.28390	−0.59770
O	−2.12130	−2.71480	−0.89270
C	−0.66060	−4.70170	−1.16290
C	−0.62790	−3.13910	0.91030
C	−2.61830	−2.05220	0.28530
C	−1.42280	−1.88840	1.22950
C	−3.37750	−0.78530	−0.10240
Cl	−0.42430	−0.43820	0.90350
C	−4.56380	−1.05140	−1.03770
C	−0.70810	−4.80390	−2.70080
Br	−1.97260	−5.95880	−0.39960
C	−1.97200	−4.33670	−3.37480
C	−2.85980	−5.15580	−3.95890
C	−4.10810	−4.61990	−4.57480
C	−5.11100	−5.63090	−5.06480
O	−4.30390	−3.41220	−4.70090
C	−5.66050	−1.84980	−0.39930
O	−5.13010	0.21290	−1.39120
C	−6.19530	−2.92090	−0.91230
C	−6.73180	−3.97520	−1.43880
H	−7.48650	−3.95550	−2.21340
Br	−6.10120	−5.62420	−0.91510
H	−0.10190	−2.64030	−1.10420
H	0.30470	−5.09830	−0.82600
H	−1.06520	−3.97810	1.46120
H	0.43050	−3.08870	1.18540
H	−3.31490	−2.75910	0.75460
H	−1.72840	−1.83310	2.27790
H	−3.71600	−0.25710	0.79830
H	−2.71230	−0.09150	−0.63160
H	−4.21240	−1.52390	−1.96280
H	0.11960	−4.21250	−3.11340
H	−0.50070	−5.84200	−2.99420
H	−2.15850	−3.26310	−3.37040
H	−2.72060	−6.23040	−3.97440
H	−4.97630	−6.59420	−4.56520
H	−6.11970	−5.27460	−4.83700
H	−4.99670	−5.76090	−6.14370
H	−6.07880	−1.45600	0.52290
H	−5.77180	0.02990	−2.10080

Table S6. *Cont.*

**Marilzafurollene_A_57**			
C	−0.83290	−3.28290	−0.59410
O	−2.12460	−2.71530	−0.89920
C	−0.67280	−4.70060	−1.16480
C	−0.61910	−3.12740	0.91020
C	−2.61560	−2.05260	0.28810
C	−1.41080	−1.87270	1.21830
C	−3.38160	−0.78840	−0.09530
Cl	−0.42260	−0.42400	0.85370
C	−4.66220	−1.08060	−0.89340
C	−0.83360	−4.71110	−2.69560
Br	−2.01080	−5.92300	−0.37790
C	−0.37570	−5.99770	−3.34260
C	0.69200	−6.07640	−4.15170
C	1.16620	−7.32260	−4.81760
C	0.41700	−8.61290	−4.61250
O	2.17170	−7.26440	−5.52610
C	−5.78330	−1.75020	−0.15120
O	−5.19440	0.15530	−1.37740
C	−5.94820	−1.81530	1.14020
C	−6.12240	−1.88640	2.42180
H	−5.96820	−2.77930	3.01280
Br	−6.49990	−0.32950	3.33190
H	−0.10210	−2.64110	−1.10440
H	0.29940	−5.11220	−0.87100
H	−1.05340	−3.96090	1.47150
H	0.44070	−3.07400	1.17900
H	−3.29610	−2.76800	0.76540
H	−1.70590	−1.80040	2.26860
H	−3.62040	−0.19660	0.79710
H	−2.74870	−0.14930	−0.72440
H	−4.42200	−1.69000	−1.77250
H	−1.87920	−4.53740	−2.98000
H	−0.25920	−3.87680	−3.11920
H	−0.98030	−6.87670	−3.13220
H	1.29570	−5.20160	−4.37660
H	0.90650	−9.40850	−5.18260
H	0.42960	−8.89170	−3.55560
H	−0.60940	−8.51460	−4.97540
H	−6.61250	−2.07310	−0.77600
H	−4.51790	0.54750	−1.95600

Table S6. *Cont.*

**Marilzafurollene_A_58**			
C	−0.83300	−3.28290	−0.59430
O	−2.12430	−2.71530	−0.89880
C	−0.66360	−4.69570	−1.17770
C	−0.62490	−3.13890	0.91170
C	−2.61580	−2.05260	0.28790
C	−1.41460	−1.88400	1.22500
C	−3.37060	−0.78260	−0.09960
Cl	−0.42080	−0.43490	0.87730
C	−4.64740	−1.06610	−0.90180
C	−0.83530	−4.69250	−2.70910
Br	−1.99850	−5.92900	−0.40470
C	−0.41600	−5.91400	−3.50010
C	0.24960	−6.98910	−3.04960
C	0.63340	−8.16230	−3.88740
C	0.21820	−8.21980	−5.33410
O	1.29180	−9.06450	−3.36850
C	−5.73760	−1.67180	−0.07120
O	−5.16380	0.17590	−1.38570
C	−6.12310	−2.91490	−0.12620
C	−6.50840	−4.15010	−0.17680
H	−7.21590	−4.54360	−0.89420
Br	−5.92120	−5.29010	1.14620
H	−0.10230	−2.63460	−1.09650
H	0.31300	−5.09340	−0.88370
H	−1.06470	−3.97490	1.46500
H	0.43380	−3.09050	1.18560
H	−3.30530	−2.76020	0.76420
H	−1.71430	−1.81890	2.27450
H	−3.61620	−0.19790	0.79620
H	−2.73100	−0.14340	−0.72150
H	−4.43250	−1.69250	−1.77540
H	−1.88550	−4.48990	−2.95870
H	−0.25820	−3.85470	−3.12290
H	−0.70420	−5.86030	−4.54880
H	0.56740	−7.08350	−2.01670
H	−0.87050	−8.16750	−5.41720
H	0.68980	−7.40760	−5.89310
H	0.54660	−9.17040	−5.76540
H	−6.24500	−1.00710	0.62260
H	−4.52860	0.50250	−2.04680

Table S6. *Cont.*

**Marilzafurollene_A_59**			
C	−0.83310	−3.28310	−0.59480
O	−2.12390	−2.71520	−0.89780
C	−0.65820	−4.68450	−1.19800
C	−0.63640	−3.16200	0.91420
C	−2.61610	−2.05250	0.28750
C	−1.42220	−1.90780	1.23860
C	−3.35010	−0.76750	−0.09510
Cl	−0.41660	−0.45880	0.92800
C	−4.62870	−1.02230	−0.90600
C	−0.81720	−4.66180	−2.72790
Br	−1.98670	−5.94000	−0.45030
C	−0.34680	−5.92920	−3.39550
C	−1.14340	−6.73230	−4.11770
C	−0.60850	−7.97220	−4.75090
C	−1.59010	−8.83860	−5.49550
O	0.58460	−8.26470	−4.68680
C	−5.73190	−1.63630	−0.09470
O	−5.11980	0.22470	−1.40540
C	−6.14920	−2.86500	−0.21120
C	−6.56420	−4.08630	−0.32410
H	−7.29500	−4.42230	−1.04740
Br	−5.96950	−5.31530	0.91300
H	−0.10100	−2.62550	−1.08280
H	0.31650	−5.09440	−0.90990
H	−1.08570	−4.00310	1.45180
H	0.42030	−3.12330	1.19750
H	−3.32050	−2.75340	0.75210
H	−1.73260	−1.85820	2.28590
H	−3.59050	−0.18740	0.80520
H	−2.69980	−0.13270	−0.71040
H	−4.40470	−1.64100	−1.78220
H	−1.85710	−4.44550	−3.00500
H	−0.21190	−3.84460	−3.14060
H	0.70700	−6.17980	−3.27770
H	−2.19630	−6.51570	−4.25740
H	−1.40830	−9.88630	−5.24020
H	−2.62060	−8.59940	−5.21900
H	−1.46150	−8.68800	−6.57030
H	−6.21320	−1.00660	0.64780
H	−5.18200	0.84240	−0.65720

Table S6. *Cont.*

Marilzafurollene_A_60			
C	−0.83290	−3.28290	−0.59410
O	−2.12470	−2.71520	−0.89910
C	−0.67460	−4.70330	−1.15770
C	−0.61620	−3.12190	0.90930
C	−2.61560	−2.05260	0.28800
C	−1.40940	−1.86750	1.21520
C	−3.38650	−0.79140	−0.09520
Cl	−0.42430	−0.41850	0.84360
C	−4.66750	−1.08820	−0.89090
C	−0.83690	−4.72280	−2.68780
Br	−2.01330	−5.92250	−0.36910
C	−0.40140	−6.02200	−3.32380
C	−1.22110	−6.79130	−4.05690
C	−0.83240	−8.07430	−4.70690
C	0.56730	−8.60430	−4.53750
O	−1.67380	−8.67520	−5.37570
C	−5.78640	−1.75900	−0.14650
O	−5.20320	0.14590	−1.37580
C	−5.94430	−1.83310	1.14530
C	−6.11170	−1.91370	2.42720
H	−5.95640	−2.81160	3.01020
Br	−6.47400	−0.36200	3.35220
H	−0.10270	−2.64420	−1.10890
H	0.29690	−5.11510	−0.86170
H	−1.04770	−3.95450	1.47400
H	0.44410	−3.06590	1.17560
H	−3.29270	−2.76960	0.76780
H	−1.70230	−1.79240	2.26590
H	−3.62580	−0.19970	0.79730
H	−2.75670	−0.15070	−0.72570
H	−4.42720	−1.69820	−1.76950
H	−1.87150	−4.48580	−2.96790
H	−0.21070	−3.93380	−3.12390
H	0.63720	−6.30200	−3.16590
H	−2.25770	−6.51020	−4.22180
H	1.28850	−7.90760	−4.97230
H	0.78100	−8.77320	−3.47890
H	0.65600	−9.56150	−5.06030
H	−6.61960	−2.07630	−0.76880
H	−4.52930	0.53770	−1.95780

Table S6. *Cont.*

**Marilzafurollene_A_61**			
C	−0.83270	−3.28360	−0.59570
O	−2.12320	−2.71520	−0.89700
C	−0.64060	−4.70210	−1.15340
C	−0.63400	−3.14570	0.91380
C	−2.61720	−2.05190	0.28750
C	−1.42100	−1.89190	1.23180
C	−3.36300	−0.77500	−0.09600
Cl	−0.41900	−0.44490	0.89980
C	−4.60870	−1.03490	−0.95220
C	−0.60310	−4.79950	−2.69060
Br	−1.99230	−5.95160	−0.45880
C	−1.84800	−4.38370	−3.43440
C	−2.57630	−5.22790	−4.18150
C	−3.81120	−4.85570	−4.92610
C	−4.34370	−3.45020	−4.83700
O	−4.37360	−5.71670	−5.60370
C	−5.71240	−1.71230	−0.20010
O	−5.13640	0.22280	−1.38030
C	−6.08600	−2.94930	−0.36510
C	−6.46260	−4.17800	−0.52420
H	−7.14190	−4.51690	−1.29480
Br	−5.95530	−5.40530	0.75290
H	−0.10010	−2.63510	−1.09450
H	0.30640	−5.10110	−0.77000
H	−1.08040	−3.98530	1.45720
H	0.42310	−3.10320	1.19500
H	−3.31450	−2.75650	0.75750
H	−1.72600	−1.83280	2.28020
H	−3.64000	−0.21260	0.80500
H	−2.70490	−0.11880	−0.67950
H	−4.35000	−1.60170	−1.85370
H	0.22060	−4.17290	−3.05660
H	−0.33610	−5.82800	−2.96980
H	−2.12900	−3.33800	−3.34290
H	−2.29870	−6.27330	−4.28230
H	−3.60920	−2.74220	−5.22900
H	−5.25280	−3.36990	−5.44070
H	−4.59960	−3.21130	−3.80180
H	−6.24790	−1.10680	0.52610
H	−4.47180	0.62140	−1.96880

Table S6. *Cont.*

**Marilzafurollene_A_62**			
C	−0.83250	−3.28320	−0.59430
O	−2.12460	−2.71510	−0.89880
C	−0.67400	−4.70230	−1.16230
C	−0.62250	−3.13210	0.91160
C	−2.61590	−2.05250	0.28790
C	−1.41370	−1.87780	1.22220
C	−3.37710	−0.78520	−0.09480
Cl	−0.42240	−0.42870	0.86920
C	−4.65500	−1.07100	−0.89940
C	−0.84670	−4.77760	−2.68870
Br	−2.00770	−5.93070	−0.37430
C	0.23820	−4.05450	−3.44720
C	−0.00390	−3.00220	−4.24420
C	1.03020	−2.26490	−5.02310
C	2.47210	−2.69200	−4.94230
O	0.67580	−1.31360	−5.72000
C	−5.78260	−1.73810	−0.16480
O	−5.18030	0.16820	−1.38250
C	−5.94690	−1.82040	1.12570
C	−6.12070	−1.90920	2.40630
H	−5.97390	−2.81230	2.98340
Br	−6.47720	−0.36200	3.34090
H	−0.10140	−2.63610	−1.09520
H	0.29500	−5.12030	−0.86640
H	−1.05880	−3.96650	1.46970
H	0.43680	−3.08070	1.18290
H	−3.29980	−2.76660	0.76230
H	−1.71230	−1.81020	2.27190
H	−3.61820	−0.19550	0.79850
H	−2.73970	−0.14590	−0.71900
H	−4.41290	−1.67880	−1.77910
H	−0.81940	−5.82640	−3.01330
H	−1.83660	−4.40110	−2.97810
H	1.24410	−4.45000	−3.32870
H	−1.01060	−2.61200	−4.36830
H	3.07840	−2.03590	−5.57420
H	2.83330	−2.60570	−3.91420
H	2.58260	−3.71630	−5.30750
H	−6.61490	−2.04520	−0.79330
H	−4.50010	0.55870	−1.95810

Table S6. *Cont.*

**Marilzafurollene_A_63**			
C	−0.83240	−3.28300	−0.59350
O	−2.12580	−2.71460	−0.89900
C	−0.69660	−4.72300	−1.11390
C	−0.59890	−3.08810	0.90470
C	−2.61490	−2.05320	0.28740
C	−1.40340	−1.83850	1.19970
C	−3.42490	−0.81530	−0.08960
Cl	−0.44210	−0.38280	0.79730
C	−4.65920	−1.13240	−0.94630
C	−0.86490	−4.84480	−2.63730
Br	−2.05880	−5.89810	−0.29120
C	0.24290	−4.17500	−3.40570
C	0.05020	−3.15160	−4.25230
C	1.19690	−2.53750	−4.98230
C	0.87890	−1.41740	−5.93740
O	2.35330	−2.91160	−4.79310
C	−5.65980	−2.01840	−0.25950
O	−5.30330	0.09390	−1.29530
C	−6.53690	−1.64750	0.63090
C	−7.40670	−1.28090	1.51760
H	−7.17570	−1.07320	2.55340
Br	−9.18340	−1.25780	1.02970
H	−0.10330	−2.65800	−1.12420
H	0.26310	−5.14920	−0.79990
H	−1.01010	−3.91590	1.49120
H	0.46430	−3.01410	1.15520
H	−3.27290	−2.77720	0.78500
H	−1.68690	−1.75200	2.25220
H	−3.72650	−0.27190	0.81500
H	−2.80500	−0.12120	−0.67090
H	−4.35740	−1.61220	−1.88460
H	−0.86310	−5.90380	−2.92770
H	−1.84180	−4.45020	−2.94570
H	1.24710	−4.57200	−3.25860
H	−0.93460	−2.73490	−4.43040
H	1.11590	−0.46120	−5.46450
H	1.47370	−1.54210	−6.84660
H	−0.17650	−1.42810	−6.22290
H	−5.55900	−3.08260	−0.45630
H	−4.67550	0.59950	−1.83910

Table S6. *Cont.*

Marilzafurollene_A_64			
C	−0.83260	−3.28320	−0.59450
O	−2.12450	−2.71510	−0.89850
C	−0.67670	−4.70240	−1.16290
C	−0.62170	−3.13100	0.91150
C	−2.61600	−2.05250	0.28780
C	−1.41420	−1.87740	1.22240
C	−3.37730	−0.78590	−0.09670
Cl	−0.42500	−0.42660	0.87150
C	−4.65230	−1.07300	−0.90550
C	−0.85350	−4.79010	−2.68890
Br	−2.01070	−5.92770	−0.36490
C	0.21930	−4.06660	−3.45940
C	1.14910	−4.68310	−4.20570
C	2.17310	−3.89700	−4.95410
C	3.12980	−4.67160	−5.82180
O	2.24950	−2.67420	−4.84660
C	−5.78140	−1.74150	−0.17450
O	−5.17750	0.16580	−1.39000
C	−5.95020	−1.82300	1.11550
C	−6.12780	−1.91140	2.39550
H	−5.97940	−2.81340	2.97390
Br	−6.49780	−0.36560	3.32710
H	−0.10110	−2.63620	−1.09400
H	0.29190	−5.12160	−0.86760
H	−1.05650	−3.96550	1.47080
H	0.43770	−3.07830	1.18210
H	−3.30010	−2.76640	0.76220
H	−1.71330	−1.81100	2.27210
H	−3.62160	−0.19650	0.79590
H	−2.73870	−0.14580	−0.71910
H	−4.40690	−1.68030	−1.78460
H	−0.86660	−5.84450	−2.99680
H	−1.82740	−4.38180	−2.98780
H	0.20320	−2.97780	−3.41490
H	1.19010	−5.76260	−4.29490
H	3.28010	−4.13110	−6.76060
H	4.08290	−4.78310	−5.29890
H	2.73160	−5.66060	−6.06440
H	−6.61040	−2.05170	−0.80580
H	−4.49560	0.55680	−1.96310

Table S6. *Cont.*

**Marilzafurollene_A_65**			
C	−0.83270	−3.28400	−0.59680
O	−2.12230	−2.71510	−0.89520
C	−0.65480	−4.70180	−1.15870
C	−0.63900	−3.14980	0.91300
C	−2.61810	−2.05170	0.28680
C	−1.42160	−1.89080	1.22830
C	−3.36370	−0.78120	−0.11390
Cl	−0.42010	−0.44580	0.88830
C	−4.66160	−1.07450	−0.87860
C	−0.55710	−4.78630	−2.69420
Br	−2.07750	−5.91300	−0.53700
C	−1.70300	−4.21090	−3.48350
C	−2.53010	−4.93760	−4.25040
C	−3.64220	−4.28730	−5.00330
C	−4.56510	−5.19440	−5.77400
O	−3.78610	−3.06580	−5.01820
C	−5.75260	−1.59180	0.01020
O	−5.14790	0.15280	−1.42650
C	−6.17470	−2.82350	0.04650
C	−6.59450	−4.04770	0.08560
H	−7.34070	−4.46280	−0.57830
Br	−5.88360	−5.16310	1.36790
H	−0.09620	−2.63760	−1.09250
H	0.26160	−5.13580	−0.74090
H	−1.09220	−3.99010	1.45020
H	0.41690	−3.11180	1.19880
H	−3.31370	−2.75580	0.75910
H	−1.72490	−1.82780	2.27690
H	−3.58430	−0.17220	0.77220
H	−2.72970	−0.16590	−0.76520
H	−4.48420	−1.76140	−1.71470
H	0.34970	−4.25310	−3.00890
H	−0.39750	−5.83330	−2.98550
H	−1.84370	−3.13210	−3.42090
H	−2.42940	−6.01280	−4.34060
H	−4.51350	−6.22100	−5.40100
H	−5.59440	−4.84520	−5.65250
H	−4.28800	−5.17860	−6.83090
H	−6.21770	−0.86960	0.67560
H	−4.58290	0.34940	−2.19490

Table S6. *Cont.*

**Marilzafurollene_A_66**			
C	−0.83330	−3.28380	−0.59720
O	−2.12160	−2.71530	−0.89470
C	−0.64160	−4.68720	−1.18910
C	−0.65420	−3.17900	0.91650
C	−2.61830	−2.05170	0.28670
C	−1.43160	−1.92030	1.24620
C	−3.34120	−0.76500	−0.10570
Cl	−0.41080	−0.47870	0.95300
C	−4.60110	−1.02370	−0.94180
C	−0.56060	−4.73890	−2.72730
Br	−2.03350	−5.93570	−0.57350
C	−1.75130	−4.22760	−3.49570
C	−2.51570	−4.99210	−4.29080
C	−3.67820	−4.41400	−5.02640
C	−4.49000	−5.36180	−5.87020
O	−3.94930	−3.21560	−4.97140
C	−5.72020	−1.61590	−0.13820
O	−5.07220	0.23650	−1.42520
C	−6.17590	−2.82960	−0.26210
C	−6.62910	−4.03730	−0.37490
H	−7.37670	−4.34900	−1.09180
Br	−6.04940	−5.29150	0.84400
H	−0.09620	−2.62350	−1.07320
H	0.28760	−5.11240	−0.79080
H	−1.11970	−4.02440	1.43460
H	0.39890	−3.15400	1.21410
H	−3.33200	−2.74840	0.74360
H	−1.74840	−1.87630	2.29180
H	−3.59820	−0.18390	0.78930
H	−2.67830	−0.13080	−0.70820
H	−4.36050	−1.64180	−1.81470
H	0.30790	−4.14730	−3.04510
H	−0.34160	−5.77040	−3.03500
H	−1.97980	−3.16690	−3.39460
H	−2.32290	−6.05060	−4.42080
H	−4.33770	−6.39880	−5.55890
H	−5.55170	−5.12850	−5.75010
H	−4.20000	−5.25170	−6.91800
H	−6.17160	−0.97230	0.61240
H	−5.73220	0.03220	−2.11160

Table S6. *Cont.*

**Marilzafurollene_A_67**			
C	−0.83290	−3.28290	−0.59400
O	−2.12480	−2.71520	−0.89920
C	−0.67530	−4.70430	−1.15630
C	−0.61380	−3.11810	0.90880
C	−2.61550	−2.05270	0.28800
C	−1.40840	−1.86420	1.21320
C	−3.38920	−0.79330	−0.09520
Cl	−0.42580	−0.41450	0.83720
C	−4.67220	−1.09350	−0.88650
C	−0.84360	−4.72530	−2.68620
Br	−2.01110	−5.91970	−0.35470
C	−0.35470	−6.00060	−3.32770
C	0.67430	−6.05830	−4.18790
C	1.09520	−7.35510	−4.79550
C	2.23210	−7.30980	−5.78240
O	0.55620	−8.41660	−4.48710
C	−5.78880	−1.76250	−0.13700
O	−5.21000	0.13890	−1.37360
C	−5.94020	−1.83770	1.15550
C	−6.10070	−1.91980	2.43820
H	−5.94200	−2.81840	3.01920
Br	−6.45540	−0.36890	3.36730
H	−0.10290	−2.64480	−1.11010
H	0.29800	−5.11390	−0.86330
H	−1.04280	−3.94980	1.47670
H	0.44690	−3.05990	1.17310
H	−3.29050	−2.77060	0.76940
H	−1.70000	−1.78740	2.26420
H	−3.62680	−0.20020	0.79680
H	−2.76210	−0.15280	−0.72870
H	−4.43390	−1.70600	−1.76390
H	−1.89470	−4.57970	−2.96580
H	−0.29180	−3.87960	−3.11690
H	−0.89550	−6.91360	−3.07910
H	1.23160	−5.17420	−4.47500
H	2.00560	−7.97500	−6.62050
H	2.36940	−6.30150	−6.18240
H	3.15220	−7.63190	−5.28870
H	−6.62480	−2.07930	−0.75560
H	−4.53810	0.52900	−1.95900

Table S6. *Cont.*

**Marilzafurollene_A_68**			
C	−0.83300	−3.28270	−0.59360
O	−2.12530	−2.71490	−0.89900
C	−0.68300	−4.70950	−1.14280
C	−0.60960	−3.10960	0.90780
C	−2.61480	−2.05320	0.28740
C	−1.40760	−1.85750	1.21060
C	−3.40640	−0.80390	−0.09100
Cl	−0.43140	−0.40540	0.83030
C	−4.63740	−1.10230	−0.95910
C	−0.84170	−4.74310	−2.67260
Br	−2.03960	−5.90780	−0.34780
C	−0.39270	−6.04330	−3.28960
C	−1.19990	−6.85810	−3.98670
C	−0.68330	−8.12990	−4.57050
C	−1.67440	−9.00160	−5.29590
O	0.50320	−8.44270	−4.48370
C	−5.65520	−1.97730	−0.28360
O	−5.26300	0.13330	−1.30880
C	−6.52200	−1.60120	0.61440
C	−7.38120	−1.22900	1.50910
H	−7.14320	−1.04190	2.54730
Br	−9.15750	−1.15210	1.02490
H	−0.10300	−2.65060	−1.11680
H	0.28320	−5.12700	−0.83810
H	−1.03310	−3.94130	1.48000
H	0.45190	−3.04670	1.16810
H	−3.28520	−2.77200	0.77600
H	−1.69790	−1.77970	2.26190
H	−3.70850	−0.26020	0.81330
H	−2.77340	−0.11520	−0.66460
H	−4.33430	−1.58280	−1.89670
H	−1.87750	−4.51960	−2.95880
H	−0.22190	−3.95330	−3.11600
H	0.65570	−6.30900	−3.15660
H	−2.24760	−6.62820	−4.14220
H	−1.49970	−10.04610	−5.02290
H	−2.70150	−8.74960	−5.01860
H	−1.54880	−8.87140	−6.37370
H	−5.57730	−3.04030	−0.49650
H	−4.62810	0.62740	−1.85500

Table S6. *Cont.*

**Marilzafurollene_A_69**			
C	−0.83300	−3.28390	−0.59710
O	−2.12200	−2.71500	−0.89430
C	−0.65890	−4.70300	−1.15850
C	−0.63140	−3.14030	0.91100
C	−2.61810	−2.05190	0.28630
C	−1.42050	−1.88500	1.22590
C	−3.37610	−0.78620	−0.10790
Cl	−0.42320	−0.43710	0.88610
C	−4.62390	−1.08010	−0.95030
C	−0.63270	−4.78700	−2.69760
Br	−2.02550	−5.93680	−0.46180
C	−1.83160	−4.23730	−3.42580
C	−1.78270	−3.19080	−4.26470
C	−3.01720	−2.67910	−4.92800
C	−2.86870	−1.45100	−5.78740
O	−4.10310	−3.24260	−4.80190
C	−5.72200	−1.72950	−0.16250
O	−5.13920	0.16740	−1.42180
C	−6.15780	−2.94290	−0.34620
C	−6.59050	−4.14990	−0.52720
H	−7.31270	−4.43660	−1.27980
Br	−6.08260	−5.43720	0.68940
H	−0.09910	−2.64020	−1.10010
H	0.28290	−5.12000	−0.78200
H	−1.07530	−3.97910	1.45770
H	0.42610	−3.09470	1.19000
H	−3.31050	−2.75930	0.76010
H	−1.72280	−1.82280	2.27490
H	−3.65240	−0.21340	0.78660
H	−2.73030	−0.13220	−0.70770
H	−4.35710	−1.67520	−1.83140
H	0.27160	−4.27040	−3.04620
H	−0.51150	−5.83450	−3.00340
H	−2.78660	−4.72660	−3.23710
H	−0.85650	−2.66850	−4.47280
H	−2.83840	−1.74700	−6.83890
H	−3.71880	−0.78590	−5.61100
H	−1.95660	−0.90140	−5.53920
H	−6.19320	−1.12440	0.60750
H	−5.85850	−0.05310	−2.04020

Table S7. Calculated Energies (Hartrees) for diastereoisomer S_a,4*R*,6*S*,7*S*,9*R*,10*R* of **1**.

Entry ID	Gas Phase Energy
1	−849,892312
2	−849,891362
3	−849,889723
4	−849,888014
5	−849,890752
6	−849,888881
7	−849,891284
8	−849,887862
9	−849,890823
10	−849,8903
11	−849,89047
12	−849,887116
13	−849,887459
14	−849,885981
15	−849,886131
16	−849,886643
17	−849,89112
18	−849,889149
19	−849,888072
20	−849,889069
21	−849,888506
22	−849,887669
23	−849,887238
24	−849,886538
25	−849,88672
26	−849,888164
27	−849,887177
28	−849,886464
29	−849,88626
30	−849,887009
31	−849,8872
32	−849,88652
33	−849,887065
34	−849,886281
35	−849,88669
36	−849,886453
37	−849,886943
38	−849,889594
39	−849,888768
40	−849,88626
41	−849,888689
42	−849,887404
43	−849,887051
44	−849,889586

45	−849,888582
46	−849,888918
47	−849,886761
48	−849,888629
49	−849,887692
50	−849,890553
51	−849,890372
52	−849,887395
53	−849,88919
54	−849,886823

Table S8. Coordinates (Angstroms) of calculated geometries for diastereoisomer S_a,4*R*,6*S*,7*S*,9*R*,10*R* of **1**.

**Marilzafurollene-A_1**			
C	−0.60640	1.28380	1.68150
O	0.17000	0.12240	1.31470
C	−1.72510	0.93540	2.67640
C	0.38090	2.36100	2.13110
C	1.56710	0.47880	1.38080
C	1.62790	2.00840	1.34520
C	2.36450	−0.27280	0.31760
Cl	1.52880	2.70040	−0.30250
C	2.29850	−1.79620	0.48100
C	−2.72540	−0.10030	2.13690
Br	−0.97130	0.18590	4.34580
C	−3.53510	0.41310	0.97640
C	−3.48450	−0.11370	−0.25700
C	−4.31440	0.44640	−1.36260
C	−4.20020	−0.21980	−2.70840
O	−5.03940	1.42470	−1.18950
C	2.93750	−2.28660	1.74600
O	3.01600	−2.38530	−0.60520
C	2.30180	−2.87100	2.72130
C	1.65840	−3.44090	3.68990
H	1.18210	−2.91230	4.50510
Br	1.44310	−5.26950	3.62550
H	−1.07230	1.63320	0.75160
H	−2.24820	1.84530	2.99180
H	0.00310	3.37320	1.95440
H	0.60660	2.28500	3.19970
H	1.91020	0.16460	2.37500
H	2.54270	2.39800	1.80010
H	1.97530	−0.04340	−0.68240
H	3.41020	0.06090	0.32400
H	1.25880	−2.13620	0.40530
H	−2.20400	−1.02690	1.86290
H	−3.43520	−0.37840	2.92720

H	−4.20010	1.25220	1.17880
H	−2.83780	−0.95090	−0.49430
H	−3.55110	0.37780	−3.35330
H	−3.78970	−1.22910	−2.61720
H	−5.19580	−0.30150	−3.15340
H	4.00960	−2.13820	1.84280
H	2.91420	−3.34930	−0.51110
**Marilzafurollene-A_2**			
C	0.13020	1.71480	1.05350
O	0.88890	0.49740	0.89270
C	−1.37580	1.44060	1.18540
C	0.77630	2.50160	2.19330
C	2.03480	0.56570	1.76770
C	2.21950	2.04290	2.12680
C	3.23040	−0.15260	1.14680
Cl	3.10270	2.98570	0.88780
C	2.97040	−1.64180	0.88820
C	−1.92890	0.72920	−0.06170
Br	−1.74120	0.30780	2.76490
C	−3.43540	0.71020	−0.11970
C	−4.16800	−0.41380	−0.15270
C	−5.65740	−0.35480	−0.21270
C	−6.39370	−1.66870	−0.21180
O	−6.25690	0.71670	−0.28470
C	2.75020	−2.43310	2.14310
O	4.12840	−2.18470	0.25110
C	1.63970	−3.03800	2.45550
C	0.52880	−3.62810	2.76330
H	−0.27180	−3.17220	3.33070
Br	0.24810	−5.33110	2.11880
H	0.28260	2.28230	0.12570
H	−1.91160	2.37790	1.37320
H	0.63730	3.58220	2.08650
H	0.36290	2.21930	3.16700
H	1.74140	0.04350	2.68750
H	2.75400	2.17700	3.07120
H	3.48490	0.29810	0.17920
H	4.11590	−0.03090	1.78400
H	2.13110	−1.76020	0.19280
H	−1.58540	1.25930	−0.95930
H	−1.52920	−0.29060	−0.13330
H	−3.93390	1.67870	−0.14660
H	−3.70940	−1.39570	−0.13100
H	−7.27960	−1.58280	0.42380
H	−6.69060	−1.91700	−1.23380
H	−5.76980	−2.47170	0.19000
H	3.58810	−2.50250	2.83150

H	3.91550	−3.11090	0.03830
	**Marilzafurollene-A_3**		
C	−0.57480	1.20940	1.67730
O	0.18940	0.03560	1.32240
C	−1.71290	0.88120	2.65730
C	0.42170	2.27230	2.14090
C	1.59080	0.36760	1.42060
C	1.67780	1.89600	1.38150
C	2.39430	−0.39780	0.37200
Cl	1.62280	2.58160	−0.27130
C	2.31530	−1.92080	0.54150
C	−2.72520	−0.13600	2.10550
Br	−0.99550	0.12100	4.33750
C	−3.51600	0.39540	0.94010
C	−3.48380	−0.14220	−0.28920
C	−4.29650	0.43650	−1.39810
C	−4.22160	−0.25460	−2.73410
O	−4.97740	1.44780	−1.23540
C	2.96740	−2.41370	1.79760
O	3.02100	−2.53120	−0.54130
C	2.33110	−2.85710	2.84480
C	1.68930	−3.28640	3.88430
H	1.26780	−2.65030	4.65120
Br	1.38400	−5.09750	4.02680
H	−1.02180	1.56430	0.74040
H	−2.22350	1.80080	2.96520
H	0.06420	3.28970	1.95230
H	0.62520	2.19820	3.21400
H	1.90770	0.05000	2.42190
H	2.59000	2.27200	1.85270
H	2.01780	−0.16840	−0.63290
H	3.44280	−0.07350	0.38870
H	1.27540	−2.26450	0.48820
H	−2.21680	−1.07010	1.83310
H	−3.44680	−0.40400	2.88850
H	−4.15310	1.25760	1.13580
H	−2.86780	−1.00400	−0.51980
H	−3.56140	0.31070	−3.39650
H	−3.84320	−1.27530	−2.63160
H	−5.22500	−0.31030	−3.16530
H	4.05350	−2.40540	1.81870
H	2.50530	−2.35200	−1.34670
	**Marilzafurollene-A_4**		
C	−0.87590	0.94090	1.83400
O	−0.33320	−0.33260	1.42260
C	−2.17350	0.78200	2.64210
C	0.25470	1.70870	2.51740

C	1.09500	−0.30300	1.63700
C	1.48290	1.16840	1.81350
C	1.81100	−1.07440	0.52970
Cl	1.73910	2.04050	0.27100
C	1.50170	−2.57960	0.55350
C	−3.28720	0.04840	1.87660
Br	−1.84900	−0.24370	4.30140
C	−3.78810	0.81880	0.68440
C	−3.67410	0.38730	−0.58130
C	−4.19210	1.20290	−1.71750
C	−4.03350	0.62820	−3.10050
O	−4.70240	2.30720	−1.53540
C	1.99470	−3.33030	1.75810
O	2.08980	−3.18910	−0.59780
C	3.10700	−3.11170	2.40160
C	4.20620	−2.88500	3.04780
H	5.12210	−3.44700	2.92160
Br	4.17850	−1.59480	4.36330
H	−1.11820	1.47180	0.90480
H	−2.52610	1.76090	2.98620
H	0.12810	2.79350	2.44310
H	0.32730	1.46450	3.58200
H	1.25880	−0.80190	2.59990
H	2.39770	1.28730	2.40060
H	1.49850	−0.69880	−0.45320
H	2.89450	−0.91150	0.58890
H	0.41950	−2.72840	0.46740
H	−2.94720	−0.95080	1.57410
H	−4.14740	−0.11580	2.53900
H	−4.27690	1.77100	0.88890
H	−3.19730	−0.55640	−0.82200
H	−3.18720	1.10950	−3.59670
H	−3.86520	−0.45150	−3.06400
H	−4.95050	0.80580	−3.66940
H	1.37540	−4.16440	2.07780
H	3.05510	−3.13420	−0.48760
**Marilzafurollene-A_5**			
C	0.15780	1.71440	0.99100
O	0.89500	0.48010	0.86660
C	−1.35200	1.47050	1.13990
C	0.82380	2.52680	2.10060
C	2.05310	0.56140	1.72460
C	2.26010	2.04660	2.03680
C	3.23180	−0.18980	1.11020
Cl	3.14530	2.94010	0.76320
C	2.94820	−1.68090	0.89090
C	−1.92500	0.72710	−0.07980

Br	−1.72870	0.40130	2.75930
C	−3.43530	0.70980	−0.12220
C	−4.15520	−0.42280	−0.14140
C	−5.64290	−0.48920	−0.18930
C	−6.44810	0.78340	−0.20590
O	−6.18310	−1.59550	−0.21690
C	2.72820	−2.43790	2.16660
O	4.09220	−2.25560	0.25650
C	1.60610	−3.00230	2.51190
C	0.48280	−3.54990	2.85150
H	−0.32160	−3.03560	3.36070
Br	0.19190	−5.30150	2.35980
H	0.31410	2.24780	0.04390
H	−1.87070	2.42290	1.29710
H	0.69820	3.60540	1.96160
H	0.41510	2.28000	3.08580
H	1.76560	0.07070	2.66340
H	2.80400	2.20220	2.97250
H	3.48250	0.23280	0.12910
H	4.12570	−0.06480	1.73500
H	2.10080	−1.80420	0.20620
H	−1.58990	1.23110	−0.99540
H	−1.52820	−0.29530	−0.12730
H	−3.92020	1.68230	−0.15050
H	−3.67200	−1.39580	−0.11940
H	−6.24550	1.36980	0.69400
H	−6.21980	1.36130	−1.10510
H	−7.51390	0.53530	−0.21860
H	3.57430	−2.51190	2.84450
H	3.86080	−3.18110	0.06010
**Marilzafurollene-A_6**			
C	0.15800	1.62640	1.04560
O	0.89270	0.39420	0.88390
C	−1.35320	1.38240	1.17700
C	0.81920	2.39980	2.18560
C	2.03820	0.43770	1.76110
C	2.25240	1.91100	2.12110
C	3.21700	−0.30010	1.13120
Cl	3.15740	2.83390	0.88230
C	2.93710	−1.78910	0.88620
C	−1.91890	0.68010	−0.06940
Br	−1.74140	0.25930	2.75780
C	−3.42510	0.69420	−0.13200
C	−4.18200	−0.41370	−0.16040
C	−5.66940	−0.32260	−0.22910
C	−6.43530	−1.61950	−0.22620
O	−6.24470	0.76170	−0.30850

C	2.77110	−2.57750	2.15000
O	4.06240	−2.35240	0.20850
C	1.64010	−3.04690	2.59540
C	0.51060	−3.50060	3.03750
H	−0.20950	−2.91600	3.59440
Br	0.05060	−5.23540	2.62150
H	0.32190	2.19090	0.11790
H	−1.87040	2.33030	1.36340
H	0.70300	3.48300	2.07790
H	0.39880	2.12700	3.15900
H	1.73570	−0.08030	2.67990
H	2.78850	2.03410	3.06610
H	3.46280	0.14310	0.15800
H	4.11210	−0.18560	1.75610
H	2.06480	−1.91930	0.23480
H	−1.56130	1.19980	−0.96760
H	−1.54180	−0.34850	−0.13730
H	−3.90210	1.67330	−0.16790
H	−3.74480	−1.40510	−0.12960
H	−5.82170	−2.44150	0.15230
H	−7.30500	−1.52040	0.42950
H	−6.75940	−1.84980	−1.24410
H	3.67210	−2.77000	2.72580
H	4.06980	−1.96800	−0.68550
**Marilzafurollene-A_7**			
C	−0.02800	1.39930	1.01470
O	0.81200	0.23070	0.90800
C	−1.49060	1.03040	1.30760
C	0.64330	2.34750	2.00710
C	2.02380	0.48100	1.65180
C	2.10950	1.99780	1.84730
C	3.21510	−0.20140	0.98350
Cl	2.79600	2.87310	0.44490
C	3.06600	−1.72540	0.89800
C	−2.08300	0.15370	0.18960
Br	−1.62920	0.05180	3.02040
C	−3.58690	0.02200	0.25790
C	−4.41460	0.54350	−0.66070
C	−5.89990	0.42380	−0.63480
C	−6.57000	−0.34530	0.47330
O	−6.54910	0.96510	−1.53040
C	3.04250	−2.39610	2.23910
O	4.19980	−2.23770	0.19540
C	2.01980	−3.03330	2.73400
C	0.99370	−3.65450	3.22220
H	0.16500	−3.17130	3.72280
Br	0.88580	−5.47620	2.97030

H	0.00080	1.87490	0.02520
H	−2.08460	1.94070	1.44710
H	0.40200	3.39710	1.81080
H	0.34250	2.13570	3.03820
H	1.86460	0.03600	2.64240
H	2.71060	2.27060	2.71900
H	3.33510	0.16400	−0.04420
H	4.14240	0.05650	1.51120
H	2.17640	−1.98260	0.31090
H	−1.80820	0.58340	−0.78270
H	−1.64900	−0.85400	0.21160
H	−3.97630	−0.54770	1.09820
H	−4.03420	1.10510	−1.50930
H	−6.35730	0.12220	1.43810
H	−7.65310	−0.33220	0.31730
H	−6.23650	−1.38620	0.46480
H	3.95180	−2.33880	2.83140
H	4.05080	−3.19490	0.09480
**Marilzafurollene-A_8**			
C	−0.87700	1.14340	1.78110
O	−0.28820	−0.13650	1.46080
C	−2.17200	0.99570	2.59540
C	0.22260	1.99530	2.41430
C	1.13530	−0.04270	1.68520
C	1.47340	1.45000	1.75470
C	1.89590	−0.87840	0.65930
Cl	1.70460	2.22040	0.15520
C	1.53590	−2.36940	0.69910
C	−3.26120	0.18520	1.87370
Br	−1.82110	0.08090	4.31330
C	−3.78800	0.87420	0.64340
C	−3.66370	0.37980	−0.59820
C	−4.21000	1.11810	−1.77350
C	−4.03380	0.47840	−3.12550
O	−4.75670	2.21270	−1.64710
C	1.81550	−3.03430	2.01870
O	2.27850	−3.02870	−0.32920
C	2.99170	−3.35820	2.47760
C	4.16280	−3.67070	2.93350
H	4.64480	−4.62800	2.78740
Br	5.10770	−2.37550	3.84170
H	−1.13230	1.60010	0.81660
H	−2.55420	1.98270	2.87990
H	0.05960	3.06730	2.26390
H	0.29550	1.82850	3.49380
H	1.30670	−0.45970	2.68600
H	2.38010	1.64270	2.33460

H	1.67370	−0.52820	−0.35660
H	2.97730	−0.74930	0.79640
H	0.47560	−2.50130	0.45510
H	−2.88850	−0.81690	1.62450
H	−4.11530	0.02890	2.54590
H	−4.30600	1.82030	0.79860
H	−3.15850	−0.55990	−0.79100
H	−3.20300	0.95960	−3.64730
H	−3.83200	−0.59230	−3.03390
H	−4.95590	0.59800	−3.70120
H	0.96650	−3.11980	2.69300
H	2.15440	−3.98480	−0.19950
**Marilzafurollene-A_9**			
C	−0.59890	1.28820	1.68060
O	0.17480	0.12200	1.32720
C	−1.73310	0.94940	2.66060
C	0.38950	2.35930	2.14130
C	1.57300	0.47440	1.39170
C	1.63980	2.00420	1.36170
C	2.36640	−0.27610	0.32470
Cl	1.55320	2.70470	−0.28300
C	2.29380	−1.80010	0.48000
C	−2.73330	−0.08130	2.11050
Br	−1.00720	0.20060	4.34220
C	−3.53830	0.43420	0.94370
C	−3.48290	−0.11080	−0.28150
C	−4.27170	0.35180	−1.45800
C	−5.20600	1.52460	−1.31660
O	−4.13920	−0.24300	−2.52790
C	2.92700	−2.29960	1.74440
O	3.01380	−2.38600	−0.60620
C	2.28560	−2.88030	2.71810
C	1.63680	−3.44570	3.68570
H	1.08150	−2.91250	4.44610
Br	1.57000	−5.28640	3.72210
H	−1.05080	1.63800	0.74390
H	−2.25470	1.86350	2.96630
H	0.01720	3.37390	1.96650
H	0.60860	2.27740	3.21080
H	1.91630	0.15570	2.38430
H	2.55340	2.38830	1.82360
H	1.97790	−0.04020	−0.67400
H	3.41360	0.05290	0.33220
H	1.25310	−2.13570	0.39890
H	−2.21270	−1.00960	1.84070
H	−3.44970	−0.35790	2.89540
H	−4.19620	1.27350	1.15580

H	−2.83140	−0.95500	−0.49180
H	−5.68390	1.72310	−2.28090
H	−5.98780	1.29730	−0.58740
H	−4.64840	2.41730	−1.02180
H	4.00010	−2.15970	1.84300
H	2.89360	−3.34920	−0.52720
**Marilzafurollene-A_10**			
C	−0.01540	1.39680	0.94960
O	0.81550	0.21880	0.88250
C	−1.47740	1.05140	1.27350
C	0.67340	2.38180	1.89330
C	2.03360	0.48850	1.60860
C	2.13490	2.01140	1.73730
C	3.21440	−0.23390	0.96440
Cl	2.81750	2.81810	0.29240
C	3.04990	−1.75870	0.94510
C	−2.08610	0.12720	0.20360
Br	−1.60200	0.15320	3.03090
C	−3.59090	0.03950	0.27010
C	−4.40850	0.46750	−0.70490
C	−5.88980	0.33650	−0.57710
C	−6.72000	0.80440	−1.74360
O	−6.41200	−0.10970	0.44300
C	3.02440	−2.37080	2.31390
O	4.17610	−2.31220	0.26160
C	2.00010	−2.98430	2.83490
C	0.97240	−3.58240	3.34800
H	0.13200	−3.07580	3.80400
Br	0.87760	−5.41640	3.20060
H	0.00560	1.83020	−0.05930
H	−2.06390	1.97120	1.37740
H	0.44010	3.42390	1.65230
H	0.37980	2.21960	2.93540
H	1.87590	0.08840	2.61840
H	2.74580	2.31620	2.59150
H	3.33270	0.08570	−0.07860
H	4.14710	0.03690	1.47590
H	2.15570	−2.03180	0.37240
H	−1.79750	0.49420	−0.79010
H	−1.67760	−0.88800	0.28590
H	−4.01700	−0.41950	1.16190
H	−4.02880	0.91690	−1.61520
H	−7.12940	1.79310	−1.52220
H	−6.12380	0.85590	−2.65870
H	−7.53320	0.09300	−1.91290
H	3.93310	−2.28910	2.90420
H	4.01880	−3.27180	0.20620

**Marilzafurollene-A_11**			
C	−0.48990	1.13260	1.40400
O	0.38880	0.01020	1.17750
C	−1.74550	0.72740	2.19190
C	0.35690	2.24850	2.01660
C	1.73880	0.43060	1.46650
C	1.73270	1.96200	1.44880
C	2.72950	−0.27070	0.54010
Cl	1.87890	2.67130	−0.18770
C	2.70960	−1.79770	0.68170
C	−2.60020	−0.34860	1.49960
Br	−1.25040	0.00340	3.96830
C	−3.23420	0.12370	0.21780
C	−4.55330	0.31760	0.06320
C	−5.11120	0.77430	−1.24340
C	−6.60950	0.88970	−1.34420
O	−4.37970	1.06850	−2.18720
C	3.16030	−2.27510	2.03000
O	3.62080	−2.33840	−0.27710
C	2.40590	−2.90100	2.88790
C	1.64480	−3.51220	3.73880
H	0.94780	−3.01890	4.40340
Br	1.67530	−5.35410	3.75850
H	−0.80350	1.46900	0.40830
H	−2.35370	1.61160	2.41430
H	−0.03310	3.24440	1.78300
H	0.40530	2.17070	3.10750
H	1.93390	0.12030	2.50110
H	2.53950	2.38640	2.05260
H	2.49410	−0.04620	−0.50790
H	3.74410	0.10980	0.71510
H	1.71300	−2.18390	0.43720
H	−1.99560	−1.23760	1.27880
H	−3.38850	−0.68840	2.18500
H	−2.56580	0.28520	−0.62780
H	−5.25650	0.14950	0.87080
H	−7.10500	0.30130	−0.56720
H	−6.89860	1.93870	−1.24360
H	−6.93400	0.50430	−2.31500
H	4.19490	−2.07860	2.29820
H	3.53830	−3.30700	−0.21760
**Marilzafurollene-A_12**			
C	−0.14240	1.32930	1.38480
O	0.50100	0.14050	0.87920
C	−1.67320	1.24350	1.29360
C	0.43950	1.59860	2.77090
C	1.57400	−0.20770	1.78170

C	1.83780	1.02820	2.64820
C	2.76250	−0.76580	1.00080
Cl	2.92320	2.23060	1.88460
C	2.45110	−2.10250	0.30950
C	−2.14020	1.05380	−0.15990
Br	−2.35320	−0.26750	2.37070
C	−3.61910	1.28160	−0.34520
C	−4.46960	0.34310	−0.78910
C	−5.92230	0.64140	−0.94920
C	−6.80590	−0.48910	−1.40720
O	−6.37570	1.76540	−0.73920
C	2.16430	−3.26160	1.22160
O	3.56560	−2.47020	−0.50630
C	2.73370	−3.50080	2.36950
C	3.29150	−3.73380	3.51490
H	4.11430	−4.41880	3.67110
Br	2.55720	−2.93860	5.00620
H	0.18000	2.14320	0.72150
H	−2.12390	2.13820	1.73780
H	0.40260	2.65860	3.04180
H	−0.09560	1.04690	3.55060
H	1.16600	−0.98590	2.43820
H	2.27990	0.76960	3.61430
H	3.06030	−0.06100	0.21380
H	3.63410	−0.87560	1.65840
H	1.59890	−1.97080	−0.36660
H	−1.62650	1.78180	−0.80090
H	−1.85510	0.06090	−0.53100
H	−3.99190	2.27750	−0.10780
H	−4.13660	−0.65820	−1.03680
H	−6.32820	−1.45820	−1.23960
H	−7.73720	−0.47060	−0.83420
H	−7.02060	−0.37100	−2.47220
H	1.45680	−3.99400	0.84110
H	4.30690	−2.66360	0.09370
**Marilzafurollene-A_13**			
C	−0.84590	0.89100	1.83940
O	−0.26450	−0.36200	1.41530
C	−2.15280	0.68630	2.62190
C	0.25230	1.67580	2.55630
C	1.15470	−0.31010	1.68330
C	1.50870	1.16670	1.87970
C	1.91940	−1.06080	0.59530
Cl	1.78240	2.05370	0.34820
C	1.64960	−2.57380	0.61560
C	−3.23600	−0.05820	1.82410
Br	−1.83230	−0.36270	4.26720

C	−3.73680	0.72460	0.63990
C	−3.60610	0.31610	−0.63190
C	−4.12800	1.14370	−1.75790
C	−3.95930	0.59160	−3.14890
O	−4.65060	2.23960	−1.56120
C	2.16110	−3.31510	1.81720
O	2.27460	−3.17400	−0.52060
C	3.20270	−3.00320	2.53620
C	4.23970	−2.68970	3.24540
H	5.24060	−3.05860	3.06490
Br	3.99490	−1.55890	4.67910
H	−1.08510	1.43150	0.91490
H	−2.53420	1.65010	2.97820
H	0.10530	2.75830	2.48690
H	0.30290	1.42550	3.62080
H	1.29160	−0.81220	2.64840
H	2.40640	1.30020	2.48960
H	1.61980	−0.69060	−0.39370
H	2.99660	−0.86990	0.67930
H	0.57280	−2.76000	0.52670
H	−2.86710	−1.04240	1.50710
H	−4.10230	−0.25600	2.46910
H	−4.24130	1.66620	0.85580
H	−3.11590	−0.61720	−0.88440
H	−3.13070	1.10510	−3.64290
H	−3.75650	−0.48260	−3.12770
H	−4.88440	0.74790	−3.71080
H	1.66010	−4.25450	2.03440
H	1.87910	−2.76560	−1.30980
**Marilzafurollene-A_14**			
C	−0.84040	1.20960	1.80490
O	−0.33870	−0.11530	1.52710
C	−2.13370	1.17420	2.63430
C	0.32060	2.01080	2.39210
C	1.09480	−0.09970	1.69620
C	1.52280	1.37200	1.72600
C	1.76100	−0.99460	0.65290
Cl	1.77840	2.09170	0.10700
C	1.31310	−2.46140	0.73430
C	−3.26760	0.37020	1.97590
Br	−1.81190	0.35370	4.40560
C	−3.74480	0.96600	0.67860
C	−3.65490	0.33660	−0.50360
C	−4.14940	0.98010	−1.75480
C	−4.04900	0.17410	−3.02330
O	−4.59520	2.12640	−1.75480
C	1.64060	−3.13510	2.03740

O	1.92310	−3.18990	−0.33350
C	2.83030	−3.46870	2.45270
C	4.01180	−3.79680	2.86880
H	4.38350	−4.81130	2.92770
Br	5.11430	−2.45120	3.47570
H	−1.07890	1.64550	0.82640
H	−2.46660	2.19420	2.85780
H	0.21930	3.08610	2.21400
H	0.40800	1.86970	3.47400
H	1.28000	−0.51240	2.69630
H	2.44950	1.52050	2.28730
H	1.51370	−0.64760	−0.35830
H	2.85290	−0.92720	0.74060
H	0.23180	−2.52620	0.57000
H	−2.95720	−0.67250	1.82720
H	−4.13270	0.33120	2.65110
H	−4.19510	1.95680	0.73110
H	−3.21770	−0.65170	−0.59220
H	−3.97100	−0.89480	−2.80680
H	−4.95190	0.33020	−3.62020
H	−3.16930	0.49490	−3.58680
H	0.81470	−3.22350	2.73930
H	2.88130	−3.19800	−0.16560
**Marilzafurollene-A_15**			
C	−0.14160	1.47860	1.34410
O	0.50480	0.24560	0.96220
C	−1.67090	1.39000	1.23300
C	0.41600	1.87210	2.71070
C	1.55990	−0.01910	1.91250
C	1.81490	1.29060	2.66610
C	2.75720	−0.66210	1.21790
Cl	2.91560	2.41830	1.81650
C	2.42660	−2.00170	0.54650
C	−2.11290	1.07540	−0.20650
Br	−2.37600	−0.02030	2.42500
C	−3.58700	1.29130	−0.43800
C	−4.43430	0.32140	−0.81570
C	−5.88220	0.61070	−1.02800
C	−6.76360	−0.55130	−1.40360
O	−6.33290	1.75040	−0.92370
C	1.91430	−3.05390	1.49080
O	3.61420	−2.48010	−0.08920
C	2.61800	−3.67040	2.39840
C	3.32020	−4.26810	3.30750
H	3.78050	−5.23990	3.19060
Br	3.61500	−3.37380	4.89160
H	0.19720	2.22690	0.61490

H	−2.12550	2.32130	1.58940
H	0.37760	2.95240	2.88320
H	−0.13540	1.39450	3.52700
H	1.13750	−0.73000	2.63450
H	2.23850	1.12090	3.65980
H	3.13990	−0.00120	0.42990
H	3.58050	−0.79290	1.93200
H	1.68540	−1.84610	−0.24580
H	−1.58420	1.74270	−0.89920
H	−1.82620	0.05280	−0.48410
H	−3.95900	2.30530	−0.29390
H	−4.10240	−0.69870	−0.97070
H	−7.70390	−0.48140	−0.84950
H	−6.96060	−0.52270	−2.47820
H	−6.29340	−1.50460	−1.14730
H	0.83600	−3.19380	1.50220
H	3.43130	−3.38880	−0.38420
**Marilzafurollene-A_16**			
C	−0.09720	1.29120	1.38540
O	0.55200	0.09420	0.90550
C	−1.62750	1.20070	1.28770
C	0.47640	1.58980	2.76930
C	1.60830	−0.24410	1.83250
C	1.87280	1.01010	2.67100
C	2.79970	−0.82820	1.07640
Cl	2.96910	2.18760	1.88380
C	2.48900	−2.19240	0.44030
C	−2.08620	0.98090	−0.16410
Br	−2.31100	−0.28940	2.39110
C	−3.56350	1.20690	−0.36400
C	−4.41190	0.26320	−0.80100
C	−5.86240	0.56240	−0.98000
C	−6.74230	−0.56870	−1.44360
O	−6.31670	1.68800	−0.78090
C	2.24590	−3.32310	1.39820
O	3.59430	−2.58870	−0.37400
C	2.74970	−3.45030	2.59360
C	3.25510	−3.57270	3.77960
H	4.18200	−4.08450	4.00150
Br	2.32200	−2.84970	5.19400
H	0.22720	2.09260	0.70800
H	−2.08190	2.10340	1.71150
H	0.44280	2.65600	3.01500
H	−0.06750	1.05960	3.55780
H	1.18280	−1.00250	2.50050
H	2.30710	0.77320	3.64610
H	3.09420	−0.15070	0.26460

H	3.67240	−0.91110	1.73640
H	1.61790	−2.10480	−0.21940
H	−1.56760	1.69460	−0.81700
H	−1.80030	−0.01990	−0.51260
H	−3.93690	2.20690	−0.14510
H	−4.07850	−0.74200	−1.03170
H	−6.26320	−1.53740	−1.27790
H	−7.67520	−0.55360	−0.87310
H	−6.95410	−0.44810	−2.50890
H	1.67100	−4.15530	1.00110
H	3.69100	−1.91270	−1.06690
**Marilzafurollene-A_17**			
C	−0.46590	1.17020	1.41000
O	0.37590	0.02300	1.17000
C	−1.70420	0.80710	2.24400
C	0.42880	2.27570	1.97100
C	1.74490	0.41320	1.40950
C	1.77630	1.94370	1.36140
C	2.68780	−0.33040	0.46660
Cl	1.88000	2.61640	−0.29410
C	2.63560	−1.85340	0.64000
C	−2.59080	−0.28090	1.61250
Br	−1.17170	0.13480	4.02940
C	−3.20680	0.12360	0.29640
C	−4.51150	0.40090	0.14930
C	−5.16390	0.77900	−1.13610
C	−4.34850	0.83540	−2.40120
O	−6.36720	1.04020	−1.13030
C	3.11960	−2.31530	1.98210
O	3.49910	−2.43560	−0.33810
C	2.37830	−2.90190	2.87830
C	1.63050	−3.47430	3.76720
H	0.98920	−2.94720	4.46130
Br	1.57840	−5.31560	3.79660
H	−0.80710	1.49790	0.42020
H	−2.29530	1.70610	2.45220
H	0.05540	3.27660	1.73130
H	0.51440	2.21790	3.06070
H	1.96440	0.11750	2.44350
H	2.61440	2.35940	1.92730
H	2.42440	−0.12070	−0.57770
H	3.71650	0.02800	0.60160
H	1.62190	−2.21900	0.43730
H	−2.01460	−1.20130	1.45100
H	−3.38980	−0.55360	2.31540
H	−2.52990	0.14900	−0.55450
H	−5.19420	0.36310	0.99380

H	−4.99920	1.10810	−3.23760
H	−3.56790	1.59530	−2.31200
H	−3.91320	−0.14460	−2.61310
H	4.16790	−2.14290	2.21070
H	3.40190	−3.40070	−0.25060
	**Marilzafurollene-A_18**		
C	0.00240	1.31610	1.00780
O	0.82330	0.13270	0.91150
C	−1.46670	0.97490	1.30290
C	0.68900	2.26240	1.99160
C	2.03540	0.36650	1.66010
C	2.14870	1.88350	1.84020
C	3.21400	−0.34210	0.99680
Cl	2.85590	2.72900	0.42940
C	3.04810	−1.86650	0.93770
C	−2.07170	0.09380	0.19520
Br	−1.62470	0.02190	3.02790
C	−3.57780	−0.01060	0.26150
C	−4.39410	0.51250	−0.66640
C	−5.88130	0.41900	−0.64280
C	−6.56740	−0.32320	0.47380
O	−6.51860	0.95920	−1.54750
C	3.06550	−2.51850	2.28710
O	4.15500	−2.41130	0.21590
C	2.02280	−3.01640	2.88930
C	0.97830	−3.49890	3.48380
H	0.26280	−2.90950	4.04140
Br	0.62340	−5.29600	3.28850
H	0.03950	1.78200	0.01400
H	−2.04550	1.89680	1.42940
H	0.46830	3.31410	1.78280
H	0.38150	2.06840	3.02420
H	1.86500	−0.06630	2.65390
H	2.75270	2.15440	2.71050
H	3.33040	0.00680	−0.03700
H	4.14780	−0.08730	1.51430
H	2.13680	−2.13820	0.39170
H	−1.78710	0.50550	−0.78200
H	−1.65550	−0.92100	0.23180
H	−3.97900	−0.56190	1.10850
H	−4.00200	1.05560	−1.52170
H	−6.34760	0.15250	1.43300
H	−7.64980	−0.29190	0.31570
H	−6.25320	−1.37020	0.47910
H	4.02870	−2.58170	2.78550
H	4.04720	−2.13300	−0.71040

**Marilzafurollene-A_19**			
C	0.18500	1.63460	0.97120
O	0.89980	0.38640	0.84900
C	−1.32850	1.41820	1.12340
C	0.86800	2.43930	2.07620
C	2.05320	0.44460	1.71490
C	2.29320	1.92680	2.01750
C	3.21510	−0.33490	1.10420
Cl	3.19960	2.78900	0.73660
C	2.90950	−1.82700	0.91650
C	−1.91540	0.67990	−0.09270
Br	−1.72190	0.36280	2.74760
C	−3.42540	0.69350	−0.13540
C	−4.16910	−0.42380	−0.14440
C	−5.65790	−0.45760	−0.19020
C	−6.43510	0.83240	−0.20020
O	−6.22230	−1.55170	−0.22000
C	2.73170	−2.56360	2.20950
O	4.02400	−2.43450	0.25980
C	1.59610	−3.01440	2.66270
C	0.46200	−3.45080	3.11030
H	−0.27070	−2.84180	3.62310
Br	0.01280	−5.20440	2.76770
H	0.34900	2.16200	0.02200
H	−1.83010	2.38020	1.27740
H	0.76690	3.51950	1.92960
H	0.45240	2.20890	3.06250
H	1.75050	−0.03390	2.65490
H	2.84010	2.07700	2.95230
H	3.46070	0.06790	0.11350
H	4.11670	−0.21180	1.71790
H	2.03390	−1.96640	0.27130
H	−1.56990	1.17240	−1.01070
H	−1.53940	−0.35060	−0.13470
H	−3.88970	1.67570	−0.17300
H	−3.70690	−1.40670	−0.11430
H	−6.19780	1.40760	−1.09880
H	−7.50610	0.60780	−0.20880
H	−6.21560	1.41170	0.70040
H	3.62660	−2.73080	2.80220
H	4.03230	−2.08990	−0.65020
**Marilzafurollene-A_20**			
C	−0.56780	1.22930	1.68910
O	0.18890	0.04840	1.34700
C	−1.71630	0.91550	2.66100
C	0.43460	2.28650	2.15190
C	1.59250	0.37540	1.42760

C	1.68640	1.90370	1.38820
C	2.37910	−0.39420	0.36940
Cl	1.62900	2.59180	−0.26350
C	2.29020	−1.91680	0.53780
C	−2.72590	−0.10650	2.11170
Br	−1.01800	0.17380	4.35720
C	−3.50660	0.40470	0.92660
C	−3.44320	−0.15980	−0.28940
C	−4.20570	0.29790	−1.48470
C	−5.13530	1.47760	−1.37140
O	−4.05460	−0.30600	−2.54710
C	2.95080	−2.41560	1.78720
O	2.98090	−2.53120	−0.55220
C	2.32100	−2.86100	2.83730
C	1.68450	−3.29370	3.87870
H	1.25460	−2.65950	4.64250
Br	1.39100	−5.10680	4.02170
H	−1.00620	1.58180	0.74700
H	−2.22790	1.83980	2.95240
H	0.08200	3.30600	1.96540
H	0.64150	2.20940	3.22410
H	1.91960	0.05630	2.42520
H	2.60210	2.27520	1.85630
H	1.99350	−0.16090	−0.63100
H	3.43010	−0.07760	0.37510
H	1.24730	−2.25270	0.49440
H	−2.21690	−1.04680	1.86210
H	−3.45700	−0.36030	2.89080
H	−4.15280	1.25820	1.11670
H	−2.80270	−1.01770	−0.47710
H	−5.60270	1.66290	−2.34340
H	−5.92570	1.26600	−0.64670
H	−4.57560	2.37170	−1.08500
H	4.03710	−2.41090	1.79970
H	2.45120	−2.35800	−1.35010
**Marilzafurollene-A_21**			
C	−0.46940	1.06470	1.41890
O	0.39000	−0.07550	1.20500
C	−1.73950	0.68860	2.19780
C	0.39380	2.16820	2.03070
C	1.74580	0.32220	1.49990
C	1.76670	1.85350	1.47140
C	2.72250	−0.40090	0.57580
Cl	1.93220	2.54570	−0.17120
C	2.69790	−1.92580	0.74580
C	−2.60680	−0.37730	1.50560
Br	−1.27510	−0.02860	3.98470

C	−3.21150	0.09190	0.20870
C	−4.52430	0.30690	0.03010
C	−5.05210	0.75780	−1.29090
C	−6.54650	0.89330	−1.42000
O	−4.29950	1.03180	−2.22420
C	3.20820	−2.38060	2.07950
O	3.56720	−2.50150	−0.23180
C	2.47020	−2.87440	3.03310
C	1.72720	−3.35590	3.97810
H	1.13470	−2.75780	4.65760
Br	1.56630	−5.18700	4.10350
H	−0.76860	1.40100	0.41870
H	−2.33360	1.58550	2.40670
H	0.02340	3.16980	1.78980
H	0.43510	2.09480	3.12210
H	1.93180	0.01470	2.53670
H	2.57860	2.26840	2.07510
H	2.47460	−0.19160	−0.47250
H	3.74130	−0.02370	0.73240
H	1.69210	−2.32210	0.56290
H	−2.01790	−1.28140	1.30440
H	−3.41180	−0.69200	2.18350
H	−2.52700	0.23190	−0.62780
H	−5.24360	0.15990	0.82750
H	−7.06420	0.31860	−0.64710
H	−6.82270	1.94710	−1.33360
H	−6.85880	0.50400	−2.39310
H	4.27770	−2.29180	2.24810
H	3.14330	−2.36220	−1.09670
**Marilzafurollene-A_22**			
C	−0.31070	1.00560	1.25960
O	0.40990	−0.17890	0.85960
C	−1.82920	0.77420	1.29560
C	0.33090	1.50600	2.55210
C	1.56530	−0.31230	1.71640
C	1.76630	1.04580	2.39690
C	2.74970	−0.86730	0.92720
Cl	2.68170	2.22430	1.40690
C	2.51920	−2.30300	0.42950
C	−2.36610	0.35310	−0.08420
Br	−2.28790	−0.62840	2.60980
C	−3.87200	0.41940	−0.19240
C	−4.51670	1.29420	−0.97980
C	−5.99760	1.38070	−1.12230
C	−6.88030	0.42820	−0.35940
O	−6.46720	2.24070	−1.86830
C	2.38940	−3.35420	1.49520

O	3.61340	−2.68090	−0.40890
C	3.04440	−3.39790	2.62130
C	3.68710	−3.43740	3.74500
H	4.57390	−4.03010	3.92660
Br	2.97550	−2.51860	5.17500
H	−0.11130	1.74980	0.47670
H	−2.33520	1.67830	1.65260
H	0.21150	2.58560	2.68820
H	−0.09810	1.01980	3.43410
H	1.27370	−1.02780	2.49490
H	2.29180	0.95670	3.35170
H	2.93440	−0.24800	0.04000
H	3.66590	−0.81940	1.52920
H	1.62130	−2.32880	−0.19800
H	−1.92610	1.00420	−0.85090
H	−2.04900	−0.66780	−0.33220
H	−4.42300	−0.30840	0.39840
H	−3.97440	2.01770	−1.58210
H	−6.73910	0.56330	0.71610
H	−7.92770	0.63890	−0.59650
H	−6.66440	−0.60240	−0.65240
H	1.72740	−4.18310	1.25800
H	4.40150	−2.73830	0.15900
**Marilzafurollene-A_23**			
C	−0.31490	1.17600	1.23340
O	0.40810	−0.04290	0.95910
C	−1.83420	0.95120	1.27050
C	0.31070	1.79680	2.48110
C	1.55370	−0.09060	1.83750
C	1.74870	1.32700	2.38620
C	2.74570	−0.73020	1.13050
Cl	2.67090	2.40980	1.29850
C	2.47860	−2.16580	0.65930
C	−2.35370	0.40560	−0.07190
Br	−2.31280	−0.32510	2.70140
C	−3.85770	0.46380	−0.20660
C	−4.48880	1.26220	−1.08140
C	−5.96730	1.33810	−1.25240
C	−6.86380	0.46330	−0.41610
O	−6.42350	2.12510	−2.08240
C	2.12390	−3.12070	1.76550
O	3.65200	−2.63430	−0.00950
C	2.94970	−3.60860	2.64800
C	3.77220	−4.08400	3.52810
H	4.31810	−5.01190	3.42230
Br	4.09100	−3.08020	5.04010
H	−0.10280	1.84150	0.38570

H	−2.34280	1.88480	1.53620
H	0.18840	2.88410	2.51390
H	−0.12860	1.39420	3.39950
H	1.25420	−0.72520	2.68160
H	2.26380	1.33090	3.35070
H	3.01400	−0.15150	0.23770
H	3.62700	−0.70710	1.78450
H	1.67130	−2.16830	−0.08210
H	−1.90160	0.98280	−0.88910
H	−2.03590	−0.63420	−0.22050
H	−4.41920	−0.20430	0.44210
H	−3.93600	1.92520	−1.74110
H	−6.73810	0.69930	0.64380
H	−7.90690	0.65200	−0.68790
H	−6.64650	−0.59080	−0.60710
H	1.06120	−3.30110	1.91030
H	3.51810	−3.58180	−0.18490
**Marilzafurollene-A_24**			
C	−0.10040	1.52670	1.26920
O	0.51480	0.26360	0.93810
C	−1.63330	1.46240	1.19210
C	0.49290	1.97760	2.60260
C	1.58340	0.02580	1.88030
C	1.87960	1.36730	2.55940
C	2.75300	−0.67900	1.19850
Cl	2.98130	2.42860	1.62880
C	2.37810	−2.03630	0.58870
C	−2.10960	1.08160	−0.22080
Br	−2.34180	0.13500	2.47190
C	−3.59050	1.28980	−0.43430
C	−4.42790	0.30090	−0.78390
C	−5.89140	0.46270	−1.01120
C	−6.53210	1.81310	−0.82620
O	−6.54780	−0.52250	−1.35040
C	1.85200	−3.03310	1.58440
O	3.54370	−2.57030	−0.04340
C	2.56410	−3.69460	2.45290
C	3.27520	−4.34330	3.31920
H	3.70690	−5.32100	3.15260
Br	3.63490	−3.52030	4.92810
H	0.23690	2.23010	0.49620
H	−2.06290	2.42060	1.50530
H	0.47800	3.06580	2.72080
H	−0.04970	1.55250	3.45320
H	1.16160	−0.63780	2.64630
H	2.32180	1.24100	3.55140
H	3.14240	−0.06320	0.37800

H	3.58220	−0.80170	1.90720
H	1.62920	−1.89470	−0.19890
H	−1.59420	1.71460	−0.95460
H	−1.82930	0.04650	−0.45530
H	−3.94980	2.30720	−0.30110
H	−4.07000	−0.71540	−0.92400
H	−6.11350	2.52900	−1.53820
H	−7.60710	1.73090	−1.01380
H	−6.39090	2.15990	0.20080
H	0.76920	−3.09110	1.66640
H	3.32840	−3.48090	−0.31000
**Marilzafurollene-A_25**			
C	−0.10840	1.34990	1.34020
O	0.50410	0.13400	0.86240
C	−1.64200	1.28890	1.27790
C	0.50440	1.65350	2.70570
C	1.58810	−0.20420	1.75540
C	1.89010	1.05420	2.57570
C	2.75080	−0.80910	0.97070
Cl	2.98100	2.21180	1.75280
C	2.40010	−2.15840	0.32390
C	−2.13720	1.05470	−0.16020
Br	−2.33150	−0.16520	2.42290
C	−3.62060	1.28380	−0.33160
C	−4.46410	0.33570	−0.76850
C	−5.93010	0.52050	−0.95920
C	−6.56540	1.84610	−0.63130
O	−6.59300	−0.42510	−1.38690
C	2.10390	−3.28380	1.27430
O	3.49350	−2.57290	−0.49820
C	2.69140	−3.50500	2.41670
C	3.26730	−3.72000	3.55660
H	4.07480	−4.42240	3.71570
Br	2.58610	−2.86320	5.03890
H	0.21580	2.13560	0.64450
H	−2.06870	2.20820	1.69460
H	0.49120	2.72220	2.94240
H	−0.02490	1.13690	3.51290
H	1.17940	−0.95330	2.44450
H	2.34630	0.81930	3.54130
H	3.04860	−0.13370	0.15820
H	3.63180	−0.91770	1.61590
H	1.54000	−2.02800	−0.34240
H	−1.63070	1.75950	−0.83210
H	−1.86160	0.04900	−0.50310
H	−3.97640	2.28260	−0.09140
H	−4.10960	−0.66130	−1.01540

H	−6.41080	2.08770	0.42350
H	−6.15400	2.62950	−1.27300
H	−7.64280	1.78420	−0.81290
H	1.36960	−4.00770	0.92990
H	4.23810	−2.77120	0.09610
**Marilzafurollene-A_26**			
C	0.01370	1.31300	0.94210
O	0.82530	0.12030	0.89120
C	−1.45470	0.99740	1.26820
C	0.71790	2.29920	1.87310
C	2.04410	0.37710	1.62090
C	2.17270	1.89970	1.72790
C	3.21190	−0.37320	0.98520
Cl	2.87510	2.67040	0.27240
C	3.03130	−1.89700	0.99970
C	−2.07710	0.06640	0.21210
Br	−1.59910	0.13110	3.03980
C	−3.58350	0.00860	0.27510
C	−4.38980	0.43600	−0.70940
C	−5.87380	0.33600	−0.58430
C	−6.69130	0.79860	−1.76180
O	−6.40770	−0.08170	0.44180
C	3.04720	−2.48340	2.37870
O	4.13040	−2.48670	0.30190
C	2.00530	−2.96140	2.99820
C	0.96150	−3.42670	3.60740
H	0.23020	−2.81870	4.12320
Br	0.62650	−5.23450	3.49060
H	0.04310	1.73220	−0.07260
H	−2.02530	1.92890	1.35560
H	0.50480	3.34160	1.61540
H	0.41810	2.15870	2.91650
H	1.87590	−0.00660	2.63510
H	2.78670	2.20600	2.57920
H	3.32540	−0.07500	−0.06460
H	4.15130	−0.10310	1.48470
H	2.11550	−2.18520	0.47000
H	−1.77860	0.41090	−0.78670
H	−1.68820	−0.95470	0.31320
H	−4.02090	−0.42720	1.17320
H	−3.99880	0.86260	−1.62580
H	−6.08930	0.83140	−2.67400
H	−7.51200	0.09470	−1.92680
H	−7.09010	1.79490	−1.55570
H	4.00840	−2.51630	2.88370
H	4.02080	−2.25450	−0.63670

**Marilzafurollene-A_27**			
C	−0.27590	0.96480	1.28130
O	0.44680	−0.22930	0.91300
C	−1.79550	0.73900	1.30330
C	0.35240	1.49000	2.57060
C	1.59210	−0.34740	1.78720
C	1.78850	1.02450	2.44030
C	2.78040	−0.91730	1.01540
Cl	2.71560	2.17910	1.43250
C	2.56100	−2.37410	0.57730
C	−2.31570	0.28750	−0.07340
Br	−2.27710	−0.63050	2.64320
C	−3.81940	0.36000	−0.20460
C	−4.44690	1.21730	−1.02460
C	−5.92510	1.30980	−1.19090
C	−6.82550	0.38460	−0.41520
O	−6.37760	2.15200	−1.96700
C	2.49440	−3.38760	1.68340
O	3.64140	−2.77390	−0.26790
C	3.10550	−3.32300	2.83290
C	3.71690	−3.25480	3.97230
H	4.70090	−3.66070	4.16590
Br	2.84710	−2.42730	5.36960
H	−0.06540	1.69240	0.48590
H	−2.30270	1.65300	1.63230
H	0.23360	2.57230	2.68350
H	−0.08700	1.02230	3.45750
H	1.29010	−1.04670	2.57600
H	2.30470	0.95580	3.40180
H	2.95150	−0.32710	0.10590
H	3.69990	−0.83730	1.60870
H	1.64250	−2.44920	−0.01650
H	−1.86100	0.91720	−0.84930
H	−2.00150	−0.74100	−0.29200
H	−4.38360	−0.34790	0.39780
H	−3.89130	1.92080	−1.63830
H	−6.69960	0.54920	0.65810
H	−7.86770	0.59500	−0.67430
H	−6.61200	−0.65520	−0.67580
H	1.96150	−4.30410	1.44520
H	3.62320	−2.18880	−1.04490
**Marilzafurollene-A_28**			
C	−0.82220	1.15020	1.80540
O	−0.28440	−0.15760	1.51000
C	−2.12930	1.06920	2.61000
C	0.30690	1.96720	2.43320
C	1.14020	−0.12100	1.74470

C	1.54040	1.35720	1.79870
C	1.86450	−0.99590	0.72430
Cl	1.82700	2.09290	0.19140
C	1.44880	−2.47280	0.78910
C	−3.23690	0.26340	1.91070
Br	−1.82350	0.20750	4.36460
C	−3.71080	0.89170	0.62770
C	−3.61820	0.29250	−0.56970
C	−4.11370	0.96550	−1.80500
C	−4.01890	0.18620	−3.09040
O	−4.55390	2.11350	−1.77900
C	1.75740	−3.13850	2.09870
O	2.12830	−3.19390	−0.24060
C	2.94800	−3.39700	2.56140
C	4.13580	−3.65380	3.00760
H	4.70320	−4.54090	2.76030
Br	4.96910	−2.36660	4.02890
H	−1.05170	1.60040	0.83140
H	−2.48480	2.07670	2.85440
H	0.19060	3.04200	2.26070
H	0.36390	1.81970	3.51630
H	1.28830	−0.53690	2.74970
H	2.44750	1.51900	2.38750
H	1.64380	−0.64950	−0.29320
H	2.95090	−0.90370	0.84950
H	0.37520	−2.57230	0.59120
H	−2.90190	−0.76690	1.73240
H	−4.10960	0.18450	2.57250
H	−4.16350	1.88000	0.70420
H	−3.18180	−0.69350	−0.68240
H	−3.12160	0.49240	−3.63380
H	−3.97770	−0.88920	−2.89640
H	−4.90750	0.38310	−3.69660
H	0.90510	−3.34420	2.74040
H	1.85200	−2.80510	−1.08800
**Marilzafurollene-A_29**			
C	−0.06510	1.31070	1.34290
O	0.55300	0.08830	0.88700
C	−1.59830	1.24760	1.27000
C	0.53610	1.63590	2.70900
C	1.61760	−0.24490	1.80630
C	1.91960	1.02670	2.60490
C	2.78500	−0.87290	1.04790
Cl	3.02450	2.16060	1.76740
C	2.43850	−2.24920	0.45770
C	−2.08270	0.99640	−0.16880
Br	−2.29530	−0.19340	2.42680

C	−3.56550	1.21900	−0.35340
C	−4.40280	0.26370	−0.78670
C	−5.86800	0.44150	−0.98930
C	−6.51120	1.76630	−0.67340
O	−6.52390	−0.50910	−1.41690
C	2.19150	−3.34630	1.45280
O	3.52230	−2.68960	−0.36280
C	2.71650	−3.44930	2.64140
C	3.24300	−3.54780	3.82040
H	4.16170	−4.07520	4.03990
Br	2.35560	−2.76090	5.23010
H	0.26410	2.08630	0.63840
H	−2.02870	2.17150	1.67250
H	0.52670	2.70890	2.92560
H	−0.00490	1.13800	3.52020
H	1.18870	−0.97600	2.50190
H	2.36540	0.80860	3.57920
H	3.07770	−0.22500	0.21160
H	3.66720	−0.95300	1.69530
H	1.55770	−2.16470	−0.18960
H	−1.57350	1.69540	−0.84470
H	−1.80160	−0.01200	−0.49890
H	−3.92630	2.21900	−0.12600
H	−4.04340	−0.73460	−1.02110
H	−7.58770	1.69740	−0.85750
H	−6.36110	2.01680	0.38000
H	−6.10170	2.54680	−1.31980
H	1.59040	−4.17660	1.09220
H	3.61620	−2.03840	−1.07950
**Marilzafurollene-A_30**			
C	−0.85670	1.14870	1.77190
O	−0.26920	−0.13340	1.46220
C	−2.15670	1.00930	2.57920
C	0.24110	2.00090	2.40770
C	1.15440	−0.03960	1.68540
C	1.49370	1.45310	1.75370
C	1.91580	−0.87680	0.66130
Cl	1.73090	2.22270	0.15480
C	1.54850	−2.36620	0.69450
C	−3.24590	0.19950	1.85610
Br	−1.81890	0.10290	4.30400
C	−3.77900	0.88700	0.62380
C	−3.66860	0.36920	−0.60950
C	−4.19490	1.00220	−1.85150
C	−4.89690	2.33240	−1.77250
O	−4.04190	0.41510	−2.92300
C	1.80660	−3.03440	2.01680

O	2.30360	−3.02790	−0.32310
C	2.97500	−3.36180	2.49280
C	4.13860	−3.67780	2.96520
H	4.62310	−4.63380	2.81900
Br	5.07150	−2.38760	3.89280
H	−1.10610	1.60080	0.80360
H	−2.53700	1.99910	2.85650
H	0.07950	3.07280	2.25510
H	0.31010	1.83560	3.48770
H	1.32570	−0.45550	2.68680
H	2.39850	1.64580	2.33660
H	1.70060	−0.52320	−0.35500
H	2.99700	−0.75300	0.80410
H	0.49120	−2.49280	0.43510
H	−2.87320	−0.80310	1.60880
H	−4.09980	0.04360	2.52880
H	−4.28830	1.83290	0.79190
H	−3.16750	−0.58060	−0.77720
H	−5.79760	2.24670	−1.15930
H	−4.22250	3.09100	−1.36720
H	−5.19510	2.64290	−2.77860
H	0.94700	−3.12050	2.67740
H	2.15560	−3.98260	−0.20890
**Marilzafurollene-A_31**			
C	−0.83590	0.90560	1.84960
O	−0.26670	−0.35810	1.44490
C	−2.15160	0.72730	2.62320
C	0.26710	1.68420	2.56530
C	1.15590	−0.31090	1.69640
C	1.51920	1.16470	1.88870
C	1.90510	−1.06500	0.59990
Cl	1.79680	2.04830	0.35590
C	1.63010	−2.57700	0.62380
C	−3.23740	−0.01700	1.82810
Br	−1.85700	−0.29870	4.28730
C	−3.72750	0.75020	0.62530
C	−3.58640	0.30500	−0.63290
C	−4.06630	1.01700	−1.85040
C	−4.77280	2.34000	−1.71270
O	−3.87040	0.50030	−2.95080
C	2.15770	−3.32070	1.81700
O	2.23560	−3.17830	−0.52230
C	3.20860	−3.01030	2.52300
C	4.25530	−2.69860	3.21870
H	5.25420	−3.06390	3.02120
Br	4.02960	−1.57430	4.66060
H	−1.06230	1.43820	0.91720

H	−2.52500	1.70070	2.96110
H	0.12770	2.76760	2.49420
H	0.31700	1.43440	3.63000
H	1.30120	−0.81320	2.66020
H	2.41860	1.29370	2.49710
H	1.59500	−0.69400	−0.38560
H	2.98390	−0.87800	0.67080
H	0.55150	−2.75960	0.55160
H	−2.87690	−1.01070	1.53110
H	−4.10980	−0.19570	2.47060
H	−4.22990	1.69130	0.83530
H	−3.09050	−0.63940	−0.84160
H	−5.68790	2.21920	−1.12730
H	−4.10960	3.07440	−1.24850
H	−5.04790	2.70690	−2.70630
H	1.66060	−4.26140	2.03740
H	1.81760	−2.77830	−1.30450
**Marilzafurollene-A_32**			
C	−0.28890	1.18240	1.15430
O	0.40930	−0.06220	0.93670
C	−1.81030	0.98550	1.23990
C	0.37590	1.86710	2.34720
C	1.57180	−0.07830	1.79360
C	1.80320	1.36630	2.24990
C	2.73720	−0.78220	1.10380
Cl	2.71820	2.36370	1.07760
C	2.43420	−2.23800	0.72500
C	−2.36950	0.36500	−0.05300
Br	−2.27190	−0.19160	2.75870
C	−3.86950	0.47580	−0.17310
C	−4.49250	1.18690	−1.12620
C	−5.98270	1.24160	−1.19260
C	−6.58740	2.03650	−2.32020
O	−6.68640	0.68460	−0.35200
C	2.08500	−3.11760	1.89370
O	3.58460	−2.76820	0.06250
C	2.92120	−3.57600	2.78230
C	3.75400	−4.02240	3.66770
H	4.26970	−4.97110	3.60440
Br	4.14050	−2.94170	5.10930
H	−0.08740	1.79240	0.26340
H	−2.29770	1.94230	1.45860
H	0.27270	2.95640	2.31540
H	−0.04860	1.52990	3.29830
H	1.27900	−0.65450	2.68090
H	2.34040	1.41960	3.20080
H	2.99800	−0.26350	0.17270

H	3.63180	−0.73730	1.73820
H	1.61170	−2.26910	0.00130
H	−1.90880	0.86370	−0.91580
H	−2.09480	−0.69460	−0.12890
H	−4.46240	−0.07720	0.55510
H	−3.94360	1.74090	−1.87880
H	−6.91050	3.01000	−1.94320
H	−5.86930	2.18270	−3.13160
H	−7.44380	1.48980	−2.72480
H	1.02220	−3.25980	2.07580
H	3.43070	−3.72210	−0.05110
**Marilzafurollene-A_33**			
C	−0.86310	0.93300	1.81400
O	−0.31720	−0.34210	1.41450
C	−2.16500	0.77890	2.61560
C	0.26230	1.70490	2.50120
C	1.11000	−0.30980	1.63490
C	1.49540	1.16280	1.80760
C	1.83340	−1.08490	0.53500
Cl	1.76000	2.02990	0.26370
C	1.52670	−2.59060	0.56260
C	−3.27890	0.05060	1.84510
Br	−1.85160	−0.24680	4.27670
C	−3.79390	0.83430	0.66340
C	−3.69910	0.39820	−0.60230
C	−4.20780	1.12930	−1.79680
C	−4.86740	2.47250	−1.62540
O	−4.07550	0.61180	−2.90620
C	2.00840	−3.33440	1.77620
O	2.12910	−3.20510	−0.57850
C	3.11670	−3.11510	2.42630
C	4.21220	−2.88830	3.07870
H	5.12740	−3.45330	2.96100
Br	4.17890	−1.59310	4.38920
H	−1.10060	1.45800	0.88020
H	−2.51530	1.75920	2.95830
H	0.13510	2.78920	2.42150
H	0.32820	1.46520	3.56730
H	1.26980	−0.80420	2.60070
H	2.40640	1.28500	2.39990
H	1.52640	−0.71370	−0.45140
H	2.91640	−0.92010	0.60010
H	0.44580	−2.74200	0.46550
H	−2.93620	−0.94370	1.53010
H	−4.13560	−0.12480	2.50930
H	−4.27610	1.78050	0.89670
H	−3.22510	−0.55180	−0.83520

H	−4.16940	3.17990	−1.17020
H	−5.15520	2.86060	−2.60730
H	−5.77060	2.37340	−1.01790
H	1.38530	−4.16590	2.09540
H	3.09300	−3.14090	−0.46170
**Marilzafurollene-A_34**			
C	−0.78430	0.90940	1.53400
O	−0.07180	−0.32120	1.28490
C	−2.18210	0.65740	2.12050
C	0.14250	1.80230	2.35880
C	1.29220	−0.14740	1.72550
C	1.51520	1.35920	1.89360
C	2.25230	−0.89110	0.80110
Cl	1.94860	2.20730	0.37810
C	1.99120	−2.40180	0.73660
C	−3.09260	−0.20090	1.22500
Br	−2.05170	−0.27820	3.86040
C	−3.46280	0.46970	−0.07150
C	−4.70170	0.89330	−0.36730
C	−4.99640	1.53730	−1.68080
C	−6.42990	1.91080	−1.95260
O	−4.10520	1.77650	−2.49380
C	2.10070	−3.10290	2.06240
O	2.93320	−2.97260	−0.17480
C	3.20510	−3.32540	2.71810
C	4.30470	−3.53090	3.37020
H	4.89210	−4.43760	3.31680
Br	4.95620	−2.15710	4.41150
H	−0.90780	1.38360	0.55260
H	−2.66930	1.61030	2.35650
H	−0.06520	2.86730	2.21400
H	0.05570	1.59960	3.43100
H	1.33930	−0.59390	2.72720
H	2.30330	1.58210	2.61820
H	2.16220	−0.51300	−0.22510
H	3.28980	−0.70150	1.10540
H	0.99520	−2.58820	0.31890
H	−2.61120	−1.15940	0.99250
H	−4.01090	−0.45500	1.77170
H	−2.66750	0.58260	−0.80820
H	−5.52680	0.78300	0.32690
H	−7.11360	1.34970	−1.30970
H	−6.56400	2.98110	−1.77750
H	−6.67230	1.66920	−2.99140
H	1.15970	−3.30300	2.56950
H	2.84310	−3.93880	−0.10600

**Marilzafurollene-A_35**			
C	−0.75120	0.68380	1.57930
O	−0.03630	−0.52540	1.24580
C	−2.15370	0.39120	2.13450
C	0.16920	1.51550	2.47210
C	1.32280	−0.38840	1.71780
C	1.54500	1.10250	1.98970
C	2.28440	−1.05930	0.73940
Cl	1.99180	2.04500	0.53400
C	2.11510	−2.58620	0.69290
C	−3.05490	−0.40370	1.17350
Br	−2.03980	−0.66000	3.80750
C	−3.40630	0.35130	−0.08130
C	−4.63970	0.79880	−0.36410
C	−4.91530	1.52770	−1.63670
C	−6.34370	1.92560	−1.90100
O	−4.01300	1.81400	−2.42190
C	2.50210	−3.32740	1.94040
O	2.93150	−3.11310	−0.35490
C	3.40490	−2.96760	2.80910
C	4.30490	−2.60670	3.66730
H	5.34580	−2.89820	3.62390
Br	3.77680	−1.54300	5.07580
H	−0.86750	1.22780	0.63390
H	−2.64400	1.32570	2.43000
H	−0.03510	2.58850	2.39890
H	0.07240	1.23980	3.52700
H	1.35260	−0.90640	2.68380
H	2.32810	1.27560	2.73300
H	2.10630	−0.68170	−0.27580
H	3.32330	−0.80330	0.98250
H	1.07620	−2.83770	0.44940
H	−2.57290	−1.34660	0.88490
H	−3.98060	−0.68990	1.69090
H	−2.60150	0.50800	−0.79930
H	−5.47390	0.64760	0.31140
H	−7.03770	1.32760	−1.30420
H	−6.47520	2.98300	−1.65820
H	−6.57430	1.75340	−2.95610
H	2.04500	−4.30560	2.06220
H	2.61200	−2.71980	−1.18550
**Marilzafurollene-A_36**			
C	−0.29370	1.00080	1.18950
O	0.39200	−0.22320	0.85210
C	−1.81560	0.80780	1.27420
C	0.39000	1.57050	2.43080
C	1.56700	−0.32440	1.68640

C	1.81150	1.07020	2.27200
C	2.71870	−0.95260	0.90360
Cl	2.72580	2.16210	1.18640
C	2.45270	−2.41630	0.51800
C	−2.39280	0.30330	−0.06060
Br	−2.27260	−0.48950	2.69290
C	−3.89270	0.43750	−0.15590
C	−4.51730	1.23000	−1.04130
C	−6.00730	1.30370	−1.08850
C	−6.61450	2.19040	−2.14390
O	−6.70900	0.68820	−0.28780
C	2.34450	−3.38430	1.66210
O	3.51350	−2.87070	−0.32530
C	3.04410	−3.36010	2.76180
C	3.73070	−3.32760	3.85940
H	4.60090	−3.93860	4.05980
Br	3.11300	−2.25850	5.22740
H	−0.09720	1.68730	0.35490
H	−2.29290	1.74620	1.57840
H	0.29560	2.65900	2.49830
H	−0.02600	1.15230	3.35290
H	1.28310	−0.98210	2.51690
H	2.35880	1.03180	3.21790
H	2.88410	−0.40070	−0.03080
H	3.65380	−0.87700	1.47280
H	1.53420	−2.47320	−0.07670
H	−1.93500	0.86850	−0.88300
H	−2.12930	−0.74870	−0.22790
H	−4.48420	−0.16800	0.53050
H	−3.96950	1.83850	−1.75160
H	−6.93730	3.12840	−1.68550
H	−5.89820	2.40560	−2.94150
H	−7.47150	1.67910	−2.59150
H	1.64490	−4.20390	1.51980
H	4.31830	−2.90260	0.22080
**Marilzafurollene-A_37**			
C	−0.78030	0.72200	1.54360
O	−0.08800	−0.49900	1.20770
C	−2.17630	0.45190	2.12590
C	0.16640	1.54640	2.41520
C	1.28180	−0.37650	1.64830
C	1.52820	1.11250	1.91200
C	2.21990	−1.06390	0.65780
Cl	1.96660	2.04660	0.44880
C	2.01000	−2.58480	0.58940
C	−3.10650	−0.33410	1.18540
Br	−2.04510	−0.59420	3.80130

C	−3.48200	0.42730	−0.05870
C	−4.71870	0.88360	−0.31140
C	−5.01930	1.61920	−1.57440
C	−6.45230	2.01960	−1.80800
O	−4.13290	1.90870	−2.37640
C	2.33350	−3.34860	1.84210
O	2.82850	−3.11660	−0.45490
C	3.30750	−3.09660	2.67100
C	4.26890	−2.84160	3.50050
H	5.22850	−3.34140	3.50330
Br	3.94240	−1.62790	4.84800
H	−0.90560	1.26080	0.59640
H	−2.64780	1.39440	2.42690
H	−0.02520	2.62160	2.33960
H	0.08420	1.27780	3.47320
H	1.32700	−0.89100	2.61600
H	2.32480	1.27640	2.64290
H	2.05140	−0.67290	−0.35390
H	3.26560	−0.83820	0.90230
H	0.97140	−2.79500	0.31020
H	−2.63930	−1.28020	0.88350
H	−4.02220	−0.61380	1.72390
H	−2.69280	0.58120	−0.79450
H	−5.53800	0.73480	0.38260
H	−6.70640	1.84560	−2.85740
H	−7.13440	1.42470	−1.19460
H	−6.57610	3.07780	−1.56460
H	1.72550	−4.23130	2.02330
H	3.75440	−2.99900	−0.17940
**Marilzafurollene-A_38**			
C	−0.09270	2.13750	1.71690
O	0.38050	0.89040	1.16750
C	−1.48090	2.02480	2.36510
C	1.00870	2.66080	2.64080
C	1.74710	0.70010	1.59020
C	2.26180	2.07440	2.02460
C	2.54920	−0.03290	0.51770
Cl	2.81360	3.10510	0.66940
C	1.98350	−1.41720	0.17990
C	−2.64050	1.78570	1.37970
Br	−1.51990	0.63590	3.76200
C	−2.60090	0.51420	0.56910
C	−3.54470	−0.43670	0.64680
C	−3.54590	−1.70140	−0.13960
C	−2.39120	−2.01560	−1.05340
O	−4.50680	−2.46340	−0.02680
C	2.01760	−2.37480	1.33300

O	2.78960	−1.97910	−0.85710
C	0.96410	−2.86060	1.92540
C	−0.08960	−3.32960	2.51410
H	−0.57600	−2.86760	3.36320
Br	−0.88760	−4.82380	1.79040
H	−0.17710	2.82630	0.86600
H	−1.69100	2.95370	2.90900
H	1.00700	3.75300	2.71590
H	0.89890	2.26800	3.65750
H	1.69820	0.07360	2.49020
H	3.09270	2.00300	2.73170
H	2.55260	0.54310	−0.41620
H	3.59880	−0.12040	0.82680
H	0.96760	−1.31640	−0.21910
H	−3.58760	1.83850	1.93380
H	−2.66780	2.62320	0.67040
H	−1.75920	0.40870	−0.11060
H	−4.39490	−0.33310	1.31490
H	−1.45460	−2.01930	−0.49070
H	−2.53190	−3.01210	−1.48290
H	−2.34960	−1.28960	−1.86920
H	2.99810	−2.67370	1.69370
H	2.39170	−2.83910	−1.08180
**Marilzafurollene-A_39**			
C	−0.43480	1.09770	1.40750
O	0.39560	−0.06080	1.17970
C	−1.68440	0.75430	2.23340
C	0.46790	2.19620	1.97010
C	1.76540	0.31080	1.44300
C	1.81900	1.84020	1.38240
C	2.71000	−0.45230	0.51760
Cl	1.95490	2.49430	−0.27860
C	2.64730	−1.97340	0.71040
C	−2.57880	−0.32630	1.60030
Br	−1.17570	0.08700	4.02730
C	−3.18580	0.08310	0.28140
C	−4.48740	0.37260	0.12980
C	−5.13220	0.75650	−1.15780
C	−4.31210	0.80590	−2.42000
O	−6.33320	1.02820	−1.15600
C	3.15290	−2.42360	2.04740
O	3.49470	−2.58580	−0.26410
C	2.40500	−2.87080	3.01620
C	1.65180	−3.30620	3.97540
H	1.11090	−2.67360	4.66650
Br	1.39660	−5.12510	4.11710
H	−0.76390	1.42400	0.41320

H	−2.26620	1.66170	2.43120
H	0.11170	3.19960	1.71580
H	0.53770	2.14810	3.06140
H	1.96330	0.02000	2.48240
H	2.65510	2.24950	1.95590
H	2.45440	−0.25240	−0.53060
H	3.74030	−0.10000	0.65540
H	1.63030	−2.34580	0.53890
H	−2.01090	−1.25230	1.44240
H	−3.38340	−0.59030	2.30030
H	−2.50590	0.10200	−0.56700
H	−5.17330	0.34130	0.97200
H	−4.95710	1.08640	−3.25830
H	−3.52390	1.55750	−2.32740
H	−3.88630	−0.17830	−2.63160
H	4.22730	−2.38120	2.20190
H	3.07000	−2.44750	−1.12870
**Marilzafurollene-A_**			
C	−0.24920	0.97080	1.20680
O	0.45100	−0.25060	0.88690
C	−1.77060	0.76640	1.27580
C	0.41470	1.55740	2.45130
C	1.60640	−0.34320	1.75070
C	1.83970	1.05930	2.32050
C	2.77150	−0.98060	0.99690
Cl	2.76560	2.13710	1.22980
C	2.52070	−2.45810	0.65570
C	−2.32890	0.24930	−0.06220
Br	−2.23310	−0.52560	2.69740
C	−3.82890	0.36780	−0.17420
C	−4.45250	1.15010	−1.06930
C	−5.94270	1.20730	−1.13220
C	−6.54860	2.08170	−2.19850
O	−6.64580	0.58830	−0.33540
C	2.45970	−3.39930	1.82420
O	3.57830	−2.92820	−0.18220
C	3.08260	−3.26640	2.96150
C	3.70580	−3.13030	4.08840
H	4.68540	−3.53730	4.30070
Br	2.85830	−2.20430	5.43670
H	−0.04850	1.65190	0.36890
H	−2.25860	1.70290	1.56870
H	0.31650	2.64640	2.50390
H	−0.01460	1.14990	3.37220
H	1.30210	−0.98970	2.58250
H	2.37400	1.03610	3.27420
H	2.93640	−0.45140	0.04940

H	3.70250	−0.87980	1.56880
H	1.58980	−2.55470	0.08470
H	−1.86810	0.81480	−0.88270
H	−2.05310	−0.80090	−0.22120
H	−4.42140	−0.24120	0.50840
H	−3.90400	1.76150	−1.77640
H	−5.82660	2.30020	−2.98990
H	−7.39550	1.55880	−2.65180
H	−6.88600	3.01870	−1.74850
H	1.91580	−4.32390	1.65080
H	3.55270	−2.39410	−0.99490
**Marilzafurollene-A_41**			
C	−0.51940	1.95130	1.94480
O	−0.13220	0.60100	1.64070
C	−1.90450	2.04170	2.60000
C	0.65520	2.52520	2.72400
C	1.31140	0.52420	1.62850
C	1.83490	1.92820	1.97360
C	1.73930	−0.09290	0.29700
Cl	2.22710	2.94300	0.55000
C	1.31040	−1.56270	0.17920
C	−3.06680	1.65900	1.65620
Br	−2.00630	0.96290	4.23950
C	−2.98950	0.28710	1.03090
C	−2.53200	0.07490	−0.21370
C	−2.29760	−1.27630	−0.78830
C	−3.06470	−2.45690	−0.25530
O	−1.44360	−1.40020	−1.66970
C	2.25220	−2.48950	0.88810
O	1.32980	−1.93510	−1.19910
C	1.98060	−3.14680	1.97970
C	1.71200	−3.80140	3.06440
H	2.01300	−3.49610	4.05740
Br	0.76940	−5.37710	2.91370
H	−0.58610	2.47890	0.98340
H	−2.07140	3.06830	2.94650
H	0.64790	3.61880	2.76140
H	0.66970	2.15210	3.75390
H	1.58030	−0.14510	2.45460
H	2.74340	1.88540	2.58080
H	1.27090	0.45680	−0.53020
H	2.82430	−0.00660	0.15830
H	0.28870	−1.71240	0.54560
H	−4.02230	1.73250	2.19140
H	−3.11850	2.41350	0.85950
H	−3.25950	−0.54540	1.67620
H	−2.19170	0.89400	−0.83950

H	−4.10140	−2.18090	−0.04460
H	−3.07960	−3.24590	−1.01300
H	−2.57980	−2.83390	0.64860
H	3.22080	−2.64380	0.42050
H	0.44090	−1.70480	−1.55030
**Marilzafurollene-A_42**			
C	−0.75340	0.78330	1.56940
O	−0.11360	−0.46590	1.23470
C	−2.13430	0.57070	2.20810
C	0.25340	1.59910	2.37940
C	1.27620	−0.37680	1.61820
C	1.57890	1.11000	1.83140
C	2.15180	−1.11880	0.61000
Cl	1.98080	1.99200	0.32560
C	1.89150	−2.63330	0.58960
C	−3.11210	−0.23860	1.33760
Br	−1.97200	−0.39890	3.92570
C	−3.46270	0.43030	0.03190
C	−4.66320	0.97620	−0.21610
C	−5.05770	1.62280	−1.49950
C	−4.08490	1.66060	−2.64860
O	−6.18220	2.11660	−1.58790
C	2.24290	−3.37640	1.84720
O	2.64790	−3.21600	−0.47410
C	3.25600	−3.13370	2.63060
C	4.25650	−2.88730	3.41520
H	5.19910	−3.41830	3.39640
Br	4.01950	−1.62990	4.74120
H	−0.90100	1.30310	0.61450
H	−2.57590	1.53670	2.47800
H	0.09150	2.67750	2.28340
H	0.20920	1.36020	3.44650
H	1.34390	−0.86770	2.59670
H	2.41100	1.26770	2.52310
H	1.95540	−0.74780	−0.40420
H	3.21300	−0.92110	0.80730
H	0.83600	−2.81620	0.35890
H	−2.69730	−1.22970	1.11240
H	−4.03400	−0.42760	1.90450
H	−2.68490	0.41940	−0.72820
H	−5.44790	0.97690	0.53560
H	−3.19790	2.23620	−2.37170
H	−3.81090	0.64470	−2.94440
H	−4.55800	2.14980	−3.50560
H	1.61670	−4.23540	2.07500
H	3.58750	−3.13120	−0.23570

**Marilzafurollene-A_43**			
C	−0.75410	0.97750	1.53250
O	−0.08360	−0.27800	1.29170
C	−2.13590	0.77900	2.17400
C	0.22640	1.87000	2.29300
C	1.29850	−0.13020	1.68360
C	1.56830	1.37440	1.79160
C	2.20740	−0.93220	0.75600
Cl	1.96220	2.15910	0.23120
C	1.89930	−2.43540	0.74810
C	−3.09140	−0.10080	1.34860
Br	−1.96760	−0.08050	3.94890
C	−3.45290	0.48600	0.00680
C	−4.66570	0.98710	−0.27370
C	−5.07170	1.55270	−1.59140
C	−4.09440	1.55660	−2.73730
O	−6.20930	2.00890	−1.71020
C	2.03550	−3.09810	2.09100
O	2.78880	−3.06130	−0.17950
C	3.15610	−3.38010	2.69420
C	4.27160	−3.65080	3.29350
H	4.80560	−4.58750	3.20620
Br	5.04360	−2.32690	4.31670
H	−0.90330	1.42620	0.54250
H	−2.59750	1.75120	2.38110
H	0.04160	2.93520	2.12110
H	0.17570	1.70420	3.37380
H	1.36550	−0.54430	2.69810
H	2.38980	1.59970	2.47720
H	2.09800	−0.58380	−0.27870
H	3.25870	−0.76560	1.02390
H	0.88340	−2.60350	0.37260
H	−2.65270	−1.09290	1.18030
H	−4.01080	−0.27920	1.92290
H	−2.67230	0.45340	−0.74980
H	−5.45310	1.00910	0.47490
H	−3.22340	2.16760	−2.48680
H	−3.79380	0.53400	−2.97980
H	−4.57530	1.98960	−3.61980
H	1.11210	−3.22100	2.65220
H	2.67580	−4.02200	−0.07540
**Marilzafurollene-A_44**			
C	−0.37230	1.92640	1.98460
O	0.12040	0.71620	1.37410
C	−1.56480	1.68420	2.92230
C	0.82990	2.61310	2.63450
C	1.55960	0.71190	1.47150

C	1.98120	2.15420	1.76350
C	2.18290	0.03940	0.25070
Cl	2.09430	3.19000	0.30850
C	1.71750	−1.40820	0.05390
C	−2.86790	1.26860	2.21340
Br	−1.13130	0.36260	4.31970
C	−2.81020	0.02460	1.36300
C	−3.04520	0.02120	0.04180
C	−2.98940	−1.18800	−0.82590
C	−2.64270	−2.52480	−0.22590
O	−3.23630	−1.06390	−2.02610
C	2.11480	−2.32260	1.17370
O	2.33500	−1.90900	−1.13320
C	1.28600	−2.87100	2.01600
C	0.45800	−3.40140	2.85860
H	0.19900	−2.97260	3.81770
Br	−0.37670	−4.97180	2.37900
H	−0.72030	2.56030	1.15830
H	−1.76760	2.60300	3.48550
H	0.70910	3.69960	2.69360
H	0.99460	2.25100	3.65520
H	1.79690	0.11930	2.36450
H	2.94830	2.20940	2.27050
H	1.91300	0.58310	−0.66330
H	3.27780	0.07840	0.31720
H	0.63400	−1.43190	−0.10990
H	−3.65640	1.11870	2.96270
H	−3.20070	2.11560	1.59880
H	−2.56430	−0.89650	1.88510
H	−3.29110	0.93520	−0.49060
H	−1.64940	−2.48710	0.22700
H	−3.39480	−2.81280	0.51290
H	−2.62850	−3.28220	−1.01550
H	3.17590	−2.53090	1.28240
H	1.96390	−2.79670	−1.28380
**Marilzafurollene-A_45**			
C	−0.57800	1.65920	2.16130
O	−0.42730	0.30670	1.69900
C	−2.00610	1.97440	2.62700
C	0.54230	1.85140	3.17350
C	0.95040	−0.09450	1.88100
C	1.68420	1.10350	2.50380
C	1.46170	−0.63360	0.54490
Cl	2.49620	2.17190	1.31550
C	0.79550	−1.97110	0.17830
C	−3.04510	1.98540	1.48280
Br	−2.59900	0.74600	4.04160

C	−3.14810	0.71860	0.66790
C	−2.53000	0.55580	−0.51320
C	−2.48590	−0.73580	−1.24770
C	−3.58170	−1.74760	−1.04180
O	−1.52290	−0.96100	−1.98470
C	1.34300	−3.19400	0.85850
O	0.94330	−2.19010	−1.22540
C	2.39560	−3.27110	1.62250
C	3.44620	−3.35430	2.37520
H	4.42520	−3.65900	2.02980
Br	3.25690	−2.97740	4.16830
H	−0.37550	2.30630	1.29680
H	−2.01790	2.95980	3.10730
H	0.75130	2.90500	3.38220
H	0.30890	1.35840	4.12360
H	0.92490	−0.89680	2.62760
H	2.45510	0.78620	3.21150
H	1.22820	0.08390	−0.25300
H	2.55270	−0.74220	0.55430
H	−0.27980	−1.93240	0.38700
H	−4.04150	2.20930	1.88530
H	−2.80440	2.82290	0.81410
H	−3.69890	−0.09870	1.12740
H	−1.91660	1.34030	−0.94510
H	−3.62430	−2.41110	−1.91060
H	−3.37450	−2.33820	−0.14600
H	−4.55300	−1.25310	−0.95530
H	0.80310	−4.11440	0.65150
H	0.21560	−1.68720	−1.65420
**Marilzafurollene-A_46**			
C	0.03250	1.43510	0.78790
O	0.87100	0.26100	0.77000
C	−1.45780	1.07900	0.92500
C	0.60200	2.37890	1.84620
C	1.99860	0.50210	1.63840
C	2.07420	2.01810	1.84310
C	3.24910	−0.18920	1.10010
Cl	2.91240	2.88870	0.52270
C	3.09770	−1.71180	0.99420
C	−1.93300	0.20020	−0.24770
Br	−1.77760	0.09670	2.61210
C	−3.41680	−0.00110	−0.45650
C	−4.43270	0.57460	0.20580
C	−5.84940	0.25030	−0.14140
C	−6.92170	0.89270	0.69930
O	−6.13040	−0.48540	−1.08600
C	2.92020	−2.38510	2.32240

O	4.29800	−2.23240	0.41990
C	1.84640	−3.02110	2.69570
C	0.77000	−3.64150	3.06110
H	−0.11960	−3.15570	3.43980
Br	0.70110	−5.46670	2.82060
H	0.16910	1.91030	−0.19280
H	−2.04700	1.99860	1.00040
H	0.39080	3.43010	1.62560
H	0.19170	2.17010	2.83950
H	1.73190	0.05810	2.60610
H	2.58110	2.28640	2.77410
H	3.48220	0.17720	0.09240
H	4.11630	0.05970	1.72520
H	2.27640	−1.95990	0.31150
H	−1.55550	0.63560	−1.18250
H	−1.47630	−0.79560	−0.17010
H	−3.65490	−0.70460	−1.25650
H	−4.28480	1.28380	1.01000
H	−7.31490	1.76830	0.17700
H	−7.72410	0.16950	0.87080
H	−6.53040	1.19560	1.67440
H	3.75600	−2.33080	3.01470
H	4.15290	−3.18810	0.30090
**Marilzafurollene-A_47**			
C	−0.72060	0.73100	1.57790
O	−0.04690	−0.49990	1.23810
C	−2.10950	0.48010	2.18520
C	0.25050	1.55710	2.42110
C	1.32650	−0.39470	1.67610
C	1.59890	1.09470	1.90690
C	2.24490	−1.11620	0.69210
Cl	2.02510	1.99070	0.41590
C	2.03300	−2.63850	0.68840
C	−3.05450	−0.32970	1.27980
Br	−1.96190	−0.52020	3.88640
C	−3.40110	0.36460	−0.01380
C	−4.61150	0.88580	−0.26700
C	−5.00280	1.55670	−1.53890
C	−4.01180	1.65550	−2.66860
O	−6.13990	2.01970	−1.63400
C	2.42730	−3.35880	1.94560
O	2.81070	−3.21400	−0.36310
C	3.35880	−3.00260	2.78500
C	4.28690	−2.64520	3.61420
H	5.31710	−2.97130	3.55970
Br	3.81980	−1.52850	5.00300
H	−0.85930	1.26150	0.62770

H	−2.57460	1.43220	2.46520
H	0.07320	2.63330	2.32820
H	0.18010	1.31000	3.48510
H	1.36350	−0.89100	2.65310
H	2.40930	1.26220	2.62170
H	2.05250	−0.75880	−0.32780
H	3.29600	−0.88360	0.90390
H	0.98230	−2.86670	0.47420
H	−2.61280	−1.30460	1.03630
H	−3.98020	−0.55450	1.82690
H	−2.61150	0.39500	−0.76090
H	−5.40790	0.84550	0.47120
H	−3.14450	2.24370	−2.35790
H	−3.70730	0.65650	−2.99120
H	−4.48230	2.16020	−3.51790
H	1.94530	−4.31970	2.10350
H	2.48470	−2.83270	−1.19660
**Marilzafurollene-A_48**			
C	0.03040	1.43590	0.78920
O	0.87060	0.26280	0.76940
C	−1.45900	1.07740	0.92780
C	0.59980	2.38000	1.84730
C	1.99750	0.50440	1.63880
C	2.07240	2.02050	1.84310
C	3.24850	−0.18670	1.10170
Cl	2.90880	2.89080	0.52140
C	3.09750	−1.70930	0.99670
C	−1.93300	0.19590	−0.24400
Br	−1.77570	0.09280	2.61390
C	−3.41770	−0.02140	−0.44820
C	−4.42750	0.59290	0.18800
C	−5.87430	0.33060	−0.06390
C	−6.28430	−0.72490	−1.05690
O	−6.70770	0.99510	0.55300
C	2.92030	−2.38200	2.32530
O	4.29760	−2.23010	0.42210
C	1.84360	−3.01110	2.70210
C	0.76440	−3.62410	3.07170
H	−0.11640	−3.13320	3.46430
Br	0.68050	−5.44830	2.82880
H	0.16500	1.91170	−0.19140
H	−2.04860	1.99660	1.00390
H	0.38760	3.43120	1.62710
H	0.19030	2.17070	2.84080
H	1.73010	0.06070	2.60640
H	2.57980	2.28950	2.77360
H	3.48200	0.17920	0.09390

H	4.11530	0.06280	1.72710
H	2.27610	−1.95820	0.31440
H	−1.56180	0.63220	−1.18090
H	−1.46810	−0.79620	−0.16620
H	−3.63680	−0.76400	−1.21400
H	−4.25970	1.34670	0.94980
H	−5.94530	−0.45030	−2.05900
H	−5.88040	−1.69720	−0.76310
H	−7.37590	−0.80070	−1.07190
H	3.75860	−2.33280	3.01510
H	4.15420	−3.18640	0.30660
**Marilzafurollene-A_49**			
C	−0.34190	1.86710	1.98060
O	0.11520	0.64010	1.37640
C	−1.55580	1.66660	2.90090
C	0.87680	2.51210	2.64180
C	1.55230	0.58710	1.48260
C	2.01930	2.01440	1.77980
C	2.14930	−0.10050	0.25710
Cl	2.17970	3.04480	0.32500
C	1.64560	−1.53700	0.06440
C	−2.85540	1.27440	2.17160
Br	−1.18060	0.35120	4.32010
C	−2.81240	0.01140	1.34860
C	−2.95440	−0.00700	0.01440
C	−2.89040	−1.23430	−0.82670
C	−2.70730	−2.57970	−0.17590
O	−2.98750	−1.11590	−2.04890
C	2.07450	−2.47210	1.15400
O	2.20230	−2.05100	−1.14730
C	1.30240	−2.91240	2.10700
C	0.53230	−3.33460	3.05860
H	0.43750	−2.86160	4.02690
Br	−0.53160	−4.80100	2.72620
H	−0.65780	2.51130	1.14940
H	−1.74460	2.59720	3.44940
H	0.79280	3.60200	2.70050
H	1.02080	2.14390	3.66330
H	1.76660	−0.01560	2.37420
H	2.98380	2.03700	2.29450
H	1.88470	0.45210	−0.65300
H	3.24530	−0.09010	0.31440
H	0.55530	−1.55400	−0.04660
H	−3.66480	1.16280	2.90510
H	−3.15030	2.11710	1.53210
H	−2.64820	−0.90970	1.90220
H	−3.10950	0.90770	−0.54980

H	−3.52630	−2.77780	0.52030
H	−2.71800	−3.35750	−0.94550
H	−1.74400	−2.61890	0.33770
H	3.10840	−2.80480	1.13170
H	1.77420	−1.56870	−1.87650
	**Marilzafurollene-A_50**		
C	−0.19380	2.12080	1.85070
O	0.29590	0.91130	1.24220
C	−1.57110	1.93570	2.50540
C	0.91430	2.62410	2.77700
C	1.67210	0.72670	1.62370
C	2.16830	2.08900	2.11220
C	2.44740	0.05490	0.49330
Cl	2.67860	3.19080	0.79770
C	1.86460	−1.30890	0.09740
C	−2.73010	1.67190	1.52350
Br	−1.54260	0.49820	3.85630
C	−2.58130	0.49530	0.59490
C	−3.32340	−0.62030	0.66210
C	−3.10270	−1.74280	−0.29620
C	−3.89510	−3.00050	−0.05640
O	−2.33750	−1.63740	−1.25300
C	1.96820	−2.33620	1.18500
O	2.62020	−1.80400	−1.01010
C	0.95740	−2.83170	1.84040
C	−0.05220	−3.30600	2.49800
H	−0.52690	−2.80930	3.33360
Br	−0.77530	−4.90540	1.94060
H	−0.30860	2.84460	1.03310
H	−1.81490	2.83520	3.08320
H	0.89400	3.71160	2.90030
H	0.83130	2.18500	3.77700
H	1.65690	0.05690	2.49320
H	3.01240	2.00130	2.80160
H	2.42640	0.68060	−0.40780
H	3.50450	−0.04940	0.76910
H	0.82830	−1.19120	−0.24120
H	−3.66620	1.57710	2.09030
H	−2.85710	2.56350	0.89560
H	−1.81820	0.58160	−0.17880
H	−4.08900	−0.75560	1.41710
H	−3.32120	−3.86120	−0.41160
H	−4.08470	−3.14230	1.01120
H	−4.84320	−2.94170	−0.59650
H	2.96790	−2.67360	1.44450
H	2.12480	−2.57060	−1.35070

**Marilzafurollene-A_51**			
C	−0.62970	1.64320	2.02530
O	−0.05570	0.32530	2.00190
C	−1.97430	1.69370	2.76610
C	0.50880	2.53270	2.49630
C	1.33390	0.46310	1.61150
C	1.67300	1.96970	1.69410
C	1.52130	−0.22380	0.25430
Cl	1.77840	2.81160	0.11550
C	1.25480	−1.73470	0.30020
C	−2.96780	0.58930	2.34330
Br	−1.74210	1.54150	4.71710
C	−3.09300	0.41600	0.84780
C	−2.53020	−0.63030	0.22270
C	−2.44190	−0.81300	−1.24780
C	−3.39220	−0.07380	−2.15000
O	−1.55260	−1.54610	−1.68650
C	2.37950	−2.49520	0.93640
O	1.14460	−2.21740	−1.03970
C	2.33340	−3.06580	2.10680
C	2.28930	−3.63170	3.27080
H	2.66520	−3.19000	4.18370
Br	1.53190	−5.30670	3.39180
H	−0.84480	1.90170	0.97900
H	−2.42770	2.68170	2.62640
H	0.31610	3.59750	2.33610
H	0.72880	2.38090	3.55800
H	1.91330	−0.05990	2.38070
H	2.63460	2.13550	2.18840
H	0.82260	0.20680	−0.47510
H	2.53180	−0.03960	−0.13200
H	0.31150	−1.96080	0.80830
H	−2.67860	−0.36830	2.79820
H	−3.96290	0.81600	2.74750
H	−3.59380	1.20790	0.29750
H	−1.99500	−1.38750	0.78950
H	−3.42510	−0.57310	−3.12290
H	−4.40380	−0.08700	−1.73560
H	−3.04920	0.95490	−2.28460
H	3.28870	−2.59380	0.34980
H	0.23700	−1.99250	−1.34410
**Marilzafurollene-A_52**			
C	−0.03190	2.07510	1.68460
O	0.40530	0.80710	1.15320
C	−1.43570	2.02120	2.30670
C	1.07150	2.56650	2.62370
C	1.75630	0.57020	1.60160

C	2.31290	1.92840	2.03620
C	2.54740	−0.19290	0.54180
Cl	2.92810	2.92650	0.68320
C	1.94200	−1.55940	0.19690
C	−2.58330	1.79570	1.30410
Br	−1.54730	0.66930	3.73540
C	−2.57900	0.49650	0.53750
C	−3.56180	−0.41290	0.62660
C	−3.59640	−1.70500	−0.11330
C	−2.42270	−2.11160	−0.96420
O	−4.60000	−2.41200	−0.01550
C	1.95540	−2.52550	1.34160
O	2.72820	−2.15330	−0.83820
C	0.90430	−2.89890	2.01510
C	−0.14410	−3.25970	2.68390
H	−0.55820	−2.71240	3.52020
Br	−1.04840	−4.76450	2.12630
H	−0.07530	2.75850	0.82620
H	−1.62560	2.96920	2.82460
H	1.10880	3.65870	2.68870
H	0.93010	2.18830	3.64190
H	1.67080	−0.04990	2.50310
H	3.12750	1.83220	2.75910
H	2.58160	0.38240	−0.39190
H	3.58950	−0.31440	0.86440
H	0.92490	−1.44240	−0.19460
H	−3.53840	1.90400	1.83610
H	−2.56300	2.60950	0.56750
H	−1.72840	0.33300	−0.11900
H	−4.42230	−0.25120	1.26970
H	−1.50830	−2.12220	−0.36620
H	−2.58780	−3.12340	−1.34650
H	−2.32160	−1.43160	−1.81370
H	2.91880	−2.94870	1.61180
H	2.60590	−1.60590	−1.63320
**Marilzafurollene-A_53**			
C	−0.59390	1.25210	1.67480
O	0.12940	0.05460	1.31730
C	−1.71780	0.96370	2.68290
C	0.44230	2.29190	2.10090
C	1.54030	0.35330	1.35310
C	1.66380	1.87930	1.30440
C	2.28210	−0.43700	0.27630
Cl	1.57760	2.56120	−0.34820
C	2.16770	−1.95800	0.45460
C	−2.75810	−0.04620	2.17080
Br	−0.98030	0.22070	4.36250

C	−3.54900	0.46660	0.99720
C	−3.51720	−0.09370	−0.22200
C	−4.32570	0.46640	−1.34300
C	−4.23870	−0.24090	−2.66980
O	−5.01320	1.47590	−1.19740
C	2.84010	−2.46810	1.69600
O	2.78540	−2.60090	−0.66330
C	2.21420	−2.94620	2.73440
C	1.58100	−3.40980	3.76450
H	1.10110	−2.79730	4.51630
Br	1.36980	−5.23500	3.90050
H	−1.05290	1.60850	0.74420
H	−2.20570	1.89790	2.98320
H	0.10480	3.31700	1.91740
H	0.67860	2.21790	3.16720
H	1.89100	0.03130	2.34180
H	2.59960	2.23320	1.74580
H	1.87420	−0.20280	−0.71500
H	3.33860	−0.14030	0.25300
H	1.11350	−2.25740	0.44010
H	−2.27310	−0.99900	1.92110
H	−3.47660	−0.27610	2.96870
H	−4.18310	1.33430	1.17720
H	−2.90090	−0.95980	−0.43610
H	−3.58410	0.32430	−3.33800
H	−3.84730	−1.25510	−2.55380
H	−5.24040	−0.31500	−3.10250
H	3.92410	−2.42040	1.73390
H	3.70680	−2.29500	−0.71250
**Marilzafurollene-A_54**			
C	0.06250	1.34820	0.77320
O	0.88120	0.15960	0.76930
C	−1.43380	1.02050	0.91670
C	0.64990	2.29660	1.81770
C	2.00980	0.38980	1.63930
C	2.11470	1.90700	1.82200
C	3.24610	−0.33080	1.10710
Cl	2.97160	2.73930	0.48850
C	3.07520	−1.85430	1.03810
C	−1.92540	0.13170	−0.24150
Br	−1.76990	0.07190	2.61950
C	−3.41270	−0.04450	−0.44790
C	−4.41790	0.55870	0.20620
C	−5.84030	0.25630	−0.13770
C	−6.90130	0.92710	0.69510
O	−6.13440	−0.48520	−1.07380
C	2.94130	−2.49800	2.38490

O	4.24900	−2.41190	0.44340
C	1.83940	−3.00140	2.86480
C	0.73660	−3.49120	3.33470
H	−0.05000	−2.90470	3.79060
Br	0.42340	−5.29220	3.10600
H	0.20590	1.80770	−0.21400
H	−2.00650	1.95160	0.97770
H	0.45980	3.34830	1.58080
H	0.23480	2.11140	2.81370
H	1.73320	−0.03590	2.61210
H	2.62620	2.17930	2.74920
H	3.47360	0.01130	0.08970
H	4.12230	−0.08000	1.71890
H	2.22600	−2.12190	0.39820
H	−1.53960	0.54470	−1.18310
H	−1.48770	−0.87110	−0.14770
H	−3.66390	−0.75430	−1.23850
H	−4.25690	1.27540	1.00110
H	−7.29220	1.79240	0.15410
H	−7.70730	0.21420	0.89070
H	−6.50060	1.25210	1.65920
H	3.84110	−2.55250	2.99110
H	4.24180	−2.14230	−0.49170

Table S9. Calculated Energies (Hartrees) for diastereoisomer 4*S**,6*R**,7*R**,9*S**,10*S**,14*S** of **3**.

Entry ID	Gas Phase Energy
1	−1335,914446
2	−1335,916256
3	−1335,91134
4	−1335,91517
5	−1335,91346
6	−1335,913569
7	−1335,913417
8	−1335,915523
9	−1335,911506
10	−1335,915896
11	−1335,916525
12	−1335,91004
13	−1335,914391
14	−1335,910539
15	−1335,912527
16	−1335,914866
17	−1335,912661
18	−1335,910434
19	−1335,914381

20	−1335,909918
21	−1335,912263
22	−1335,914974
23	−1335,911609
24	−1335,912037
25	−1335,913897
26	−1335,912701
27	−1335,910775
28	−1335,911195
29	−1335,914683
30	−1335,911719
31	−1335,91515
32	−1335,911583
33	−1335,915222
34	−1335,915761
35	−1335,914861
36	−1335,910835
37	−1335,908888
38	−1335,912922
39	−1335,912555
40	−1335,914515
41	−1335,910589
42	−1335,91306
43	−1335,910704
44	−1335,914248
45	−1335,91281
46	−1335,909631
47	−1335,912855
48	−1335,906209
49	−1335,91171
50	−1335,913508
51	−1335,914507
52	−1335,911226
53	−1335,910697
54	−1335,908852
55	−1335,911472
56	−1335,912174
57	−1335,909609
58	−1335,911884
59	−1335,906562
60	−1335,911257
61	−1335,91464
62	−1335,914206
63	−1335,912782
64	−1335,910404
65	−1335,913251
66	−1335,906259

67	−1335,91363
68	−1335,91121
69	−1335,910968
70	−1335,913105
71	−1335,911797
72	−1335,912348
73	−1335,914211
74	−1335,912671
75	−1335,910164
76	−1335,908801
77	−1335,912049
78	−1335,913949
79	−1335,909965
80	−1335,913957
81	−1335,908301
82	−1335,910315
83	−1335,911187
84	−1335,913139
85	−1335,912342
86	−1335,909157
87	−1335,911956
88	−1335,913616
89	−1335,906279
90	−1335,914129
91	−1335,908341
92	−1335,914187
93	−1335,909781
94	−1335,913575
95	−1335,911571
96	−1335,913443
97	−1335,908821
98	−1335,907442
99	−1335,906272
100	−1335,909431
101	−1335,910797
102	−1335,910265
103	−1335,911906
104	−1335,914471
105	−1335,912234
106	−1335,914976
107	−1335,913506
108	−1335,909822
109	−1335,908804
110	−1335,915056
111	−1335,91099
112	−1335,909737
113	−1335,913668

114	−1335,911323
115	−1335,906175
116	−1335,906472
117	−1335,907731
118	−1335,91137
119	−1335,909504
120	−1335,910704
121	−1335,909266
122	−1335,910932
123	−1335,912148
124	−1335,911438
125	−1335,913825
126	−1335,90975
127	−1335,910236
128	−1335,91279
129	−1335,911724
130	−1335,910216
131	−1335,909309
132	−1335,912317
133	−1335,909104
134	−1335,907802
135	−1335,911302
136	−1335,909562
137	−1335,910724
138	−1335,910953
139	−1335,912951
140	−1335,910395
141	−1335,913709
142	−1335,915466
143	−1335,913115
144	−1335,907243
145	−1335,91047
146	−1335,913401
147	−1335,913185
148	−1335,913168
149	−1335,913169
150	−1335,907502
151	−1335,910645
152	−1335,912831
153	−1335,911961
154	−1335,911163
155	−1335,912695
156	−1335,913543
157	−1335,912967
158	−1335,912393
159	−1335,911443
160	−1335,911457

161	−1335,910828
162	−1335,910443
163	−1335,911635
164	−1335,91104
165	−1335,90902
166	−1335,913207
167	−1335,912907
168	−1335,913864
169	−1335,910721
170	−1335,908344
171	−1335,909066
172	−1335,911942
173	−1335,908846
174	−1335,909078
175	−1335,912925
176	−1335,910267
177	−1335,912499
178	−1335,911099
179	−1335,911123
180	−1335,911018
181	−1335,905061
182	−1335,908958

Table S10. Coordinates (Angstroms) of calculated geometries for diastereoisomer 4*S**,6*R**,7*R**,9*S**,10*S**,14*S** of **3**.

**Marilzafurollene C_2**			
C	2.91730	−1.56500	−1.12860
O	2.26350	−1.81540	0.11490
C	3.00560	−0.05030	−1.39630
C	2.14800	−2.36860	−2.18670
C	1.12820	−2.64820	−0.09490
C	3.89730	0.66540	−0.36770
Br	1.17760	0.75610	−1.34790
C	1.47080	−3.44290	−1.35900
C	0.78130	−3.46670	1.16460
C	4.09850	2.13280	−0.69310
Cl	2.62950	−4.76710	−1.03540
C	0.43640	−2.59320	2.38490
C	−0.77520	−1.71070	2.14860
O	0.18190	−3.44330	3.48440
C	−0.71270	−0.39870	2.11950
C	5.28820	2.70000	−0.95340
C	5.48680	4.16910	−1.27690
C	6.10410	4.34260	−2.66690
O	6.36670	4.78560	−0.34810
C	−0.62090	0.90910	2.07310
Br	−0.83080	2.04700	3.67910

C	5.81260	4.96760	0.94800
H	3.92370	−1.97930	−1.05040
H	3.40640	0.11520	−2.39760
H	1.38840	−1.75200	−2.66780
H	2.79150	−2.77000	−2.97000
H	0.28910	−1.99530	−0.34000
H	3.45740	0.58280	0.62710
H	4.86970	0.17360	−0.31750
H	0.59310	−3.87020	−1.84500
H	−0.05140	−4.13490	0.94260
H	1.62400	−4.10680	1.42650
H	3.20600	2.74250	−0.70810
H	1.30420	−1.97480	2.62740
H	−1.71640	−2.21890	1.99670
H	0.18840	−2.92180	4.27490
H	6.18540	2.09800	−0.93730
H	4.52310	4.68220	−1.26230
H	6.23300	5.39870	−2.90420
H	7.08340	3.86730	−2.72700
H	5.46910	3.90440	−3.43730
H	−0.42590	1.48130	1.17750
H	5.56420	4.01630	1.41990
H	6.53740	5.47380	1.58550
H	4.91350	5.58380	0.91040
	**Marilzafurollene C_1**		
C	2.94210	−1.27340	−1.05170
O	2.15660	−1.23740	0.14050
C	3.30180	0.15300	−1.50700
C	2.13830	−2.09410	−2.06880
C	0.94870	−1.97200	−0.04990
C	4.22300	0.86680	−0.50330
Br	1.63250	1.22570	−1.74000
C	1.26790	−2.95780	−1.17930
C	0.45180	−2.58410	1.27410
C	4.68890	2.22040	−1.00350
Cl	2.21170	−4.36380	−0.60220
C	0.04190	−1.52400	2.31140
C	−0.61140	−2.15540	3.52480
O	1.19910	−0.82770	2.71950
C	−1.84140	−1.89250	3.90340
C	5.97180	2.55120	−1.22580
C	6.43490	3.90740	−1.72460
C	7.16790	3.77510	−3.06190
O	7.33830	4.51050	−0.80970
C	−3.07530	−1.62950	4.26320
Br	−4.62070	−2.66940	3.59260
C	6.73340	4.96580	0.39300

H	3.85630	−1.82170	−0.81820
H	3.79580	0.10720	−2.47900
H	1.50370	−1.44870	−2.67650
H	2.76830	−2.67060	−2.74670
H	0.19950	−1.26930	−0.41970
H	3.70510	1.00290	0.44700
H	5.09390	0.24400	−0.29480
H	0.37650	−3.33170	−1.68430
H	−0.41030	−3.21630	1.05650
H	1.20940	−3.24170	1.70210
H	3.91360	2.95200	−1.18370
H	−0.6 40	−0.81110	1.85020
H	−0.00380	−2.85170	4.08420
H	1.74110	−0.71120	1.94240
H	6.75280	1.82650	−1.04500
H	5.57170	4.56020	−1.86890
H	7.48740	4.75150	−3.42650
H	8.05600	3.15010	−2.96620
H	6.52400	3.33160	−3.82170
H	−3.36530	−0.84130	4.94260
H	7.48610	5.44230	1.02090
H	5.95420	5.70150	0.18950
H	6.29770	4.14500	0.96370
**Marilzafurollene C_3**			
C	2.89580	−1.53350	−1.08610
O	2.24770	−1.77410	0.16270
C	2.98370	−0.02130	−1.36750
C	2.12140	−2.34490	−2.13420
C	1.11120	−2.60800	−0.03530
C	3.88410	0.70270	−0.35250
Br	1.15660	0.78690	−1.31290
C	1.44740	−3.41210	−1.29520
C	0.77940	−3.41940	1.23250
C	4.08310	2.16720	−0.69200
Cl	2.60640	−4.73470	−0.96550
C	0.40880	−2.53900	2.44060
C	−0.84320	−1.71440	2.20450
O	0.20550	−3.37380	3.56260
C	−0.83710	−0.40300	2.12440
C	5.27060	2.73150	−0.96810
C	5.46690	4.19780	−1.30560
C	6.07190	4.35930	−2.70240
O	6.35520	4.82150	−0.38980
C	−0.80520	0.90450	2.02090
Br	−1.02950	2.10360	3.58030
C	5.81240	5.01500	0.90940
H	3.90240	−1.94750	−1.00950

H	3.37710	0.13520	−2.37320
H	1.35990	−1.73160	−2.61660
H	2.76100	−2.75270	−2.91730
H	0.26910	−1.95830	−0.27860
H	3.45220	0.62910	0.64650
H	4.85660	0.21100	−0.30590
H	0.56730	−3.84240	−1.77420
H	−0.03840	−4.10790	1.01590
H	1.63690	−4.03750	1.49840
H	3.19070	2.77720	−0.70370
H	1.24420	−1.87090	2.66420
H	−1.76600	−2.26610	2.10180
H	1.05030	−3.62840	3.90640
H	6.16770	2.12920	−0.95530
H	4.50350	4.71140	−1.28690
H	6.19910	5.41340	−2.94970
H	7.05060	3.88320	−2.76720
H	5.43000	3.91500	−3.46350
H	−0.65700	1.44480	1.09710
H	5.56720	4.06800	1.39150
H	6.54300	5.52600	1.53640
H	4.91340	5.63170	0.87430
**Marilzafurollene C_4**			
C	2.97030	−1.57910	−1.36500
O	2.31690	−1.47270	−0.09960
C	3.63930	−0.24470	−1.74360
C	1.90750	−2.07430	−2.35480
C	0.95050	−1.86300	−0.22270
C	4.78320	0.12240	−0.78270
Br	2.27990	1.21900	−1.72550
C	0.91910	−2.78080	−1.44970
C	0.41900	−2.46060	1.09440
C	5.51970	1.37820	−1.20680
Cl	1.51780	−4.42540	−1.07870
C	0.37680	−1.43980	2.24510
C	−0.32000	−2.00580	3.46660
O	1.70190	−1.09670	2.58660
C	−1.40870	−1.48490	3.98490
C	5.63380	2.48260	−0.45050
C	6.35840	3.74580	−0.87620
C	7.52340	4.05190	0.06830
O	5.49210	4.87100	−0.85100
C	−2.50280	−0.96130	4.48480
Br	−4.30970	−1.51560	3.89540
C	4.48250	4.85470	−1.85170
H	3.73340	−2.35330	−1.26840
H	4.02600	−0.31290	−2.76150

H	1.41110	−1.23680	−2.84560
H	2.31560	−2.71870	−3.13380
H	0.37880	−0.96400	−0.46110
H	4.39260	0.24420	0.22880
H	5.50710	−0.69210	−0.73960
H	−0.07490	−2.87250	−1.88900
H	−0.59050	−2.83470	0.91760
H	1.01430	−3.32450	1.39270
H	5.97220	1.35850	−2.18780
H	−0.13850	−0.53650	1.91070
H	0.13110	−2.88230	3.90840
H	2.19230	−1.03720	1.76970
H	5.18200	2.50780	0.53100
H	6.75650	3.61760	−1.88470
H	8.05230	4.95120	−0.24770
H	7.17300	4.21430	1.08790
H	8.24220	3.23240	0.08590
H	−2.52840	−0.19640	5.24710
H	3.89610	5.77140	−1.79130
H	4.91640	4.80100	−2.85090
H	3.79730	4.01610	−1.72340
**Marilzafurollene C_5**			
C	2.90470	−1.84780	−1.41580
O	2.27630	−1.98780	−0.14240
C	3.37130	−0.39510	−1.63130
C	1.89690	−2.36780	−2.45140
C	0.94580	−2.46430	−0.31060
C	4.45380	0.01870	−0.61990
Br	1.81960	0.85280	−1.46160
C	0.99420	−3.25790	−1.62010
C	0.44660	−3.21780	0.93850
C	5.01190	1.40110	−0.89740
Cl	1.76260	−4.86050	−1.41540
C	0.45340	−2.35760	2.21560
C	−0.42250	−1.12420	2.09770
O	−0.00590	−3.15130	3.29060
C	0.05450	0.09930	2.13670
C	4.93450	2.43620	−0.04470
C	5.48360	3.82290	−0.32270
C	6.52570	4.21800	0.72660
O	4.45540	4.80180	−0.28170
C	0.56070	1.30920	2.16490
Br	0.78170	2.32760	3.84790
C	3.54050	4.73330	−1.36740
H	3.77140	−2.51070	−1.41800
H	3.75940	−0.29060	−2.64540
H	1.30980	−1.54990	−2.86930

H	2.37010	−2.89120	−3.28260
H	0.30940	−1.59450	−0.48030
H	4.04840	−0.02340	0.39230
H	5.28220	−0.68970	−0.65120
H	0.00990	−3.40690	−2.06520
H	−0.55970	−3.59450	0.75250
H	1.07210	−4.09340	1.11260
H	5.49710	1.53680	−1.85330
H	1.48320	−2.06040	2.42910
H	−1.48150	−1.29740	1.97230
H	0.19760	−2.70550	4.10100
H	4.44710	2.30640	0.91110
H	5.96060	3.83830	−1.30470
H	6.93040	5.20820	0.51690
H	6.09030	4.24250	1.72590
H	7.35850	3.51450	0.73930
H	0.89960	1.85620	1.29660
H	2.98950	3.79230	−1.37470
H	2.81310	5.54040	−1.28120
H	4.05210	4.84560	−2.32410
**Marilzafurollene C_6**			
C	2.90800	−1.81890	−1.42170
O	2.29720	−1.96840	−0.14060
C	3.36470	−0.36340	−1.63780
C	1.88870	−2.33770	−2.44610
C	0.97290	−2.46810	−0.29270
C	4.47610	0.04480	−0.65610
Br	1.81460	0.87750	−1.41070
C	1.01290	−3.24920	−1.60990
C	0.50780	−3.24310	0.95620
C	5.03020	1.42620	−0.94670
Cl	1.80780	−4.84200	−1.43100
C	0.48100	−2.38510	2.23440
C	−0.46240	−1.20170	2.12430
O	0.07300	−3.20330	3.31160
C	−0.05640	0.04680	2.17410
C	4.98150	2.45980	−0.09010
C	5.52720	3.84510	−0.38180
C	6.60100	4.23470	0.63710
O	4.50430	4.82760	−0.30940
C	0.37570	1.28470	2.21430
Br	0.52140	2.30200	3.90640
C	3.55710	4.76330	−1.36740
H	3.77710	−2.47830	−1.44050
H	3.72230	−0.24910	−2.66200
H	1.28430	−1.52120	−2.84180
H	2.35310	−2.84430	−3.29250

H	0.31850	−1.60860	−0.44510
H	4.09940	0.00040	0.36690
H	5.30170	−0.66520	−0.71320
H	0.02410	−3.40890	−2.04110
H	−0.48280	−3.66040	0.77240
H	1.16940	−4.09280	1.12580
H	5.48770	1.56240	−1.91600
H	1.49500	−2.03320	2.43970
H	−1.50960	−1.43370	1.99430
H	0.25870	−2.74830	4.12120
H	4.52170	2.32970	0.87920
H	5.97530	3.86040	−1.37730
H	7.00310	5.22370	0.41710
H	6.19520	4.25920	1.64870
H	7.43120	3.52810	0.62440
H	0.68700	1.85740	1.35220
H	3.00260	3.82430	−1.35870
H	2.83580	5.57310	−1.25910
H	4.04040	4.87420	−2.33880
**Marilzafurollene C_7**			
C	2.90460	−1.85380	−1.41490
O	2.27270	−1.99170	−0.14300
C	3.37330	−0.40170	−1.63040
C	1.89900	−2.37400	−2.45270
C	0.94090	−2.46320	−0.31440
C	4.45030	0.01300	−0.61350
Br	1.82160	0.84770	−1.47180
C	0.99060	−3.25940	−1.62230
C	0.43460	−3.21190	0.93470
C	5.00860	1.39590	−0.88800
Cl	1.75320	−4.86410	−1.41220
C	0.44780	−2.35130	2.21150
C	−0.41390	−1.10810	2.09140
O	−0.02230	−3.13940	3.28580
C	0.07730	0.10990	2.12940
C	4.92530	2.43090	−0.03570
C	5.47440	3.81820	−0.31050
C	6.50990	4.21450	0.74480
O	4.44490	4.79590	−0.27540
C	0.59810	1.31360	2.15630
Br	0.83140	2.33090	3.83820
C	3.53650	4.72670	−1.36650
H	3.77070	−2.51750	−1.41390
H	3.76720	−0.29930	−2.64260
H	1.31550	−1.55580	−2.87510
H	2.37370	−2.90100	−3.28070
H	0.30840	−1.59120	−0.48770

H	4.03950	−0.02910	0.39650
H	5.27930	−0.69490	−0.64030
H	0.00720	−3.40600	−2.07010
H	−0.57460	−3.58030	0.74790
H	1.05260	−4.09260	1.11000
H	5.49890	1.53210	−1.84120
H	1.48030	−2.06540	2.42710
H	−1.47470	−1.26920	1.96540
H	0.18420	−2.69540	4.09650
H	4.43280	2.30050	0.91740
H	5.95710	3.83420	−1.28970
H	6.91470	5.20520	0.53750
H	6.06880	4.23850	1.74160
H	7.34350	3.51200	0.76240
H	0.94360	1.85560	1.28760
H	2.98670	3.78500	−1.37720
H	2.80780	5.53290	−1.28440
H	4.05360	4.83970	−2.32010
**Marilzafurollene C_8**			
C	3.04750	−1.35300	−1.07540
O	2.18950	−1.34460	0.06620
C	3.36430	0.08510	−1.52580
C	2.34850	−2.22180	−2.12930
C	1.03300	−2.14010	−0.18740
C	4.18490	0.85360	−0.47620
Br	1.66210	1.07100	−1.87530
C	1.46800	−3.11890	−1.28380
C	0.48730	−2.76440	1.11180
C	4.61420	2.22380	−0.96410
Cl	2.44100	−4.47230	−0.63410
C	−0.03670	−1.71750	2.11050
C	−0.72940	−2.37080	3.29040
O	1.05940	−0.96200	2.57750
C	−2.00280	−2.20520	3.56780
C	5.89070	2.61540	−1.11210
C	6.31680	3.98800	−1.59870
C	7.13590	3.87950	−2.88740
O	7.13210	4.64360	−0.63840
C	−3.27790	−2.05490	3.83920
Br	−3.95630	−0.61040	5.01070
C	6.43320	5.07990	0.51960
H	3.97130	−1.85320	−0.77990
H	3.91960	0.05460	−2.46480
H	1.72190	−1.61430	−2.78260
H	3.04620	−2.77310	−2.76020
H	0.27400	−1.47870	−0.60980
H	3.60280	0.97310	0.43840

H	5.07070	0.27710	−0.20640
H	0.62820	−3.54010	−1.83770
H	−0.32710	−3.44140	0.84990
H	1.24940	−3.37920	1.59230
H	3.81670	2.91440	−1.20030
H	−0.72970	−1.04330	1.60180
H	−0.11070	−2.99630	3.91720
H	1.63790	−0.82060	1.83160
H	6.69340	1.93200	−0.87510
H	5.43330	4.59600	−1.80330
H	7.42930	4.86690	−3.24400
H	8.04590	3.29990	−2.73020
H	6.56210	3.39760	−3.67930
H	−4.06750	−2.68740	3.46090
H	6.00400	4.24400	1.07300
H	7.12210	5.59900	1.18610
H	5.63310	5.77430	0.26010
**Marilzafurollene C_9**			
C	3.13900	−1.27500	−1.94060
O	2.50940	−0.84220	−0.73250
C	2.91060	−0.25420	−3.07570
C	2.57750	−2.67550	−2.21880
C	1.60200	−1.83680	−0.26640
C	3.77660	1.01770	−2.97490
Br	0.98430	0.26440	−3.14340
C	2.15030	−3.14390	−0.84170
C	1.44840	−1.75680	1.26310
C	3.68530	1.71860	−1.63210
Cl	3.57470	−3.72960	0.06810
C	0.71530	−0.48250	1.71930
C	0.49530	−0.47280	3.21880
O	1.49140	0.63840	1.35790
C	−0.69380	−0.43400	3.77510
C	4.74790	2.03160	−0.87210
C	4.67610	2.69860	0.48780
C	5.26110	4.11180	0.43130
O	5.43750	1.97330	1.44250
C	−1.88830	−0.40690	4.31690
Br	−2.89530	−2.05580	4.74820
C	4.82420	0.76830	1.87940
H	4.20800	−1.36830	−1.74190
H	3.13440	−0.73120	−4.03120
H	1.70260	−2.61400	−2.86740
H	3.29920	−3.33940	−2.69550
H	0.63350	−1.64460	−0.73240
H	4.81900	0.75860	−3.16490
H	3.48790	1.72100	−3.75650

H	1.40990	−3.94410	−0.86970
H	0.88480	−2.62710	1.60230
H	2.42440	−1.81850	1.74640
H	2.69000	1.96330	−1.28790
H	−0.24510	−0.41360	1.20330
H	1.38480	−0.49800	3.83100
H	1.91360	0.42690	0.52720
H	5.74280	1.79490	−1.22030
H	3.63580	2.76570	0.81360
H	6.30880	4.09440	0.13040
H	4.71660	4.73460	−0.27870
H	5.20370	4.59320	1.40760
H	−2.42840	0.49220	4.57550
H	4.59530	0.09710	1.05040
H	5.49620	0.24240	2.55710
H	3.90420	0.97800	2.42500
**Marilzafurollene C_10**			
C	2.82030	−0.50310	−1.02720
O	1.95170	−0.57190	0.10430
C	2.63260	0.83110	−1.77820
C	2.53640	−1.75960	−1.86060
C	1.18060	−1.77120	0.05410
C	3.02070	2.07100	−0.95310
Br	0.71890	1.03190	−2.30690
C	1.99920	−2.72440	−0.82380
C	0.83220	−2.27260	1.46900
C	4.49790	2.12630	−0.61180
Cl	3.36260	−3.46270	0.06910
C	−0.09230	−1.31270	2.23810
C	−0.56220	−1.91980	3.54510
O	0.62190	−0.12680	2.50990
C	−1.82590	−2.14090	3.82740
C	5.35780	3.03540	−1.10020
C	6.83520	3.09230	−0.75900
C	7.19530	4.43380	−0.11560
O	7.63620	2.95800	−1.92390
C	−3.09080	−2.36780	4.09120
Br	−3.98120	−4.07490	3.63030
C	7.64630	1.64960	−2.47820
H	3.84160	−0.57960	−0.65300
H	3.21540	0.81930	−2.70060
H	1.76620	−1.56870	−2.60800
H	3.41710	−2.13440	−2.38280
H	0.25630	−1.53660	−0.47740
H	2.75360	2.96910	−1.51140
H	2.44420	2.10250	−0.02770
H	1.40080	−3.52790	−1.25480

H	0.33690	−3.24030	1.37650
H	1.73910	−2.45060	2.04810
H	4.85780	1.37410	0.07590
H	−0.95440	−1.06180	1.61570
H	0.20950	−2.16980	4.25850
H	1.14970	0.06260	1.73730
H	5.00620	3.79130	−1.78800
H	7.08060	2.29600	−0.05340
H	8.25390	4.46800	0.14240
H	6.99180	5.26430	−0.79200
H	6.62520	4.59740	0.79930
H	−3.75620	−1.66630	4.57300
H	8.30530	1.62740	−3.34610
H	8.01730	0.91860	−1.75870
H	6.65350	1.34170	−2.80820
**Marilzafurollene C_11**			
C	2.68170	−0.59440	−1.59160
O	1.96690	−0.55290	−0.35580
C	2.36230	0.64930	−2.44630
C	2.32380	−1.93510	−2.24590
C	1.22320	−1.75730	−0.18060
C	2.84290	1.96490	−1.81010
Br	0.38950	0.78550	−2.71310
C	1.94170	−2.79060	−1.05560
C	1.07350	−2.10800	1.31240
C	4.35390	2.06300	−1.72150
Cl	3.42070	−3.42720	−0.27590
C	0.24160	−1.07540	2.09300
C	−0.03970	−1.54160	3.50770
O	0.96370	0.13570	2.14060
C	−1.25060	−1.73530	3.97840
C	5.04070	2.15950	−0.57100
C	6.55180	2.25740	−0.47490
C	7.12380	1.08260	0.32240
O	6.95000	3.44760	0.18970
C	−2.46540	−1.93580	4.43150
Br	−3.38090	−3.68410	4.27590
C	6.74230	4.63520	−0.56270
H	3.74480	−0.61310	−1.34680
H	2.80760	0.54060	−3.43630
H	1.46190	−1.82990	−2.90500
H	3.14020	−2.35490	−2.83420
H	0.23270	−1.58760	−0.60710
H	2.49060	2.80800	−2.40490
H	2.40080	2.08060	−0.81940
H	1.30820	−3.63730	−1.32230
H	0.58830	−3.08220	1.38820

H	2.05200	−2.21930	1.78110
H	4.88910	2.05240	−2.66030
H	−0.69970	−0.89580	1.56840
H	0.82430	−1.71200	4.13330
H	1.38580	0.24030	1.29060
H	4.50850	2.17050	0.36990
H	6.98550	2.23690	−1.47660
H	8.21080	1.14370	0.37770
H	6.73960	1.07340	1.34280
H	6.86720	0.12970	−0.14130
H	−3.07220	−1.18850	4.92160
H	5.68540	4.80140	−0.77380
H	7.10510	5.49270	0.00400
H	7.28660	4.60490	−1.50730
**Marilzafurollene C_12**			
C	2.64260	−1.22510	−1.96920
O	2.52770	−1.04030	−0.55700
C	1.80030	−0.18290	−2.73690
C	2.24260	−2.68400	−2.22900
C	2.03600	−2.22650	0.05410
C	2.40750	1.23440	−2.76010
Br	−0.04380	−0.09730	−1.97500
C	2.49100	−3.34370	−0.88660
C	2.48700	−2.32480	1.52340
C	2.72110	1.78400	−1.38170
Cl	4.23290	−3.70960	−0.70710
C	1.90180	−1.20820	2.40730
C	0.39130	−1.28890	2.52340
O	2.47160	−1.31360	3.69560
C	−0.41380	−0.35010	2.08030
C	3.92360	2.25100	−1.00740
C	4.25430	2.76290	0.38100
C	4.60530	4.25220	0.34430
O	5.37820	2.08200	0.92030
C	−1.19760	0.59460	1.61730
Br	−1.77320	2.13220	2.72290
C	5.12440	0.73060	1.28190
H	3.69540	−1.10710	−2.23070
H	1.69070	−0.51220	−3.77120
H	1.18180	−2.75150	−2.47370
H	2.80200	−3.14260	−3.04480
H	0.94650	−2.18970	0.01250
H	3.32270	1.22600	−3.35330
H	1.72200	1.91900	−3.26040
H	1.93180	−4.27010	−0.75220
H	2.20770	−3.29850	1.92720
H	3.57520	−2.27580	1.56960

H	1.90670	1.77550	−0.67110
H	2.18580	−0.24100	1.98680
H	−0.00690	−2.17610	2.99440
H	2.23240	−0.54560	4.19550
H	4.73830	2.26470	−1.71670
H	3.39300	2.62560	1.03810
H	4.83190	4.62130	1.34460
H	5.47730	4.43720	−0.28330
H	3.77660	4.84220	−0.04790
H	−1.59640	0.62810	0.61350
H	4.80320	0.12970	0.43030
H	6.03550	0.28190	1.67710
H	4.36280	0.67100	2.05910
**Marilzafurollene C_13**			
C	2.72890	−0.68090	−1.00020
O	2.19340	−1.13390	0.24260
C	2.19500	0.72700	−1.33860
C	2.39450	−1.76780	−2.03170
C	1.51860	−2.37340	0.05500
C	2.62950	1.81240	−0.33770
Br	0.19900	0.69720	−1.36450
C	2.20320	−2.99650	−1.16530
C	1.50520	−3.21610	1.34570
C	4.12570	2.06380	−0.33320
Cl	3.79870	−3.69910	−0.76300
C	0.76480	−2.53540	2.51160
C	−0.69960	−2.28310	2.20410
O	0.86140	−3.36910	3.64820
C	−1.21900	−1.07930	2.11850
C	4.70560	3.20700	−0.73530
C	6.20150	3.46210	−0.72910
C	6.54820	4.65070	0.17090
O	6.67480	3.77200	−2.03170
C	−1.71240	0.13200	2.01750
Br	−2.48090	1.09790	3.56530
C	6.70290	2.66480	−2.92180
H	3.81330	−0.65220	−0.88990
H	2.52010	1.01300	−2.34010
H	1.46150	−1.54230	−2.54860
H	3.16920	−1.89120	−2.78880
H	0.49200	−2.14220	−0.23320
H	2.11600	2.74450	−0.57620
H	2.31600	1.53860	0.67060
H	1.60140	−3.77010	−1.64350
H	1.05180	−4.18600	1.13870
H	2.52980	−3.41910	1.65780
H	4.74510	1.25460	0.02690

H	1.26500	−1.59070	2.73920
H	−1.31430	−3.15800	2.04890
H	0.60390	−2.86890	4.41010
H	4.09330	4.02130	−1.09560
H	6.72340	2.58080	−0.35080
H	7.62430	4.82440	0.18580
H	6.06960	5.56540	−0.17980
H	6.22620	4.47460	1.19750
H	−1.74770	0.70850	1.10420
H	5.70560	2.25950	−3.09580
H	7.10410	2.98200	−3.88440
H	7.34200	1.86750	−2.54030
**Marilzafurollene C_14**			
C	2.66480	−1.90140	−1.44580
O	2.31290	−2.02020	−0.06790
C	3.52610	−0.64370	−1.66800
C	1.34610	−1.97300	−2.22810
C	0.90410	−2.19220	0.06280
C	2.87830	0.66890	−1.19050
Br	3.98700	−0.50340	−3.60310
C	0.45360	−2.75750	−1.28840
C	0.56230	−3.03580	1.30590
C	3.83870	1.84010	−1.24700
Cl	0.80220	−4.50600	−1.44000
C	0.88000	−2.31950	2.63140
C	−0.03980	−1.13960	2.88600
O	0.73540	−3.24300	3.69070
C	0.37130	0.10680	2.94310
C	4.26570	2.52310	−0.17210
C	5.21640	3.70370	−0.22430
C	6.51380	3.39200	0.52560
O	4.64620	4.85230	0.38640
C	0.76310	1.35830	2.97780
Br	0.79370	2.50290	1.36300
C	3.55490	5.41530	−0.32960
H	3.25920	−2.78170	−1.69550
H	4.46620	−0.78430	−1.13200
H	0.92450	−0.98070	−2.38280
H	1.45850	−2.43940	−3.20730
H	0.46520	−1.19810	0.16320
H	2.00230	0.91510	−1.78930
H	2.53040	0.55030	−0.16380
H	−0.60910	−2.60640	−1.48090
H	−0.49310	−3.31040	1.28340
H	1.12090	−3.97100	1.25930
H	4.18480	2.12390	−2.23100
H	1.91890	−1.98100	2.61880

H	−1.08440	−1.37560	3.02730
H	1.46630	−3.84490	3.66910
H	3.92160	2.24130	0.81290
H	5.46360	3.92970	−1.26350
H	7.20240	4.23550	0.47550
H	6.32120	3.18290	1.57820
H	7.01790	2.52560	0.09690
H	1.09680	1.87730	3.86460
H	2.71040	4.72760	−0.38860
H	3.21160	6.31520	0.18050
H	3.84840	5.69620	−1.34180
**Marilzafurollene C_15**			
C	2.83300	−1.08730	−0.45360
O	1.53620	−0.56530	−0.15120
C	3.62900	−0.05880	−1.27860
C	2.59830	−2.43020	−1.16110
C	0.55690	−1.59880	−0.23210
C	3.89330	1.23860	−0.49590
Br	2.63280	0.37450	−2.95440
C	1.09910	−2.44480	−1.38630
C	0.44890	−2.31700	1.13470
C	4.79480	2.19720	−1.24950
Cl	0.43050	−4.09870	−1.46210
C	−0.04370	−1.40140	2.26980
C	−0.32590	−2.18600	3.53590
O	0.95160	−0.43800	2.53730
C	−1.50900	−2.24270	4.10360
C	5.99660	2.61360	−0.81690
C	6.89570	3.57430	−1.57260
C	8.23690	2.91680	−1.90730
O	7.17800	4.73090	−0.79840
C	−2.69870	−2.29980	4.65380
Br	−4.02500	−3.68860	4.17290
C	6.07450	5.61310	−0.64390
H	3.35460	−1.27810	0.48590
H	4.58300	−0.49750	−1.57630
H	3.15610	−2.53980	−2.09160
H	2.89090	−3.24860	−0.50230
H	−0.41230	−1.17270	−0.49430
H	2.95060	1.74210	−0.27780
H	4.34840	1.00220	0.46680
H	0.86600	−1.95330	−2.33210
H	−0.26110	−3.13920	1.03600
H	1.39830	−2.77680	1.41100
H	4.41840	2.55630	−2.19730
H	−0.94950	−0.88220	1.94820
H	0.51300	−2.71240	3.96700

H	1.25930	−0.11830	1.69320
H	6.37820	2.26170	0.13080
H	6.41490	3.86940	−2.50750
H	8.09670	2.02520	−2.51890
H	8.87660	3.60290	−2.46270
H	8.77090	2.62450	−1.00280
H	−3.06510	−1.61440	5.40410
H	5.24920	5.14040	−0.11050
H	6.38410	6.48530	−0.06810
H	5.71030	5.96350	−1.61040
**Marilzafurollene C_16**			
C	2.44500	−0.64620	−1.48460
O	2.15020	−0.97320	−0.12690
C	1.68690	0.62810	−1.91230
C	2.11640	−1.90000	−2.30700
C	1.62740	−2.29490	−0.04920
C	2.13370	1.88510	−1.14670
Br	−0.26890	0.38510	−1.59440
C	2.20760	−3.00660	−1.27530
C	1.91070	−2.93870	1.32250
C	3.56410	2.28770	−1.45040
Cl	3.91960	−3.47200	−1.04510
C	1.26290	−2.18280	2.49710
C	−0.25080	−2.14010	2.39890
O	1.62970	−2.82850	3.69920
C	−0.93020	−1.02560	2.24970
C	4.55110	2.31790	−0.53970
C	5.98480	2.71720	−0.83470
C	6.94480	1.56620	−0.52390
O	6.38580	3.82340	−0.03940
C	−1.58750	0.09710	2.08060
Br	−2.24610	1.18130	3.60040
C	5.78020	5.05600	−0.40440
H	3.52080	−0.47620	−1.54610
H	1.81480	0.79060	−2.98340
H	1.09940	−1.85400	−2.69700
H	2.78830	−2.04400	−3.15350
H	0.54880	−2.22430	−0.19690
H	1.48960	2.72370	−1.41250
H	2.00860	1.72750	−0.07420
H	1.64750	−3.89980	−1.55370
H	1.56420	−3.97260	1.31450
H	2.98730	−2.97930	1.48940
H	3.77630	2.56280	−2.47370
H	1.66280	−1.16600	2.52120
H	−0.76130	−3.09080	2.45290
H	1.42580	−2.25300	4.42310

H	4.34120	2.04240	0.48420
H	6.08470	2.97270	−1.89140
H	7.97330	1.84650	−0.75160
H	6.90160	1.28930	0.52970
H	6.70280	0.68140	−1.11340
H	−1.82860	0.53090	1.12070
H	4.69740	5.02860	−0.27800
H	6.16720	5.85320	0.23020
H	6.00440	5.31510	−1.43990
**Marilzafurollene C_17**			
C	2.67070	−1.90740	−1.48390
O	2.30820	−2.02690	−0.10910
C	3.54270	−0.65600	−1.69840
C	1.35680	−1.96830	−2.27440
C	0.90160	−2.22660	0.01070
C	2.90120	0.66070	−1.22380
Br	4.01820	−0.51650	−3.63020
C	0.46380	−2.77320	−1.35280
C	0.56070	−3.09540	1.23720
C	3.86970	1.82540	−1.27500
Cl	0.82330	−4.51690	−1.53250
C	0.86420	−2.40460	2.57910
C	−0.05140	−1.22210	2.83730
O	0.68780	−3.35150	3.61290
C	0.36980	0.01810	2.93860
C	4.29740	2.50450	−0.19780
C	5.25660	3.67850	−0.24550
C	6.54890	3.35720	0.50940
O	4.69220	4.83080	0.36360
C	0.76950	1.26550	3.01260
Br	0.85750	2.44330	1.42440
C	3.60730	5.40150	−0.35600
H	3.26020	−2.79150	−1.73180
H	4.47780	−0.80440	−1.15590
H	0.93230	−0.97480	−2.41230
H	1.47540	−2.41660	−3.26130
H	0.44760	−1.24090	0.12490
H	2.03020	0.91280	−1.82750
H	2.54680	0.54340	−0.19920
H	−0.59860	−2.62570	−1.54920
H	−0.49130	−3.38110	1.20320
H	1.12910	−4.02450	1.18180
H	4.22170	2.10780	−2.25730
H	1.90880	−2.08390	2.58420
H	−1.10300	−1.44990	2.93740
H	1.12650	−3.03680	4.39080
H	3.94770	2.22410	0.78560

H	5.50950	3.90330	−1.28360
H	7.24370	4.19580	0.46230
H	6.35070	3.14900	1.56110
H	7.04850	2.48740	0.08210
H	1.07980	1.76360	3.91970
H	3.90580	5.67980	−1.36740
H	2.75800	4.71990	−0.41720
H	3.26890	6.30410	0.15270
**Marilzafurollene C_18**			
C	2.89090	−1.78890	−1.36950
O	2.28330	−1.93000	−0.08580
C	3.34470	−0.33470	−1.59990
C	1.87090	−2.31840	−2.38760
C	0.95970	−2.43310	−0.23050
C	4.45250	0.08780	−0.62020
Br	1.79160	0.90550	−1.39160
C	0.99760	−3.22350	−1.54230
C	0.50730	−3.20420	1.02520
C	5.01120	1.46260	−0.93210
Cl	1.79370	−4.81460	−1.35250
C	0.46620	−2.33440	2.29540
C	−0.51860	−1.18470	2.18960
O	0.10520	−3.15140	3.39050
C	−0.15260	0.07720	2.18290
C	4.96250	2.51100	−0.09360
C	5.51360	3.88930	−0.40740
C	6.58760	4.29170	0.60640
O	4.49450	4.87690	−0.35280
C	0.23900	1.32910	2.15830
Br	0.42890	2.41730	3.80120
C	3.54700	4.79720	−1.40950
H	3.76150	−2.44660	−1.38460
H	3.70470	−0.23120	−2.62440
H	1.26450	−1.50660	−2.78990
H	2.33480	−2.83180	−3.23020
H	0.30130	−1.57670	−0.38440
H	4.07150	0.06120	0.40190
H	5.27710	−0.62460	−0.66210
H	0.00800	−3.38720	−1.97050
H	−0.47740	−3.63750	0.84540
H	1.18310	−4.04200	1.19670
H	5.47260	1.58100	−1.90190
H	1.46580	−1.93630	2.48650
H	−1.56190	−1.45410	2.11720
H	0.86640	−3.64410	3.66330
H	4.49870	2.39900	0.87610
H	5.96310	3.88660	−1.40230

H	6.99360	5.27550	0.37060
H	6.18070	4.33440	1.61700
H	7.41520	3.58200	0.60640
H	0.49100	1.87770	1.26210
H	2.98860	3.86090	−1.38370
H	2.82900	5.61180	−1.31600
H	4.03080	4.88840	−2.38280
**Marilzafurollene C_19**			
C	3.09660	−1.65480	−1.36420
O	2.34150	−1.55580	−0.15620
C	3.73900	−0.29970	−1.71470
C	2.13620	−2.20940	−2.42450
C	1.00640	−2.00620	−0.37740
C	4.78650	0.13090	−0.67360
Br	2.32400	1.10560	−1.83320
C	1.10900	−2.94270	−1.58650
C	0.39890	−2.60680	0.90530
C	5.50370	1.40890	−1.06330
Cl	1.74200	−4.55540	−1.14020
C	0.22370	−1.57260	2.03140
C	−0.54060	−2.15190	3.20580
O	1.50360	−1.16850	2.46600
C	−1.70930	−1.70310	3.60420
C	5.51240	2.52980	−0.32300
C	6.21760	3.81450	−0.71550
C	7.29010	4.18480	0.31200
O	5.30780	4.90320	−0.78190
C	−2.88390	−1.26860	3.99600
Br	−3.09560	0.16820	5.34150
C	4.38230	4.82740	−1.85830
H	3.88030	−2.39470	−1.19350
H	4.20830	−0.36820	−2.69720
H	1.64650	−1.40130	−2.96830
H	2.63040	−2.84810	−3.15710
H	0.41830	−1.13610	−0.67550
H	4.31160	0.25350	0.30100
H	5.53670	−0.65190	−0.55680
H	0.15730	−3.08210	−2.10050
H	−0.57650	−3.02750	0.65710
H	1.00520	−3.43930	1.26440
H	6.03370	1.39130	−2.00480
H	−0.30220	−0.69710	1.64350
H	−0.06650	−2.97430	3.72130
H	2.05120	−1.09440	1.68780
H	4.98280	2.55290	0.61880
H	6.69990	3.68580	−1.68650
H	7.80620	5.09980	0.02070

H	6.85300	4.34980	1.29710
H	8.03750	3.39630	0.40310
H	−3.83110	−1.64040	3.63380
H	3.75700	5.72010	−1.86340
H	4.89590	4.77390	−2.81900
H	3.72280	3.96380	−1.76670
**Marilzafurollene C_20**			
C	2.61480	−1.14200	−1.76660
O	1.48130	−1.09150	−0.90570
C	2.46620	−0.14190	−2.92880
C	2.75600	−2.62270	−2.14990
C	1.43110	−2.29790	−0.15470
C	2.30020	1.31700	−2.45810
Br	0.87430	−0.62070	−4.03080
C	1.72930	−3.31400	−1.26160
C	2.45650	−2.24640	1.00690
C	3.38250	1.75850	−1.48800
Cl	2.28870	−4.89930	−0.65950
C	2.30810	−0.98950	1.88300
C	1.05770	−1.02170	2.73930
O	3.44340	−0.86290	2.71180
C	0.07770	−0.15640	2.61320
C	3.13530	2.19140	−0.23740
C	4.16270	2.59510	0.81390
C	5.61500	2.31160	0.40670
O	3.94090	1.88680	2.02920
C	−0.88420	0.72440	2.47280
Br	−0.90590	2.43590	3.46660
C	2.92190	2.45110	2.84730
H	3.49410	−0.86980	−1.18150
H	3.33710	−0.21680	−3.58140
H	2.55860	−2.81820	−3.20390
H	3.76740	−2.96790	−1.93350
H	0.42770	−2.44260	0.24710
H	2.30090	1.98700	−3.31820
H	1.32600	1.42900	−1.97990
H	0.81820	−3.48460	−1.83720
H	2.36120	−3.13960	1.62520
H	3.47010	−2.28160	0.60790
H	4.39570	1.69270	−1.85540
H	2.28840	−0.10850	1.23960
H	1.00630	−1.80750	3.47860
H	3.78940	0.01830	2.58790
H	2.10480	2.25490	0.08000
H	4.06610	3.66880	0.98660
H	6.30020	2.60430	1.20250
H	5.77330	1.25050	0.21170

H	5.89750	2.87140	−0.48510
H	−1.73650	0.60940	1.81910
H	1.95850	2.47520	2.33880
H	2.80210	1.85220	3.75020
H	3.17820	3.46590	3.15430
**Marilzafurollene C_21**			
C	3.04110	−1.16440	−1.41450
O	1.72560	−0.68380	−1.12770
C	3.60360	−0.43900	−2.65360
C	2.91220	−2.68790	−1.56300
C	0.91530	−1.74690	−0.63430
C	3.72430	1.08370	−2.46720
Br	2.41380	−0.77550	−4.21870
C	1.41500	−2.91840	−1.48230
C	1.11640	−1.88920	0.89410
C	4.67690	1.46900	−1.35170
Cl	0.99970	−4.52630	−0.82650
C	0.69330	−0.63920	1.68640
C	0.71490	−0.89260	3.18070
O	1.58750	0.40950	1.38560
C	−0.34730	−0.79400	3.94690
C	4.30940	2.12180	−0.23650
C	5.24940	2.50040	0.89220
C	4.79640	1.86510	2.20890
O	5.27810	3.90660	1.09090
C	−1.41890	−0.70050	4.69800
Br	−2.62130	−2.23800	5.02840
C	5.93560	4.62990	0.05970
H	3.67390	−0.96030	−0.54920
H	4.58110	−0.85170	−2.90770
H	3.33780	−3.07640	−2.48860
H	3.41960	−3.18220	−0.73370
H	−0.13510	−1.54160	−0.84430
H	4.08180	1.54030	−3.39060
H	2.73870	1.51020	−2.27480
H	0.98120	−2.84810	−2.48120
H	0.51400	−2.72940	1.24190
H	2.14890	−2.14790	1.13110
H	5.71100	1.18480	−1.48540
H	−0.31010	−0.33730	1.37730
H	1.67080	−1.16450	3.60380
H	1.72230	0.39310	0.44150
H	3.27650	2.40940	−0.09830
H	6.25640	2.14430	0.66640
H	3.79860	2.20540	2.48850
H	4.76410	0.77800	2.13140
H	5.47610	2.12260	3.02100

H	−1.74460	0.19960	5.19870
H	5.94320	5.69110	0.30810
H	6.97110	4.30590	−0.05190
H	5.42930	4.51830	−0.89970
**Marilzafurollene C_22**			
C	2.97130	−1.70130	−1.33220
O	2.35570	−1.54120	−0.05440
C	4.05030	−0.62400	−1.55260
C	1.82350	−1.73760	−2.34900
C	0.93580	−1.64150	−0.17750
C	3.54150	0.82740	−1.46860
Br	4.91250	−0.92810	−3.32470
C	0.69140	−2.32250	−1.53060
C	0.31260	−2.32590	1.05520
C	4.67480	1.83430	−1.48650
Cl	0.86970	−4.10060	−1.43870
C	0.47740	−1.51100	2.34990
C	−0.30500	−2.12230	3.49540
O	1.84660	−1.48200	2.68940
C	−1.26710	−1.49380	4.13140
C	4.94410	2.69610	−0.49180
C	6.07870	3.70350	−0.51120
C	7.04770	3.45500	0.64740
O	5.59080	5.02990	−0.37210
C	−2.23430	−0.85870	4.74980
Br	−4.11910	−0.95570	4.15210
C	4.90670	5.52290	−1.51610
H	3.45370	−2.68020	−1.32630
H	4.82710	−0.76900	−0.79970
H	1.55130	−0.73500	−2.67590
H	2.06140	−2.32280	−3.23790
H	0.55350	−0.62140	−0.24710
H	2.87310	1.05300	−2.29880
H	2.96260	0.96210	−0.55390
H	−0.29350	−2.10460	−1.94550
H	−0.75190	−2.47040	0.86400
H	0.73260	−3.32290	1.19430
H	5.29260	1.83760	−2.37380
H	0.14180	−0.48550	2.17910
H	−0.02930	−3.12810	3.77720
H	2.33760	−1.43950	1.87260
H	4.32920	2.69960	0.39690
H	6.63290	3.61470	−1.44770
H	7.87040	4.16990	0.62450
H	6.54700	3.55640	1.61060
H	7.47630	2.45390	0.59460
H	−2.10440	−0.23610	5.62300

H	5.55200	5.51670	−2.39550
H	4.01190	4.93960	−1.73580
H	4.59540	6.55220	−1.33820
**Marilzafurollene C_23**			
C	2.96160	−1.35970	−0.61240
O	1.66540	−1.11960	−0.06000
C	3.45870	−0.09740	−1.34170
C	2.82090	−2.60030	−1.50720
C	0.85000	−2.27980	−0.18950
C	3.64500	1.09270	−0.38560
Br	2.16260	0.40660	−2.77560
C	1.32380	−2.83700	−1.53350
C	1.06410	−3.20360	1.03360
C	4.26430	2.29590	−1.06990
Cl	0.90050	−4.54940	−1.81090
C	0.69340	−2.54690	2.37580
C	−0.78290	−2.20930	2.46780
O	1.02720	−3.44540	3.41360
C	−1.23500	−0.98660	2.63030
C	5.44300	2.84380	−0.73040
C	6.05930	4.04880	−1.41630
C	7.41500	3.69190	−2.03090
O	6.28250	5.10620	−0.49500
C	−1.66740	0.24100	2.79560
Br	−1.95790	1.03160	4.58690
C	5.09710	5.75080	−0.04850
H	3.64440	−1.59770	0.20490
H	4.40950	−0.31600	−1.83050
H	3.23550	−2.47000	−2.50720
H	3.33090	−3.44650	−1.04550
H	−0.19920	−1.99010	−0.25280
H	2.68300	1.38540	0.03710
H	4.27450	0.79530	0.45410
H	0.87600	−2.25440	−2.34000
H	0.47560	−4.11340	0.91110
H	2.10440	−3.52670	1.07370
H	3.69510	2.72350	−1.88360
H	1.29260	−1.64270	2.50710
H	−1.46860	−3.04060	2.38850
H	0.95190	−2.99100	4.24120
H	6.01610	2.42380	0.08370
H	5.39990	4.39400	−2.21540
H	7.85030	4.55440	−2.53590
H	8.12110	3.36320	−1.26790
H	7.31630	2.89220	−2.76540
H	−1.90220	0.92610	1.99390
H	4.44430	5.06720	0.49550

H	5.35850	6.56590	0.62630
H	4.53930	6.17530	−0.88420
**Marilzafurollene C_24**			
C	2.92710	−1.51350	−1.17750
O	2.25760	−1.82500	0.04360
C	3.17510	0.00330	−1.28530
C	2.07040	−2.11390	−2.30150
C	1.03400	−2.49710	−0.23440
C	4.14200	0.50790	−0.20100
Br	1.44360	0.98890	−1.12770
C	1.28060	−3.18750	−1.57940
C	0.59980	−3.39940	0.93780
C	4.49450	1.97250	−0.37290
Cl	2.28550	−4.65840	−1.41350
C	0.36600	−2.62720	2.24930
C	−0.72820	−1.58300	2.12650
O	0.01070	−3.55370	3.25520
C	−0.50620	−0.29370	2.24580
C	5.73200	2.43820	−0.61700
C	6.10350	3.90180	−0.79530
C	6.91060	4.40620	0.40340
O	4.96250	4.74220	−0.92070
C	−0.25520	0.98970	2.35010
Br	−0.33600	1.95050	4.07890
C	4.39350	4.75890	−2.22360
H	3.88410	−2.03750	−1.16020
H	3.58800	0.23110	−2.26920
H	1.38240	−1.37110	−2.70590
H	2.66330	−2.50000	−3.13100
H	0.27380	−1.73160	−0.39720
H	3.70120	0.36640	0.78670
H	5.05730	−0.08490	−0.21890
H	0.35880	−3.46150	−2.09350
H	−0.30700	−3.93830	0.66090
H	1.36230	−4.15770	1.11630
H	3.67390	2.67090	−0.28440
H	1.30340	−2.14880	2.54390
H	−1.72360	−1.95260	1.92670
H	0.07980	−3.12430	4.09640
H	6.55310	1.74010	−0.69640
H	6.72570	3.99070	−1.68790
H	7.20040	5.44790	0.26500
H	6.32810	4.34310	1.32300
H	7.82160	3.82350	0.54170
H	0.01430	1.63390	1.52540
H	4.08330	3.76460	−2.54650
H	3.51250	5.40060	−2.22800

H	5.09890	5.15620	−2.95440
	**Marilzafurollene C_25**		
C	2.93960	−1.20690	−1.08800
O	2.19730	−1.24290	0.13160
C	3.43800	0.22090	−1.37790
C	2.02300	−1.80320	−2.16430
C	0.91190	−1.81750	−0.09750
C	4.45960	0.70200	−0.33360
Br	1.88830	1.48150	−1.40860
C	1.09000	−2.67840	−1.35300
C	0.38940	−2.53910	1.15980
C	5.05510	2.05170	−0.68360
Cl	1.89210	−4.23710	−0.99520
C	0.12720	−1.58400	2.33740
C	−0.55840	−2.29370	3.48810
O	1.36470	−1.07260	2.78170
C	−1.74190	−1.95420	3.94570
C	6.35620	2.26970	−0.94330
C	6.97090	3.61420	−1.29880
C	7.83810	4.13450	−0.14990
O	5.98960	4.60750	−1.57100
C	−2.92990	−1.61200	4.38460
Br	−4.59640	−2.39060	3.65240
C	5.44580	4.54290	−2.88320
H	3.79560	−1.87210	−0.96310
H	3.89650	0.24850	−2.36770
H	1.44470	−1.02330	−2.66020
H	2.56720	−2.35080	−2.93420
H	0.23320	−0.99910	−0.34530
H	3.98770	0.77040	0.64740
H	5.26320	−0.02950	−0.24030
H	0.14910	−2.89000	−1.86240
H	−0.54230	−3.04460	0.90160
H	1.08270	−3.32170	1.47020
H	4.36200	2.88090	−0.71610
H	−0.48970	−0.74890	1.99760
H	−0.01560	−3.11570	3.93150
H	1.89300	−0.90920	2.00360
H	7.04930	1.44160	−0.90180
H	7.60910	3.47810	−2.17400
H	8.30090	5.08570	−0.41340
H	7.24320	4.29290	0.75000
H	8.63630	3.43300	0.09380
H	−3.11440	−0.89130	5.16790
H	4.68570	5.31530	−3.00070
H	6.21600	4.71600	−3.63570
H	4.97580	3.57960	−3.08350

**Marilzafurollene C_26**			
C	3.26570	−1.77560	−1.82380
O	2.67620	−1.32910	−0.60160
C	3.58540	−0.58710	−2.75530
C	2.28120	−2.79730	−2.40990
C	1.41100	−1.95360	−0.40480
C	4.76920	0.29270	−2.30110
Br	1.96530	0.55710	−2.98600
C	1.51390	−3.26460	−1.18890
C	1.08240	−2.06530	1.09610
C	4.57890	0.92430	−0.93440
Cl	2.47010	−4.49820	−0.31490
C	0.87650	−0.69540	1.76790
C	0.41600	−0.84270	3.20440
O	2.10090	0.00510	1.74360
C	−0.72780	−0.37450	3.64910
C	4.39910	2.23680	−0.71480
C	4.19380	2.85430	0.65560
C	2.83430	3.55200	0.73810
O	5.18800	3.83040	0.93260
C	−1.87820	0.08930	4.07630
Br	−3.54000	−0.98540	4.02700
C	6.47760	3.28940	1.18430
H	4.18990	−2.29670	−1.56910
H	3.82680	−0.97860	−3.74480
H	1.59280	−2.31390	−3.10400
H	2.77490	−3.60880	−2.94520
H	0.65830	−1.32830	−0.88880
H	5.68260	−0.30220	−2.27160
H	4.93810	1.07720	−3.03980
H	0.54190	−3.69570	−1.43120
H	0.17020	−2.65330	1.20660
H	1.86460	−2.61720	1.61880
H	4.58650	0.24640	−0.09290
H	0.14110	−0.12040	1.20040
H	1.08940	−1.36390	3.86900
H	2.50340	−0.17920	0.89760
H	4.38840	2.92350	−1.54870
H	4.22400	2.07420	1.41920
H	2.68010	3.98330	1.72710
H	2.75500	4.35570	0.00600
H	2.02310	2.84620	0.55520
H	−2.03120	1.07680	4.48680
H	7.17130	4.09680	1.41840
H	6.46040	2.60690	2.03500
H	6.86980	2.75660	0.31730

Marilzafurollene C_27			
C	3.24430	−1.29710	−1.96050
O	2.57740	−0.93720	−0.74850
C	2.93830	−0.27940	−3.07990
C	2.79890	−2.73340	−2.26480
C	1.75160	−2.00930	−0.30320
C	3.69710	1.05710	−2.95390
Br	0.97630	0.08110	−3.15030
C	2.40510	−3.25810	−0.89820
C	1.58760	−1.96880	1.22710
C	3.54280	1.72680	−1.60100
Cl	3.86800	−3.74430	0.00920
C	0.75230	−0.76640	1.70290
C	0.52950	−0.80090	3.20190
O	1.43780	0.41840	1.36310
C	−0.65560	−0.91390	3.75740
C	4.57310	2.11510	−0.83120
C	4.44130	2.75320	0.53810
C	4.90800	4.21060	0.50540
O	5.25670	2.07880	1.48550
C	−1.83580	−1.04200	4.31640
Br	−2.96640	0.51270	4.78990
C	4.74120	0.82250	1.90380
H	4.31660	−1.30710	−1.75800
H	3.20440	−0.72060	−4.04180
H	1.92460	−2.73170	−2.91710
H	3.57380	−3.32870	−2.74850
H	0.77230	−1.88760	−0.77010
H	4.75790	0.88690	−3.14190
H	3.35540	1.74700	−3.72600
H	1.73120	−4.11430	−0.94400
H	1.09620	−2.88820	1.54850
H	2.56410	−1.95920	1.71310
H	2.52950	1.88280	−1.25790
H	−0.20970	−0.76550	1.18520
H	1.41490	−0.72200	3.81580
H	1.87370	0.25790	0.52820
H	5.58540	1.96720	−1.17800
H	3.39780	2.72950	0.85970
H	4.80730	4.67070	1.48840
H	5.95460	4.28430	0.20940
H	4.31640	4.79710	−0.19800
H	−2.29960	−1.98430	4.56890
H	5.45200	0.34270	2.57620
H	3.80640	0.95010	2.44950
H	4.56860	0.14690	1.06480

**Marilzafurollene C_28**			
C	2.69120	−0.67110	−0.97150
O	2.15760	−1.10910	0.27770
C	2.15660	0.73220	−1.32780
C	2.35670	−1.77110	−1.98910
C	1.47810	−2.34800	0.10510
C	2.58620	1.82980	−0.33810
Br	0.16080	0.69990	−1.36100
C	2.16150	−2.98790	−1.10720
C	1.47000	−3.17270	1.40700
C	4.08260	2.07950	−0.32730
Cl	3.75460	−3.68980	−0.69400
C	0.71380	−2.48530	2.55920
C	−0.75960	−2.28460	2.25620
O	0.84090	−3.28040	3.72060
C	−1.31040	−1.10070	2.10980
C	4.66670	3.21630	−0.74110
C	6.16280	3.46940	−0.72840
C	6.50490	4.67010	0.15730
O	6.64520	3.76050	−2.03190
C	−1.83660	0.08920	1.94120
Br	−2.61720	1.13050	3.43320
C	6.67780	2.64100	−2.90640
H	3.77560	−0.64020	−0.86260
H	2.48500	1.00710	−2.33130
H	1.42530	−1.55100	−2.51100
H	3.13270	−1.90580	−2.74310
H	0.45130	−2.11880	−0.18370
H	2.07510	2.75930	−0.59160
H	2.26600	1.56920	0.67160
H	1.55790	−3.76580	−1.57620
H	1.02870	−4.15070	1.21130
H	2.49720	−3.35830	1.72100
H	4.69850	1.27450	0.04780
H	1.17640	−1.51610	2.76130
H	−1.35410	−3.18140	2.16260
H	1.69870	−3.13430	4.09400
H	4.05780	4.02640	−1.11650
H	6.68090	2.59290	−0.33420
H	7.58110	4.84260	0.17730
H	6.03000	5.58040	−0.20950
H	6.17540	4.50880	1.18400
H	−1.89510	0.60730	0.99480
H	7.08610	2.94430	−3.87050
H	7.31290	1.84810	−2.50950
H	5.68090	2.23490	−3.08170

Marilzafurollene C_29			
C	2.80400	−1.17180	−0.87310
O	1.91680	−0.92970	0.21970
C	3.32010	0.15640	−1.45690
C	2.03340	−2.05740	−1.86120
C	0.67310	−1.59060	−0.00740
C	4.19770	0.92700	−0.45630
Br	1.77340	1.30390	−1.98900
C	1.01390	−2.73480	−0.96860
C	−0.00340	−1.98760	1.31940
C	4.81790	2.17000	−1.06460
Cl	1.77990	−4.11680	−0.12950
C	−0.41730	−0.76880	2.17620
C	−1.25040	−1.09050	3.40980
O	0.75460	−0.09720	2.58340
C	−1.63390	−2.28950	3.78950
C	6.13890	2.37960	−1.19110
C	6.75640	3.62540	−1.79860
C	7.59850	3.27270	−3.02730
O	7.61610	4.27600	−0.87430
C	−2.02650	−3.48760	4.15220
Br	−3.73270	−4.27680	3.53210
C	6.94010	4.92250	0.19570
H	3.64460	−1.74810	−0.48310
H	3.89790	−0.04730	−2.35990
H	1.51520	−1.45240	−2.60550
H	2.67390	−2.76010	−2.39480
H	0.02740	−0.88800	−0.53770
H	3.60550	1.21910	0.41190
H	4.99130	0.27710	−0.08520
H	0.14610	−3.10720	−1.51420
H	−0.88950	−2.57620	1.07710
H	0.64980	−2.64010	1.90020
H	4.12550	2.92470	−1.41080
H	−1.00340	−0.08470	1.56020
H	−1.53130	−0.23170	4.00200
H	1.37240	−0.15120	1.85710
H	6.83730	1.63140	−0.84460
H	5.96690	4.31300	−2.10890
H	8.02990	4.16990	−3.47140
H	8.41940	2.60470	−2.76570
H	6.99470	2.78180	−3.79090
H	−1.47430	−4.14650	4.80620
H	6.38650	4.21380	0.81270
H	7.66700	5.42150	0.83650
H	6.24680	5.67880	−0.17440

**Marilzafurollene C_30**			
C	2.41080	−0.64230	−1.45380
O	2.11780	−0.95370	−0.09180
C	1.65540	0.62910	−1.89520
C	2.07800	−1.90440	−2.26190
C	1.58390	−2.26970	0.00040
C	2.10210	1.89280	−1.14060
Br	−0.30150	0.39210	−1.58030
C	2.16000	−2.99920	−1.21710
C	1.86920	−2.89690	1.37890
C	3.53430	2.28960	−1.44370
Cl	3.86850	−3.47360	−0.97820
C	1.20870	−2.13570	2.54340
C	−0.30660	−2.13700	2.46030
O	1.60360	−2.73890	3.75880
C	−1.01830	−1.05050	2.26160
C	4.51880	2.32550	−0.53050
C	5.95410	2.71940	−0.82480
C	6.91040	1.56750	−0.50600
O	6.35650	3.82870	−0.03450
C	−1.70980	0.04150	2.03680
Br	−2.39020	1.19210	3.49740
C	5.75490	5.06110	−0.40720
H	3.48690	−0.47540	−1.51880
H	1.78610	0.78120	−2.96750
H	1.06240	−1.85790	−2.65560
H	2.75140	−2.06200	−3.10470
H	0.50540	−2.19410	−0.14610
H	1.46040	2.72990	−1.41670
H	1.97320	1.74620	−0.06710
H	1.59420	−3.89180	−1.48610
H	1.53120	−3.93390	1.38020
H	2.94630	−2.92630	1.54380
H	3.74990	2.55550	−2.46880
H	1.56970	−1.10420	2.54550
H	−0.79000	−3.09610	2.57380
H	2.48260	−2.45400	3.96530
H	4.30530	2.05930	0.49520
H	6.05680	2.96910	−1.88270
H	7.94010	1.84400	−0.73320
H	6.86460	1.29640	0.54900
H	6.66730	0.68030	−1.09130
H	−1.97090	0.41310	1.05640
H	5.98200	5.31420	−1.44350
H	4.67170	5.03720	−0.28300
H	6.14260	5.86050	0.22410

**Marilzafurollene C_31**			
C	3.07980	−0.66300	−0.59010
O	2.30200	−0.21920	0.52150
C	3.67290	0.53270	−1.35860
C	2.16360	−1.57480	−1.41500
C	1.00690	−0.81470	0.48340
C	4.67480	1.32820	−0.50520
Br	2.20640	1.75280	−1.95230
C	1.18320	−2.07390	−0.37450
C	0.45320	−1.00830	1.90820
C	5.36620	2.42500	−1.29190
Cl	1.91880	−3.42320	0.54370
C	−0.91050	−1.72300	1.97720
C	−1.36170	−1.90260	3.41360
O	−1.87740	−0.96450	1.28080
C	−1.59140	−3.07500	3.96000
C	6.69070	2.49070	−1.50680
C	7.37950	3.59060	−2.29310
C	8.11330	3.01530	−3.50700
O	8.34630	4.26230	−1.49880
C	−1.83640	−4.24890	4.49240
Br	−3.62500	−5.09370	4.41930
C	7.79400	5.09290	−0.48640
H	3.89140	−1.27180	−0.18840
H	4.17380	0.16910	−2.25710
H	1.62470	−1.00500	−2.17220
H	2.70070	−2.37630	−1.92260
H	0.35890	−0.12220	−0.05720
H	4.16310	1.77560	0.34790
H	5.42730	0.65330	−0.09540
H	0.24520	−2.42490	−0.80620
H	1.18040	−1.56490	2.50020
H	0.37650	−0.03010	2.38400
H	4.72410	3.19570	−1.69490
H	−0.83570	−2.70210	1.49930
H	−1.48730	−0.99740	3.99050
H	−2.70610	−1.42230	1.31690
H	7.33890	1.72560	−1.10430
H	6.63710	4.30810	−2.64860
H	8.59550	3.80780	−4.07940
H	8.88580	2.30820	−3.20380
H	7.42450	2.49620	−4.17400
H	−1.10860	−4.85720	5.00960
H	7.14960	5.86190	−0.91410
H	8.59850	5.59370	0.05210
H	7.21740	4.51850	0.23940

**Marilzafurollene C_32**			
C	3.05640	−1.30920	−0.25520
O	1.87730	−0.58270	0.10220
C	4.09000	−0.35230	−0.87840
C	2.59430	−2.44280	−1.18290
C	0.71460	−1.36070	−0.17470
C	4.56490	0.72170	0.11560
Br	3.29940	0.54130	−2.47980
C	1.13910	−2.10590	−1.44200
C	0.38400	−2.24940	1.04870
C	5.66820	1.59300	−0.45280
Cl	0.15380	−3.54850	−1.81090
C	0.02370	−1.44450	2.31020
C	−0.48510	−2.34430	3.41900
O	1.17730	−0.76780	2.75920
C	−1.68580	−2.24080	3.94160
C	5.58800	2.92720	−0.58720
C	6.68400	3.80100	−1.16780
C	7.17180	4.82240	−0.13740
O	6.21900	4.53140	−2.29370
C	−2.89180	−2.13370	4.44670
Br	−4.44300	−3.11720	3.70810
C	5.95220	3.73310	−3.43940
H	3.47480	−1.75200	0.65030
H	4.94840	−0.92920	−1.22570
H	3.17070	−2.51790	−2.10550
H	2.67570	−3.39850	−0.66390
H	−0.13080	−0.70400	−0.38360
H	3.72180	1.34280	0.42160
H	4.94550	0.24690	1.02050
H	1.06580	−1.43140	−2.29660
H	−0.47160	−2.87720	0.79640
H	1.20240	−2.93680	1.26520
H	6.56760	1.08210	−0.76530
H	−0.73720	−0.70150	2.06000
H	0.20250	−3.09770	3.77460
H	1.59080	−0.38770	1.98850
H	4.69030	3.44300	−0.27720
H	7.53060	3.17850	−1.46410
H	7.56160	4.32850	0.75300
H	7.96960	5.43930	−0.55120
H	6.36510	5.48730	0.17220
H	−3.15370	−1.51570	5.29300
H	5.14580	3.02150	−3.25950
H	5.64720	4.37450	−4.26620
H	6.83970	3.18260	−3.75370

**Marilzafurollene C_33**			
C	2.92210	−0.60920	−1.06190
O	2.01510	−0.72670	0.03500
C	2.68090	0.70700	−1.82940
C	2.75040	−1.88560	−1.89600
C	1.32220	−1.97160	−0.03630
C	2.95090	1.97260	−0.99620
Br	0.78250	0.78060	−2.44010
C	2.23240	−2.87650	−0.87430
C	0.94970	−2.48680	1.36770
C	4.40630	2.13180	−0.59770
Cl	3.60150	−3.52550	0.07710
C	−0.06140	−1.58220	2.09410
C	−0.54310	−2.21100	3.38690
O	0.57000	−0.35430	2.38380
C	−1.79310	−2.54930	3.60830
C	5.21310	3.11020	−1.04080
C	6.66780	3.27360	−0.64100
C	6.89610	4.62220	0.04610
O	7.51980	3.23310	−1.77640
C	−3.03760	−2.90260	3.82810
Br	−4.36880	−1.67670	4.63100
C	7.65620	1.94440	−2.35900
H	3.93030	−0.61910	−0.64700
H	3.30220	0.72990	−2.72610
H	2.00100	−1.74720	−2.67550
H	3.67320	−2.20750	−2.37940
H	0.40690	−1.79740	−0.60530
H	2.64440	2.84890	−1.56880
H	2.33780	1.96640	−0.09420
H	1.70230	−3.71730	−1.32320
H	0.51960	−3.48380	1.26280
H	1.84250	−2.60530	1.98290
H	4.79540	1.39850	0.09440
H	−0.91290	−1.38700	1.43810
H	0.20880	−2.37130	4.14580
H	1.11030	−0.13410	1.62850
H	4.83180	3.84820	−1.73210
H	6.94910	2.48110	0.05550
H	7.93840	4.73280	0.34590
H	6.65270	5.45130	−0.61880
H	6.28130	4.71720	0.94150
H	−3.45270	−3.87320	3.59920
H	8.34600	1.99710	−3.20130
H	8.05880	1.22750	−1.64230
H	6.70430	1.56640	−2.73340

**Marilzafurollene C_34**			
C	2.78140	−0.67880	−1.63550
O	2.03380	−0.69180	−0.41870
C	2.40280	0.54620	−2.49310
C	2.53040	−2.03660	−2.30430
C	1.36590	−1.94280	−0.26820
C	2.77270	1.88710	−1.83610
Br	0.43410	0.54940	−2.81870
C	2.17290	−2.92200	−1.12840
C	1.20060	−2.31150	1.21900
C	4.26980	2.08400	−1.69350
Cl	3.66860	−3.46680	−0.31200
C	0.28320	−1.34020	1.98260
C	−0.00250	−1.83230	3.38800
O	0.92530	−0.08600	2.05300
C	−1.20290	−2.15240	3.81540
C	4.90770	2.21550	−0.51860
C	6.40470	2.41030	−0.36750
C	7.02260	1.26750	0.44200
O	6.70090	3.61770	0.31900
C	−2.39770	−2.48840	4.24130
Br	−3.63090	−1.19340	5.09080
C	6.44260	4.79610	−0.43200
H	3.83660	−0.62880	−1.36270
H	2.88380	0.47530	−3.46970
H	1.68190	−1.98480	−2.98680
H	3.38840	−2.39860	−2.87150
H	0.37790	−1.83530	−0.72000
H	2.38600	2.70870	−2.43960
H	2.28990	1.96730	−0.86090
H	1.60300	−3.80620	−1.41610
H	0.77920	−3.31600	1.27760
H	2.17200	−2.36110	1.71270
H	4.83720	2.11640	−2.61270
H	−0.65450	−1.21850	1.43550
H	0.85020	−1.90770	4.04690
H	1.35700	0.05280	1.21280
H	4.34290	2.18370	0.40260
H	6.87420	2.42700	−1.35310
H	8.10070	1.39800	0.53700
H	6.60370	1.22430	1.44760
H	6.84500	0.30430	−0.03720
H	−2.82010	−3.48020	4.17360
H	7.02060	4.80990	−1.35690
H	5.38520	4.89530	−0.67940
H	6.72880	5.67000	0.15320

**Marilzafurollene C_35**			
C	2.87200	−0.55490	−1.38500
O	2.02710	−0.49470	−0.23520
C	2.53020	0.58070	−2.37130
C	2.71860	−1.97190	−1.95270
C	1.39200	−1.75490	−0.02890
C	2.79830	1.98380	−1.80070
Br	0.59850	0.48080	−2.86140
C	2.29870	−2.76930	−0.73510
C	1.11930	−2.00330	1.46730
C	4.26780	2.24080	−1.52940
Cl	3.74250	−3.17970	0.23860
C	0.11450	−1.00580	2.07000
C	−0.26880	−1.38790	3.48590
O	0.70880	0.27370	2.08130
C	−1.49830	−1.67060	3.85140
C	4.78100	2.50610	−0.31560
C	6.24930	2.75890	−0.01660
C	6.49370	4.23780	0.29290
O	7.09190	2.40720	−1.10760
C	−2.72890	−1.96280	4.20000
Br	−3.44820	−3.80510	4.11020
C	7.38180	1.01770	−1.18840
H	3.89890	−0.44410	−1.03380
H	3.09760	0.45050	−3.29400
H	1.92520	−2.01020	−2.69930
H	3.62990	−2.34600	−2.41990
H	0.44070	−1.72590	−0.56380
H	2.45390	2.73830	−2.50850
H	2.21850	2.12930	−0.88810
H	1.78470	−3.69760	−0.98700
H	0.72440	−3.01400	1.58020
H	2.04870	−1.97550	2.03730
H	4.92580	2.20420	−2.38620
H	−0.77890	−0.96610	1.44260
H	0.53640	−1.41760	4.20540
H	1.20140	0.35680	1.26760
H	4.11950	2.54910	0.53830
H	6.52420	2.17540	0.86420
H	7.54190	4.41550	0.53360
H	6.23800	4.86530	−0.56130
H	5.89700	4.56820	1.14340
H	−3.45420	−1.24830	4.56120
H	8.00340	0.82490	−2.06260
H	7.93050	0.67990	−0.30840
H	6.47630	0.41750	−1.28300

**Marilzafurollene C_36**			
C	2.73330	−1.38920	−1.03770
O	2.20180	−1.58330	0.27270
C	2.88400	0.11020	−1.35310
C	1.81310	−2.16380	−1.98840
C	1.09170	−2.47580	0.22640
C	3.90380	0.79640	−0.42910
Br	1.11680	1.02080	−1.16520
C	1.26110	−3.24860	−1.08780
C	1.01800	−3.32930	1.50720
C	4.15670	2.24120	−0.81430
Cl	2.45500	−4.57470	−0.94880
C	0.65360	−2.51970	2.76470
C	−0.78870	−2.04900	2.75350
O	0.85130	−3.33970	3.89850
C	−1.14340	−0.78500	2.79350
C	5.34760	2.73180	−1.19640
C	5.59710	4.17850	−1.57970
C	6.09610	4.28170	−3.02320
O	6.58910	4.76620	−0.75060
C	−1.51780	0.47120	2.84420
Br	−1.82310	1.55700	1.21870
C	6.16340	5.01230	0.58280
H	3.71660	−1.86210	−1.05660
H	3.19950	0.22940	−2.39070
H	0.99400	−1.53570	−2.33890
H	2.33190	−2.55210	−2.86510
H	0.19140	−1.86520	0.13770
H	3.55080	0.76430	0.60250
H	4.84740	0.24970	−0.45090
H	0.32550	−3.67330	−1.45340
H	0.29500	−4.13440	1.37020
H	1.98390	−3.81160	1.65980
H	3.30130	2.90060	−0.76730
H	1.32280	−1.65990	2.84870
H	−1.53980	−2.82430	2.71510
H	1.78230	−3.42600	4.04930
H	6.20760	2.07920	−1.24260
H	4.66820	4.74630	−1.49590
H	6.26110	5.32240	−3.30240
H	7.03850	3.74960	−3.15500
H	5.37150	3.86170	−3.72120
H	−1.69520	1.02530	3.75430
H	5.30000	5.67850	0.60540
H	5.90540	4.08920	1.10290
H	6.96890	5.49150	1.13920

**Marilzafurollene C_37**			
C	2.84560	−1.69020	−1.14340
O	2.20090	−1.93010	0.10730
C	2.94570	−0.17670	−1.42230
C	2.06360	−2.49400	−2.19500
C	0.98980	−2.64220	−0.10700
C	3.75270	0.55530	−0.33650
Br	1.11940	0.62790	−1.53440
C	1.28000	−3.48380	−1.35520
C	0.53930	−3.41160	1.15050
C	3.95000	2.02620	−0.64680
Cl	2.31580	−4.89800	−0.99640
C	0.37360	−2.53250	2.40450
C	−0.58290	−1.37090	2.20500
O	−0.10740	−3.35460	3.44890
C	−0.28310	−0.13170	2.52400
C	5.14300	2.61630	−0.82710
C	5.33180	4.09050	−1.13210
C	6.05630	4.28310	−2.46660
O	6.11580	4.72570	−0.13280
C	0.01480	1.09110	2.89700
Br	0.91710	2.39520	1.71270
C	5.45010	4.88770	1.11260
H	3.85040	−2.10800	−1.06330
H	3.42180	−0.02190	−2.39160
H	1.36780	−1.85790	−2.74140
H	2.71000	−2.97380	−2.93030
H	0.22180	−1.91210	−0.36670
H	3.24410	0.46990	0.62480
H	4.72610	0.07860	−0.21530
H	0.37520	−3.84180	−1.84750
H	−0.39910	−3.92260	0.93230
H	1.26110	−4.19370	1.38470
H	3.04850	2.61910	−0.71710
H	1.35610	−2.15220	2.69480
H	−1.54430	−1.60940	1.77390
H	0.01120	−2.89930	4.27030
H	6.04860	2.03150	−0.75330
H	4.35750	4.57940	−1.19640
H	6.17760	5.34280	−2.69180
H	7.04880	3.83260	−2.44650
H	5.49750	3.83130	−3.28660
H	−0.19680	1.50210	3.87310
H	6.10500	5.41000	1.80990
H	4.54120	5.48020	1.00090
H	5.18760	3.92850	1.56020

**Marilzafurollene C_38**			
C	2.77370	−1.42840	−1.09550
O	2.22600	−1.63330	0.20640
C	2.91750	0.07400	−1.39970
C	1.87220	−2.20380	−2.06360
C	1.12120	−2.53090	0.14000
C	3.90910	0.76420	−0.44840
Br	1.13810	0.96760	−1.24860
C	1.31230	−3.29550	−1.17640
C	1.02450	−3.39340	1.41380
C	4.15860	2.21300	−0.82020
Cl	2.50980	−4.61760	−1.03130
C	0.67450	−2.59310	2.68100
C	−0.73820	−2.04050	2.64670
O	0.79510	−3.46020	3.79060
C	−1.02510	−0.76490	2.77490
C	5.35410	2.71570	−1.17080
C	5.60020	4.16660	−1.54070
C	6.13460	4.28130	−2.97060
O	6.56560	4.75900	−0.68400
C	−1.33180	0.50220	2.92240
Br	−1.49460	1.74600	1.39260
C	6.10450	4.99340	0.63980
H	3.76060	−1.89390	−1.10270
H	3.25580	0.20140	−2.42910
H	1.05490	−1.57950	−2.42450
H	2.40670	−2.58560	−2.93370
H	0.22090	−1.92220	0.04010
H	3.53200	0.72370	0.57430
H	4.85750	0.22540	−0.45020
H	0.38360	−3.72190	−1.55700
H	0.28660	−4.18270	1.26530
H	1.97860	−3.89700	1.57230
H	3.29650	2.86470	−0.79080
H	1.39670	−1.78180	2.80040
H	−1.52790	−2.76490	2.50860
H	0.84120	−2.93780	4.57880
H	6.22060	2.07080	−1.19920
H	4.66440	4.72560	−1.47770
H	6.29720	5.32490	−3.24060
H	7.08480	3.75830	−3.08100
H	5.43170	3.85830	−3.68870
H	−1.52420	0.98370	3.87000
H	5.23400	5.65060	0.64440
H	5.84340	4.06480	1.14850
H	6.89100	5.47780	1.21840

**Marilzafurollene C_39**			
C	2.96130	−0.97930	−0.96510
O	1.54550	−0.80900	−0.86580
C	3.49310	−0.21910	−2.19660
C	3.20500	−2.49600	−0.99020
C	0.94440	−2.00370	−0.37340
C	3.23220	1.29760	−2.15670
Br	2.63240	−0.94260	−3.84420
C	1.80290	−3.07140	−1.05500
C	0.97650	−2.00320	1.17450
C	3.90860	1.99430	−0.99100
Cl	1.68730	−4.68960	−0.30950
C	0.17750	−0.84770	1.80400
C	0.07430	−0.99590	3.30910
O	0.82950	0.36730	1.50400
C	−1.06720	−1.11520	3.94790
C	4.92260	2.86700	−1.11070
C	5.59770	3.56520	0.05520
C	5.47250	5.08540	−0.07270
O	6.98410	3.26130	0.10160
C	−2.21390	−1.24470	4.57200
Br	−3.04540	−3.00930	4.90910
C	7.27730	1.93390	0.51520
H	3.41850	−0.58070	−0.05850
H	4.56530	−0.39380	−2.29960
H	3.82410	−2.82790	−1.82410
H	3.70120	−2.80400	−0.06910
H	−0.08960	−2.06770	−0.71470
H	3.57860	1.74490	−3.08920
H	2.16040	1.49260	−2.10360
H	1.49570	−3.16660	−2.09780
H	0.54620	−2.94170	1.52670
H	2.00200	−1.99130	1.54500
H	3.52780	1.75820	−0.00700
H	−0.82320	−0.81990	1.36670
H	1.00760	−0.99810	3.85290
H	1.04510	0.33810	0.57550
H	5.30940	3.10950	−2.09020
H	5.12510	3.25910	0.99070
H	5.94510	5.58540	0.77290
H	5.95260	5.44690	−0.98230
H	4.42700	5.39360	−0.09840
H	−2.80420	−0.42490	4.95460
H	8.35770	1.79480	0.55240
H	6.88070	1.73390	1.51140
H	6.87160	1.19500	−0.17670

**Marilzafurollene C_40**			
C	3.12920	−0.93470	−0.14720
O	2.07000	−1.03180	0.80510
C	3.79880	0.44770	−0.05190
C	2.47620	−1.24410	−1.49860
C	0.90200	−1.59290	0.20610
C	4.98720	0.61170	−1.01640
Br	4.45140	0.69720	1.81680
C	1.37800	−2.21290	−1.11360
C	0.17020	−2.53420	1.18270
C	5.57160	2.01070	−0.98250
Cl	2.07280	−3.84680	−0.89130
C	−0.38920	−1.80850	2.41890
C	−1.27790	−2.71940	3.24250
O	0.69160	−1.37600	3.21640
C	−2.54760	−2.47580	3.47420
C	5.61260	2.84600	−2.03390
C	6.19880	4.24510	−1.99690
C	5.13570	5.29070	−2.34310
O	7.24910	4.38690	−2.94200
C	−3.81760	−2.22540	3.68770
Br	−5.24410	−2.86580	2.47340
C	8.44200	3.69600	−2.59640
H	3.85450	−1.71430	0.09180
H	3.05800	1.22750	−0.23450
H	2.03540	−0.34150	−1.92400
H	3.17420	−1.65500	−2.22880
H	0.24470	−0.75690	−0.03940
H	5.77270	−0.10270	−0.76710
H	4.67550	0.38740	−2.03700
H	0.58620	−2.28320	−1.86030
H	−0.65520	−3.00610	0.64740
H	0.82710	−3.34450	1.50150
H	5.97790	2.33290	−0.03370
H	−0.95590	−0.93080	2.09980
H	−0.80830	−3.60490	3.64540
H	1.38250	−1.08020	2.62730
H	5.21150	2.53160	−2.98680
H	6.57740	4.45880	−0.99520
H	5.55420	6.29640	−2.30220
H	4.74120	5.13640	−3.34770
H	4.30070	5.25010	−1.64320
H	−4.20310	−1.66050	4.52390
H	8.28500	2.61810	−2.54270
H	9.20280	3.88230	−3.35440
H	8.83420	4.03970	−1.63840

**Marilzafurollene C_41**			
C	3.17430	−1.84750	−0.76670
O	2.39960	−2.06540	0.41190
C	4.00660	−0.55900	−0.62380
C	2.18600	−1.89540	−1.94070
C	1.03040	−2.25650	0.06590
C	3.18350	0.70020	−0.29280
Br	5.04410	−0.26420	−2.30110
C	1.06200	−2.74580	−1.38580
C	0.32380	−3.17530	1.08090
C	4.05700	1.90410	−0.00170
Cl	1.49700	−4.47730	−1.50610
C	0.17330	−2.53960	2.47520
C	−0.79830	−1.37370	2.47810
O	−0.29810	−3.52400	3.37210
C	−0.45280	−0.13770	2.75870
C	3.98900	3.06880	−0.66710
C	4.86760	4.27240	−0.38440
C	4.02190	5.48030	0.02610
O	5.60990	4.65030	−1.53490
C	−0.11850	1.10420	3.01770
Br	0.45250	2.36610	1.60320
C	6.62420	3.72560	−1.90540
H	3.85450	−2.69580	−0.85810
H	4.73740	−0.72180	0.16950
H	1.80530	−0.90300	−2.17830
H	2.62770	−2.30370	−2.85020
H	0.55560	−1.27400	0.06930
H	2.49300	0.93930	−1.10080
H	2.57520	0.51520	0.59280
H	0.11540	−2.59760	−1.90670
H	−0.65940	−3.45370	0.69920
H	0.88980	−4.10310	1.16820
H	4.77310	1.79120	0.79940
H	1.15220	−2.20440	2.82650
H	−1.82360	−1.61110	2.23470
H	0.40810	−4.12710	3.55810
H	3.27370	3.18670	−1.46820
H	5.55570	4.04010	0.43080
H	4.65470	6.33990	0.24700
H	3.33440	5.76950	−0.76910
H	3.43230	5.26300	0.91720
H	−0.12210	1.55230	4.00060
H	7.17270	4.11260	−2.76410
H	7.33740	3.57420	−1.09430
H	6.20820	2.75810	−2.18900

**Marilzafurollene C_42**			
C	2.63480	−0.55730	−1.27560
O	2.22180	−0.88720	0.05030
C	1.85120	0.66430	−1.79990
C	2.46810	−1.83760	−2.10600
C	1.77290	−2.23730	0.09570
C	2.13970	1.95310	−1.01170
Br	−0.10930	0.30600	−1.68490
C	2.51950	−2.92490	−1.05140
C	1.94910	−2.84830	1.50000
C	3.57170	2.42890	−1.16050
Cl	4.22390	−3.27850	−0.63790
C	1.13780	−2.12130	2.58810
C	−0.35720	−2.17280	2.33280
O	1.41530	−2.73020	3.83260
C	−1.08280	−1.10360	2.09530
C	4.44580	2.53510	−0.14470
C	5.88700	3.00110	−0.26800
C	6.05700	4.40270	0.32310
O	6.32500	3.05350	−1.62060
C	−1.78450	−0.02480	1.84070
Br	−2.66110	1.03260	3.26640
C	6.72200	1.79420	−2.14770
H	3.69930	−0.32340	−1.22980
H	2.08210	0.82520	−2.85380
H	1.49540	−1.85840	−2.59810
H	3.23060	−1.94900	−2.87720
H	0.71330	−2.23400	−0.16410
H	1.48620	2.75170	−1.36370
H	1.90310	1.80150	0.04250
H	2.04730	−3.85470	−1.37020
H	1.66760	−3.90150	1.47340
H	3.00280	−2.82130	1.77840
H	3.88560	2.69590	−2.15970
H	1.47100	−1.08160	2.63630
H	−0.81260	−3.15240	2.34940
H	1.09560	−2.16430	4.52140
H	4.12620	2.27300	0.85410
H	6.51830	2.31280	0.29730
H	7.09570	4.72700	0.25910
H	5.44790	5.13130	−0.21260
H	5.76520	4.42760	1.37320
H	−1.94980	0.38390	0.85400
H	5.91480	1.06210	−2.10550
H	7.01300	1.91090	−3.19160
H	7.57990	1.39490	−1.60530

**Marilzafurollene C_43**			
C	3.59340	−1.73920	−1.69670
O	3.06760	−1.45850	−0.40020
C	5.05260	−1.25230	−1.84130
C	2.57360	−1.17620	−2.69350
C	1.74030	−0.93960	−0.51080
C	5.29280	0.27230	−1.83260
Br	5.78580	−1.97600	−3.54570
C	1.27450	−1.30850	−1.92490
C	0.85150	−1.41600	0.65380
C	4.79610	0.95660	−0.57440
Cl	0.67850	−2.99250	−2.02650
C	1.30760	−0.87240	2.01900
C	0.31740	−1.21580	3.11380
O	2.55760	−1.44880	2.32990
C	−0.32250	−0.31280	3.82110
C	3.94860	2.00160	−0.56660
C	3.39190	2.71180	0.66180
C	3.98410	2.22230	1.99070
O	1.98460	2.53400	0.75860
C	−0.97170	0.59280	4.51360
Br	−2.72650	1.30930	3.94240
C	1.22350	3.30100	−0.16410
H	3.59070	−2.82680	−1.78550
H	5.64150	−1.70790	−1.04390
H	2.76100	−0.12290	−2.89710
H	2.57080	−1.70660	−3.64620
H	1.81760	0.14690	−0.46750
H	6.36380	0.46160	−1.91040
H	4.84420	0.73540	−2.71130
H	0.48860	−0.65060	−2.29720
H	−0.16900	−1.07940	0.46520
H	0.81160	−2.50550	0.68570
H	5.16110	0.54240	0.35380
H	1.42840	0.21130	1.95670
H	0.15600	−2.26810	3.29600
H	3.04190	−1.50960	1.51000
H	3.61190	2.39300	−1.51500
H	3.62220	3.77450	0.56740
H	3.56770	2.78490	2.82630
H	3.76130	1.16950	2.16590
H	5.06620	2.35170	2.01670
H	−0.62420	1.02740	5.43940
H	1.42410	3.01400	−1.19620
H	0.16180	3.13820	0.02160
H	1.42090	4.36770	−0.05110

**Marilzafurollene C_44**			
C	3.25410	−1.49530	−0.65400
O	2.37830	−1.43800	0.47160
C	4.24350	−0.31460	−0.63120
C	2.34730	−1.60580	−1.88620
C	1.03050	−1.66890	0.05680
C	3.58710	1.07950	−0.62020
Br	5.46310	−0.46470	−2.20150
C	1.13640	−2.32320	−1.32710
C	0.24060	−2.45170	1.12440
C	4.59880	2.18930	−0.41190
Cl	1.46900	−4.07850	−1.22540
C	0.05510	−1.67040	2.43690
C	−0.87870	−2.39330	3.38750
O	1.31640	−1.52270	3.05150
C	−2.00830	−1.88190	3.82080
C	4.81300	3.19450	−1.27700
C	5.83540	4.29750	−1.07730
C	5.15700	5.66880	−1.02750
O	6.77100	4.33150	−2.14530
C	−3.14000	−1.36410	4.23590
Br	−4.84410	−1.61930	3.26120
C	7.66100	3.22330	−2.17840
H	3.81990	−2.42400	−0.56320
H	4.86830	−0.42090	0.25680
H	2.04860	−0.62370	−2.24950
H	2.81890	−2.13550	−2.71460
H	0.57020	−0.68810	−0.07580
H	3.04130	1.25690	−1.54640
H	2.85900	1.13900	0.18960
H	0.24010	−2.18560	−1.93310
H	−0.74250	−2.69010	0.71540
H	0.72110	−3.40850	1.33290
H	5.17550	2.14340	0.50090
H	−0.34020	−0.67640	2.21530
H	−0.56660	−3.37740	3.70520
H	1.95670	−1.40850	2.35370
H	4.24040	3.24540	−2.19200
H	6.36530	4.14040	−0.13570
H	4.43270	5.72080	−0.21430
H	5.89150	6.45870	−0.86910
H	4.63350	5.88480	−1.95910
H	−3.25390	−0.76300	5.12630
H	7.13320	2.28320	−2.34310
H	8.36990	3.35070	−2.99630
H	8.23090	3.14550	−1.25170

**Marilzafurollene C_45**			
C	2.65410	−0.61020	−0.60260
O	2.63040	−0.66130	0.82350
C	3.07830	0.78690	−1.09320
C	1.26680	−1.06670	−1.06730
C	1.35990	−1.11520	1.28250
C	4.52510	1.12380	−0.69570
Br	1.85290	2.17190	−0.33650
C	0.79680	−1.91350	0.09650
C	1.50930	−1.83330	2.64190
C	4.99940	2.43750	−1.28660
Cl	1.51670	−3.54730	−0.02810
C	0.18820	−2.10300	3.39160
C	−0.63870	−3.20210	2.75330
O	0.50800	−2.48750	4.71270
C	−1.87420	−3.03290	2.34060
C	6.03040	2.56260	−2.13880
C	6.50330	3.87900	−2.72690
C	6.40170	3.86140	−4.25420
O	7.86260	4.12920	−2.40050
C	−3.09070	−2.84420	1.88790
Br	−3.43760	−2.22750	0.03980
C	8.08330	4.45970	−1.03590
H	3.38500	−1.34830	−0.93680
H	2.98370	0.83020	−2.17930
H	0.59510	−0.21660	−1.18780
H	1.28840	−1.60780	−2.01370
H	0.74550	−0.22370	1.42150
H	4.60980	1.17520	0.39060
H	5.19220	0.32420	−1.02040
H	−0.28740	−2.01870	0.12000
H	2.08700	−2.75160	2.53660
H	2.12070	−1.18530	3.27130
H	4.45240	3.31890	−0.98230
H	−0.38940	−1.17730	3.44470
H	−0.15120	−4.16080	2.64310
H	−0.28270	−2.48390	5.23350
H	6.58370	1.68640	−2.44490
H	5.88170	4.69400	−2.35060
H	6.72790	4.81270	−4.67500
H	7.02640	3.07750	−4.68320
H	5.37510	3.68950	−4.57870
H	−3.99640	−2.99850	2.45640
H	9.14170	4.66750	−0.87870
H	7.52220	5.35000	−0.74920
H	7.80420	3.64200	−0.37050

**Marilzafurollene C_46**			
C	2.86230	−1.82290	−1.85700
O	2.56590	−1.64530	−0.47210
C	2.74690	−0.49870	−2.64090
C	1.92300	−2.93290	−2.34840
C	1.51790	−2.52490	−0.08030
C	3.87640	0.51260	−2.35700
Br	0.97060	0.35690	−2.31590
C	1.60420	−3.68690	−1.07380
C	1.61120	−2.88780	1.41480
C	3.80540	1.15490	−0.98350
Cl	2.94280	−4.81070	−0.69140
C	1.46430	−1.67300	2.34860
C	0.11910	−0.98760	2.20030
O	1.61310	−2.11590	3.68190
C	−0.01470	0.25270	1.78940
C	3.86600	2.47860	−0.76280
C	3.77970	3.12740	0.60520
C	2.69390	4.20550	0.62400
O	5.00150	3.76180	0.95350
C	−0.12820	1.49170	1.37470
Br	−0.15020	3.02470	2.62660
C	6.05800	2.86220	1.25970
H	3.88670	−2.19320	−1.92150
H	2.78300	−0.73560	−3.70540
H	1.00450	−2.50830	−2.75510
H	2.36860	−3.56040	−3.12070
H	0.57620	−2.00710	−0.26890
H	4.84510	0.02270	−2.46070
H	3.84920	1.29950	−3.11160
H	0.68160	−4.26490	−1.13550
H	0.84800	−3.62920	1.65400
H	2.57140	−3.36420	1.61420
H	3.70390	0.48450	−0.14260
H	2.26860	−0.96520	2.13300
H	−0.75000	−1.58040	2.44640
H	1.71410	−1.35890	4.24170
H	3.98100	3.15530	−1.59710
H	3.53080	2.37250	1.35410
H	2.59460	4.63920	1.61920
H	2.92700	5.01380	−0.06930
H	1.72510	3.79310	0.34280
H	−0.21940	1.79050	0.34000
H	6.32730	2.24610	0.40120
H	6.94320	3.42710	1.55190
H	5.79120	2.20690	2.08980

**Marilzafurollene C_47**			
C	3.16910	−1.82410	−0.78770
O	2.39230	−2.03630	0.39010
C	4.00940	−0.54090	−0.64860
C	2.18080	−1.87160	−1.96070
C	1.03330	−2.28980	0.04070
C	3.19470	0.72850	−0.33640
Br	5.06360	−0.26960	−2.31950
C	1.08070	−2.75990	−1.41770
C	0.36360	−3.24760	1.04570
C	4.07870	1.92450	−0.04310
Cl	1.56270	−4.47710	−1.56290
C	0.19500	−2.64110	2.45060
C	−0.79950	−1.49490	2.46700
O	−0.26540	−3.65690	3.31800
C	−0.47950	−0.25720	2.76950
C	4.05020	3.07700	−0.73250
C	4.94210	4.27070	−0.44840
C	4.10740	5.50160	−0.08680
O	5.72360	4.61220	−1.58420
C	−0.16720	0.98790	3.04060
Br	0.38860	2.26900	1.63780
C	6.73230	3.66360	−1.90610
H	3.84480	−2.67630	−0.87660
H	4.73210	−0.70310	0.15230
H	1.77480	−0.88450	−2.17700
H	2.62960	−2.25210	−2.87880
H	0.51670	−1.32870	0.05360
H	2.51830	0.96900	−1.15580
H	2.57250	0.55550	0.54190
H	0.13060	−2.63120	−1.93730
H	−0.60930	−3.55800	0.66290
H	0.96270	−4.15550	1.12350
H	4.77000	1.81540	0.78000
H	1.17000	−2.30200	2.80890
H	−1.81820	−1.74690	2.20910
H	−0.11330	−3.38470	4.21200
H	3.36030	3.19090	−1.55630
H	5.60230	4.04300	0.39080
H	4.74950	6.35420	0.13480
H	3.44820	5.78670	−0.90710
H	3.48900	5.31350	0.79140
H	−0.18290	1.42930	4.02650
H	7.31140	4.02330	−2.75640
H	7.41950	3.51800	−1.07170
H	6.30820	2.69730	−2.18130

**Marilzafurollene C_48**			
C	2.83930	−1.67070	−1.10430
O	2.19210	−1.90090	0.14700
C	2.93590	−0.15940	−1.39790
C	2.06270	−2.48750	−2.15010
C	0.97570	−2.60380	−0.06530
C	3.72820	0.58780	−0.31150
Br	1.10830	0.63790	−1.53900
C	1.26600	−3.46090	−1.30310
C	0.51990	−3.35730	1.19970
C	3.92310	2.05590	−0.63580
Cl	2.28780	−4.87960	−0.92170
C	0.34650	−2.45930	2.43950
C	−0.62490	−1.31260	2.22700
O	−0.11530	−3.26200	3.50750
C	−0.33510	−0.06470	2.52030
C	5.11560	2.64790	−0.81350
C	5.30200	4.11940	−1.13250
C	6.03310	4.30040	−2.46510
O	6.07910	4.76690	−0.13560
C	−0.04380	1.16500	2.87430
Br	0.83250	2.46300	1.66360
C	5.40650	4.93960	1.10460
H	3.84530	−2.08390	−1.01690
H	3.42180	−0.01290	−2.36370
H	1.37540	−1.85710	−2.71340
H	2.71360	−2.98180	−2.87170
H	0.21340	−1.87130	−0.33440
H	3.20930	0.51130	0.64500
H	4.70200	0.11620	−0.17440
H	0.36130	−3.81690	−1.79680
H	−0.41780	−3.87070	0.98340
H	1.24260	−4.13530	1.44360
H	3.02030	2.64510	−0.71920
H	1.32110	−2.05270	2.72080
H	−1.58880	−1.56990	1.81390
H	0.60780	−3.78180	3.82940
H	6.02240	2.06680	−0.72700
H	4.32690	4.60510	−1.20700
H	6.15300	5.35810	−2.70020
H	7.02660	3.85260	−2.43520
H	5.47980	3.83900	−3.28340
H	−0.24770	1.58480	3.84830
H	6.05630	5.47070	1.80000
H	4.49650	5.52840	0.98210
H	5.14420	3.98420	1.56050

**Marilzafurollene C_49**			
C	3.13200	−1.20910	−1.43660
O	1.78110	−0.82330	−1.17240
C	3.67030	−0.42760	−2.65220
C	3.10990	−2.73530	−1.61040
C	1.03420	−1.94660	−0.71410
C	3.68260	1.09670	−2.44160
Br	2.54060	−0.81960	−4.24880
C	1.63030	−3.06730	−1.56840
C	1.21100	−2.10180	0.81620
C	4.58130	1.52830	−1.29870
Cl	1.30960	−4.71040	−0.94750
C	0.68670	−0.89750	1.61890
C	0.68940	−1.17730	3.10900
O	1.51800	0.21180	1.35730
C	−0.40000	−1.21510	3.84220
C	4.14540	2.14030	−0.18490
C	5.03160	2.56420	0.97080
C	4.58820	1.88560	2.26920
O	4.96590	3.96690	1.18490
C	−1.48930	−1.26920	4.57190
Br	−2.24560	0.31980	5.47890
C	5.60070	4.74280	0.17790
H	3.73090	−0.97680	−0.55460
H	4.67900	−0.76880	−2.88980
H	3.58170	−3.07920	−2.53130
H	3.63090	−3.20760	−0.77680
H	−0.02280	−1.80880	−0.94490
H	4.02880	1.59080	−3.34980
H	2.66610	1.45220	−2.26650
H	1.21510	−3.00900	−2.57600
H	0.65990	−2.98700	1.13610
H	2.25330	−2.29410	1.07280
H	5.63470	1.31470	−1.41070
H	−0.32670	−0.65580	1.29040
H	1.65670	−1.34610	3.55900
H	1.66910	0.22690	0.41580
H	3.09280	2.35750	−0.06830
H	6.06450	2.27540	0.76660
H	5.23020	2.17590	3.10060
H	3.56420	2.15860	2.52730
H	4.62690	0.79970	2.17950
H	−2.06980	−2.16290	4.74790
H	5.12570	4.61100	−0.79480
H	5.53470	5.79930	0.43760
H	6.65710	4.48640	0.08860

**Marilzafurollene C_50**			
C	3.18690	−1.51850	−1.02500
O	2.36450	−1.39490	0.13600
C	3.71270	−0.14000	−1.46600
C	2.34050	−2.25400	−2.07250
C	1.09050	−1.98940	−0.10450
C	4.65820	0.47960	−0.42300
Br	2.17650	1.10120	−1.76650
C	1.34460	−3.01120	−1.21820
C	0.47330	−2.53380	1.19820
C	5.29620	1.76690	−0.90740
Cl	2.10320	−4.50890	−0.60010
C	0.14280	−1.42790	2.21580
C	−0.62530	−1.97460	3.40280
O	1.35300	−0.86200	2.66940
C	−1.83300	−1.57470	3.72970
C	5.13740	2.96440	−0.31760
C	5.76540	4.26670	−0.78600
C	4.71010	5.18020	−1.41410
O	6.78190	4.05970	−1.75970
C	−3.04400	−1.17470	4.03750
Br	−4.66610	−2.04270	3.30640
C	8.03880	3.68330	−1.21230
H	4.02850	−2.15910	−0.75600
H	4.23640	−0.24150	−2.41780
H	1.80610	−1.54750	−2.70800
H	2.93100	−2.90080	−2.72200
H	0.44110	−1.20910	−0.50630
H	4.11760	0.65720	0.50780
H	5.46050	−0.22010	−0.18700
H	0.43870	−3.28750	−1.75890
H	−0.44470	−3.06650	0.94560
H	1.13380	−3.26970	1.65840
H	5.91300	1.69110	−1.79160
H	−0.44150	−0.64580	1.72540
H	−0.12190	−2.72980	3.98850
H	1.93520	−0.81140	1.91450
H	4.51140	3.03990	0.56020
H	6.18590	4.77650	0.08290
H	5.15240	6.12500	−1.73040
H	4.25960	4.71340	−2.29050
H	3.91140	5.40650	−0.70740
H	−3.27260	−0.35450	4.70220
H	8.75230	3.51560	−2.01890
H	8.43890	4.47040	−0.57200
H	7.97090	2.76400	−0.62980

**Marilzafurollene C_51**			
C	2.81520	−0.55230	−1.19740
O	1.98150	−0.56540	−0.03770
C	2.56790	0.72240	−2.03030
C	2.54030	−1.86930	−1.93530
C	1.23650	−1.78080	0.01350
C	2.95040	2.02390	−1.30350
Br	0.63280	0.84040	−2.50410
C	2.05270	−2.77330	−0.82190
C	0.93780	−2.19250	1.46810
C	4.44010	2.14950	−1.04610
Cl	3.45500	−3.42260	0.07970
C	0.01600	−1.20010	2.19810
C	−0.40470	−1.72620	3.55610
O	0.71350	0.01430	2.36900
C	−1.65520	−1.94930	3.89010
C	5.23390	3.08440	−1.59700
C	6.72730	3.22900	−1.35080
C	7.52290	2.84080	−2.59960
O	7.18450	2.40440	−0.28560
C	−2.90780	−2.17840	4.20590
Br	−3.78000	−3.92660	3.88650
C	6.96370	2.95290	1.00750
H	3.84870	−0.57750	−0.85060
H	3.12070	0.66170	−2.96910
H	1.74730	−1.74670	−2.67310
H	3.41640	−2.25880	−2.45440
H	0.29310	−1.60160	−0.50590
H	2.62260	2.87620	−1.89970
H	2.42100	2.09260	−0.35240
H	1.46120	−3.61580	−1.18190
H	0.46000	−3.17330	1.45470
H	1.86390	−2.31430	2.03120
H	4.87030	1.42640	−0.36770
H	−0.86810	−1.00750	1.58580
H	0.39190	−1.91480	4.26100
H	1.21890	0.15820	1.57200
H	4.80590	3.80620	−2.27800
H	6.93740	4.27610	−1.12380
H	7.24380	3.45660	−3.45480
H	8.59250	2.96940	−2.43300
H	7.35150	1.79750	−2.86580
H	−3.57160	−1.45700	4.65960
H	5.90740	3.15070	1.19230
H	7.30930	2.25020	1.76550
H	7.51800	3.88310	1.13820

**Marilzafurollene C_52**			
C	3.17180	−1.08680	−1.24670
O	1.78160	−0.79180	−1.09310
C	3.70300	−0.43410	−2.53880
C	3.28700	−2.61790	−1.20100
C	1.11100	−1.90030	−0.50080
C	3.56960	1.09880	−2.54480
Br	2.69590	−1.14700	−4.10600
C	1.84050	−3.07360	−1.15840
C	1.22550	−1.81430	1.04080
C	4.36740	1.76420	−1.44020
Cl	1.63350	−4.63110	−0.31040
C	0.56920	−0.55410	1.63370
C	0.52460	−0.60780	3.14790
O	1.31760	0.57340	1.23410
C	−0.59110	−0.57940	3.84040
C	3.82310	2.45120	−0.42080
C	4.59360	3.11590	0.70730
C	4.55530	4.63950	0.56680
O	5.96150	2.72500	0.73170
C	−1.71380	−0.56120	4.51890
Br	−2.69240	−2.21450	4.99630
C	6.19240	1.47680	1.37160
H	3.70290	−0.68140	−0.38410
H	4.74900	−0.70740	−2.68560
H	3.82600	−3.04290	−2.04810
H	3.80950	−2.92000	−0.29260
H	0.05850	−1.89370	−0.78700
H	3.91670	1.49580	−3.49910
H	2.51820	1.37720	−2.46000
H	1.46900	−3.20080	−2.17660
H	0.73120	−2.68690	1.47000
H	2.26610	−1.87740	1.36040
H	5.44200	1.66320	−1.49810
H	−0.44410	−0.45410	1.23780
H	1.47850	−0.67100	3.65080
H	1.51630	0.45230	0.30870
H	2.74840	2.55610	−0.36690
H	4.11460	2.84720	1.65120
H	5.08540	5.11810	1.39040
H	5.02610	4.96030	−0.36280
H	3.53040	5.01120	0.57100
H	−2.20450	0.33240	4.87610
H	7.25300	1.23110	1.32090
H	5.63790	0.66720	0.89610
H	5.90940	1.51810	2.42420

**Marilzafurollene C_53**			
C	3.21590	−1.59080	−0.43800
O	1.95360	−1.13700	0.05540
C	4.03440	−0.39390	−0.95810
C	2.90110	−2.65720	−1.49800
C	0.92730	−2.06420	−0.28330
C	4.37260	0.60980	0.15750
Br	3.01650	0.54510	−2.39710
C	1.39720	−2.55090	−1.65570
C	0.82560	−3.14950	0.81540
C	5.27980	1.72710	−0.32010
Cl	0.64940	−4.08200	−2.18970
C	0.47270	−2.58980	2.20550
C	−0.90440	−1.95390	2.24180
O	0.51410	−3.65450	3.13320
C	−1.10880	−0.69080	2.53940
C	4.96530	3.03230	−0.27290
C	5.86480	4.15220	−0.76150
C	6.21840	5.10670	0.38170
O	5.22690	4.92370	−1.76910
C	−1.29390	0.57190	2.84350
Br	−1.58670	1.18350	4.70310
C	5.04720	4.24050	−3.00280
H	3.75410	−2.06670	0.38340
H	4.95820	−0.76090	−1.40750
H	3.42640	−2.50740	−2.44160
H	3.17420	−3.64310	−1.12020
H	−0.02630	−1.54400	−0.37640
H	3.45320	1.02750	0.57060
H	4.87580	0.09660	0.97750
H	1.16100	−1.79290	−2.40400
H	0.07480	−3.88750	0.53110
H	1.76690	−3.69510	0.88260
H	6.23630	1.42660	−0.72290
H	1.23010	−1.85860	2.49770
H	−1.73570	−2.60140	2.00220
H	0.45420	−3.29480	4.00730
H	4.00930	3.33780	0.12750
H	6.79080	3.73170	−1.15870
H	6.87700	5.90210	0.03250
H	5.32510	5.57400	0.79670
H	6.72990	4.58210	1.18900
H	−1.30500	1.38350	2.13050
H	6.00020	3.89970	−3.40910
H	4.38510	3.38030	−2.89770
H	4.59670	4.91590	−3.73000

**Marilzafurollene C_54**			
C	2.91050	−1.47990	−1.13510
O	2.25350	−1.78540	0.09470
C	3.15560	0.03640	−1.25590
C	2.04350	−2.08750	−2.24690
C	1.02800	−2.46020	−0.16730
C	4.13830	0.54900	−0.18960
Br	1.42540	1.02100	−1.07940
C	1.26200	−3.15750	−1.51100
C	0.61560	−3.36020	1.01400
C	4.48710	2.01260	−0.37680
Cl	2.26940	−4.62680	−1.34500
C	0.35450	−2.57820	2.31490
C	−0.79580	−1.59620	2.18910
O	0.06220	−3.50050	3.34490
C	−0.64150	−0.29330	2.25990
C	5.72030	2.47730	−0.64340
C	6.08800	3.93980	−0.83750
C	6.91290	4.45290	0.34540
O	4.94470	4.77880	−0.95110
C	−0.46260	1.00550	2.30930
Br	−0.55830	2.03070	4.00030
C	4.35550	4.78590	−2.24510
H	3.86830	−2.00260	−1.12470
H	3.55390	0.25850	−2.24710
H	1.35080	−1.34770	−2.64860
H	2.62850	−2.47780	−3.08010
H	0.26310	−1.69830	−0.32440
H	3.71220	0.41410	0.80550
H	5.05370	−0.04330	−0.21710
H	0.33550	−3.43520	−2.01470
H	−0.27600	−3.92610	0.74130
H	1.39970	−4.09490	1.19720
H	3.66740	2.71100	−0.28010
H	1.26200	−2.03710	2.59370
H	−1.77480	−2.02500	2.03390
H	0.87440	−3.88060	3.64900
H	6.54050	1.77920	−0.73110
H	6.69640	4.02300	−1.74000
H	7.20000	5.49380	0.19540
H	6.34450	4.39590	1.27410
H	7.82630	3.87160	0.47380
H	−0.25060	1.63030	1.45360
H	4.04070	3.78930	−2.55600
H	3.47420	5.42720	−2.24040
H	5.04920	5.17830	−2.98970

**Marilzafurollene C_55**			
C	3.07290	−1.15900	−0.81580
O	2.19380	−1.16960	0.30980
C	3.41690	0.28610	−1.22140
C	2.38290	−1.99020	−1.90540
C	1.03130	−1.94100	0.01220
C	4.22700	1.01580	−0.13660
Br	1.73450	1.30360	−1.57610
C	1.47460	−2.89700	−1.10080
C	0.45090	−2.59100	1.28310
C	4.70470	2.38180	−0.59050
Cl	2.41750	−4.27960	−0.46810
C	−0.07740	−1.56250	2.29830
C	−0.80440	−2.23520	3.44570
O	1.01850	−0.83530	2.80860
C	−2.06940	−2.02000	3.72650
C	5.99810	2.74100	−0.68600
C	6.53630	4.09610	−1.13360
C	5.45090	5.15690	−1.36540
O	7.25750	3.98780	−2.35360
C	−3.33680	−1.80460	3.98810
Br	−4.78560	−2.88490	3.18000
C	8.53230	3.37000	−2.24100
H	3.98430	−1.67930	−0.51680
H	3.98980	0.27350	−2.15030
H	1.77710	−1.35710	−2.55410
H	3.08540	−2.53410	−2.53740
H	0.29040	−1.25790	−0.40760
H	3.62270	1.13470	0.76360
H	5.08970	0.41180	0.14720
H	0.64020	−3.29250	−1.68130
H	−0.36850	−3.24800	0.98760
H	1.19390	−3.23000	1.76190
H	3.92380	3.08330	−0.84560
H	−0.74940	−0.86510	1.79270
H	−0.22030	−2.91980	4.04320
H	1.61610	−0.68600	2.07930
H	6.75160	2.01490	−0.41900
H	7.19900	4.46640	−0.34930
H	5.90010	6.11030	−1.64360
H	4.77610	4.86740	−2.17140
H	4.85870	5.32480	−0.46540
H	−3.70810	−1.03770	4.65230
H	8.45490	2.32800	−1.93070
H	9.02890	3.38710	−3.21110
H	9.16920	3.90090	−1.53240

**Marilzafurollene C_56**			
C	3.44010	−1.86900	−1.50710
O	2.82440	−1.34190	−0.33380
C	4.55750	−0.94670	−2.04270
C	2.28950	−2.19430	−2.46590
C	1.41000	−1.54220	−0.38720
C	4.14580	0.45800	−2.53050
Br	5.45520	−1.88190	−3.55520
C	1.17790	−2.56740	−1.50570
C	0.84920	−1.89490	1.00420
C	3.61210	1.35260	−1.42790
Cl	1.40240	−4.25120	−0.94320
C	1.00110	−0.75070	2.02230
C	0.30070	−1.07020	3.32800
O	2.37600	−0.55300	2.27000
C	−0.67740	−0.34490	3.82060
C	4.24570	2.43550	−0.94910
C	3.70790	3.32620	0.15520
C	3.56650	4.77120	−0.32950
O	4.58590	3.34150	1.27060
C	−1.66100	0.38250	4.29450
Br	−3.55530	0.02970	3.84040
C	4.55190	2.15280	2.04940
H	3.90170	−2.81070	−1.20480
H	5.31500	−0.83720	−1.26490
H	1.99210	−1.31700	−3.03840
H	2.53460	−2.98780	−3.17260
H	0.96960	−0.59860	−0.71380
H	5.01960	0.94130	−2.96930
H	3.40910	0.39840	−3.33040
H	0.18530	−2.49300	−1.95150
H	−0.21000	−2.13430	0.89780
H	1.32590	−2.79620	1.39200
H	2.65380	1.08050	−1.01230
H	0.59180	0.16920	1.59840
H	0.65050	−1.94620	3.85460
H	2.83700	−0.75820	1.45970
H	5.20740	2.71770	−1.35310
H	2.72380	2.97120	0.46820
H	3.16720	5.40710	0.46080
H	4.52970	5.18310	−0.63130
H	2.89010	4.83650	−1.18200
H	−1.54100	1.22380	4.96140
H	5.25090	2.24080	2.88090
H	3.55830	1.98600	2.46710
H	4.83650	1.27560	1.46720

**Marilzafurollene C_57**			
C	3.07980	−1.44730	−0.91600
O	2.32070	−1.76660	0.24960
C	3.16010	0.07940	−1.10610
C	2.42720	−2.21300	−2.07560
C	1.22640	−2.60650	−0.10200
C	3.94600	0.76090	0.02670
Br	1.31810	0.85190	−1.17920
C	1.69990	−3.33590	−1.36300
C	0.78180	−3.48860	1.08150
C	4.16270	2.24070	−0.22560
Cl	2.85140	−4.65410	−0.99240
C	0.29340	−2.67890	2.29670
C	−0.91800	−1.82210	1.97850
O	−0.03880	−3.58310	3.33040
C	−0.90000	−0.50910	2.01890
C	5.37050	2.82120	−0.34810
C	5.64910	4.29950	−0.60000
C	4.39650	5.18700	−0.57460
O	6.25750	4.50150	−1.86850
C	−0.85360	0.80180	2.04570
Br	−1.27640	1.84660	3.67310
C	7.61650	4.09420	−1.95060
H	4.08340	−1.84790	−0.76470
H	3.64180	0.29900	−2.06050
H	1.70210	−1.58680	−2.59600
H	3.14760	−2.56640	−2.81370
H	0.40040	−1.95660	−0.39480
H	3.41690	0.63760	0.97250
H	4.91390	0.27300	0.14810
H	0.87970	−3.75480	−1.94680
H	−0.00560	−4.16640	0.75050
H	1.61320	−4.11880	1.39790
H	3.26290	2.83260	−0.30680
H	1.11310	−2.04730	2.64790
H	−1.81990	−2.35030	1.70480
H	−0.12770	−3.09630	4.13790
H	6.24850	2.19900	−0.25760
H	6.31950	4.65040	0.18660
H	4.66550	6.23330	−0.72010
H	3.70000	4.91660	−1.36860
H	3.87480	5.11340	0.37990
H	−0.58670	1.42590	1.20490
H	7.72920	3.01870	−1.81440
H	8.00900	4.34270	−2.93670
H	8.23010	4.60820	−1.20970

**Marilzafurollene C_58**			
C	3.15280	−1.75130	−1.12350
O	2.41920	−1.91390	0.08990
C	3.42950	−0.25990	−1.39310
C	2.34300	−2.46800	−2.21320
C	1.20380	−2.60790	−0.17050
C	4.35980	0.35480	−0.33410
Br	1.70950	0.75740	−1.41220
C	1.50780	−3.44850	−1.41430
C	0.70450	−3.36930	1.07330
C	4.76250	1.77780	−0.66880
Cl	2.48700	−4.89620	−1.03190
C	0.38830	−2.44880	2.26640
C	−0.71300	−1.45150	1.95760
O	−0.01000	−3.25390	3.35720
C	−0.52030	−0.15210	1.93550
C	4.48910	2.84780	0.09740
C	4.88210	4.28120	−0.22010
C	3.65700	5.09760	−0.63850
O	5.82820	4.36250	−1.27950
C	−0.30130	1.14090	1.90060
Br	−0.51060	2.29600	3.49470
C	7.16890	4.11290	−0.87780
H	4.09950	−2.27940	−0.99900
H	3.88000	−0.15090	−2.38070
H	1.68370	−1.77000	−2.72980
H	2.96940	−2.94830	−2.96530
H	0.46030	−1.86270	−0.45730
H	3.87900	0.32090	0.64470
H	5.27160	−0.23760	−0.25220
H	0.60920	−3.77440	−1.93890
H	−0.18170	−3.94790	0.81040
H	1.45900	−4.09210	1.38450
H	5.30260	1.91550	−1.59470
H	1.30080	−1.91870	2.55010
H	−1.68820	−1.86510	1.74550
H	0.00520	−2.72450	4.14220
H	3.94000	2.70960	1.01800
H	5.30060	4.73010	0.68280
H	3.93130	6.13210	−0.84480
H	3.20240	4.68730	−1.54080
H	2.89920	5.10410	0.14520
H	0.00720	1.68990	1.02240
H	7.28340	3.12500	−0.43080
H	7.82530	4.16460	−1.74640
H	7.50820	4.85980	−0.15910

**Marilzafurollene C_59**			
C	2.62370	−1.22600	−1.92390
O	2.50200	−1.03820	−0.51130
C	1.78500	−0.19040	−2.70590
C	2.22520	−2.68560	−2.18380
C	1.98090	−2.21540	0.09400
C	2.38360	1.23010	−2.73490
Br	−0.06780	−0.10840	−1.96620
C	2.44230	−3.34050	−0.83440
C	2.40250	−2.32260	1.57170
C	2.68750	1.78850	−1.35850
Cl	4.17750	−3.72040	−0.62110
C	1.81980	−1.20200	2.45300
C	0.30490	−1.24150	2.52970
O	2.34770	−1.33340	3.75730
C	−0.46600	−0.28900	2.05570
C	3.87950	2.28500	−0.98910
C	4.20270	2.80070	0.39940
C	4.52570	4.29630	0.36610
O	5.33890	2.13810	0.93660
C	−1.21960	0.66340	1.55950
Br	−1.79040	2.23280	2.62260
C	5.12070	0.77100	1.25830
H	3.67790	−1.10800	−2.17980
H	1.68750	−0.52670	−3.73930
H	1.16970	−2.75190	−2.45050
H	2.79990	−3.15000	−2.98560
H	0.89290	−2.16530	0.03140
H	3.30160	1.22410	−3.32390
H	1.69600	1.90740	−3.24210
H	1.87250	−4.26150	−0.70670
H	2.09640	−3.29280	1.96490
H	3.49040	−2.29610	1.63590
H	1.87580	1.76050	−0.64510
H	2.13210	−0.23410	2.05440
H	−0.12640	−2.10990	3.00560
H	3.23680	−1.00910	3.76240
H	4.69060	2.32030	−1.70180
H	3.34510	2.64520	1.05730
H	4.74600	4.66740	1.36710
H	5.39350	4.49930	−0.26180
H	3.68570	4.87160	−0.02400
H	−1.59480	0.68710	0.54660
H	6.03360	0.34510	1.67410
H	4.33500	0.66710	2.00620
H	4.85010	0.17970	0.38270

**Marilzafurollene C_60**			
C	2.87300	−1.33870	−0.58140
O	1.66190	−1.08830	0.13460
C	3.31260	−0.06450	−1.32700
C	2.58940	−2.54500	−1.48920
C	0.80590	−2.22470	0.07330
C	3.62530	1.09400	−0.36500
Br	1.88020	0.50320	−2.59800
C	1.09420	−2.74430	−1.33670
C	1.15100	−3.19360	1.23010
C	4.19110	2.30580	−1.07980
Cl	0.59150	−4.43480	−1.61680
C	0.92310	−2.58840	2.62700
C	−0.54430	−2.32320	2.90670
O	1.41750	−3.49900	3.58790
C	−1.03110	−1.12800	3.15170
C	5.41030	2.82360	−0.85540
C	5.97260	4.03750	−1.57130
C	7.24810	3.67660	−2.33670
O	6.31260	5.06480	−0.65150
C	−1.52280	0.06860	3.36930
Br	−2.17840	1.22620	1.90420
C	5.19370	5.71490	−0.06380
H	3.64390	−1.61950	0.13810
H	4.19990	−0.28560	−1.92230
H	2.88170	−2.39140	−2.52830
H	3.12820	−3.41820	−1.11960
H	−0.23530	−1.91050	0.15250
H	2.72040	1.39090	0.16690
H	4.33520	0.76160	0.39330
H	0.56670	−2.12180	−2.06090
H	0.55590	−4.10270	1.13890
H	2.19050	−3.51060	1.14540
H	3.54580	2.76630	−1.81480
H	1.49630	−1.66210	2.71220
H	−1.19520	−3.18560	2.89180
H	1.46570	−3.05940	4.42500
H	6.05940	2.37050	−0.11990
H	5.23850	4.41560	−2.28570
H	7.64330	4.54600	−2.86250
H	8.02520	3.31530	−1.66270
H	7.05650	2.89980	−3.07730
H	−1.61840	0.53120	4.34080
H	5.54050	6.50650	0.60030
H	4.55790	6.17130	−0.82350
H	4.59000	5.02650	0.52870

**Marilzafurollene C_61**			
C	3.04920	−0.70770	−0.53300
O	2.15600	−0.21290	0.46440
C	3.68770	0.45010	−1.32280
C	2.23960	−1.70010	−1.37630
C	0.88550	−0.84890	0.34380
C	4.58200	1.33100	−0.43450
Br	2.25930	1.58310	−2.14010
C	1.17450	−2.15740	−0.40180
C	0.19660	−0.96010	1.71780
C	5.32260	2.39180	−1.22580
Cl	1.85010	−3.41760	0.67480
C	−1.15140	−1.70770	1.70530
C	−1.75220	−1.77360	3.09610
O	−2.05290	−1.04210	0.84490
C	−2.03240	−2.89780	3.71550
C	6.66000	2.48220	−1.31520
C	7.39770	3.54600	−2.10660
C	8.26140	2.91140	−3.19960
O	8.26540	4.29890	−1.27150
C	−2.30300	−4.01440	4.34940
Br	−0.98320	−4.92270	5.51160
C	7.59640	5.17940	−0.37870
H	3.83110	−1.26370	−0.01300
H	4.28290	0.04130	−2.14070
H	1.76380	−1.19910	−2.21950
H	2.84240	−2.51740	−1.77290
H	0.27870	−0.21460	−0.30520
H	3.97850	1.81990	0.33140
H	5.30560	0.70800	0.09280
H	0.29180	−2.56580	−0.89510
H	0.87280	−1.45240	2.41760
H	0.05110	0.04660	2.11100
H	4.70600	3.11390	−1.74270
H	−1.00310	−2.72190	1.32820
H	−1.94330	−0.82460	3.57680
H	−2.86580	−1.52740	0.81960
H	7.28240	1.76580	−0.79860
H	6.67800	4.21510	−2.58250
H	8.77950	3.67720	−3.77700
H	9.01620	2.25020	−2.77330
H	7.65420	2.32770	−3.89190
H	−3.24660	−4.53840	4.30320
H	8.33180	5.73970	0.19860
H	6.97970	5.89780	−0.92020
H	6.96430	4.63860	0.32650

**Marilzafurollene C_62**			
C	3.08900	−1.76840	−1.31150
O	2.35250	−1.59260	−0.10160
C	4.15430	−0.66590	−1.46250
C	2.04230	−1.86560	−2.42830
C	0.95390	−1.74570	−0.35070
C	3.60070	0.77140	−1.47020
Br	5.18730	−0.99260	−3.13660
C	0.85680	−2.46620	−1.70210
C	0.24290	−2.42240	0.83820
C	4.70290	1.81080	−1.41140
Cl	1.07720	−4.23520	−1.54680
C	0.25900	−1.57120	2.12010
C	−0.60120	−2.18580	3.20710
O	1.59030	−1.48260	2.57870
C	−1.66760	−1.60270	3.70600
C	4.85140	2.70880	−0.42330
C	5.95510	3.74850	−0.36570
C	6.81200	3.56450	0.88920
O	5.42070	5.06350	−0.31900
C	−2.74190	−1.03140	4.19730
Br	−2.68480	0.18080	5.76150
C	4.83900	5.50060	−1.53970
H	3.59490	−2.73210	−1.23230
H	4.86040	−0.76700	−0.63650
H	1.77240	−0.88040	−2.80580
H	2.37890	−2.46430	−3.27540
H	0.54670	−0.74140	−0.48180
H	3.00840	0.95440	−2.36600
H	2.93420	0.91360	−0.61840
H	−0.09110	−2.28940	−2.21180
H	−0.79330	−2.61110	0.55390
H	0.68290	−3.39940	1.04250
H	5.40330	1.80700	−2.23500
H	−0.09680	−0.56370	1.89310
H	−0.28830	−3.15410	3.56980
H	2.15120	−1.43720	1.80860
H	4.15360	2.71970	0.40180
H	6.60080	3.64780	−1.24050
H	7.61360	4.30250	0.92270
H	6.21660	3.68030	1.79520
H	7.27020	2.57540	0.91170
H	−3.74140	−1.16020	3.80860
H	5.56710	5.48660	−2.35170
H	4.48460	6.52510	−1.42730
H	3.98570	4.88450	−1.82550

**Marilzafurollene C_63**			
C	2.72440	−0.70000	−1.10440
O	2.22680	−1.06120	0.18370
C	2.08390	0.62070	−1.58120
C	2.46990	−1.90910	−2.01540
C	1.64820	−2.36100	0.13170
C	2.44890	1.83550	−0.70990
Br	0.09540	0.44290	−1.56690
C	2.37370	−3.05330	−1.02610
C	1.70430	−3.06460	1.50210
C	3.91820	2.20530	−0.78410
Cl	4.01790	−3.59230	−0.57150
C	0.91740	−2.32570	2.60020
C	−0.56330	−2.22020	2.28540
O	1.08250	−3.02720	3.81560
C	−1.17550	−1.07540	2.08360
C	4.39130	3.35440	−1.29760
C	5.85830	3.74540	−1.38030
C	6.34200	3.73170	−2.83260
O	6.69820	2.86170	−0.64740
C	−1.76240	0.07740	1.86540
Br	−2.59890	1.13450	3.31520
C	6.75050	3.13530	0.74700
H	3.80380	−0.57930	−1.00890
H	2.37700	0.82000	−2.61310
H	1.52120	−1.80750	−2.54270
H	3.24970	−2.05140	−2.76400
H	0.60600	−2.23900	−0.16680
H	1.85330	2.69330	−1.02410
H	2.18560	1.64250	0.33080
H	1.82950	−3.91390	−1.41650
H	1.32660	−4.08260	1.40100
H	2.74260	−3.15510	1.82180
H	4.61380	1.47850	−0.38900
H	1.34350	−1.32690	2.72330
H	−1.10820	−3.15140	2.22720
H	0.79150	−2.47130	4.52490
H	3.69660	4.08030	−1.69580
H	5.96450	4.76110	−0.99450
H	7.38650	4.03730	−2.89590
H	6.26160	2.73350	−3.26390
H	5.75850	4.41390	−3.45130
H	−1.84580	0.55280	0.89850
H	5.76410	3.08960	1.20940
H	7.38460	2.39810	1.23960
H	7.17660	4.12120	0.93690

**Marilzafurollene C_64**			
C	2.61480	−0.65670	−0.60850
O	2.59010	−0.72530	0.81700
C	3.03360	0.74810	−1.08120
C	1.22940	−1.11250	−1.08030
C	1.31790	−1.18070	1.26840
C	4.48040	1.08430	−0.68280
Br	1.80570	2.11860	−0.30230
C	0.75770	−1.96990	0.07500
C	1.46630	−1.90820	2.62290
C	4.94880	2.40760	−1.25690
Cl	1.48010	−3.60150	−0.06160
C	0.14500	−2.16280	3.37760
C	−0.71240	−3.23140	2.72790
O	0.45220	−2.57570	4.69330
C	−1.94100	−3.02210	2.31340
C	5.97740	2.54790	−2.10950
C	6.44440	3.87380	−2.68090
C	6.33930	3.87650	−4.20800
O	7.80370	4.12410	−2.35430
C	−3.15150	−2.79160	1.86430
Br	−3.48250	−2.15320	0.02040
C	8.02650	4.43720	−0.98600
H	3.34870	−1.38790	−0.95140
H	2.93620	0.80600	−2.16650
H	0.55640	−0.26260	−1.19550
H	1.25450	−1.64550	−2.03110
H	0.70400	−0.28970	1.41240
H	4.56800	1.12100	0.40380
H	5.14930	0.29150	−1.02040
H	−0.32640	−2.07690	0.09480
H	2.02760	−2.83560	2.50710
H	2.09420	−1.27300	3.24850
H	4.39950	3.28290	−0.93940
H	−0.41870	−1.22950	3.44700
H	−0.25510	−4.20420	2.61620
H	0.81720	−1.84300	5.16940
H	6.53300	1.67790	−2.42890
H	5.82100	4.68140	−2.29200
H	6.66110	4.83460	−4.61660
H	6.96550	3.10070	−4.64910
H	5.31250	3.70560	−4.53230
H	−4.05990	−2.92030	2.43480
H	9.08460	4.64640	−0.82850
H	7.46330	5.32190	−0.68620
H	7.75160	3.60990	−0.33080

**Marilzafurollene C_65**			
C	2.54570	0.24970	−0.77330
O	1.41350	−0.10080	0.02100
C	2.10580	1.11320	−1.97080
C	3.19790	−1.08570	−1.14250
C	1.47400	−1.47530	0.40340
C	3.24810	1.50780	−2.92760
Br	1.24340	2.77300	−1.28000
C	2.90210	−1.93310	0.07690
C	1.00530	−1.67090	1.85870
C	4.38830	2.22780	−2.23220
Cl	4.06450	−1.53600	1.37840
C	−0.47610	−1.31290	2.07150
C	−0.95220	−1.71810	3.45250
O	−0.62960	0.08180	1.92280
C	−1.92260	−2.57870	3.66010
C	5.65720	1.78800	−2.19990
C	6.79370	2.49510	−1.48640
C	7.88930	2.90210	−2.47480
O	7.39620	1.64800	−0.51880
C	−2.88560	−3.44860	3.85230
Br	−2.54320	−5.39160	4.01630
C	6.58480	1.39000	0.61960
H	3.22270	0.81640	−0.13250
H	1.33680	0.58310	−2.53410
H	2.71140	−1.51780	−2.01780
H	4.26350	−0.99640	−1.35670
H	0.79540	−2.01240	−0.26170
H	3.63450	0.61870	−3.42710
H	2.85990	2.15530	−3.71450
H	2.97040	−3.00400	−0.11840
H	1.15740	−2.71720	2.12810
H	1.62300	−1.08860	2.54360
H	4.13220	3.15180	−1.73270
H	−1.08320	−1.81030	1.31180
H	−0.44520	−1.25380	4.28570
H	−0.05750	0.35860	1.21010
H	5.91990	0.86660	−2.69930
H	6.41720	3.39620	−0.99820
H	8.69900	3.42210	−1.96260
H	8.31680	2.03120	−2.97220
H	7.49870	3.57050	−3.24250
H	−3.93340	−3.20030	3.93820
H	6.31300	2.31460	1.13030
H	5.67160	0.85790	0.35290
H	7.13420	0.76730	1.32550

**Marilzafurollene C_66**			
C	2.94620	−1.76560	−1.65930
O	2.71950	−1.46150	−0.28460
C	3.60900	−0.59620	−2.41950
C	1.60030	−2.26300	−2.19500
C	1.46020	−1.98820	0.13500
C	2.83550	0.73680	−2.47430
Br	3.95620	−1.18910	−4.28960
C	1.01920	−2.95040	−0.97680
C	1.56380	−2.58340	1.55240
C	2.73130	1.41860	−1.12460
Cl	1.74030	−4.57870	−0.79510
C	1.71750	−1.51430	2.64950
C	0.42910	−0.74590	2.87690
O	2.08100	−2.15130	3.85660
C	0.29460	0.54230	2.65910
C	3.40070	2.53010	−0.77820
C	3.30500	3.19650	0.58060
C	2.83470	4.64670	0.44880
O	4.56550	3.21540	1.23400
C	0.12700	1.82120	2.42220
Br	−0.46580	2.49680	0.65900
C	5.00500	1.94120	1.68550
H	3.64230	−2.60610	−1.67210
H	4.58830	−0.41050	−1.97570
H	0.95920	−1.43080	−2.48300
H	1.70060	−2.92380	−3.05660
H	0.75460	−1.15610	0.13720
H	3.35480	1.41120	−3.15610
H	1.83760	0.60340	−2.89000
H	−0.06370	−3.06820	−1.02920
H	0.68390	−3.19340	1.76170
H	2.41670	−3.26250	1.58090
H	2.07120	0.95540	−0.40580
H	2.52010	−0.82480	2.37620
H	−0.40700	−1.32480	3.24160
H	2.96730	−2.47470	3.77500
H	4.06510	3.00130	−1.48820
H	2.58830	2.65770	1.20150
H	1.85680	4.70000	−0.03080
H	2.74950	5.11700	1.42840
H	3.53350	5.23730	−0.14400
H	0.29240	2.61110	3.14050
H	5.95770	2.04750	2.20410
H	4.29170	1.50170	2.38400
H	5.15410	1.24630	0.85820

**Marilzafurollene C_67**			
C	2.80240	−1.46450	−1.15850
O	2.11830	−1.15930	0.05740
C	3.62170	−0.25220	−1.63880
C	1.72990	−1.95190	−2.14170
C	0.72280	−1.41350	−0.09210
C	4.76110	0.09820	−0.66640
Br	2.42430	1.33470	−1.83620
C	0.63890	−2.45130	−1.21680
C	0.07500	−1.80310	1.25110
C	5.64110	1.22100	−1.18060
Cl	1.04600	−4.09810	−0.64840
C	0.10400	−0.65910	2.29120
C	−0.68000	−0.91970	3.57050
O	1.44970	−0.40320	2.62860
C	−1.35940	−2.01030	3.84930
C	5.84260	2.38250	−0.53640
C	6.71150	3.51360	−1.05360
C	7.86660	3.79930	−0.09060
O	5.96830	4.71680	−1.18460
C	−2.04830	−3.09540	4.11260
Br	−3.95520	−3.28650	3.61670
C	5.00010	4.69600	−2.22540
H	3.47580	−2.29700	−0.94780
H	4.03690	−0.46710	−2.62450
H	1.34460	−1.12600	−2.74000
H	2.09510	−2.71530	−2.82920
H	0.26390	−0.49050	−0.45190
H	4.34800	0.36600	0.30730
H	5.39320	−0.77570	−0.50540
H	−0.34240	−2.48860	−1.69140
H	−0.96020	−2.08350	1.05150
H	0.55900	−2.68840	1.66540
H	6.12510	1.05140	−2.13170
H	−0.30920	0.24330	1.83740
H	−0.65090	−0.11450	4.29020
H	1.96170	−0.51090	1.82970
H	5.35980	2.55740	0.41450
H	7.13090	3.23940	−2.02360
H	8.49860	4.60190	−0.47090
H	7.49770	4.10360	0.88920
H	8.49400	2.91800	0.04480
H	−1.65630	−3.96970	4.61130
H	4.22600	3.94950	−2.04480
H	4.51180	5.66850	−2.28700
H	5.46230	4.49250	−3.19210

**Marilzafurollene C_68**			
C	2.94350	−1.52870	−1.07720
O	2.29970	−1.76860	0.17330
C	3.02730	−0.01620	−1.35830
C	2.16700	−2.34270	−2.12190
C	1.16240	−2.60220	−0.01840
C	3.92180	0.70990	−0.33950
Br	1.19770	0.78690	−1.30880
C	1.49290	−3.40730	−1.27940
C	0.83150	−3.41100	1.25260
C	4.11770	2.17530	−0.67710
Cl	2.64800	−4.73380	−0.95310
C	0.48240	−2.52720	2.46560
C	−0.74310	−1.66010	2.23400
O	0.27260	−3.35110	3.59620
C	−0.69060	−0.34870	2.17110
C	5.30420	2.74290	−0.95040
C	5.49750	4.21010	−1.28550
C	6.10250	4.37520	−2.68190
O	6.38410	4.83440	−0.36840
C	−0.61150	0.95790	2.08110
Br	−0.80380	2.14990	3.64960
C	5.84110	5.02340	0.93130
H	3.95120	−1.94080	−1.00270
H	3.42340	0.14190	−2.36270
H	1.40570	−1.73020	−2.60530
H	2.80550	−2.75310	−2.90460
H	0.32080	−1.95140	−0.26020
H	3.48680	0.63420	0.65800
H	4.89560	0.22110	−0.28980
H	0.61030	−3.83520	−1.75610
H	0.00860	−4.09310	1.03720
H	1.68680	−4.03410	1.51560
H	3.22380	2.78300	−0.68970
H	1.33960	−1.88630	2.68590
H	−1.68650	−2.17170	2.11400
H	−0.40170	−3.98490	3.40230
H	6.20270	2.14280	−0.93680
H	4.53300	4.72160	−1.26610
H	6.22750	5.43000	−2.92740
H	7.08210	3.90130	−2.74710
H	5.46170	3.93080	−3.44380
H	−0.43750	1.50150	1.16380
H	5.59900	4.07470	1.41150
H	6.57030	5.53520	1.55920
H	4.94030	5.63750	0.89780

**Marilzafurollene C_69**			
C	3.26720	−1.52910	−0.78610
O	2.39640	−1.83590	0.30210
C	3.51100	−0.00980	−0.87150
C	2.63210	−2.15850	−2.03450
C	1.25010	−2.52990	−0.17800
C	4.25640	0.51790	0.37230
Br	1.75930	0.93990	−1.04110
C	1.74050	−3.23450	−1.44670
C	0.62540	−3.42220	0.91300
C	4.73410	1.96310	0.31110
Cl	2.71900	−4.68700	−1.08120
C	0.15710	−2.63540	2.15110
C	−0.90780	−1.60560	1.82220
O	−0.36100	−3.55240	3.09290
C	−0.71960	−0.31210	1.95420
C	4.63390	2.80870	−0.73100
C	5.13690	4.23990	−0.72460
C	3.98870	5.22110	−0.97330
O	6.09780	4.45040	−1.74910
C	−0.49910	0.97550	2.07520
Br	−0.88010	1.96620	3.74600
C	7.35110	3.82410	−1.51190
H	4.21260	−2.03820	−0.59170
H	4.09190	0.19980	−1.77040
H	2.01750	−1.43290	−2.56750
H	3.36830	−2.54690	−2.73870
H	0.52140	−1.77840	−0.48540
H	3.61920	0.40260	1.25010
H	5.13710	−0.09880	0.55410
H	0.92830	−3.52990	−2.11160
H	−0.21310	−3.97580	0.48920
H	1.35200	−4.16860	1.23440
H	5.20400	2.31960	1.21680
H	1.02360	−2.14230	2.59860
H	−1.85100	−1.98930	1.46090
H	−0.44620	−3.11070	3.92620
H	4.17140	2.49370	−1.65400
H	5.58180	4.47010	0.24550
H	4.34670	6.25050	−0.95430
H	3.52480	5.04850	−1.94480
H	3.21580	5.12340	−0.21040
H	−0.09720	1.60740	1.29630
H	7.25930	2.73840	−1.46460
H	8.03650	4.06290	−2.32510
H	7.79990	4.17740	−0.58270

**Marilzafurollene C_70**			
C	3.05780	−1.28980	−1.09520
O	2.22280	−1.33590	0.06240
C	3.50750	0.15470	−1.38270
C	2.26070	−1.95600	−2.22430
C	0.98990	−1.98100	−0.25120
C	4.41570	0.71120	−0.27310
Br	1.90560	1.33330	−1.57190
C	1.30950	−2.85900	−1.46640
C	0.40580	−2.70130	0.97980
C	4.97130	2.08100	−0.61080
Cl	2.15490	−4.36730	−1.00680
C	0.00360	−1.73640	2.10910
C	−0.73330	−2.45690	3.22120
O	1.17640	−1.15300	2.63320
C	−1.97970	−2.20310	3.54980
C	6.27710	2.35890	−0.77030
C	6.85260	3.72420	−1.11180
C	7.59520	4.31520	0.08900
O	5.84970	4.65930	−1.49070
C	−3.22940	−1.96390	3.87100
Br	−3.74360	−0.61270	5.22350
C	5.41990	4.53580	−2.84040
H	3.93250	−1.90760	−0.88540
H	4.04330	0.18280	−2.33280
H	1.68670	−1.21790	−2.78490
H	2.89110	−2.49230	−2.93410
H	0.29320	−1.20430	−0.57230
H	3.86260	0.77840	0.66460
H	5.24380	0.02390	−0.09560
H	0.42390	−3.12870	−2.04290
H	−0.47540	−3.26050	0.66230
H	1.11100	−3.43970	1.36340
H	4.24350	2.87280	−0.72210
H	−0.62610	−0.94170	1.70250
H	−0.17080	−3.21050	3.75270
H	1.75290	−0.97410	1.89380
H	7.00410	1.56840	−0.65010
H	7.56690	3.60020	−1.92800
H	8.03100	5.28230	−0.16220
H	6.92070	4.46430	0.93270
H	8.40380	3.66140	0.41630
H	−4.08350	−2.46660	3.44140
H	5.01570	3.54530	−3.05120
H	4.63530	5.26530	−3.04090
H	6.24030	4.72990	−3.53240

**Marilzafurollene C_71**			
C	2.76660	−1.01860	−1.56530
O	1.60370	−0.93140	−0.73840
C	2.66460	−0.00460	−2.72320
C	2.86220	−2.48590	−2.01020
C	1.22570	−2.22640	−0.28380
C	2.62020	1.45830	−2.25020
Br	1.01090	−0.36360	−3.77970
C	1.56360	−3.08350	−1.50540
C	1.99950	−2.56750	1.01250
C	3.90240	1.89730	−1.56900
Cl	1.69410	−4.83160	−1.16500
C	1.70170	−1.60680	2.17830
C	0.26090	−1.69680	2.64620
O	2.55620	−1.94040	3.25280
C	−0.57150	−0.68050	2.63110
C	3.98650	2.25800	−0.27770
C	5.26330	2.69470	0.41550
C	5.60630	1.74790	1.56870
O	5.12950	3.99610	0.96790
C	−1.39800	0.33880	2.63220
Br	−1.58070	1.55630	4.18310
C	5.08860	5.03920	0.00320
H	3.63980	−0.79110	−0.95200
H	3.50650	−0.13840	−3.40400
H	2.98200	−2.61170	−3.08660
H	3.71450	−2.96280	−1.52470
H	0.15280	−2.25420	−0.09120
H	2.45360	2.11530	−3.10420
H	1.77180	1.60330	−1.57940
H	0.78520	−2.95360	−2.25890
H	1.76140	−3.58500	1.32430
H	3.07150	−2.56190	0.81380
H	4.79120	1.90930	−2.18410
H	1.93320	−0.58490	1.86770
H	−0.06520	−2.66440	3.00030
H	2.48670	−1.26560	3.91370
H	3.09830	2.24540	0.33840
H	6.09080	2.68370	−0.29700
H	6.53180	2.05190	2.05830
H	4.81850	1.74170	2.32270
H	5.73860	0.72550	1.21350
H	−2.04410	0.61040	1.81000
H	4.22400	4.94880	−0.65550
H	5.01860	6.00120	0.51090
H	5.99300	5.04860	−0.60690

**Marilzafurollene C_72**			
C	3.07250	−1.34250	−0.07040
O	2.08500	−0.52820	0.56770
C	4.31420	−0.48530	−0.38020
C	2.37890	−1.97770	−1.28650
C	0.78030	−0.89410	0.12040
C	4.05070	0.69770	−1.33080
Br	5.72810	−1.66210	−1.15050
C	1.05000	−1.25240	−1.34300
C	0.22930	−2.04070	1.00230
C	5.25990	1.60070	−1.47640
Cl	−0.23470	−2.22540	−2.11060
C	0.02100	−1.63720	2.47300
C	−0.71320	−2.71440	3.24690
O	1.28230	−1.42270	3.06740
C	−1.87520	−2.52320	3.82860
C	5.28810	2.89810	−1.12890
C	6.49900	3.80070	−1.27580
C	6.93770	4.34940	0.08420
O	6.21000	4.91720	−2.10450
C	−3.04100	−2.32110	4.39550
Br	−4.75060	−2.63040	3.44650
C	6.06840	4.59990	−3.48250
H	3.35530	−2.13410	0.62560
H	4.70880	−0.10300	0.56280
H	2.94230	−1.89310	−2.21570
H	2.22230	−3.04080	−1.09980
H	0.11040	−0.03570	0.18490
H	3.76730	0.34020	−2.32050
H	3.21160	1.28730	−0.95900
H	1.15730	−0.33580	−1.92470
H	−0.73710	−2.34860	0.60090
H	0.86850	−2.92230	0.94370
H	6.14690	1.14340	−1.89230
H	−0.54430	−0.70330	2.51540
H	−0.22530	−3.67650	3.30360
H	1.80260	−0.92290	2.44480
H	4.40480	3.36260	−0.71460
H	7.32980	3.23480	−1.70220
H	7.19270	3.54190	0.77100
H	7.81630	4.98620	−0.02020
H	6.14890	4.94560	0.54350
H	−3.18260	−1.97530	5.40910
H	6.97630	4.14290	−3.87800
H	5.23170	3.92290	−3.65860
H	5.88050	5.51230	−4.04830

**Marilzafurollene C_73**			
C	2.97320	−0.99810	−1.07340
O	2.63010	−0.69480	0.27860
C	3.82520	0.12640	−1.69160
C	1.65070	−1.28210	−1.79560
C	1.22170	−0.81350	0.46620
C	5.18570	0.27270	−0.98920
Br	2.84460	1.86310	−1.57730
C	0.75640	−1.74120	−0.66310
C	0.89820	−1.24760	1.90880
C	6.08100	1.29610	−1.66070
Cl	1.08180	−3.46450	−0.30340
C	−0.59840	−1.48680	2.18890
C	−0.82120	−1.94290	3.61770
O	−1.31780	−0.29160	1.96810
C	−1.37190	−3.09280	3.93410
C	6.57890	2.38480	−1.05100
C	7.46350	3.41810	−1.72310
C	8.83000	3.49600	−1.03790
O	6.88650	4.71420	−1.65510
C	−1.93960	−4.23520	4.24120
Br	−3.89670	−4.41730	4.47550
C	5.71840	4.87680	−2.44900
H	3.55460	−1.92140	−1.05460
H	3.98170	−0.08480	−2.75010
H	1.23980	−0.37080	−2.23050
H	1.74760	−2.01710	−2.59490
H	0.79680	0.17660	0.29070
H	5.03350	0.53980	0.05780
H	5.70920	−0.68390	−0.99220
H	−0.30530	−1.63290	−0.88750
H	1.45620	−2.15510	2.14110
H	1.27960	−0.48710	2.59110
H	6.31710	1.11560	−2.69950
H	−0.98200	−2.24730	1.50550
H	−0.49420	−1.26290	4.39160
H	−2.23700	−0.45630	2.12840
H	6.34420	2.56990	−0.01260
H	7.61690	3.14430	−2.76880
H	9.46970	4.22770	−1.53150
H	8.73160	3.79240	0.00670
H	9.34130	2.53350	−1.06790
H	−1.41290	−5.16620	4.39270
H	5.37090	5.90730	−2.37730
H	5.92090	4.66490	−3.49950
H	4.90610	4.23120	−2.11320

**Marilzafurollene C_74**			
C	3.22620	−1.21530	−0.71740
O	2.32190	−1.23710	0.38780
C	3.72630	0.21730	−0.98250
C	2.48450	−1.87050	−1.89010
C	1.09620	−1.85640	0.00180
C	4.57350	0.75500	0.18980
Br	2.15750	1.42250	−1.26960
C	1.46980	−2.74860	−1.18720
C	0.42180	−2.55550	1.19810
C	5.29370	2.07610	−0.04810
Cl	2.25340	−4.27130	−0.66920
C	−0.02660	−1.57390	2.29500
C	−0.84540	−2.26960	3.36430
O	1.12330	−1.01040	2.88690
C	−2.08920	−1.95330	3.64360
C	5.32430	2.79150	−1.18760
C	6.06800	4.10300	−1.35610
C	5.10480	5.22930	−1.73930
O	7.03960	4.01290	−2.38810
C	−3.33510	−1.63490	3.90370
Br	−4.86360	−2.48620	2.97720
C	8.16850	3.21490	−2.05910
H	4.07190	−1.85220	−0.45270
H	4.31770	0.21590	−1.89900
H	1.96170	−1.12360	−2.48790
H	3.14500	−2.42430	−2.55780
H	0.43800	−1.06650	−0.36520
H	3.94220	0.86150	1.07290
H	5.33760	0.02170	0.44960
H	0.61510	−3.00320	−1.81500
H	−0.45140	−3.09640	0.83040
H	1.08490	−3.30720	1.62800
H	5.83330	2.45430	0.80860
H	−0.61290	−0.76940	1.84500
H	−0.34730	−3.06070	3.90560
H	1.74980	−0.85620	2.18340
H	4.79870	2.45100	−2.06670
H	6.55660	4.37300	−0.41780
H	5.63610	6.17510	−1.84560
H	4.60770	5.01900	−2.68660
H	4.33470	5.36360	−0.97920
H	−3.64480	−0.89690	4.62930
H	8.68140	3.60060	−1.17710
H	7.89110	2.17640	−1.87540
H	8.87600	3.22670	−2.88800

**Marilzafurollene C_75**			
C	3.16660	−1.40920	−1.75320
O	2.37670	−0.78370	−0.73920
C	3.26880	−0.51050	−3.00410
C	2.51280	−2.77520	−2.00020
C	1.28780	−1.62640	−0.37370
C	4.23590	0.68170	−2.85690
Br	1.45870	0.16790	−3.50160
C	1.78330	−3.03710	−0.69730
C	0.85080	−1.35440	1.07730
C	3.97170	1.53960	−1.63350
Cl	2.93610	−3.63950	0.53050
C	0.19710	0.03660	1.25080
C	−0.35670	0.32830	2.63860
O	1.15250	1.02180	0.92550
C	−0.33670	−0.49440	3.66410
C	4.90020	1.84220	−0.71100
C	4.64740	2.66630	0.53660
C	5.38630	4.00470	0.46260
O	5.12860	1.99070	1.68950
C	−0.33280	−1.32570	4.67910
Br	−1.81070	−2.59980	5.01110
C	4.31450	0.90330	2.10660
H	4.16290	−1.57630	−1.34050
H	3.61340	−1.11320	−3.84590
H	1.78860	−2.71040	−2.81350
H	3.23160	−3.55410	−2.25610
H	0.45580	−1.40050	−1.04340
H	5.26010	0.30970	−2.80980
H	4.17870	1.31220	−3.74460
H	0.97590	−3.76380	−0.79430
H	0.13560	−2.12580	1.36650
H	1.70260	−1.45420	1.75150
H	2.96270	1.91100	−1.51940
H	−0.63150	0.12800	0.54650
H	−0.79680	1.30820	2.75310
H	1.70430	0.65620	0.23590
H	5.91050	1.47860	−0.82950
H	3.57830	2.86460	0.63970
H	5.19910	4.59940	1.35680
H	6.46340	3.85780	0.38050
H	5.05880	4.58750	−0.39860
H	0.45480	−1.39830	5.41480
H	4.78200	0.40230	2.95390
H	3.33520	1.25480	2.43150
H	4.17720	0.16330	1.31690

**Marilzafurollene C_76**			
C	2.78860	−1.86920	−1.44090
O	2.24870	−1.99970	−0.12590
C	3.25930	−0.42410	−1.69740
C	1.70690	−2.37830	−2.40590
C	0.89450	−2.42820	−0.19900
C	4.36540	0.00500	−0.71800
Br	1.71930	0.83780	−1.53320
C	0.82690	−3.22410	−1.50820
C	0.46070	−3.17960	1.07510
C	4.90000	1.39280	−1.01320
Cl	1.54290	−4.85660	−1.35180
C	0.56580	−2.34800	2.36670
C	−0.29590	−1.09900	2.34450
O	0.15630	−3.16900	3.44200
C	0.15870	0.09600	2.64810
C	4.90450	2.40600	−0.13100
C	5.41930	3.80200	−0.42700
C	6.62390	4.13860	0.45520
O	4.42700	4.78570	−0.17180
C	0.60810	1.27720	3.00210
Br	1.41360	2.55360	1.72340
C	3.30920	4.73360	−1.04880
H	3.64570	−2.54210	−1.49660
H	3.62700	−0.34520	−2.72120
H	1.11850	−1.55480	−2.80980
H	2.11630	−2.93180	−3.25130
H	0.27720	−1.53630	−0.31750
H	3.98750	−0.03440	0.30500
H	5.19830	−0.69670	−0.76930
H	−0.18980	−3.33490	−1.88640
H	−0.56330	−3.53280	0.94870
H	1.07490	−4.07160	1.19820
H	5.29510	1.55210	−2.00610
H	1.61190	−2.07450	2.52460
H	−1.32890	−1.23530	2.05890
H	0.43950	−2.76900	4.25200
H	4.50980	2.25140	0.86330
H	5.72750	3.86360	−1.47250
H	7.00210	5.13590	0.23040
H	6.35710	4.11520	1.51200
H	7.43790	3.43060	0.29750
H	0.58470	1.66570	4.00970
H	3.61700	4.83810	−2.08980
H	2.75110	3.80280	−0.94180
H	2.62790	5.55220	−0.81740

**Marilzafurollene C_77**			
C	3.39380	−1.80060	−1.83550
O	2.72630	−1.40060	−0.63750
C	3.68850	−0.58640	−2.74160
C	2.49480	−2.86830	−2.47400
C	1.49530	−2.10370	−0.49730
C	4.80190	0.35080	−2.22820
Br	2.01970	0.47060	−3.03300
C	1.70710	−3.39570	−1.29130
C	1.11750	−2.25580	0.98860
C	4.51850	0.95630	−0.86590
Cl	2.69680	−4.58390	−0.39210
C	0.79430	−0.91200	1.66720
C	0.29550	−1.11030	3.08500
O	1.96870	−0.13050	1.69390
C	−0.91000	−0.77420	3.48450
C	4.26400	2.25570	−0.64270
C	3.96650	2.84680	0.72250
C	2.57300	3.47830	0.74800
O	4.89940	3.86540	1.05450
C	−2.11800	−0.45560	3.88600
Br	−2.56180	1.32690	4.62380
C	6.19980	3.38270	1.36220
H	4.33310	−2.27300	−1.54370
H	3.99350	−0.95320	−3.72290
H	1.80840	−2.41510	−3.19020
H	3.05590	−3.64250	−2.99800
H	0.72500	−1.51840	−1.00330
H	5.74390	−0.19460	−2.16350
H	4.96020	1.15100	−2.95210
H	0.77280	−3.87910	−1.57890
H	0.24370	−2.90520	1.05720
H	1.91480	−2.76100	1.53530
H	4.52150	0.27010	−0.03110
H	0.04130	−0.38050	1.08080
H	0.99740	−1.55110	3.77770
H	2.41130	−0.27250	0.86010
H	4.25720	2.95070	−1.46980
H	3.99860	2.06000	1.47920
H	2.35280	3.89030	1.73280
H	2.48900	4.28570	0.02060
H	1.80560	2.73700	0.52130
H	−2.97200	−1.11630	3.85570
H	6.65680	2.87800	0.51040
H	6.84290	4.21920	1.63550
H	6.17510	2.69140	2.20550

**Marilzafurollene C_78**			
C	2.98720	−1.42420	−0.95950
O	1.85290	−1.24550	−0.11050
C	3.48180	−0.06780	−1.49500
C	2.56020	−2.42880	−2.03760
C	0.77270	−2.06100	−0.56120
C	4.02000	0.83660	−0.37350
Br	1.98730	0.87070	−2.43170
C	1.44280	−3.18920	−1.35360
C	−0.13140	−2.49060	0.61090
C	4.62930	2.11980	−0.90420
Cl	2.13950	−4.44370	−0.28530
C	−0.86540	−1.31220	1.27590
C	−1.91140	−1.79930	2.27290
O	0.09750	−0.46820	1.88210
C	−1.89730	−1.57740	3.56940
C	5.91450	2.47700	−0.74470
C	6.52060	3.76280	−1.27570
C	7.65420	3.46510	−2.26040
O	7.07260	4.54300	−0.22520
C	−1.86300	−1.35380	4.86180
Br	−0.99660	−2.59680	6.13750
C	6.10530	5.14560	0.62410
H	3.77130	−1.88470	−0.35610
H	4.27000	−0.23660	−2.23070
H	2.16300	−1.91590	−2.91380
H	3.37630	−3.06990	−2.37210
H	0.18700	−1.46140	−1.26070
H	3.21670	1.08970	0.31970
H	4.77080	0.29790	0.20620
H	0.76590	−3.67870	−2.05480
H	−0.86800	−3.20010	0.23160
H	0.44620	−3.02840	1.36400
H	3.95950	2.77530	−1.44330
H	−1.38620	−0.73270	0.51180
H	−2.72330	−2.36790	1.84330
H	0.86990	−0.47870	1.32100
H	6.58940	1.82870	−0.20450
H	5.75570	4.34180	−1.79710
H	8.07940	4.38930	−2.65210
H	8.45870	2.90710	−1.78070
H	7.29640	2.87930	−3.10750
H	−2.29550	−0.49220	5.34830
H	5.50280	4.40060	1.14490
H	6.60960	5.74940	1.37850
H	5.43970	5.80170	0.06180

**Marilzafurollene C_79**			
C	2.66780	−1.63830	−1.37520
O	2.27390	−1.75360	−0.00830
C	3.14720	−0.20960	−1.69030
C	1.47140	−2.12100	−2.20340
C	0.98240	−2.34900	0.08360
C	4.41350	0.16220	−0.90040
Br	1.70330	1.09930	−1.25590
C	0.78830	−3.07980	−1.25100
C	0.86820	−3.21520	1.35270
C	4.95680	1.52640	−1.27930
Cl	1.62680	−4.66050	−1.28990
C	0.86980	−2.39430	2.65490
C	−0.41580	−1.61190	2.84690
O	1.02500	−3.28040	3.74470
C	−0.46930	−0.30140	2.91670
C	5.12520	2.54220	−0.41660
C	5.65440	3.91290	−0.79480
C	6.95680	4.21990	−0.05160
O	4.72850	4.93560	−0.45650
C	−0.54750	1.00610	2.98800
Br	−0.84800	2.14060	1.39510
C	3.54050	4.93110	−1.23740
H	3.49190	−2.33680	−1.52980
H	3.34530	−0.12570	−2.75970
H	0.79520	−1.29680	−2.43110
H	1.76180	−2.58270	−3.14740
H	0.25530	−1.53620	0.12460
H	4.20360	0.12950	0.16990
H	5.19570	−0.57440	−1.08610
H	−0.26160	−3.24810	−1.49310
H	−0.03780	−3.82070	1.30460
H	1.70200	−3.91740	1.37030
H	5.21580	1.66530	−2.31910
H	1.72230	−1.71050	2.65150
H	−1.31850	−2.19870	2.93270
H	1.92380	−3.57790	3.76520
H	4.86650	2.40840	0.62400
H	5.85330	3.94540	−1.86780
H	7.34370	5.19850	−0.33600
H	6.80380	4.22470	1.02790
H	7.72440	3.47990	−0.27950
H	−0.46110	1.58070	3.89850
H	2.91250	5.77380	−0.94840
H	3.76440	5.03020	−2.30020
H	2.95900	4.02080	−1.08670

**Marilzafurollene C_80**			
C	2.80870	−1.21060	−0.85780
O	1.81980	−0.92190	0.13120
C	3.37350	0.09150	−1.45520
C	2.13990	−2.15050	−1.86930
C	0.60830	−1.60980	−0.17530
C	4.14730	0.91840	−0.41460
Br	1.88080	1.19730	−2.19060
C	1.04490	−2.79470	−1.04420
C	−0.18330	−1.95000	1.10290
C	4.81820	2.13530	−1.02180
Cl	1.73560	−4.12790	−0.07190
C	−0.69460	−0.69630	1.85000
C	−1.62310	−0.97010	3.02600
O	0.42340	0.02860	2.31390
C	−2.05210	−2.15120	3.41400
C	6.14440	2.34920	−1.03260
C	6.81250	3.56910	−1.63920
C	7.76770	3.16430	−2.76480
O	7.57950	4.26990	−0.67100
C	−2.49280	−3.32160	3.81130
Br	−1.52940	−4.42850	5.13990
C	6.80380	4.96080	0.29910
H	3.61000	−1.75980	−0.36060
H	4.03670	−0.15170	−2.28700
H	1.69040	−1.58730	−2.68750
H	2.83180	−2.87130	−2.30570
H	0.00810	−0.94230	−0.79660
H	3.47290	1.24760	0.37700
H	4.90410	0.29470	0.06310
H	0.23460	−3.20140	−1.65020
H	−1.03290	−2.56930	0.81230
H	0.42300	−2.55710	1.77680
H	4.15920	2.86650	−1.46880
H	−1.23930	−0.06200	1.14860
H	−1.93450	−0.08950	3.56890
H	1.11010	−0.06790	1.65720
H	6.80930	1.62440	−0.58520
H	6.05370	4.23500	−2.05510
H	8.23620	4.04240	−3.20950
H	8.56240	2.51580	−2.39520
H	7.23990	2.63270	−3.55700
H	−3.40180	−3.78940	3.46230
H	6.19810	4.27750	0.89530
H	7.46570	5.49610	0.97990
H	6.14490	5.69260	−0.16990

**Marilzafurollene C_81**			
C	2.95530	−1.77820	−1.70450
O	2.72520	−1.48590	−0.32810
C	3.61960	−0.60240	−2.45360
C	1.60970	−2.26970	−2.24610
C	1.47390	−2.03380	0.08950
C	2.84930	0.73290	−2.49140
Br	3.96040	−1.17540	−4.33120
C	1.03340	−2.97830	−1.03780
C	1.58390	−2.65400	1.49630
C	2.76220	1.40540	−1.13580
Cl	1.75760	−4.60840	−0.88680
C	1.73240	−1.60840	2.61620
C	0.44620	−0.83580	2.84260
O	2.07160	−2.28410	3.80960
C	0.32540	0.45880	2.65760
C	3.42970	2.51900	−0.79280
C	3.35240	3.17530	0.57220
C	2.86540	4.62170	0.45880
O	4.62450	3.20370	1.20270
C	0.16770	1.74390	2.44760
Br	−0.39720	2.46110	0.69190
C	5.09360	1.93010	1.62540
H	3.65100	−2.61880	−1.72380
H	4.60050	−0.42460	−2.01010
H	0.96630	−1.43430	−2.51910
H	1.70990	−2.91630	−3.11830
H	0.76180	−1.20750	0.10900
H	3.36350	1.41080	−3.17380
H	1.84670	0.60500	−2.89730
H	−0.04930	−3.09790	−1.08960
H	0.71000	−3.27580	1.69500
H	2.44300	−3.32580	1.51550
H	2.11660	0.93290	−0.40980
H	2.54910	−0.92750	2.36410
H	−0.40220	−1.41810	3.17320
H	2.40080	−1.65410	4.43490
H	4.07910	2.99960	−1.51030
H	2.65290	2.62480	1.20280
H	2.79290	5.08460	1.44300
H	3.54790	5.22310	−0.14200
H	1.87890	4.66860	−0.00340
H	0.32520	2.51730	3.18540
H	6.05210	2.04490	2.13140
H	4.39900	1.46580	2.32670
H	5.24270	1.25220	0.78410

**Marilzafurollene C_82**			
C	3.12110	−1.48920	−0.17580
O	2.13580	−1.93020	0.75770
C	3.51540	−0.03510	0.14130
C	2.49250	−1.69690	−1.56230
C	0.94540	−2.30940	0.07900
C	4.62060	0.50780	−0.78180
Br	4.16120	0.04270	2.02780
C	1.43190	−2.74810	−1.30380
C	0.14240	−3.34280	0.89400
C	4.91300	1.97440	−0.53100
Cl	2.18740	−4.36940	−1.27470
C	−0.32930	−2.80120	2.25670
C	−1.29230	−1.63660	2.11970
O	−0.96510	−3.84270	2.96920
C	−0.97750	−0.40400	2.44720
C	4.76800	2.94830	−1.44490
C	5.05950	4.41530	−1.18930
C	3.80260	5.26430	−1.39570
O	6.04830	4.90600	−2.08280
C	−0.61840	0.82600	2.72710
Br	0.22020	2.00570	1.37610
C	7.36000	4.42520	−1.82180
H	3.98740	−2.14310	−0.06430
H	2.63250	0.60350	0.08560
H	2.01230	−0.77850	−1.90290
H	3.21780	−1.99450	−2.32020
H	0.34680	−1.40700	−0.05550
H	5.53910	−0.06300	−0.64070
H	4.33320	0.38130	−1.82590
H	0.64010	−2.75480	−2.05370
H	−0.71770	−3.67500	0.31140
H	0.75910	−4.22590	1.06080
H	5.26110	2.22630	0.46110
H	0.54310	−2.48300	2.83310
H	−2.27190	−1.86880	1.72840
H	−0.29970	−4.42680	3.30540
H	4.42330	2.70560	−2.43970
H	5.39870	4.54860	−0.16000
H	4.00850	6.31600	−1.19640
H	3.43590	5.18620	−2.41950
H	3.00130	4.95010	−0.72620
H	−0.73680	1.30390	3.68860
H	7.42440	3.34230	−1.93350
H	8.05870	4.87290	−2.52830
H	7.68580	4.69220	−0.81570

**Marilzafurollene C_83**			
C	2.62140	−0.59780	−0.56050
O	2.56330	−0.60920	0.86560
C	3.04350	0.78930	−1.08040
C	1.25050	−1.08210	−1.04550
C	1.28500	−1.05860	1.30640
C	4.47690	1.15270	−0.65840
Br	1.78670	2.18210	−0.39280
C	0.75820	−1.89700	0.13170
C	1.41280	−1.73640	2.68840
C	4.95230	2.45390	−1.27550
Cl	1.49500	−3.52740	0.07740
C	0.07670	−2.01840	3.40660
C	−0.71790	−3.14760	2.77720
O	0.34620	−2.34340	4.75510
C	−1.94820	−3.00620	2.33950
C	6.00250	2.56560	−2.10570
C	6.47670	3.86950	−2.72010
C	6.41300	3.80690	−4.24820
O	7.82490	4.14340	−2.36800
C	−3.16040	−2.84180	1.86670
Br	−3.48940	−2.27130	0.00030
C	8.00860	4.51560	−1.00870
H	3.36750	−1.33720	−0.85590
H	2.97460	0.80110	−2.16930
H	0.57460	−0.24260	−1.20920
H	1.30100	−1.65170	−1.97380
H	0.65960	−0.16940	1.40530
H	4.53500	1.23610	0.42760
H	5.15960	0.35140	−0.94400
H	−0.32570	−2.01080	0.13140
H	2.01720	−2.64070	2.61960
H	1.98860	−1.05360	3.31500
H	4.38930	3.33780	−1.01000
H	−0.52400	−1.10590	3.41280
H	−0.21880	−4.10230	2.69320
H	0.96510	−3.05850	4.78610
H	6.57190	1.68690	−2.37300
H	5.83790	4.68830	−2.38270
H	7.05580	3.01780	−4.63900
H	5.39640	3.61480	−4.59250
H	6.73990	4.74920	−4.68830
H	−4.07130	−2.99420	2.42730
H	7.72120	3.71470	−0.32670
H	9.06060	4.73910	−0.83180
H	7.43190	5.40790	−0.76190

**Marilzafurollene C_84**			
C	2.85140	−0.21040	−1.12610
O	1.94040	−0.29020	−0.02920
C	2.46880	0.94900	−2.07050
C	2.85430	−1.59860	−1.77810
C	1.42280	−1.61490	0.08100
C	2.59820	2.34010	−1.41690
Br	0.56910	0.72590	−2.63960
C	2.45500	−2.49940	−0.62770
C	1.10600	−1.96990	1.54680
C	4.01230	2.80510	−1.09540
Cl	3.88330	−2.82790	0.39860
C	−0.01820	−1.10710	2.14630
C	−0.42950	−1.60290	3.51850
O	0.44800	0.22010	2.25510
C	−1.64200	−2.01950	3.80390
C	5.16270	2.25640	−1.52740
C	6.54400	2.76160	−1.15630
C	7.31870	3.19740	−2.40250
O	7.30630	1.74400	−0.52390
C	−2.85400	−2.44420	4.07170
Br	−3.38850	−4.33730	3.84880
C	6.86520	1.41080	0.78590
H	3.84060	−0.03910	−0.70100
H	3.06810	0.91040	−2.98070
H	2.10200	−1.66220	−2.56460
H	3.81660	−1.86180	−2.21820
H	0.49900	−1.64670	−0.49990
H	2.15400	3.09030	−2.07160
H	2.01450	2.35830	−0.49560
H	2.04480	−3.45570	−0.95430
H	0.80670	−3.01830	1.58790
H	2.00070	−1.88570	2.16490
H	4.06600	3.67340	−0.45370
H	−0.88050	−1.11650	1.47560
H	0.33990	−1.59790	4.27660
H	0.96890	0.39530	1.47430
H	5.15500	1.39510	−2.17760
H	6.45580	3.61990	−0.48720
H	6.79540	3.99300	−2.93360
H	8.30620	3.57230	−2.13290
H	7.45740	2.36560	−3.09350
H	−3.66060	−1.82190	4.43100
H	5.85470	1.00020	0.78200
H	7.52610	0.65530	1.21070
H	6.88610	2.28060	1.44370

**Marilzafurollene C_85**			
C	2.66030	−1.61480	−1.40230
O	2.27120	−1.73190	−0.03460
C	3.14940	−0.18940	−1.71680
C	1.45620	−2.08660	−2.22410
C	0.99940	−2.36940	0.06380
C	4.43170	0.16470	−0.94480
Br	1.72570	1.13170	−1.25370
C	0.79880	−3.07480	−1.28380
C	0.92440	−3.26570	1.31560
C	4.99080	1.51970	−1.33370
Cl	1.65660	−4.64330	−1.36840
C	0.91120	−2.47470	2.63580
C	−0.39420	−1.73030	2.84500
O	1.08650	−3.38890	3.69930
C	−0.48400	−0.42270	2.93140
C	5.19240	2.53250	−0.47450
C	5.73810	3.89380	−0.86260
C	7.05580	4.18090	−0.13840
O	4.83360	4.93160	−0.51310
C	−0.60150	0.88200	3.00050
Br	−0.94380	2.00010	1.40470
C	3.63600	4.94640	−1.27910
H	3.47790	−2.31920	−1.56410
H	3.33230	−0.10220	−2.78860
H	0.76970	−1.26250	−2.41960
H	1.73570	−2.52260	−3.18350
H	0.25170	−1.57760	0.13280
H	4.23660	0.13510	0.12830
H	5.20040	−0.58340	−1.14120
H	−0.25190	−3.25230	−1.51580
H	0.03960	−3.90100	1.26220
H	1.78180	−3.93960	1.31550
H	5.23310	1.65430	−2.37800
H	1.75280	−1.77770	2.64140
H	−1.28100	−2.34250	2.92430
H	1.34880	−2.90760	4.47130
H	4.95050	2.40290	0.57070
H	5.92250	3.92140	−1.93840
H	7.80800	3.42820	−0.37590
H	7.45440	5.15270	−0.43010
H	6.91810	4.18980	0.94310
H	−0.52940	1.46270	3.90840
H	3.84840	5.04310	−2.34450
H	3.04240	4.04510	−1.12220
H	3.02500	5.79850	−0.98190

**Marilzafurollene C_86**			
C	3.38410	−1.47810	−1.81560
O	2.40830	−1.08400	−0.84940
C	3.46050	−0.45370	−2.96680
C	2.98700	−2.89560	−2.24710
C	1.45390	−2.12380	−0.65680
C	4.19050	0.85650	−2.60840
Br	1.62260	−0.02770	−3.61910
C	2.20100	−3.39930	−1.05270
C	0.87710	−2.06670	0.76940
C	3.64500	1.54960	−1.37310
Cl	3.32610	−3.94160	0.22780
C	−0.02030	−0.83800	1.00630
C	−0.64500	−0.87200	2.39630
O	0.74630	0.33450	0.79540
C	−0.40970	−0.00880	3.36050
C	4.39710	1.92390	−0.32450
C	3.86760	2.59380	0.92880
C	4.35590	4.04180	1.01730
O	4.33950	1.92830	2.09090
C	−0.14420	0.85210	4.31390
Br	1.31300	0.56860	5.62470
C	3.67010	0.70620	2.37000
H	4.34900	−1.53180	−1.30860
H	3.98810	−0.90640	−3.80780
H	2.33450	−2.86050	−3.12050
H	3.84310	−3.52320	−2.49620
H	0.64710	−1.96860	−1.37580
H	5.25010	0.64810	−2.45520
H	4.13230	1.54880	−3.44880
H	1.53500	−4.22820	−1.29500
H	0.29360	−2.97120	0.94600
H	1.68470	−2.07780	1.50290
H	2.58050	1.73860	−1.36170
H	−0.83300	−0.83850	0.27790
H	−1.33620	−1.68400	2.56940
H	1.41670	0.11730	0.14960
H	5.46190	1.74230	−0.33810
H	2.77540	2.59620	0.91580
H	3.96990	4.52520	1.91500
H	5.44430	4.09060	1.05520
H	4.02180	4.62260	0.15730
H	−0.67910	1.77590	4.47640
H	2.62180	0.88560	2.60890
H	3.72500	0.00660	1.53480
H	4.12650	0.22870	3.23670

**Marilzafurollene C_87**			
C	3.12860	−1.50360	−0.21040
O	2.13440	−1.94420	0.71350
C	3.52640	−0.05210	0.11370
C	2.51050	−1.70510	−1.60230
C	0.95600	−2.34120	0.02320
C	4.64230	0.48760	−0.79840
Br	4.15510	0.01970	2.00610
C	1.45850	−2.76830	−1.35780
C	0.15500	−3.38610	0.82630
C	4.93940	1.95240	−0.54220
Cl	2.23120	−4.38150	−1.33300
C	−0.31090	−2.86730	2.19950
C	−1.25090	−1.68180	2.08370
O	−0.97170	−3.91740	2.87590
C	−0.91960	−0.46530	2.45270
C	4.80650	2.92880	−1.45530
C	5.10310	4.39380	−1.19450
C	3.85180	5.24920	−1.40770
O	6.10030	4.88170	−2.08030
C	−0.54760	0.75150	2.77100
Br	0.32300	1.95710	1.46430
C	7.40770	4.39330	−1.81220
H	3.99130	−2.16140	−0.09410
H	2.64700	0.59040	0.05090
H	2.02400	−0.78830	−1.93810
H	3.24300	−1.99020	−2.35810
H	0.34690	−1.44620	−0.11400
H	5.55680	−0.08780	−0.65050
H	4.36340	0.36460	−1.84530
H	0.67280	−2.77910	−2.11390
H	−0.70630	−3.71130	0.24190
H	0.76970	−4.27260	0.98340
H	5.28060	2.20060	0.45330
H	0.57050	−2.58820	2.78230
H	−2.22750	−1.88300	1.66760
H	−0.98600	−3.71770	3.80140
H	4.46870	2.68960	−2.45330
H	5.43600	4.52340	−0.16270
H	4.06140	6.29960	−1.20490
H	3.49160	5.17480	−2.43400
H	3.04460	4.93760	−0.74410
H	−0.67200	1.20510	3.74350
H	7.46720	3.31030	−1.92630
H	8.11330	4.83900	−2.51320
H	7.72850	4.65610	−0.80340

**Marilzafurollene C_88**			
C	3.10590	−1.47190	−1.01890
O	2.00830	−1.39360	−0.10870
C	3.49160	−0.06940	−1.52460
C	2.68410	−2.46120	−2.11300
C	0.95610	−2.25580	−0.53770
C	4.03200	0.82500	−0.39610
Br	1.89850	0.80990	−2.34970
C	1.64980	−3.31210	−1.40520
C	0.13830	−2.78160	0.65870
C	4.53740	2.16100	−0.90530
Cl	2.47270	−4.55950	−0.42150
C	−0.62660	−1.67380	1.40540
C	−1.59860	−2.25810	2.42530
O	0.32290	−0.81120	2.00680
C	−1.62180	−1.98010	3.71080
C	5.80570	2.58960	−0.79360
C	6.30780	3.92810	−1.30250
C	7.40760	3.73530	−2.34960
O	6.86300	4.70290	−0.24980
C	−1.62950	−1.69370	4.99120
Br	−2.72120	−0.22590	5.75070
C	5.90420	5.21450	0.66610
H	3.94590	−1.90510	−0.47340
H	4.24970	−0.16310	−2.30390
H	2.21310	−1.94220	−2.94810
H	3.51830	−3.03910	−2.51180
H	0.30180	−1.66770	−1.18410
H	3.25150	1.00400	0.34450
H	4.84150	0.31220	0.12510
H	0.96800	−3.81630	−2.09100
H	−0.57510	−3.51750	0.28530
H	0.78230	−3.31380	1.36000
H	3.80410	2.79350	−1.38600
H	−1.21120	−1.09290	0.69000
H	−2.31970	−2.95530	2.02410
H	1.05480	−0.73480	1.39870
H	6.54370	1.96460	−0.31160
H	5.48520	4.47730	−1.76520
H	7.75750	4.69700	−2.72520
H	8.26630	3.21120	−1.92950
H	7.04440	3.15880	−3.20080
H	−1.05790	−2.21880	5.74200
H	6.40770	5.82110	1.41870
H	5.17390	5.84790	0.16100
H	5.37330	4.41600	1.18550

**Marilzafurollene C_89**			
C	2.79070	−1.85290	−1.40060
O	2.25380	−1.97440	−0.08340
C	3.25790	−0.40860	−1.66910
C	1.70800	−2.37210	−2.35930
C	0.89560	−2.38970	−0.15080
C	4.35840	0.03360	−0.68910
Br	1.71390	0.85060	−1.52340
C	0.82140	−3.19980	−1.45090
C	0.46390	−3.12570	1.13260
C	4.88630	1.42230	−0.99230
Cl	1.52410	−4.83600	−1.27380
C	0.56460	−2.27300	2.41110
C	−0.32580	−1.04450	2.38440
O	0.19400	−3.08070	3.51040
C	0.10810	0.16540	2.65760
C	4.87580	2.44340	−0.11930
C	5.38410	3.84010	−0.42310
C	6.58040	4.19130	0.46480
O	4.38410	4.81980	−0.18300
C	0.54010	1.35980	2.98750
Br	1.28720	2.63920	1.67650
C	3.27300	4.75430	−1.06770
H	3.64950	−2.52400	−1.45240
H	3.62960	−0.33810	−2.69210
H	1.12460	−1.55250	−2.77780
H	2.11640	−2.94080	−3.19500
H	0.28440	−1.49440	−0.27630
H	3.97710	0.00010	0.33280
H	5.19520	−0.66400	−0.73200
H	−0.19620	−3.30690	−1.82800
H	−0.55930	−3.48350	1.01080
H	1.08250	−4.01340	1.26320
H	5.28930	1.57530	−1.98290
H	1.60280	−1.96370	2.55430
H	−1.36160	−1.20950	2.12740
H	0.90360	−3.67890	3.69810
H	4.47270	2.29530	0.87270
H	5.69940	3.89510	−1.46690
H	6.95400	5.18910	0.23450
H	6.30610	4.17490	1.51990
H	7.39970	3.48710	0.31880
H	0.53150	1.75770	3.99160
H	2.58480	5.57000	−0.84700
H	3.58790	4.85340	−2.10710
H	2.72030	3.82060	−0.95810

**Marilzafurollene C_90**			
C	2.97090	−0.62510	−1.39170
O	2.10580	−0.63980	−0.25540
C	2.56350	0.49460	−2.37140
C	2.92770	−2.04210	−1.97890
C	1.54460	−1.93980	−0.08490
C	2.72340	1.90730	−1.78420
Br	0.65080	0.26370	−2.89110
C	2.53020	−2.88330	−0.78340
C	1.25090	−2.23130	1.39980
C	4.16830	2.27110	−1.50240
Cl	3.97310	−3.21850	0.21960
C	0.17300	−1.30750	1.99390
C	−0.21950	−1.73750	3.39380
O	0.69030	0.00430	2.04040
C	−1.42670	−2.14250	3.71750
C	4.65160	2.57760	−0.28600
C	6.09500	2.93920	0.02240
C	6.22730	4.43320	0.32750
O	6.96930	2.64670	−1.06120
C	−2.62680	−2.56250	4.04180
Br	−4.05240	−1.33940	4.66730
C	7.36120	1.28200	−1.13420
H	3.98160	−0.44490	−1.02240
H	3.15120	0.41480	−3.28700
H	2.15870	−2.12310	−2.74730
H	3.87390	−2.34820	−2.42590
H	0.60680	−1.96050	−0.64340
H	2.32820	2.64130	−2.48700
H	2.12900	2.00020	−0.87410
H	2.08210	−3.83780	−1.06180
H	0.91660	−3.26610	1.48570
H	2.16280	−2.15720	1.99350
H	4.83370	2.28040	−2.35410
H	−0.70650	−1.30980	1.34580
H	0.55990	−1.69190	4.14040
H	1.19170	0.13300	1.23820
H	3.98230	2.57480	0.56300
H	6.40570	2.38070	0.90750
H	7.25770	4.68870	0.57490
H	5.93230	5.03700	−0.53110
H	5.60150	4.72170	1.17230
H	−2.95160	−3.59190	4.00170
H	6.50300	0.61650	−1.23310
H	8.00190	1.13240	−2.00320
H	7.92670	0.98850	−0.24900

**Marilzafurollene C_91**			
C	2.48610	−1.20020	−2.19820
O	2.54530	−1.13960	−0.77500
C	1.78920	0.05350	−2.77430
C	1.79930	−2.53530	−2.52440
C	1.85750	−2.24000	−0.20180
C	2.60130	1.35770	−2.62320
Br	−0.00490	0.30020	−1.93180
C	2.00120	−3.34190	−1.25380
C	2.38760	−2.53300	1.21450
C	2.97100	1.67850	−1.18640
Cl	3.63660	−4.06660	−1.25530
C	2.16510	−1.35920	2.18820
C	0.69350	−1.07100	2.41790
O	2.77120	−1.67890	3.42360
C	0.12100	0.06650	2.09540
C	4.22570	1.88340	−0.74910
C	4.63080	2.15430	0.69080
C	5.47570	0.99990	1.23560
O	3.51270	2.30210	1.55830
C	−0.46020	1.19040	1.74900
Br	−0.63970	2.72380	2.98790
C	2.95280	3.60830	1.55770
H	3.51400	−1.24190	−2.56180
H	1.60840	−0.10690	−3.83810
H	0.73190	−2.38770	−2.69120
H	2.20800	−3.02040	−3.41110
H	0.80040	−1.97690	−0.14430
H	3.51230	1.28970	−3.21890
H	2.02940	2.19350	−3.02690
H	1.27280	−4.14430	−1.13340
H	1.91350	−3.43410	1.60430
H	3.45570	−2.74550	1.16380
H	2.15050	1.71700	−0.48430
H	2.65710	−0.46900	1.78830
H	0.11600	−1.86720	2.86460
H	2.92430	−0.86960	3.89160
H	5.04130	1.84100	−1.45610
H	5.24070	3.05940	0.71120
H	5.77030	1.18630	2.26830
H	4.92220	0.06080	1.21050
H	6.38480	0.86480	0.64960
H	−0.89180	1.38560	0.77810
H	2.60880	3.90430	0.56640
H	2.09750	3.63850	2.23150
H	3.67600	4.34550	1.90830

**Marilzafurollene C_92**			
C	2.67100	−1.13800	−1.31390
O	2.63740	−1.08680	0.11140
C	3.86040	−0.32110	−1.85350
C	1.27600	−0.70810	−1.78600
C	1.32220	−0.76310	0.55990
C	3.86360	1.16360	−1.44260
Br	3.89560	−0.46670	−3.84220
C	0.40370	−1.11500	−0.61710
C	1.02130	−1.43520	1.91330
C	5.15090	1.86410	−1.83190
Cl	0.05460	−2.86830	−0.70610
C	−0.40220	−1.18870	2.45080
C	−0.61100	−1.88160	3.78350
O	−0.60930	0.19940	2.61480
C	−1.53930	−2.78680	3.99560
C	6.00450	2.42840	−0.96150
C	7.29210	3.12940	−1.35270
C	8.50240	2.44030	−0.71750
O	7.29820	4.47880	−0.91000
C	−2.45340	−3.70230	4.21510
Br	−2.11490	−5.63120	3.92840
C	6.41290	5.33100	−1.62400
H	2.81610	−2.18510	−1.58460
H	4.77690	−0.78810	−1.48920
H	1.21720	0.37120	−1.91900
H	0.98320	−1.17410	−2.72730
H	1.29980	0.32010	0.69280
H	3.03360	1.69650	−1.90480
H	3.72690	1.24610	−0.36340
H	−0.54600	−0.57980	−0.58660
H	1.19140	−2.50890	1.82630
H	1.75120	−1.08180	2.64240
H	5.37140	1.89770	−2.88970
H	−1.13430	−1.56200	1.73150
H	0.06140	−1.59440	4.57950
H	−1.49360	0.34120	2.92340
H	5.78830	2.39980	0.09690
H	7.41070	3.10090	−2.43780
H	9.42820	2.93530	−1.01100
H	8.44450	2.46340	0.37090
H	8.57010	1.39760	−1.02900
H	−3.45610	−3.50340	4.56440
H	5.37280	5.02550	−1.50570
H	6.50380	6.34900	−1.24530
H	6.65290	5.34800	−2.68780

**Marilzafurollene C_93**			
C	2.59930	−0.55670	−1.24580
O	2.18530	−0.87120	0.08380
C	1.82010	0.66140	−1.78470
C	2.42890	−1.84530	−2.06250
C	1.72260	−2.21600	0.14140
C	2.10880	1.95780	−1.00930
Br	−0.14150	0.30870	−1.67340
C	2.46720	−2.92150	−0.99620
C	1.89620	−2.81120	1.55240
C	3.54200	2.42970	−1.15920
Cl	4.16690	−3.28470	−0.57150
C	1.07240	−2.07930	2.62830
C	−0.42280	−2.17060	2.38530
O	1.36960	−2.64690	3.88800
C	−1.17060	−1.12980	2.09590
C	4.41190	2.55090	−0.14160
C	5.85400	3.01380	−0.26630
C	6.02270	4.42430	0.30370
O	6.29750	3.04510	−1.61780
C	−1.89580	−0.08210	1.78300
Br	−2.79190	1.03960	3.14650
C	6.69590	1.77770	−2.12400
H	3.66450	−0.32530	−1.20200
H	2.05480	0.81100	−2.83950
H	1.45910	−1.86550	−2.56000
H	3.19490	−1.97020	−2.82830
H	0.66320	−2.20710	−0.11920
H	1.45780	2.75370	−1.37180
H	1.86850	1.81820	0.04580
H	1.98850	−3.85070	−1.30720
H	1.62080	−3.86640	1.53480
H	2.94950	−2.77470	1.83030
H	3.86030	2.68080	−2.16110
H	1.37110	−1.02840	2.65250
H	−0.85960	−3.15550	2.46160
H	2.20450	−2.31010	4.18130
H	4.08790	2.30510	0.85990
H	6.48260	2.33390	0.31210
H	7.06200	4.74690	0.23900
H	5.41640	5.14500	−0.24570
H	5.72680	4.46580	1.35220
H	−2.07230	0.26490	0.77510
H	5.88810	1.04680	−2.07380
H	6.99110	1.87830	−3.16830
H	7.55150	1.38640	−1.57220

**Marilzafurollene C_94**			
C	2.98370	−1.87440	−1.38670
O	2.31620	−1.88500	−0.12610
C	3.93440	−0.66700	−1.49260
C	1.87640	−1.96660	−2.44300
C	0.93510	−2.20270	−0.30200
C	3.25830	0.70530	−1.31280
Br	4.86110	−0.73140	−3.25750
C	0.82200	−2.77890	−1.72120
C	0.40670	−3.09860	0.83690
C	4.26400	1.83930	−1.26090
Cl	1.24760	−4.51610	−1.79350
C	0.40350	−2.41850	2.21850
C	−0.54860	−1.23730	2.28500
O	0.00650	−3.37870	3.17670
C	−0.17030	−0.01710	2.59700
C	4.45110	2.64670	−0.20350
C	5.45640	3.78290	−0.15780
C	6.48670	3.55650	0.95240
O	4.81680	5.02230	0.10960
C	0.19920	1.19700	2.93570
Br	0.19070	1.82060	4.81610
C	4.04510	5.52600	−0.97290
H	3.57810	−2.78860	−1.43430
H	4.70660	−0.78160	−0.73000
H	1.47340	−0.98370	−2.68220
H	2.21280	−2.42440	−3.37390
H	0.39510	−1.25450	−0.29690
H	2.56470	0.90600	−2.12870
H	2.67080	0.70660	−0.39350
H	−0.17460	−2.66110	−2.14780
H	−0.60300	−3.43420	0.59490
H	1.01910	−3.99950	0.89520
H	4.85860	1.98680	−2.15200
H	1.42100	−2.09730	2.45830
H	−1.58510	−1.44690	2.05720
H	0.20070	−3.04080	4.04020
H	3.86100	2.50620	0.69100
H	5.98680	3.84360	−1.11080
H	7.21540	4.36710	0.97640
H	6.00920	3.51070	1.93190
H	7.03190	2.62480	0.79980
H	0.53520	1.95800	2.24500
H	3.22380	4.85670	−1.23200
H	3.61340	6.48740	−0.69520
H	4.66230	5.68090	−1.85910

**Marilzafurollene C_95**			
C	3.24850	−1.63400	−0.10120
O	2.02810	−1.05880	0.36890
C	4.31840	−0.53130	−0.20720
C	2.89910	−2.35600	−1.41300
C	0.91430	−1.68570	−0.26020
C	3.96650	0.60500	−1.18550
Br	6.04890	−1.36620	−0.74220
C	1.46550	−1.93440	−1.66600
C	0.50590	−2.95030	0.53380
C	4.96900	1.74130	−1.13390
Cl	0.54790	−3.13720	−2.61380
C	0.09380	−2.66240	1.98890
C	−1.14390	−1.78970	2.08020
O	−0.16390	−3.89670	2.62580
C	−1.15640	−0.61400	2.66610
C	4.67210	3.00390	−0.78400
C	5.67630	4.14030	−0.73330
C	5.77350	4.71890	0.68050
O	5.29430	5.20240	−1.59520
C	−1.15130	0.55490	3.26200
Br	−1.55180	0.76530	5.18890
C	5.43860	4.91150	−2.97880
H	3.57450	−2.37370	0.63170
H	4.47060	−0.11000	0.78800
H	3.55960	−2.11330	−2.24520
H	2.95610	−3.43420	−1.25940
H	0.07400	−0.99220	−0.30360
H	3.92150	0.23200	−2.20840
H	2.97550	0.99640	−0.95150
H	1.45370	−0.99890	−2.22670
H	−0.31980	−3.44850	0.02470
H	1.32750	−3.66690	0.53570
H	5.98550	1.49040	−1.40320
H	0.92830	−2.18360	2.50700
H	−2.04160	−2.18560	1.62730
H	−0.25050	−3.74350	3.55650
H	3.65770	3.26240	−0.51570
H	6.66280	3.77220	−1.02250
H	6.50580	5.52550	0.71940
H	4.81610	5.12420	1.00890
H	6.08050	3.95710	1.39760
H	−0.93450	1.49500	2.77600
H	6.47220	4.66460	−3.22490
H	4.79910	4.08410	−3.28780
H	5.15580	5.78550	−3.56550

**Marilzafurollene C_96**			
C	2.88170	−0.90920	−0.57080
O	1.92170	−0.93440	0.48540
C	3.37560	0.53030	−0.79890
C	2.16860	−1.54540	−1.76870
C	0.79400	−1.73180	0.12450
C	4.44970	0.63340	−1.89720
Br	4.14650	1.20270	0.91360
C	1.23910	−2.53800	−1.10220
C	0.27510	−2.54230	1.32840
C	4.88120	2.06410	−2.15440
Cl	2.15370	−4.01080	−0.65920
C	−0.27240	−1.65840	2.46280
C	−0.96260	−2.48730	3.52800
O	0.80290	−0.95820	3.04980
C	−2.23050	−2.35090	3.84300
C	6.14470	2.50740	−2.04540
C	6.57690	3.94140	−2.28780
C	7.58490	4.02130	−3.43700
O	7.20660	4.49110	−1.13960
C	−3.50100	−2.21200	4.13900
Br	−4.92300	−3.23680	3.21870
C	6.33040	4.71440	−0.04240
H	3.71660	−1.54330	−0.26790
H	2.52830	1.17450	−1.03650
H	1.58210	−0.79740	−2.30380
H	2.85360	−2.00900	−2.47940
H	0.01400	−1.04050	−0.19940
H	5.31570	0.02690	−1.62790
H	4.06740	0.23210	−2.83600
H	0.40560	−2.83910	−1.73800
H	−0.52150	−3.20050	0.97850
H	1.05720	−3.19440	1.71930
H	4.10060	2.75430	−2.44080
H	−0.97450	−0.93040	2.05010
H	−0.34850	−3.21740	4.03480
H	1.39920	−0.70710	2.34760
H	6.92980	1.82260	−1.75840
H	5.70710	4.54730	−2.54920
H	7.88310	5.05380	−3.61950
H	8.48630	3.45070	−3.21220
H	7.16040	3.62970	−4.36170
H	−3.88930	−1.54340	4.89350
H	5.90540	3.78330	0.33400
H	6.88230	5.17840	0.77480
H	5.51470	5.38420	−0.31710

**Marilzafurollene C_97**			
C	3.03190	−1.83240	−1.06190
O	2.43480	−1.90520	0.23190
C	3.77460	−0.49420	−1.23470
C	1.90720	−2.12440	−2.06390
C	1.06900	−2.29820	0.11960
C	2.89880	0.75750	−1.04050
Br	4.62070	−0.45160	−3.03990
C	0.96520	−2.98470	−1.24750
C	0.63000	−3.13380	1.33780
C	3.71630	2.03360	−1.01990
Cl	1.56590	−4.67000	−1.20830
C	0.59890	−2.32930	2.65010
C	−0.49390	−1.27660	2.65800
O	0.37620	−3.22170	3.72250
C	−0.26020	0.01440	2.72490
C	3.80120	2.86710	0.03140
C	4.61180	4.15190	0.07670
C	3.68880	5.37280	0.07290
O	5.49120	4.28140	−1.03400
C	−0.03610	1.30620	2.76740
Br	0.18320	2.39700	1.13040
C	6.70000	3.54380	−0.90620
H	3.75860	−2.64460	−1.11700
H	4.58820	−0.46780	−0.50820
H	1.39450	−1.21060	−2.36120
H	2.26300	−2.61350	−2.97130
H	0.47590	−1.38350	0.07020
H	2.15550	0.84230	−1.83220
H	2.34860	0.67520	−0.10260
H	−0.05180	−2.99800	−1.64090
H	−0.35470	−3.56380	1.15000
H	1.31320	−3.97600	1.45070
H	4.25420	2.26760	−1.92800
H	1.57020	−1.85300	2.80410
H	−1.50800	−1.64550	2.60790
H	1.16770	−3.72030	3.87050
H	3.25460	2.63350	0.93400
H	5.18700	4.16100	1.00450
H	4.26540	6.29580	0.13490
H	3.09500	5.41210	−0.84070
H	3.00120	5.35200	0.91880
H	0.04720	1.88920	3.67300
H	6.51580	2.47660	−0.77870
H	7.30210	3.67280	−1.80550
H	7.28740	3.89780	−0.05810

**Marilzafurollene C_98**			
C	2.94610	−1.34240	−0.56370
O	1.65810	−1.09960	0.00710
C	3.43530	−0.08370	−1.30530
C	2.79330	−2.58680	−1.45080
C	0.82900	−2.25010	−0.12680
C	3.62920	1.11250	−0.35830
Br	2.12840	0.41150	−2.73220
C	1.29450	−2.81140	−1.47240
C	1.03450	−3.17700	1.09540
C	4.24240	2.31150	−1.05550
Cl	0.85850	−4.52090	−1.74810
C	0.63640	−2.52920	2.43480
C	−0.84800	−2.22490	2.51760
O	0.98090	−3.41420	3.48140
C	−1.33340	−1.01060	2.64390
C	5.42470	2.86060	−0.73080
C	6.03470	4.06160	−1.42950
C	7.38320	3.70000	−2.05730
O	6.26930	5.12320	−0.51590
C	−1.80620	0.20810	2.75700
Br	−2.13230	1.06640	4.51080
C	5.08970	5.77130	−0.05920
H	3.64060	−1.57670	0.24460
H	4.38220	−0.30450	−1.80070
H	3.20740	−2.46580	−2.45220
H	3.29680	−3.43470	−0.98530
H	−0.21710	−1.94890	−0.18910
H	2.67080	1.40780	0.07050
H	4.26590	0.82090	0.47790
H	0.84830	−2.22630	−2.27790
H	0.45310	−4.09000	0.96240
H	2.07660	−3.49240	1.14310
H	3.66540	2.73490	−1.86580
H	1.20490	−1.60660	2.57410
H	−1.51080	−3.07650	2.46860
H	1.89500	−3.29260	3.69510
H	6.00580	2.44500	0.07990
H	5.36680	4.40350	−2.22280
H	7.81370	4.55950	−2.57140
H	8.09730	3.37430	−1.30060
H	7.27530	2.89690	−2.78680
H	−2.05890	0.85040	1.92600
H	4.44240	5.09120	0.49560
H	5.35960	6.58950	0.60850
H	4.52290	6.19220	−0.89060

**Marilzafurollene C_99**			
C	2.84940	−1.83550	−1.79820
O	2.53540	−1.63670	−0.41990
C	2.76960	−0.52010	−2.60120
C	1.89990	−2.93580	−2.29210
C	1.46250	−2.48930	−0.03620
C	3.89930	0.48460	−2.29510
Br	0.99140	0.35500	−2.34540
C	1.54200	−3.66540	−1.01400
C	1.52840	−2.83760	1.46380
C	3.77030	1.17460	−0.94910
Cl	2.84950	−4.81200	−0.59290
C	1.39930	−1.60910	2.38280
C	0.07260	−0.89260	2.21600
O	1.52900	−2.03310	3.72450
C	−0.02990	0.33660	1.76420
C	3.86330	2.50260	−0.76890
C	3.71860	3.20290	0.56840
C	2.68110	4.32430	0.48070
O	4.94220	3.80020	0.97220
C	−0.11300	1.56140	1.30260
Br	−0.09720	3.14270	2.49310
C	5.93630	2.87070	1.38070
H	3.86840	−2.22320	−1.84130
H	2.83720	−0.77200	−3.66070
H	0.99720	−2.50200	−2.72340
H	2.34920	−3.58240	−3.04630
H	0.53460	−1.95430	−0.24470
H	4.86480	−0.02040	−2.33670
H	3.91910	1.24700	−3.07480
H	0.60860	−4.22480	−1.08570
H	0.74250	−3.55600	1.69990
H	2.47330	−3.33730	1.67700
H	3.59660	0.53720	−0.09450
H	2.21510	−0.91470	2.16730
H	−0.81000	−1.45210	2.48870
H	2.44310	−2.20500	3.90150
H	4.05090	3.14520	−1.61690
H	3.39110	2.48650	1.32480
H	2.54190	4.80050	1.45140
H	2.98930	5.09460	−0.22640
H	1.71350	3.94070	0.15700
H	−0.19820	1.82210	0.25730
H	6.23210	2.20940	0.56560
H	6.82510	3.40980	1.70830
H	5.58850	2.26160	2.21590

**Marilzafurollene C_100**			
C	2.52010	−1.08050	−1.71520
O	1.46660	−0.89910	−0.77310
C	2.35750	−0.12390	−2.91200
C	2.51810	−2.58280	−2.03500
C	1.35740	−2.07370	0.02110
C	2.31970	1.36120	−2.50000
Br	0.66760	−0.53890	−3.88650
C	1.48530	−3.14390	−1.06720
C	2.46140	−2.08090	1.10900
C	3.48700	1.76290	−1.61530
Cl	1.92190	−4.76140	−0.44950
C	2.41580	−0.83730	2.01520
C	1.28620	−0.90280	3.02440
O	3.64500	−0.71910	2.70000
C	0.29190	−0.04560	3.05970
C	3.34730	2.23220	−0.36130
C	4.46260	2.60020	0.60960
C	5.86660	2.22150	0.11840
O	4.27990	1.93620	1.85590
C	−0.71860	0.79050	3.09140
Br	−2.38800	0.47340	2.07700
C	3.35440	2.58140	2.72370
H	3.46130	−0.85360	−1.21310
H	3.17550	−0.28610	−3.61510
H	2.25480	−2.80770	−3.06860
H	3.50650	−3.00310	−1.84700
H	0.37490	−2.11440	0.49290
H	2.31160	1.99430	−3.38750
H	1.38680	1.55990	−1.97020
H	0.52770	−3.23660	−1.58220
H	2.37670	−2.98000	1.72030
H	3.44290	−2.14760	0.64070
H	4.46810	1.63500	−2.04780
H	2.30720	0.05580	1.39670
H	1.33560	−1.71240	3.73780
H	4.03770	0.11390	2.44680
H	2.34490	2.35580	0.02160
H	4.43950	3.68160	0.75710
H	6.61810	2.49110	0.86070
H	5.95020	1.14870	−0.05810
H	6.12230	2.74290	−0.80420
H	−0.74280	1.71000	3.65750
H	3.27900	2.02330	3.65720
H	3.68090	3.59280	2.96890
H	2.35570	2.63250	2.28970

**Marilzafurollene C_101**			
C	3.25790	−1.12180	−1.26600
O	1.84910	−0.93280	−1.11540
C	3.74510	−0.41020	−2.54430
C	3.48570	−2.64090	−1.24480
C	1.25900	−2.09820	−0.54720
C	3.49660	1.10830	−2.52770
Br	2.80350	−1.17170	−4.12930
C	2.07650	−3.20250	−1.22080
C	1.35860	−2.03310	0.99650
C	4.23200	1.81380	−1.40500
Cl	1.97960	−4.78640	−0.40210
C	0.60890	−0.83560	1.60830
C	0.55570	−0.92430	3.12100
O	1.28030	0.34890	1.23790
C	−0.56190	−1.03730	3.80220
C	3.62840	2.44680	−0.38400
C	4.33700	3.14850	0.76200
C	4.20190	4.66780	0.63500
O	5.72690	2.84680	0.80400
C	−1.67750	−1.16590	4.48110
Br	−2.71470	0.38960	5.13230
C	6.02800	1.60840	1.43350
H	3.75380	−0.69280	−0.39400
H	4.80960	−0.60150	−2.68830
H	4.06010	−3.01040	−2.09470
H	4.02310	−2.91910	−0.33760
H	0.21050	−2.16380	−0.84040
H	3.82100	1.54560	−3.47230
H	2.42650	1.30530	−2.44900
H	1.72120	−3.33790	−2.24370
H	0.92770	−2.94770	1.40570
H	2.39940	−2.02580	1.32190
H	5.31170	1.79010	−1.45030
H	−0.40580	−0.79890	1.20510
H	1.50580	−0.88590	3.63320
H	1.48660	0.26540	0.31020
H	2.54850	2.47490	−0.34230
H	3.86270	2.83960	1.69570
H	4.68790	5.17130	1.47070
H	4.66400	5.02790	−0.28450
H	3.15500	4.97200	0.62720
H	−2.13220	−2.10880	4.74730
H	5.53490	0.77060	0.93950
H	7.10300	1.43240	1.39790
H	5.72610	1.61810	2.48160

**Marilzafurollene C_102**			
C	2.95250	−0.99320	−0.21440
O	1.62130	−0.50670	−0.02270
C	3.78710	0.05680	−0.97120
C	2.81430	−2.34160	−0.93690
C	0.68040	−1.56610	−0.18420
C	3.94940	1.36080	−0.17170
Br	2.92630	0.46150	−2.72720
C	1.34030	−2.39630	−1.28740
C	0.47710	−2.28800	1.16970
C	4.88470	2.34320	−0.84890
Cl	0.72520	−4.06760	−1.41670
C	−0.13210	−1.38660	2.25850
C	−0.49980	−2.17950	3.49710
O	0.81260	−0.39860	2.60780
C	−1.72460	−2.26680	3.96340
C	6.04800	2.77400	−0.33040
C	6.99970	3.75580	−0.99550
C	6.98840	5.10090	−0.26520
O	6.66010	4.00930	−2.35350
C	−2.95430	−2.35440	4.41200
Br	−4.20020	−3.77560	3.82330
C	7.10700	3.00990	−3.26060
H	3.39820	−1.17040	0.76590
H	4.77450	−0.35530	−1.18590
H	3.45120	−2.43560	−1.81690
H	3.07220	−3.15220	−0.25440
H	−0.27430	−1.16590	−0.52760
H	2.97890	1.83920	−0.03430
H	4.32840	1.13620	0.82610
H	1.17460	−1.91050	−2.25030
H	−0.20060	−3.12830	1.01290
H	1.41170	−2.72330	1.52490
H	4.56690	2.71010	−1.81510
H	−1.02050	−0.89000	1.86140
H	0.31300	−2.68500	3.99770
H	1.17970	−0.06800	1.79210
H	6.36020	2.41330	0.63920
H	8.00960	3.34520	−0.93940
H	7.27150	4.98640	0.78130
H	7.68970	5.79750	−0.72480
H	5.99840	5.55670	−0.29790
H	−3.39940	−1.67910	5.12810
H	6.70180	2.02720	−3.01760
H	6.78340	3.26340	−4.27010
H	8.19570	2.94420	−3.26630

**Marilzafurollene C_103**			
C	2.44210	−0.88050	−1.14950
O	2.75080	−1.08590	0.22870
C	3.06640	0.43120	−1.66180
C	0.91510	−0.95720	−1.25900
C	1.55650	−1.28380	0.98040
C	4.60370	0.38830	−1.63410
Br	2.43870	1.96360	−0.54410
C	0.54440	−1.79750	−0.05530
C	1.84470	−2.16160	2.21830
C	5.23210	1.62690	−2.24320
Cl	0.78450	−3.52830	−0.44190
C	0.71900	−2.20120	3.27250
C	−0.48560	−3.00300	2.81950
O	1.24060	−2.79250	4.44440
C	−1.70080	−2.50930	2.75080
C	6.06530	2.45410	−1.59030
C	6.68520	3.70140	−2.19180
C	8.21270	3.60230	−2.20090
O	6.34520	4.85940	−1.44270
C	−2.90300	−1.99660	2.63950
Br	−3.51790	−1.11750	0.97660
C	4.97640	5.23290	−1.53250
H	2.86600	−1.72180	−1.70020
H	2.72490	0.61560	−2.68120
H	0.46560	0.03090	−1.15960
H	0.57400	−1.38210	−2.20330
H	1.23590	−0.29610	1.31660
H	4.95100	0.25990	−0.60770
H	4.96040	−0.47770	−2.19260
H	−0.49340	−1.65500	0.24450
H	2.13160	−3.17070	1.92290
H	2.73170	−1.74410	2.69680
H	4.97450	1.83670	−3.27130
H	0.42230	−1.17900	3.51870
H	−0.29540	−4.03020	2.54070
H	0.62690	−2.66180	5.15350
H	6.32460	2.24880	−0.56150
H	6.34120	3.82180	−3.22090
H	8.65600	4.49250	−2.64720
H	8.60830	3.50620	−1.18950
H	8.54810	2.73950	−2.77690
H	−3.65430	−1.99940	3.41590
H	4.68320	5.41670	−2.56680
H	4.31910	4.47000	−1.11390
H	4.81310	6.15240	−0.97080

**Marilzafurollene C_104**			
C	2.82110	−0.60130	−0.93780
O	1.72670	−0.46250	−0.03080
C	2.83550	0.55770	−1.95570
C	2.69080	−2.00130	−1.55240
C	0.94790	−1.65770	−0.02030
C	3.06140	1.94160	−1.32110
Br	1.09010	0.61470	−2.92150
C	1.91190	−2.75210	−0.49220
C	0.27570	−1.88020	1.34870
C	4.43040	2.09620	−0.68560
Cl	3.02540	−3.27780	0.80580
C	−0.76840	−0.79330	1.69450
C	−1.59510	−1.05660	2.94600
O	−0.09200	0.43510	1.84840
C	−1.49200	−2.10650	3.73090
C	5.38520	2.93000	−1.13030
C	6.75380	3.08900	−0.49440
C	6.97170	4.52880	−0.02280
O	7.78800	2.79140	−1.42100
C	−1.39430	−3.16520	4.49960
Br	−2.45340	−4.80710	4.18180
C	7.91410	1.41080	−1.73240
H	3.73470	−0.58560	−0.34280
H	3.60630	0.37870	−2.70700
H	2.10940	−1.97290	−2.47400
H	3.65360	−2.45770	−1.78390
H	0.17310	−1.54290	−0.78080
H	2.93910	2.70920	−2.08620
H	2.29890	2.13750	−0.56610
H	1.40050	−3.63420	−0.87920
H	−0.21330	−2.85520	1.32370
H	1.02640	−1.93290	2.13820
H	4.62470	1.48690	0.18570
H	−1.46640	−0.69410	0.86140
H	−2.31960	−0.28980	3.17870
H	0.60870	0.44980	1.19990
H	5.19890	3.54370	−2.00020
H	6.83660	2.42780	0.37050
H	7.94930	4.63890	0.44680
H	6.92350	5.22990	−0.85640
H	6.21760	4.82240	0.70780
H	−0.74420	−3.24660	5.35840
H	7.01520	1.01960	−2.21010
H	8.74510	1.26820	−2.42320
H	8.11880	0.82120	−0.83790

**Marilzafurollene C_105**			
C	3.57210	−1.94140	−1.43270
O	2.95960	−1.50650	−0.22100
C	5.00430	−1.38710	−1.58290
C	2.57640	−1.59750	−2.54490
C	1.58420	−1.19360	−0.45180
C	5.15560	0.13730	−1.76370
Br	5.85210	−2.27480	−3.15130
C	1.24910	−1.76930	−1.83550
C	0.70570	−1.66820	0.72250
C	4.62800	0.95250	−0.59850
Cl	0.81880	−3.50500	−1.76710
C	0.98570	−0.91710	2.03630
C	0.02120	−1.33260	3.12930
O	2.30340	−1.20670	2.44860
C	−0.80750	−0.50230	3.72030
C	3.85670	2.04550	−0.72440
C	3.33600	2.87060	0.43820
C	1.81440	3.02200	0.36900
O	3.87710	4.18330	0.41720
C	−1.64840	0.32120	4.30000
Br	−3.47460	0.63680	3.60400
C	5.25230	4.25460	0.76830
H	3.63880	−3.02830	−1.36000
H	5.58350	−1.70010	−0.71290
H	2.68230	−0.56090	−2.86210
H	2.68320	−2.23180	−3.42540
H	1.52090	−0.10760	−0.52760
H	6.21620	0.36990	−1.86440
H	4.69000	0.46650	−2.69250
H	0.43120	−1.24140	−2.32750
H	−0.34100	−1.52200	0.45130
H	0.83060	−2.74040	0.88040
H	4.91350	0.61570	0.38820
H	0.90770	0.15820	1.86420
H	0.04500	−2.37450	3.41370
H	2.82100	−1.33840	1.65760
H	3.57600	2.39140	−1.70880
H	3.59310	2.38250	1.38050
H	1.44670	3.60960	1.21040
H	1.50860	3.52860	−0.54670
H	1.31650	2.05440	0.40070
H	−1.43570	0.89510	5.19010
H	5.42570	3.85750	1.76930
H	5.87950	3.70900	0.06260
H	5.57560	5.29550	0.76190

**Marilzafurollene C_106**			
C	2.70670	−0.71180	−1.52590
O	1.79060	−0.45860	−0.45980
C	2.62280	0.40090	−2.59090
C	2.38430	−2.12150	−2.03900
C	0.94580	−1.59050	−0.26240
C	3.06060	1.77940	−2.06690
Br	0.74250	0.56420	−3.23860
C	1.73980	−2.77150	−0.83200
C	0.50030	−1.70810	1.20840
C	4.53190	1.83510	−1.70300
Cl	3.01130	−3.33800	0.29180
C	−0.38850	−0.52910	1.66750
C	−1.00790	−0.67820	3.05070
O	0.39120	0.64640	1.64460
C	−0.85000	−1.70290	3.85930
C	4.99730	2.07620	−0.46610
C	6.46690	2.13110	−0.09340
C	6.80240	1.06660	0.95410
O	6.81060	3.39090	0.46490
C	−0.70020	−2.73670	4.65310
Br	−1.91440	−4.29920	4.59930
C	6.82400	4.46100	−0.47010
H	3.70680	−0.73350	−1.09050
H	3.23150	0.13070	−3.45500
H	1.66330	−2.08450	−2.85580
H	3.26340	−2.65670	−2.39880
H	0.06380	−1.44600	−0.88930
H	2.87890	2.53550	−2.83130
H	2.45110	2.05890	−1.20620
H	1.11040	−3.62420	−1.08930
H	−0.05460	−2.64150	1.31380
H	1.36810	−1.79040	1.86410
H	5.23060	1.66570	−2.50990
H	−1.20850	−0.40580	0.95790
H	−1.62450	0.15260	3.36160
H	0.97510	0.57760	0.89170
H	4.30090	2.24620	0.34310
H	7.07760	1.94650	−0.97940
H	7.86150	1.09450	1.21030
H	6.23470	1.22280	1.87180
H	6.57530	0.06590	0.58550
H	0.07890	−2.83780	5.39460
H	5.83800	4.63320	−0.90300
H	7.12950	5.37880	0.03220
H	7.53180	4.27110	−1.27790

**Marilzafurollene C_107**			
C	3.35790	−1.53850	−0.60290
O	2.37130	−1.47890	0.42680
C	4.28710	−0.31150	−0.53440
C	2.58560	−1.74120	−1.91250
C	1.08440	−1.79610	−0.10720
C	3.57230	1.04870	−0.65020
Br	5.66450	−0.46480	−1.96790
C	1.35900	−2.49630	−1.44490
C	0.23090	−2.57790	0.91130
C	4.50800	2.21340	−0.39160
Cl	1.75930	−4.22780	−1.23610
C	−0.12470	−1.75690	2.16340
C	−1.10930	−2.49320	3.05090
O	1.06120	−1.51600	2.88860
C	−2.31970	−2.05460	3.31250
C	4.76480	3.19260	−1.27470
C	5.71230	4.35050	−1.02320
C	4.97290	5.68850	−1.10200
O	6.74920	4.38690	−1.99310
C	−3.53480	−1.62770	3.56410
Br	−3.95460	−0.44740	5.09710
C	7.68560	3.32240	−1.88820
H	3.95200	−2.43450	−0.41560
H	4.82310	−0.35220	0.41500
H	2.28010	−0.78970	−2.34510
H	3.16190	−2.27800	−2.66680
H	0.59280	−0.84650	−0.32630
H	3.11600	1.16300	−1.63300
H	2.76410	1.10510	0.07980
H	0.52280	−2.42660	−2.14160
H	−0.69240	−2.88570	0.41830
H	0.73570	−3.49810	1.20870
H	4.99030	2.23150	0.57530
H	−0.54700	−0.79590	1.86110
H	−0.76150	−3.42290	3.47710
H	1.76000	−1.39060	2.25160
H	4.28660	3.17940	−2.24360
H	6.15010	4.25650	−0.02750
H	5.65220	6.51830	−0.90640
H	4.53770	5.84190	−2.08980
H	4.16820	5.73780	−0.36810
H	−4.40780	−1.86990	2.97580
H	7.21900	2.35220	−2.06280
H	8.46760	3.45120	−2.63630
H	8.16130	3.30900	−0.90680

**Marilzafurollene C_108**			
C	3.71270	−1.76940	−1.62070
O	3.03150	−1.47600	−0.40170
C	5.19350	−1.33160	−1.56920
C	2.85120	−1.16820	−2.73730
C	1.74730	−0.91320	−0.67970
C	5.48100	0.18420	−1.52300
Br	6.12160	−2.08020	−3.16440
C	1.45900	−1.25920	−2.14600
C	0.69870	−1.36590	0.35460
C	4.83710	0.88690	−0.34410
Cl	0.82490	−2.92110	−2.34030
C	0.98890	−0.84620	1.77380
C	−0.14580	−1.16820	2.72620
O	2.16990	−1.46520	2.23580
C	−0.86490	−0.25400	3.33700
C	4.03070	1.95870	−0.45170
C	3.33040	2.68770	0.68910
C	3.71420	2.18030	2.08610
O	1.91830	2.55630	0.58530
C	−1.59940	0.64730	3.94540
Br	−1.13410	1.38030	5.72480
C	1.32100	3.35320	−0.42820
H	3.68540	−2.85600	−1.71780
H	5.65610	−1.80610	−0.70260
H	3.09840	−0.12110	−2.90580
H	2.95540	−1.69430	−3.68670
H	1.85290	0.16980	−0.61650
H	6.55810	0.33780	−1.45150
H	5.17350	0.66020	−2.45390
H	0.75120	−0.57280	−2.61200
H	−0.27680	−0.99520	0.03710
H	0.62050	−2.45370	0.37020
H	5.05600	0.46290	0.62470
H	1.14920	0.23380	1.74090
H	−0.34390	−2.21740	2.88980
H	2.75540	−1.53320	1.48550
H	3.84230	2.35910	−1.43660
H	3.60660	3.74220	0.63220
H	3.20130	2.75530	2.85710
H	3.43710	1.13490	2.22410
H	4.78520	2.27640	2.26490
H	−2.50970	1.07100	3.54750
H	0.23910	3.22430	−0.39760
H	1.53380	4.41220	−0.27700
H	1.65790	3.06620	−1.42430

**Marilzafurollene C_109**			
C	3.13030	−1.73670	−1.07470
O	2.38870	−1.88370	0.13590
C	3.41120	−0.24920	−1.36170
C	2.32740	−2.46650	−2.16100
C	1.17080	−2.57290	−0.12450
C	4.32030	0.38320	−0.29450
Br	1.69220	0.76730	−1.43030
C	1.47920	−3.43060	−1.35580
C	0.66560	−3.31830	1.12650
C	4.72640	1.80170	−0.64340
Cl	2.44580	−4.87980	−0.94730
C	0.34930	−2.38250	2.30810
C	−0.76090	−1.39590	1.99640
O	−0.03150	−3.16930	3.41850
C	−0.56800	−0.09900	1.91250
C	4.42850	2.88360	0.09660
C	4.82340	4.31300	−0.23570
C	3.60620	5.11570	−0.70130
O	5.79730	4.38040	−1.27080
C	−0.34710	1.19010	1.80920
Br	−0.48850	2.42250	3.35210
C	7.12780	4.14440	−0.82880
H	4.07570	−2.26380	−0.93630
H	3.88050	−0.15510	−2.34210
H	1.67660	−1.77420	−2.69540
H	2.95880	−2.96150	−2.89930
H	0.43210	−1.82750	−0.42290
H	3.82050	0.36380	0.67510
H	5.23110	−0.20630	−0.18570
H	0.58260	−3.75720	−1.88370
H	−0.22290	−3.89530	0.86720
H	1.41760	−4.04080	1.44340
H	5.28980	1.92580	−1.55730
H	1.25430	−1.83290	2.57880
H	−1.74390	−1.81590	1.84180
H	0.74870	−3.53420	3.81160
H	3.85590	2.75900	1.00480
H	5.21540	4.77990	0.66990
H	3.88120	6.14790	−0.91840
H	3.17790	4.68730	−1.60800
H	2.82750	5.13210	0.06160
H	−0.07220	1.69570	0.89470
H	7.23460	3.16500	−0.36160
H	7.80720	4.18420	−1.68020
H	7.44430	4.90550	−0.11440

**Marilzafurollene C_110**			
C	3.21340	−0.29190	−0.73240
O	1.88150	0.07660	−0.37520
C	3.59290	0.28100	−2.11380
C	3.27620	−1.82020	−0.62230
C	1.13710	−1.07890	0.00410
C	3.59050	1.81900	−2.17010
Br	2.30320	−0.37400	−3.48850
C	2.19820	−2.11330	0.39980
C	0.07650	−0.72130	1.06300
C	4.64450	2.45580	−1.28380
Cl	2.84130	−1.83030	2.04660
C	−0.72790	−1.92180	1.59900
C	−1.72090	−1.48540	2.65850
O	−1.42670	−2.53510	0.53580
C	−1.70720	−1.93360	3.89340
C	5.70310	3.14740	−1.73740
C	6.75640	3.78720	−0.85210
C	6.80700	5.30120	−1.07200
O	8.05000	3.27880	−1.14360
C	−1.69780	−2.40030	5.11960
Br	−2.70770	−4.01940	5.64550
C	8.26300	1.94180	−0.71210
H	3.87290	0.11980	0.03200
H	4.57760	−0.08680	−2.40560
H	3.01220	−2.29350	−1.56800
H	4.25990	−2.18870	−0.33060
H	0.63260	−1.43590	−0.89580
H	3.75400	2.13920	−3.19970
H	2.61070	2.20180	−1.88150
H	1.82860	−3.13800	0.34800
H	0.56270	−0.21400	1.89690
H	−0.60640	0.01200	0.63280
H	4.50880	2.33380	−0.21850
H	−0.04510	−2.65800	2.02840
H	−2.46300	−0.76360	2.34790
H	−1.90370	−3.27990	0.87540
H	5.84490	3.27440	−2.80120
H	6.51820	3.60000	0.19690
H	7.55380	5.76190	−0.42510
H	7.06700	5.54280	−2.10290
H	5.84490	5.76370	−0.85030
H	−1.15350	−1.96220	5.94360
H	7.59010	1.24320	−1.21050
H	9.28440	1.64230	−0.94670
H	8.12710	1.84830	0.36610

**Marilzafurollene C_111**			
C	3.04700	−1.83820	−1.09170
O	2.42750	−1.90360	0.19170
C	3.80240	−0.50640	−1.25720
C	1.93660	−2.12580	−2.11010
C	1.07410	−2.33490	0.06260
C	2.93190	0.75240	−1.08830
Br	4.68470	−0.48040	−3.04530
C	0.99580	−3.00630	−1.31440
C	0.64050	−3.19560	1.26590
C	3.75830	2.02270	−1.05530
Cl	1.61520	−4.68520	−1.29160
C	0.59210	−2.41850	2.59390
C	−0.49210	−1.35680	2.60210
O	0.33640	−3.33970	3.63420
C	−0.25120	−0.07200	2.73200
C	3.83530	2.85400	−0.00170
C	4.65330	4.13350	0.05720
C	3.73810	5.36020	0.04650
O	5.54660	4.26130	−1.04250
C	−0.02180	1.21550	2.83650
Br	0.27360	2.37000	1.25660
C	6.74900	3.51540	−0.90300
H	3.76870	−2.65570	−1.13250
H	4.60130	−0.48190	−0.51450
H	1.41800	−1.21270	−2.39900
H	2.30640	−2.59900	−3.02020
H	0.45890	−1.43480	0.01840
H	2.20720	0.83860	−1.89690
H	2.36020	0.67830	−0.16260
H	−0.01640	−3.02690	−1.71960
H	−0.33630	−3.63730	1.06550
H	1.33420	−4.03000	1.37410
H	4.30990	2.25380	−1.95600
H	1.56910	−1.96320	2.77310
H	−1.50630	−1.71310	2.49180
H	0.56990	−2.93830	4.45930
H	3.27600	2.62230	0.89350
H	5.21740	4.13610	0.99180
H	4.31960	6.27940	0.11870
H	3.15570	5.40640	−0.87410
H	3.04020	5.34080	0.88400
H	0.02630	1.76170	3.76730
H	6.55660	2.44890	−0.78170
H	7.36270	3.64380	−1.79450
H	7.32850	3.86240	−0.04670

**Marilzafurollene C_112**			
C	2.42250	0.20690	−0.06380
O	1.82500	−0.50190	1.02030
C	1.51010	1.37160	−0.49490
C	2.68280	−0.84800	−1.14690
C	1.74530	−1.89120	0.72080
C	2.02700	2.17040	−1.70730
Br	1.29140	2.61770	1.04680
C	2.82690	−2.12060	−0.33850
C	1.83160	−2.74690	2.00070
C	3.43380	2.70340	−1.51150
Cl	4.46900	−2.20020	0.36800
C	0.68290	−2.47190	2.98970
C	−0.67820	−2.81260	2.41210
O	0.89120	−3.23490	4.16060
C	−1.56360	−1.90000	2.08270
C	4.48160	2.38540	−2.28960
C	5.89440	2.89480	−2.07850
C	6.36770	3.70790	−3.28580
O	6.80720	1.81900	−1.91390
C	−2.39920	−0.96270	1.70430
Br	−2.34640	−0.18840	−0.11730
C	6.66390	1.11000	−0.68990
H	3.37610	0.59270	0.29900
H	0.51390	0.98590	−0.71720
H	1.82030	−0.93360	−1.80910
H	3.55640	−0.62420	−1.75990
H	0.78130	−2.05800	0.23760
H	2.00040	1.54410	−2.59970
H	1.36080	3.01090	−1.90460
H	2.66480	−3.02500	−0.92600
H	1.83960	−3.80340	1.72980
H	2.78020	−2.55120	2.50050
H	3.57440	3.37640	−0.67700
H	0.70200	−1.41550	3.26920
H	−0.89200	−3.86240	2.27500
H	1.57650	−2.82450	4.66930
H	4.34590	1.71580	−3.12670
H	5.92540	3.53690	−1.19600
H	7.37810	4.08470	−3.12670
H	6.37880	3.10120	−4.19160
H	5.71770	4.56520	−3.46250
H	−3.17080	−0.53060	2.32450
H	5.69460	0.61600	−0.61890
H	7.43100	0.33870	−0.62400
H	6.78160	1.77260	0.16850

**Marilzafurollene C_113**			
C	3.20980	−0.97650	−0.12250
O	2.06590	−1.09420	0.72350
C	3.77700	0.45190	−0.04370
C	2.72100	−1.40190	−1.51130
C	1.00180	−1.76130	0.04530
C	5.04840	0.64600	−0.88950
Br	4.20990	0.84550	1.86360
C	1.64820	−2.41830	−1.18070
C	0.23030	−2.69500	0.99870
C	5.53580	2.08210	−0.88100
Cl	2.41060	−3.98990	−0.79290
C	−0.49810	−1.94250	2.12630
C	−1.40880	−2.86580	2.91210
O	0.46720	−1.39580	2.99840
C	−2.71460	−2.72980	2.96080
C	5.63660	2.85990	−1.97170
C	6.12550	4.29630	−1.95990
C	5.04210	5.24510	−2.47880
O	7.26080	4.45920	−2.79730
C	−4.02060	−2.60740	2.99900
Br	−4.95030	−1.51350	4.36230
C	8.45070	3.87290	−2.28720
H	3.95370	−1.69070	0.23450
H	3.01210	1.16770	−0.34790
H	2.27390	−0.55480	−2.03320
H	3.51480	−1.80510	−2.14100
H	0.32480	−0.98420	−0.31410
H	5.84670	0.00250	−0.51790
H	4.86180	0.34580	−1.92100
H	0.94370	−2.57930	−1.99760
H	−0.50390	−3.24960	0.41260
H	0.89870	−3.44090	1.43080
H	5.81660	2.48270	0.08320
H	−1.08260	−1.12400	1.70020
H	−0.92230	−3.66500	3.45210
H	1.19390	−1.08340	2.46370
H	5.36110	2.46720	−2.93990
H	6.37930	4.59000	−0.93930
H	5.38910	6.27820	−2.45630
H	4.76910	5.00930	−3.50760
H	4.14050	5.18460	−1.86860
H	−4.70450	−3.07850	2.30840
H	8.71430	4.29470	−1.31650
H	9.27570	4.07010	−2.97160
H	8.35760	2.79120	−2.18390

**Marilzafurollene C_114**			
C	2.74380	−0.29970	−0.93560
O	2.20920	−0.74020	0.31250
C	1.94550	0.90710	−1.47450
C	2.75190	−1.52910	−1.85360
C	1.86870	−2.12040	0.23400
C	2.08510	2.16940	−0.59960
Br	0.00740	0.43330	−1.54550
C	2.77990	−2.67680	−0.86450
C	1.94970	−2.80610	1.61220
C	3.45530	2.83410	−0.58960
Cl	4.45550	−2.93360	−0.29210
C	0.97330	−2.21620	2.64630
C	−0.47810	−2.36810	2.23030
O	1.17300	−2.88060	3.87730
C	−1.26440	−1.34610	1.97900
C	4.47820	2.59260	−1.43030
C	5.82750	3.28180	−1.35690
C	6.10950	4.06700	−2.64030
O	6.87610	2.33620	−1.20620
C	−2.02630	−0.31160	1.71390
Br	−3.13050	0.57610	3.09670
C	6.91270	1.70020	0.06470
H	3.77940	−0.01390	−0.75050
H	2.24360	1.13200	−2.49890
H	1.83510	−1.58080	−2.44140
H	3.59200	−1.53790	−2.54840
H	0.84410	−2.17850	−0.13640
H	1.36610	2.91990	−0.92910
H	1.81650	1.92230	0.42840
H	2.41670	−3.61510	−1.28490
H	1.76110	−3.87390	1.49590
H	2.96340	−2.71660	2.00330
H	3.58950	3.57820	0.18290
H	1.21090	−1.15900	2.78790
H	−0.84770	−3.37940	2.14170
H	0.74010	−2.38580	4.55890
H	4.38390	1.86510	−2.22170
H	5.84110	3.97820	−0.51620
H	7.07370	4.57220	−2.58090
H	6.13300	3.40940	−3.50960
H	5.34680	4.82680	−2.81310
H	−2.12470	0.14760	0.74070
H	6.01490	1.10900	0.24920
H	7.76680	1.02450	0.10840
H	7.02220	2.42900	0.86870

**Marilzafurollene C_115**			
C	2.73950	−1.63990	−1.93950
O	2.59140	−1.49670	−0.52570
C	2.43120	−0.31630	−2.67270
C	1.83080	−2.81220	−2.33610
C	1.61010	−2.40730	−0.04480
C	3.53220	0.75740	−2.55600
Br	0.69670	0.44040	−2.03450
C	1.67340	−3.57220	−1.03390
C	1.84400	−2.74280	1.43980
C	3.85470	1.16530	−1.13040
Cl	3.10550	−4.60900	−0.76110
C	1.60760	−1.53930	2.37180
C	0.15470	−1.10000	2.38970
O	1.99310	−1.90040	3.68230
C	−0.25960	0.06220	1.93790
C	3.67980	2.39790	−0.62690
C	4.01350	2.79310	0.79900
C	2.75610	3.22960	1.55170
O	4.92580	3.88110	0.83180
C	−0.67990	1.20910	1.45900
Br	−0.84300	2.83580	2.57570
C	6.24670	3.54630	0.42890
H	3.77350	−1.92990	−2.13250
H	2.29830	−0.53230	−3.73390
H	0.85550	−2.44690	−2.66030
H	2.24250	−3.41970	−3.14240
H	0.63390	−1.93460	−0.16090
H	4.45200	0.38790	−3.01050
H	3.24040	1.63870	−3.12840
H	0.78450	−4.20350	−1.00900
H	1.19160	−3.56590	1.73420
H	2.86540	−3.10270	1.56590
H	4.25320	0.38840	−0.49360
H	2.23700	−0.70550	2.05040
H	−0.54980	−1.80770	2.80140
H	2.93870	−1.89850	3.73120
H	3.27710	3.18130	−1.25240
H	4.44580	1.93910	1.32440
H	2.99220	3.50670	2.57910
H	2.28380	4.08860	1.07480
H	2.02650	2.42110	1.58670
H	−0.97200	1.37710	0.43240
H	6.28120	3.21060	−0.60810
H	6.88640	4.42460	0.51390
H	6.66700	2.76470	1.06300

**Marilzafurollene C_116**			
C	3.05150	−1.42330	−0.87400
O	2.29280	−1.73120	0.29520
C	3.13100	0.10120	−1.08180
C	2.39990	−2.20200	−2.02550
C	1.19770	−2.57360	−0.04760
C	3.91210	0.79720	0.04550
Br	1.28880	0.87110	−1.17150
C	1.67080	−3.31600	−1.30130
C	0.76120	−3.44510	1.14640
C	4.12890	2.27380	−0.22450
Cl	2.82010	−4.63200	−0.91590
C	0.25720	−2.62470	2.34840
C	−0.98180	−1.80990	2.02550
O	−0.03810	−3.51130	3.40850
C	−0.99440	−0.49620	2.00470
C	5.33690	2.85360	−0.35000
C	5.61550	4.32870	−0.61940
C	4.36220	5.21560	−0.61070
O	6.22930	4.51500	−1.88770
C	−0.97910	0.81510	1.96370
Br	−1.38240	1.93610	3.54500
C	7.58900	4.10820	−1.95870
H	4.05550	−1.82130	−0.71830
H	3.61600	0.30990	−2.03690
H	1.67590	−1.58170	−2.55430
H	3.12110	−2.56440	−2.75850
H	0.37020	−1.92750	−0.34440
H	3.37930	0.68570	0.99070
H	4.87980	0.31150	0.17680
H	0.85030	−3.73990	−1.88130
H	−0.01750	−4.13640	0.82200
H	1.60120	−4.06030	1.46860
H	3.22900	2.86410	−0.31620
H	1.05340	−1.95420	2.68120
H	−1.87850	−2.37070	1.80670
H	0.77390	−3.76690	3.82310
H	6.21490	2.23310	−0.24860
H	6.28220	4.69010	0.16560
H	4.63100	6.26010	−0.76850
H	3.66920	4.93440	−1.40410
H	3.83650	5.15380	0.34250
H	−0.75060	1.40030	1.08460
H	7.70230	3.03470	−1.80800
H	7.98550	4.34430	−2.94620
H	8.19890	4.63250	−1.22190

**Marilzafurollene C_117**			
C	2.87320	−1.30400	−0.52900
O	1.67390	−1.04710	0.20570
C	3.30610	−0.03600	−1.28970
C	2.57430	−2.51500	−1.42490
C	0.80020	−2.17070	0.14340
C	3.64380	1.12620	−0.34080
Br	1.85480	0.53380	−2.53790
C	1.07790	−2.69700	−1.26670
C	1.13510	−3.13970	1.30280
C	4.20450	2.32970	−1.07340
Cl	0.55680	−4.38260	−1.54190
C	0.85220	−2.54680	2.69560
C	−0.63140	−2.36300	2.95430
O	1.37790	−3.42060	3.67410
C	−1.19120	−1.19780	3.18630
C	5.43120	2.84010	−0.87510
C	5.98860	4.04580	−1.60870
C	7.24670	3.67110	−2.39620
O	6.35360	5.07570	−0.70150
C	−1.76390	−0.03760	3.40140
Br	−2.48700	1.07570	1.93350
C	5.25080	5.73780	−0.09690
H	3.65540	−1.58210	0.17920
H	4.18120	−0.26520	−1.89990
H	2.86580	−2.37400	−2.46600
H	3.10390	−3.39090	−1.04880
H	−0.23670	−1.84230	0.22320
H	2.75080	1.43240	0.20570
H	4.36550	0.79400	0.40640
H	0.55420	−2.07100	−1.99070
H	0.56050	−4.06000	1.19360
H	2.18230	−3.43460	1.23800
H	3.54880	2.79050	−1.79910
H	1.36390	−1.58640	2.79300
H	−1.22950	−3.26230	2.94110
H	2.31490	−3.29440	3.72310
H	6.09100	2.38690	−0.14920
H	5.24370	4.42520	−2.31120
H	7.63810	4.53460	−2.93430
H	8.03390	3.30790	−1.73500
H	7.03540	2.89150	−3.12860
H	−1.89580	0.41520	4.37310
H	5.61610	6.53040	0.55610
H	4.60470	6.19500	−0.84740
H	4.65290	5.05760	0.51060

**Marilzafurollene C_118**			
C	2.45640	0.19650	−0.09210
O	1.85930	−0.51990	0.98690
C	1.54000	1.35880	−0.52100
C	2.72420	−0.85270	−1.17880
C	1.80370	−1.91120	0.68950
C	2.05830	2.16700	−1.72650
Br	1.30600	2.59520	1.02620
C	2.88540	−2.12510	−0.37340
C	1.90300	−2.76900	1.96750
C	3.46100	2.70730	−1.52050
Cl	4.53130	−2.18690	0.32600
C	0.76890	−2.49370	2.97260
C	−0.60160	−2.80570	2.40070
O	0.98340	−3.28720	4.12200
C	−1.48510	−1.87700	2.11350
C	4.51450	2.40000	−2.29520
C	5.92330	2.91630	−2.07370
C	6.39800	3.73960	−3.27350
O	6.84150	1.84490	−1.91100
C	−2.32270	−0.92650	1.77410
Br	−2.29130	−0.09770	−0.02370
C	6.69520	1.12670	−0.69260
H	3.40730	0.58480	0.27510
H	0.54720	0.96880	−0.75040
H	1.86020	−0.94610	−1.83810
H	3.59320	−0.61780	−1.79430
H	0.84150	−2.09270	0.20820
H	2.04010	1.54520	−2.62230
H	1.38820	3.00460	−1.92280
H	2.73220	−3.03020	−0.96210
H	1.90820	−3.82510	1.69580
H	2.85540	−2.57600	2.46170
H	3.59330	3.37600	−0.68140
H	0.81720	−1.44320	3.27020
H	−0.82330	−3.84880	2.22650
H	0.49040	−2.91650	4.84050
H	4.38710	1.73460	−3.13690
H	5.94600	3.55310	−1.18700
H	7.40550	4.12130	−3.10690
H	6.41730	3.13860	−4.18300
H	5.74390	4.59420	−3.44830
H	−3.08580	−0.51110	2.41610
H	6.80380	1.78410	0.17090
H	5.72860	0.62650	−0.63080
H	7.46660	0.35950	−0.62730

**Marilzafurollene C_119**			
C	2.93210	−0.72970	−1.30450
O	1.52550	−0.63630	−1.06810
C	3.29920	0.01700	−2.60450
C	3.26050	−2.23060	−1.31000
C	1.05680	−1.83530	−0.45790
C	2.95770	1.52130	−2.57160
Br	2.29630	−0.79650	−4.12490
C	1.89670	−2.88440	−1.18940
C	1.26420	−1.75880	1.07460
C	3.72170	2.36340	−1.55930
Cl	1.96240	−4.46980	−0.37100
C	0.48100	−0.61510	1.74390
C	0.54730	−0.70520	3.25560
O	1.03640	0.61490	1.33190
C	−0.51220	−0.84470	4.01940
C	4.85330	2.02320	−0.91580
C	5.55110	2.89950	0.10680
C	6.97970	3.22510	−0.33590
O	5.63920	2.24360	1.36360
C	−1.57790	−0.99490	4.76960
Br	−2.28780	−2.77650	5.26040
C	4.39410	2.09810	2.03560
H	3.44230	−0.27740	−0.45400
H	4.35810	−0.11630	−2.82910
H	3.79470	−2.55980	−2.20170
H	3.88000	−2.47430	−0.44610
H	−0.00330	−1.97260	−0.67440
H	3.12870	1.95680	−3.55650
H	1.89210	1.63880	−2.37050
H	1.48090	−3.04470	−2.18560
H	0.92750	−2.69970	1.51210
H	2.32290	−1.68010	1.32400
H	3.28670	3.33230	−1.35760
H	−0.56090	−0.65070	1.41730
H	1.53290	−0.64690	3.69360
H	1.16580	0.55500	0.38870
H	5.32830	1.07170	−1.09900
H	5.00380	3.83700	0.22400
H	6.98640	3.74660	−1.29330
H	7.47440	3.86580	0.39430
H	7.57770	2.31960	−0.44110
H	−2.15990	−0.18480	5.18420
H	3.91820	3.06480	2.20460
H	3.70460	1.46360	1.47840
H	4.55650	1.63200	3.00730

**Marilzafurollene C_120**			
C	3.03530	−1.63250	−1.25060
O	2.31200	−1.46560	−0.03050
C	3.56510	−0.27740	−1.75550
C	2.08620	−2.35660	−2.21470
C	1.00520	−2.02240	−0.15920
C	4.61000	0.32640	−0.80160
Br	2.04750	1.00660	−1.95250
C	1.13870	−3.06860	−1.27100
C	0.47680	−2.52640	1.19770
C	5.23090	1.59910	−1.34480
Cl	1.89820	−4.58000	−0.68820
C	0.26290	−1.39460	2.21820
C	−0.42600	−1.89720	3.47150
O	1.52240	−0.86190	2.56510
C	−1.58960	−1.45070	3.88610
C	5.16330	2.79450	−0.73070
C	5.75670	4.10930	−1.22500
C	6.35190	4.03590	−2.63830
O	6.80060	4.56030	−0.37240
C	−2.75760	−1.00340	4.28210
Br	−4.46180	−1.82360	3.69730
C	6.36950	5.05560	0.88800
H	3.87600	−2.29480	−1.03790
H	4.00700	−0.40950	−2.74420
H	1.52460	−1.64330	−2.81840
H	2.60310	−3.03180	−2.89720
H	0.35030	−1.22800	−0.52240
H	4.15540	0.51810	0.17140
H	5.41450	−0.38980	−0.63120
H	0.18460	−3.32440	−1.73340
H	−0.47450	−3.03280	1.02790
H	1.14850	−3.27620	1.61750
H	5.75270	1.49870	−2.28450
H	−0.33330	−0.60140	1.76110
H	0.09700	−2.66090	4.02830
H	2.04500	−0.84360	1.76640
H	4.62970	2.85540	0.20650
H	4.95430	4.84910	−1.24210
H	6.71620	5.01450	−2.95130
H	7.19500	3.34600	−2.68090
H	5.60730	3.71550	−3.36750
H	−2.90400	−0.16610	4.94880
H	5.90620	4.27790	1.49510
H	7.22940	5.43400	1.44070
H	5.66200	5.87740	0.77050

**Marilzafurollene C_121**			
C	2.45960	−1.19220	−1.88950
O	2.38630	−0.88790	−0.49500
C	1.77030	−0.10830	−2.74430
C	1.85290	−2.59200	−2.03570
C	1.87700	−2.00260	0.23140
C	2.57210	1.20030	−2.88900
Br	−0.03450	0.30000	−1.99740
C	2.12700	−3.20790	−0.67980
C	2.49770	−2.07210	1.63920
C	2.94850	1.83390	−1.56380
Cl	3.82300	−3.77380	−0.60560
C	1.98560	−0.96750	2.58050
C	0.55680	−1.21500	3.02650
O	2.81930	−0.93660	3.72100
C	−0.44040	−0.40000	2.76850
C	4.19930	2.17760	−1.21490
C	4.58320	2.78090	0.12220
C	5.14920	4.19160	−0.05770
O	5.58660	2.00670	0.76360
C	−1.45460	0.38950	2.50590
Br	−2.57390	0.17330	0.88900
C	5.13360	0.74560	1.23850
H	3.51570	−1.24930	−2.15820
H	1.60440	−0.50570	−3.74660
H	0.77550	−2.52750	−2.19340
H	2.27900	−3.16020	−2.86290
H	0.79680	−1.86950	0.31160
H	3.48070	1.00800	−3.46100
H	1.99180	1.92180	−3.46470
H	1.47690	−4.05360	−0.45330
H	2.30310	−3.04900	2.08320
H	3.58150	−1.99340	1.54670
H	2.13590	1.99900	−0.87030
H	2.06130	−0.00230	2.07420
H	0.38550	−2.12330	3.58640
H	2.61840	−0.15990	4.22360
H	5.01300	2.01610	−1.90670
H	3.70210	2.84050	0.76430
H	4.42190	4.84960	−0.53380
H	5.41270	4.62880	0.90540
H	6.04810	4.18080	−0.67440
H	−1.77540	1.21260	3.12750
H	4.33150	0.86740	1.96610
H	4.77730	0.10690	0.42950
H	5.95320	0.22470	1.73300

**Marilzafurollene C_122**			
C	3.07460	−1.23280	−0.15300
O	2.11420	−1.59310	0.83910
C	3.57280	0.20400	0.08610
C	2.36140	−1.45750	−1.49010
C	0.99260	−2.24490	0.24650
C	4.63600	0.65210	−0.93370
Br	4.36630	0.29200	1.91470
C	1.44150	−2.61720	−1.17200
C	0.49560	−3.40580	1.13130
C	5.05040	2.09920	−0.74620
Cl	2.36770	−4.14780	−1.22000
C	−0.11860	−2.93990	2.46430
C	−1.43880	−2.21490	2.27190
O	−0.33900	−4.07290	3.28020
C	−1.62370	−0.94910	2.57460
C	4.91710	3.05790	−1.67790
C	5.33420	4.50460	−1.48750
C	4.13550	5.44370	−1.64790
O	6.30100	4.89150	−2.45350
C	−1.79870	0.32320	2.84690
Br	−1.48250	1.74330	1.50440
C	7.58690	4.31890	−2.25400
H	3.90900	−1.93100	−0.06760
H	2.72570	0.89150	0.07480
H	1.76830	−0.58150	−1.75630
H	3.04580	−1.66570	−2.31290
H	0.20660	−1.49410	0.15140
H	5.52140	0.02100	−0.85020
H	4.25880	0.52540	−1.94880
H	0.60810	−2.70710	−1.86960
H	−0.23630	−3.99900	0.58100
H	1.33440	−4.07180	1.33780
H	5.48300	2.34950	0.21250
H	0.59070	−2.28570	2.97900
H	−2.24920	−2.80310	1.86530
H	0.49650	−4.37120	3.61330
H	4.48960	2.81660	−2.64060
H	5.74500	4.63990	−0.48450
H	4.43350	6.48120	−1.49550
H	3.70260	5.36610	−2.64590
H	3.35410	5.21310	−0.92280
H	−2.12410	0.71360	3.80050
H	7.56290	3.23130	−2.33260
H	8.27300	4.69060	−3.01530
H	7.99280	4.58950	−1.27810

**Marilzafurollene C_123**			
C	2.99910	−0.75090	−0.57950
O	2.17330	−0.29430	0.49200
C	3.62430	0.43730	−1.33410
C	2.11980	−1.67180	−1.43500
C	0.87500	−0.87160	0.38680
C	4.59330	1.23870	−0.44890
Br	2.18370	1.65400	−1.99480
C	1.08240	−2.14680	−0.43930
C	0.23310	−1.03090	1.77890
C	5.31680	2.32780	−1.21710
Cl	1.76000	−3.48460	0.53760
C	−1.16040	−1.68850	1.76560
C	−1.71240	−1.85800	3.16890
O	−2.05720	−0.90930	0.99940
C	−2.06240	−3.01940	3.67330
C	6.64880	2.39010	−1.38020
C	7.36970	3.48230	−2.14820
C	8.14980	2.89550	−3.32740
O	8.30580	4.15950	−1.32230
C	−2.42300	−4.18060	4.16630
Br	−1.17020	−5.35030	5.15670
C	7.71570	4.99950	−0.33950
H	3.79230	−1.35510	−0.13610
H	4.15960	0.06530	−2.20910
H	1.62550	−1.11230	−2.22950
H	2.67670	−2.48560	−1.90000
H	0.27280	−0.17870	−0.20370
H	4.04950	1.69400	0.37990
H	5.32820	0.56630	−0.00440
H	0.16450	−2.49870	−0.91230
H	0.89910	−1.61670	2.41370
H	0.17500	−0.04900	2.24930
H	4.69230	3.09580	−1.65190
H	−1.09070	−2.67110	1.29400
H	−1.80650	−0.96090	3.76330
H	−2.12180	−0.04420	1.37810
H	7.27940	1.62770	−0.94580
H	6.64320	4.19770	−2.53880
H	8.65550	3.68240	−3.88710
H	8.90850	2.19000	−2.98790
H	7.48680	2.37160	−4.01640
H	−3.41320	−4.60470	4.08510
H	8.49930	5.50390	0.22580
H	7.08970	5.76570	−0.79870
H	7.11010	4.43210	0.36810

**Marilzafurollene C_124**			
C	3.57120	−1.89830	−1.48060
O	2.84550	−1.39400	−0.36150
C	4.68950	−0.93300	−1.93170
C	2.51250	−2.27020	−2.52450
C	1.44860	−1.65080	−0.52330
C	4.26200	0.45500	−2.45260
Br	5.73580	−1.83160	−3.36880
C	1.34490	−2.68560	−1.65220
C	0.79670	−2.02420	0.82250
C	3.61270	1.32790	−1.39550
Cl	1.59000	−4.35860	−1.06650
C	0.81930	−0.87200	1.84280
C	0.03920	−1.22090	3.09510
O	2.16210	−0.61290	2.18990
C	−1.04160	−0.58030	3.47950
C	4.16920	2.43070	−0.86870
C	3.51660	3.30010	0.18980
C	3.36870	4.74160	−0.30300
O	4.30210	3.33880	1.37150
C	−2.13150	0.04420	3.85910
Br	−2.09630	1.62530	5.05000
C	4.23490	2.15050	2.14880
H	4.04450	−2.82050	−1.13910
H	5.38050	−0.79440	−1.09850
H	2.22660	−1.40610	−3.12250
H	2.84300	−3.05440	−3.20640
H	0.99780	−0.72590	−0.88680
H	5.14720	0.97190	−2.82530
H	3.59060	0.36750	−3.30580
H	0.38810	−2.65070	−2.17450
H	−0.23930	−2.31040	0.63540
H	1.28090	−2.90280	1.25120
H	2.63480	1.02180	−1.05610
H	0.40130	0.02760	1.38530
H	0.42330	−2.04360	3.68050
H	2.69090	−0.79840	1.41710
H	5.14940	2.74650	−1.19600
H	2.52220	2.91330	0.42250
H	2.88780	5.36170	0.45360
H	4.33950	5.18480	−0.52650
H	2.76070	4.78890	−1.20680
H	−3.13410	−0.23330	3.56810
H	4.58280	1.27930	1.59260
H	4.86560	2.25510	3.03140
H	3.21600	1.96230	2.48940

**Marilzafurollene C_125**			
C	2.91050	−0.64260	−1.24120
O	2.04500	−0.70960	−0.10720
C	2.60400	0.61250	−2.08420
C	2.74660	−1.97600	−1.98250
C	1.38150	−1.97170	−0.07310
C	2.86640	1.93680	−1.34540
Br	0.68400	0.59480	−2.62830
C	2.28600	−2.90890	−0.88130
C	1.06910	−2.39950	1.37420
C	4.33170	2.16250	−1.02470
Cl	3.70020	−3.46430	0.06330
C	0.06660	−1.46640	2.07600
C	−0.35620	−2.01590	3.42440
O	0.68340	−0.21090	2.26050
C	−1.59050	−2.36080	3.71340
C	5.08480	3.14620	−1.54690
C	6.55330	3.39010	−1.23810
C	7.42520	3.04790	−2.44880
O	7.01710	2.60280	−0.14790
C	−2.81890	−2.72130	4.00120
Br	−4.14610	−1.46910	4.76960
C	6.70380	3.14140	1.13030
H	3.93230	−0.59830	−0.86310
H	3.19360	0.59210	−3.00220
H	1.97040	−1.90800	−2.74480
H	3.66230	−2.30710	−2.47300
H	0.44380	−1.85630	−0.62000
H	2.50720	2.76520	−1.95700
H	2.29380	1.96930	−0.41760
H	1.76230	−3.78880	−1.25660
H	0.65600	−3.40890	1.35070
H	1.98520	−2.46080	1.96300
H	4.77900	1.47190	−0.32390
H	−0.81130	−1.33040	1.44020
H	0.42530	−2.11170	4.16400
H	1.19540	−0.03460	1.47430
H	4.63960	3.83570	−2.25010
H	6.68460	4.44980	−1.01090
H	8.47610	3.24690	−2.23800
H	7.33370	1.99420	−2.71350
H	7.14370	3.63970	−3.32010
H	−3.22170	−3.71240	3.85210
H	5.62970	3.26880	1.26760
H	7.06210	2.46680	1.90790
H	7.18860	4.10720	1.27780

**Marilzafurollene C_126**			
C	2.68520	−0.69180	−1.07830
O	2.18940	−1.03890	0.21460
C	2.04680	0.62500	−1.56860
C	2.42700	−1.90990	−1.97640
C	1.60090	−2.33440	0.17520
C	2.41040	1.84790	−0.70800
Br	0.05810	0.44920	−1.55850
C	2.32240	−3.04280	−0.97530
C	1.65970	−3.02200	1.55330
C	3.88090	2.21350	−0.77880
Cl	3.96270	−3.58760	−0.51270
C	0.86120	−2.27820	2.64010
C	−0.62480	−2.21780	2.33790
O	1.05350	−2.93970	3.87400
C	−1.26310	−1.09830	2.08170
C	4.35930	3.35470	−1.30470
C	5.82760	3.74130	−1.38400
C	6.32070	3.70410	−2.83270
O	6.66080	2.86740	−0.63180
C	−1.87750	0.02680	1.80260
Br	−2.72560	1.15180	3.19370
C	6.70430	3.16260	0.75840
H	3.76490	−0.57190	−0.98580
H	2.34270	0.81450	−2.60150
H	1.48030	−1.80970	−2.50750
H	3.20790	−2.06420	−2.72140
H	0.55860	−2.20960	−0.12210
H	1.81820	2.70370	−1.03370
H	2.14160	1.66610	0.33340
H	1.77310	−3.90390	−1.35750
H	1.29020	−4.04410	1.46220
H	2.69930	−3.09990	1.87140
H	4.57270	1.49050	−0.37040
H	1.25200	−1.26220	2.73710
H	−1.15130	−3.16080	2.34150
H	1.90340	−2.69980	4.21560
H	3.66850	4.07690	−1.71620
H	5.93360	4.76270	−1.01340
H	7.36620	4.00650	−2.89400
H	6.24080	2.69950	−3.24900
H	5.74270	4.37780	−3.46560
H	−1.97980	0.44190	0.81020
H	5.71470	3.12600	1.21470
H	7.33360	2.43220	1.26660
H	7.13100	4.15060	0.93570

**Marilzafurollene C_127**			
C	2.70450	−1.88470	−1.46150
O	2.36890	−2.00690	−0.08030
C	3.56710	−0.62910	−1.68950
C	1.37590	−1.94920	−2.22680
C	0.96440	−2.19650	0.06870
C	2.92960	0.68360	−1.19850
Br	4.00430	−0.48220	−3.62970
C	0.49770	−2.74580	−1.28400
C	0.64650	−3.06150	1.30490
C	3.89280	1.85210	−1.26260
Cl	0.84410	−4.49230	−1.46060
C	0.96010	−2.35670	2.63820
C	0.03720	−1.18140	2.90890
O	0.84720	−3.28910	3.69550
C	0.45260	0.06220	2.99110
C	4.33630	2.52880	−0.19040
C	5.29000	3.70670	−0.25000
C	6.59630	3.38750	0.48120
O	4.73180	4.85450	0.37320
C	0.85320	1.31020	3.04600
Br	0.90040	2.47630	1.44710
C	3.63180	5.42310	−0.32480
H	3.29250	−2.76630	−1.72180
H	4.51310	−0.77520	−1.16550
H	0.95100	−0.95570	−2.36400
H	1.47570	−2.40410	−3.21280
H	0.51810	−1.20800	0.18880
H	2.04670	0.93480	−1.78500
H	2.59460	0.56160	−0.16780
H	−0.56750	−2.59350	−1.46130
H	−0.40280	−3.35730	1.28030
H	1.22710	−3.98310	1.24580
H	4.22660	2.13920	−2.24980
H	1.99670	−2.01140	2.61940
H	−1.01090	−1.40960	3.03620
H	0.01040	−3.72630	3.64120
H	4.00460	2.24340	0.79790
H	5.52400	3.93650	−1.29140
H	7.28690	4.22900	0.42560
H	6.41700	3.17420	1.53520
H	7.09180	2.52140	0.04180
H	1.18560	1.81440	3.94180
H	3.91120	5.70660	−1.34020
H	2.78450	4.73810	−0.37360
H	3.29880	6.32230	0.19340

**Marilzafurollene C_128**			
C	3.30720	−1.58540	−1.02190
O	2.40090	−1.48990	0.07780
C	3.79360	−0.18720	−1.44600
C	2.57300	−2.37450	−2.11400
C	1.17720	−2.14960	−0.24080
C	4.63040	0.49200	−0.34870
Br	2.22260	0.97060	−1.87300
C	1.55750	−3.16980	−1.31950
C	0.49850	−2.70970	1.02450
C	5.23600	1.80440	−0.80670
Cl	2.34220	−4.62180	−0.62900
C	0.04500	−1.61090	2.00190
C	−0.77510	−2.18290	3.14170
O	1.19370	−0.98280	2.52770
C	−2.03420	−1.87520	3.35610
C	4.97520	2.99910	−0.24840
C	5.56890	4.32620	−0.69130
C	4.51680	5.17670	−1.40720
O	6.66200	4.16000	−1.58670
C	−3.29640	−1.58270	3.56430
Br	−3.86650	−0.07180	4.70960
C	7.89350	3.85520	−0.94520
H	4.15860	−2.17960	−0.68580
H	4.38800	−0.27330	−2.35690
H	2.05070	−1.70310	−2.79600
H	3.23830	−2.99880	−2.71100
H	0.52000	−1.40700	−0.69750
H	4.01680	0.65270	0.53890
H	5.44800	−0.16310	−0.04610
H	0.70610	−3.49630	−1.91770
H	−0.37220	−3.29040	0.71690
H	1.16100	−3.40670	1.53930
H	5.91760	1.74980	−1.64350
H	−0.54160	−0.86330	1.46290
H	−0.26280	−2.87440	3.79460
H	1.82080	−0.90650	1.81210
H	4.28490	3.05290	0.58150
H	5.89960	4.86740	0.19730
H	4.93250	6.13900	−1.70590
H	4.15470	4.67670	−2.30610
H	3.65890	5.37000	−0.76290
H	−4.13190	−2.12210	3.14300
H	7.83020	2.94070	−0.35470
H	8.67080	3.71480	−1.69620
H	8.20640	4.66940	−0.29030

**Marilzafurollene C_129**			
C	3.04180	−0.66850	0.63020
O	1.69980	−0.18630	0.73830
C	3.98270	0.51490	0.33550
C	3.01080	−1.77920	−0.43230
C	0.79130	−1.12500	0.16370
C	3.67820	1.25860	−0.97900
Br	5.86160	−0.15270	0.30590
C	1.61390	−1.66620	−1.00800
C	0.37440	−2.16690	1.23000
C	4.53840	2.49440	−1.15570
Cl	1.01590	−3.21030	−1.67510
C	−0.41060	−1.55990	2.40660
C	−0.97680	−2.63600	3.31190
O	0.45820	−0.73900	3.15580
C	−2.26190	−2.79240	3.53440
C	5.36880	2.70020	−2.19140
C	6.24140	3.92940	−2.36280
C	5.88330	4.67870	−3.64860
O	7.61450	3.57760	−2.45160
C	−3.54890	−2.94440	3.73870
Br	−4.67480	−4.10550	2.59710
C	8.17350	3.09190	−1.23810
H	3.31890	−1.10880	1.58960
H	3.91780	1.21580	1.16920
H	3.78620	−1.69040	−1.19320
H	3.14340	−2.74740	0.05180
H	−0.09680	−0.60780	−0.20150
H	3.81180	0.59540	−1.83360
H	2.63510	1.57690	−0.98970
H	1.60970	−0.93660	−1.81900
H	−0.26400	−2.91100	0.75180
H	1.24050	−2.71530	1.60190
H	4.46170	3.24360	−0.38060
H	−1.22250	−0.94040	2.01860
H	−0.25710	−3.28940	3.78310
H	0.96760	−0.22980	2.53180
H	5.45100	1.95440	−2.96900
H	6.09390	4.60470	−1.51760
H	4.84030	4.99640	−3.64220
H	6.50110	5.56960	−3.76260
H	6.03920	4.05250	−4.52740
H	−4.10920	−2.46530	4.52820
H	7.70160	2.16270	−0.91690
H	9.23470	2.88980	−1.38200
H	8.07900	3.82600	−0.43700

**Marilzafurollene C_130**			
C	3.11730	−1.57010	−0.48320
O	1.95390	−1.12110	0.21560
C	3.88410	−0.36500	−1.06000
C	2.62080	−2.57300	−1.53550
C	0.86790	−2.01570	−0.00580
C	4.40000	0.57930	0.03920
Br	2.70130	0.65890	−2.30130
C	1.11340	−2.43240	−1.45750
C	0.91930	−3.15680	1.03900
C	5.26540	1.69560	−0.51270
Cl	0.25530	−3.91750	−1.95450
C	0.72230	−2.67210	2.48660
C	−0.68600	−2.16480	2.73560
O	0.97830	−3.75880	3.35300
C	−0.96150	−0.93330	3.10100
C	5.00190	3.00450	−0.36430
C	5.85770	4.12360	−0.92740
C	6.40640	5.00890	0.19430
O	5.10650	4.96230	−1.79330
C	−1.24940	0.30060	3.44220
Br	−1.60810	1.71360	2.10370
C	4.71990	4.34600	−3.01470
H	3.75800	−2.09870	0.22460
H	4.72660	−0.72840	−1.65010
H	3.00150	−2.38190	−2.53910
H	2.92280	−3.58230	−1.25350
H	−0.07860	−1.48040	0.07690
H	3.55780	0.99860	0.59200
H	4.99580	0.01830	0.75980
H	0.78650	−1.62870	−2.11900
H	0.15630	−3.90170	0.81140
H	1.87330	−3.67870	0.96450
H	6.14580	1.39120	−1.06020
H	1.45090	−1.88700	2.70350
H	−1.48170	−2.88210	2.59240
H	1.05630	−3.42920	4.23720
H	4.12190	3.31400	0.18140
H	6.70100	3.69930	−1.47590
H	7.01220	4.43050	0.89220
H	7.03350	5.80330	−0.21080
H	5.59940	5.47800	0.75770
H	−1.31960	0.66130	4.45800
H	4.04780	3.50320	−2.84940
H	4.19320	5.07000	−3.63620
H	5.58840	3.99520	−3.57350

**Marilzafurollene C_131**			
C	2.84080	−1.40780	−0.56250
O	2.11790	−1.80720	0.60130
C	3.05720	0.11610	−0.53340
C	2.01380	−1.91830	−1.75250
C	0.89840	−2.43900	0.23370
C	3.86410	0.63710	−1.73600
Br	4.03700	0.57840	1.14190
C	1.19240	−3.03720	−1.14350
C	0.42150	−3.41360	1.32890
C	4.00220	2.14700	−1.72760
Cl	2.18280	−4.52330	−1.04070
C	0.12260	−2.71440	2.66860
C	−1.01520	−1.71580	2.56420
O	−0.21040	−3.69240	3.63260
C	−0.83750	−0.41640	2.63960
C	5.17460	2.80280	−1.73670
C	5.31470	4.31330	−1.71210
C	6.03100	4.81590	−2.96800
O	6.07890	4.74200	−0.59440
C	−0.62490	0.87760	2.65890
Br	−0.23470	1.89980	1.00850
C	5.43110	4.56540	0.65880
H	3.80310	−1.92140	−0.53850
H	2.09120	0.61970	−0.47910
H	1.34380	−1.13660	−2.11290
H	2.62880	−2.24360	−2.59210
H	0.15390	−1.65310	0.09670
H	4.85110	0.17280	−1.75110
H	3.37300	0.35770	−2.66830
H	0.29020	−3.26770	−1.71130
H	−0.46750	−3.94200	0.98230
H	1.18600	−4.17340	1.49110
H	3.07980	2.71010	−1.70960
H	1.02480	−2.20000	3.00910
H	−2.00260	−2.12860	2.41810
H	0.58520	−4.12660	3.90760
H	6.10000	2.24520	−1.75340
H	4.32490	4.77250	−1.67220
H	6.11760	5.90250	−2.95570
H	7.03780	4.40380	−3.04010
H	5.48710	4.53610	−3.87060
H	−0.63810	1.49590	3.54450
H	6.06270	4.96180	1.45360
H	4.47970	5.09780	0.69130
H	5.24870	3.51260	0.87700

**Marilzafurollene C_132**			
C	2.85590	−0.56330	−1.49200
O	2.03780	−0.47010	−0.32460
C	2.43760	0.49580	−2.53260
C	2.74840	−2.01630	−1.97280
C	1.45110	−1.73760	−0.03670
C	2.67420	1.93910	−2.05650
Br	0.49420	0.29770	−2.94060
C	2.38030	−2.75760	−0.70390
C	1.21470	−1.91120	1.47610
C	4.14520	2.27760	−1.90970
Cl	3.85480	−3.06260	0.26190
C	0.18220	−0.92030	2.04160
C	−0.15890	−1.23390	3.48480
O	0.72460	0.37990	1.96680
C	−1.36940	−1.54040	3.89210
C	4.74200	2.55940	−0.73740
C	6.21020	2.91150	−0.52340
C	6.99400	3.14220	−1.82310
O	6.89440	1.88300	0.17920
C	−2.58140	−1.85630	4.28270
Br	−3.23290	−3.72540	4.31360
C	6.54280	1.75900	1.55060
H	3.88600	−0.38830	−1.17760
H	2.97550	0.32830	−3.46690
H	1.94460	−2.12770	−2.70080
H	3.66570	−2.38200	−2.43500
H	0.48980	−1.77160	−0.55290
H	2.24460	2.63720	−2.77540
H	2.15220	2.10830	−1.11350
H	1.89540	−3.71590	−0.89320
H	0.86180	−2.92820	1.65320
H	2.15240	−1.81570	2.02500
H	4.71640	2.27850	−2.82620
H	−0.72360	−0.95210	1.43180
H	0.66050	−1.19220	4.18750
H	1.20130	0.43300	1.14110
H	4.13830	2.54450	0.15860
H	6.24860	3.83580	0.05580
H	8.01880	3.44190	−1.60320
H	7.04360	2.23530	−2.42600
H	6.54430	3.93220	−2.42500
H	−3.32570	−1.14880	4.61770
H	5.50430	1.45300	1.67840
H	7.16770	0.99690	2.01610
H	6.70260	2.69450	2.08810

**Marilzafurollene C_133**			
C	2.96050	−1.83310	−1.32120
O	2.36210	−1.94000	−0.02990
C	3.22100	−0.35730	−1.67790
C	2.02920	−2.57790	−2.28750
C	1.12640	−2.64080	−0.12710
C	4.28510	0.27750	−0.76670
Br	1.52450	0.68730	−1.52150
C	1.28850	−3.52620	−1.36620
C	0.77210	−3.35710	1.19100
C	4.65350	1.68590	−1.19250
Cl	2.30290	−4.96440	−1.04420
C	0.55970	−2.39210	2.37180
C	−0.59960	−1.44000	2.14240
O	0.30580	−3.15720	3.53210
C	−0.45380	−0.13790	2.04750
C	4.53640	2.77040	−0.40460
C	4.88140	4.20860	−0.77600
C	5.23570	4.40550	−2.25680
O	5.99410	4.68410	−0.03050
C	−0.28650	1.15840	1.93500
Br	−0.37370	2.37870	3.49150
C	5.72660	4.94440	1.34060
H	3.90950	−2.37030	−1.28120
H	3.54630	−0.29200	−2.71710
H	1.31750	−1.89150	−2.74690
H	2.56360	−3.08640	−3.09040
H	0.35420	−1.90470	−0.35540
H	3.93550	0.27590	0.26680
H	5.19480	−0.32340	−0.78640
H	0.33640	−3.86610	−1.77510
H	−0.12440	−3.96050	1.04470
H	1.56940	−4.05410	1.45040
H	5.03320	1.78340	−2.19840
H	1.48080	−1.82610	2.53160
H	−1.57770	−1.89010	2.05220
H	0.39100	−2.59340	4.28820
H	4.15190	2.63420	0.59540
H	4.00690	4.82520	−0.56050
H	5.42250	5.45830	−2.46880
H	6.13630	3.85440	−2.52860
H	4.42320	4.08000	−2.90710
H	−0.08980	1.67590	1.00680
H	6.61810	5.36020	1.81010
H	4.92050	5.67000	1.45670
H	5.46270	4.03700	1.88390

**Marilzafurollene C_134**			
C	3.24640	−1.49830	−0.75010
O	2.38510	−1.79800	0.34790
C	3.48820	0.02050	−0.85060
C	2.60150	−2.13790	−1.98820
C	1.23370	−2.49320	−0.11760
C	4.24530	0.55970	0.38100
Br	1.73410	0.96720	−1.01150
C	1.71310	−3.20760	−1.38500
C	0.62520	−3.37980	0.98660
C	4.71790	2.00580	0.30380
Cl	2.69160	−4.65910	−1.01480
C	0.13640	−2.58150	2.20980
C	−0.97540	−1.60610	1.86970
O	−0.33090	−3.49100	3.18530
C	−0.83610	−0.30160	1.94340
C	4.61100	2.84080	−0.74600
C	5.10840	4.27400	−0.75510
C	3.95630	5.24790	−1.01480
O	6.06880	4.47720	−1.78140
C	−0.66780	0.99840	1.99980
Br	−1.05040	2.05320	3.63100
C	7.32490	3.85950	−1.53610
H	4.19400	−2.00480	−0.56000
H	4.06010	0.22290	−1.75690
H	1.98370	−1.41610	−2.52290
H	3.33200	−2.53300	−2.69460
H	0.50110	−1.74440	−0.42260
H	3.61720	0.45000	1.26610
H	5.12950	−0.05330	0.55890
H	0.89530	−3.50680	−2.04150
H	−0.20200	−3.95610	0.57030
H	1.36840	−4.10590	1.31580
H	5.18990	2.37280	1.20420
H	0.97950	−2.03260	2.63670
H	−1.91260	−2.04090	1.55520
H	0.41590	−3.86660	3.63010
H	4.14640	2.51520	−1.66430
H	5.55190	4.51640	0.21270
H	4.31020	6.27880	−1.00650
H	3.49360	5.06310	−1.98460
H	3.18340	5.15520	−0.25130
H	−0.31220	1.60860	1.18210
H	7.23800	2.77410	−1.47600
H	8.00970	4.09190	−2.35160
H	7.77130	4.22590	−0.61090

**Marilzafurollene C_135**			
C	3.26220	−1.67930	−1.85490
O	3.35290	−1.37080	−0.46590
C	4.51680	−1.22290	−2.63000
C	1.91670	−1.11480	−2.32540
C	2.11810	−0.82230	−0.00580
C	4.79360	0.29400	−2.68880
Br	4.36740	−1.89270	−4.50020
C	1.07990	−1.20890	−1.06720
C	1.83470	−1.25150	1.44630
C	5.11600	0.91060	−1.34120
Cl	0.48190	−2.88350	−0.85930
C	0.50470	−0.72620	2.01990
C	0.28150	−1.22870	3.43290
O	0.52240	0.68630	2.02880
C	−0.75020	−1.96090	3.78700
C	4.49170	1.98170	−0.82350
C	4.82580	2.60640	0.51860
C	3.61800	2.57950	1.45800
O	5.20650	3.96670	0.36850
C	−1.79320	−2.67630	4.13600
Br	−3.47980	−1.85390	4.76610
C	6.48080	4.15350	−0.23190
H	3.20920	−2.76770	−1.91360
H	5.38400	−1.72480	−2.19870
H	2.00560	−0.06930	−2.61630
H	1.49500	−1.66230	−3.16880
H	2.23210	0.26180	−0.03710
H	5.66010	0.46380	−3.32850
H	3.96890	0.82640	−3.16120
H	0.21900	−0.53960	−1.07740
H	1.84940	−2.34020	1.50660
H	2.65980	−0.91380	2.07420
H	5.90990	0.43850	−0.78030
H	−0.32270	−1.05390	1.38680
H	1.03200	−0.95480	4.16070
H	−0.30540	0.99760	2.36930
H	3.69540	2.45750	−1.37720
H	5.63870	2.05070	0.99050
H	3.85700	3.04990	2.41160
H	2.76730	3.11000	1.02990
H	3.30320	1.55740	1.66760
H	−1.83760	−3.75550	4.11540
H	6.70820	5.21840	−0.27840
H	7.26640	3.66970	0.34990
H	6.51030	3.76400	−1.25000

**Marilzafurollene C_136**			
C	2.40460	−0.91730	−1.13910
O	2.70100	−1.12080	0.24220
C	3.02500	0.39860	−1.64520
C	0.87930	−1.00410	−1.26400
C	1.50020	−1.32520	0.98120
C	4.56240	0.36510	−1.60560
Br	2.37920	1.92600	−0.53080
C	0.50170	−1.84630	−0.06380
C	1.78480	−2.20000	2.22220
C	5.18790	1.60870	−2.20740
Cl	0.75740	−3.57560	−0.44700
C	0.65800	−2.22790	3.27560
C	−0.56060	−3.00660	2.81970
O	1.15810	−2.82980	4.45180
C	−1.76420	−2.48700	2.73870
C	6.01120	2.43950	−1.54660
C	6.62810	3.69180	−2.14070
C	8.15610	3.60200	−2.13830
O	6.27520	4.84590	−1.39180
C	−2.95360	−1.94730	2.62030
Br	−3.54540	−1.06890	0.94840
C	4.90520	5.21210	−1.49250
H	2.83940	−1.75560	−1.68590
H	2.69050	0.58200	−2.66710
H	0.42240	−0.01880	−1.16960
H	0.55050	−1.43150	−2.21160
H	1.17040	−0.33900	1.31310
H	4.90250	0.23680	−0.57690
H	4.92870	−0.49760	−2.16300
H	−0.54010	−1.71080	0.22500
H	2.06230	−3.21260	1.92920
H	2.67670	−1.78680	2.69440
H	4.93670	1.81910	−3.23700
H	0.36930	−1.20410	3.52500
H	−0.39160	−4.04070	2.55520
H	1.79150	−2.24940	4.85010
H	6.26410	2.23360	−0.51630
H	6.29120	3.81220	−3.17220
H	8.59740	4.49590	−2.57940
H	8.54450	3.50620	−1.12410
H	8.50120	2.74250	−2.71350
H	−3.70530	−1.92620	3.39600
H	4.73190	6.12910	−0.92960
H	4.62000	5.39730	−2.52870
H	4.24860	4.44440	−1.08180

**Marilzafurollene C_137**			
C	3.18390	−1.23410	−0.82240
O	2.23130	−1.27530	0.24080
C	3.47460	0.22110	−1.23380
C	2.61750	−2.12120	−1.93890
C	1.13620	−2.11570	−0.11850
C	4.16500	1.01430	−0.11140
Br	1.76710	1.13490	−1.72490
C	1.70640	−3.06360	−1.17950
C	0.50670	−2.77640	1.12350
C	4.59660	2.39720	−0.56010
Cl	2.67680	−4.38130	−0.45660
C	−0.14550	−1.76300	2.08060
C	−0.91020	−2.45750	3.19050
O	0.87330	−0.96860	2.64740
C	−2.20870	−2.34690	3.35720
C	5.87160	2.82760	−0.57420
C	6.36360	4.20320	−1.01240
C	5.23970	5.19650	−1.34000
O	7.17270	4.11510	−2.17760
C	−3.50750	−2.25210	3.51850
Br	−4.34400	−0.85030	4.63870
C	8.46900	3.57300	−1.96500
H	4.09990	−1.69690	−0.45160
H	4.11090	0.22510	−2.12050
H	2.02450	−1.53440	−2.64060
H	3.39080	−2.63570	−2.51000
H	0.38930	−1.48220	−0.60100
H	3.49360	1.11400	0.74240
H	5.03790	0.46520	0.24390
H	0.93690	−3.51410	−1.80740
H	−0.25270	−3.48340	0.78660
H	1.24950	−3.36430	1.66400
H	3.79760	3.04870	−0.88260
H	−0.81920	−1.11220	1.51850
H	−0.32280	−3.06460	3.86380
H	1.50680	−0.79330	1.95520
H	6.64380	2.15010	−0.24110
H	6.94870	4.62390	−0.19250
H	5.65360	6.16900	−1.60680
H	4.63960	4.85550	−2.18410
H	4.57810	5.34590	−0.48640
H	−4.23460	−2.91180	3.06810
H	8.42840	2.53390	−1.63850
H	9.03010	3.60150	−2.89900
H	9.02460	4.15170	−1.22600

**Marilzafurollene C_138**			
C	2.85100	−1.42490	−0.60190
O	2.11950	−1.82720	0.55540
C	3.07150	0.09820	−0.56590
C	2.03080	−1.92890	−1.79910
C	0.91150	−2.47610	0.17800
C	3.88980	0.62090	−1.76000
Br	4.03810	0.55290	1.11930
C	1.21760	−3.06030	−1.20280
C	0.43730	−3.46370	1.26320
C	4.03260	2.13030	−1.74500
Cl	2.22230	−4.53750	−1.11020
C	0.14640	−2.78420	2.61430
C	−0.97260	−1.76340	2.52140
O	−0.21260	−3.78030	3.55010
C	−0.77590	−0.47010	2.64140
C	5.20700	2.78250	−1.74270
C	5.35140	4.29240	−1.71120
C	6.07810	4.79780	−2.96000
O	6.10890	4.71450	−0.58640
C	−0.54930	0.82040	2.70350
Br	−0.12080	1.88790	1.09190
C	5.45200	4.53440	0.66160
H	3.81170	−1.94150	−0.57350
H	2.10640	0.60390	−0.51820
H	1.35560	−1.14850	−2.15260
H	2.65020	−2.24070	−2.64060
H	0.15760	−1.69890	0.04260
H	4.87550	0.15340	−1.76860
H	3.40540	0.34610	−2.69710
H	0.32010	−3.29490	−1.77610
H	−0.45260	−3.98800	0.91350
H	1.20030	−4.22690	1.41790
H	3.11190	2.69610	−1.73200
H	1.06250	−2.30180	2.96460
H	−1.96370	−2.15390	2.34020
H	−0.09140	−3.43210	4.42230
H	6.13090	2.22220	−1.75440
H	4.36270	4.75450	−1.67650
H	6.16770	5.88410	−2.94290
H	7.08410	4.38310	−3.02660
H	5.53980	4.52310	−3.86740
H	−0.57080	1.41250	3.60670
H	5.26540	3.48110	0.87410
H	6.07900	4.92590	1.46240
H	4.50170	5.06900	0.68970

**Marilzafurollene C_139**			
C	3.09650	−1.26230	−0.18370
O	2.12470	−1.63300	0.79270
C	3.58340	0.17580	0.07060
C	2.40190	−1.48110	−1.53200
C	1.01350	−2.28520	0.18060
C	4.66710	0.62990	−0.92460
Br	4.33650	0.26460	1.91640
C	1.48250	−2.64690	−1.23440
C	0.49820	−3.45030	1.05030
C	5.06230	2.08170	−0.73280
Cl	2.41620	−4.17310	−1.28170
C	−0.09910	−2.99610	2.39460
C	−1.37860	−2.19850	2.21660
O	−0.38250	−4.14700	3.16520
C	−1.50080	−0.93630	2.56160
C	4.94680	3.03400	−1.67380
C	5.34390	4.48570	−1.48080
C	4.14130	5.41190	−1.68460
O	6.33560	4.87600	−2.42020
C	−1.61070	0.33490	2.86970
Br	−1.19580	1.77350	1.57400
C	7.62010	4.31570	−2.17970
H	3.93300	−1.95720	−0.09090
H	2.73340	0.85950	0.04240
H	1.80830	−0.60580	−1.79980
H	3.09790	−1.68070	−2.34740
H	0.22930	−1.53340	0.07720
H	5.55640	0.00780	−0.81670
H	4.31670	0.49560	−1.94840
H	0.65860	−2.73560	−1.94350
H	−0.24600	−4.02180	0.49400
H	1.32290	−4.13620	1.24880
H	5.46450	2.34080	0.23690
H	0.64720	−2.40170	2.92860
H	−2.21250	−2.72790	1.77760
H	−0.45980	−3.89040	4.07330
H	4.54950	2.78300	−2.64690
H	5.72290	4.63150	−0.46680
H	4.42390	6.45340	−1.53000
H	3.74060	5.32370	−2.69530
H	3.34000	5.17780	−0.98240
H	−1.92880	0.71520	3.82950
H	7.60850	3.22770	−2.25450
H	8.32530	4.69080	−2.92180
H	7.99430	4.59380	−1.19320

**Marilzafurollene C_140**			
C	2.94140	−1.78220	−1.37800
O	2.34790	−1.92830	−0.08860
C	3.39160	−0.32640	−1.60530
C	1.90990	−2.30660	−2.38670
C	1.02440	−2.43470	−0.21890
C	4.51400	0.08860	−0.63910
Br	1.84110	0.91120	−1.36410
C	1.04690	−3.21680	−1.53620
C	0.58700	−3.21370	1.03850
C	5.06560	1.46730	−0.94640
Cl	1.84320	−4.80970	−1.36530
C	0.55270	−2.34950	2.31360
C	−0.42100	−1.18790	2.21500
O	0.20730	−3.16460	3.41700
C	−0.04150	0.06950	2.24870
C	5.02650	2.50780	−0.09770
C	5.57040	3.89020	−0.40620
C	6.65520	4.28690	0.59810
O	4.54940	4.87450	−0.33140
C	0.36000	1.31840	2.26310
Br	0.50550	2.37040	3.93410
C	3.59020	4.80150	−1.37790
H	3.81170	−2.43980	−1.40580
H	3.73640	−0.21540	−2.63430
H	1.29880	−1.49270	−2.77730
H	2.36420	−2.81520	−3.23750
H	0.36290	−1.57880	−0.36080
H	4.14890	0.05160	0.38850
H	5.33880	−0.62210	−0.70020
H	0.05260	−3.37740	−1.95420
H	−0.39390	−3.65520	0.86010
H	1.27280	−4.04500	1.20420
H	5.51290	1.59550	−1.92150
H	1.55780	−1.96220	2.49760
H	−1.46620	−1.43630	2.10610
H	−0.62450	−3.58230	3.25080
H	4.57670	2.38570	0.87740
H	6.00810	3.89700	−1.40640
H	7.05590	5.27370	0.36590
H	6.26010	4.32000	1.61380
H	7.48450	3.57930	0.58250
H	0.64350	1.88390	1.38690
H	3.03460	3.86340	−1.35410
H	2.87110	5.61320	−1.26900
H	4.06260	4.90270	−2.35570

**Marilzafurollene C_141**			
C	2.90270	−1.01180	−1.01710
O	2.49400	−0.64660	0.30090
C	3.80930	0.07030	−1.63300
C	1.61500	−1.30120	−1.79800
C	1.07540	−0.73190	0.41590
C	5.13290	0.22110	−0.86390
Br	2.85960	1.82780	−1.64310
C	0.65260	−1.69730	−0.69810
C	0.66410	−1.09630	1.85560
C	6.08360	1.19920	−1.52680
Cl	0.92500	−3.40930	−0.25270
C	−0.85050	−1.29370	2.06390
C	−1.16140	−1.65360	3.50390
O	−1.53290	−0.10280	1.72890
C	−1.76690	−2.76300	3.86250
C	6.57000	2.30320	−0.93590
C	7.51000	3.29140	−1.60040
C	8.83940	3.37290	−0.84580
O	6.95680	4.59950	−1.61650
C	−2.35820	−3.87410	4.23340
Br	−1.35000	−5.52520	4.65200
C	5.83640	4.75020	−2.47840
H	3.46310	−1.94400	−0.92950
H	4.01830	−0.18770	−2.67200
H	1.24650	−0.40140	−2.29080
H	1.73970	−2.07010	−2.56090
H	0.68200	0.25770	0.17580
H	4.93000	0.53430	0.16140
H	5.63610	−0.74420	−0.79950
H	−0.39340	−1.58000	−0.98340
H	1.18840	−2.00340	2.15810
H	1.02460	−0.31350	2.52380
H	6.37140	0.97100	−2.54290
H	−1.20870	−2.09000	1.40790
H	−0.84950	−0.93730	4.25070
H	−2.46370	−0.24510	1.83020
H	6.28360	2.53590	0.07990
H	7.71350	2.97160	−2.62430
H	9.51930	4.07180	−1.33330
H	8.69140	3.71420	0.17900
H	9.33180	2.40090	−0.80920
H	−3.42540	−3.99900	4.34490
H	5.50650	5.78890	−2.46750
H	6.09040	4.49120	−3.50700
H	4.99440	4.13380	−2.16140

**Marilzafurollene C_142**			
C	3.10960	−0.47980	−1.39920
O	2.03980	0.11130	−0.66150
C	3.31240	0.23890	−2.74920
C	2.77800	−1.97300	−1.50610
C	1.11130	−0.89030	−0.25280
C	3.74660	1.70650	−2.59580
Br	1.60840	0.21210	−3.78730
C	1.90920	−2.19980	−0.28680
C	0.44920	−0.50620	1.08440
C	5.12930	1.85540	−1.98990
Cl	2.94540	−2.40300	1.15890
C	−0.51500	−1.56550	1.65310
C	−1.09060	−1.12250	2.98410
O	−1.57230	−1.78080	0.74150
C	−0.92850	−1.79200	4.10270
C	5.38050	2.46030	−0.81710
C	6.75950	2.61210	−0.20310
C	6.82830	1.91620	1.15850
O	7.08230	3.97890	0.00820
C	−0.77740	−2.47640	5.21190
Br	−2.05160	−3.88420	5.77090
C	7.33630	4.71060	−1.18330
H	4.00930	−0.37970	−0.79030
H	4.05130	−0.29880	−3.34460
H	2.19660	−2.18060	−2.40450
H	3.66380	−2.60810	−1.52980
H	0.34310	−0.93970	−1.02680
H	3.75980	2.18800	−3.57390
H	3.01340	2.24720	−1.99540
H	1.27110	−3.07960	−0.37610
H	1.22710	−0.29700	1.81940
H	−0.08370	0.43530	0.94700
H	5.94770	1.43820	−2.55910
H	0.01660	−2.50980	1.78840
H	−1.65520	−0.20100	2.97620
H	−2.14760	−2.44530	1.09520
H	4.56280	2.87740	−0.24630
H	7.50530	2.15880	−0.85920
H	7.82390	2.00980	1.59230
H	6.11760	2.35200	1.86110
H	6.60270	0.85320	1.06920
H	0.03420	−2.34610	5.91300
H	7.60840	5.73510	−0.92970
H	8.16310	4.27520	−1.74580
H	6.45820	4.75160	−1.82860

**Marilzafurollene C_143**			
C	3.24300	−1.63900	−0.96370
O	2.44510	−1.43800	0.20280
C	4.17690	−0.43590	−1.19470
C	2.25930	−1.96740	−2.09380
C	1.08210	−1.76590	−0.07270
C	3.45950	0.91800	−1.35360
Br	5.30140	−0.79180	−2.80250
C	1.11950	−2.62460	−1.34360
C	0.39680	−2.39350	1.15720
C	4.43040	2.08190	−1.37180
Cl	1.52940	−4.33180	−0.99810
C	0.27020	−1.42130	2.34310
C	−0.56620	−2.01280	3.46050
O	1.56360	−1.14140	2.83260
C	−1.68320	−1.47290	3.89200
C	4.46590	3.06200	−0.45200
C	5.43360	4.23470	−0.45040
C	4.70540	5.53890	−0.78490
O	6.48000	4.07880	−1.40130
C	−2.80370	−0.92950	4.30470
Br	−4.55900	−1.38400	3.51000
C	7.54270	3.24180	−0.96310
H	3.85480	−2.52290	−0.77550
H	4.86000	−0.37390	−0.34590
H	1.89700	−1.06250	−2.57960
H	2.69440	−2.60520	−2.86400
H	0.57720	−0.83040	−0.32050
H	2.87920	0.94530	−2.27510
H	2.75390	1.05850	−0.53380
H	0.17900	−2.60970	−1.89580
H	−0.60160	−2.72030	0.86250
H	0.92710	−3.29210	1.47530
H	5.13090	2.10150	−2.19520
H	−0.17930	−0.48660	2.00020
H	−0.19350	−2.92530	3.90260
H	2.15030	−1.12310	2.08070
H	3.76010	3.04430	0.36630
H	5.85740	4.32740	0.55140
H	5.39280	6.38450	−0.75800
H	4.26730	5.49890	−1.78260
H	3.90410	5.73940	−0.07340
H	−2.88040	−0.19960	5.09740
H	8.28540	3.15110	−1.75570
H	8.03880	3.66370	−0.08820
H	7.19620	2.23820	−0.71460

**Marilzafurollene C_144**			
C	2.64840	−1.25090	−1.95770
O	2.53820	−1.03500	−0.55060
C	1.82800	−0.20670	−2.74530
C	2.21620	−2.70570	−2.18840
C	2.01270	−2.19240	0.08540
C	2.44930	1.20440	−2.77420
Br	−0.02730	−0.09570	−2.01430
C	2.43680	−3.34270	−0.83030
C	2.46500	−2.26710	1.55620
C	2.71520	1.78700	−1.39870
Cl	4.16490	−3.76040	−0.63300
C	1.92290	−1.10390	2.40780
C	0.41050	−1.12380	2.54270
O	2.51570	−1.15040	3.69090
C	−0.36270	−0.17790	2.05910
C	3.89940	2.28080	−1.00070
C	4.18070	2.84180	0.37980
C	4.50580	4.33550	0.30410
O	5.30330	2.20110	0.96780
C	−1.11590	0.76760	1.54910
Br	−1.63520	2.38240	2.56950
C	5.04330	0.88050	1.42420
H	3.70350	−1.15980	−2.22100
H	1.73120	−0.54520	−3.77790
H	1.15630	−2.75430	−2.44120
H	2.77100	−3.19520	−2.98920
H	0.92460	−2.12500	0.04400
H	3.38490	1.17710	−3.33420
H	1.78740	1.88250	−3.31330
H	1.84800	−4.24820	−0.68040
H	2.15580	−3.22140	1.98320
H	3.55480	−2.25010	1.59430
H	1.88050	1.78560	−0.71190
H	2.22440	−0.16250	1.94370
H	−0.02210	−1.96730	3.05990
H	2.32790	−1.98500	4.09390
H	4.73500	2.28570	−1.68520
H	3.30380	2.71040	1.01730
H	4.69830	4.74020	1.29780
H	5.39110	4.51700	−0.30570
H	3.67740	4.89690	−0.12860
H	−1.51840	0.76040	0.54640
H	4.69030	0.23020	0.62280
H	5.95860	0.44580	1.82510
H	4.30270	0.88530	2.22410

**Marilzafurollene C_145**			
C	2.94400	−1.77660	−1.36700
O	2.34310	−1.92460	−0.08120
C	3.39480	−0.32050	−1.59150
C	1.91890	−2.30080	−2.38240
C	1.02200	−2.43510	−0.21960
C	4.51170	0.09620	−0.61970
Br	1.84280	0.91690	−1.35920
C	1.05330	−3.21450	−1.53840
C	0.57950	−3.21830	1.03340
C	5.07280	1.46940	−0.93450
Cl	1.85220	−4.80600	−1.36670
C	0.53780	−2.35860	2.31120
C	−0.43370	−1.19540	2.21050
O	0.18440	−3.17720	3.40950
C	−0.05240	0.06120	2.25210
C	5.03180	2.51840	−0.09640
C	5.58710	3.89420	−0.41370
C	6.66380	4.29510	0.59770
O	4.57180	4.88580	−0.36020
C	0.35260	1.30880	2.27650
Br	0.49000	2.34950	3.95510
C	3.62280	4.80730	−1.41560
H	3.81480	−2.43370	−1.39050
H	3.74500	−0.20990	−2.61870
H	1.30800	−1.48720	−2.77420
H	2.37860	−2.80650	−3.23190
H	0.35880	−1.58070	−0.36320
H	4.13890	0.06860	0.40540
H	5.33360	−0.61870	−0.66960
H	0.06160	−3.37640	−1.96210
H	−0.39990	−3.66050	0.84870
H	1.26540	−4.04940	1.20010
H	5.52980	1.58610	−1.90660
H	1.54220	−1.97320	2.50300
H	−1.47850	−1.44180	2.09340
H	−0.64780	−3.59180	3.23730
H	4.57240	2.40850	0.87570
H	6.03530	3.88780	−1.40930
H	7.07240	5.27730	0.35980
H	6.25840	4.34060	1.60880
H	7.48920	3.58280	0.59770
H	0.64350	1.87870	1.40560
H	3.06090	3.87310	−1.38700
H	2.90800	5.62490	−1.32320
H	4.10580	4.89440	−2.38960

**Marilzafurollene C_146**			
C	3.11440	−1.37840	−1.21590
O	2.43450	−1.53140	0.03000
C	3.30040	0.11370	−1.55030
C	2.30480	−2.17680	−2.24740
C	1.25290	−2.30290	−0.15630
C	4.23630	0.81700	−0.55300
Br	1.52670	1.03480	−1.54240
C	1.55670	−3.17100	−1.38220
C	0.84630	−3.04670	1.13120
C	4.53330	2.25100	−0.94700
Cl	2.63050	−4.54830	−0.99320
C	0.56560	−2.12530	2.33430
C	−0.54200	−1.10950	2.06630
O	0.26960	−2.95050	3.45000
C	−1.70820	−1.06680	2.67310
C	5.75720	2.72570	−1.23240
C	6.05140	4.16160	−1.62510
C	6.67540	4.22770	−3.02140
O	6.97230	4.76200	−0.72610
C	−2.86190	−1.03380	3.29660
Br	−3.13640	−0.00380	4.96480
C	6.43450	5.04160	0.55940
H	4.09180	−1.85160	−1.10860
H	3.71050	0.20860	−2.55700
H	1.58840	−1.53530	−2.76110
H	2.92790	−2.64970	−3.00680
H	0.45620	−1.61470	−0.44120
H	3.79170	0.81020	0.44300
H	5.17450	0.26590	−0.47670
H	0.65850	−3.56460	−1.85910
H	−0.03400	−3.65740	0.92780
H	1.63770	−3.74120	1.41330
H	3.68240	2.91640	−0.99200
H	1.47320	−1.56520	2.56700
H	−0.31180	−0.36740	1.31450
H	0.22900	−2.40670	4.22410
H	6.61290	2.06740	−1.18650
H	5.12360	4.73710	−1.63570
H	6.87320	5.26060	−3.30850
H	7.62110	3.68650	−3.05820
H	6.01090	3.79640	−3.77050
H	−3.75370	−1.55010	2.97280
H	7.19280	5.52840	1.17280
H	5.57810	5.71370	0.49180
H	6.12480	4.13240	1.07580

**Marilzafurollene C_147**			
C	3.35880	−1.72500	−0.66140
O	2.38960	−1.85490	0.37690
C	4.16250	−0.42220	−0.49230
C	2.58610	−1.88930	−1.97540
C	1.14270	−2.28940	−0.16470
C	3.31410	0.86360	−0.47990
Br	5.52370	−0.30950	−1.94530
C	1.46800	−2.82510	−1.56640
C	0.42020	−3.26620	0.78500
C	4.13610	2.09070	−0.13680
Cl	2.07450	−4.50880	−1.53420
C	−0.00240	−2.63740	2.12520
C	−1.03800	−1.54160	1.95400
O	−0.54800	−3.65850	2.93460
C	−0.85660	−0.30450	2.35690
C	4.26310	3.16690	−0.93080
C	5.09810	4.38850	−0.59590
C	4.22580	5.64450	−0.52740
O	6.09010	4.62070	−1.58540
C	−0.68170	0.92430	2.78200
Br	−1.19810	1.49950	4.60420
C	7.13140	3.65290	−1.60790
H	4.04230	−2.56970	−0.55970
H	4.71510	−0.49340	0.44570
H	2.16150	−0.94340	−2.30810
H	3.20260	−2.28030	−2.78550
H	0.53210	−1.39510	−0.29690
H	2.82590	1.01320	−1.44240
H	2.52180	0.77500	0.26410
H	0.61290	−2.79050	−2.24220
H	−0.45660	−3.67720	0.28310
H	1.07530	−4.11310	0.99180
H	4.64680	2.07100	0.81530
H	0.88450	−2.24270	2.62690
H	−1.96090	−1.82950	1.47130
H	−0.61700	−3.33560	3.82230
H	3.75590	3.19220	−1.88460
H	5.57910	4.24920	0.37430
H	4.82510	6.51860	−0.27240
H	3.74170	5.84210	−1.48410
H	3.44760	5.54310	0.22940
H	−0.25040	1.72490	2.19900
H	6.75800	2.66020	−1.86190
H	7.86890	3.93010	−2.36100
H	7.64150	3.59780	−0.64550

**Marilzafurollene C_148**			
C	3.35790	−1.72030	−0.66110
O	2.38990	−1.84890	0.37840
C	4.16290	−0.41790	−0.49380
C	2.58330	−1.88500	−1.97380
C	1.14340	−2.28830	−0.16070
C	3.31550	0.86860	−0.48280
Br	5.52340	−0.30810	−1.94760
C	1.46770	−2.82290	−1.56310
C	0.42680	−3.26790	0.79060
C	4.13840	2.09540	−0.14100
Cl	2.07710	−4.50550	−1.53260
C	0.00140	−2.63990	2.13030
C	−1.04240	−1.55200	1.95850
O	−0.53610	−3.66370	2.94190
C	−0.86990	−0.31280	2.35910
C	4.26740	3.16990	−0.93700
C	5.10270	4.39160	−0.60330
C	4.23160	5.64870	−0.54080
O	6.09770	4.62010	−1.59080
C	−0.70440	0.91820	2.78180
Br	−1.22370	1.49270	4.60330
C	7.13830	3.65150	−1.60730
H	4.04110	−2.56530	−0.55970
H	4.71580	−0.48840	0.44400
H	2.15650	−0.93960	−2.30520
H	3.19890	−2.27460	−2.78520
H	0.52920	−1.39630	−0.29160
H	2.82740	1.01750	−1.44540
H	2.52310	0.78130	0.26140
H	0.61130	−2.78970	−2.23750
H	−0.44800	−3.68420	0.28970
H	1.08670	−4.11090	0.99790
H	4.64780	2.07720	0.81190
H	0.88600	−2.23790	2.63030
H	−1.96360	−1.84750	1.47710
H	−0.60590	−3.34010	3.82920
H	3.76160	3.19370	−1.89160
H	5.58100	4.25470	0.36860
H	4.83100	6.52300	−0.28670
H	3.75030	5.84410	−1.49940
H	3.45110	5.55030	0.21410
H	−0.27990	1.72110	2.19690
H	6.76490	2.65830	−1.85940
H	7.87820	3.92580	−2.35910
H	7.64550	3.59900	−0.64320

**Marilzafurollene C_149**			
C	3.35780	−1.71920	−0.66060
O	2.38960	−1.84750	0.37880
C	4.16300	−0.41700	−0.49360
C	2.58310	−1.88400	−1.97330
C	1.14350	−2.28800	−0.16010
C	3.31590	0.86970	−0.48300
Br	5.52380	−0.30790	−1.94710
C	1.46790	−2.82240	−1.56260
C	0.42770	−3.26810	0.79120
C	4.13900	2.09650	−0.14170
Cl	2.07790	−4.50470	−1.53230
C	0.00170	−2.64040	2.13080
C	−1.04340	−1.55370	1.95890
O	−0.53460	−3.66470	2.94260
C	−0.87230	−0.31440	2.35970
C	4.26850	3.17060	−0.93810
C	5.10410	4.39220	−0.60490
C	4.23320	5.64960	−0.54310
O	6.09920	4.61990	−1.59230
C	−0.70840	0.91680	2.78250
Br	−1.22880	1.49040	4.60400
C	7.13960	3.65110	−1.60830
H	4.04080	−2.56430	−0.55900
H	4.71570	−0.48740	0.44430
H	2.15600	−0.93870	−2.30450
H	3.19880	−2.27330	−2.78480
H	0.52850	−1.39640	−0.29090
H	2.82780	1.01840	−1.44570
H	2.52350	0.78290	0.26120
H	0.61150	−2.78940	−2.23700
H	−0.44670	−3.68540	0.29030
H	1.08840	−4.11050	0.99860
H	4.64830	2.07860	0.81130
H	0.88580	−2.23740	2.63080
H	−1.96410	−1.85030	1.47740
H	−0.60490	−3.34110	3.82990
H	3.76280	3.19400	−1.89280
H	5.58220	4.25560	0.36710
H	4.83290	6.52380	−0.28930
H	3.75220	5.84460	−1.50190
H	3.45270	5.55170	0.21170
H	−0.28480	1.72020	2.19770
H	6.76590	2.65790	−1.85990
H	7.87960	3.92480	−2.36010
H	7.64670	3.59890	−0.64410

**Marilzafurollene C_150**			
C	2.90620	−2.04680	−1.37760
O	2.27310	−2.08540	−0.10030
C	3.25450	−0.59050	−1.75000
C	1.95890	−2.77370	−2.34630
C	0.95640	−2.60490	−0.22750
C	4.19890	0.04510	−0.71300
Br	1.58750	0.50600	−1.87010
C	1.06180	−3.56850	−1.41420
C	0.44620	−3.18170	1.10870
C	4.55410	1.48410	−1.03390
Cl	1.87610	−5.09940	−0.97390
C	0.52020	−2.17590	2.27400
C	−0.27780	−0.90960	2.02180
O	0.03470	−2.80580	3.44190
C	0.28270	0.26590	1.84550
C	4.25070	2.52470	−0.23590
C	4.56280	3.99530	−0.48790
C	5.23820	4.26910	−1.83890
O	5.42890	4.51820	0.51030
C	0.87160	1.41950	1.63530
Br	1.34540	2.66620	3.09700
C	4.82240	4.71780	1.78080
H	3.82670	−2.62610	−1.29030
H	3.72160	−0.56950	−2.73570
H	1.35290	−2.06540	−2.91070
H	2.48610	−3.39790	−3.06820
H	0.30570	−1.78160	−0.52560
H	3.74110	−0.00020	0.27630
H	5.12310	−0.53000	−0.64950
H	0.09390	−3.81210	−1.85360
H	−0.57850	−3.53220	0.98190
H	1.03530	−4.05690	1.38210
H	5.07910	1.64160	−1.96410
H	1.56920	−1.91960	2.44200
H	−1.35240	−1.01650	1.99250
H	0.28990	−2.28630	4.19140
H	3.72200	2.32140	0.68340
H	3.61810	4.54190	−0.47190
H	5.40640	5.33770	−1.97310
H	6.20780	3.77510	−1.90670
H	4.61950	3.92910	−2.66980
H	1.15560	1.79570	0.66370
H	3.95840	5.37970	1.70930
H	4.50660	3.77930	2.23630
H	5.54150	5.18260	2.45510

**Marilzafurollene C_151**			
C	2.50350	−0.53400	−0.91380
O	2.03290	−0.88030	0.38830
C	2.06620	0.89450	−1.29720
C	2.00760	−1.64130	−1.85100
C	1.39100	−2.15270	0.35780
C	2.63380	1.98730	−0.37430
Br	0.07700	1.02430	−1.23030
C	1.91080	−2.82920	−0.91710
C	1.62690	−2.91830	1.67410
C	4.14470	2.10780	−0.44230
Cl	3.53480	−3.54770	−0.69320
C	0.90640	−2.29420	2.88280
C	−0.59800	−2.47930	2.81810
O	1.38640	−2.91100	4.06010
C	−1.45330	−1.48420	2.75930
C	4.80150	3.17400	−0.92860
C	6.31240	3.29720	−0.99510
C	6.79810	4.49690	−0.17770
O	6.75320	3.49500	−2.33050
C	−2.32400	−0.50520	2.69480
Br	−2.97340	0.22630	0.97480
C	6.64610	2.34460	−3.15760
H	3.59200	−0.59100	−0.88490
H	2.36070	1.10510	−2.32630
H	1.01730	−1.41060	−2.24370
H	2.66970	−1.81200	−2.70020
H	0.32340	−1.97040	0.22290
H	2.18990	2.94690	−0.64180
H	2.34280	1.79220	0.65870
H	1.24370	−3.60960	−1.28490
H	1.31050	−3.95570	1.55750
H	2.69870	−2.94830	1.87160
H	4.70790	1.26690	−0.06330
H	1.14630	−1.22940	2.93740
H	−0.95050	−3.50030	2.82800
H	2.25880	−2.58720	4.23580
H	4.24520	4.01910	−1.30850
H	6.77320	2.39480	−0.58810
H	7.88470	4.57620	−0.21410
H	6.38460	5.43010	−0.56080
H	6.50680	4.40430	0.86890
H	−2.76470	−0.01380	3.54980
H	7.03140	2.57370	−4.15110
H	7.22900	1.51430	−2.75690
H	5.61050	2.02170	−3.27020

**Marilzafurollene C_152**			
C	2.54920	−0.54990	−0.96260
O	2.06670	−0.90480	0.33240
C	2.09440	0.87180	−1.35100
C	2.08350	−1.66370	−1.90770
C	1.45510	−2.19180	0.29530
C	2.62520	1.97250	−0.41550
Br	0.10230	0.96800	−1.32380
C	1.99740	−2.85530	−0.97760
C	1.69140	−2.95980	1.61070
C	4.13540	2.11180	−0.44780
Cl	3.63050	−3.55000	−0.74500
C	0.97610	−2.34200	2.82530
C	−0.53260	−2.47880	2.73860
O	1.42620	−3.01200	3.98550
C	−1.35860	−1.45740	2.74750
C	4.79110	3.17750	−0.93630
C	6.30170	3.31710	−0.96840
C	6.75320	4.54440	−0.17270
O	6.77360	3.48260	−2.29760
C	−2.20200	−0.45250	2.75830
Br	−2.81990	0.43500	1.10170
C	6.69810	2.30830	−3.09430
H	3.63800	−0.58980	−0.91880
H	2.40570	1.08960	−2.37370
H	1.09370	−1.45030	−2.31100
H	2.75790	−1.82050	−2.74980
H	0.38530	−2.03090	0.15120
H	2.17530	2.92610	−0.69420
H	2.31260	1.77370	0.61050
H	1.34590	−3.64490	−1.35340
H	1.37690	−3.99720	1.49090
H	2.76250	−2.98950	1.81350
H	4.69880	1.28390	−0.04100
H	1.25810	−1.28940	2.90670
H	−0.91430	−3.48710	2.66730
H	1.20500	−2.48770	4.74220
H	4.23440	4.00910	−1.34430
H	6.76210	2.43180	−0.52520
H	7.83950	4.63530	−0.18400
H	6.33930	5.46180	−0.59180
H	6.43650	4.47710	0.86840
H	−2.63550	−0.01960	3.64790
H	7.10660	2.51340	−4.08380
H	7.27810	1.49540	−2.65580
H	5.66880	1.97210	−3.22400

**Marilzafurollene C_153**			
C	2.42980	−1.14070	−1.38150
O	2.86000	−1.44620	−0.05650
C	3.31270	−0.03760	−1.99520
C	0.93120	−0.83750	−1.27530
C	1.74610	−1.48340	0.83440
C	3.34030	1.28340	−1.20340
Br	2.70440	0.31290	−3.86180
C	0.52330	−1.67070	−0.07900
C	1.98970	−2.52060	1.95350
C	4.37340	2.25380	−1.74180
Cl	0.29960	−3.37330	−0.58400
C	1.03420	−2.42220	3.16100
C	−0.37780	−2.86870	2.83580
O	1.54520	−3.24490	4.18890
C	−1.43160	−2.09890	2.98470
C	5.42060	2.71880	−1.04040
C	6.45340	3.69020	−1.58060
C	7.85280	3.07050	−1.55010
O	6.50290	4.87520	−0.79940
C	−2.46930	−1.30350	3.08930
Br	−3.03780	−0.12450	1.60500
C	5.37320	5.72350	−0.95420
H	2.55410	−2.05250	−1.96800
H	4.32990	−0.42650	−2.06560
H	0.75260	0.21460	−1.05850
H	0.38130	−1.08650	−2.18340
H	1.68690	−0.49080	1.28440
H	2.36760	1.77300	−1.23100
H	3.55900	1.07820	−0.15460
H	−0.40840	−1.32430	0.36730
H	1.99930	−3.53370	1.55140
H	3.00300	−2.35380	2.32120
H	4.23250	2.57820	−2.76340
H	1.02870	−1.39400	3.53010
H	−0.48370	−3.87600	2.45740
H	1.06690	−3.07210	4.98770
H	5.56580	2.40000	−0.01810
H	6.21280	3.94470	−2.61480
H	8.59210	3.76340	−1.95210
H	8.15220	2.81970	−0.53220
H	7.89280	2.15910	−2.14710
H	−3.09650	−1.21070	3.96430
H	5.50590	6.61990	−0.34860
H	5.25350	6.03670	−1.99210
H	4.45330	5.23690	−0.62820

**Marilzafurollene C_154**			
C	2.23150	−0.53690	−1.38160
O	1.96840	−0.73810	0.00670
C	1.58170	0.76880	−1.88290
C	1.75640	−1.81070	−2.08940
C	1.49570	−2.06340	0.23300
C	2.15460	2.02860	−1.21180
Br	−0.37880	0.72320	−1.51930
C	1.92210	−2.85780	−1.00830
C	1.99970	−2.59960	1.58680
C	3.60920	2.27770	−1.56220
Cl	3.63380	−3.37560	−0.93320
C	1.37610	−1.88320	2.79820
C	−0.08860	−2.23170	2.98490
O	2.08260	−2.26680	3.96040
C	−1.06160	−1.35160	2.92130
C	4.61360	2.27480	−0.67010
C	6.07110	2.52140	−1.01180
C	6.92890	1.30800	−0.64460
O	6.58790	3.63220	−0.29350
C	−2.04870	−0.49020	2.85530
Br	−3.00730	−0.10160	1.16870
C	6.08950	4.89100	−0.72560
H	3.31480	−0.47540	−1.49540
H	1.69890	0.84660	−2.96450
H	0.70350	−1.73770	−2.36180
H	2.31980	−2.03240	−2.99610
H	0.40530	−2.01770	0.23650
H	1.58270	2.90230	−1.52560
H	2.03680	1.95540	−0.12960
H	1.30900	−3.74420	−1.17430
H	1.80220	−3.67020	1.65480
H	3.08360	−2.48760	1.62310
H	3.82400	2.46620	−2.60440
H	1.48700	−0.80280	2.67660
H	−0.30640	−3.27050	3.18490
H	2.92550	−1.83510	3.95650
H	4.40050	2.08560	0.37250
H	6.17140	2.70190	−2.08390
H	7.97350	1.47790	−0.90570
H	6.88340	1.10100	0.42480
H	6.59470	0.41500	−1.17370
H	−2.42970	0.07710	3.69170
H	6.56060	5.68620	−0.14810
H	6.31460	5.06480	−1.77850
H	5.01160	4.97120	−0.58110

**Marilzafurollene C_155**			
C	2.50880	0.24380	−0.47400
O	2.18390	0.38970	0.90790
C	2.41800	1.59950	−1.20510
C	1.57990	−0.84870	−1.01600
C	1.14450	−0.51470	1.27150
C	3.41310	2.65270	−0.68610
Br	0.58110	2.35130	−0.99540
C	1.24820	−1.64060	0.23100
C	1.26040	−0.88800	2.76600
C	4.86410	2.28030	−0.92660
Cl	2.58490	−2.77620	0.58680
C	0.02710	−1.59650	3.36340
C	−0.13460	−3.01980	2.86610
O	0.17650	−1.62190	4.76770
C	−1.21040	−3.45290	2.24920
C	5.69080	2.93810	−1.75640
C	7.14290	2.56860	−1.99570
C	8.07050	3.72190	−1.60490
O	7.38160	2.28600	−3.36680
C	−2.26790	−3.86130	1.58960
Br	−2.40090	−3.70090	−0.37840
C	6.82480	1.05810	−3.81560
H	3.52950	−0.13650	−0.52290
H	2.57790	1.45170	−2.27410
H	0.66400	−0.42070	−1.42340
H	2.03940	−1.45120	−1.79980
H	0.20420	0.01670	1.11350
H	3.20820	3.60770	−1.17130
H	3.26420	2.81300	0.38250
H	0.33020	−2.21700	0.12170
H	2.16560	−1.46480	2.95540
H	1.40110	0.04600	3.31170
H	5.23460	1.42640	−0.37720
H	−0.86710	−1.01230	3.13400
H	0.70170	−3.68260	3.04000
H	−0.64440	−1.88520	5.15910
H	5.32780	3.79340	−2.30830
H	7.40490	1.69720	−1.39200
H	9.11430	3.45030	−1.76340
H	7.86580	4.61290	−2.19900
H	7.95160	3.98390	−0.55330
H	−3.14440	−4.30920	2.03530
H	5.73740	1.05260	−3.73360
H	7.07860	0.90550	−4.86450
H	7.22180	0.21420	−3.24990

**Marilzafurollene C_156**			
C	2.94120	−1.04060	−0.80770
O	2.71720	−1.14270	0.59750
C	4.22470	−0.23680	−1.08890
C	1.64100	−0.48700	−1.40530
C	1.36840	−0.79400	0.90550
C	4.23640	1.18800	−0.50200
Br	4.51620	−0.15080	−3.05990
C	0.60320	−0.96690	−0.41250
C	0.85950	−1.58760	2.12440
C	5.58760	1.85960	−0.65060
Cl	0.19770	−2.67860	−0.74310
C	−0.61180	−1.31900	2.49730
C	−1.02770	−2.13650	3.70490
O	−0.78000	0.05340	2.78880
C	−2.01310	−3.00540	3.69130
C	5.79640	3.00900	−1.31400
C	7.15020	3.67410	−1.47630
C	7.15110	5.07110	−0.85060
O	7.48680	3.82880	−2.84750
C	−2.98570	−3.88620	3.68550
Br	−2.69180	−5.78980	3.22970
C	7.76720	2.60900	−3.52170
H	3.07980	−2.05840	−1.17620
H	5.06330	−0.79820	−0.67480
H	1.64390	0.60170	−1.42370
H	1.45930	−0.83430	−2.42290
H	1.37390	0.26850	1.15530
H	3.47150	1.80610	−0.97090
H	3.99990	1.15070	0.56210
H	−0.31920	−0.38590	−0.44510
H	0.99420	−2.65390	1.93980
H	1.49860	−1.35550	2.97690
H	6.42280	1.35690	−0.18420
H	−1.25520	−1.57140	1.65160
H	−0.45790	−1.97370	4.60880
H	−1.69140	0.21220	2.99270
H	4.96500	3.51520	−1.78320
H	7.91550	3.07360	−0.98060
H	8.12900	5.54150	−0.95480
H	6.41820	5.71970	−1.33090
H	6.91560	5.02650	0.21300
H	−4.01730	−3.66960	3.92160
H	6.89610	1.95370	−3.55490
H	8.05930	2.82100	−4.55010
H	8.58880	2.07310	−3.04500

**Marilzafurollene C_157**			
C	3.05460	−1.70850	−1.10280
O	2.02060	−1.35810	−0.18230
C	3.89640	−0.47190	−1.46840
C	2.35880	−2.39170	−2.28730
C	0.75400	−1.77340	−0.68970
C	4.66690	0.08220	−0.25760
Br	2.70840	0.96080	−2.19440
C	1.08110	−2.91260	−1.66180
C	−0.22470	−2.10760	0.45320
C	5.58310	1.23260	−0.62720
Cl	1.41520	−4.45090	−0.81180
C	−0.59790	−0.88460	1.31030
C	−1.73140	−1.20780	2.27790
O	0.56560	−0.43550	1.98220
C	−1.64730	−1.19200	3.59050
C	5.50420	2.46510	−0.09850
C	6.41030	3.62220	−0.47500
C	7.20170	4.11390	0.73950
O	5.66170	4.72670	−0.96200
C	−1.54330	−1.17920	4.89830
Br	−1.04830	−2.78580	5.94570
C	5.05350	4.50840	−2.22830
H	3.68870	−2.44600	−0.60790
H	4.60060	−0.73760	−2.25820
H	2.11040	−1.66840	−3.06420
H	2.96430	−3.17390	−2.74590
H	0.35130	−0.94140	−1.27060
H	3.96450	0.39960	0.51470
H	5.27890	−0.70480	0.18420
H	0.28980	−3.09260	−2.39030
H	−1.13190	−2.52510	0.01460
H	0.18700	−2.88730	1.09540
H	6.34000	1.02130	−1.36890
H	−0.94230	−0.07860	0.66020
H	−2.67220	−1.46230	1.81200
H	1.30000	−0.56870	1.38700
H	4.74810	2.68160	0.64270
H	7.11860	3.29900	−1.24040
H	7.86340	4.93520	0.46410
H	6.53660	4.47300	1.52520
H	7.81720	3.31700	1.15760
H	−1.71570	−0.31340	5.52020
H	4.31250	3.70910	−2.19030
H	4.54210	5.41650	−2.54720
H	5.79650	4.25950	−2.98680

**Marilzafurollene C_158**			
C	2.60680	0.17430	−0.78120
O	1.50150	−0.24720	0.01630
C	2.10970	1.00660	−1.97840
C	3.34170	−1.11720	−1.15070
C	1.65370	−1.61290	0.40530
C	3.22160	1.47070	−2.93970
Br	1.14720	2.61020	−1.28690
C	3.10650	−1.97900	0.07180
C	1.20770	−1.83060	1.86480
C	4.31640	2.26250	−2.24950
Cl	4.24800	−1.50640	1.36660
C	−0.29350	−1.57340	2.08520
C	−0.73170	−1.99750	3.47350
O	−0.54090	−0.19350	1.92540
C	−1.60150	−2.95580	3.69980
C	5.61060	1.90370	−2.22060
C	6.70260	2.68280	−1.51260
C	7.76760	3.15500	−2.50550
O	7.36000	1.87790	−0.54470
C	−2.45760	−3.92300	3.93120
Br	−4.40820	−3.61240	4.06450
C	6.57000	1.57370	0.59730
H	3.24810	0.78370	−0.14280
H	1.37350	0.42840	−2.53830
H	2.87980	−1.58170	−2.02290
H	4.39830	−0.96030	−1.37030
H	1.00740	−2.19640	−0.25270
H	3.66180	0.60680	−3.43910
H	2.79070	2.09090	−3.72630
H	3.24210	−3.04360	−0.12220
H	1.43320	−2.86200	2.13980
H	1.78810	−1.20260	2.54190
H	4.00420	3.16960	−1.75080
H	−0.87080	−2.11780	1.33450
H	−0.28690	−1.45790	4.29690
H	0.00480	0.11490	1.20530
H	5.92930	0.99970	−2.71920
H	6.27170	3.55990	−1.02550
H	8.54450	3.72620	−1.99720
H	8.24740	2.31130	−3.00190
H	7.33350	3.79540	−3.27370
H	−2.19260	−4.96110	4.06890
H	7.15920	0.98810	1.30290
H	6.24360	2.48120	1.10660
H	5.69040	0.98560	0.33540

**Marilzafurollene C_159**			
C	2.58300	−0.97410	−1.51980
O	1.53760	−0.83320	−0.55520
C	2.38960	0.04660	−2.66010
C	2.55240	−2.44430	−1.96460
C	1.17380	−2.10780	−0.03620
C	2.43850	1.50790	−2.18230
Br	0.62420	−0.25780	−3.53750
C	1.30850	−2.98270	−1.28400
C	2.10720	−2.47030	1.14480
C	3.79080	1.89910	−1.61720
Cl	1.40750	−4.73400	−0.94840
C	1.98520	−1.50620	2.33910
C	0.64030	−1.62120	3.03300
O	3.00710	−1.81900	3.26400
C	−0.21770	−0.63040	3.12600
C	4.00420	2.22990	−0.33270
C	5.35110	2.61690	0.24830
C	5.77170	1.63370	1.34360
O	5.30540	3.90750	0.83940
C	−1.09320	0.34410	3.20310
Br	−2.48760	0.62640	1.82670
C	5.20090	4.97400	−0.09440
H	3.53470	−0.78930	−1.01920
H	3.15070	−0.11010	−3.42570
H	2.51900	−2.57550	−3.04660
H	3.44090	−2.95860	−1.59630
H	0.13730	−2.09060	0.30260
H	2.21630	2.17550	−3.01520
H	1.66030	1.67690	−1.43610
H	0.44210	−2.82030	−1.92750
H	1.89480	−3.48410	1.48610
H	3.14260	−2.48440	0.80350
H	4.61840	1.89970	−2.31240
H	2.14600	−0.48260	1.99070
H	0.40670	−2.58660	3.46000
H	3.06260	−1.12130	3.90180
H	3.17600	2.22710	0.36220
H	6.10860	2.60200	−0.53810
H	6.74700	1.90090	1.75130
H	5.05710	1.62990	2.16730
H	5.84040	0.61710	0.95500
H	−1.14260	1.07020	4.00180
H	6.04250	4.97560	−0.78860
H	4.27480	4.92100	−0.66840
H	5.20630	5.92500	0.43840

**Marilzafurollene C_160**			
C	2.58030	−0.96570	−1.51170
O	1.53240	−0.83210	−0.54890
C	2.38340	0.05670	−2.64960
C	2.55800	−2.43470	−1.96040
C	1.17260	−2.10960	−0.03500
C	2.43590	1.51700	−2.16940
Br	0.61450	−0.24570	−3.52070
C	1.31550	−2.98110	−1.28440
C	2.10300	−2.47030	1.14830
C	3.79460	1.90780	−1.62000
Cl	1.42330	−4.73250	−0.95280
C	1.96950	−1.51120	2.34460
C	0.62410	−1.63880	3.03380
O	2.99080	−1.81890	3.27180
C	−0.24010	−0.65450	3.13160
C	4.02350	2.23980	−0.33860
C	5.37780	2.62530	0.22500
C	5.80870	1.64450	1.31900
O	5.34220	3.91860	0.81180
C	−1.12250	0.31270	3.21570
Br	−2.51720	0.59680	1.84020
C	5.23440	4.98260	−0.12490
H	3.53000	−0.77750	−1.00880
H	3.14130	−0.09940	−3.41840
H	2.52750	−2.56330	−3.04260
H	3.44790	−2.94550	−1.59130
H	0.13500	−2.09790	0.29990
H	2.20400	2.18580	−2.99860
H	1.66620	1.68410	−1.41440
H	0.45020	−2.82230	−1.92980
H	1.89590	−3.48630	1.48540
H	3.13960	−2.47670	0.81140
H	4.61390	1.90740	−2.32490
H	2.12290	−0.48550	2.00030
H	0.39590	−2.60830	3.45340
H	3.03840	−1.12320	3.91210
H	3.20400	2.23900	0.36630
H	6.12610	2.60520	−0.57030
H	6.78930	1.91060	1.71370
H	5.10410	1.64580	2.15160
H	5.87020	0.62580	0.93350
H	−1.17770	1.03130	4.02030
H	5.24690	5.93590	0.40450
H	6.07070	4.97900	−0.82550
H	4.30420	4.93100	−0.69150

**Marilzafurollene C_161**			
C	3.22040	−1.04930	0.52350
O	1.81040	−0.85860	0.65360
C	3.90200	0.32290	0.36850
C	3.40980	−2.02800	−0.64720
C	1.10940	−1.90890	−0.00610
C	3.44790	1.12270	−0.86750
Br	5.87720	0.05500	0.30420
C	2.01150	−2.15650	−1.21720
C	0.93410	−3.10870	0.95620
C	4.03960	2.51810	−0.90130
Cl	1.73730	−3.72160	−2.03100
C	0.13540	−2.77150	2.22850
C	−1.29610	−2.37010	1.92610
O	0.12840	−3.91390	3.05940
C	−1.79870	−1.19760	2.23990
C	4.80630	2.99430	−1.89630
C	5.41140	4.38530	−1.92510
C	4.90340	5.17720	−3.13250
O	6.82690	4.32900	−2.02660
C	−2.28540	−0.02410	2.56750
Br	−3.15630	0.31390	4.31250
C	7.47780	3.84080	−0.86080
H	3.58110	−1.52190	1.43860
H	3.69770	0.90430	1.26880
H	4.13620	−1.69940	−1.39040
H	3.75310	−2.98950	−0.26390
H	0.13140	−1.55420	−0.33260
H	3.70910	0.59280	−1.78350
H	2.36200	1.22310	−0.86290
H	1.84460	−1.37110	−1.95560
H	0.43590	−3.92540	0.43260
H	1.91100	−3.49590	1.24600
H	3.81640	3.15100	−0.05420
H	0.64420	−1.96740	2.76550
H	−1.90350	−3.10970	1.42430
H	−0.20980	−3.66700	3.90890
H	5.03400	2.36590	−2.74530
H	5.13410	4.92480	−1.01730
H	5.32760	6.18130	−3.14330
H	5.17890	4.69070	−4.06850
H	3.81780	5.27550	−3.11080
H	−2.26150	0.85250	1.93670
H	8.55740	3.87350	−1.00660
H	7.24000	4.45210	0.01070
H	7.20530	2.80690	−0.64630

**Marilzafurollene C_162**			
C	2.62480	−0.58090	−1.34810
O	2.23500	−0.81780	0.00470
C	1.73830	0.51120	−1.98320
C	2.57450	−1.94410	−2.05200
C	1.90390	−2.19090	0.18020
C	1.91530	1.88950	−1.32420
Br	−0.18360	−0.00230	−1.81630
C	2.71160	−2.91820	−0.89900
C	2.12640	−2.64710	1.63580
C	3.28950	2.48510	−1.56220
Cl	4.43710	−3.08740	−0.45790
C	1.24860	−1.89410	2.65220
C	−0.23460	−2.10490	2.41020
O	1.57480	−2.35180	3.94860
C	−1.05320	−1.13370	2.07510
C	4.20180	2.68580	−0.59420
C	5.59510	3.28170	−0.76320
C	5.85380	3.88250	−2.15210
O	6.60730	2.30620	−0.55420
C	−1.84900	−0.15240	1.72180
Br	−2.82330	0.95300	3.04390
C	6.74950	1.86880	0.79050
H	3.66450	−0.25070	−1.32890
H	1.95440	0.58760	−3.04970
H	1.61070	−2.09490	−2.53870
H	3.34930	−2.06190	−2.81000
H	0.84910	−2.30380	−0.07450
H	1.17840	2.58600	−1.72490
H	1.71790	1.81380	−0.25370
H	2.32090	−3.91040	−1.12700
H	1.93940	−3.71890	1.71100
H	3.17220	−2.50040	1.90650
H	3.51340	2.74670	−2.58570
H	1.48640	−0.82890	2.59780
H	−0.59940	−3.11560	2.52290
H	1.19960	−1.75490	4.58080
H	3.94160	2.40580	0.41650
H	5.70450	4.08220	−0.02940
H	6.84230	4.34040	−2.19240
H	5.81770	3.11980	−2.93030
H	5.12470	4.65620	−2.39390
H	−2.04700	0.14310	0.70130
H	5.86630	1.33350	1.13980
H	7.59480	1.18430	0.86010
H	6.94250	2.70580	1.46270

**Marilzafurollene C_163**			
C	3.01310	−1.63920	−2.15430
O	3.17270	−1.49170	−0.74400
C	4.27540	−1.17770	−2.91920
C	1.69240	−0.94100	−2.51140
C	2.09460	−0.72130	−0.22160
C	4.69090	0.30470	−2.78250
Br	4.01630	−1.57220	−4.85440
C	0.94000	−0.96700	−1.19430
C	1.86330	−1.02470	1.26850
C	5.00830	0.71180	−1.35610
Cl	0.18650	−2.57070	−0.94890
C	0.72000	−0.21620	1.90890
C	0.60240	−0.49640	3.39510
O	0.91870	1.16970	1.70980
C	−0.47990	−0.98360	3.95810
C	4.54330	1.82540	−0.76010
C	4.77780	2.24620	0.68670
C	5.59080	1.23600	1.50830
O	3.54090	2.41350	1.37270
C	−1.56400	−1.45570	4.52660
Br	−1.88800	−3.39550	4.75200
C	2.88520	3.64470	1.08690
H	2.88500	−2.70890	−2.32730
H	5.10820	−1.80300	−2.59450
H	1.86450	0.09330	−2.80650
H	1.15780	−1.43240	−3.32470
H	2.37570	0.32650	−0.31420
H	5.58960	0.47200	−3.37680
H	3.92750	0.96180	−3.19840
H	0.16040	−0.20680	−1.12960
H	1.67070	−2.09030	1.39810
H	2.79240	−0.82790	1.80420
H	5.64040	0.02580	−0.81210
H	−0.22140	−0.48350	1.42390
H	1.47250	−0.27440	3.99540
H	1.84240	1.38430	1.81050
H	3.92890	2.49550	−1.34250
H	5.32870	3.18850	0.67850
H	5.71920	1.58800	2.53190
H	5.09270	0.26710	1.55420
H	6.58620	1.08950	1.08840
H	−2.36810	−0.85040	4.91890
H	2.58550	3.71260	0.04110
H	1.98310	3.73010	1.69270
H	3.52070	4.49860	1.32500

**Marilzafurollene C_164**			
C	2.87490	−0.93430	−1.18050
O	1.50310	−0.63830	−0.90580
C	3.34550	−0.12710	−2.40750
C	2.95530	−2.45940	−1.34620
C	0.82960	−1.81120	−0.45610
C	3.27000	1.39840	−2.21610
Br	2.21850	−0.60240	−3.98340
C	1.50160	−2.88960	−1.30880
C	1.00710	−1.95460	1.07490
C	4.19980	1.91150	−1.13290
Cl	1.29030	−4.55100	−0.69000
C	0.37290	−0.80200	1.87440
C	0.40090	−1.07650	3.36500
O	1.09330	0.38210	1.61060
C	−0.68060	−1.16580	4.10500
C	5.23530	2.74220	−1.34530
C	6.17890	3.26760	−0.27480
C	7.58000	2.67660	−0.45040
O	5.74620	2.93960	1.03990
C	−1.76980	−1.26270	4.82980
Br	−2.71230	−2.98170	5.10510
C	4.75460	3.81590	1.56060
H	3.46690	−0.66030	−0.30640
H	4.37060	−0.40460	−2.65840
H	3.45450	−2.77570	−2.26250
H	3.50180	−2.89160	−0.50720
H	−0.23290	−1.74390	−0.69280
H	3.51580	1.89060	−3.15780
H	2.24960	1.69590	−1.97180
H	1.08620	−2.85930	−2.31740
H	0.53140	−2.88400	1.39070
H	2.06020	−2.05670	1.33890
H	3.99400	1.57300	−0.12700
H	−0.65880	−0.65880	1.54500
H	1.37830	−1.19800	3.80860
H	1.24780	0.40590	0.66980
H	5.44290	3.08080	−2.35050
H	6.24970	4.35170	−0.38190
H	7.56450	1.59130	−0.34650
H	7.99100	2.91500	−1.43170
H	8.26410	3.07190	0.30070
H	−2.24950	−0.43780	5.33610
H	3.86440	3.84610	0.93160
H	4.45160	3.47600	2.55090
H	5.14230	4.83040	1.66040

**Marilzafurollene C_165**			
C	3.03290	−2.10940	−1.17300
O	2.30560	−1.34750	−0.21340
C	4.51570	−2.27660	−0.77530
C	2.74720	−1.44490	−2.52260
C	1.38300	−0.47160	−0.86510
C	5.39580	−1.01010	−0.79450
Br	5.34100	−3.60930	−2.00420
C	1.33070	−0.94710	−2.32480
C	0.02420	−0.41010	−0.13560
C	4.93200	0.06780	0.16530
Cl	0.17780	−2.29610	−2.56200
C	−0.01190	0.40020	1.17690
C	0.98370	−0.08060	2.21600
O	0.17440	1.77720	0.91660
C	0.64300	−0.46770	3.42370
C	4.59940	1.31680	−0.20710
C	4.12380	2.44040	0.70370
C	4.29380	2.15420	2.20230
O	2.74660	2.71700	0.48280
C	0.29100	−0.84250	4.63060
Br	−0.18260	−2.71440	5.06700
C	2.48920	3.47090	−0.69690
H	2.57290	−3.09900	−1.17520
H	4.55180	−2.72150	0.22000
H	3.40400	−0.59300	−2.69140
H	2.85440	−2.12710	−3.36640
H	1.83420	0.52050	−0.86510
H	6.41260	−1.28270	−0.51030
H	5.46910	−0.60850	−1.80490
H	1.05790	−0.14980	−3.01690
H	−0.72000	0.01300	−0.81150
H	−0.31110	−1.42710	0.07190
H	4.87260	−0.22750	1.20220
H	−1.01880	0.30530	1.58920
H	2.01950	−0.10300	1.91400
H	1.10680	1.96670	0.82720
H	4.66950	1.57500	−1.25350
H	4.72090	3.32530	0.47530
H	3.98310	3.01600	2.79320
H	3.69360	1.30630	2.52740
H	5.33540	1.94770	2.44950
H	0.22610	−0.18590	5.48580
H	2.79010	2.93320	−1.59620
H	1.42100	3.67310	−0.77700
H	3.00550	4.43150	−0.67410

**Marilzafurollene C_166**			
C	2.26620	−0.55530	−1.43630
O	1.99450	−0.76670	−0.05140
C	1.60970	0.74780	−1.93530
C	1.80690	−1.82900	−2.15420
C	1.54980	−2.10310	0.16720
C	2.16220	2.00760	−1.24760
Br	−0.35470	0.68050	−1.59640
C	1.98620	−2.88190	−1.08100
C	2.06120	−2.64540	1.51640
C	3.61860	2.27290	−1.57800
Cl	3.70410	−3.37950	−1.01360
C	1.45140	−1.93630	2.73870
C	−0.02540	−2.24120	2.90600
O	2.13930	−2.38000	3.89090
C	−0.96410	−1.32230	2.90470
C	4.61190	2.27150	−0.67360
C	6.07120	2.53370	−0.99510
C	6.93580	1.32680	−0.62230
O	6.56810	3.64630	−0.26550
C	−1.91820	−0.42180	2.90680
Br	−2.86400	0.12870	1.25870
C	6.06260	4.90200	−0.69830
H	3.34990	−0.48420	−1.54130
H	1.73930	0.83490	−3.01480
H	0.75310	−1.76750	−2.42590
H	2.37300	−2.03660	−3.06250
H	0.45880	−2.07650	0.17190
H	1.58600	2.87830	−1.56150
H	2.03200	1.92470	−0.16750
H	1.38420	−3.77460	−1.25360
H	1.86450	−3.71620	1.57960
H	3.14540	−2.53360	1.55220
H	3.84450	2.47220	−2.61580
H	1.60850	−0.85940	2.63970
H	−0.28350	−3.28290	3.03160
H	1.96980	−1.77220	4.59660
H	4.38770	2.07120	0.36470
H	6.18360	2.72000	−2.06510
H	7.98200	1.50770	−0.86910
H	6.87830	1.11450	0.44560
H	6.61680	0.43300	−1.15940
H	−2.27720	0.09570	3.78440
H	4.98220	4.97100	−0.56680
H	6.51870	5.69930	−0.11140
H	6.29910	5.08280	−1.74760

**Marilzafurollene C_167**			
C	2.79850	−1.48590	−1.11350
O	2.01650	−1.10790	0.02010
C	3.67920	−0.31190	−1.58010
C	1.80700	−2.00830	−2.16150
C	0.63550	−1.35620	−0.23540
C	4.73450	0.07310	−0.52910
Br	2.53320	1.27880	−1.96400
C	0.63190	−2.44710	−1.31200
C	−0.12830	−1.67220	1.06580
C	5.67730	1.15490	−1.01950
Cl	0.95910	−4.06900	−0.63230
C	−0.18100	−0.47460	2.04270
C	−1.06320	−0.67010	3.26900
O	1.13400	−0.19450	2.47010
C	−1.80210	−1.72640	3.52960
C	5.84220	2.34590	−0.42030
C	6.77450	3.43600	−0.91500
C	7.84420	3.75650	0.13190
O	6.06820	4.64060	−1.17440
C	−2.54850	−2.77060	3.80230
Br	−1.87590	−4.32250	4.83080
C	5.19630	4.57780	−2.29540
H	3.43500	−2.31590	−0.80230
H	4.17570	−0.58400	−2.51270
H	1.48880	−1.20860	−2.83070
H	2.21740	−2.80960	−2.77650
H	0.22170	−0.44780	−0.67740
H	4.24190	0.39750	0.38890
H	5.33340	−0.79990	−0.26790
H	−0.30490	−2.49510	−1.86840
H	−1.14410	−1.96100	0.79260
H	0.31210	−2.53530	1.56690
H	6.24060	0.92860	−1.91330
H	−0.55570	0.40170	1.51110
H	−1.05310	0.15390	3.96750
H	1.71030	−0.36310	1.72740
H	5.28020	2.57750	0.47320
H	7.27350	3.10480	−1.82790
H	8.52230	4.52900	−0.23100
H	7.39520	4.11750	1.05760
H	8.44060	2.87510	0.36850
H	−3.57900	−2.88800	3.50030
H	4.73340	5.55220	−2.45090
H	5.73880	4.31680	−3.20490
H	4.39540	3.85270	−2.14670

**Marilzafurollene C_168**			
C	3.25220	−0.94860	−0.78480
O	2.47820	−0.75030	0.39840
C	3.65580	0.40060	−1.40840
C	2.41790	−1.85940	−1.69420
C	1.25730	−1.47980	0.31110
C	4.59500	1.20280	−0.49230
Br	2.02290	1.48870	−1.78470
C	1.54260	−2.59550	−0.70160
C	0.77720	−1.90940	1.71110
C	5.10650	2.46970	−1.15040
Cl	2.46440	−3.94810	0.02320
C	−0.50680	−2.76170	1.71110
C	−0.85800	−3.23850	3.11600
O	−1.56640	−2.03310	1.11250
C	−1.98480	−2.99770	3.75050
C	6.40050	2.73060	−1.40020
C	6.90910	4.00010	−2.05760
C	7.64470	3.68160	−3.36170
O	7.82630	4.68310	−1.21550
C	−3.10950	−2.73680	4.37370
Br	−3.34490	−1.13220	5.50870
C	7.22950	5.30170	−0.08370
H	4.15060	−1.49380	−0.49080
H	4.15140	0.22070	−2.36370
H	1.78770	−1.27240	−2.36250
H	3.02540	−2.52320	−2.30980
H	0.51960	−0.80410	−0.12580
H	4.07660	1.46940	0.42970
H	5.44420	0.58300	−0.20150
H	0.63470	−3.00100	−1.15020
H	1.57720	−2.46180	2.20520
H	0.62550	−1.01600	2.31760
H	4.35560	3.19700	−1.42580
H	−0.34460	−3.65860	1.11160
H	−0.09280	−3.82740	3.60190
H	−1.76660	−1.28410	1.65640
H	7.15680	2.00960	−1.12490
H	6.06780	4.65650	−2.28900
H	7.99700	4.59530	−3.84060
H	8.51190	3.04590	−3.18170
H	6.99100	3.16800	−4.06720
H	−3.99590	−3.35280	4.33170
H	6.76450	4.57130	0.57940
H	7.99360	5.82820	0.48810
H	6.47550	6.03060	−0.38340

**Marilzafurollene C_169**			
C	2.95870	−1.89330	−1.36570
O	2.31660	−1.92040	−0.09190
C	3.88350	−0.66690	−1.47990
C	1.83510	−2.00120	−2.40420
C	0.92440	−2.18860	−0.24880
C	3.18540	0.68960	−1.26830
Br	4.77210	−0.69350	−3.26520
C	0.78420	−2.79130	−1.65290
C	0.38190	−3.04230	0.91430
C	4.17130	1.84010	−1.21580
Cl	1.19600	−4.53260	−1.69280
C	0.41590	−2.32670	2.27730
C	−0.51260	−1.12740	2.33120
O	0.02660	−3.24870	3.27440
C	−0.09990	0.10210	2.54160
C	4.36470	2.63140	−0.14770
C	5.35030	3.78400	−0.10030
C	6.39880	3.56120	0.99260
O	4.69310	5.00860	0.19180
C	0.30580	1.32890	2.76940
Br	0.43840	2.08380	4.59440
C	3.89660	5.51130	−0.87250
H	3.56890	−2.79590	−1.42810
H	4.67470	−0.77460	−0.73590
H	1.43570	−1.02080	−2.65980
H	2.15610	−2.48000	−3.32980
H	0.41620	−1.22320	−0.25950
H	2.47350	0.88920	−2.06840
H	2.61500	0.66740	−0.33870
H	−0.21750	−2.67350	−2.06780
H	−0.64050	−3.34990	0.69090
H	0.96710	−3.95970	0.98250
H	4.74530	2.01380	−2.11550
H	1.43880	−2.00800	2.49250
H	−1.56310	−1.33360	2.18720
H	0.75610	−3.82460	3.45620
H	3.79510	2.46440	0.75530
H	5.86620	3.86610	−1.05910
H	7.11460	4.38310	1.01590
H	5.93590	3.49670	1.97770
H	6.95650	2.64010	0.82150
H	0.60090	2.03370	2.00590
H	3.08070	4.83280	−1.12380
H	3.45620	6.46390	−0.57840
H	4.49600	5.68300	−1.76750

**Marilzafurollene C_170**			
C	3.24650	−1.50700	−1.69610
O	2.30240	−0.86950	−0.83520
C	3.54170	−0.62380	−2.92580
C	2.64420	−2.88040	−2.01440
C	1.21620	−1.74450	−0.54800
C	4.45600	0.58410	−2.63390
Br	1.82740	0.02240	−3.71820
C	1.77640	−3.14670	−0.79970
C	0.66520	−1.44800	0.85850
C	3.98050	1.44980	−1.48120
Cl	2.79210	−3.71780	0.55740
C	−0.09010	−0.09920	0.93530
C	−0.65690	0.24810	2.30490
O	0.77990	0.93620	0.53320
C	−0.50810	−0.46300	3.40090
C	4.72680	1.75140	−0.40530
C	4.25540	2.58340	0.77200
C	4.99270	3.92390	0.81870
O	4.52780	1.91990	1.99810
C	−0.32560	−1.16190	4.49610
Br	1.22330	−0.86660	5.69260
C	3.64490	0.84370	2.28500
H	4.16490	−1.66020	−1.12670
H	4.02620	−1.23120	−3.69180
H	2.01640	−2.82520	−2.90470
H	3.39780	−3.64990	−2.18380
H	0.43680	−1.55590	−1.28880
H	5.46240	0.22690	−2.41190
H	4.54050	1.20530	−3.52600
H	1.00030	−3.89110	−0.98080
H	−0.01420	−2.25280	1.14240
H	1.48150	−1.46370	1.58210
H	2.96990	1.82720	−1.55320
H	−0.92470	−0.12430	0.23270
H	−1.21920	1.16980	2.34180
H	1.42440	0.54890	−0.05740
H	5.73920	1.38130	−0.33560
H	3.18450	2.77890	0.68300
H	6.06770	3.78030	0.92900
H	4.82080	4.49900	−0.09140
H	4.64940	4.52450	1.66110
H	−0.97990	−1.94700	4.84590
H	3.95560	0.35130	3.20620
H	2.62730	1.20500	2.43380
H	3.63940	0.09440	1.49240

**Marilzafurollene C_171**			
C	3.43060	−1.38480	−1.91770
O	2.53460	−1.11580	−0.83790
C	3.25770	−0.34380	−3.04390
C	3.14780	−2.83390	−2.33430
C	1.72920	−2.25840	−0.56480
C	3.87200	1.03710	−2.73620
Br	1.32010	−0.12240	−3.46960
C	2.56490	−3.43600	−1.07070
C	1.31340	−2.28060	0.91780
C	3.41130	1.63590	−1.41990
Cl	3.88220	−3.85760	0.06360
C	0.31470	−1.16440	1.27730
C	−0.15080	−1.28210	2.72450
O	0.92510	0.08650	1.01430
C	−0.03860	−0.34770	3.64370
C	4.24170	2.06020	−0.45270
C	3.80030	2.62710	0.88290
C	4.14420	4.11520	0.98190
O	4.47260	1.97540	1.95100
C	0.08430	0.59590	4.54700
Br	−1.32260	1.95060	4.87430
C	3.98860	0.66880	2.22900
H	4.44740	−1.33340	−1.52490
H	3.73080	−0.72210	−3.95150
H	2.40000	−2.86420	−3.12790
H	4.03470	−3.35740	−2.69230
H	0.83450	−2.18820	−1.18650
H	4.95920	0.95160	−2.72120
H	3.62990	1.73330	−3.53970
H	1.97330	−4.33340	−1.25540
H	0.85830	−3.24750	1.13650
H	2.19400	−2.20700	1.55750
H	2.34210	1.70710	−1.27570
H	−0.56580	−1.25020	0.63850
H	−0.60950	−2.22510	2.98370
H	1.52000	−0.04180	0.27750
H	5.31010	1.99760	−0.59950
H	2.72010	2.51190	0.99620
H	5.21850	4.27960	0.89590
H	3.65150	4.68560	0.19410
H	3.82080	4.52360	1.93940
H	0.94280	0.72420	5.18940
H	4.02710	0.01900	1.35360
H	4.59780	0.21270	3.00890
H	2.96210	0.70640	2.59340

**Marilzafurollene C_172**			
C	3.33350	−1.27500	−0.70670
O	2.34720	−1.32250	0.32500
C	3.77560	0.17690	−0.96990
C	2.72300	−1.99600	−1.91580
C	1.19070	−2.01730	−0.13800
C	4.49570	0.78720	0.25080
Br	2.17240	1.28600	−1.41240
C	1.70380	−2.91290	−1.27130
C	0.46310	−2.72710	1.02060
C	5.16070	2.14070	0.03500
Cl	2.52320	−4.37920	−0.65530
C	−0.12210	−1.74920	2.05470
C	−0.98230	−2.46730	3.07610
O	0.94780	−1.11130	2.71710
C	−2.26940	−2.24900	3.22370
C	5.24460	2.83060	−1.11740
C	5.92780	4.17740	−1.26190
C	4.93960	5.23910	−1.75090
O	6.98300	4.11800	−2.21070
C	−3.55810	−2.04550	3.36400
Br	−4.29490	−0.67990	4.59350
C	8.12300	3.39220	−1.77130
H	4.18850	−1.85810	−0.36030
H	4.43800	0.18780	−1.83640
H	2.21030	−1.29270	−2.57220
H	3.46280	−2.52730	−2.51520
H	0.52170	−1.27330	−0.57500
H	3.79070	0.87830	1.07810
H	5.27470	0.10360	0.58980
H	0.91620	−3.22720	−1.95700
H	−0.34760	−3.32410	0.60050
H	1.13040	−3.43100	1.51950
H	5.60840	2.56770	0.92120
H	−0.71460	−0.98790	1.54200
H	−0.47520	−3.18760	3.70140
H	1.61530	−0.93250	2.05870
H	4.81060	2.44150	−2.02590
H	6.32380	4.49550	−0.29540
H	5.42610	6.21050	−1.84040
H	4.53260	4.97980	−2.72860
H	4.10450	5.34770	−1.05820
H	−4.32910	−2.58790	2.83660
H	8.89350	3.42430	−2.54160
H	8.54160	3.82630	−0.86240
H	7.88810	2.34430	−1.58160

**Marilzafurollene C_173**			
C	2.55260	−0.94820	−1.67510
O	2.51940	−0.09370	−0.53050
C	2.41810	−0.15020	−2.98930
C	1.44250	−1.98000	−1.44960
C	1.51830	−0.52090	0.38830
C	3.67540	0.64930	−3.38610
Br	0.84970	1.08140	−2.88790
C	1.32090	−2.00720	0.05900
C	1.92180	−0.13740	1.82860
C	4.10840	1.66360	−2.34510
Cl	2.61730	−3.03440	0.74270
C	0.80720	−0.27550	2.88540
C	0.48780	−1.71910	3.22170
O	1.24260	0.37730	4.05990
C	−0.70980	−2.24410	3.09810
C	5.33930	1.72930	−1.81110
C	5.76020	2.73030	−0.75200
C	6.87980	3.63590	−1.27080
O	6.25390	2.07770	0.40930
C	−1.89750	−2.77400	2.92750
Br	−2.44110	−3.59840	1.21260
C	5.26390	1.36910	1.14370
H	3.51110	−1.46960	−1.66420
H	2.20550	−0.85000	−3.79880
H	0.50230	−1.63410	−1.88060
H	1.66840	−2.95490	−1.88230
H	0.60600	0.01660	0.12300
H	4.49930	−0.03870	−3.57980
H	3.48900	1.17840	−4.32100
H	0.36080	−2.40160	0.39040
H	2.81680	−0.67630	2.13940
H	2.21740	0.91170	1.79850
H	3.35150	2.36500	−2.02360
H	−0.08950	0.23810	2.53090
H	1.31520	−2.31980	3.57320
H	0.51240	0.45250	4.65810
H	6.10070	1.03160	−2.12760
H	4.90750	3.35600	−0.48080
H	7.16990	4.36480	−0.51400
H	7.76640	3.05770	−1.53190
H	6.56250	4.18540	−2.15740
H	−2.67450	−2.81260	3.67720
H	4.44380	2.02450	1.43910
H	4.85580	0.53510	0.57220
H	5.70620	0.95900	2.05130

**Marilzafurollene C_174**			
C	2.66560	−0.81150	−1.07860
O	2.18560	−1.26010	0.18860
C	2.06300	0.56480	−1.43770
C	2.36020	−1.93610	−2.08130
C	1.45980	−2.47120	0.02470
C	2.35960	1.66240	−0.40040
Br	0.07930	0.42220	−1.60930
C	2.12770	−3.13600	−1.18490
C	1.41400	−3.30160	1.32280
C	3.83740	1.97380	−0.26150
Cl	3.70510	−3.87310	−0.77220
C	0.83900	−2.54870	2.53710
C	−0.55110	−1.98910	2.29600
O	0.79360	−3.45040	3.62460
C	−0.87340	−0.73970	2.54520
C	4.40700	3.13650	−0.61930
C	5.88530	3.44830	−0.47990
C	6.10350	4.66490	0.42310
O	6.46690	3.75160	−1.73960
C	−1.18450	0.49950	2.84530
Br	−0.98270	2.00180	1.57330
C	6.61220	2.63050	−2.60060
H	3.74900	−0.72600	−0.98970
H	2.43730	0.88230	−2.41200
H	1.45160	−1.73010	−2.64600
H	3.16210	−2.09040	−2.80380
H	0.44100	−2.20410	−0.26010
H	1.82750	2.57280	−0.67900
H	1.97090	1.36880	0.57560
H	1.50510	−3.90250	−1.64750
H	0.83180	−4.20570	1.14090
H	2.41740	−3.63680	1.58390
H	4.45060	1.18990	0.16000
H	1.52130	−1.73550	2.79670
H	−1.28270	−2.67570	1.89560
H	0.67000	−2.95550	4.42200
H	3.79870	3.92410	−1.04070
H	6.40080	2.59420	−0.03630
H	7.16650	4.87890	0.53470
H	5.62770	5.55460	0.00990
H	5.69260	4.49510	1.41860
H	−1.57280	0.82000	3.80090
H	7.09210	2.94380	−3.52770
H	7.23660	1.86090	−2.14510
H	5.64850	2.19030	−2.85880

**Marilzafurollene C_175**			
C	2.90820	−1.27690	−1.02520
O	2.11980	−1.26720	0.16570
C	3.28120	0.15830	−1.44060
C	2.10000	−2.06210	−2.06650
C	0.90610	−1.98550	−0.04800
C	4.20820	0.83590	−0.41730
Br	1.62180	1.25190	−1.64630
C	1.21960	−2.94240	−1.20350
C	0.40000	−2.62970	1.25710
C	4.68770	2.19790	−0.88070
Cl	2.14930	−4.37230	−0.66340
C	−0.00350	−1.59540	2.32250
C	−0.66750	−2.25450	3.51520
O	1.15870	−0.92230	2.75440
C	−1.89660	−1.99100	3.89620
C	5.97280	2.51730	−1.10750
C	6.45190	3.87800	−1.56760
C	7.15650	3.77360	−2.92690
O	7.35190	4.39900	−0.60090
C	−3.12960	−1.72690	4.25840
Br	−4.68150	−2.73340	3.55300
C	7.42380	5.81740	−0.57350
H	3.81710	−1.83920	−0.80480
H	3.77600	0.13470	−2.41290
H	1.47280	−1.39460	−2.65780
H	2.72690	−2.62520	−2.75850
H	0.16410	−1.26610	−0.40000
H	3.69160	0.95130	0.53640
H	5.07320	0.19970	−0.22570
H	0.32630	−3.29400	−1.72090
H	−0.46700	−3.24790	1.01960
H	1.15050	−3.30580	1.66860
H	3.92050	2.94500	−1.02880
H	−0.68210	−0.86310	1.87920
H	−0.06860	−2.97170	4.05720
H	1.70400	−0.78640	1.98270
H	6.74430	1.77590	−0.95740
H	5.57890	4.52790	−1.65800
H	7.46020	4.75120	−3.29940
H	8.04930	3.15140	−2.86020
H	6.49700	3.33210	−3.67460
H	−3.41520	−0.95530	4.95840
H	7.76300	6.22500	−1.52590
H	6.45570	6.25700	−0.32990
H	8.13290	6.13050	0.19290

**Marilzafurollene C_176**			
C	2.43200	−0.86950	−1.09130
O	2.71640	−1.03760	0.29730
C	3.05580	0.43360	−1.62550
C	0.90790	−0.96120	−1.22690
C	1.50970	−1.22120	1.03220
C	4.59270	0.40340	−1.57010
Br	2.39750	1.98870	−0.55780
C	0.52020	−1.77000	−0.00700
C	1.78570	−2.06590	2.29540
C	5.22260	1.63170	−2.19810
Cl	0.77990	−3.50960	−0.33860
C	0.63400	−2.10780	3.32100
C	−0.55060	−2.93750	2.86190
O	1.12520	−2.64160	4.53360
C	−1.77100	−2.46100	2.76790
C	6.03870	2.48040	−1.55140
C	6.65990	3.71770	−2.17200
C	8.18790	3.62940	−2.15410
O	6.29980	4.89040	−1.45600
C	−2.97700	−1.96260	2.63640
Br	−3.58480	−1.11830	0.95270
C	4.93020	5.25190	−1.57650
H	2.87220	−1.72100	−1.61270
H	2.73100	0.59000	−2.65500
H	0.44960	0.02590	−1.16380
H	0.58760	−1.41500	−2.16510
H	1.17630	−0.22750	1.33720
H	4.92280	0.30270	−0.53500
H	4.96550	−0.47310	−2.10110
H	−0.52400	−1.62610	0.26950
H	2.10300	−3.07260	2.02390
H	2.65020	−1.61510	2.78520
H	4.98090	1.81510	−3.23510
H	0.30730	−1.08680	3.53170
H	−0.34850	−3.96640	2.60070
H	1.53040	−3.47980	4.36410
H	6.28220	2.30160	−0.51380
H	6.33180	3.81140	−3.20910
H	8.56760	3.56000	−1.13440
H	8.53860	2.75580	−2.70410
H	8.63240	4.51210	−2.61410
H	−3.73510	−1.96060	3.40620
H	4.27130	4.49430	−1.15080
H	4.75150	6.18310	−1.03920
H	4.65260	5.40940	−2.61940

**Marilzafurollene C_177**			
C	3.20310	−1.76510	−1.17110
O	2.17030	−1.52490	−0.21460
C	3.93900	−0.45670	−1.51440
C	2.52990	−2.46100	−2.36130
C	0.92520	−2.01670	−0.70670
C	4.69710	0.11570	−0.30430
Br	2.62740	0.90360	−2.16270
C	1.31090	−3.09610	−1.72440
C	0.00220	−2.46030	0.44560
C	5.51400	1.34370	−0.65660
Cl	1.77980	−4.63170	−0.93520
C	−0.43690	−1.29750	1.35400
C	−1.52580	−1.73290	2.32910
O	0.71040	−0.80240	2.02170
C	−1.46920	−1.62990	3.63940
C	5.35630	2.54930	−0.08510
C	6.16250	3.78340	−0.44390
C	6.94640	4.29500	0.76720
O	5.32040	4.84270	−0.87530
C	−1.39550	−1.52310	4.94490
Br	−2.05350	0.06710	5.92540
C	4.69650	4.61900	−2.13300
H	3.90410	−2.46750	−0.71720
H	4.64020	−0.64220	−2.32940
H	2.20850	−1.73410	−3.10760
H	3.18010	−3.18040	−2.85990
H	0.44780	−1.19860	−1.24940
H	3.99340	0.35400	0.49470
H	5.37810	−0.63600	0.09610
H	0.51670	−3.31130	−2.44020
H	−0.88290	−2.92700	0.01130
H	0.48440	−3.23010	1.04950
H	6.26480	1.21450	−1.42280
H	−0.84810	−0.49440	0.74000
H	−2.40820	−2.15620	1.87170
H	1.43270	−0.83770	1.39870
H	4.60590	2.68400	0.68090
H	6.87260	3.53950	−1.23650
H	7.53700	5.17260	0.50400
H	6.27720	4.57720	1.58030
H	7.63100	3.53430	1.14340
H	−0.98180	−2.27470	5.60070
H	4.11030	5.49540	−2.40880
H	5.43560	4.45120	−2.91730
H	4.01880	3.76520	−2.10380

**Marilzafurollene C_178**			
C	2.88880	−1.58040	−1.11370
O	2.22380	−1.85600	0.11850
C	3.00140	−0.06030	−1.33820
C	2.11390	−2.34180	−2.19850
C	1.07680	−2.66420	−0.12160
C	3.90080	0.61270	−0.28770
Br	1.18570	0.77270	−1.27410
C	1.41490	−3.42820	−1.40560
C	0.70880	−3.51240	1.11200
C	4.12660	2.08500	−0.57240
Cl	2.55060	−4.77920	−1.11270
C	0.36740	−2.66840	2.35380
C	−0.82900	−1.76140	2.13310
O	0.09120	−3.54520	3.42680
C	−0.74780	−0.45020	2.14620
C	5.32450	2.63680	−0.82870
C	5.55100	4.10730	−1.11040
C	6.14650	4.30550	−2.51080
O	6.43930	4.63040	−0.13410
C	−0.63760	0.85700	2.14290
Br	−0.85250	1.94560	3.78200
C	6.29740	6.02450	0.09860
H	3.88850	−2.01190	−1.04160
H	3.40870	0.12700	−2.33310
H	1.36720	−1.70000	−2.66650
H	2.75620	−2.73100	−2.98900
H	0.24980	−1.99120	−0.35320
H	3.45700	0.51020	0.70350
H	4.86520	0.10460	−0.24840
H	0.53380	−3.82770	−1.90870
H	−0.13190	−4.16170	0.86550
H	1.54010	−4.17190	1.36170
H	3.24550	2.71090	−0.55980
H	1.24230	−2.07030	2.62030
H	−1.77550	−2.25100	1.95530
H	0.10060	−3.04640	4.23190
H	6.20940	2.01690	−0.83870
H	4.58490	4.61290	−1.04970
H	6.26580	5.36110	−2.75220
H	7.12590	3.83340	−2.59190
H	5.50140	3.86920	−3.27380
H	−0.42340	1.45470	1.26850
H	5.29760	6.26400	0.46310
H	7.01230	6.34060	0.85820
H	6.49020	6.60780	−0.80190

**Marilzafurollene C_179**			
C	2.68020	−0.59020	−1.24370
O	1.91610	−0.65820	−0.03900
C	2.52960	0.79430	−1.90710
C	2.22260	−1.77340	−2.10660
C	1.05010	−1.79130	−0.07470
C	3.09290	1.95340	−1.06560
Br	0.59630	1.16680	−2.23270
C	1.70670	−2.75210	−1.07220
C	0.79240	−2.34410	1.34040
C	4.59810	1.88780	−0.88520
Cl	3.08200	−3.63990	−0.35020
C	0.02090	−1.36240	2.23970
C	−0.37680	−2.00560	3.55340
O	0.84930	−0.25100	2.50160
C	−1.62350	−2.14590	3.94230
C	5.46390	2.77650	−1.40620
C	6.98240	2.76100	−1.26250
C	7.53380	1.50720	−0.56870
O	7.44280	3.87290	−0.50670
C	−2.87380	−2.29050	4.31270
Br	−3.93750	−3.89580	3.85360
C	7.37410	5.12290	−1.17930
H	3.72230	−0.76280	−0.97310
H	3.01900	0.78990	−2.88240
H	1.40540	−1.48350	−2.76730
H	3.01960	−2.18290	−2.72780
H	0.10310	−1.45830	−0.50390
H	2.83490	2.89940	−1.54310
H	2.62080	1.96830	−0.08240
H	1.00920	−3.48290	−1.48330
H	0.21510	−3.26520	1.24770
H	1.73100	−2.62180	1.82160
H	4.96000	1.06350	−0.28880
H	−0.87070	−1.01240	1.71440
H	0.43340	−2.35360	4.17740
H	1.32050	−0.05890	1.69400
H	5.06830	3.59040	−1.99580
H	7.41120	2.79970	−2.26550
H	8.62310	1.53510	−0.53740
H	7.18110	1.43300	0.46030
H	7.24520	0.59860	−1.09760
H	−3.43390	−1.56900	4.88940
H	6.34640	5.41210	−1.39890
H	7.80440	5.89820	−0.54540
H	7.94040	5.10360	−2.11140

**Marilzafurollene C_180**			
C	2.71520	−0.89290	−0.95250
O	1.50130	−0.90850	−0.19860
C	2.62310	0.16150	−2.07510
C	2.92900	−2.33260	−1.44400
C	1.16620	−2.24200	0.17170
C	2.40330	1.59770	−1.56760
Br	1.11150	−0.29190	−3.29560
C	1.63540	−3.02270	−1.05770
C	1.86900	−2.59940	1.50380
C	3.55590	2.12100	−0.73160
Cl	1.84350	−4.77490	−0.78330
C	1.41570	−1.72870	2.68940
C	−0.03240	−1.97910	3.06620
O	2.23860	−2.03030	3.79790
C	−0.96350	−1.05350	3.02230
C	4.37050	3.12310	−1.10140
C	5.52330	3.64750	−0.26580
C	5.33160	5.13130	0.05810
O	6.75660	3.52630	−0.95930
C	−1.90020	−0.13770	2.95270
Br	−2.97130	0.15840	1.31530
C	7.23130	2.19220	−1.07870
H	3.53360	−0.64190	−0.27680
H	3.53130	0.13260	−2.67910
H	3.13370	−2.40690	−2.51230
H	3.77060	−2.78090	−0.91470
H	0.08580	−2.33570	0.28680
H	2.25440	2.26000	−2.42130
H	1.48840	1.65020	−0.97580
H	0.90350	−2.90140	−1.85770
H	1.68770	−3.64680	1.74680
H	2.94850	−2.50580	1.38570
H	3.70830	1.63940	0.22380
H	1.56230	−0.67600	2.43600
H	−0.27610	−2.98440	3.37850
H	2.08970	−1.38160	4.47130
H	4.22350	3.60970	−2.05520
H	5.57730	3.09470	0.67430
H	6.15290	5.50340	0.67070
H	5.29650	5.73320	−0.85030
H	4.40510	5.29790	0.60820
H	−2.17570	0.52840	3.75730
H	8.19200	2.19280	−1.59360
H	7.37800	1.73600	−0.09890
H	6.54690	1.56870	−1.65510

**Marilzafurollene C_181**			
C	2.83860	−1.68860	−2.09200
O	2.59630	−1.62580	−0.68560
C	2.89710	−0.28450	−2.73070
C	1.73890	−2.59140	−2.66950
C	1.40760	−2.33960	−0.36550
C	4.15840	0.53150	−2.38360
Br	1.26480	0.76820	−2.26230
C	1.29960	−3.39870	−1.46600
C	1.41940	−2.86300	1.08410
C	4.23470	0.96430	−0.93100
Cl	2.43370	−4.75940	−1.21380
C	1.47720	−1.75210	2.14820
C	0.30730	−0.79080	2.05530
O	1.47250	−2.36370	3.42200
C	0.45240	0.50990	1.94440
C	4.29910	2.24640	−0.52770
C	4.37250	2.74700	0.90970
C	4.49140	1.63110	1.95670
O	3.21400	3.49340	1.25330
C	0.61060	1.80980	1.87000
Br	0.65110	2.95870	3.48240
C	3.13470	4.78460	0.66680
H	3.79820	−2.18900	−2.23050
H	2.87970	−0.40790	−3.81460
H	0.89890	−1.99500	−3.02720
H	2.08350	−3.20830	−3.49980
H	0.57170	−1.65000	−0.49290
H	5.05130	−0.04920	−2.61710
H	4.19450	1.41700	−3.01910
H	0.29390	−3.80790	−1.56710
H	0.53290	−3.47690	1.24700
H	2.27540	−3.52260	1.22760
H	4.23230	0.16450	−0.20580
H	2.41660	−1.20600	2.03470
H	−0.67850	−1.23210	2.07490
H	1.67090	−1.70400	4.07190
H	4.30130	3.01820	−1.28270
H	5.25560	3.38230	0.99810
H	4.56590	2.05270	2.95920
H	3.61830	0.97920	1.94250
H	5.37870	1.01990	1.79010
H	0.73310	2.36790	0.95240
H	3.05050	4.73500	−0.41870
H	2.24760	5.29510	1.04180
H	4.00270	5.39220	0.92560

**Marilzafurollene C_182**			
C	3.18460	−1.56530	−1.90680
O	2.55990	−1.09320	−0.71240
C	3.28790	−0.45960	−2.97710
C	2.37380	−2.79080	−2.34860
C	1.46430	−1.93760	−0.36340
C	4.31700	0.64470	−2.66160
Br	1.49510	0.35380	−3.31470
C	1.76670	−3.27430	−1.04890
C	1.25410	−1.98600	1.16250
C	3.94280	1.53480	−1.49040
Cl	2.97370	−4.24190	−0.15010
C	0.87730	−0.62060	1.76090
C	0.48820	−0.73780	3.22100
O	1.99530	0.23020	1.64860
C	−0.69230	−0.39630	3.68450
C	4.66440	1.65070	−0.36050
C	4.34710	2.54400	0.83520
C	3.06750	3.37770	0.67420
O	5.39550	3.47520	1.07100
C	−1.87940	−0.06090	4.13100
Br	−3.38470	−1.34440	4.21110
C	6.55570	2.92260	1.67720
H	4.18690	−1.90010	−1.63490
H	3.59150	−0.92710	−3.91510
H	1.57500	−2.50030	−3.03180
H	2.97910	−3.54670	−2.84960
H	0.57390	−1.51320	−0.83140
H	5.29530	0.19610	−2.48460
H	4.43070	1.28830	−3.53440
H	0.87820	−3.89010	−1.19360
H	0.45620	−2.69890	1.37560
H	2.14430	−2.36900	1.66280
H	3.03230	2.10190	−1.61380
H	0.05270	−0.18700	1.19040
H	1.24650	−1.12430	3.88610
H	2.38860	0.05850	0.79450
H	5.56200	1.05860	−0.26740
H	4.22300	1.90420	1.71070
H	2.88670	3.97580	1.56720
H	3.14140	4.06230	−0.17080
H	2.19270	2.74390	0.52750
H	−2.14860	0.92160	4.49030
H	7.27830	3.71670	1.86470
H	6.31860	2.45670	2.63450
H	7.03880	2.18350	1.03830

Table S11. Calculated Energies (Hartrees) for diastereoisomer 4*S**,6*R**,7*R**,9*S**,10*S**,14*R** of **3**.

Entry ID	Gas Phase Energy
143	−1335,69167
144	−1335,686622
145	−1335,688013
146	−1335,690842
147	−1335,691849
148	−1335,691951
149	−1335,689106
150	−1335,686557
151	−1335,688437
152	−1335,690532
153	−1335,689517
154	−1335,689371
155	−1335,68772
156	−1335,686384
157	−1335,679403
158	−1335,688972
159	−1335,690139
160	−1335,690955
161	−1335,68773
162	−1335,688286
163	−1335,687563
164	−1335,68634
165	−1335,685297
166	−1335,686988
167	−1335,690233
168	−1335,690051
169	−1335,688278
170	−1335,689638
171	−1335,689451
172	−1335,689709
173	−1335,689253
174	−1335,683545
175	−1335,686713
176	−1335,684604
177	−1335,684408
178	−1335,68635
179	−1335,682776
180	−1335,681469
181	−1335,687188
182	−1335,687735
183	−1335,686522
184	−1335,686052
185	−1335,689022

186	−1335,689024
187	−1335,687393
188	−1335,687453
189	−1335,686534
190	−1335,689686
191	−1335,688534
192	−1335,688622
193	−1335,684539
194	−1335,68199
195	−1335,68405
196	−1335,688334
197	−1335,687957
198	−1335,687629
199	−1335,686071
200	−1335,687074
201	−1335,689343
202	−1335,6847
203	−1335,684909
204	−1335,69019

Table S12. Coordinates (Angstroms) of calculated geometries for diastereoisomer 4*S**,6*R**,7*R**,9*S**,10*S**,14*R** of **3**.

**Marilzafurollene C_143**			
C	3.44890	−0.59010	1.12620
O	4.77200	−1.10180	1.29210
C	2.41640	−1.54130	1.75900
C	3.26930	−0.34860	−0.37830
C	5.42410	−1.18490	0.02610
C	2.56760	−1.62720	3.28710
Br	2.61810	−3.36900	0.97760
C	4.69560	−0.16270	−0.85350
C	6.94780	−1.00400	0.16830
C	1.47550	−2.45740	3.93310
Cl	5.22360	1.51380	−0.51990
C	7.61700	−2.13620	0.96710
C	9.12840	−2.02680	0.92900
O	7.18160	−2.05520	2.30660
C	9.90890	−2.95750	0.42900
C	0.58240	−1.98570	4.81890
C	−0.51060	−2.81790	5.46310
C	−0.36250	−2.82760	6.98660
O	−1.79640	−2.29080	5.17050
C	10.67560	−3.89050	−0.08380
Br	11.19310	−3.90700	−1.99470
C	−2.21350	−2.48390	3.82570
H	3.41170	0.37570	1.63290
H	1.41070	−1.19420	1.51620

H	2.83860	−1.22380	−0.86530
H	2.62610	0.50320	−0.60100
H	5.22050	−2.17920	−0.37620
H	3.53560	−2.05990	3.54290
H	2.55400	−0.62410	3.71540
H	4.82020	−0.35180	−1.92040
H	7.38330	−0.97280	−0.83150
H	7.17970	−0.04340	0.62980
H	1.43670	−3.49820	3.64330
H	7.30550	−3.10110	0.56040
H	9.55090	−1.12370	1.34460
H	6.25670	−1.82040	2.28400
H	0.61490	−0.94640	5.11290
H	−0.44780	−3.84760	5.10520
H	0.60250	−3.23740	7.28590
H	−0.44330	−1.82130	7.39850
H	−1.13950	−3.43770	7.44740
H	11.09070	−4.72080	0.46860
H	−2.24520	−3.54380	3.57030
H	−3.21690	−2.07910	3.69440
H	−1.55580	−1.97480	3.12030
**Marilzafurollene C_144**			
C	2.84550	−1.20500	0.46430
O	4.10400	−1.87570	0.46670
C	1.70900	−2.16210	0.05370
C	3.04610	0.03750	−0.41390
C	5.14110	−0.91100	0.33860
C	1.60790	−3.39930	0.96760
Br	2.00540	−2.79480	−1.81420
C	4.53400	0.00520	−0.72960
C	5.42890	−0.26470	1.71810
C	1.48280	−3.04390	2.43880
Cl	5.26130	1.63460	−0.79540
C	5.93110	−1.28100	2.76180
C	7.37320	−1.68370	2.52120
O	5.81830	−0.71990	4.05300
C	7.74660	−2.90560	2.21670
C	2.50270	−3.14310	3.30790
C	2.45390	−2.76830	4.77520
C	2.97660	−3.91100	5.64870
O	3.27770	−1.63490	5.02370
C	8.12490	−4.11770	1.88730
Br	8.25410	−4.71160	0.00380
C	2.63770	−0.39630	4.74150
H	2.65350	−0.85410	1.47680
H	0.76030	−1.62390	0.05910
H	2.44810	0.02670	−1.32520

H	2.77980	0.93460	0.14590
H	6.04730	−1.38620	−0.03900
H	0.75880	−4.01840	0.67730
H	2.49770	−4.01520	0.82750
H	4.67810	−0.45680	−1.70770
H	6.16930	0.52860	1.61050
H	4.53310	0.22970	2.09160
H	0.52210	−2.67730	2.77150
H	5.29750	−2.16860	2.74000
H	8.10640	−0.89600	2.61470
H	5.02460	−1.06280	4.45910
H	3.45460	−3.50890	2.95120
H	1.42260	−2.55940	5.06720
H	2.38360	−4.81540	5.51030
H	4.01380	−4.14940	5.41130
H	2.93160	−3.64350	6.70460
H	8.39650	−4.89240	2.58940
H	1.76430	−0.25120	5.37850
H	3.32650	0.42680	4.93190
H	2.32180	−0.33230	3.70100
**Marilzafurollene C_145**			
C	2.80090	0.26420	0.61520
O	3.85070	−0.55830	1.12760
C	1.41420	−0.35570	0.88640
C	3.12120	0.46930	−0.87150
C	4.78220	−0.87140	0.09460
C	0.95700	−0.30360	2.35910
Br	1.36710	−2.24430	0.23990
C	4.62100	0.26240	−0.92230
C	6.19530	−1.08470	0.67080
C	1.83030	−1.10490	3.30700
Cl	5.45030	1.75950	−0.40140
C	6.29030	−2.31490	1.59030
C	7.71840	−2.57600	2.02570
O	5.49400	−2.07880	2.73020
C	8.36970	−3.68220	1.74730
C	1.41820	−2.18050	3.99760
C	2.29800	−2.99240	4.92920
C	1.76380	−2.94480	6.36280
O	2.32660	−4.35730	4.53880
C	9.01920	−4.78270	1.45020
Br	10.08620	−4.97050	−0.20670
C	3.08830	−4.60470	3.36450
H	2.87080	1.23100	1.11630
H	0.67540	0.18830	0.29580
H	2.63210	−0.29240	−1.47980
H	2.80740	1.44400	−1.24640

H	4.44520	−1.79730	−0.37570
H	0.93900	0.73030	2.70520
H	−0.06900	−0.66710	2.42720
H	4.98500	0.00020	−1.91640
H	6.89100	−1.21050	−0.16020
H	6.52600	−0.19700	1.21140
H	2.85120	−0.76920	3.41550
H	5.89490	−3.18970	1.06890
H	8.19380	−1.79000	2.59410
H	4.72060	−1.60690	2.42850
H	0.39880	−2.52370	3.89650
H	3.31240	−2.58760	4.92490
H	1.72730	−1.92080	6.73520
H	0.75850	−3.36210	6.42410
H	2.40340	−3.51940	7.03280
H	9.03710	−5.67300	2.06170
H	3.04890	−5.66640	3.12190
H	2.70660	−4.05340	2.50440
H	4.13490	−4.33710	3.51320
**Marilzafurollene C_146**			
C	3.29370	−0.23440	0.61960
O	4.54440	−0.79580	1.01970
C	2.12380	−1.00800	1.25550
C	3.30260	−0.22030	−0.91470
C	5.32170	−1.12260	−0.13070
C	2.10250	−0.86900	2.78740
Br	2.26030	−2.94380	0.78130
C	4.78320	−0.20620	−1.23490
C	6.83030	−1.02370	0.16740
C	0.88780	−1.52410	3.41610
Cl	5.41010	1.46330	−1.09250
C	7.30860	−2.05690	1.20260
C	8.81870	−2.05540	1.33440
O	6.73550	−1.73650	2.45120
C	9.56870	−3.10250	1.07680
C	0.94380	−2.51700	4.31930
C	−0.26790	−3.17830	4.94880
C	−0.29980	−4.67420	4.62640
O	−0.24620	−3.05460	6.36350
C	10.30650	−4.15250	0.80450
Br	11.02950	−4.50090	−1.00520
C	−0.49500	−1.73870	6.83890
H	3.27870	0.79900	0.97000
H	1.18330	−0.64040	0.84260
H	2.85620	−1.13050	−1.31580
H	2.76190	0.62670	−1.33790
H	5.07990	−2.15230	−0.40120

H	3.01470	−1.29280	3.21020
H	2.09370	0.18570	3.06400
H	5.00850	−0.56900	−2.23840
H	7.37440	−1.18090	−0.76510
H	7.09070	−0.01970	0.50460
H	−0.07490	−1.14950	3.09900
H	6.96390	−3.05130	0.90940
H	9.26700	−1.12830	1.66080
H	5.84110	−1.44960	2.28050
H	1.90450	−2.89480	4.63900
H	−1.17950	−2.72270	4.55700
H	−0.33000	−4.84360	3.54960
H	0.58020	−5.18230	5.02140
H	−1.18000	−5.14670	5.06260
H	10.58660	−4.91210	1.51970
H	−0.49450	−1.73980	7.92880
H	0.27130	−1.03680	6.50840
H	−1.46820	−1.37570	6.50600
**Marilzafurollene C_147**			
C	2.68350	−1.46650	0.74910
O	4.00120	−1.92310	1.05590
C	1.73410	−2.65820	0.51240
C	2.83100	−0.50960	−0.44040
C	4.94860	−1.29480	0.19450
C	1.52140	−3.52460	1.76580
Br	2.47960	−3.82890	−0.92100
C	4.25400	−0.01470	−0.28390
C	6.30870	−1.11620	0.89650
C	0.81040	−2.78570	2.88370
Cl	4.30360	1.28320	0.94690
C	6.99020	−2.45280	1.23810
C	8.39880	−2.24290	1.75740
O	6.22510	−3.10420	2.22830
C	9.46750	−2.72700	1.16680
C	1.33600	−2.56450	4.10010
C	0.63290	−1.82080	5.22040
C	0.41020	−2.73660	6.42650
O	1.40360	−0.71680	5.67270
C	10.52700	−3.20660	0.55980
Br	11.49730	−2.19580	−0.83870
C	1.49820	0.35500	4.74430
H	2.33880	−0.88420	1.60400
H	0.76980	−2.29560	0.15380
H	2.73670	−1.04420	−1.38580
H	2.09250	0.29240	−0.43830
H	5.07800	−1.94880	−0.67000
H	0.92020	−4.39700	1.50850

H	2.48090	−3.90460	2.12010
H	4.67550	0.37900	−1.20950
H	6.96260	−0.54520	0.23580
H	6.19780	−0.51970	1.80290
H	−0.18340	−2.42400	2.66080
H	7.01440	−3.08600	0.34830
H	8.49020	−1.66130	2.66320
H	5.30710	−2.94630	2.01830
H	2.32990	−2.92430	4.32690
H	−0.33930	−1.46670	4.87200
H	−0.19350	−3.60360	6.15690
H	1.35710	−3.09780	6.82840
H	−0.10830	−2.20620	7.22540
H	10.96530	−4.17430	0.75590
H	2.05590	1.17660	5.19370
H	2.02430	0.05840	3.83670
H	0.51170	0.73110	4.47050
**Marilzafurollene C_148**			
C	3.13600	−1.07890	0.95810
O	4.43930	−1.63050	1.14850
C	2.07500	−2.19740	0.90420
C	3.22170	−0.20520	−0.30040
C	5.32300	−1.15440	0.13500
C	1.94960	−3.00080	2.21110
Br	2.53740	−3.48250	−0.55090
C	4.69200	0.15650	−0.34740
C	6.77200	−1.06800	0.65250
C	1.45440	−2.17240	3.38190
Cl	5.01020	1.51300	0.77500
C	7.36810	−2.44150	1.00730
C	8.84470	−2.34020	1.33450
O	6.68000	−2.95030	2.12900
C	9.78070	−2.96410	0.65630
C	0.27080	−2.34910	3.99230
C	−0.22260	−1.52480	5.16700
C	−1.52320	−0.79780	4.81560
O	−0.49390	−2.34610	6.29330
C	10.70610	−3.58310	−0.03760
Br	11.57650	−2.76240	−1.61500
C	0.66530	−2.87220	6.92530
H	2.94000	−0.41970	1.80420
H	1.10300	−1.77050	0.65170
H	2.96250	−0.77530	−1.19250
H	2.56490	0.66430	−0.26250
H	5.28190	−1.86960	−0.68880
H	1.26450	−3.83470	2.05350
H	2.91210	−3.44080	2.47480

H	5.02930	0.45220	−1.34150
H	7.38530	−0.60700	−0.12310
H	6.83240	−0.40800	1.51870
H	2.11960	−1.39660	3.73360
H	7.22080	−3.12860	0.17090
H	9.10380	−1.71500	2.17640
H	5.75980	−2.72300	2.01610
H	−0.39910	−3.12510	3.65010
H	0.52790	−0.77760	5.43290
H	−1.38460	−0.13160	3.96380
H	−2.31570	−1.50360	4.56570
H	−1.86890	−0.19510	5.65550
H	11.07680	−4.57520	0.17510
H	0.37040	−3.46000	7.79450
H	1.22870	−3.52650	6.25900
H	1.32410	−2.07420	7.27000
	**Marilzafurollene C_149**		
C	2.68710	−0.14210	0.43220
O	3.74480	−0.93230	0.97810
C	1.42480	−0.99440	0.18420
C	3.26630	0.51330	−0.82900
C	4.90510	−0.83870	0.15630
C	0.66460	−1.40250	1.46230
Br	1.89650	−2.63120	−0.85760
C	4.75700	0.50800	−0.55650
C	6.18520	−1.04680	0.98700
C	1.49690	−2.20420	2.44600
Cl	5.17880	1.88340	0.50670
C	6.30190	−2.47240	1.55500
C	7.63280	−2.68970	2.24690
O	5.25960	−2.67030	2.48460
C	8.50520	−3.59590	1.86900
C	1.64800	−1.89240	3.74400
C	2.48900	−2.68480	4.72750
C	3.52350	−1.78540	5.40890
O	1.68350	−3.24400	5.75550
C	9.37160	−4.49810	1.47330
Br	10.77400	−4.10620	0.13190
C	0.86640	−4.32560	5.32930
H	2.46000	0.64400	1.15410
H	0.73630	−0.42200	−0.43940
H	3.05920	−0.09730	−1.70870
H	2.86580	1.51000	−1.01590
H	4.83690	−1.62730	−0.59560
H	0.28310	−0.51030	1.96040
H	−0.20670	−1.99880	1.18990
H	5.35960	0.58090	−1.46260

H	7.04750	−0.84900	0.34850
H	6.23590	−0.32110	1.79980
H	1.99120	−3.07990	2.04930
H	6.18230	−3.19610	0.74550
H	7.84020	−2.04940	3.09180
H	4.49990	−2.19510	2.15220
H	1.15080	−1.02240	4.14760
H	3.01790	−3.48200	4.20100
H	4.18470	−1.32160	4.67630
H	3.04380	−0.99070	5.98030
H	4.14440	−2.36170	6.09460
H	9.40700	−5.51740	1.82910
H	0.14240	−4.01430	4.57560
H	1.46840	−5.13820	4.92050
H	0.31010	−4.71830	6.18030
**Marilzafurollene C_150**			
C	3.53720	−0.15920	0.27650
O	4.82200	−0.29410	0.88210
C	2.46140	−0.82400	1.15560
C	3.68380	−0.70000	−1.15220
C	5.75670	−0.80910	−0.06290
C	2.70660	−2.31450	1.45470
Br	0.68090	−0.59590	0.28710
C	5.15540	−0.47510	−1.43260
C	7.17430	−0.27140	0.21200
C	1.74070	−2.86100	2.48720
Cl	5.42990	1.23170	−1.89500
C	7.77770	−0.79570	1.52800
C	8.09270	−2.27900	1.46900
O	8.97220	−0.08920	1.79210
C	7.48840	−3.18200	2.20730
C	2.10690	−3.36010	3.67940
C	1.14470	−3.90950	4.71520
C	1.45560	−5.37670	5.02100
O	1.24400	−3.19570	5.93910
C	6.87010	−4.09540	2.91730
Br	5.24570	−5.01910	2.26520
C	0.71610	−1.87720	5.88960
H	3.32920	0.91040	0.21800
H	2.42060	−0.27980	2.10050
H	3.46720	−1.76650	−1.19570
H	3.02660	−0.20060	−1.86480
H	5.75120	−1.89560	0.03880
H	2.61660	−2.91520	0.55040
H	3.72540	−2.44970	1.81950
H	5.54070	−1.10810	−2.23270
H	7.83170	−0.52930	−0.61940

H	7.13640	0.81790	0.24320
H	0.69390	−2.83680	2.21890
H	7.08190	−0.59930	2.34730
H	8.86460	−2.57200	0.77230
H	8.74930	0.78760	2.07230
H	3.15270	−3.38580	3.95050
H	0.12230	−3.84550	4.33740
H	1.38270	−5.98980	4.12230
H	2.46200	−5.49150	5.42440
H	0.75570	−5.77710	5.75460
H	7.17600	−4.42810	3.89840
H	0.80090	−1.41440	6.87280
H	1.25780	−1.25010	5.18070
H	−0.33910	−1.88420	5.61380
**Marilzafurollene C_151**			
C	3.85810	−0.96730	1.43160
O	4.54630	−2.10320	0.90060
C	2.44620	−1.38030	1.88750
C	3.88680	0.10260	0.32990
C	5.32800	−1.72270	−0.22990
C	2.47940	−2.40080	3.03770
Br	1.44240	−2.15740	0.34570
C	4.44480	−0.64610	−0.86450
C	6.72540	−1.25350	0.24200
C	1.09770	−2.70570	3.58300
Cl	5.29960	0.42430	−2.00980
C	7.54420	−2.36250	0.92700
C	8.97030	−1.92080	1.18990
O	6.92850	−2.68590	2.15450
C	10.02010	−2.52200	0.67830
C	0.70320	−2.45610	4.84270
C	−0.68050	−2.76070	5.38590
C	−0.60150	−3.73310	6.56550
O	−1.31760	−1.58200	5.85660
C	11.05940	−3.12540	0.15190
Br	11.81890	−2.59810	−1.59860
C	−1.73690	−0.69550	4.82820
H	4.42030	−0.59500	2.28980
H	1.89970	−0.49320	2.21240
H	2.91200	0.54420	0.12110
H	4.56010	0.91020	0.61960
H	5.44060	−2.57090	−0.90620
H	2.93570	−3.33170	2.69880
H	3.10740	−2.02410	3.84620
H	3.63000	−1.12540	−1.40950
H	7.28630	−0.91320	−0.62950
H	6.64570	−0.38750	0.89990

H	0.40240	−3.15580	2.88820
H	7.54480	−3.25390	0.29550
H	9.09220	−1.06470	1.83720
H	5.99200	−2.74630	1.98540
H	1.39270	−2.00610	5.54250
H	−1.29000	−3.21800	4.60380
H	−0.13400	−4.67290	6.27050
H	−0.02170	−3.31210	7.38730
H	−1.59700	−3.96260	6.94560
H	11.59150	−3.94780	0.60730
H	−0.89360	−0.32200	4.24630
H	−2.44040	−1.18100	4.15060
H	−2.23990	0.16390	5.27150
**Marilzafurollene C_152**			
C	3.28820	−0.16060	0.65590
O	4.51530	−0.73500	1.10510
C	2.13290	−0.54270	1.60060
C	3.14010	−0.58280	−0.81120
C	5.21120	−1.32740	0.00680
C	1.89870	−2.05720	1.75610
Br	0.45930	0.33020	0.95770
C	4.58300	−0.71450	−1.25190
C	6.73700	−1.16740	0.15690
C	0.89300	−2.37320	2.84570
Cl	5.24960	0.90360	−1.62540
C	7.31130	−1.92890	1.36430
C	8.82700	−1.90140	1.36970
O	6.83430	−1.32130	2.54490
C	9.57070	−2.98240	1.30980
C	1.16610	−3.08630	3.95100
C	0.15930	−3.39930	5.04220
C	−0.02360	−4.91100	5.19830
O	0.59110	−2.89790	6.29870
C	10.30110	−4.06980	1.23720
Br	10.88170	−4.84450	−0.48960
C	0.53570	−1.48240	6.41270
H	3.41980	0.92230	0.68830
H	2.34840	−0.11740	2.58230
H	2.64710	−1.55000	−0.89790
H	2.57070	0.13370	−1.40420
H	4.96420	−2.39060	0.01510
H	1.53840	−2.49210	0.82450
H	2.84180	−2.55290	1.99010
H	4.70460	−1.34050	−2.13670
H	7.21060	−1.54140	−0.75210
H	7.00760	−0.11300	0.22680
H	−0.10530	−1.98590	2.69680

H	6.96080	−2.96330	1.34190
H	9.28540	−0.92490	1.42790
H	5.94120	−1.03410	2.37190
H	2.16220	−3.47550	4.10650
H	−0.80740	−2.96050	4.78700
H	−0.37200	−5.36370	4.26970
H	0.91190	−5.39570	5.47890
H	−0.75780	−5.13440	5.97250
H	10.64990	−4.63690	2.08790
H	0.85130	−1.18550	7.41290
H	1.19710	−0.99190	5.69770
H	−0.47910	−1.11310	6.25950
**Marilzafurollene C_153**			
C	3.46490	−0.33940	1.13500
O	4.73970	−0.96040	1.30560
C	2.34830	−1.22350	1.72050
C	3.33300	−0.03790	−0.36360
C	5.40500	−1.05960	0.04770
C	2.46610	−1.37140	3.24690
Br	2.41560	−3.03500	0.88080
C	4.77810	0.04470	−0.81060
C	6.93560	−1.00880	0.21900
C	1.28260	−2.09940	3.85440
Cl	5.43470	1.66150	−0.41640
C	7.49530	−2.21490	0.99340
C	9.01110	−2.22880	0.98290
O	7.04320	−2.13770	2.32760
C	9.72220	−3.20450	0.46560
C	0.44920	−1.57180	4.76840
C	−0.74050	−2.28320	5.39340
C	−2.05630	−1.69770	4.87460
O	−0.75520	−3.67610	5.10590
C	10.41970	−4.18060	−0.06550
Br	10.96920	−4.17930	−1.96750
C	0.10150	−4.44850	5.93730
H	3.49560	0.61020	1.67170
H	1.37780	−0.78880	1.47510
H	2.84110	−0.85930	−0.88510
H	2.76550	0.87020	−0.56880
H	5.12800	−2.02140	−0.38810
H	3.37950	−1.91130	3.49960
H	2.55160	−0.38610	3.70680
H	4.90600	−0.12200	−1.88080
H	7.39010	−0.98460	−0.77250
H	7.23690	−0.08440	0.71310
H	1.12030	−3.11160	3.51060
H	7.11380	−3.13870	0.55240

H	9.49800	−1.37660	1.43410
H	6.14050	−1.82870	2.29700
H	0.60980	−0.55810	5.10670
H	−0.70140	−2.13080	6.47370
H	−2.12780	−0.63120	5.08940
H	−2.14670	−1.83080	3.79620
H	−2.90990	−2.18850	5.34230
H	10.75570	−5.05900	0.46590
H	−0.20860	−4.39010	6.98140
H	0.05670	−5.49460	5.63470
H	1.14020	−4.12460	5.86590
**Marilzafurollene C_154**			
C	3.38080	−0.15140	0.97880
O	4.64040	−0.77340	1.23630
C	2.24280	−0.92600	1.66890
C	3.25990	−0.04640	−0.54720
C	5.30480	−1.05530	0.00610
C	2.35440	−0.87500	3.20210
Br	2.27150	−2.83430	1.07800
C	4.70750	−0.05880	−0.99350
C	6.83570	−1.02090	0.17750
C	1.18170	−1.54470	3.89080
Cl	5.40360	1.57920	−0.81190
C	7.36260	−2.12870	1.10650
C	8.87750	−2.17990	1.10880
O	6.90690	−1.86790	2.41600
C	9.56670	−3.23140	0.72820
C	1.28820	−2.57990	4.74180
C	0.12520	−3.27510	5.43000
C	0.07390	−2.90800	6.91500
O	−1.13010	−2.92400	4.85970
C	10.24250	−4.28440	0.33380
Br	10.79930	−4.54410	−1.54810
C	−1.43270	−3.62200	3.65810
H	3.43180	0.85930	1.38730
H	1.28370	−0.50740	1.36030
H	2.74880	−0.91710	−0.95860
H	2.71570	0.84040	−0.87320
H	5.00400	−2.05910	−0.30020
H	3.28850	−1.33910	3.52190
H	2.39170	0.16190	3.53740
H	4.83360	−0.36800	−2.03170
H	7.29300	−1.13820	−0.80610
H	7.15930	−0.04740	0.54790
H	0.20330	−1.14440	3.66600
H	6.96030	−3.09270	0.78660
H	9.38330	−1.28800	1.44870

H	6.01360	−1.54030	2.33850
H	2.26760	−2.97430	4.97220
H	0.27570	−4.35330	5.34900
H	0.99990	−3.17960	7.42230
H	−0.08170	−1.83720	7.04950
H	−0.74360	−3.42780	7.41480
H	10.55490	−5.09370	0.97740
H	−0.67990	−3.45410	2.88700
H	−1.51070	−4.69510	3.83700
H	−2.39120	−3.27910	3.26870
**Marilzafurollene C_155**			
C	2.84070	−0.68950	0.86550
O	3.57110	−1.80410	0.34690
C	1.32760	−0.93030	0.69180
C	3.37800	0.54900	0.13270
C	4.76080	−1.35540	−0.29760
C	0.80700	−2.15010	1.47150
Br	0.91620	−1.21140	−1.23960
C	4.28890	−0.04240	−0.92530
C	5.89850	−1.23210	0.74430
C	0.93010	−1.98920	2.97480
Cl	5.60990	1.06410	−1.39280
C	6.28190	−2.57500	1.39090
C	7.52390	−2.44530	2.25020
O	5.20980	−3.01010	2.19700
C	8.63260	−3.11240	2.02490
C	1.61790	−2.82160	3.77420
C	1.74690	−2.65860	5.27760
C	1.06480	−3.81590	6.01120
O	3.10680	−2.65350	5.68580
C	9.73650	−3.77850	1.78200
Br	11.16560	−3.07270	0.60790
C	3.84750	−1.51740	5.26160
H	3.07770	−0.58730	1.92540
H	0.77610	−0.04170	1.00260
H	2.59800	1.17610	−0.29990
H	3.95110	1.16600	0.82570
H	5.05440	−2.06520	−1.07200
H	−0.24670	−2.30700	1.23910
H	1.33140	−3.05020	1.14750
H	3.71070	−0.26200	−1.82430
H	6.78270	−0.83660	0.24280
H	5.64530	−0.50490	1.51640
H	0.41860	−1.14130	3.40750
H	6.45010	−3.31860	0.60860
H	7.45720	−1.76150	3.08360
H	4.41000	−2.86930	1.69690

H	2.13120	−3.67320	3.34990
H	1.27290	−1.72510	5.58700
H	0.00540	−3.87220	5.76020
H	1.52310	−4.77100	5.75330
H	1.14450	−3.69080	7.09120
H	9.97130	−4.75210	2.18680
H	4.86300	−1.57970	5.65240
H	3.91780	−1.46010	4.17510
H	3.40150	−0.59380	5.63210
	**Marilzafurollene C_156**		
C	3.70530	−0.48040	0.94900
O	5.09210	−0.66720	1.23070
C	2.86170	−1.60930	1.56880
C	3.59520	−0.34870	−0.57460
C	5.85480	−0.57640	0.03030
C	2.93600	−1.61090	3.10470
Br	3.48910	−3.37640	0.88230
C	4.95880	0.19220	−0.94950
C	7.24040	0.03970	0.30400
C	2.00940	−2.63540	3.73020
Cl	4.99900	1.95900	−0.66660
C	8.16210	−0.86740	1.13890
C	8.62970	−2.08710	0.36720
O	9.29550	−0.11820	1.52760
C	8.38730	−3.32300	0.74020
C	0.98610	−2.33900	4.54860
C	0.05920	−3.36620	5.17140
C	0.11690	−3.29730	6.69950
O	−1.29000	−3.13640	4.79220
C	8.16700	−4.56400	1.10400
Br	6.58400	−5.58550	0.49950
C	−1.57670	−3.45420	3.43700
H	3.41480	0.47270	1.39410
H	1.82230	−1.49330	1.25810
H	3.45340	−1.32280	−1.04280
H	2.77330	0.29410	−0.89080
H	5.97170	−1.59110	−0.35420
H	3.95740	−1.81740	3.42720
H	2.68470	−0.62060	3.48660
H	5.21890	0.01110	−1.99290
H	7.72710	0.28190	−0.64160
H	7.10310	0.98700	0.82600
H	2.21020	−3.66870	3.48350
H	7.63770	−1.18160	2.04480
H	9.19520	−1.89080	−0.53180
H	9.03770	0.48650	2.20920
H	0.77980	−1.30830	4.79880

H	0.36010	−4.36880	4.86090
H	1.12780	−3.48140	7.06410
H	−0.19960	−2.31970	7.06380
H	−0.53830	−4.04560	7.14590
H	8.81160	−5.14010	1.75160
H	−2.63390	−3.27870	3.23810
H	−1.00250	−2.83660	2.74560
H	−1.36670	−4.50320	3.22440
	**Marilzafurollene C_157**		
C	3.03700	−0.38610	0.07160
O	4.08880	−0.54920	1.02050
C	1.72100	−1.04450	0.54050
C	3.60580	−0.86670	−1.26950
C	5.29040	−0.94080	0.35770
C	1.77350	−2.55220	0.86280
Br	0.34750	−0.75500	−0.87420
C	5.08190	−0.56620	−1.11370
C	6.52370	−0.33070	1.05150
C	2.45350	−2.85830	2.18400
Cl	5.38630	1.17030	−1.42140
C	6.79380	−0.94410	2.43690
C	7.34760	−2.35330	2.34250
O	7.72640	−0.13510	3.12400
C	6.64880	−3.42890	2.62430
C	1.83690	−3.40130	3.24840
C	2.48820	−3.72040	4.58430
C	2.61930	−5.23320	4.77580
O	3.79040	−3.16110	4.70910
C	5.92900	−4.50240	2.84480
Br	4.92950	−5.40820	1.39550
C	3.79560	−1.78410	5.06290
H	2.86410	0.68840	−0.00830
H	1.36110	−0.51220	1.42230
H	3.46500	−1.93920	−1.39660
H	3.15340	−0.36840	−2.12740
H	5.34110	−2.02950	0.40690
H	0.75240	−2.93090	0.91730
H	2.25860	−3.11710	0.06820
H	5.71020	−1.14430	−1.79220
H	7.40430	−0.45320	0.41940
H	6.36840	0.74380	1.15220
H	3.50540	−2.62130	2.24790
H	5.86550	−0.95090	3.01190
H	8.37710	−2.43900	2.02690
H	7.30450	0.67870	3.36120
H	0.78670	−3.64660	3.17940
H	1.84530	−3.33310	5.37700

H	3.24490	−5.67610	4.00100
H	3.06970	−5.46510	5.74100
H	1.64550	−5.72190	4.73800
H	5.80910	−4.98200	3.80540
H	4.82440	−1.43630	5.15270
H	3.29900	−1.16900	4.31170
H	3.30700	−1.62160	6.02430
	**Marilzafurollene C_158**		
C	3.24430	−0.78600	0.91740
O	3.91590	−1.84840	0.23640
C	1.72210	−1.03000	0.89070
C	3.70150	0.51150	0.23340
C	5.05520	−1.34100	−0.45320
C	1.28680	−2.34080	1.57100
Br	1.09450	−1.08820	−1.00190
C	4.53150	0.01370	−0.93490
C	6.26640	−1.28990	0.51000
C	1.62650	−2.39520	3.04880
Cl	5.81000	1.16510	−1.41190
C	6.68580	−2.67240	1.04160
C	7.98300	−2.59960	1.82260
O	5.66570	−3.15940	1.88650
C	9.07080	−3.25200	1.48140
C	0.71890	−2.41530	4.03880
C	1.06040	−2.47590	5.51620
C	0.50810	−1.25400	6.25470
O	0.49420	−3.62570	6.12750
C	10.15360	−3.90000	1.12250
Br	11.50290	−3.11870	−0.09740
C	1.11660	−4.84890	5.75800
H	3.59750	−0.76360	1.94910
H	1.20880	−0.19280	1.36660
H	2.87960	1.15100	−0.08980
H	4.31910	1.09130	0.92030
H	5.29330	−1.98390	−1.30150
H	0.21080	−2.46860	1.44730
H	1.75470	−3.19260	1.07600
H	3.88600	−0.13910	−1.80140
H	7.11450	−0.86400	−0.02790
H	6.07570	−0.61310	1.34340
H	2.67850	−2.42510	3.29680
H	6.79850	−3.36320	0.20280
H	7.97610	−1.97020	2.70040
H	4.84120	−3.00950	1.43110
H	−0.33520	−2.39080	3.80160
H	2.14490	−2.49250	5.64230
H	0.91600	−0.32900	5.84650

H	−0.57850	−1.20470	6.18040
H	0.76650	−1.29200	7.31310
H	10.41200	−4.89750	1.44700
H	0.64580	−5.67290	6.29410
H	1.01660	−5.04840	4.69060
H	2.17670	−4.84630	6.01460
**Marilzafurollene C_159**			
C	3.43350	−1.15630	0.93780
O	4.83340	−1.19040	1.21580
C	2.63570	−1.41280	2.22850
C	3.22320	−2.19530	−0.16910
C	5.52250	−1.97640	0.24390
C	1.11190	−1.33870	2.02340
Br	3.16950	−0.03980	3.57390
C	4.53720	−2.14060	−0.91990
C	6.89470	−1.36390	−0.09850
C	0.34090	−1.69180	3.28050
Cl	4.53900	−0.73120	−2.02230
C	7.86720	−1.35260	1.09390
C	9.26330	−0.94380	0.66770
O	7.39270	−0.43540	2.05560
C	10.31640	−1.71760	0.80010
C	−0.50010	−2.73350	3.39170
C	−1.26820	−3.08660	4.65180
C	−2.77720	−3.06080	4.39610
O	−0.94170	−4.39350	5.10140
C	11.35730	−2.50580	0.92790
Br	11.90650	−3.77620	−0.48770
C	0.35750	−4.50810	5.66580
H	3.20250	−0.16240	0.55080
H	2.91050	−2.38390	2.64250
H	3.08920	−3.18850	0.26170
H	2.35940	−1.98200	−0.79960
H	5.67540	−2.96100	0.68960
H	0.82510	−0.33560	1.70580
H	0.80840	−2.01630	1.22470
H	4.72840	−3.03370	−1.51600
H	7.33600	−1.94720	−0.90810
H	6.77850	−0.35020	−0.48390
H	0.49740	−1.04350	4.13150
H	7.89670	−2.34540	1.54860
H	9.35980	0.04260	0.23800
H	6.44100	−0.50990	2.07780
H	−0.66190	−3.38680	2.54640
H	−1.04020	−2.36250	5.43660
H	−3.10310	−2.07580	4.06070
H	−3.06220	−3.78720	3.63470

H	−3.32820	−3.30050	5.30570
H	12.00220	−2.54080	1.79390
H	1.13710	−4.27920	4.93840
H	0.47550	−3.84480	6.52360
H	0.51570	−5.53000	6.01030
**Marilzafurollene C_160**			
C	2.95950	−0.96440	1.03970
O	4.12930	−1.78040	1.11500
C	1.72410	−1.81800	0.68630
C	3.28160	0.15090	0.03650
C	5.14700	−1.25540	0.26510
C	1.36950	−2.85120	1.76910
Br	2.05950	−2.80700	−1.01400
C	4.79320	0.22710	0.10240
C	6.55080	−1.56950	0.81780
C	0.92680	−2.21190	3.07060
Cl	5.28300	1.19980	1.52180
C	6.86390	−3.07580	0.84560
C	8.30910	−3.33770	1.22010
O	6.02140	−3.68870	1.79690
C	9.16000	−3.95560	0.43300
C	1.58070	−2.33530	4.23860
C	1.16220	−1.70050	5.55430
C	2.11450	−0.56330	5.93230
O	−0.15020	−1.15380	5.49910
C	10.00220	−4.56360	−0.36840
Br	11.11070	−3.58390	−1.68390
C	−1.18380	−2.11310	5.68040
H	2.82510	−0.50520	2.02020
H	0.86560	−1.16800	0.51050
H	2.97910	−0.13160	−0.97200
H	2.79150	1.09500	0.27580
H	5.03220	−1.73660	−0.70810
H	0.55590	−3.48600	1.41720
H	2.22100	−3.51000	1.94570
H	5.23840	0.67650	−0.78590
H	7.28690	−1.06710	0.18850
H	6.67220	−1.15240	1.81830
H	0.02480	−1.61770	3.02810
H	6.65100	−3.50920	−0.13440
H	8.62010	−2.98540	2.19270
H	5.16850	−3.26570	1.72330
H	2.48720	−2.92320	4.27480
H	1.21280	−2.46250	6.33440
H	3.14110	−0.91970	6.02120
H	2.09820	0.22810	5.18250
H	1.83200	−0.12180	6.88810

H	10.17180	−5.63020	−0.39110
H	−1.12730	−2.56740	6.67040
H	−2.15420	−1.62460	5.59330
H	−1.14020	−2.90570	4.93280
**Marilzafurollene C_161**			
C	3.09620	−0.59470	0.89660
O	3.64610	−1.74020	0.24120
C	1.56020	−0.60420	0.76180
C	3.79220	0.62430	0.27280
C	4.90070	−1.40810	−0.34640
C	0.90170	−1.83150	1.41580
Br	1.06280	−0.58960	−1.16980
C	4.62940	0.01590	−0.83650
C	6.02310	−1.55690	0.70950
C	1.13830	−1.89320	2.91210
Cl	6.10230	0.96220	−1.18690
C	6.18540	−2.99660	1.23050
C	7.40380	−3.13260	2.12180
O	5.03420	−3.34200	1.96920
C	8.40900	−3.93510	1.85590
C	1.87120	−2.83890	3.52510
C	2.14290	−2.91540	5.01770
C	3.60060	−2.55800	5.31750
O	1.32420	−2.02230	5.76380
C	9.41040	−4.73370	1.57230
Br	10.96540	−4.14600	0.49730
C	0.01480	−2.51480	6.01680
H	3.37410	−0.63920	1.95090
H	1.14740	0.30520	1.20110
H	3.10100	1.37930	−0.10260
H	4.43150	1.10230	1.01590
H	5.10440	−2.07090	−1.18830
H	−0.17460	−1.81020	1.24310
H	1.27170	−2.74320	0.94460
H	4.03990	−0.02800	−1.75380
H	6.96670	−1.25510	0.25300
H	5.86740	−0.87370	1.54510
H	0.68660	−1.10720	3.50070
H	6.26910	−3.68010	0.38240
H	7.41120	−2.52420	3.01440
H	4.28690	−3.00420	1.48120
H	2.32970	−3.61950	2.93410
H	1.96760	−3.94150	5.34660
H	4.28640	−3.22320	4.79110
H	3.82610	−1.53690	5.00930
H	3.81050	−2.63920	6.38390
H	9.48640	−5.76400	1.88790

H	−0.55760	−1.76500	6.56290
H	−0.52190	−2.73860	5.09440
H	0.04710	−3.41840	6.62670
	**Marilzafurollene C_162**		
C	3.65350	−1.59850	1.26170
O	4.76810	−1.02590	1.94480
C	2.80350	−2.45250	2.22100
C	4.23370	−2.35500	0.06160
C	5.98220	−1.35160	1.27350
C	2.15210	−1.60440	3.32610
Br	3.94190	−3.86230	3.06310
C	5.54570	−1.63640	−0.17190
C	7.03730	−0.24970	1.51480
C	1.20740	−2.41030	4.19660
Cl	5.24480	−0.13640	−1.10070
C	8.47820	−0.61800	1.10450
C	8.67160	−0.65830	−0.39900
O	9.34730	0.35490	1.64590
C	9.10250	−1.71690	−1.04620
C	−0.11110	−2.17850	4.30850
C	−1.05470	−2.98740	5.17880
C	−1.72870	−2.09770	6.22630
O	−2.08780	−3.57850	4.40390
C	9.48790	−2.79220	−1.69100
Br	8.19660	−4.14150	−2.34530
C	−1.65670	−4.65420	3.58130
H	3.05100	−0.77100	0.88350
H	2.02440	−2.96290	1.65270
H	4.43420	−3.39610	0.31510
H	3.57650	−2.34710	−0.80830
H	6.33920	−2.28320	1.71630
H	2.92280	−1.16170	3.95840
H	1.60480	−0.77390	2.87840
H	6.25750	−2.24560	−0.72810
H	6.73340	0.68950	1.05270
H	7.03960	−0.04950	2.58710
H	1.65330	−3.22110	4.75550
H	8.74130	−1.58220	1.54570
H	8.42630	0.24840	−0.93430
H	10.24010	0.05210	1.55810
H	−0.56330	−1.36990	3.75240
H	−0.49630	−3.76830	5.69920
H	−0.99050	−1.62060	6.87140
H	−2.32210	−1.31310	5.75620
H	−2.39510	−2.68260	6.86040
H	10.51270	−3.04460	−1.92220
H	−0.92700	−4.32940	2.83880

H	−1.21570	−5.45470	4.17670
H	−2.51140	−5.06840	3.04660
**Marilzafurollene C_163**			
C	3.78720	−0.64880	1.19860
O	4.45100	−1.90360	1.02360
C	2.39010	−0.87770	1.80540
C	3.78190	0.02720	−0.18060
C	5.19180	−1.90480	−0.19500
C	2.45880	−1.49800	3.21150
Br	1.32770	−2.07700	0.61360
C	4.29110	−1.06360	−1.10210
C	6.60820	−1.33540	0.06050
C	1.09600	−1.60510	3.86820
Cl	5.10820	−0.41470	−2.55100
C	7.44620	−2.19050	1.02780
C	8.88310	−1.71180	1.09300
O	6.87450	−2.10800	2.31500
C	9.91050	−2.45810	0.75740
C	0.53390	−2.75900	4.26500
C	−0.83060	−2.87260	4.91840
C	−1.76740	−3.73970	4.07380
O	−0.73970	−3.47860	6.19970
C	10.92670	−3.21080	0.40800
Br	11.62260	−3.26140	−1.44460
C	−0.13660	−2.65940	7.19200
H	4.38120	−0.03830	1.88090
H	1.86040	0.07490	1.85480
H	2.80270	0.39920	−0.48360
H	4.47020	0.87320	−0.17770
H	5.27500	−2.92260	−0.57830
H	2.92500	−2.48300	3.15980
H	3.09310	−0.88810	3.85560
H	3.45420	−1.67480	−1.44360
H	7.13800	−1.29100	−0.89190
H	6.55720	−0.30690	0.41950
H	0.56150	−0.67740	4.01410
H	7.41940	−3.23360	0.70420
H	9.03280	−0.69970	1.43930
H	5.93210	−2.20350	2.20410
H	1.06460	−3.68920	4.12060
H	−1.27490	−1.87990	5.01510
H	−1.89320	−3.32350	3.07380
H	−1.38020	−4.75350	3.96900
H	−2.75380	−3.80660	4.53300
H	11.47170	−3.86100	1.07670
H	−0.13780	−3.18470	8.14700
H	0.89900	−2.42160	6.94650

H	−0.68740	−1.72720	7.32310
	**Marilzafurollene C_164**		
C	3.41960	−0.26550	0.31030
O	4.75720	−0.14560	0.79310
C	2.51120	−0.85760	1.40440
C	3.51070	−1.05440	−1.00360
C	5.66210	−0.76210	−0.11930
C	2.97290	−2.22150	1.95090
Br	0.65490	−1.01390	0.69370
C	4.92380	−0.75930	−1.46240
C	7.03680	−0.06640	−0.09480
C	2.16900	−2.66740	3.15610
Cl	4.97810	0.84090	−2.26160
C	7.78970	−0.24920	1.23580
C	8.22300	−1.68540	1.46520
O	8.93820	0.57350	1.22090
C	7.79800	−2.42350	2.46540
C	1.45200	−3.80190	3.21270
C	0.65210	−4.25400	4.41960
C	−0.83370	−4.37720	4.07310
O	1.08350	−5.52780	4.87660
C	7.37330	−3.17730	3.45160
Br	5.77000	−4.32790	3.29530
C	2.37270	−5.52790	5.47510
H	3.07740	0.74470	0.07960
H	2.47070	−0.14130	2.22620
H	3.40320	−2.12440	−0.83200
H	2.75090	−0.76010	−1.72830
H	5.77340	−1.80260	0.19040
H	2.92460	−2.98780	1.17810
H	4.01610	−2.15620	2.26060
H	5.31020	−1.50040	−2.16290
H	7.64990	−0.44130	−0.91530
H	6.89560	0.99830	−0.28250
H	2.19390	−2.01160	4.01440
H	7.15090	0.07860	2.05960
H	8.92330	−2.08920	0.74870
H	8.66640	1.47460	1.32610
H	1.42430	−4.46050	2.35670
H	0.75880	−3.52400	5.22440
H	−1.23620	−3.42540	3.72510
H	−0.99650	−5.11720	3.28930
H	−1.41140	−4.68400	4.94510
H	7.84310	−3.24600	4.42190
H	2.60670	−6.52980	5.83450
H	3.14930	−5.24260	4.76490
H	2.41340	−4.84910	6.32780

**Marilzafurollene C_165**			
C	3.54000	−0.73690	0.44400
O	4.86720	−0.26980	0.68190
C	3.00550	−1.40810	1.72210
C	3.64700	−1.67040	−0.77340
C	5.78590	−0.95750	−0.15550
C	1.55430	−1.90260	1.59380
Br	3.10220	−0.08800	3.21520
C	4.96420	−1.26820	−1.40760
C	7.07800	−0.14130	−0.35440
C	1.11270	−2.68490	2.81480
Cl	4.72030	0.17760	−2.43220
C	7.84410	0.10080	0.95980
C	8.33960	−1.18610	1.59330
O	8.95070	0.93910	0.69650
C	7.85820	−1.66600	2.71760
C	0.76030	−3.98100	2.80360
C	0.33940	−4.76500	4.03230
C	−1.07760	−5.32000	3.86810
O	1.20160	−5.87180	4.25420
C	7.33150	−2.15250	3.81600
Br	5.78040	−3.38310	3.78510
C	2.50960	−5.51580	4.68320
H	2.92880	0.13130	0.19300
H	3.65820	−2.23800	1.99600
H	3.70890	−2.71060	−0.45060
H	2.79880	−1.58410	−1.45310
H	6.02520	−1.90630	0.32780
H	0.88000	−1.05780	1.44950
H	1.45020	−2.53820	0.71400
H	5.40300	−2.05320	−2.02450
H	7.72390	−0.65250	−1.06930
H	6.82940	0.82180	−0.80020
H	1.09550	−2.13850	3.74760
H	7.18810	0.62070	1.66260
H	9.12560	−1.70640	1.06590
H	8.63650	1.82560	0.58490
H	0.77430	−4.53490	1.87600
H	0.35440	−4.11200	4.90730
H	−1.79890	−4.51790	3.70960
H	−1.13970	−6.00140	3.01930
H	−1.38340	−5.87030	4.75810
H	7.67780	−1.93840	4.81650
H	3.03940	−4.93430	3.92810
H	2.48030	−4.93960	5.60890
H	3.09010	−6.41900	4.87070

**Marilzafurollene C_166**			
C	3.98400	−0.65390	1.41840
O	5.00230	−1.54090	0.95080
C	2.71970	−1.45490	1.78220
C	3.78350	0.38760	0.30720
C	5.72340	−0.94540	−0.12310
C	2.96970	−2.44760	2.92980
Br	2.06580	−2.45110	0.17930
C	4.61620	−0.16390	−0.83350
C	6.88680	−0.09670	0.44460
C	1.70000	−3.13970	3.38620
Cl	5.20470	1.11070	−1.93710
C	7.92680	−0.92280	1.22280
C	8.68740	−1.88180	0.32640
O	8.84230	−0.02800	1.82110
C	8.66730	−3.18640	0.47800
C	1.17540	−3.03520	4.61850
C	−0.09640	−3.72730	5.07220
C	0.18200	−4.66060	6.25320
O	−1.06750	−2.78480	5.50300
C	8.62590	−4.49090	0.61130
Br	7.27150	−5.60600	−0.30420
C	−1.65580	−2.03180	4.45100
H	4.35660	−0.14430	2.30850
H	1.92840	−0.76240	2.07380
H	2.74070	0.53320	0.02430
H	4.17610	1.35190	0.63170
H	6.11800	−1.72050	−0.78090
H	3.68970	−3.20520	2.61720
H	3.41730	−1.92590	3.77660
H	4.01420	−0.85590	−1.42430
H	7.39050	0.42800	−0.36770
H	6.48940	0.68040	1.09760
H	1.20390	−3.75420	2.64790
H	7.42120	−1.47290	2.02000
H	9.26250	−1.43340	−0.47100
H	9.35950	−0.50330	2.45590
H	1.66600	−2.42230	5.36090
H	−0.50350	−4.32250	4.25230
H	0.91260	−5.42490	5.98720
H	0.56840	−4.10920	7.11070
H	−0.72950	−5.16830	6.56880
H	9.30640	−5.07320	1.21530
H	−2.40680	−1.35750	4.86230
H	−0.91790	−1.42490	3.92520
H	−2.15090	−2.68220	3.72880

**Marilzafurollene C_167**			
C	3.84860	−1.17020	2.15230
O	4.60050	−2.21350	1.53300
C	2.35690	−1.54400	2.23780
C	4.15390	0.10530	1.35760
C	5.37880	−1.68700	0.46070
C	2.11760	−2.75900	3.14960
Br	1.65220	−1.94660	0.41220
C	5.52160	−0.19390	0.78040
C	6.67860	−2.49480	0.27990
C	0.64380	−3.05570	3.34810
Cl	6.77520	0.11840	2.01920
C	7.62950	−1.95300	−0.80560
C	8.88020	−2.80430	−0.91010
O	6.96850	−1.95000	−2.05420
C	10.09510	−2.33830	−0.72930
C	0.01000	−3.01240	4.53180
C	−1.46530	−3.30820	4.72760
C	−1.66320	−4.48200	5.69000
O	−2.14320	−2.19130	5.28410
C	11.31230	−1.88740	−0.53700
Br	12.17310	−1.88010	1.24540
C	−2.30980	−1.10340	4.38490
H	4.24740	−1.04510	3.16030
H	1.79470	−0.69070	2.62000
H	3.44160	0.23800	0.54310
H	4.13080	1.00740	1.96920
H	4.77360	−1.78490	−0.44250
H	2.60090	−3.64130	2.72780
H	2.58180	−2.58700	4.12150
H	5.75380	0.40590	−0.10030
H	7.20870	−2.53650	1.23210
H	6.41180	−3.52650	0.04840
H	0.08930	−3.31840	2.45810
H	7.91160	−0.92580	−0.56470
H	8.72640	−3.84840	−1.14310
H	7.55010	−1.58570	−2.70690
H	0.55830	−2.75000	5.42520
H	−1.91800	−3.56970	3.76900
H	−1.17860	−5.38410	5.31560
H	−1.24900	−4.26110	6.67400
H	−2.72280	−4.70330	5.81830
H	11.95160	−1.48480	−1.30920
H	−1.35190	−0.69340	4.06290
H	−2.87510	−1.40380	3.50180
H	−2.86270	−0.30450	4.87900

**Marilzafurollene C_168**			
C	3.36020	−0.46200	0.60050
O	4.67760	−0.78910	1.04210
C	2.34450	−0.64360	1.74460
C	3.12320	−1.29540	−0.66510
C	5.32980	−1.61210	0.07300
C	2.29390	−2.06130	2.34580
Br	0.53280	−0.13630	1.08450
C	4.52490	−1.43460	−1.22140
C	6.83620	−1.29570	−0.00990
C	1.42920	−2.12920	3.58970
Cl	4.97490	0.05640	−2.10260
C	7.60020	−1.63580	1.28170
C	9.09740	−1.48450	1.09860
O	7.16860	−0.76030	2.30040
C	9.94830	−2.47420	1.24580
C	0.34240	−2.90830	3.71830
C	−0.52700	−2.97510	4.95990
C	−1.96480	−2.55860	4.64020
O	−0.57890	−4.29510	5.48140
C	10.78600	−3.47440	1.38320
Br	11.28750	−4.65320	−0.12610
C	0.62790	−4.72890	6.09360
H	3.37800	0.59150	0.31640
H	2.59780	0.06860	2.53130
H	2.73160	−2.28240	−0.42340
H	2.42740	−0.82420	−1.36010
H	5.20270	−2.64520	0.40180
H	1.92930	−2.77920	1.61170
H	3.29860	−2.38080	2.62490
H	4.63120	−2.27550	−1.90770
H	7.26180	−1.87300	−0.83210
H	6.99480	−0.24670	−0.26350
H	1.72890	−1.49710	4.41350
H	7.36640	−2.65930	1.58340
H	9.44760	−0.49720	0.83460
H	6.23860	−0.60230	2.15910
H	0.03790	−3.54220	2.89790
H	−0.13400	−2.29740	5.72030
H	−2.00100	−1.54320	4.24420
H	−2.41400	−3.22300	3.90170
H	−2.58580	−2.58880	5.53560
H	11.27340	−3.74880	2.30740
H	1.45330	−4.76480	5.38170
H	0.90840	−4.07560	6.92070
H	0.49200	−5.73370	6.49350

**Marilzafurollene C_169**			
C	3.78780	−0.71440	1.23630
O	4.62570	−1.83250	1.54180
C	2.50340	−0.79670	2.08270
C	3.58620	−0.73770	−0.28760
C	5.27510	−2.29920	0.35990
C	1.65290	−2.05560	1.82920
Br	1.40710	0.83390	1.74180
C	4.19080	−2.06590	−0.69490
C	6.59630	−1.52080	0.14900
C	0.48970	−2.16950	2.79480
Cl	4.78940	−2.06430	−2.37670
C	7.63170	−1.75680	1.26330
C	8.97570	−1.15010	0.91160
O	7.15850	−1.16690	2.45420
C	10.07870	−1.85340	0.79320
C	0.32200	−3.17470	3.67010
C	−0.84100	−3.28620	4.63820
C	−1.63760	−4.56930	4.38840
O	−0.38690	−3.33050	5.98300
C	11.17040	−2.57000	0.66740
Br	11.71860	−3.37570	−1.05580
C	0.12070	−2.09490	6.46840
H	4.32950	0.19410	1.50500
H	2.78680	−0.76530	3.13620
H	2.54740	−0.63640	−0.60160
H	4.14150	0.08530	−0.73900
H	5.49430	−3.36370	0.45150
H	1.25790	−2.05510	0.81340
H	2.27760	−2.94500	1.92150
H	3.43730	−2.85080	−0.61700
H	7.04000	−1.84630	−0.79280
H	6.40820	−0.45280	0.03390
H	−0.24080	−1.37350	2.75470
H	7.74480	−2.83040	1.43070
H	8.99030	−0.08080	0.75880
H	6.23480	−1.38960	2.52780
H	1.04860	−3.97330	3.71650
H	−1.51140	−2.43440	4.50710
H	−2.03290	−4.59620	3.37270
H	−1.01720	−5.45430	4.53190
H	−2.48130	−4.64110	5.07490
H	11.86170	−2.78710	1.46850
H	0.41620	−2.20770	7.51140
H	0.99830	−1.76940	5.90890
H	−0.63540	−1.31040	6.41870

**Marilzafurollene C_170**			
C	3.08840	−1.09580	1.20370
O	4.32710	−1.80650	1.21610
C	1.90970	−2.06360	0.97190
C	3.23870	0.00490	0.14490
C	5.22770	−1.23390	0.26990
C	1.72280	−3.09730	2.09670
Br	2.19950	−3.06770	−0.72830
C	4.73900	0.20770	0.09150
C	6.69250	−1.41110	0.71460
C	1.31290	−2.47750	3.41930
Cl	5.24840	1.26610	1.44090
C	7.13490	−2.88430	0.75690
C	8.62310	−3.01150	1.01520
O	6.42930	−3.53310	1.79200
C	9.45700	−3.58250	0.17620
C	0.12710	−2.67850	4.02020
C	−0.30400	−2.06980	5.34490
C	−0.33910	−3.13390	6.44460
O	0.57450	−1.03920	5.78030
C	10.28040	−4.14480	−0.67640
Br	11.18930	−3.12120	−2.10660
C	0.33580	0.22210	5.16960
H	2.98690	−0.61020	2.17480
H	0.98560	−1.49640	0.85010
H	2.88710	−0.33710	−0.82860
H	2.68970	0.91380	0.39280
H	5.07750	−1.75860	−0.67560
H	0.96280	−3.81920	1.79560
H	2.64290	−3.66420	2.24340
H	5.07740	0.66060	−0.84110
H	7.33050	−0.87110	0.01350
H	6.85670	−0.95130	1.68980
H	2.04440	−1.84030	3.89620
H	6.88100	−3.36730	−0.18950
H	8.98090	−2.60160	1.94840
H	5.53990	−3.18620	1.78180
H	−0.60100	−3.32390	3.54970
H	−1.31230	−1.66870	5.22560
H	−1.02070	−3.94560	6.18930
H	0.64980	−3.56360	6.60670
H	−0.67320	−2.70440	7.38920
H	10.53890	−5.19370	−0.68440
H	−0.65790	0.59650	5.41820
H	1.06500	0.94650	5.53240
H	0.42640	0.17220	4.08410

**Marilzafurollene C_171**			
C	3.63370	−0.77240	1.36380
O	4.76380	−1.63010	1.20020
C	2.39800	−1.58150	1.79940
C	3.47720	−0.01430	0.03910
C	5.32300	−1.45700	−0.10060
C	2.57390	−2.20090	3.19610
Br	2.04660	−3.03740	0.47700
C	4.88110	−0.05300	−0.52870
C	6.84030	−1.72940	−0.10060
C	1.31580	−2.88930	3.68890
Cl	5.86920	1.22860	0.23340
C	7.19110	−3.20150	0.21730
C	8.66150	−3.56970	0.06730
O	6.78750	−3.47640	1.54090
C	9.62040	−2.77060	−0.34690
C	0.62480	−2.52360	4.78130
C	−0.63480	−3.21310	5.27180
C	−0.44060	−3.76100	6.68790
O	−1.72920	−2.30930	5.31860
C	10.58840	−1.98790	−0.76220
Br	11.65530	−0.86580	0.47130
C	−2.23190	−1.93580	4.04290
H	3.88790	−0.05030	2.14160
H	1.52330	−0.92900	1.80300
H	2.80140	−0.54130	−0.63470
H	3.09210	0.99750	0.16830
H	4.83370	−2.17730	−0.75910
H	3.38890	−2.92570	3.18340
H	2.85960	−1.42640	3.90910
H	4.91070	0.09310	−1.60900
H	7.22380	−1.47160	−1.08870
H	7.34620	−1.07060	0.60670
H	0.97440	−3.73040	3.10170
H	6.62470	−3.85390	−0.44950
H	8.90120	−4.58770	0.33800
H	5.98640	−2.98020	1.69680
H	0.95990	−1.68370	5.37280
H	−0.87820	−4.04750	4.61090
H	0.37960	−4.47840	6.72350
H	−0.21900	−2.96100	7.39470
H	−1.34110	−4.26890	7.03350
H	10.89330	−1.87750	−1.79250
H	−2.57050	−2.80720	3.48110
H	−3.08440	−1.26850	4.16900
H	−1.48310	−1.40850	3.45080

**Marilzafurollene C_172**			
C	3.27850	−0.84200	0.88550
O	4.37500	−1.26680	1.69340
C	1.94340	−1.12360	1.60030
C	3.47630	−1.50950	−0.48170
C	5.27960	−2.05250	0.91890
C	1.70510	−2.60110	1.96520
Br	0.44710	−0.46440	0.45840
C	4.97970	−1.68140	−0.53890
C	6.72860	−1.86670	1.40900
C	0.47880	−2.78630	2.83750
Cl	5.74600	−0.13640	−1.01820
C	7.78670	−2.63440	0.59240
C	9.17970	−2.39800	1.14320
O	7.50180	−4.01770	0.63580
C	10.16120	−1.87790	0.44170
C	0.49950	−3.28550	4.08450
C	−0.72790	−3.46730	4.95790
C	−0.91450	−4.93850	5.33720
O	−0.61010	−2.73440	6.16860
C	11.14510	−1.34630	−0.24470
Br	11.47000	0.60550	−0.29800
C	−0.71440	−1.32590	6.01040
H	3.38510	0.23650	0.75830
H	1.92450	−0.52900	2.51500
H	3.00160	−2.48890	−0.51740
H	3.07670	−0.91770	−1.30560
H	4.98910	−3.09410	1.06820
H	1.57960	−3.20770	1.06910
H	2.57670	−2.99440	2.49030
H	5.29480	−2.45110	−1.24430
H	6.97450	−0.80420	1.40660
H	6.78050	−2.17630	2.45330
H	−0.46380	−2.48800	2.40020
H	7.75080	−2.30940	−0.44970
H	9.33770	−2.68030	2.17450
H	8.14380	−4.47770	0.11280
H	1.43900	−3.58450	4.52700
H	−1.61630	−3.13640	4.41620
H	−1.02850	−5.56240	4.45040
H	−0.06150	−5.31190	5.90420
H	−1.80480	−5.06990	5.95240
H	11.85850	−1.90280	−0.83500
H	−0.64690	−0.84440	6.98580
H	0.08780	−0.92740	5.38820
H	−1.67100	−1.04790	5.56640

**Marilzafurollene C_173**			
C	3.58570	−0.35670	1.26620
O	4.75380	−1.13640	1.00730
C	2.40810	−1.26280	1.67070
C	3.34910	0.48950	0.00850
C	5.26420	−0.83470	−0.29010
C	2.65920	−1.97630	3.00980
Br	2.09260	−2.63090	0.24930
C	4.73490	0.57060	−0.59790
C	6.79380	−1.01820	−0.34820
C	1.45440	−2.76690	3.48170
Cl	5.67780	1.84430	0.23240
C	7.23820	−2.47880	−0.15010
C	8.72410	−2.65400	−0.44610
O	6.90880	−2.86410	1.17290
C	9.61870	−3.14940	0.38140
C	0.78060	−2.52400	4.61820
C	−0.42570	−3.31550	5.08780
C	−0.15840	−3.96050	6.45000
O	−1.56260	−2.47760	5.23680
C	10.49880	−3.64320	1.21990
Br	10.94360	−5.57200	1.28840
C	−2.12460	−2.03370	4.00940
H	3.82510	0.31740	2.09030
H	1.50100	−0.66130	1.74840
H	2.68160	−0.02140	−0.68580
H	2.91540	1.46600	0.22540
H	4.79620	−1.53020	−0.98950
H	3.50980	−2.65310	2.91880
H	2.92670	−1.24400	3.77280
H	4.72310	0.79830	−1.66440
H	7.14100	−0.66910	−1.32150
H	7.28490	−0.38600	0.39260
H	1.13810	−3.57750	2.84000
H	6.68800	−3.12080	−0.84010
H	9.03600	−2.33650	−1.43040
H	6.07250	−2.45440	1.38280
H	1.09100	−1.71550	5.26440
H	−0.64760	−4.10790	4.37000
H	0.69780	−4.63410	6.40550
H	0.04500	−3.20730	7.21140
H	−1.02040	−4.54080	6.77960
H	11.05800	−3.06130	1.93740
H	−2.43670	−2.87580	3.39040
H	−3.00530	−1.42430	4.21190
H	−1.42310	−1.42320	3.43980

**Marilzafurollene C_174**			
C	3.46820	0.26420	1.12800
O	3.70090	−1.14080	1.00280
C	1.99230	0.54890	1.47350
C	3.93630	0.88290	−0.19890
C	4.64870	−1.39320	−0.02960
C	1.55340	0.05020	2.86610
Br	0.79150	−0.20430	0.06680
C	4.25500	−0.32870	−1.05430
C	6.08600	−1.27720	0.53370
C	1.50020	−1.46150	2.99460
Cl	5.50870	0.00400	−2.28120
C	6.38180	−2.26800	1.67310
C	7.85570	−2.28890	2.02630
O	5.64280	−1.87760	2.80950
C	8.60530	−3.36380	1.93850
C	2.23300	−2.18680	3.85600
C	2.15910	−3.69770	3.97740
C	3.50530	−4.34390	3.64390
O	1.81900	−4.09130	5.29910
C	9.34210	−4.44460	1.83810
Br	10.31310	−4.91700	0.17890
C	0.47230	−3.81780	5.66070
H	4.10330	0.64440	1.93010
H	1.84350	1.62970	1.45150
H	3.19470	1.53030	−0.66770
H	4.83400	1.47880	−0.03070
H	4.49690	−2.39450	−0.43460
H	2.21370	0.46100	3.63100
H	0.55570	0.43110	3.08570
H	3.35500	−0.65160	−1.58030
H	6.78620	−1.47430	−0.27930
H	6.29780	−0.26000	0.86470
H	0.81300	−1.96810	2.33210
H	6.05950	−3.26890	1.37670
H	8.27750	−1.35410	2.36550
H	4.74870	−1.71960	2.51640
H	2.92830	−1.69450	4.52090
H	1.41350	−4.08500	3.28020
H	3.80950	−4.11040	2.62330
H	4.29010	−3.99410	4.31540
H	3.44910	−5.42860	3.73370
H	9.49000	−5.16610	2.62840
H	0.28680	−4.17930	6.67210
H	0.25800	−2.74860	5.64690
H	−0.22660	−4.32250	4.99250

**Marilzafurollene C_175**			
C	3.08580	−0.51210	0.75440
O	4.15540	−1.40570	0.45230
C	1.75400	−1.03910	0.18470
C	3.54880	0.86090	0.24730
C	5.38330	−0.69110	0.51340
C	1.38180	−2.43220	0.72850
Br	1.87140	−1.15720	−1.80270
C	4.97980	0.60290	−0.20100
C	5.83130	−0.54810	1.99000
C	1.35550	−2.48200	2.24550
Cl	6.06490	1.98440	0.11990
C	6.08290	−1.90700	2.67110
C	7.41170	−2.51440	2.26440
O	6.06410	−1.73440	4.07220
C	7.52610	−3.64590	1.60750
C	2.29360	−3.09850	2.98430
C	2.33600	−3.12550	4.49620
C	2.34630	−4.57070	5.01170
O	3.51070	−2.44880	4.92930
C	7.65640	−4.76390	0.93360
Br	7.71160	−4.80870	−1.04450
C	3.41960	−1.91590	6.24500
H	2.99990	−0.44500	1.83940
H	0.95590	−0.33170	0.41380
H	2.94160	1.24940	−0.57040
H	3.51380	1.58720	1.05990
H	6.15140	−1.21870	−0.05350
H	0.40940	−2.74180	0.34510
H	2.10470	−3.16220	0.36090
H	4.98730	0.42420	−1.27750
H	6.73740	0.05540	2.04910
H	5.08250	0.00810	2.55290
H	0.53960	−1.96660	2.73170
H	5.27390	−2.59690	2.42360
H	8.29230	−1.95770	2.54950
H	5.19040	−1.96910	4.38330
H	3.10400	−3.60920	2.48410
H	1.44670	−2.61010	4.86510
H	2.31700	−4.61230	6.09990
H	1.48000	−5.12140	4.64370
H	3.24120	−5.09960	4.68280
H	7.74500	−5.74520	1.37640
H	2.61360	−1.18480	6.32000
H	3.25440	−2.69540	6.98890
H	4.35070	−1.40910	6.49930

**Marilzafurollene C_176**			
C	3.54050	−0.00630	0.67060
O	4.91180	−0.23200	0.99300
C	2.62520	−0.68180	1.70880
C	3.37030	−0.45590	−0.78630
C	5.62180	−0.67250	−0.16250
C	2.82420	−2.20220	1.85140
Br	0.72550	−0.29410	1.24250
C	4.75790	−0.23660	−1.35230
C	7.07470	−0.15850	−0.15060
C	2.07310	−2.77230	3.03870
Cl	4.97970	1.49270	−1.75630
C	7.93090	−0.77180	0.97260
C	8.18750	−2.25230	0.76190
O	9.17200	−0.09810	1.01420
C	7.75940	−3.18760	1.57880
C	2.65320	−3.40490	4.07360
C	1.92590	−3.98950	5.27340
C	2.20310	−3.16190	6.53080
O	0.51540	−4.02500	5.09220
C	7.31140	−4.12720	2.37680
Br	5.54870	−4.98700	2.11120
C	0.05850	−5.12830	4.32070
H	3.38270	1.07250	0.71650
H	2.81200	−0.21140	2.67550
H	3.11950	−1.51400	−0.84710
H	2.59460	0.09920	−1.31460
H	5.61910	−1.76350	−0.14150
H	2.49260	−2.72420	0.95460
H	3.88600	−2.42250	1.96710
H	4.94950	−0.82030	−2.25330
H	7.54120	−0.36000	−1.11590
H	7.06270	0.92600	−0.03900
H	0.99930	−2.64730	3.02680
H	7.43110	−0.61730	1.93200
H	8.75900	−2.51450	−0.11650
H	9.03640	0.76330	1.38380
H	3.72740	−3.52250	4.08330
H	2.30140	−5.00090	5.44050
H	3.27040	−3.11770	6.74880
H	1.84050	−2.14010	6.41540
H	1.70440	−3.59520	7.39790
H	7.84280	−4.51630	3.23320
H	−1.02300	−5.06610	4.20060
H	0.50480	−5.14330	3.32590
H	0.28510	−6.07330	4.81600

**Marilzafurollene C_177**			
C	3.41670	0.01020	0.54620
O	4.78940	−0.18790	0.88480
C	2.54560	−1.12540	1.11870
C	3.36480	0.15890	−0.98220
C	5.56690	−0.30920	−0.29970
C	2.62410	−1.19340	2.65350
Br	3.12420	−2.88080	0.36030
C	4.79430	0.51760	−1.33470
C	7.03560	0.09540	−0.06550
C	1.71510	−2.25630	3.23930
Cl	5.03060	2.27760	−1.11460
C	7.76180	−0.74820	0.99850
C	7.81970	−2.22490	0.65300
O	9.08030	−0.25210	1.11470
C	7.47160	−3.17600	1.49040
C	2.14330	−3.28410	3.99090
C	1.24820	−4.36190	4.57150
C	1.66920	−5.74370	4.06530
O	1.32690	−4.39150	5.98950
C	7.17340	−4.12240	2.34950
Br	5.35330	−4.87380	2.54200
C	0.71590	−3.28100	6.63170
H	3.11540	0.95720	0.99660
H	1.51030	−0.97210	0.81160
H	3.10400	−0.78400	−1.46210
H	2.64110	0.90400	−1.31330
H	5.52830	−1.35470	−0.61000
H	3.65430	−1.38130	2.96070
H	2.34260	−0.23150	3.08310
H	5.05800	0.26620	−2.36250
H	7.57580	0.03730	−1.01110
H	7.08030	1.13950	0.24430
H	0.66150	−2.16000	3.02080
H	7.25920	−0.61130	1.95910
H	8.16800	−2.47050	−0.33980
H	9.45880	−0.58570	1.91570
H	3.19710	−3.38140	4.21040
H	0.21490	−4.18340	4.26750
H	1.62250	−5.79390	2.97690
H	2.69050	−5.97940	4.36570
H	1.01670	−6.52030	4.46410
H	7.87930	−4.57940	3.02730
H	1.20470	−2.34220	6.36950
H	−0.34140	−3.20830	6.37430
H	0.78860	−3.40110	7.71260

**Marilzafurollene C_178**			
C	4.04450	−0.76510	1.52310
O	4.59990	−1.96520	0.97820
C	2.60930	−1.03130	2.01420
C	4.15580	0.29680	0.41890
C	5.38810	−1.66630	−0.17200
C	2.56550	−2.05290	3.16320
Br	1.49380	−1.69760	0.49770
C	4.60450	−0.50470	−0.78720
C	6.83750	−1.34300	0.26440
C	1.18150	−2.18950	3.76760
Cl	5.53630	0.47280	−1.95530
C	7.55470	−2.53010	0.93200
C	9.02440	−2.23650	1.15980
O	6.93880	−2.78910	2.17460
C	9.99480	−2.94150	0.62440
C	0.87990	−1.92730	5.05160
C	−0.49850	−2.06010	5.67970
C	−1.09040	−0.68190	5.98490
O	−1.41970	−2.73810	4.83420
C	10.95390	−3.64770	0.07440
Br	11.72070	−3.20000	−1.69490
C	−1.30440	−4.15470	4.86960
H	4.66250	−0.45250	2.36660
H	2.16460	−0.09370	2.35220
H	3.22650	0.83610	0.23320
H	4.91520	1.03100	0.69040
H	5.39650	−2.52140	−0.84910
H	2.89160	−3.03050	2.80610
H	3.26740	−1.75850	3.94450
H	3.73170	−0.89770	−1.31100
H	7.40870	−1.06190	−0.62140
H	6.86300	−0.47340	0.92180
H	0.40130	−2.52220	3.09680
H	7.44900	−3.41670	0.30260
H	9.24860	−1.39760	1.80230
H	5.99720	−2.75230	2.02800
H	1.66000	−1.58980	5.71900
H	−0.39460	−2.60310	6.62110
H	−0.44730	−0.11410	6.65770
H	−1.22030	−0.09980	5.07210
H	−2.06700	−0.77620	6.46010
H	11.41000	−4.52050	0.51840
H	−0.30800	−4.49040	4.58060
H	−1.52480	−4.54110	5.86540
H	−2.01950	−4.59590	4.17540

**Marilzafurollene C_179**			
C	3.36230	0.24540	1.17120
O	3.60380	−1.16160	1.09730
C	1.88020	0.51540	1.50360
C	3.82560	0.81840	−0.17690
C	4.52110	−1.45980	0.05080
C	1.47860	0.17460	2.95360
Br	0.70210	−0.46520	0.22510
C	4.13010	−0.41870	−0.99860
C	5.96410	−1.36760	0.59940
C	1.63700	−1.29120	3.31410
Cl	5.37770	−0.13050	−2.24270
C	6.25450	−2.39070	1.71300
C	7.72560	−2.41760	2.07650
O	5.50480	−2.04370	2.85690
C	8.47950	−3.48700	1.96150
C	2.46190	−1.75550	4.26920
C	2.64710	−3.21540	4.65160
C	2.08890	−3.48190	6.05150
O	1.98570	−4.10560	3.76030
C	9.22240	−4.56050	1.83180
Br	10.20580	−4.97540	0.16460
C	2.71210	−4.37900	2.56940
H	3.99110	0.66570	1.95800
H	1.67890	1.57700	1.35160
H	3.08600	1.45790	−0.65950
H	4.73030	1.41000	−0.03240
H	4.34020	−2.46770	−0.32480
H	2.06530	0.78060	3.64510
H	0.43570	0.44850	3.11600
H	3.22400	−0.74810	−1.50990
H	6.65670	−1.55530	−0.22210
H	6.18590	−0.35910	0.95030
H	1.03410	−1.98680	2.74750
H	5.93840	−3.38290	1.38290
H	8.14200	−1.49230	2.44710
H	4.63310	−1.79760	2.55410
H	3.06240	−1.05570	4.83260
H	3.71710	−3.43200	4.66510
H	2.57580	−2.85400	6.79790
H	1.01750	−3.28350	6.09090
H	2.24520	−4.52160	6.33920
H	9.36900	−5.30600	2.59960
H	2.14140	−5.06490	1.94370
H	2.89550	−3.47620	1.98880
H	3.67150	−4.84810	2.79100

**Marilzafurollene C_180**			
C	3.24960	0.08330	0.42010
O	4.39670	−0.33440	1.15630
C	1.92970	−0.36690	1.08290
C	3.49450	−0.38360	−1.01830
C	5.42580	−0.76100	0.26320
C	1.73040	−1.88160	1.29370
Br	0.41740	0.31180	−0.02290
C	5.00110	−0.27020	−1.12710
C	6.80850	−0.29690	0.76200
C	2.62600	−2.45600	2.37450
Cl	5.46500	1.43380	−1.41910
C	7.28000	−1.05180	2.01830
C	7.67360	−2.48280	1.70410
O	8.39940	−0.38370	2.56230
C	6.99020	−3.53150	2.10100
C	2.19850	−2.84290	3.58790
C	3.08900	−3.41570	4.67400
C	3.16000	−2.46770	5.87370
O	2.58330	−4.65290	5.15350
C	6.28000	−4.58040	2.43950
Br	4.84250	−5.28470	1.27600
C	2.72960	−5.73730	4.24780
H	3.26680	1.17450	0.42310
H	1.84300	0.13180	2.04950
H	3.19650	−1.42290	−1.15070
H	2.96290	0.21490	−1.75870
H	5.38970	−1.85100	0.23990
H	0.69490	−2.05600	1.58820
H	1.87470	−2.43840	0.36870
H	5.41750	−0.87380	−1.93430
H	7.54710	−0.41620	−0.03190
H	6.76020	0.77180	0.97350
H	3.67420	−2.54450	2.12810
H	6.48430	−1.04120	2.76710
H	8.57200	−2.60840	1.11740
H	8.10780	0.42940	2.95070
H	1.15190	−2.75280	3.84110
H	4.09930	−3.55640	4.28530
H	2.17450	−2.31190	6.31330
H	3.80950	−2.87250	6.64990
H	3.55640	−1.49450	5.58270
H	6.40070	−5.14500	3.35260
H	3.78180	−5.93350	4.04070
H	2.30430	−6.63910	4.68790
H	2.21500	−5.55380	3.30390

**Marilzafurollene C_181**			
C	3.99770	−0.10610	1.34600
O	5.03690	−1.08670	1.32720
C	2.83730	−0.61210	2.22320
C	3.65120	0.17210	−0.12580
C	5.64600	−1.13510	0.03980
C	2.19080	−1.92070	1.73120
Br	1.45350	0.81950	2.34220
C	4.44000	−0.88690	−0.86890
C	6.77710	−0.08090	−0.03950
C	1.15460	−2.45280	2.70170
Cl	4.85900	−0.39640	−2.53350
C	7.93770	−0.35080	0.93490
C	8.71500	−1.60130	0.56860
O	8.81080	0.75950	0.89720
C	8.82060	−2.64780	1.35530
C	1.23890	−3.63480	3.33440
C	0.20170	−4.16410	4.30730
C	−0.38430	−5.48880	3.81250
O	0.77290	−4.40890	5.58450
C	8.90700	−3.70740	2.12370
Br	7.59950	−5.18950	2.03140
C	1.10700	−3.23310	6.30960
H	4.40410	0.80420	1.78980
H	3.21800	−0.76260	3.23480
H	2.58500	0.13310	−0.34830
H	4.00250	1.16760	−0.39940
H	6.05470	−2.12950	−0.14230
H	1.71120	−1.77360	0.76370
H	2.96270	−2.67780	1.58570
H	3.84950	−1.80140	−0.93720
H	7.17360	−0.04010	−1.05440
H	6.36960	0.91070	0.15810
H	0.29850	−1.81660	2.87810
H	7.54060	−0.43560	1.94940
H	9.19230	−1.59740	−0.40100
H	9.40260	0.70490	1.63440
H	2.09250	−4.27550	3.16400
H	−0.61260	−3.44300	4.40440
H	−0.85670	−5.37060	2.83690
H	0.38760	−6.25360	3.72220
H	−1.14010	−5.86050	4.50450
H	9.67680	−3.87540	2.86240
H	1.50690	−3.50870	7.28540
H	1.86580	−2.64200	5.79560
H	0.22900	−2.60680	6.47330

**Marilzafurollene C_182**			
C	3.12760	−0.53680	0.99140
O	4.23340	−1.43580	1.08380
C	1.87910	−1.26480	0.45210
C	3.60770	0.64330	0.13650
C	5.35610	−0.90340	0.38400
C	1.37040	−2.37500	1.38760
Br	2.30310	−2.10110	−1.30940
C	5.10830	0.60880	0.34140
C	6.68050	−1.36420	1.02330
C	0.86180	−1.84620	2.71520
Cl	5.51970	1.40650	1.88910
C	6.89810	−2.88450	0.92860
C	8.28380	−3.27950	1.39920
O	5.93530	−3.52370	1.73800
C	9.16530	−3.87860	0.63150
C	1.40840	−2.14970	3.90660
C	0.95400	−1.64980	5.27360
C	−0.14470	−0.57900	5.21510
O	0.44040	−2.70970	6.06890
C	10.04010	−4.46600	−0.15010
Br	11.32310	−3.44670	−1.26100
C	1.41900	−3.61700	6.55770
H	2.93300	−0.16420	1.99810
H	1.07960	−0.54390	0.27530
H	3.38470	0.47860	−0.91780
H	3.15490	1.59220	0.42520
H	5.30480	−1.28080	−0.63910
H	0.55080	−2.91060	0.90780
H	2.16080	−3.10840	1.55440
H	5.66100	1.10890	−0.45480
H	7.50130	−0.85730	0.51360
H	6.73290	−1.05190	2.06690
H	0.00600	−1.19010	2.65870
H	6.75140	−3.20900	−0.10420
H	8.52380	−3.04170	2.42520
H	5.11910	−3.04040	1.62940
H	2.26530	−2.80750	3.92060
H	1.82030	−1.20840	5.76980
H	0.17470	0.28870	4.63730
H	−1.06040	−0.97050	4.77160
H	−0.39260	−0.23040	6.21780
H	10.14630	−5.53500	−0.26360
H	0.93940	−4.35190	7.20430
H	1.91000	−4.16140	5.75100
H	2.17890	−3.10180	7.14670

**Marilzafurollene C_183**			
C	3.31320	−1.16760	0.86140
O	4.58370	−1.55360	1.38410
C	2.60410	−2.35860	0.18670
C	3.58190	0.03380	−0.05040
C	5.58070	−0.60820	1.00450
C	2.27100	−3.49890	1.16390
Br	3.76860	−3.10540	−1.24970
C	4.80440	0.65060	0.59580
C	6.62170	−0.42810	2.12640
C	1.26980	−3.09340	2.22920
Cl	4.30330	1.63160	2.00710
C	7.49940	−1.67240	2.35150
C	8.47480	−1.90540	1.21310
O	8.23360	−1.49550	3.54590
C	8.46360	−2.97550	0.45160
C	1.52520	−3.09770	3.54820
C	0.53380	−2.68500	4.62000
C	0.21140	−3.86060	5.54580
O	1.05540	−1.64450	5.43430
C	8.45790	−4.03100	−0.32710
Br	7.42970	−4.09680	−2.01630
C	1.18330	−0.39090	4.77710
H	2.71530	−0.81670	1.70290
H	1.68910	−2.01500	−0.29740
H	3.83130	−0.28960	−1.06110
H	2.73620	0.71850	−0.11810
H	6.06710	−0.99990	0.10950
H	1.85130	−4.34120	0.61340
H	3.18760	−3.86130	1.63180
H	5.36740	1.29450	−0.08060
H	7.25650	0.43080	1.90470
H	6.09730	−0.18780	3.05160
H	0.29600	−2.78440	1.87710
H	6.85920	−2.54930	2.47540
H	9.20870	−1.12950	1.05160
H	7.64460	−1.60160	4.27970
H	2.49880	−3.40590	3.90220
H	−0.39390	−2.34980	4.15200
H	−0.21220	−4.69640	4.98840
H	1.10550	−4.21660	6.05820
H	−0.51230	−3.56770	6.30650
H	8.99980	−4.94490	−0.13250
H	0.22650	−0.05240	4.37770
H	1.53210	0.35810	5.48800
H	1.90730	−0.43310	3.96310

**Marilzafurollene C_184**			
C	4.05170	−0.61490	1.60150
O	4.63370	−1.85120	1.17860
C	2.66060	−0.87400	2.20950
C	4.04900	0.29970	0.36750
C	5.33790	−1.67170	−0.04840
C	2.73010	−1.73810	3.48020
Br	1.49600	−1.78090	0.86420
C	4.46460	−0.63190	−0.75390
C	6.79300	−1.23640	0.24990
C	1.37800	−1.89480	4.14770
Cl	5.27800	0.22340	−2.09370
C	7.60490	−2.29830	1.01310
C	9.06950	−1.91840	1.10730
O	7.07640	−2.42140	2.31540
C	10.03970	−2.64550	0.60190
C	0.77110	−3.07310	4.37220
C	−0.58980	−3.25020	5.02500
C	−0.44390	−3.79970	6.44610
O	−1.31220	−2.02750	5.11200
C	10.99790	−3.37570	0.08250
Br	11.63580	−3.12440	−1.77470
C	−1.95120	−1.64810	3.89940
H	4.69970	−0.17140	2.35950
H	2.18960	0.08090	2.44790
H	3.08630	0.77380	0.17440
H	4.78760	1.09180	0.49570
H	5.34810	−2.60600	−0.61110
H	3.14280	−2.71910	3.24070
H	3.40880	−1.28280	4.20210
H	3.58270	−1.12430	−1.16670
H	7.29690	−1.04790	−0.69890
H	6.81480	−0.28960	0.79050
H	0.88820	−0.98180	4.45500
H	7.50570	−3.26160	0.50750
H	9.29010	−0.99630	1.62490
H	6.12710	−2.44020	2.22660
H	1.26500	−3.98620	4.07200
H	−1.15960	−3.97140	4.43590
H	0.07800	−4.75680	6.44900
H	0.11540	−3.10960	7.07830
H	−1.42100	−3.95270	6.90470
H	11.52160	−4.16580	0.60060
H	−2.45650	−0.69220	4.03710
H	−1.23990	−1.53460	3.08040
H	−2.70220	−2.38240	3.60560

**Marilzafurollene C_185**			
C	3.40170	−0.42900	1.03910
O	4.80210	−0.66380	1.18690
C	2.59160	−1.54590	1.72420
C	3.14900	−0.27670	−0.46750
C	5.41910	−0.72600	−0.09430
C	2.78670	−1.54820	3.24960
Br	3.13770	−3.32370	0.99250
C	4.51320	0.12320	−0.99250
C	6.90520	−0.32080	−0.03390
C	1.89510	−2.55650	3.94830
Cl	4.75670	1.87800	−0.74250
C	7.76540	−1.20040	0.89440
C	7.74580	−2.67740	0.50880
O	9.08130	−0.66980	0.89430
C	8.77580	−3.37220	0.07720
C	0.94550	−2.24180	4.84490
C	0.05310	−3.25210	5.54170
C	0.23350	−3.18360	7.06020
O	−1.31770	−2.99850	5.27080
C	9.81690	−4.05170	−0.34180
Br	11.03090	−5.00360	0.89900
C	−1.71760	−3.31250	3.94370
H	3.18370	0.52580	1.52020
H	1.53260	−1.41320	1.49730
H	2.85660	−1.22850	−0.91150
H	2.36860	0.44870	−0.69890
H	5.34110	−1.75620	−0.44370
H	3.82680	−1.77040	3.49170
H	2.58210	−0.55370	3.64830
H	4.64430	−0.09020	−2.05390
H	7.32160	−0.34000	−1.04160
H	6.98530	0.71150	0.30700
H	2.05740	−3.59330	3.68870
H	7.37610	−1.12320	1.91130
H	6.78930	−3.16850	0.62250
H	9.58500	−1.11430	1.56160
H	0.77760	−1.20750	5.10890
H	0.31050	−4.26010	5.21010
H	1.26690	−3.38520	7.34350
H	−0.03580	−2.20040	7.44670
H	−0.39700	−3.91990	7.55870
H	10.10910	−4.15810	−1.37610
H	−2.78400	−3.11790	3.82950
H	−1.18940	−2.70610	3.20710
H	−1.54440	−4.36530	3.71760

**Marilzafurollene C_186**			
C	3.78360	−1.07500	1.29480
O	5.14900	−1.48620	1.36200
C	2.93960	−1.88930	2.29130
C	3.38980	−1.24610	−0.17630
C	5.68680	−1.66840	0.05240
C	1.45900	−1.46840	2.31430
Br	3.70460	−1.62720	4.11480
C	4.69610	−0.98000	−0.89480
C	7.15120	−1.19360	−0.02110
C	0.62430	−2.32940	3.24280
Cl	4.96940	0.78480	−1.00680
C	8.10060	−2.01920	0.86490
C	9.55140	−1.66550	0.60390
O	7.79930	−1.75620	2.21820
C	10.44070	−2.52990	0.17120
C	−0.07510	−1.86000	4.28930
C	−0.90730	−2.71810	5.22360
C	−0.39300	−2.61300	6.66140
O	−2.26520	−2.30220	5.23240
C	11.31540	−3.40150	−0.27200
Br	11.62750	−3.70340	−2.20350
C	−2.97680	−2.60510	4.04020
H	3.74880	−0.01650	1.55840
H	3.02810	−2.95210	2.06310
H	3.06950	−2.27110	−0.36790
H	2.58560	−0.57790	−0.48590
H	5.65130	−2.73980	−0.15340
H	1.37540	−0.41990	2.60390
H	1.02850	−1.55130	1.31610
H	4.72090	−1.38850	−1.90580
H	7.48220	−1.26840	−1.05800
H	7.22800	−0.13890	0.24620
H	0.60890	−3.38820	3.02740
H	7.94110	−3.08320	0.67600
H	9.83310	−0.64050	0.79610
H	6.85020	−1.68090	2.29140
H	−0.06300	−0.80210	4.50980
H	−0.84770	−3.76310	4.91300
H	0.64790	−2.93050	6.73060
H	−0.45650	−1.58890	7.03010
H	−0.97850	−3.24380	7.33030
H	11.93640	−4.02530	0.35420
H	−4.01310	−2.28350	4.14370
H	−2.55560	−2.09150	3.17540
H	−2.97850	−3.67790	3.84280

**Marilzafurollene C_187**			
C	3.72320	−1.04770	0.86990
O	4.73440	−0.98650	1.87520
C	2.52120	−1.88230	1.35060
C	4.41230	−1.57280	−0.39450
C	5.98480	−1.42180	1.34820
C	1.80830	−1.23140	2.54810
Br	3.13120	−3.71100	1.87510
C	5.85680	−1.18190	−0.16390
C	7.14760	−0.73820	2.10080
C	0.54910	−1.97420	2.95160
Cl	6.08410	0.53840	−0.60230
C	8.54380	−1.34080	1.84050
C	9.07650	−1.01960	0.45760
O	9.43290	−0.81070	2.80150
C	9.43330	−1.93630	−0.41280
C	0.34610	−2.52150	4.16160
C	−0.90940	−3.26930	4.56920
C	−0.58080	−4.71330	4.95600
O	−1.52780	−2.66380	5.69530
C	9.74310	−2.85730	−1.29380
Br	8.40630	−3.59680	−2.55160
C	−2.14620	−1.41420	5.42060
H	3.39910	−0.02300	0.68060
H	1.81620	−2.00210	0.52690
H	4.33960	−2.65870	−0.45740
H	3.99490	−1.15980	−1.31310
H	6.03630	−2.49780	1.52420
H	2.49300	−1.16890	3.39540
H	1.52760	−0.20720	2.30080
H	6.54680	−1.78000	−0.75820
H	7.15190	0.33650	1.91890
H	6.93480	−0.84390	3.16550
H	−0.21990	−2.05560	2.19700
H	8.50050	−2.42250	1.98750
H	9.14740	0.02980	0.20750
H	10.24300	−1.30000	2.77310
H	1.11320	−2.44250	4.91870
H	−1.61220	−3.28870	3.73390
H	−0.11120	−5.24480	4.12770
H	0.10030	−4.74920	5.80670
H	−1.48470	−5.25700	5.23080
H	10.72380	−3.29300	−1.41890
H	−1.42230	−0.66710	5.09330
H	−2.91590	−1.51410	4.65430
H	−2.62350	−1.03810	6.32550

**Marilzafurollene C_188**			
C	3.60320	−0.65910	0.84320
O	4.58110	−0.57650	1.87810
C	2.27240	−1.19930	1.39980
C	4.25350	−1.47020	−0.28290
C	5.81450	−1.14990	1.44700
C	2.36380	−2.58840	2.05910
Br	0.92710	−1.24290	−0.07180
C	5.72270	−1.16180	−0.08810
C	7.00500	−0.41550	2.10340
C	1.07660	−2.97690	2.76000
Cl	6.10020	0.43220	−0.80950
C	8.36380	−1.13870	1.99680
C	8.92930	−1.13060	0.59010
O	9.27400	−0.48450	2.85590
C	9.22490	−2.22310	−0.07650
C	0.96850	−3.21440	4.07780
C	−0.32100	−3.59960	4.77850
C	−0.18700	−4.96610	5.45530
O	−0.65530	−2.66180	5.79120
C	9.46760	−3.32200	−0.75030
Br	8.07950	−4.20130	−1.85310
C	−1.09360	−1.40270	5.29890
H	3.44260	0.35860	0.48370
H	1.90580	−0.48380	2.13770
H	4.08950	−2.53850	−0.14970
H	3.88210	−1.19900	−1.27170
H	5.80500	−2.18350	1.79730
H	2.59280	−3.35500	1.31980
H	3.17900	−2.59750	2.78390
H	6.36660	−1.90640	−0.55510
H	7.08330	0.60760	1.73570
H	6.76710	−0.31650	3.16330
H	0.20110	−3.06150	2.13140
H	8.25130	−2.16580	2.35180
H	9.07700	−0.15790	0.14160
H	10.05960	−1.00810	2.92810
H	1.83980	−3.13040	4.71150
H	−1.13260	−3.65470	4.05020
H	0.05470	−5.74300	4.72950
H	0.59720	−4.95710	6.21270
H	−1.11860	−5.24830	5.94590
H	10.41380	−3.84310	−0.77060
H	−0.31480	−0.89790	4.72630
H	−1.97620	−1.51100	4.66730
H	−1.35960	−0.75750	6.13610

**Marilzafurollene C_189**			
C	3.43330	−1.25420	0.82370
O	4.77980	−1.36340	1.28410
C	2.73190	−2.62780	0.83750
C	3.50910	−0.57560	−0.54900
C	5.66560	−0.71790	0.37290
C	2.61080	−3.25020	2.23970
Br	3.74940	−3.90860	−0.30380
C	4.77850	0.24130	−0.43100
C	6.85080	−0.07680	1.12050
C	1.72470	−2.45140	3.17710
Cl	4.43430	1.75660	0.45740
C	7.81570	−1.10640	1.73560
C	8.62410	−1.84210	0.68340
O	8.70760	−0.42820	2.59660
C	8.56970	−3.14130	0.49820
C	0.54560	−2.87890	3.65830
C	−0.33930	−2.08200	4.59880
C	−1.70530	−1.81100	3.96320
O	−0.57320	−2.78920	5.80780
C	8.52880	−4.43650	0.29450
Br	7.27880	−5.27010	−0.99290
C	0.55850	−2.87370	6.66330
H	2.91940	−0.57060	1.50000
H	1.73740	−2.53650	0.39830
H	3.62670	−1.31050	−1.34540
H	2.62860	0.02470	−0.77910
H	6.03250	−1.48240	−0.31420
H	2.21020	−4.26050	2.14910
H	3.59900	−3.35120	2.69040
H	5.20820	0.50630	−1.39760
H	7.40060	0.57870	0.44380
H	6.45810	0.56350	1.91080
H	2.09180	−1.47660	3.46530
H	7.24770	−1.82310	2.33390
H	9.27420	−1.23210	0.07350
H	8.24050	−0.18510	3.38370
H	0.17330	−3.85390	3.37780
H	0.13210	−1.12290	4.82260
H	−1.60310	−1.25240	3.03250
H	−2.23010	−2.74070	3.74210
H	−2.33520	−1.22630	4.63370
H	9.15730	−5.15970	0.79320
H	1.37820	−3.42230	6.19820
H	0.91670	−1.88230	6.94370
H	0.28470	−3.40020	7.57760

**Marilzafurollene C_190**			
C	3.37120	−2.44550	1.63620
O	4.73800	−2.66970	1.29270
C	2.60330	−3.78090	1.65020
C	2.87010	−1.42390	0.61130
C	5.18980	−1.68740	0.35980
C	1.10370	−3.64940	1.98060
Br	3.45280	−4.99770	2.98230
C	4.11100	−0.59570	0.35330
C	6.62680	−1.23200	0.68100
C	0.84230	−2.98170	3.31790
Cl	4.32470	0.58920	1.67720
C	7.66600	−2.35740	0.53490
C	9.08070	−1.82500	0.64950
O	7.44840	−3.30850	1.55430
C	9.97070	−1.92170	−0.31170
C	0.16700	−1.83120	3.47530
C	−0.08340	−1.15880	4.81170
C	0.54530	0.23640	4.84320
O	−1.47360	−0.99890	5.05370
C	10.84570	−2.01540	−1.28470
Br	11.03590	−0.62240	−2.67850
C	−2.15750	−2.21000	5.34530
H	3.35910	−1.99490	2.63000
H	2.71300	−4.26860	0.68080
H	2.56460	−1.92500	−0.30810
H	2.02650	−0.83470	0.97220
H	5.18150	−2.16490	−0.62150
H	0.59880	−3.09000	1.19220
H	0.64390	−4.63810	1.99520
H	4.07080	−0.04430	−0.58680
H	6.88770	−0.42060	−0.00010
H	6.68200	−0.81310	1.68670
H	1.24220	−3.48640	4.18640
H	7.53490	−2.85030	−0.43110
H	9.33540	−1.34880	1.58510
H	6.50440	−3.39350	1.67000
H	−0.23770	−1.32280	2.61220
H	0.36110	−1.75410	5.61190
H	1.62160	0.18700	4.67450
H	0.11480	0.88210	4.07740
H	0.38220	0.71360	5.80970
H	11.53170	−2.83870	−1.42040
H	−3.20670	−1.99540	5.54810
H	−2.11810	−2.90910	4.50930
H	−1.74000	−2.69700	6.22750

**Marilzafurollene C_191**			
C	3.35890	−0.17730	0.75940
O	4.58640	−0.79730	1.14260
C	2.24820	−0.49900	1.77720
C	3.10420	−0.61170	−0.68940
C	5.18580	−1.43770	0.01490
C	1.96270	−2.00050	1.96850
Br	0.57620	0.43430	1.22100
C	4.51060	−0.81420	−1.21360
C	6.72360	−1.34670	0.07050
C	1.03650	−2.26510	3.13930
Cl	5.22460	0.76690	−1.65220
C	7.33460	−2.11900	1.25280
C	8.84770	−2.15810	1.16900
O	6.95480	−1.47800	2.45090
C	9.53900	−3.27150	1.08000
C	1.36670	−2.98590	4.22530
C	0.45500	−3.26620	5.40930
C	0.90930	−2.47990	6.64160
O	−0.89710	−2.90640	5.15260
C	10.21590	−4.39070	0.97860
Br	10.65830	−5.20830	−0.76930
C	−1.62280	−3.87510	4.40670
H	3.53670	0.89940	0.76730
H	2.53850	−0.07060	2.73810
H	2.56440	−1.55680	−0.73050
H	2.53210	0.12160	−1.25870
H	4.89150	−2.48800	0.05640
H	1.51140	−2.42640	1.07310
H	2.89940	−2.53470	2.13260
H	4.55060	−1.45680	−2.09390
H	7.12380	−1.75350	−0.85940
H	7.04540	−0.30520	0.10800
H	0.04860	−1.83070	3.07090
H	6.93890	−3.13710	1.26510
H	9.35060	−1.20220	1.18720
H	6.06920	−1.14670	2.32370
H	2.35720	−3.41270	4.29460
H	0.51450	−4.33100	5.64250
H	1.93430	−2.73020	6.91590
H	0.86210	−1.40570	6.46080
H	0.27280	−2.70050	7.49860
H	10.59000	−4.96370	1.81450
H	−1.16210	−4.07320	3.43860
H	−1.69780	−4.81560	4.95390
H	−2.63500	−3.51290	4.22690

**Marilzafurollene C_192**			
C	3.56280	−2.25620	1.44010
O	4.75570	−2.29190	2.22250
C	2.67150	−3.47890	1.74220
C	4.01560	−2.12780	−0.01870
C	5.90260	−2.18050	1.38410
C	2.16830	−3.53650	3.19560
Br	3.69470	−5.15630	1.39180
C	5.37720	−1.48080	0.12110
C	7.06530	−1.51890	2.15630
C	1.23850	−2.39290	3.55540
Cl	5.17640	0.28260	0.35290
C	8.44590	−1.61430	1.47430
C	8.56710	−0.71550	0.25910
O	9.42490	−1.23240	2.41820
C	8.86830	−1.15600	−0.94110
C	−0.06640	−2.53420	3.84170
C	−0.99550	−1.39090	4.20570
C	−2.14160	−1.27800	3.19730
O	−1.58080	−1.59100	5.48390
C	9.12280	−1.60890	−2.14550
Br	7.68360	−2.19420	−3.37120
C	−0.67800	−1.43400	6.57040
H	3.03600	−1.33870	1.70360
H	1.81520	−3.48480	1.06610
H	4.12920	−3.10730	−0.48310
H	3.32420	−1.54970	−0.63220
H	6.19110	−3.19950	1.11960
H	1.64640	−4.48050	3.35630
H	3.01400	−3.53190	3.88450
H	6.00330	−1.64110	−0.75610
H	6.82950	−0.48440	2.40560
H	7.13430	−2.03240	3.11620
H	1.68210	−1.40760	3.58010
H	8.63780	−2.65340	1.19710
H	8.38360	0.33680	0.42590
H	10.28080	−1.45460	2.07970
H	−0.51660	−3.51650	3.82310
H	−0.44030	−0.45070	4.20180
H	−1.76400	−1.10550	2.18910
H	−2.74440	−2.18630	3.18030
H	−2.80050	−0.44760	3.45140
H	10.10830	−1.70730	−2.57730
H	0.12720	−2.16870	6.53770
H	−0.23940	−0.43550	6.57860
H	−1.21280	−1.57100	7.51020

**Marilzafurollene C_193**			
C	2.91440	−0.71900	0.54450
O	3.64620	−1.89200	0.18090
C	1.40590	−0.95860	0.33290
C	3.51070	0.42660	−0.28720
C	4.89600	−1.52580	−0.39650
C	0.84810	−2.11030	1.18780
Br	1.06500	−1.39160	−1.58410
C	4.49960	−0.28150	−1.19410
C	5.94090	−1.30460	0.72440
C	0.97450	−1.85370	2.67750
Cl	5.87470	0.75800	−1.65930
C	6.22420	−2.56960	1.55510
C	7.38160	−2.36140	2.51160
O	5.06670	−2.89130	2.29500
C	8.48700	−3.06990	2.47500
C	1.77380	−2.56160	3.49650
C	1.96710	−2.35120	4.99380
C	1.23640	−1.12210	5.55260
O	1.50270	−3.46930	5.73810
C	9.59010	−3.77790	2.41990
Br	11.14320	−3.23890	1.31710
C	2.33000	−4.62210	5.65440
H	3.10960	−0.50900	1.59730
H	0.85380	−0.04330	0.55210
H	2.76840	0.99120	−0.85200
H	4.02820	1.12670	0.36960
H	5.24260	−2.31520	−1.06460
H	−0.20680	−2.26390	0.95870
H	1.35670	−3.03980	0.92820
H	3.99490	−0.59020	−2.11120
H	6.87710	−0.98720	0.26310
H	5.64330	−0.48650	1.38120
H	0.37470	−1.04360	3.06490
H	6.44590	−3.40120	0.88220
H	7.25590	−1.58230	3.24920
H	4.32710	−2.77470	1.70340
H	2.36130	−3.36180	3.07060
H	3.03510	−2.21160	5.17210
H	1.55620	−0.20720	5.05340
H	0.15600	−1.21610	5.44150
H	1.44540	−1.00410	6.61590
H	9.75860	−4.70070	2.95560
H	1.93030	−5.40180	6.30280
H	2.36820	−5.02500	4.64240
H	3.34720	−4.40540	5.98320

**Marilzafurollene C_194**			
C	2.99570	−0.78530	−0.27320
O	3.98740	−0.84810	0.75250
C	2.31860	−2.15510	−0.50160
C	3.70600	−0.22000	−1.51300
C	5.25900	−0.48900	0.23090
C	1.47270	−2.66160	0.68470
Br	3.67960	−3.53780	−0.97880
C	4.92770	0.45860	−0.92790
C	6.18420	0.07300	1.32770
C	2.28120	−2.89760	1.94570
Cl	4.47760	2.09660	−0.36560
C	6.45250	−0.90080	2.49000
C	7.12640	−2.18720	2.05070
O	7.28800	−0.23960	3.41880
C	6.72580	−3.37880	2.43330
C	1.94790	−2.46150	3.17080
C	2.79370	−2.69090	4.40860
C	3.07790	−1.37020	5.12830
O	2.13690	−3.54370	5.33460
C	6.36190	−4.56410	2.86340
Br	5.01900	−5.68370	1.93830
C	2.05950	−4.90050	4.91750
H	2.24590	−0.06440	0.05610
H	1.65870	−2.06890	−1.36590
H	4.02790	−1.02110	−2.17820
H	3.07670	0.45430	−2.09440
H	5.70500	−1.39200	−0.18920
H	0.66690	−1.95770	0.89590
H	0.99870	−3.60430	0.40990
H	5.74540	0.55870	−1.64230
H	7.12940	0.37620	0.87600
H	5.74430	0.97880	1.74480
H	3.19500	−3.45900	1.82060
H	5.50540	−1.13010	2.98350
H	7.97440	−2.08130	1.38970
H	7.28470	−0.72620	4.23060
H	1.03210	−1.90720	3.31510
H	3.74810	−3.13590	4.11960
H	3.58420	−0.66390	4.46980
H	2.15540	−0.90300	5.47370
H	3.71440	−1.53120	5.99830
H	6.75130	−5.04000	3.75150
H	1.58230	−5.49420	5.69710
H	1.46820	−5.01220	4.00810
H	3.05180	−5.31750	4.74030

**Marilzafurollene C_195**			
C	2.68100	−0.48820	0.64760
O	3.60900	−1.43690	0.12580
C	1.23600	−0.86110	0.26200
C	3.18280	0.88040	0.16720
C	4.89080	−0.82770	0.04220
C	0.82080	−2.25640	0.76810
Br	1.04790	−0.84060	−1.72330
C	4.50640	0.55070	−0.50660
C	5.57400	−0.84380	1.43380
C	1.00050	−2.42270	2.26680
Cl	5.74560	1.81020	−0.24920
C	5.75110	−2.25970	2.01820
C	6.80650	−3.07150	1.27280
O	6.03980	−2.14800	3.40200
C	7.90080	−3.57550	1.80050
C	1.99640	−3.13910	2.81480
C	2.24840	−3.30880	4.29940
C	2.39520	−4.78810	4.66310
O	3.45660	−2.65740	4.67500
C	8.99750	−4.06100	2.33250
Br	9.09260	−5.87920	3.11210
C	3.29930	−1.26350	4.91110
H	2.76780	−0.49990	1.73300
H	0.54870	−0.11000	0.65370
H	2.50000	1.37470	−0.52400
H	3.32710	1.54370	1.02070
H	5.51010	−1.36130	−0.67890
H	−0.21930	−2.45850	0.51140
H	1.41590	−3.01190	0.25280
H	4.34430	0.46660	−1.58250
H	6.55040	−0.36210	1.37170
H	4.99930	−0.23610	2.13220
H	0.28920	−1.91340	2.90140
H	4.81130	−2.80450	1.93600
H	6.60530	−3.23430	0.22430
H	5.27430	−2.44190	3.89000
H	2.69780	−3.63900	2.16270
H	1.40690	−2.90100	4.86360
H	1.49620	−5.34840	4.40510
H	3.23690	−5.24350	4.14060
H	2.56540	−4.90900	5.73310
H	9.93680	−3.53190	2.39470
H	2.93390	−0.74310	4.02660
H	2.60650	−1.07920	5.73320
H	4.25910	−0.82430	5.18330

**Marilzafurollene C_196**			
C	3.14600	−2.46630	1.04130
O	4.20130	−2.83880	1.92660
C	2.18380	−3.64720	0.80060
C	3.82520	−1.91520	−0.21640
C	5.46630	−2.50670	1.36050
C	1.44720	−4.09950	2.07320
Br	3.20340	−5.21110	0.09600
C	5.14570	−1.41420	0.32810
C	6.48150	−2.18150	2.47820
C	0.51980	−3.03670	2.63150
Cl	4.90580	0.19090	1.08350
C	7.95530	−2.11150	2.02750
C	8.26310	−0.87520	1.20520
O	8.76470	−2.09730	3.18500
C	8.75840	−0.91790	−0.01060
C	0.63360	−2.50250	3.85900
C	−0.28120	−1.43030	4.42080
C	−1.05070	−1.95240	5.63660
O	0.45510	−0.29420	4.85070
C	9.20890	−0.96890	−1.24140
Br	7.99300	−1.06420	−2.79980
C	1.05280	0.45310	3.79980
H	2.60760	−1.64480	1.51480
H	1.45470	−3.37630	0.03590
H	4.01150	−2.70810	−0.94100
H	3.24150	−1.14070	−0.71440
H	5.80450	−3.39640	0.82620
H	0.84880	−4.98410	1.85410
H	2.17280	−4.39690	2.83200
H	5.90350	−1.31640	−0.44870
H	6.19760	−1.27410	3.01120
H	6.39880	−2.98020	3.21650
H	−0.27480	−2.70450	1.97870
H	8.20000	−3.01310	1.46110
H	8.04510	0.07410	1.67440
H	9.66630	−2.23570	2.93100
H	1.42720	−2.83370	4.51390
H	−1.00250	−1.12810	3.65910
H	−1.65700	−2.82030	5.37620
H	−0.37170	−2.24460	6.43800
H	−1.71880	−1.18650	6.03070
H	10.25230	−0.96590	−1.52210
H	1.81200	−0.12550	3.27280
H	0.30720	0.79110	3.07910
H	1.54110	1.33520	4.21360

**Marilzafurollene C_197**			
C	2.95660	0.07420	0.18980
O	4.00500	−0.55340	0.92630
C	1.62140	0.07840	0.96760
C	2.95140	−0.60040	−1.18790
C	4.73340	−1.44430	0.07990
C	1.02580	−1.29170	1.35550
Br	0.27220	1.05050	−0.12950
C	4.40290	−1.00410	−1.35090
C	6.22370	−1.48050	0.46770
C	1.83330	−2.02560	2.41020
Cl	5.37340	0.40840	−1.86480
C	6.46730	−2.09170	1.85920
C	7.94700	−2.25510	2.14430
O	5.89980	−1.24140	2.83190
C	8.51340	−3.41550	2.38520
C	1.41130	−2.27600	3.66030
C	2.22950	−3.00550	4.70980
C	2.46550	−2.11580	5.93270
O	1.56000	−4.17300	5.16300
C	9.06760	−4.58270	2.61250
Br	9.79500	−5.71270	1.15890
C	1.52350	−5.22990	4.21400
H	3.26630	1.11140	0.05100
H	1.75270	0.66980	1.87530
H	2.32550	−1.49150	−1.18920
H	2.59510	0.05670	−1.98200
H	4.30860	−2.43960	0.22160
H	0.90430	−1.93350	0.48420
H	0.02100	−1.13290	1.74920
H	4.54700	−1.79710	−2.08570
H	6.75840	−2.07380	−0.27560
H	6.65510	−0.47950	0.42330
H	2.81530	−2.36310	2.11600
H	5.97050	−3.06270	1.92030
H	8.53560	−1.34920	2.14260
H	5.13940	−0.82410	2.43390
H	0.42960	−1.94970	3.97270
H	3.20120	−3.27960	4.29340
H	3.06410	−2.63640	6.68030
H	1.52420	−1.82860	6.40180
H	2.99800	−1.20470	5.65830
H	9.18000	−5.03310	3.58800
H	0.97340	−4.94870	3.31540
H	2.52960	−5.53690	3.92580
H	1.02350	−6.09410	4.65130

**Marilzafurollene C_198**			
C	4.22500	−0.78850	1.75910
O	4.98340	−2.00060	1.79020
C	3.17090	−0.82190	2.88180
C	3.67510	−0.66710	0.32860
C	5.28660	−2.42620	0.46220
C	2.15550	−1.97490	2.76780
Br	2.20180	0.92070	2.90200
C	4.01990	−2.00740	−0.28790
C	6.60020	−1.75870	−0.01220
C	1.23480	−2.05610	3.96970
Cl	4.20940	−1.92270	−2.06090
C	7.83340	−2.18170	0.80600
C	9.11750	−1.67390	0.18030
O	7.71630	−1.65300	2.10870
C	10.07930	−2.46340	−0.24020
C	−0.10320	−1.95150	3.90990
C	−1.02720	−2.02770	5.11080
C	−1.82640	−0.73140	5.26510
O	−1.96540	−3.08480	4.97200
C	11.02730	−3.26400	−0.66630
Br	11.06980	−3.96870	−2.51570
C	−1.40510	−4.38400	5.10470
H	4.90980	0.04110	1.94320
H	3.69380	−0.89510	3.83670
H	2.60940	−0.44410	0.27780
H	4.19770	0.13530	−0.19340
H	5.40330	−3.51040	0.43640
H	1.56230	−1.87340	1.85890
H	2.68390	−2.92590	2.69180
H	3.22350	−2.72280	−0.07830
H	6.77500	−2.04410	−1.05030
H	6.50880	−0.67200	−0.01550
H	1.71280	−2.20730	4.92710
H	7.86410	−3.27140	0.87690
H	9.21310	−0.60150	0.09260
H	6.81530	−1.79190	2.38590
H	−0.58640	−1.80000	2.95540
H	−0.43870	−2.17860	6.01800
H	−1.16440	0.12610	5.38920
H	−2.45390	−0.54750	4.39270
H	−2.47720	−0.77830	6.13840
H	11.85770	−3.61010	−0.06840
H	−2.19420	−5.13090	5.01770
H	−0.66820	−4.58890	4.32740
H	−0.93050	−4.51200	6.07840

**Marilzafurollene C_199**			
C	4.07390	−0.11160	1.39090
O	4.92020	−1.22370	1.09060
C	2.68290	−0.61010	1.82640
C	4.07040	0.77190	0.13420
C	5.74510	−0.92850	−0.03240
C	2.74080	−1.45010	3.11360
Br	1.86890	−1.70460	0.36790
C	4.79870	−0.08130	−0.88550
C	7.03430	−0.21880	0.44800
C	1.36370	−1.82440	3.62750
Cl	5.61040	0.89060	−2.14470
C	7.91870	−1.09860	1.34990
C	8.53570	−2.25990	0.59200
O	8.95350	−0.29070	1.87370
C	8.31420	−3.52100	0.88750
C	0.92970	−3.08600	3.78500
C	−0.44790	−3.46580	4.29440
C	−1.20910	−4.28220	3.24690
O	−0.36260	−4.26320	5.46680
C	8.07890	−4.78360	1.15870
Br	6.59340	−5.78880	0.32100
C	0.06530	−3.55720	6.62350
H	4.52670	0.45100	2.20900
H	2.02920	0.24920	1.98290
H	3.07240	1.05470	−0.20170
H	4.62680	1.68960	0.32790
H	6.00290	−1.84870	−0.55760
H	3.33250	−2.35050	2.94110
H	3.24990	−0.89040	3.89890
H	4.08680	−0.73840	−1.38750
H	7.62150	0.10420	−0.41210
H	6.77190	0.69240	0.98580
H	0.70670	−1.00310	3.87500
H	7.32060	−1.46820	2.18680
H	9.18550	−1.99850	−0.23150
H	9.37950	−0.76220	2.57560
H	1.58360	−3.91030	3.53790
H	−1.02170	−2.56210	4.50910
H	−1.32260	−3.72170	2.31840
H	−0.68840	−5.21190	3.01590
H	−2.20610	−4.53970	3.60450
H	8.65490	−5.38690	1.84530
H	0.07040	−4.23150	7.47980
H	1.07520	−3.16270	6.50690
H	−0.60820	−2.73060	6.85360

**Marilzafurollene C_200**			
C	4.19260	−1.14040	1.66720
O	4.32980	−1.79690	0.40390
C	2.71920	−1.17400	2.11390
C	4.77170	0.26990	1.47910
C	5.28160	−1.11100	−0.40650
C	2.21430	−2.60710	2.35240
Br	1.57790	−0.29430	0.73110
C	5.01780	0.34480	−0.01490
C	6.70260	−1.64470	−0.10110
C	0.81050	−2.63990	2.92470
Cl	6.33490	1.47220	−0.44200
C	6.89630	−3.13290	−0.47640
C	8.32070	−3.65700	−0.34650
O	6.05160	−3.91540	0.33880
C	9.36870	−2.95940	0.03380
C	0.48820	−3.15120	4.12450
C	−0.91710	−3.18160	4.69600
C	−1.36220	−4.61970	4.97330
O	−0.98540	−2.47710	5.92720
C	10.41340	−2.27040	0.42860
Br	10.94290	−2.13620	2.33150
C	−0.89080	−1.06520	5.79720
H	4.80430	−1.67090	2.39900
H	2.60960	−0.59860	3.03480
H	4.11320	1.06550	1.82890
H	5.71270	0.35640	2.02370
H	5.05460	−1.26890	−1.46160
H	2.22470	−3.16790	1.41690
H	2.88990	−3.12710	3.03290
H	4.11370	0.69270	−0.51710
H	7.41400	−1.05160	−0.67650
H	6.95840	−1.48750	0.94740
H	0.03450	−2.21660	2.30220
H	6.59190	−3.27760	−1.51440
H	8.44200	−4.70130	−0.59530
H	5.22700	−3.44190	0.41880
H	1.25860	−3.57580	4.75200
H	−1.61390	−2.73780	3.98220
H	−1.34920	−5.21860	4.06230
H	−0.71070	−5.10190	5.70250
H	−2.37730	−4.64130	5.37020
H	11.07310	−1.72010	−0.22620
H	0.06740	−0.76030	5.37520
H	−1.69000	−0.67200	5.16770
H	−0.98210	−0.60250	6.77990

**Marilzafurollene C_201**			
C	3.61860	−0.54180	1.43550
O	4.47670	−1.66270	1.64750
C	2.16330	−0.88350	1.80670
C	3.83290	−0.10980	−0.02010
C	5.23500	−1.92950	0.47000
C	2.00690	−1.19300	3.30510
Br	1.54550	−2.46260	0.75060
C	5.23710	−0.60300	−0.29970
C	6.60540	−2.53680	0.82760
C	0.56050	−1.40500	3.70940
Cl	6.42340	0.56340	0.36060
C	7.53530	−2.78560	−0.37640
C	8.85870	−3.37720	0.06890
O	6.91240	−3.67670	−1.27850
C	10.02210	−2.80960	−0.15590
C	0.08290	−2.53710	4.25260
C	−1.36340	−2.75610	4.65470
C	−1.97230	−3.92660	3.87870
O	−1.47330	−3.06640	6.03640
C	11.18850	−2.24500	−0.36220
Br	11.99380	−0.97110	0.92090
C	−1.21090	−1.97170	6.90360
H	3.97660	0.25990	2.08350
H	1.51950	−0.04520	1.53750
H	3.13470	−0.61830	−0.68530
H	3.71360	0.96330	−0.17110
H	4.66600	−2.65840	−0.11010
H	2.59980	−2.07180	3.56370
H	2.40220	−0.36750	3.89770
H	5.44010	−0.73280	−1.36330
H	7.11000	−1.88450	1.54110
H	6.43690	−3.47490	1.35760
H	−0.10990	−0.57530	3.53730
H	7.71860	−1.84330	−0.89710
H	8.80450	−4.31590	0.60180
H	7.47790	−3.79720	−2.02860
H	0.75050	−3.36910	4.42540
H	−1.94510	−1.85860	4.43530
H	−1.93190	−3.74860	2.80370
H	−1.44130	−4.85620	4.08490
H	−3.01710	−4.07280	4.15300
H	11.81130	−2.41190	−1.22900
H	−1.35040	−2.28580	7.93790
H	−0.18670	−1.61110	6.80250
H	−1.89360	−1.14290	6.71150

**Marilzafurollene C_202**			
C	4.08130	−0.38230	1.29690
O	5.49840	−0.54580	1.25580
C	3.45130	−1.58150	2.02820
C	3.64570	−0.22130	−0.16820
C	5.94860	−0.61080	−0.09080
C	1.92230	−1.47500	2.16590
Br	4.24870	−1.69280	3.85360
C	4.92010	0.22000	−0.86040
C	7.42320	−0.17710	−0.20780
C	1.31360	−2.70950	2.80220
Cl	5.15470	1.97750	−0.62420
C	8.38250	−1.08800	0.58160
C	8.39270	−2.51410	0.06300
O	9.68990	−0.55880	0.49860
C	7.88790	−3.52510	0.73290
C	0.58960	−2.70300	3.93370
C	−0.01390	−3.93780	4.57600
C	0.54110	−4.14260	5.98770
O	−1.42460	−3.82070	4.69230
C	7.32880	−4.50910	1.39590
Br	5.41880	−4.97880	1.16580
C	−2.12170	−3.91090	3.45720
H	3.87660	0.53880	1.84460
H	3.72140	−2.50410	1.51320
H	3.32940	−1.18140	−0.57830
H	2.82200	0.48200	−0.29400
H	5.84670	−1.64640	−0.41930
H	1.66200	−0.58860	2.74620
H	1.46300	−1.35190	1.18500
H	4.92100	0.01700	−1.93190
H	7.71370	−0.15810	−1.25890
H	7.52730	0.84460	0.15750
H	1.49170	−3.64560	2.29230
H	8.08600	−1.08830	1.63360
H	8.84210	−2.66700	−0.90720
H	9.74870	0.18940	1.07630
H	0.40910	−1.77000	4.44810
H	0.23130	−4.81880	3.97940
H	1.62590	−4.25280	5.97090
H	0.29960	−3.29830	6.63370
H	0.12230	−5.04010	6.44300
H	7.83430	−5.14110	2.11140
H	−1.85050	−3.10130	2.77880
H	−1.92560	−4.86250	2.96150
H	−3.19420	−3.84460	3.63950

**Marilzafurollene C_203**			
C	3.18870	−1.15730	0.57750
O	4.46440	−1.24500	1.21230
C	2.75450	−2.53070	0.02140
C	3.30380	−0.04150	−0.47310
C	5.39240	−0.40060	0.54470
C	2.64930	−3.61890	1.10370
Br	4.06260	−3.15970	−1.34860
C	4.52180	0.73280	−0.01110
C	6.56000	0.01940	1.45970
C	1.56940	−3.33310	2.12980
Cl	4.02920	1.89790	1.25460
C	7.32500	−1.15800	2.09240
C	7.86300	−2.14530	1.07310
O	8.40290	−0.62520	2.83560
C	7.67000	−3.44220	1.16130
C	1.79820	−3.16840	3.44320
C	0.72540	−2.87530	4.47520
C	0.62990	−4.00920	5.49900
O	1.01130	−1.68570	5.19690
C	7.50570	−4.73740	1.29560
Br	5.94700	−5.71070	0.56150
C	0.90450	−0.49320	4.43130
H	2.48130	−0.83260	1.34100
H	1.79540	−2.43080	−0.48810
H	3.49450	−0.44970	−1.46520
H	2.40560	0.57270	−0.54140
H	5.79130	−0.96230	−0.30140
H	2.42330	−4.57810	0.63710
H	3.61370	−3.73940	1.59980
H	5.00710	1.28820	−0.81430
H	7.24880	0.64130	0.88690
H	6.18900	0.64750	2.26930
H	0.55910	−3.26230	1.75300
H	6.65570	−1.67460	2.78480
H	8.42110	−1.72780	0.24780
H	8.72820	−1.29640	3.41850
H	2.80850	−3.23810	3.82120
H	−0.24220	−2.77540	3.97960
H	0.39340	−4.95750	5.01570
H	1.56820	−4.13240	6.04050
H	−0.15150	−3.80550	6.23130
H	8.19130	−5.39180	1.81370
H	1.64180	−0.46070	3.62870
H	−0.09060	−0.38790	3.99730
H	1.08120	0.36860	5.07470

**Marilzafurollene C_204**			
C	3.44010	−0.87130	1.64350
O	4.62140	−1.63800	1.87650
C	2.18160	−1.66590	2.03980
C	3.49690	−0.43570	0.17440
C	5.42530	−1.67060	0.69920
C	2.13500	−1.95920	3.54870
Br	2.12450	−3.39210	1.03560
C	4.98480	−0.44250	−0.10670
C	6.91970	−1.77160	1.06110
C	0.83860	−2.62220	3.97220
Cl	5.71820	1.07060	0.50810
C	7.88050	−1.71950	−0.14320
C	9.33440	−1.74680	0.31500
O	7.58060	−2.78360	−1.03170
C	10.22820	−2.65350	−0.01580
C	−0.04600	−2.08820	4.83080
C	−1.34340	−2.75290	5.25150
C	−1.37710	−2.97400	6.76580
O	−2.46410	−1.94570	4.92080
C	11.10810	−3.56220	−0.36420
Br	12.24230	−3.37500	−1.97570
C	−2.75050	−1.88750	3.53010
H	3.51260	0.02260	2.26510
H	1.29450	−1.09780	1.75590
H	3.00670	−1.16660	−0.46890
H	3.02750	0.53180	−0.00450
H	5.13340	−2.56460	0.14470
H	2.96870	−2.60490	3.82780
H	2.26140	−1.03140	4.10820
H	5.21820	−0.52940	−1.16850
H	7.17040	−0.97180	1.75850
H	7.07960	−2.69970	1.61100
H	0.63850	−3.59180	3.53800
H	7.73930	−0.78180	−0.68250
H	9.62210	−0.93350	0.96640
H	8.19620	−2.76030	−1.75140
H	0.14790	−1.11930	5.26810
H	−1.43510	−3.72380	4.76030
H	−0.54800	−3.60320	7.09030
H	−1.31360	−2.02870	7.30520
H	−2.30330	−3.46500	7.06500
H	11.29720	−4.47810	0.17650
H	−3.63780	−1.27590	3.36660
H	−1.93130	−1.44020	2.96590
H	−2.95070	−2.88060	3.12600

Table S13. Calculated Energies (Hartrees) for diastereoisomer 4*S**,6*R**,7*R**,9*S**,10*S**,12*S** of **5**.

Entry ID	Gas Phase Energy
128.00000	−1360,038652
129.00000	−1360,036526
130.00000	−1360,03812
131.00000	−1360,037303
132.00000	−1360,0373
133.00000	−1360,037053
134.00000	−1360,040298
135.00000	−1360,034155
136.00000	−1360,034866
137.00000	−1360,037188
138.00000	−1360,037237
139.00000	−1360,039209
140.00000	−1360,036278
141.00000	−1360,036456
142.00000	−1360,040141

Table S14. Coordinates (Angstroms) of calculated geometries for diastereoisomer 4*S**,6*R**,7*R**,9*S**,10*S**,12*S** of **5**.

**12-Acetoxy-marilzafurenyne_128**			
C	0.67890	0.89490	−1.54770
O	1.37750	0.07050	−0.61860
C	−0.80300	0.48390	−1.68790
C	1.52610	0.88920	−2.82610
C	2.68170	−0.23400	−1.11610
C	−1.08370	−0.89030	−2.33240
Br	−1.72640	1.87200	−2.77910
C	2.92730	0.74690	−2.27100
C	3.72540	−0.22940	0.01230
C	−0.58650	−2.10320	−1.51650
Cl	3.51790	2.33870	−1.70380
C	3.47800	−1.33750	1.01550
C	−1.36890	−2.26450	−0.22730
O	−0.70600	−3.28890	−2.29100
C	4.35200	−2.31770	1.29830
C	−0.83460	−2.15160	0.99920
C	4.06460	−3.33640	2.25140
C	3.85490	−4.21810	3.06970
C	−1.61020	−2.31020	2.28920
C	0.27020	−3.63130	−3.15000
C	−0.00580	−4.95110	−3.85200
O	1.28460	−2.97520	−3.38720
H	0.70800	1.90160	−1.12740
H	−1.26460	0.51840	−0.69990

H	1.30930	0.02340	−3.44970
H	1.38940	1.78390	−3.43410
H	2.63290	−1.23680	−1.54430
H	−2.15920	−0.99410	−2.47880
H	−0.66210	−0.91150	−3.33560
H	3.63970	0.36510	−3.00320
H	4.72560	−0.33590	−0.40930
H	3.70640	0.72080	0.54640
H	0.46100	−1.97810	−1.25000
H	2.52140	−1.30370	1.51870
H	−2.42130	−2.48410	−0.33500
H	5.31180	−2.36370	0.80410
H	0.21930	−1.93020	1.09970
H	3.66920	−4.98680	3.78650
H	−2.66090	−2.53270	2.09970
H	−1.18980	−3.12270	2.88250
H	−1.55430	−1.39340	2.87650
H	0.81880	−5.20630	−4.51720
H	−0.91960	−4.88100	−4.44120
H	−0.12190	−5.74980	−3.11990
**12-Acetoxy-marilzafurenyne_129**			
C	0.87100	0.50210	−0.91300
O	1.42880	−0.73050	−0.46260
C	−0.64600	0.35510	−1.14820
C	1.71060	0.93170	−2.12730
C	2.76180	−0.86810	−0.94550
C	−1.07510	−0.64260	−2.24700
Br	−1.37410	2.14960	−1.61800
C	2.65200	−0.23860	−2.33680
C	3.73440	−0.17350	0.03080
C	−0.80700	−2.13100	−1.92950
Cl	4.21560	0.25570	−3.04390
C	3.68620	−0.79650	1.41180
C	−1.52870	−2.58170	−0.67320
O	−1.23760	−2.93430	−3.02070
C	4.74630	−1.31990	2.04980
C	−0.91140	−3.00210	0.44300
C	4.63800	−1.89430	3.34880
C	4.50780	−2.39060	4.45680
C	−1.62600	−3.45420	1.69820
C	−0.41450	−3.17430	−4.05420
C	−1.04010	−4.08220	−5.10060
O	0.72190	−2.72310	−4.18990
H	1.02350	1.23220	−0.11630
H	−1.11720	0.07860	−0.20390
H	1.12610	1.15720	−3.01870
H	2.27350	1.83120	−1.87540

H	3.02170	−1.92480	−1.02340
H	−2.14510	−0.52220	−2.41910
H	−0.59730	−0.37400	−3.18830
H	2.19830	−0.95560	−3.02070
H	4.75410	−0.23550	−0.35040
H	3.50350	0.88750	0.12460
H	0.25910	−2.29500	−1.77450
H	2.71410	−0.80250	1.88670
H	−2.60820	−2.55260	−0.70980
H	5.72450	−1.32150	1.59080
H	0.16990	−3.02640	0.47510
H	4.39070	−2.82370	5.42530
H	−1.33810	−2.82570	2.54140
H	−2.70920	−3.39950	1.58390
H	−1.35810	−4.48470	1.93260
H	−0.33270	−4.27530	−5.90680
H	−1.92930	−3.61470	−5.52250
H	−1.32450	−5.03380	−4.65230
**12-Acetoxy-marilzafurenyne_130**			
C	0.73150	0.65000	−1.36910
O	0.83640	−0.25320	−0.27140
C	−0.65700	0.58650	−2.04170
C	1.95070	0.36810	−2.25630
C	2.11020	−0.89650	−0.28600
C	−1.01710	−0.72590	−2.76990
Br	−0.77020	2.07230	−3.36460
C	2.98320	−0.06170	−1.23540
C	2.63500	−1.11000	1.14320
C	−1.13760	−1.96470	−1.85650
Cl	3.67870	1.38410	−0.43990
C	3.93420	−1.89100	1.17070
C	−2.30730	−1.84560	−0.89850
O	−1.29340	−3.13440	−2.64860
C	5.09220	−1.41820	1.66040
C	−2.18330	−1.77810	0.43710
C	6.28690	−2.19360	1.65920
C	7.30280	−2.87000	1.62250
C	−3.34470	−1.66050	1.40040
C	−0.20980	−3.76990	−3.12800
C	−0.56640	−5.02070	−3.91500
O	0.95890	−3.41070	−2.98360
H	0.84830	1.64730	−0.94160
H	−1.41460	0.79630	−1.28520
H	1.76820	−0.45930	−2.94030
H	2.25850	1.23130	−2.84710
H	1.95820	−1.87370	−0.74760
H	−1.96700	−0.58820	−3.28710

H	−0.29090	−0.91090	−3.55970
H	3.80340	−0.63540	−1.66840
H	2.75500	−0.15570	1.65680
H	1.89620	−1.67010	1.71670
H	−0.23770	−2.08000	−1.25640
H	3.89240	−2.89100	0.76010
H	−3.28920	−1.81650	−1.34830
H	5.15160	−0.42070	2.07250
H	−1.19670	−1.80440	0.88060
H	8.19210	−3.45960	1.59330
H	−3.25620	−0.74470	1.98560
H	−4.30000	−1.64060	0.87490
H	−3.34980	−2.50660	2.08800
H	−1.17180	−4.76030	−4.78270
H	−1.13080	−5.71140	−3.28920
H	0.33760	−5.52260	−4.25920
**12-Acetoxy-marilzafurenyne_131**			
C	0.73380	0.15060	−0.99520
O	1.90630	−0.55960	−0.60260
C	0.34400	−0.20430	−2.44340
C	1.02880	1.63510	−0.74610
C	2.87510	0.34570	−0.08180
C	−0.00600	−1.69320	−2.63650
Br	1.85770	0.22390	−3.67220
C	2.07070	1.57720	0.35160
C	3.73200	−0.32280	1.00510
C	−1.24360	−2.18160	−1.85350
Cl	1.26410	1.34020	1.93140
C	4.58850	−1.43690	0.43810
C	−2.48250	−1.37120	−2.18710
O	−1.48850	−3.54620	−2.16770
C	5.92630	−1.50060	0.54530
C	−3.18780	−0.66110	−1.29130
C	6.67890	−2.57440	−0.01100
C	7.29450	−3.50390	−0.50920
C	−4.42240	0.14890	−1.62360
C	−0.93680	−4.52570	−1.43260
C	−1.33000	−5.91100	−1.92030
O	−0.18920	−4.37270	−0.46850
H	−0.06290	−0.14590	−0.31240
H	−0.49680	0.41550	−2.75710
H	1.46310	2.10370	−1.62910
H	0.14260	2.20710	−0.47010
H	3.51900	0.64060	−0.91280
H	−0.16840	−1.87750	−3.69880
H	0.85920	−2.29590	−2.35690
H	2.67910	2.48000	0.41410

H	4.36900	0.42010	1.48620
H	3.09670	−0.74780	1.78240
H	−1.06280	−2.07960	−0.78230
H	4.04850	−2.21720	−0.08230
H	−2.79400	−1.38780	−3.22180
H	6.47810	−0.72940	1.06300
H	−2.87720	−0.64900	−0.25530
H	7.82860	−4.31810	−0.94630
H	−5.27620	−0.21570	−1.05180
H	−4.26480	1.19780	−1.37090
H	−4.66950	0.08540	−2.68400
H	−0.98420	−6.06290	−2.94230
H	−2.41330	−6.02550	−1.89650
H	−0.88510	−6.67650	−1.28490
**12-Acetoxy-marilzafurenyne_132**			
C	0.73390	0.15040	−0.99510
O	1.90640	−0.55980	−0.60250
C	0.34420	−0.20430	−2.44340
C	1.02890	1.63490	−0.74590
C	2.87510	0.34550	−0.08150
C	−0.00600	−1.69320	−2.63670
Br	1.85800	0.22380	−3.67200
C	2.07070	1.57700	0.35180
C	3.73200	−0.32300	1.00550
C	−1.24360	−2.18160	−1.85390
Cl	1.26390	1.33990	1.93160
C	4.58880	−1.43680	0.43860
C	−2.48250	−1.37090	−2.18720
O	−1.48870	−3.54610	−2.16860
C	5.92650	−1.50030	0.54620
C	−3.18770	−0.66120	−1.29110
C	6.67960	−2.57390	−0.01010
C	7.29540	−3.50280	−0.50900
C	−4.42230	0.14900	−1.62320
C	−0.93750	−4.52590	−1.43360
C	−1.33080	−5.91090	−1.92180
O	−0.19020	−4.37340	−0.46920
H	−0.06280	−0.14620	−0.31240
H	−0.49660	0.41560	−2.75710
H	1.46320	2.10360	−1.62880
H	0.14260	2.20680	−0.47000
H	3.51910	0.64050	−0.91240
H	−0.16830	−1.87730	−3.69900
H	0.85920	−2.29600	−2.35710
H	2.67900	2.47980	0.41440
H	4.36870	0.42000	1.48680
H	3.09660	−0.74810	1.78260

H	−1.06290	−2.07990	−0.78260
H	4.04910	−2.21720	−0.08210
H	−2.79390	−1.38700	−3.22200
H	6.47810	−0.72890	1.06400
H	−2.87730	−0.64970	−0.25520
H	7.82970	−4.31650	−0.94670
H	−4.26460	1.19780	−1.37000
H	−4.66930	0.08610	−2.68360
H	−5.27620	−0.21580	−1.05160
H	−0.88460	−6.67680	−1.28770
H	−0.98660	−6.06190	−2.94450
H	−2.41400	−6.02600	−1.89640
**12-Acetoxy-marilzafurenyne_133**			
C	0.82630	0.95160	−1.55130
O	1.53420	0.13560	−0.62290
C	−0.67520	0.59900	−1.59440
C	1.60340	0.84510	−2.86820
C	2.81530	−0.21280	−1.15260
C	−1.04390	−0.78820	−2.16180
Br	−1.60190	1.97590	−2.69660
C	3.02690	0.69300	−2.37490
C	3.89850	−0.15270	−0.06410
C	−0.49110	−2.00060	−1.37920
Cl	3.68900	2.29670	−1.93370
C	3.68680	−1.20820	1.00290
C	−1.06070	−2.07280	0.03120
O	−0.76130	−3.19510	−2.10750
C	4.59510	−2.13870	1.34290
C	−1.89880	−3.01600	0.49790
C	4.34420	−3.10810	2.35640
C	4.11550	−3.96430	3.19770
C	−2.43880	−3.05370	1.91190
C	0.08760	−3.60370	−3.06710
C	−0.33890	−4.91650	−3.70410
O	1.10160	−3.00890	−3.43350
H	0.92080	1.97410	−1.18180
H	−1.08250	0.69930	−0.58800
H	1.32760	−0.04900	−3.42550
H	1.46050	1.70670	−3.52080
H	2.74130	−1.23880	−1.51780
H	−2.13070	−0.87120	−2.19480
H	−0.72590	−0.84150	−3.20190
H	3.69050	0.24880	−3.11770
H	4.88390	−0.28120	−0.51390
H	3.89650	0.82400	0.42040
H	0.58790	−1.91760	−1.26510
H	2.72800	−1.17850	1.50390

H	−0.73610	−1.28530	0.69660
H	5.55670	−2.17960	0.85130
H	−2.22960	−3.81380	−0.15270
H	3.91140	−4.70910	3.93560
H	−2.14570	−3.98290	2.40170
H	−2.06220	−2.22050	2.50660
H	−3.52810	−3.00180	1.90020
H	0.37540	−5.21570	−4.47100
H	−1.32050	−4.81050	−4.16520
H	−0.38940	−5.70210	−2.95050
**12-Acetoxy-marilzafurenyne_134**			
C	1.54030	0.23950	−1.80740
O	2.26070	−0.01950	−0.60330
C	0.72650	−0.99650	−2.22900
C	2.57590	0.71820	−2.83250
C	3.63740	0.30180	−0.77990
C	−0.35990	−1.34570	−1.19550
Br	1.94420	−2.56430	−2.45070
C	3.66240	1.29440	−1.94840
C	4.26730	0.78590	0.53610
C	−1.32210	−2.45390	−1.65470
Cl	3.20740	2.94950	−1.44220
C	4.31820	−0.31750	1.57340
C	−2.28140	−2.84000	−0.54330
O	−2.04790	−1.98140	−2.78390
C	5.43710	−0.73400	2.18970
C	−2.40430	−4.08270	−0.04920
C	5.42290	−1.77930	3.15710
C	5.37990	−2.68950	3.97000
C	−3.35840	−4.47300	1.05910
C	−2.36600	−2.81780	−3.78720
C	−3.14610	−2.12560	−4.89360
O	−2.08020	−4.01200	−3.85570
H	0.86040	1.06760	−1.60060
H	0.26320	−0.80650	−3.19790
H	2.98110	−0.11930	−3.40060
H	2.17240	1.43780	−3.54520
H	4.14270	−0.61060	−1.10320
H	0.12440	−1.64460	−0.26480
H	−0.93870	−0.45170	−0.96080
H	4.63420	1.34560	−2.44050
H	5.27450	1.16040	0.35060
H	3.69240	1.61400	0.95100
H	−0.73360	−3.33450	−1.91750
H	3.36750	−0.77810	1.80780
H	−2.89300	−2.04320	−0.14510
H	6.39330	−0.28300	1.96670

H	−1.79360	−4.87830	−0.45420
H	5.33870	−3.48630	4.67900
H	−4.05570	−5.23240	0.70430
H	−2.80570	−4.88390	1.90430
H	−3.93490	−3.61660	1.41060
H	−3.42350	−2.84250	−5.66610
H	−4.05510	−1.67940	−4.49140
H	−2.54180	−1.34110	−5.34800
**12-Acetoxy-marilzafurenyne_135**			
C	0.75830	0.23310	−1.05720
O	2.01530	−0.29450	−1.47970
C	−0.26550	0.10350	−2.20180
C	1.03230	1.67260	−0.59640
C	3.08000	0.48190	−0.94230
C	−0.48780	−1.35120	−2.66130
Br	0.35610	1.15460	−3.78010
C	2.48110	1.88960	−0.98890
C	3.44010	−0.05000	0.46130
C	−1.05420	−2.29420	−1.57870
Cl	3.30830	3.07580	0.05900
C	3.93800	−1.48110	0.41180
C	−2.38510	−1.80340	−1.03980
O	−1.22710	−3.59320	−2.12900
C	5.12390	−1.89060	0.89270
C	−2.61260	−1.48980	0.24620
C	5.54210	−3.25020	0.81940
C	5.88880	−4.41700	0.72170
C	−3.93840	−0.99640	0.78420
C	−0.23590	−4.49760	−2.06080
C	−0.61640	−5.82740	−2.69190
O	0.87260	−4.31830	−1.55940
H	0.42140	−0.33820	−0.19170
H	−1.21600	0.54000	−1.89200
H	0.36800	2.41240	−1.04380
H	0.91900	1.73920	0.48620
H	3.95470	0.41550	−1.59070
H	−1.16220	−1.35000	−3.51800
H	0.45890	−1.75100	−3.02690
H	2.53080	2.26450	−2.01230
H	4.21000	0.57670	0.91220
H	2.57900	−0.01000	1.12780
H	−0.35970	−2.34530	−0.73870
H	3.26600	−2.19440	−0.04850
H	−3.18340	−1.71160	−1.76260
H	5.80530	−1.19110	1.35530
H	−1.81430	−1.58710	0.96940
H	6.18610	−5.43810	0.63250

H	−3.82000	−0.00990	1.23330
H	−4.68900	−0.92600	−0.00390
H	−4.31050	−1.67790	1.54980
H	−1.49690	−6.23970	−2.20020
H	0.20210	−6.54040	−2.59510
H	−0.83720	−5.69280	−3.75040
**12-Acetoxy-marilzafurenyne_136**			
C	1.02140	0.52730	−0.87620
O	1.57830	−0.69450	−0.39640
C	−0.50890	0.40270	−1.01460
C	1.79740	0.87600	−2.15700
C	2.88570	−0.87490	−0.93390
C	−1.02400	−0.62240	−2.04890
Br	−1.23210	2.19320	−1.50540
C	2.71530	−0.31610	−2.34950
C	3.91360	−0.14410	−0.04400
C	−0.68800	−2.10550	−1.76580
Cl	4.24810	0.12040	−3.15520
C	3.93080	−0.69490	1.36810
C	−1.18850	−2.56060	−0.40120
O	−1.23590	−2.91340	−2.80430
C	5.01910	−1.19230	1.97900
C	−2.18240	−3.43800	−0.17500
C	4.97420	−1.69760	3.31000
C	4.89820	−2.13120	4.44890
C	−2.64290	−3.86820	1.20130
C	−0.54450	−3.12500	−3.93630
C	−1.27610	−4.03910	−4.90590
O	0.55510	−2.64510	−4.20860
H	1.23530	1.29160	−0.12720
H	−0.92980	0.17310	−0.03520
H	1.16720	1.05660	−3.02740
H	2.38080	1.78190	−1.98870
H	3.13000	−1.93750	−0.96910
H	−2.10890	−0.52880	−2.10610
H	−0.65720	−0.34910	−3.03750
H	2.22190	−1.06140	−2.97360
H	4.91200	−0.23860	−0.47230
H	3.69940	0.92330	0.00630
H	0.39230	−2.24830	−1.75530
H	2.98370	−0.66910	1.89050
H	−0.67050	−2.12920	0.44360
H	5.97300	−1.22480	1.47230
H	−2.70900	−3.88410	−1.00720
H	4.82850	−2.50990	5.44440
H	−2.53030	−4.94670	1.31510
H	−2.06640	−3.37950	1.98750

H	−3.69470	−3.61740	1.34170
H	−0.67960	−4.19330	−5.80490
H	−2.23040	−3.59830	−5.19290
H	−1.46310	−5.00740	−4.44260
**12-Acetoxy-marilzafurenyne_137**			
C	0.66330	−0.46580	−0.69760
O	1.88490	−1.01110	−1.19280
C	−0.18370	0.10480	−1.85200
C	1.05360	0.54840	0.38570
C	2.98820	−0.44500	−0.49470
C	−0.60830	−0.95900	−2.88400
Br	0.85620	1.50570	−2.82200
C	2.40110	0.02920	0.84180
C	4.16360	−1.43360	−0.42500
C	−1.53430	−2.06720	−2.33830
Cl	2.15920	−1.31690	1.99720
C	5.38340	−0.83350	0.24630
C	−2.79820	−1.49770	−1.72090
O	−1.89460	−2.94190	−3.39940
C	5.93150	−1.30270	1.37940
C	−3.16060	−1.67130	−0.43930
C	7.07680	−0.69890	1.97270
C	8.04370	−0.14680	2.47390
C	−4.42020	−1.10210	0.17760
C	−1.15270	−4.02850	−3.67000
C	−1.71030	−4.83980	−4.82870
O	−0.12880	−4.37010	−3.08110
H	0.12570	−1.27890	−0.20920
H	−1.06790	0.59800	−1.44630
H	1.17640	1.54660	−0.03440
H	0.32230	0.61650	1.19150
H	3.29990	0.43330	−1.06350
H	−1.11080	−0.45910	−3.71240
H	0.28810	−1.41360	−3.30800
H	3.01380	0.78830	1.32920
H	3.86510	−2.35090	0.08300
H	4.44600	−1.72750	−1.43610
H	−1.01400	−2.62970	−1.56150
H	5.81540	0.02880	−0.24350
H	−3.42890	−0.92040	−2.38210
H	5.51090	−2.16250	1.88160
H	−2.53070	−2.25290	0.21990
H	8.89090	0.33240	2.91220
H	−4.16850	−0.44330	1.00920
H	−4.99900	−0.53070	−0.54900
H	−5.04940	−1.90730	0.55800
H	−1.09550	−5.72250	−5.00330

H	−1.72300	−4.23930	−5.73770
H	−2.72730	−5.16240	−4.60780
**12-Acetoxy-marilzafurenyne_138**			
C	1.52740	0.02960	−1.20660
O	2.60470	−0.78600	−0.74290
C	0.53620	−0.83040	−2.01090
C	2.16970	1.17820	−1.99890
C	3.83410	−0.07110	−0.81930
C	−0.09600	−1.93930	−1.14990
Br	1.47190	−1.66010	−3.56760
C	3.62610	0.76480	−2.08430
C	4.04030	0.72710	0.48570
C	−1.23360	−2.69930	−1.85220
Cl	4.73010	2.16060	−2.23390
C	4.14710	−0.18320	1.69320
C	−1.72170	−3.85940	−1.00340
O	−2.30050	−1.78980	−2.09580
C	5.18470	−0.19850	2.54660
C	−1.73130	−5.13990	−1.40800
C	5.22990	−1.07810	3.66610
C	5.24060	−1.85180	4.61070
C	−2.21610	−6.30030	−0.56570
C	−3.03130	−1.88330	−3.22040
C	−4.12560	−0.83010	−3.29020
O	−2.87290	−2.70320	−4.12340
H	1.01840	0.45030	−0.33780
H	−0.24890	−0.18850	−2.41270
H	1.72660	1.33600	−2.98260
H	2.07300	2.10910	−1.43920
H	4.66300	−0.76760	−0.95250
H	0.68370	−2.64230	−0.85350
H	−0.47940	−1.50650	−0.22520
H	3.77730	0.13180	−2.95990
H	4.94630	1.32990	0.41660
H	3.21770	1.42210	0.65260
H	−0.85040	−3.10230	−2.79120
H	3.31150	−0.85270	1.84750
H	−2.08040	−3.60790	−0.01570
H	6.02690	0.46400	2.40710
H	−1.37600	−5.38670	−2.39930
H	5.24910	−2.53070	5.43430
H	−1.41270	−7.02340	−0.42280
H	−2.55620	−5.96770	0.41570
H	−3.04570	−6.80400	−1.06250
H	−4.77930	−0.90770	−2.42190
H	−4.72540	−0.96710	−4.18970
H	−3.68870	0.16770	−3.31210

**12-Acetoxy-marilzafurenyne_139**			
C	1.27840	0.78160	−1.45010
O	2.04540	0.19190	−0.40280
C	0.03500	−0.07490	−1.74800
C	2.25520	1.00350	−2.61170
C	3.42890	0.18640	−0.74980
C	0.34040	−1.53110	−2.15350
Br	−1.02980	0.81110	−3.18180
C	3.56830	1.20920	−1.88510
C	4.31220	0.41520	0.48700
C	−0.90350	−2.43520	−2.18460
Cl	3.66660	2.89030	−1.27880
C	4.20090	−0.72800	1.47550
C	−0.56550	−3.80940	−2.73350
O	−1.41400	−2.55160	−0.86180
C	5.23800	−1.46180	1.91280
C	−1.15000	−4.35740	−3.81120
C	5.07100	−2.52750	2.84290
C	4.89120	−3.44200	3.63200
C	−0.81600	−5.72680	−4.36260
C	−2.73950	−2.57680	−0.64200
C	−3.07980	−2.70970	0.83390
O	−3.61930	−2.50050	−1.49810
H	0.95080	1.75730	−1.08710
H	−0.58670	−0.07640	−0.85170
H	2.32870	0.11990	−3.24400
H	1.97660	1.84470	−3.24720
H	3.64740	−0.80000	−1.16360
H	0.81360	−1.54450	−3.13470
H	1.06420	−1.95820	−1.45820
H	4.43990	1.02750	−2.51470
H	5.35260	0.53720	0.18370
H	4.02140	1.33320	0.99820
H	−1.64290	−1.97790	−2.84430
H	3.20110	−0.93810	1.83200
H	0.19660	−4.35930	−2.20020
H	6.24250	−1.26320	1.56750
H	−1.91650	−3.80580	−4.33860
H	4.73210	−4.23900	4.32380
H	−0.03700	−6.21520	−3.77610
H	−1.70180	−6.36250	−4.35110
H	−0.46790	−5.64360	−5.39250
H	−4.16050	−2.70990	0.97470
H	−2.65580	−1.87790	1.39560
H	−2.67730	−3.64130	1.23040
**12-Acetoxy-marilzafurenyne_140**			
C	1.51820	−0.95370	−2.02560

O	2.30720	−1.01150	−0.83850
C	0.88650	−2.32600	−2.33390
C	2.43910	−0.40750	−3.12190
C	3.59400	−0.44780	−1.07840
C	−0.15090	−2.76950	−1.28270
Br	2.33540	−3.69920	−2.39500
C	3.42040	0.42720	−2.32630
C	4.13250	0.25760	0.17660
C	−1.60340	−2.33330	−1.56860
Cl	2.68620	2.01500	−1.94700
C	4.40500	−0.72350	1.29890
C	−2.55810	−2.97230	−0.57870
O	−1.75450	−0.92020	−1.50440
C	5.58850	−0.86320	1.91930
C	−3.56090	−3.79980	−0.91610
C	5.78530	−1.80580	2.96900
C	5.92460	−2.63360	3.85580
C	−4.51320	−4.43680	0.07320
C	−1.90370	−0.19520	−2.62690
C	−2.12390	1.28160	−2.34220
O	−1.84030	−0.62590	−3.77770
H	0.74570	−0.20990	−1.84170
H	0.42310	−2.31320	−3.32210
H	2.97330	−1.21580	−3.62180
H	1.90540	0.16060	−3.88440
H	4.26080	−1.27240	−1.33820
H	−0.15320	−3.85970	−1.26270
H	0.16360	−2.46070	−0.28500
H	4.35850	0.60500	−2.85290
H	5.04710	0.80020	−0.06510
H	3.41310	0.99280	0.53750
H	−1.88470	−2.70440	−2.55570
H	3.56350	−1.33670	1.59340
H	−2.39630	−2.72420	0.46070
H	6.43730	−0.25720	1.63680
H	−3.72430	−4.04530	−1.95650
H	6.04300	−3.35900	4.62970
H	−5.53880	−4.13910	−0.14670
H	−4.45130	−5.52360	0.00950
H	−4.28340	−4.14040	1.09720
H	−2.36140	1.81410	−3.26280
H	−1.22440	1.71890	−1.90940
H	−2.94840	1.41270	−1.64180
**12-Acetoxy-marilzafurenyne_141**			
C	0.86200	0.70380	−1.35280
O	1.00570	−0.13400	−0.20890
C	−0.56920	0.65350	−1.92810

C	2.00680	0.31610	−2.29690
C	2.26070	−0.81370	−0.25520
C	−1.02030	−0.68560	−2.54900
Br	−0.71830	2.05860	−3.33290
C	3.09250	−0.07880	−1.31800
C	2.87260	−0.93970	1.14950
C	−1.08310	−1.88680	−1.57880
Cl	3.88630	1.39290	−0.67680
C	4.14780	−1.76000	1.15170
C	−2.13820	−1.69200	−0.49850
O	−1.31690	−3.08010	−2.32130
C	5.34910	−1.29200	1.52910
C	−3.26620	−2.41040	−0.35670
C	6.51820	−2.10510	1.50910
C	7.51020	−2.81520	1.45710
C	−4.28730	−2.18720	0.73820
C	−0.28200	−3.75330	−2.85440
C	−0.71720	−5.01860	−3.57600
O	0.90090	−3.41600	−2.80160
H	1.04370	1.72040	−0.99970
H	−1.26770	0.94240	−1.14230
H	1.75030	−0.54970	−2.90560
H	2.30090	1.12400	−2.96750
H	2.05520	−1.81710	−0.63200
H	−2.01040	−0.54960	−2.98530
H	−0.37210	−0.92190	−3.39130
H	3.86260	−0.71070	−1.76200
H	3.05350	0.04470	1.58190
H	2.15800	−1.43210	1.80930
H	−0.13680	−2.00090	−1.05530
H	4.05000	−2.78520	0.82080
H	−1.92860	−0.90310	0.20960
H	5.46450	−0.26960	1.86070
H	−3.49030	−3.20660	−1.05280
H	8.37880	−3.43410	1.41440
H	−3.99360	−1.37160	1.39990
H	−5.25730	−1.94340	0.30410
H	−4.39850	−3.09080	1.33820
H	0.14980	−5.54470	−3.97500
H	−1.38440	−4.77140	−4.40130
H	−1.24120	−5.68270	−2.88920
**12-Acetoxy-marilzafurenyne_142**			
C	1.64370	−0.53600	−1.42960
O	1.84750	−0.77880	−0.03780
C	0.57230	−1.48490	−1.99500
C	3.02360	−0.64380	−2.09190
C	3.22850	−1.00970	0.21680

C	−0.80520	−1.23970	−1.35260
Br	1.11230	−3.38160	−1.67720
C	3.95740	−0.31970	−0.94440
C	3.60980	−0.56470	1.63820
C	−1.94880	−2.02760	−2.01320
Cl	4.04620	1.45600	−0.73430
C	5.05840	−0.86680	1.96810
C	−3.24980	−1.84410	−1.25280
O	−2.09720	−1.56950	−3.35220
C	5.97790	0.06650	2.26450
C	−3.96770	−2.85140	−0.72980
C	7.32900	−0.27040	2.56340
C	8.48620	−0.58600	2.79200
C	−5.26480	−2.67400	0.02970
C	−2.41530	−2.43010	−4.33490
C	−2.53540	−1.74670	−5.68770
O	−2.59490	−3.64050	−4.21050
H	1.30080	0.49540	−1.52630
H	0.50350	−1.34410	−3.07440
H	3.21560	−1.66010	−2.43610
H	3.14210	0.02320	−2.94610
H	3.38690	−2.08650	0.13040
H	−0.74920	−1.49540	−0.29350
H	−1.04100	−0.17570	−1.39380
H	4.97050	−0.69490	−1.09300
H	3.40400	0.49680	1.77760
H	2.98380	−1.09060	2.35940
H	−1.69160	−3.08800	−1.99620
H	5.33780	−1.91170	1.95350
H	−3.59660	−0.82670	−1.14280
H	5.71530	1.11490	2.28200
H	−3.61940	−3.86870	−0.84650
H	9.49750	−0.86010	2.99570
H	−6.07080	−3.20880	−0.47350
H	−5.16740	−3.07320	1.03960
H	−5.54510	−1.62260	0.10260
H	−2.83550	−2.46600	−6.44940
H	−3.28230	−0.95450	−5.64470
H	−1.57940	−1.31180	−5.97740

Table S15. Calculated Energies (Hartrees) for diastereoisomer 4*S**,6*R**,7*R**,9*S**,10*S**,12*R** of **5**.

**Entry ID**	**Gas Phase Energy**
253.00000	−1360,03979
254.00000	−1360,037257
255.00000	−1360,037251
256.00000	−1360,03904
257.00000	−1360,039494
258.00000	−1360,039494
259.00000	−1360,038818
260.00000	−1360,038822
261.00000	−1360,036139
262.00000	−1360,035976
263.00000	−1360,038559
264.00000	−1360,03859
265.00000	−1360,03438
266.00000	−1360,031558
267.00000	−1360,038532
268.00000	−1360,034039
269.00000	−1360,037011
270.00000	−1360,035359

Table S16. Coordinates (Angstroms) of calculated geometries for diastereoisomer 4*S**,6*R**,7*R**,9*S**,10*S**,12*R** of **5**.

**12-Acetoxy-marilzafurenyne_253**			
C	1.75270	2.49080	−1.34450
O	1.90870	1.24240	−0.67160
C	0.27890	2.71040	−1.74160
C	2.74520	2.47640	−2.51330
C	2.93590	0.47770	−1.29540
C	−0.67110	2.79190	−0.53150
Br	−0.33620	1.19910	−2.89150
C	3.80400	1.50850	−2.02670
C	3.65740	−0.41690	−0.27470
C	−0.47000	4.01720	0.38470
Cl	4.92870	2.35340	−0.92040
C	2.73960	−1.48670	0.28160
C	−1.26920	3.86520	1.66440
O	−0.89080	5.20520	−0.27800
C	2.99030	−2.80590	0.23580
C	−0.73280	3.84320	2.89570
C	2.08700	−3.76620	0.77530
C	1.28500	−4.56410	1.23470
C	−1.53040	3.69200	4.17330
C	0.00260	6.04100	−0.83200
C	−0.64750	7.28300	−1.42020

O	1.21590	5.85520	−0.90530
H	2.07620	3.26670	−0.65110
H	0.19130	3.61510	−2.34290
H	2.28260	2.08010	−3.41710
H	3.13900	3.46560	−2.74890
H	2.45740	−0.15270	−2.04750
H	−1.70240	2.78470	−0.88550
H	−0.55250	1.88060	0.05650
H	4.38980	1.07170	−2.83600
H	4.52610	−0.88490	−0.73890
H	4.02620	0.17940	0.56000
H	0.57660	4.09060	0.68150
H	1.82890	−1.12410	0.73980
H	−2.33920	3.77200	1.54600
H	3.89580	−3.18260	−0.21760
H	0.33800	3.93780	3.01330
H	0.58110	−5.25860	1.63650
H	−2.59820	3.60330	3.97020
H	−1.20980	2.80100	4.71390
H	−1.37570	4.55760	4.81800
H	−1.18670	7.82810	−0.64600
H	−1.34950	7.00590	−2.20610
H	0.10940	7.94170	−1.84570
**12-Acetoxy-marilzafurenyne_254**			
C	1.70970	2.40500	−1.53540
O	1.39530	1.01770	−1.40830
C	0.46840	3.17380	−2.03110
C	2.92760	2.48390	−2.46820
C	2.51160	0.22460	−1.79730
C	−0.73970	3.06390	−1.08120
Br	−0.08810	2.46260	−3.81050
C	3.08810	1.05400	−2.94710
C	3.44260	0.02310	−0.58260
C	−0.54730	3.71360	0.30470
Cl	4.77660	0.65290	−3.36820
C	2.74700	−0.71410	0.54450
C	−1.68510	3.33980	1.23460
O	−0.50230	5.13150	0.18210
C	3.19100	−1.85450	1.09880
C	−1.53090	2.68160	2.39520
C	2.49190	−2.50190	2.15770
C	1.86410	−3.03220	3.06080
C	−2.66620	2.30820	3.32410
C	0.66470	5.79390	0.24110
C	0.48420	7.30120	0.16090
O	1.78080	5.28420	0.32410
H	2.01420	2.77240	−0.55580

H	0.72310	4.22300	−2.18180
H	2.80470	3.18520	−3.29380
H	3.80420	2.79550	−1.89900
H	2.17110	−0.74690	−2.15800
H	−1.61240	3.50880	−1.56010
H	−0.97880	2.00750	−0.95150
H	2.48080	0.89690	−3.83970
H	4.32900	−0.53820	−0.87970
H	3.79640	0.97960	−0.19830
H	0.36530	3.33450	0.76560
H	1.83250	−0.26050	0.90290
H	−2.67400	3.63730	0.91630
H	4.10270	−2.32050	0.75340
H	−0.54080	2.38550	2.71380
H	1.31260	−3.49570	3.84840
H	−3.62860	2.64110	2.93360
H	−2.70560	1.22640	3.45440
H	−2.51770	2.76550	4.30260
H	0.00870	7.57540	−0.78030
H	−0.14070	7.64960	0.98280
H	1.44990	7.80260	0.22220
**12-Acetoxy-marilzafurenyne_255**			
C	1.70880	2.40560	−1.53310
O	1.39410	1.01850	−1.40430
C	0.46820	3.17480	−2.03020
C	2.92700	2.48310	−2.46560
C	2.50920	0.22430	−1.79500
C	−0.74070	3.06720	−1.08110
Br	−0.08810	2.46220	−3.80910
C	3.08510	1.05330	−2.94540
C	3.44180	0.02120	−0.58180
C	−0.54830	3.71740	0.30450
Cl	4.77260	0.65050	−3.36900
C	2.74860	−0.71920	0.54470
C	−1.68640	3.34450	1.23440
O	−0.50250	5.13520	0.18130
C	3.19480	−1.86010	1.09610
C	−1.53280	2.68630	2.39510
C	2.49780	−2.51050	2.15450
C	1.87190	−3.04300	3.05770
C	−2.66840	2.31390	3.32390
C	0.66490	5.79690	0.23970
C	0.48530	7.30430	0.15870
O	1.78080	5.28660	0.32270
H	2.01320	2.77410	−0.55380
H	0.72380	4.22360	−2.18200
H	2.80540	3.18520	−3.29070

H	3.80410	2.79270	−1.89610
H	2.16720	−0.74670	−2.15570
H	−1.61260	3.51280	−1.56080
H	−0.98110	2.01110	−0.95090
H	2.47660	0.89720	−3.83720
H	4.32830	−0.53860	−0.88100
H	3.79500	0.97730	−0.19600
H	0.36410	3.33800	0.76590
H	1.83400	−0.26740	0.90520
H	−2.67510	3.64280	0.91600
H	4.10660	−2.32420	0.74860
H	−0.54290	2.38940	2.71370
H	1.32210	−3.50820	3.84540
H	−2.51960	2.77120	4.30240
H	−3.63050	2.64740	2.93330
H	−2.70860	1.23210	3.45440
H	1.45120	7.80520	0.22100
H	0.01110	7.57840	−0.78320
H	−0.14040	7.65330	0.97970
**12-Acetoxy-marilzafurenyne_256**			
C	1.79860	2.47060	−1.46600
O	1.96410	1.16100	−0.92440
C	0.31850	2.72930	−1.81280
C	2.76860	2.57240	−2.64950
C	2.96170	0.45410	−1.65440
C	−0.60870	2.70000	−0.58270
Br	−0.32360	1.33470	−3.08860
C	3.82610	1.54870	−2.29160
C	3.69820	−0.55730	−0.76020
C	−0.39070	3.83980	0.43460
Cl	4.98330	2.26140	−1.12730
C	2.79410	−1.69080	−0.31640
C	−1.17680	3.58150	1.70530
O	−0.81280	5.08250	−0.11770
C	2.56410	−2.02080	0.96540
C	−0.62860	3.46100	2.92560
C	1.70320	−3.09620	1.32780
C	0.95840	−4.02440	1.60160
C	−1.41330	3.20380	4.19420
C	0.07930	5.96370	−0.59900
C	−0.57170	7.25400	−1.07050
O	1.29200	5.78380	−0.69480
H	2.13630	3.17320	−0.70450
H	0.22330	3.68660	−2.32480
H	2.28390	2.27980	−3.58080
H	3.16860	3.57770	−2.78590
H	2.45400	−0.08190	−2.45870

H	−1.64620	2.72320	−0.91710
H	−0.47930	1.74000	−0.08060
H	4.38970	1.19510	−3.15540
H	4.53590	−0.98950	−1.30780
H	4.11600	−0.06050	0.11580
H	0.65970	3.88200	0.72390
H	2.31670	−2.25130	−1.10850
H	−2.24780	3.49520	1.59010
H	3.03070	−1.47000	1.76950
H	0.44320	3.54880	3.04020
H	0.30470	−4.83410	1.83870
H	−1.25390	4.01420	4.90600
H	−2.48290	3.12910	3.99470
H	−1.08570	2.27250	4.65690
H	−1.10560	7.72860	−0.24760
H	−1.27860	7.04900	−1.87390
H	0.18400	7.94700	−1.43980
**12-Acetoxy-marilzafurenyne_257**			
C	1.40610	2.34150	−0.94110
O	0.93870	1.07600	−0.47420
C	0.32840	3.03320	−1.80030
C	2.73450	2.07230	−1.65940
C	1.86380	0.05600	−0.83310
C	−0.95930	3.35460	−1.01790
Br	−0.17190	1.84900	−3.32760
C	3.20130	0.78880	−1.00450
C	1.82990	−1.10030	0.17950
C	−0.80870	4.41240	0.09550
Cl	3.97590	1.16680	0.56470
C	2.74960	−2.23940	−0.21480
C	−2.05570	4.46410	0.95670
O	−0.59190	5.70050	−0.47150
C	3.79650	−2.65510	0.51700
C	−2.07610	4.22170	2.27770
C	4.63330	−3.72900	0.09890
C	5.35010	−4.63560	−0.29520
C	−3.32060	4.27390	3.13770
C	0.63870	6.23620	−0.51960
C	0.63980	7.64440	−1.09210
O	1.68140	5.68100	−0.17770
H	1.62730	2.94200	−0.05910
H	0.73520	3.94630	−2.23460
H	2.57820	1.89810	−2.72390
H	3.44600	2.89250	−1.55880
H	1.55130	−0.32100	−1.80880
H	−1.72770	3.68810	−1.71590
H	−1.33480	2.42690	−0.58370

H	3.91660	0.23130	−1.60990
H	2.07730	−0.74370	1.17960
H	0.81510	−1.49360	0.24190
H	0.01290	4.13890	0.75820
H	2.52150	−2.72690	−1.15310
H	−2.97370	4.71710	0.44580
H	4.03860	−2.17750	1.45600
H	−1.15670	3.96990	2.78850
H	5.97660	−5.42970	−0.63620
H	−4.20200	4.53400	2.55050
H	−3.49530	3.30490	3.60620
H	−3.20260	5.01840	3.92540
H	0.00810	8.29620	−0.48920
H	0.26070	7.63740	−2.11360
H	1.65130	8.05020	−1.09880
**12-Acetoxy-marilzafurenyne_258**			
C	1.21930	2.24600	−1.01800
O	0.86630	1.07530	−0.28150
C	−0.03590	2.89890	−1.63140
C	2.28620	1.81250	−2.03150
C	1.62570	−0.03530	−0.74490
C	−1.04440	3.38900	−0.57470
Br	−0.97550	1.57840	−2.79700
C	2.88180	0.58760	−1.36910
C	1.84430	−1.06140	0.37900
C	−0.55960	4.55780	0.30860
Cl	4.07410	1.09670	−0.13450
C	2.58530	−2.29380	−0.10120
C	−1.51440	4.78720	1.46400
O	−0.47420	5.75490	−0.45750
C	3.78310	−2.68600	0.36350
C	−1.17170	4.70180	2.76000
C	4.43980	−3.85150	−0.12520
C	4.99230	−4.84090	−0.57980
C	−2.12460	4.93140	3.91350
C	0.70830	6.20350	−0.90940
C	0.58840	7.53270	−1.63730
O	1.78920	5.62790	−0.79700
H	1.69470	2.93020	−0.31550
H	0.25840	3.72800	−2.27460
H	1.83400	1.52560	−2.98070
H	3.01980	2.59290	−2.23640
H	1.04280	−0.50340	−1.54050
H	−1.96760	3.68630	−1.07270
H	−1.30920	2.54340	0.06180
H	3.38380	−0.08030	−2.06980
H	2.37100	−0.60670	1.21830

H	0.87680	−1.38050	0.76720
H	0.40650	4.31240	0.75070
H	2.09080	−2.87220	−0.87000
H	−2.53120	5.03590	1.19560
H	4.29100	−2.11820	1.13040
H	−0.15370	4.45410	3.02780
H	5.47630	−5.70690	−0.97370
H	−3.12740	5.17600	3.56130
H	−2.18840	4.03640	4.53290
H	−1.77070	5.75370	4.53580
H	0.17030	8.28890	−0.97340
H	−0.06240	7.43090	−2.50510
H	1.56830	7.87010	−1.97440
**12-Acetoxy-marilzafurenyne_259**			
C	1.75440	2.40910	−1.36380
O	1.98320	1.16610	−0.70150
C	0.24400	2.64050	−1.57150
C	2.58880	2.37180	−2.64990
C	2.91090	0.38180	−1.44550
C	−0.54160	2.74260	−0.25020
Br	−0.52720	1.12450	−2.61640
C	3.68980	1.39450	−2.29340
C	3.74750	−0.51440	−0.51840
C	−0.21600	3.97450	0.62120
Cl	4.95750	2.23410	−1.34980
C	2.89660	−1.56790	0.16160
C	−0.81080	3.81380	2.01370
O	−0.70340	5.16100	−0.00500
C	3.12360	−2.89050	0.09610
C	−1.71690	4.62490	2.58900
C	2.28620	−3.83490	0.75640
C	1.54060	−4.61900	1.32230
C	−2.27850	4.42510	3.98050
C	0.12270	5.97830	−0.67820
C	−0.58310	7.21950	−1.19980
O	1.31560	5.77740	−0.89850
H	2.17130	3.18850	−0.72650
H	0.08780	3.54000	−2.16670
H	2.00960	1.97400	−3.48310
H	2.96080	3.35380	−2.94390
H	2.33100	−0.24810	−2.12310
H	−1.61020	2.74350	−0.46740
H	−0.35730	1.83330	0.32340
H	4.16130	0.94270	−3.16660
H	4.54310	−0.99730	−1.08660
H	4.22850	0.08340	0.25600
H	0.86060	4.04150	0.77950

H	2.05710	−1.19020	0.73020
H	−0.44910	2.96000	2.56910
H	3.95830	−3.28240	−0.46700
H	−2.09180	5.48530	2.05240
H	0.88610	−5.30170	1.81700
H	−1.85650	3.54120	4.45990
H	−2.05560	5.29150	4.60390
H	−3.36130	4.30570	3.93550
H	0.11490	7.84870	−1.75180
H	−0.99020	7.79730	−0.37050
H	−1.39940	6.93900	−1.86460
**12-Acetoxy-marilzafurenyne_260**			
C	1.78160	2.42550	−1.34300
O	1.99460	1.17300	−0.69330
C	0.27680	2.65890	−1.58600
C	2.64890	2.40750	−2.60760
C	2.94220	0.40040	−1.42440
C	−0.54250	2.74090	−0.28390
Br	−0.46570	1.15800	−2.67270
C	3.74140	1.42600	−2.23710
C	3.75560	−0.50840	−0.48900
C	−0.24060	3.95990	0.61380
Cl	4.98350	2.25280	−1.24900
C	2.88870	−1.57230	0.15360
C	−0.87020	3.77790	1.98820
O	−0.71340	5.15520	−0.00680
C	3.11880	−2.89360	0.07510
C	−1.79100	4.58000	2.55280
C	2.26570	−3.84790	0.70010
C	1.50690	−4.64050	1.23580
C	−2.38740	4.35880	3.92650
C	0.12850	5.98300	−0.64670
C	−0.56530	7.23140	−1.16740
O	1.32680	5.78620	−0.84000
H	2.18130	3.19570	−0.68380
H	0.13500	3.56700	−2.17160
H	2.09170	2.02150	−3.46110
H	3.02720	3.39400	−2.87770
H	2.38070	−0.22010	−2.12580
H	−1.60520	2.74440	−0.52840
H	−0.37200	1.82320	0.28060
H	4.23580	0.98730	−3.10440
H	4.56620	−0.98250	−1.04340
H	4.21580	0.07850	0.30600
H	0.83150	4.02530	0.80030
H	2.03440	−1.20330	0.70570
H	−0.52210	2.91580	2.53950

H	3.96820	−3.27680	−0.47180
H	−2.15280	5.44840	2.02010
H	0.84080	−5.33050	1.70420
H	−1.97710	3.46780	4.40290
H	−2.18080	5.21560	4.56840
H	−3.46870	4.23990	3.85230
H	−1.36040	6.96040	−1.86130
H	0.14690	7.87210	−1.68700
H	−0.99900	7.79340	−0.34070
**12-Acetoxy-marilzafurenyne_261**			
C	2.15510	2.82530	−2.16110
O	2.03540	1.79740	−1.18740
C	2.07560	4.21960	−1.50880
C	1.11390	2.48670	−3.23100
C	1.33530	0.67730	−1.72750
C	0.70240	4.67110	−0.96950
Br	2.68000	5.56870	−2.84440
C	1.15930	0.97090	−3.22790
C	2.04220	−0.63790	−1.35880
C	0.05530	3.73530	0.06720
Cl	2.54800	0.41160	−4.20970
C	2.05080	−0.87600	0.13860
C	−1.26810	4.30280	0.55050
O	0.94710	3.55240	1.15880
C	3.16190	−1.06220	0.87040
C	−2.44530	3.66330	0.45610
C	3.11440	−1.27740	2.27760
C	3.03830	−1.47230	3.48060
C	−3.76630	4.22650	0.93410
C	0.92160	2.40360	1.85960
C	1.99060	2.35620	2.93900
O	0.13650	1.46990	1.69240
H	3.15000	2.71010	−2.59480
H	2.80060	4.25140	−0.69410
H	0.11870	2.81570	−2.93520
H	1.34100	2.92610	−4.20280
H	0.34370	0.68220	−1.27180
H	0.82210	5.65940	−0.52440
H	0.01320	4.80900	−1.80250
H	0.25800	0.51640	−3.64080
H	1.53390	−1.47670	−1.83470
H	3.06840	−0.63760	−1.72730
H	−0.14580	2.77780	−0.41340
H	1.08260	−0.88630	0.62130
H	−1.22840	5.28600	0.99690
H	4.13730	−1.05120	0.40590
H	−2.48000	2.67580	0.01660

H	2.96590	−1.64400	4.53140
H	−4.46990	4.29410	0.10400
H	−4.19730	3.57660	1.69610
H	−3.64510	5.22200	1.36260
H	2.05340	3.31220	3.45780
H	1.75740	1.57830	3.66560
H	2.95890	2.13250	2.49210
**12-Acetoxy-marilzafurenyne_262**			
C	1.13000	2.68570	−1.71280
O	1.58600	1.52810	−1.01710
C	−0.41160	2.73840	−1.72840
C	1.81790	2.65710	−3.08290
C	2.59670	0.86740	−1.77160
C	−1.06090	2.69220	−0.32670
Br	−1.12790	1.17800	−2.74700
C	3.10210	1.91570	−2.77220
C	3.65220	0.24470	−0.84360
C	−0.72760	3.84500	0.65150
Cl	4.28200	3.04420	−2.03810
C	3.05990	−0.84270	0.02970
C	−0.99750	5.21190	0.04960
O	0.61780	3.77940	1.10440
C	3.51270	−2.10610	0.08810
C	−1.94910	6.05600	0.48020
C	2.91210	−3.08530	0.93030
C	2.37060	−3.89880	1.66240
C	−2.21540	7.42060	−0.11830
C	0.96800	2.93130	2.08470
C	2.46180	2.95160	2.36420
O	0.20400	2.19860	2.71220
H	1.52140	3.55480	−1.18540
H	−0.74020	3.63200	−2.25930
H	1.24160	2.08540	−3.80930
H	1.98350	3.65090	−3.49950
H	2.10350	0.07400	−2.33650
H	−2.14240	2.68000	−0.46320
H	−0.82370	1.73750	0.14410
H	3.56430	1.47040	−3.65380
H	4.47350	−0.16330	−1.43360
H	4.07860	1.00380	−0.18790
H	−1.38430	3.74920	1.51840
H	2.21450	−0.54210	0.63520
H	−0.36220	5.50380	−0.77380
H	4.35610	−2.42110	−0.50930
H	−2.58290	5.76980	1.30840
H	1.89370	−4.60640	2.30360
H	−1.53650	7.63510	−0.94440

H	−3.23800	7.47490	−0.49280
H	−2.08710	8.19470	0.63880
H	2.71120	2.23700	3.14800
H	3.01160	2.68410	1.46180
H	2.77360	3.94550	2.68320
**12-Acetoxy-marilzafurenyne_263**			
C	2.22660	2.55120	−1.75330
O	2.48840	1.37090	−0.99600
C	0.85910	3.14730	−1.37510
C	2.38050	2.15790	−3.22790
C	2.83070	0.29760	−1.86790
C	0.82400	3.61020	0.09430
Br	−0.57540	1.78810	−1.66500
C	3.32590	0.97700	−3.15020
C	3.81090	−0.67440	−1.19180
C	−0.46800	4.35060	0.49730
Cl	5.00890	1.56640	−2.99870
C	3.17010	−1.39460	−0.02260
C	−0.67080	5.61710	−0.31420
O	−0.40710	4.69340	1.87580
C	3.10360	−2.73030	0.10670
C	−1.71450	5.83280	−1.13150
C	2.48540	−3.35090	1.23000
C	1.93390	−3.85160	2.19750
C	−1.91980	7.09410	−1.94260
C	−0.85750	3.84030	2.81070
C	−0.72520	4.39770	4.21900
O	−1.32610	2.72220	2.60430
H	3.01330	3.26710	−1.50950
H	0.64440	3.98790	−2.03600
H	1.42930	1.82990	−3.64730
H	2.75490	2.97020	−3.85130
H	1.90650	−0.23380	−2.10420
H	0.96020	2.73840	0.73560
H	1.67700	4.26130	0.28830
H	3.27210	0.32570	−4.02310
H	4.17160	−1.40390	−1.91770
H	4.68400	−0.13730	−0.82090
H	−1.32850	3.70380	0.31740
H	2.74740	−0.75490	0.74110
H	0.08970	6.37690	−0.20460
H	3.52190	−3.38170	−0.64700
H	−2.47470	5.07050	−1.23700
H	1.44910	−4.28480	3.04400
H	−1.11320	7.81000	−1.78040
H	−1.95820	6.85630	−3.00590
H	−2.86030	7.57060	−1.66470

H	−1.26400	5.34100	4.30320
H	0.32350	4.57080	4.45870
H	−1.13680	3.69600	4.94410
**12-Acetoxy-marilzafurenyne_264**			
C	1.25750	2.16450	−0.93830
O	0.97790	0.97700	−0.19690
C	−0.05070	2.81360	−1.43400
C	2.24180	1.76150	−2.04380
C	1.70530	−0.11700	−0.74370
C	−0.97450	3.26840	−0.28800
Br	−1.06780	1.50440	−2.54600
C	2.90030	0.53030	−1.45680
C	2.02460	−1.16250	0.33730
C	−0.43430	4.42550	0.57960
Cl	4.18580	1.02810	−0.31460
C	2.73360	−2.37790	−0.22730
C	−1.27250	4.58120	1.84120
O	−0.42050	5.63810	−0.17340
C	3.96890	−2.76710	0.12890
C	−1.96610	5.67520	2.20420
C	4.59260	−3.91630	−0.43580
C	5.11390	−4.89100	−0.95440
C	−2.78520	5.78370	3.47270
C	0.71740	6.10630	−0.71140
C	0.52630	7.44570	−1.40450
O	1.80920	5.54090	−0.69630
H	1.78210	2.84150	−0.26450
H	0.18040	3.65890	−2.08190
H	1.71610	1.48810	−2.95860
H	2.94930	2.55310	−2.29230
H	1.06250	−0.57530	−1.49780
H	−1.93880	3.56540	−0.70120
H	−1.17780	2.40400	0.34550
H	3.34860	−0.11910	−2.20950
H	2.61530	−0.71910	1.13930
H	1.09540	−1.49870	0.79750
H	0.56830	4.18510	0.93390
H	2.18200	−2.94600	−0.96410
H	−1.28990	3.71510	2.48770
H	4.53390	−2.20940	0.86260
H	−1.96220	6.55320	1.57350
H	5.57060	−5.74440	−1.40450
H	−2.74280	4.86390	4.05710
H	−2.41250	6.59990	4.09220
H	−3.82860	5.98720	3.23050
H	−0.21550	7.35840	−2.19790
H	1.46560	7.78370	−1.84170

H	0.18690	8.19440	−0.68920
**12-Acetoxy-marilzafurenyne_265**			
C	1.66630	2.37790	−1.47700
O	1.32140	1.00090	−1.31710
C	0.40150	3.19440	−1.81140
C	2.75940	2.42290	−2.55510
C	2.34480	0.17210	−1.85850
C	−0.67780	3.13160	−0.71380
Br	−0.40730	2.50610	−3.50080
C	2.79840	0.99590	−3.06590
C	3.41880	−0.08430	−0.77970
C	−0.28700	3.78880	0.63080
Cl	4.39990	0.53760	−3.71000
C	2.85340	−0.82820	0.41390
C	−1.28300	3.54180	1.75750
O	−0.14750	5.19530	0.45300
C	3.32830	−1.99550	0.87970
C	−2.47560	2.92380	1.66970
C	2.75430	−2.64890	2.00770
C	2.23320	−3.18590	2.97270
C	−3.41080	2.71390	2.84150
C	1.06420	5.77470	0.41760
C	0.98310	7.28680	0.28350
O	2.14570	5.19120	0.46040
H	2.10340	2.72640	−0.54170
H	0.67370	4.23320	−1.99920
H	2.56320	3.14190	−3.35080
H	3.71300	2.68930	−2.09780
H	1.92170	−0.78000	−2.18160
H	−1.58670	3.60530	−1.08600
H	−0.92590	2.08260	−0.54860
H	2.07390	0.87560	−3.87250
H	4.24080	−0.66400	−1.20040
H	3.84950	0.85250	−0.42660
H	0.64630	3.34720	0.98110
H	2.01000	−0.35610	0.89990
H	−0.96730	3.91530	2.72100
H	4.17040	−2.47970	0.40620
H	−2.83040	2.53850	0.72490
H	1.77460	−3.65330	3.81550
H	−3.59230	1.64940	2.99210
H	−2.99710	3.12390	3.76350
H	−4.36780	3.20020	2.65070
H	1.98290	7.72020	0.26580
H	0.47000	7.55750	−0.63880
H	0.43580	7.71060	1.12510

**12-Acetoxy-marilzafurenyne_266**			
C	1.90560	2.91810	−1.20790
O	1.60610	1.55510	−1.51390
C	0.77570	3.82500	−1.73610
C	3.29030	3.19760	−1.81160
C	2.79210	0.85770	−1.88010
C	−0.61760	3.51190	−1.14640
Br	0.64380	3.62100	−3.71730
C	3.57590	1.94580	−2.61760
C	3.45450	0.29330	−0.60600
C	−0.84020	3.83390	0.35100
Cl	5.32330	1.61400	−2.77790
C	2.57290	−0.73650	0.07110
C	−0.51360	5.27620	0.69350
O	−0.10410	2.96530	1.20140
C	2.97190	−1.96890	0.42560
C	−1.42300	6.19260	1.06340
C	2.09330	−2.89170	1.06170
C	1.30850	−3.66840	1.58230
C	−1.09600	7.62950	1.40910
C	−0.59460	1.75980	1.53190
C	0.32260	0.99500	2.47260
O	−1.66380	1.28650	1.14990
H	1.99460	3.02080	−0.12730
H	1.02790	4.86970	−1.55460
H	3.33110	4.10050	−2.42130
H	4.02350	3.30610	−1.01160
H	2.55000	0.03700	−2.55660
H	−1.35040	4.09040	−1.70940
H	−0.86890	2.46860	−1.34080
H	3.17010	2.05720	−3.62400
H	4.41160	−0.16800	−0.85040
H	3.66540	1.08900	0.10800
H	−1.90300	3.70180	0.56250
H	1.55700	−0.42240	0.26770
H	0.52780	5.55690	0.63680
H	3.98270	−2.30050	0.23700
H	−2.46620	5.91520	1.12820
H	0.61970	−4.34660	2.03420
H	−1.40150	7.84880	2.43250
H	−0.02760	7.82970	1.32140
H	−1.62710	8.30800	0.74100
H	0.16300	1.32090	3.49990
H	0.12060	−0.07430	2.41050
H	1.36630	1.16580	2.20930
**12-Acetoxy-marilzafurenyne_267**			
C	3.17280	2.65730	−0.34710

O	2.81840	1.30000	−0.09140
C	2.06140	3.59970	0.14850
C	3.52120	2.72770	−1.83960
C	3.06160	0.50280	−1.24870
C	0.67220	3.33230	−0.46920
Br	2.60570	5.48390	−0.20930
C	4.02530	1.32590	−2.11350
C	3.52900	−0.90940	−0.86190
C	−0.47290	4.13790	0.17980
Cl	5.72380	1.17730	−1.56890
C	2.44540	−1.67980	−0.13470
C	−0.63800	3.80600	1.65140
O	−1.69470	3.85950	−0.49250
C	1.95220	−2.86380	−0.53490
C	−0.49990	4.69560	2.64810
C	0.93030	−3.54100	0.19060
C	0.04580	−4.08930	0.82930
C	−0.66240	4.36700	4.11660
C	−2.04330	4.55500	−1.58740
C	−3.39810	4.13720	−2.13640
O	−1.37200	5.43100	−2.13010
H	4.08040	2.85770	0.22480
H	2.00300	3.49290	1.23240
H	2.63760	2.91720	−2.44720
H	4.25330	3.50250	−2.06920
H	2.11470	0.42770	−1.78680
H	0.70330	3.55530	−1.53470
H	0.43910	2.27000	−0.38810
H	3.98550	1.05440	−3.16890
H	3.83270	−1.45810	−1.75410
H	4.40130	−0.85680	−0.21010
H	−0.25280	5.20450	0.10580
H	2.06780	−1.21270	0.76520
H	−0.88300	2.77860	1.88030
H	2.32020	−3.34370	−1.43030
H	−0.25560	5.72320	2.41520
H	−0.72520	−4.56850	1.39080
H	0.26030	4.58380	4.65540
H	−1.46140	4.96880	4.55050
H	−0.90720	3.31480	4.26640
H	−4.17040	4.27920	−1.38110
H	−3.65310	4.73500	−3.01120
H	−3.38040	3.08690	−2.42560

**12-Acetoxy-marilzafurenyne_268**			
C	1.70340	2.13500	−2.17030
O	1.70920	1.64530	−0.83180
C	1.63070	3.67480	−2.17440
C	0.56580	1.39350	−2.89060
C	1.03350	0.39330	−0.76460
C	0.33260	4.31230	−1.63340
Br	1.89030	4.29910	−4.04850
C	−0.09630	0.60530	−1.77640
C	2.02310	−0.74450	−1.09470
C	0.04490	4.05950	−0.14180
Cl	−0.85600	−0.90230	−2.35750
C	3.16080	−0.81140	−0.09550
C	−1.24450	4.74030	0.28130
O	1.13540	4.54620	0.62920
C	3.48260	−1.90140	0.62110
C	−2.29250	4.10270	0.82800
C	4.56500	−1.90510	1.54700
C	5.49730	−1.87120	2.33490
C	−3.58050	4.77760	1.24820
C	1.48040	3.92940	1.77370
C	2.68130	4.57900	2.44250
O	0.91740	2.95540	2.27240
H	2.65070	1.83590	−2.62190
H	2.47830	4.06130	−1.60650
H	−0.13250	2.04460	−3.41560
H	0.99150	0.71590	−3.63170
H	0.62930	0.24290	0.23760
H	0.39430	5.38940	−1.79250
H	−0.51830	3.97780	−2.22570
H	−0.88110	1.20730	−1.31750
H	1.50240	−1.70240	−1.10160
H	2.44850	−0.61690	−2.08990
H	−0.08090	2.98880	0.01560
H	3.73220	0.09980	0.02220
H	−1.29250	5.80800	0.12190
H	2.92260	−2.81940	0.51540
H	−2.23890	3.03550	0.99440
H	6.31120	−1.83810	3.02450
H	−3.55450	5.84930	1.04810
H	−4.42470	4.34860	0.70790
H	−3.74870	4.63340	2.31580
H	3.55450	4.51210	1.79430
H	2.47630	5.62910	2.64840
H	2.90720	4.07730	3.38320

**12-Acetoxy-marilzafurenyne_269**			
C	1.08750	2.25010	−2.38290
O	1.89570	2.30020	−1.21250
C	0.29640	3.55850	−2.58370
C	0.28220	0.95190	−2.26800
C	1.89440	1.03370	−0.55570
C	−0.83210	3.86490	−1.57680
Br	−0.50420	3.52710	−4.40780
C	1.26780	0.04700	−1.55560
C	3.29200	0.69530	−0.01250
C	−0.39210	3.96880	−0.10570
Cl	2.45520	−0.58880	−2.73580
C	3.30170	−0.58350	0.80200
C	−1.57340	4.29360	0.79100
O	0.60800	4.97200	0.01240
C	4.01150	−1.67890	0.48450
C	−1.96920	3.53310	1.82480
C	3.98260	−2.85730	1.28400
C	3.91990	−3.86340	1.97300
C	−3.14720	3.85170	2.72010
C	1.59530	4.83670	0.91650
C	2.58920	5.98610	0.86590
O	1.72260	3.91400	1.72130
H	1.78300	2.14930	−3.21790
H	1.00630	4.38680	−2.57820
H	−0.59960	1.08460	−1.64280
H	−0.04290	0.56390	−3.23380
H	1.21290	1.12940	0.29100
H	−1.29810	4.80680	−1.86790
H	−1.61640	3.11370	−1.66540
H	0.79490	−0.80510	−1.06620
H	4.01870	0.63670	−0.82290
H	3.62720	1.50580	0.63570
H	0.00160	3.00200	0.20440
H	2.68530	−0.58050	1.69070
H	−2.10970	5.20270	0.55970
H	4.63130	−1.69810	−0.40090
H	−1.42520	2.62800	2.05820
H	3.86910	−4.74310	2.57540
H	−3.63990	4.77680	2.41890
H	−3.87970	3.04500	2.68080
H	−2.81480	3.96210	3.75260
H	3.13510	5.96770	−0.07680
H	2.06950	6.93980	0.95260
H	3.30450	5.90340	1.68390
**12-Acetoxy-marilzafurenyne_270**			
C	2.58610	2.13770	0.17390

O	2.18870	1.16020	−0.78910
C	1.72860	3.40580	0.01160
C	4.09330	2.35280	−0.03070
C	3.32820	0.47080	−1.29340
C	0.23330	3.12720	0.26050
Br	1.96100	4.14770	−1.82750
C	4.38060	1.58200	−1.30450
C	3.63700	−0.73610	−0.38190
C	−0.65580	4.38790	0.26530
Cl	6.06630	0.99850	−1.39040
C	2.50010	−1.73870	−0.36870
C	−0.25600	5.35790	1.36200
O	−2.01270	4.00990	0.45870
C	2.62630	−3.03820	−0.68560
C	0.18790	6.60640	1.14270
C	1.51970	−3.93470	−0.65540
C	0.54790	−4.67360	−0.63330
C	0.58780	7.57630	2.23380
C	−2.79650	3.72240	−0.59360
C	−4.21520	3.36330	−0.18150
O	−2.45270	3.72730	−1.77430
H	2.42780	1.72010	1.16960
H	2.08520	4.16450	0.70940
H	4.38180	3.40140	−0.10810
H	4.64270	1.91930	0.80560
H	3.13260	0.11840	−2.30700
H	−0.12260	2.43350	−0.50240
H	0.11620	2.61050	1.21360
H	4.21180	2.22540	−2.16920
H	4.54540	−1.23660	−0.71830
H	3.82160	−0.41440	0.64280
H	−0.55200	4.90910	−0.68810
H	1.53500	−1.34520	−0.07840
H	−0.34750	4.99220	2.37470
H	3.58310	−3.44720	−0.97710
H	0.27610	6.96990	0.12770
H	−0.30340	−5.31730	−0.61600
H	−0.02950	8.47360	2.18360
H	0.46980	7.13550	3.22430
H	1.63030	7.87150	2.11210
H	−4.82710	3.16740	−1.06180
H	−4.21150	2.47200	0.44520
H	−4.66390	4.18330	0.37850

© 2014 by the authors; licensee MDPI, Basel, Switzerland. This article is an open access article distributed under the terms and conditions of the Creative Commons Attribution license (http://creativecommons.org/licenses/by/3.0/).
